# Germline whole exome sequencing and large-scale replication identifies *FANCM* as a likely high grade serous ovarian cancer susceptibility gene

**DOI:** 10.18632/oncotarget.15871

**Published:** 2017-03-03

**Authors:** Ed Dicks, Honglin Song, Susan J. Ramus, Elke Van Oudenhove, Jonathan P. Tyrer, Maria P. Intermaggio, Siddhartha Kar, Patricia Harrington, David D. Bowtell, AOCS Study Group, Mine S. Cicek, Julie M. Cunningham, Brooke L. Fridley, Jennifer Alsop, Mercedes Jimenez-Linan, Anna Piskorz, Teodora Goranova, Emma Kent, Nadeem Siddiqui, James Paul, Robin Crawford, Samantha Poblete, Shashi Lele, Lara Sucheston-Campbell, Kirsten B. Moysich, Weiva Sieh, Valerie McGuire, Jenny Lester, Kunle Odunsi, Alice S. Whittemore, Natalia Bogdanova, Matthias Dürst, Peter Hillemanns, Beth Y. Karlan, Aleksandra Gentry-Maharaj, Usha Menon, Marc Tischkowitz, Douglas Levine, James D. Brenton, Thilo Dörk, Ellen L. Goode, Simon A. Gayther, D.P. Paul Pharoah

**Affiliations:** ^1^ Centre for Cancer Genetic Epidemiology, Department of Oncology, University of Cambridge, Cambridge, UK; ^2^ School of Women's and Children's Health, University of New South Wales, Sydney, Australia; ^3^ The Kinghorn Cancer Centre, Garvan Institute of Medical Research, Sydney, Australia; ^4^ Cancer Research UK Cambridge Institute, University of Cambridge, Cambridge, UK; ^5^ Peter MacCallum Cancer Centre, East Melbourne, Victoria, Australia; ^6^ Department of Biochemistry and Molecular Biology, University of Melbourne, Melbourne, Victoria, Australia; ^7^ Sir Peter MacCallum Department of Oncology, University of Melbourne, Melbourne, Victoria, Australia; ^8^ Ovarian Cancer Action Research Centre, Department of Surgery and Cancer, Imperial College London, London, UK; ^9^ Westmead Millennium Institute, Westmead Hospital, Sydney, Australia; ^10^ The QIMR Berghofer Medical Research Institute, Brisbane, Australia; ^11^ Mayo Clinic, Rochester, Minnesota, USA; ^12^ Department of Biostatistics, University of Kansas Medical Center, Kansas City, Kansas, USA; ^13^ Department of Histopathology, Addenbrooke's Hospital, Cambridge, UK; ^14^ MRC Clinical Trials Unit, University College London, London, UK; ^15^ Cancer Research UK Clinical Trials Unit, Institute of Cancer Sciences, University of Glasgow, Glasgow, Scotland; ^16^ Dept Gynaecol Oncology, Glasgow Royal Infirmary, Glasgow, Scotland; ^17^ Cambridge University Hospitals NHS Foundation Trust, Cambridge, UK; ^18^ Department of Gynecological Oncology, Roswell Park Cancer Institute, Buffalo, New York, USA; ^19^ Department of Cancer Prevention and Control, Roswell Park Cancer Institute, Buffalo, New York, USA; ^20^ Department of Health Research and Policy - Epidemiology, Stanford University School of Medicine, Stanford, California, USA; ^21^ Women's Cancer Program at the Samuel Oschin Comprehensive Cancer Institute, Cedars-Sinai Medical Center, Los Angeles, California, USA; ^22^ Gynaecology Research Unit, Hannover Medical School, Hannover, Germany; ^23^ Radiation Oncology Research Unit, Hannover Medical School, Hannover, Germany; ^24^ Mother and Child Hospital, Minsk, Belarus; ^25^ Department of Obstetrics and Gynaecology, Friedrich-Schiller University, Jena, Germany; ^26^ Clinics of Obstetrics and Gynaecology, Hannover Medical School, Hannover, Germany; ^27^ Department of Women's Cancer, UCL EGA Institute for Women's Health, University College London, London, UK; ^28^ Department of Medical Genetic, University of Cambridge, Cambridge, UK; ^29^ Memorial Sloan-Kettering Cancer Center, New York, New York, USA; ^30^ Center for Bioinformatics and Functional Genomics, Department Biomedical Sciences, Cedars-Sinai Medical Center, Los Angeles, California, USA; ^31^ Centre for Cancer Genetic Epidemiology, Department of Public Health and Primary Care, University of Cambridge, Cambridge, UK

**Keywords:** ovarian cancer, susceptibility genes, DNA repair, next generation sequencing

## Abstract

We analyzed whole exome sequencing data in germline DNA from 412 high grade serous ovarian cancer (HGSOC) cases from The Cancer Genome Atlas Project and identified 5,517 genes harboring a predicted deleterious germline coding mutation in at least one HGSOC case. Gene-set enrichment analysis showed enrichment for genes involved in DNA repair (p = 1.8×10^-3^). Twelve DNA repair genes - *APEX1, APLF, ATX, EME1, FANCL, FANCM, MAD2L2, PARP2, PARP3, POLN, RAD54L* and *SMUG1* – were prioritized for targeted sequencing in up to 3,107 HGSOC cases, 1,491 cases of other epithelial ovarian cancer (EOC) subtypes and 3,368 unaffected controls of European origin. We estimated mutation prevalence for each gene and tested for associations with disease risk. Mutations were identified in both cases and controls in all genes except *MAD2L2*, where we found no evidence of mutations in controls. In *FANCM* we observed a higher mutation frequency in HGSOC cases compared to controls (29/3,107 cases, 0.96 percent; 13/3,368 controls, 0.38 percent; P=0.008) with little evidence for association with other subtypes (6/1,491, 0.40 percent; P=0.82). The relative risk of HGSOC associated with deleterious *FANCM* mutations was estimated to be 2.5 (95% CI 1.3 – 5.0; P=0.006). In summary, whole exome sequencing of EOC cases with large-scale replication in case-control studies has identified *FANCM* as a likely novel susceptibility gene for HGSOC, with mutations associated with a moderate increase in risk. These data may have clinical implications for risk prediction and prevention approaches for high-grade serous ovarian cancer in the future and a significant impact on reducing disease mortality.

## INTRODUCTION

The genetic architecture of inherited susceptibility to epithelial ovarian cancer (EOC) is complex and pathogenic mutations in multiple DNA repair genes have now been shown to be associated with risk. These genes include *BRCA1* and *BRCA2* [1, 2], the mismatch repair genes [3, 4], *RAD51C* [5, 6], *RAD51D* [7] and *BRIP1* [8]. The risk alleles of these genes are rare in the population and confer ovarian cancer risks ranging from moderate (average risk by age 80 of 5 percent) to high (average risk by age 80 of 50 percent). In addition to rare susceptibility alleles, multiple, common, susceptibility alleles with weak effects have also been identified [9–18]. The known risk alleles explain less than 50 percent of the inherited genetic component of ovarian cancer risk [19] suggesting that other susceptibility alleles exist but are yet to be identified. It is likely that the genetic architecture of this so-called missing heritability is made up of a combination of common, uncommon and rare alleles with weak or moderate effects.

Ovarian cancer is a heterogeneous disease with five main sub-types – high-grade serous, low-grade serous, endometrioid, clear cell and mucinous - and the genetic and clinical characteristics of the different subtypes are distinct. There are also differences in the basis of germline genetic predisposition for the different ovarian cancer subtypes, which reflects in their underlying biology. For example, susceptibility to high-grade serous ovarian cancer (HGSOC) is driven by mutations in genes that are involved in DNA double strand break repair (*BRCA1*, *BRCA2*, *BRIP1*, *RAD51D* and *RAD51C*), and as a consequence these tumors are highly genomically unstable. Ovarian cancers that are associated with Lynch syndrome, which are caused by mutations in the mismatch repair genes, are more likely to be endometrioid and clear cell subtypes and are karyotypically less complex than HGSOCs.

One of the few successful advances in reducing mortality from EOC over the last 20 years has occurred as a result of identifying genes that increase disease risk. The clinical utility of testing for *BRCA1/BRCA2* mutations is well-established; prophylactic bilateral salpingo-oophorectomy plus/minus hysterectomy in carriers is now commonly used to reduce the risk of EOC and is offered to mutation carriers who have completed their families. The recent identification of *RAD51C*, *RAD51D* and *BRIP1* as susceptibility genes for HGSOC raises the possibility that these genes too may have utility for risk prediction and clinical intervention.

The advent of next generation sequencing technologies provides opportunities for both the discovery of novel candidate susceptibility genes and their rapid replication in large sample sizes to confirm their role in disease predisposition. The prevalence of commercially available gene testing panels is also enabling rapid clinical translation of new genes that are identified from these research studies. The aim of this study was to identify genes with uncommon or rare deleterious coding mutations associated with HGSOC using a combination of germline, whole exome sequencing data from HGSOC cases analyzed by TCGA for discovery, followed by candidate-gene, targeted sequencing in a multi-center EOC case-control study for replication.

## RESULTS

### TCGA exome sequencing data and selection of candidate genes

We identified 5,517 genes that harbored predicted deleterious germline coding sequence mutations in at least one of 412 HGSOC cases (≥0.24 percent) (Supplementary Table 2a and 2b). Based on gene-set enrichment analysis, genes in the DNA repair pathway were the most significantly enriched for protein truncating mutations (normalized enrichment = 1.86 fold, p = 1.8×10^-3^, FDR = 0.26, see Supplementary Table 3). This pathway includes several confirmed ovarian cancer susceptibility genes and we identified deleterious mutations in all 5 homologous recombination DNA repair genes known to be associated with predisposition to high-grade serous ovarian cancer: 36 in *BRCA1* (8.7 percent), 26 in *BRCA2* (6.3 percent), 2 in *RAD51C* (0.49 percent), 2 in *RAD51D* (0.49 percent) and 3 in *BRIP1* (0.73 percent) (Supplementary Table 4). Other significantly enriched pathways include ‘Response To Endogenous Stimulus’ (1.75 fold, p = 2.7×10^-3^, FDR = 0.23), ‘Cellular Lipid Metabolism’ (1.72 fold, p =3.5×10^-3^, FDR = 0.21), ‘Lipid Biosynthesis’ (1.85 fold, p = 4.2 x10^-3^, FDR = 0.16), ‘Response to DNA Damage Stimulus’ (1.73 fold, p = 4.8×10^-3^, FDR = 0.22) and ‘Fatty Acid Metabolism’ (1.78 fold, p = 8.3×10^-3^, FDR = 0.26) (Supplementary Table 4).

Genes were prioritized for replication analysis in HGSOC cases and controls based on the following criteria: (1) The presence a deleterious germline mutation in at least one HGSOC case in exome sequencing data from TCGA analysis: (2) Their known or predicted role in DNA repair pathways shown to be involved in the development of high grade serous ovarian cancers, specifically base excision repair (BER), homologous recombination (HR), Fanconi Anemia (FANC); DNA polymerase beta (DNAP) and Poly (ADP-ribose) polymerase (PARP) pathways; (3) The frequency of somatic alterations in primary ovarian tumors identified in candidate genes from the TCGA and Cosmic databases, including the frequencies of somatic coding mutations, homozygous deletions involving candidate genes and loss of heterozygosity (LOH) at the gene locus. Based on these analyses we selected *APEX1, APLF, APTX, EME1, FANCL, FANCM, MAD2L2, PARP2, PARP3, POLN, RAD54L* and *SMUG1* for targeted sequencing (Table 2).

**Table 1:** Characteristics of ovarian cancer case-control populations analyzed in this study

Study	Country	Controls		Cases					
		Number	Mean age(range)	Number	Mean age(range)	High-grade serous^d^	(%)	Stage3/4	(%)^d^
AOC	Australia	629	57 (20-80)	589	61 (23-80)	516	(88)	515	(88)
CAM^c^	UK	0	NA	325	64 (19-90)	196	(60)	237	(79)
GRR^a^	USA	0	NA	124	49 (21-83)	50	(40)	NA	
HJO/HMO	Germany/Belarus	519	36 (18-68)	341	58 (18-88)	107	(32)	153	(45)
ICN^c^	UK	0	NA	422	57 (24-79)	293	(69)	31	(86)
LAX	USA	209	62 (34-90)	175	62 (32-88)	175	(100)	159	(92)
MAY	USA	660	63 (26-93)	650	64 (23-91)	630	(97)	581	(89)
RMH^b^	UK	0	NA	61	53 (27-73)	61	(100)	NA	
SEA	UK	835	53 (29-66)	700	57 (24-74)	349	(50)	388	(70)
SRO^c^	UK	0	NA	627	57 (18-84)	318	(51)	507	(81)
STA	USA	147	48 (20-64)	151	53 (23-64)	116	(77)	111	(74)
UKO	UK	369	65 (52-78)	353	61 (25-90)	267	(75)	242	(72)
**Total**		**3,368**	**55 (18-93)**	**4,508**	**59 (18-91)**	**3,017**	**(67)**	**2,924**	**(81)**

All studies are case-control studies except for: ^a^ familial ovarian cancer registry study, ^b^ case only hospital study and ^c^ case only clinical trial; ^d^ % of those with known stage

**Table 2:** Frequency of somatic and germline mutations in 12 candidate susceptibility genes in 412 high-grade serous ovarian cancer cases from TCGA and germline mutations in up to 4,508 ovarian cancer cases and 3,368 controls

Gene	Somatic variants(412 TCGA cases)		Germline mutations				
	Mutation^a^ N (%)	LOH^b^ (%)	TCGA cases N (%)	HG serouscases (%)	Other cases N (%)	Controls N (%)	P-value^c^
				**N=2,210**	**N=924**	**N=3,368**	
*APEX1*	2 (0.49)	29	1 (0.24)	1 (0.03)	2 (0.13)	3 (0.09)	0.52
*EME1*	0	52	1 (0.24)	3 (0.10)	1 (0.07)	3 (0.09)	0.91
*FANCL*	2 (0.49)	10	1 (0.24)	18 (0.60)	8 (0.54)	28 (0.83)	0.34
*MAD2L2*	1 (0.24)	34	2 (0.49)	1 (0.03)	0	0	-
*PARP3*	1 (0.24)	31	1 (0.24)	6 (0.20)	1 (0.07)	13 (0.39)	0.22
*POLN*	1 (0.24)	42	3 (0.73)	15 (0.50)	10 (0.67)	26 (0.77)	0.09
*RAD54L*	2 (0.49)	12	1 (0.24)	4 (0.13)	1 (0.20)	6 (0.18)	0.80
*SMUG1*	0	18	1 (0.24)	2 (0.07)	1 (0.07)	2 (0.06)	0.71
				**N=3,107**	**N=1,491**	**N=3,368**	
*APLF*	2 (0.49)	10	5 (1.21)	92 (3.1)	40 (2.7)	121 (3.6)	0.16
*APTX*	2 (0.49)	40	1 (0.24)	4 (0.13)	4 (0.27)	4 (0.12)	0.97
*FANCM*	2 (0.49)	41	1 (0.24)	29 (0.96)	6 (0.40)	13 (0.39)	0.008
*PARP2*	1 (0.24)	30	1 (0.24)	4 (0.13)	3 (0.20)	5 (0.15)	0.65

a germline mutation

b Loss of heterozygosity

c Comparison of high-grade serous cases with controls

### Association of truncating mutations in candidate genes and high-grade serous ovarian cancer

After quality control analysis and exclusion of samples from participants of non-European origin, sequencing information for the coding region and splice site boundaries of the 12 candidate genes was available for 2,210 high-grade serous cases, 924 other cases and 3,368 controls. Sequencing information was also available for four of the genes - *APLF*, *APTX*, *FANCM* and *PARP2 -* in an additional 807 high-grade serous cases and 567 cases of other subtypes. The characteristics of these individuals by study are summarized in Table 1. We identified predicted-truncating, germline mutations (nonsense, frameshift indels or splice site alterations) in both cases and controls in 11 genes; only in MAD2L2 did we find no evidence of truncating mutations in controls. The frequency of mutations varied between genes. For example, in *APLF* we identified mutations in 2.9 percent of cases and 3.7 percent of controls, while in 5 genes - *APEX1*, *APTX*, *EME1*, *MAD2L2* and SMUG1 - we identified mutations in less than 0.2 percent in both cases and controls (Table 2 & Supplementary Table 5).

We observed a higher frequency of mutations in *FANCM* in high-grade serous cases compared to controls (29/3,107 cases, 0.96 percent; 13/3,368 controls, 0.38 percent; P=0.008). The frequency of *FANCM* mutations in cases of the other sub-types was similar to that of controls (6/1,491, 0.40 percent; p=0.82), although the difference in frequency between high-grade serous and other cases was not statistically significant (P=0.14). The mean age of diagnosis in *FANCM* mutation carriers with HGSOC was 61.4 years compared to 60.3 years in non-carriers. We estimated the relative risk of ovarian cancer associated with deleterious mutations in the *FANCM* gene as the odds ratio (OR), using data from the case-control studies that were not family based. The relative risk of HGSOC was 2.5 (95% confidence interval (CI) 1.3 – 5.0; P=0.006) with no increase in risk for other subtypes (OR = 0.89, 95% CI 0.32 – 2.5; P=0.82). The relative risk for all histologic subtypes of invasive epithelial ovarian cancer was 2.1 (95% 1.1 – 3.9; P=0.029).

**Figure 1:**
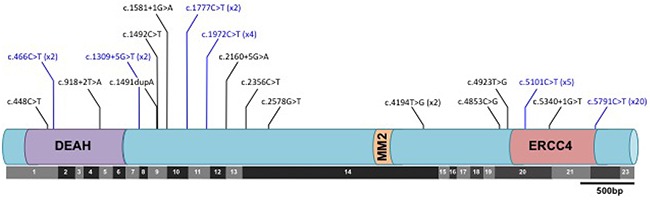
Distribution of predicted deleterious mutations in the *FANCM* gene in ovarian cancer cases and controls with respect to both the translated protein and the exonic architecture of the coding sequence Black lines indicate mutations identified in cases only; blue lines indicate mutations detected both cases and controls. No mutations were identified in controls only. Where a mutation was identified in more than one subject, the number of times the mutation was detected is given in brackets.

*FANCM* mutations were broadly clustered into two regions. The first was an approximately 2kb region (nucleotides 446-2578) in and around a DEAH helicase domain at the N-terminus of the gene, which harbors 12/19 of the different mutations we identified. The remaining seven different mutations were located in a region spanning almost 1kb between nucleotides 4853 and 5791 and a C-terminal degenerate ERCC4-like domain (Figure 1, Table 2 & Supplementary Table 6). However, there was little evidence that the distribution of *FANCM* mutations in cases along the gene differed from that in controls (P=0.60). The C-terminus nonsense variant c.C5791T was recurrent, occurring in 13 cases (0.29 percent) and 7 controls (0.21 percent). N-terminal and C-terminal truncating variants in susceptibility genes (e.g. K3326* variant in *BRCA2*; 6218delC variant in *APC*) have been shown to be associated with lower disease penetrance compared to truncating mutations elsewhere in the same gene [28, 29]. We therefore evaluated the association of *FANCM* mutations with HGSOC after re-classifying carriers of c.C5791T as non-deleterious. The relative risk for more proximal mutations was 3.6 (95% CI 1.4 – 9.5; P=0.010), suggesting that c.C5791T may also be associated with a smaller risk.

In addition to truncating deleterious mutations, we also identified 627 different rare (less than 1 percent frequency) non-synonymous coding variants in these genes (Supplementary Table 7). Of these 243 are predicted to be damaging by at least two of the three function prediction programs Polyphen-2, Provean and SIFT; there was no difference in the frequency of uncommon variants in cases compared to controls for any of the genes evaluated based on the rare admixture maximum likelihood test (Supplementary Table 8).

## DISCUSSION

We have carried out a comprehensive evaluation of germline coding sequence variation throughout the coding transcriptome in 412 HGSOC cases to identify candidate susceptibility genes associated with this subtype of disease. We used the targeted sequencing analysis of ovarian cancer cases and unaffected controls of European ancestry to establish the prevalence of predicted deleterious mutations in 12 genes involved in DNA repair. We found evidence that protein truncating mutations in *FANCM* are associated with an increased risk of HGSOC (P=0.008) with little evidence for an association for other subtypes (P=0.82). The increased risk for HGSOC is consistent with its functional interaction with *BRCA1/BRCA2* in homologous recombination and double strand DNA repair [4, 30]. It has recently been suggested that P<10^-4^ is a reasonable threshold for evaluating the association of rare, protein-truncating variants in candidate genes [31]. Assuming a population prevalence of 0.38%, as found in our controls, 6,100 cases and 6,100 controls would be required to detect an OR of 3 at this type 1 error rate with 80 percent power. Although such samples sizes are now feasible, more modest effects would need much larger samples sizes; for example 18,100 cases and 18,100 controls to detect an OR of 2. The NHLBI GO Exome Sequencing Project has generated exome sequencing data on over 6,000 individuals from multiple studies carried out in the USA (http://evs.gs.washington.edu/EVS/). Of these 4,300 individuals are of European ancestry, of whom 16 carry a protein truncating variant in *FANCM* which is a similar frequency to that in our controls (frequency 0.37 percent). If these samples are used as additional controls for the US samples the association with high-grade serous ovarian cancer becomes more significant (P=0.001) yet they do not reach the nominal threshold for significance of P<10^-4^. Further support for our findings comes from a Finnish study that reported a significant association for *FANCM* Q1701X with triple negative breast cancer [32]. While a significant association with ovarian cancer was not reported, the frequency of this nonsense variant was higher in ovarian cancer cases than controls (OR = 1.6, 95% CI 0.75 – 3.2, P = 0.23). However, this study had limited power to detect an association because of the modest sample size (number of cases = 548) with power further reduced by including all subtypes of ovarian cancer.

The prevalence of truncating mutations in *APEX1, APLF, ATX, EME1, FANCL, MAD2L2, PARP2, PARP3, POLN, RAD54L* and *SMUG1* in controls was very low with little evidence for an increased frequency in cases. It is therefore unlikely that these genes contribute substantially to ovarian cancer risk, but we cannot rule out that deleterious mutations in any one of these genes are associated with modest risks. The fact that truncating mutations in these genes were identified in controls highlights the need for caution in interpreting the findings of sequencing studies carried out in case series without appropriate controls. This is particularly important given the common practice of including such genes on gene sequencing panels that are used in clinical practice [31].

It is likely that we have underestimated the true prevalence of deleterious variants in these genes in cases and controls. Our sequencing method did not provide complete sequence coverage of each gene in all samples (mean coverage 90 to 97 percent in cases and controls) and so some mutations may have been missed. Furthermore, the PCR enrichment used for sequencing library preparation does not enable the detection of large genomic deletions and rearrangement mutations. Finally, we did not include missense variants in our prevalence or risk estimates because we cannot be certain of their pathogenicity in the absence of definitive functional assays.

**Figure 2:**
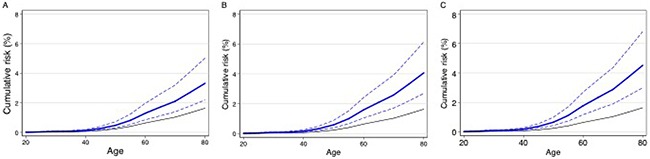
Estimated cumulative risk of epithelial ovarian cancer in women with a germline truncating mutation in *FANCM*: Black line – population risk based on UK incidence data for 2009 Blue line – estimated risk to FANCM carriers (dashed lines 80% confidence limits): **A**. average risks; **B**. risks to FANCM carriers at the 80^th^ centile of a polygenic risk distribution based on 18 known common risk alleles; **C**. risks to FANCM carriers at the 80^th^ centile of a risk distribution based on 18 known common risk alleles together with known lifestyle risk factors.

If the association of *FANCM* protein truncating mutations is confirmed, *FANCM* mutation analysis could be rapidly implemented as part of a program of clinical genetic testing followed by prophylactic surgery (salpingo-oophorectomy). However, the clinical utility in testing unaffected women for *FANCM* mutations is unclear from the risk estimates. The “best” estimate (the point estimate) of the relative risk is equivalent to a lifetime risk of invasive epithelial ovarian cancer of 3.8 percent, although the true risk may be lower than this given the uncertainty of the risk estimate. It has been suggested that 80 percent confidence limits on cumulative risk estimates are more appropriate for clinical decision-making. These are shown in Figure 2A. The cumulative risk is also likely to be modified by the presence of other lifestyle and genetic risk factors. The log-additive model on a relative risk scale for interaction between risk factors has been shown to fit well for interactions between risk alleles and lifestyle risk factors. Eighteen common risk alleles for ovarian cancer have now been identified [9–11, 14–16, 18]. Women with *FANCM* mutations at the 80^th^ centile of the polygenic risk distribution based on the 18 known common risk alleles [9–18] would have an expected lifetime risk of 4.6 percent (80% CI 3.1 – 7.0, Figure 2B), assuming that this log-additive model also applies to *FANCM* carriers. Incorporating other EOC risk factors, specifically oral contraceptive pill use, tubal ligation, parity, a history of endometriosis and family history increases the lifetime risk at the 80^th^ centile of the risk distribution to 5.2 percent (80% CI 3.4 – 7.8, Figure 2C). *FANCM* mutation testing in women with HGSOC might also have clinical utility through targeted treatment with Poly (ADP-ribose) polymerase (PARP) inhibitors, which are currently being evaluated in women with *BRCA1* and *BRCA2* associated ovarian cancer.

Gene-set enrichment analysis of genes with at least one protein truncating mutation in the TCGA HGSOC cases showed a statistically significant enrichment for genes involved in ‘DNA Repair’ processes. This is perhaps not surprising given the critical role of double strand DNA repair in the initiation and development of HGSOCs, and its contribution to the chromosomal instability of this tumor subtype. *FANCM* adds to a growing list of DNA repair associated genes (*BRCA1*, *BRCA2*, *RAD51C*, *RAD51D* and *BRIP1*) that confer susceptibility to HGSOC. We also observed significant enrichment for genes in biosynthesis and metabolic pathways including cellular lipid metabolism, lipid biosynthesis and fatty acid metabolism. It is known that ovarian cancer cells migrate to the adipocyte-rich omentum and recent reports show that lipids are transferred from omental adipocytes to ovarian cancer cells, where they are metabolised and accelerate tumorigenesis [33]. These findings are consistent with recent data suggesting that ovarian cancer cells undergo a metabolic shift during tumorigenesis to become more dependent on lipid metabolism as an energy source [34]. Genes in these pathways may represent a new class of ovarian cancer susceptibility genes, but is will require large case-control targeted sequencing analyses similar to those described in the current study to confirm this.

In summary, we have found reasonable evidence that deleterious germline mutations in *FANCM* are associated with a moderate increase in the risk of high-grade serous epithelial ovarian cancer but are not associated with non-serous disease subtypes. Predicted deleterious mutations in the other 11 candidate genes we identified from TCGA whole exome analysis do not appear to predispose to ovarian cancer. This study highlights the critical need for accurate risk estimation of candidate susceptibility genes based on very large sample sizes before genes of moderate penetrance have clinical utility in cancer prevention.

## MATERIALS AND METHODS

### TCGA germline exome sequencing data analysis

Data were collected by the TCGA project as described elsewhere (http://cancergenome.nih.gov/). We received approval from the TCGA data access committee to access and analyze the germline exome sequencing data from HGSOC cases. Sequence data for 412 cases were downloaded from the online NIH facility, dbGaP, as BAM files. We used the Samtools sequence analysis software [20] for the manipulation and analysis of the BAM files. The Samtools *pileup* function was used to assemble and quantify sequence variation in each sample. Then ANNOVAR was used to annotate the sequence variation detected. We classified all variants predicted to result in truncation of the protein (frameshift indels, nonsense substitutions and consensus splice site variants) as potentially deleterious variants. We only carried forward variants having a depth of at least 40 and an alternative allele ratio of at least 30 percent. Common variants with a minor allele frequency of >5 percent were excluded from the analysis. A finalised set of uncommon deleterious variants identified was tabulated for every RefSeq gene with a mature mRNA structure, i.e. having an NM type accession identifier. We identified 8,275 unique, uncommon, deleterious variants in 5,517 genes occurring a total of 16,969 times in 412 cases (see Supplementary file DeleteriousVariants.xls for details).

Lymphocyte DNA for 341 of the TCGA samples were also genotyped using the Illumina Omni1M genotyping array. We used these data to estimate the accuracy of the calling of substitutions by comparing the called genotypes based on the NGS data with the genotype calls from the TCGA SNP genotyping. Concordance for 1.96M genotypes was 99.0 percent. No equivalent data are available to evaluate the quality of indel calling.

### Study subjects

We carried out targeted sequencing of 12 candidate genes selected using the results from the TCGA data analysis (see results) in 2,385 high-grade serous cases and 3,569 controls. These were from 8 ovarian cancer case-control studies (AOC, HJO, HMO, LAX, MAY, SEA, STA, UKO), 1 familial ovarian cancer registry from the USA (GRR) and 1 case series (RMH). These studies have been described previously (e.g [15]). We also selected 988 cases with other tumour histologies (Table 1) as some common alleles that predispose to HGSOC are also associated with an increased risk of other subtypes. All studies had ethics committee approval, and all participants provided written, informed consent.

In addition we sequenced four of the genes of interest (*APLF*, *APTX*, *FANCM* and *PARP2*) in another 859 high grade serous cases and 609 cases of other sub-types from the SCOTROC (SRO), ICON7 (ICN) and OV04 (CAM) ovarian cancer clinical trials.

### Targeted gene sequencing

Target sequence enrichment was performed using 48.48 Fluidigm access arrays and 4-primer chemistry for addition of barcode and adapter sequences during the PCR amplification. Target sequence amplicons were 200bp or smaller for complete sequencing using 100 bp pair-end sequencing on the Illumina HiSeq2000 according to the manufacturer's protocol (Illumina Inc, San Diego, CA). A total of 314 amplicons were designed and the average fragment size was 187 base pairs (range 150-200) (Supplementary Table 1).

Sequenced reads were de-multiplexed using standard Illumina software. We used the Burrows-Wheeler Aligner (BWA) [21] for sequencing read alignment against the human genome reference sequence (hg19). The Genome Analysis Toolkit (GATK) [22] was used for base quality-score recalibration, local insertion/deletion (indel) realignment, and substitution/indel discovery. Variants were only considered for further analysis if they satisfied the set of recommended GATK filters as applicable to our data and as described in the GATK best practices guide. ANNOVAR [23] was used to annotate the sequence variation detected. The transcript identifiers used for mutation annotation of the 12 genes are detailed in Supplementary Table 1.

Variant alternate allele frequency was defined as the fraction of alternate allele bases compared to the total number of bases at the variant locus. We applied the following thresholds for variants calling: the minimum coverage is 15, a variant will be called if (1) coverage ≥500 and alternate allele frequency ≥10 percent, or (2) 250 ≤coverage <500 and alternate allele frequency ≥15 percent, or (3) 30 ≤coverage <250 and alternate allele frequency ≥20 percent, or (4) 15 ≤coverage <30 and alternate allele frequency ≥30 percent. The thresholds for coverage and alternate allele frequency used in variant calling were defined previously based on results from sequencing of positive controls with known variants.

We excluded 202 cases and 56 controls from further analyses because <80 percent of the target bases from these samples had read depth ≥15. The average coverage of coding region and splice sites screened at 15X depth for the remaining samples (up to 8,070) were: 95.9 percent for *APEX1*; 95.0 percent for *APLF*; 97.0 percent for *APTX*; 97.1 percent for *EME1*; 97.9 percent for *FANCL*; 95.8 percent for *FANCM*; 95.7 percent for *MAD2L2*; 93.9 percent for *PARP2*; 94.9 percent for *PARP3*; 94.7 percent for *POLN*; 97.4 percent for *RAD54L*; and 98.4 percent for *SMUG1*.

### Deleterious mutation identification and validation

Deleterious variants were defined as those predicted to result in protein truncation (frameshift, splice site and nonsense mutations). We used the programme MaxEntScan to identify splice site variants most likely to affect gene splicing [24]. Splice site variants with a MaxEntScan score that differed from the score for the consensus sequence by more than 40 percent were assumed to affect splicing. Sequencing alignments were visually inspected using the Integrative Genomic Viewer (IGV) [25] to confirm presence of deleterious variants. We performed Sanger sequencing using standard methods for validation in independent PCR products of all potentially deleterious truncating variants and all potentially deleterious non-synonymous variants.

### Statistical methods

We carried out a pathway analysis to identify sets of genes most likely to harbour truncating mutations in the 412 HGSOC cases in TCGA. We excluded mutations that occurred in more than 8 out of 412 cases (MAF > 0.5%). We ranked all genes containing frameshift indels and nonsense substitutions (4,640 genes after removing 189 open reading frames) in descending order of the number mutations per gene. To account for the possibility of genes with longer coding length containing a larger number of mutations simply due to their length and to break ties among genes containing the same number of mutations, we regressed the number of mutations on coding length and used the residuals as the actual ranking metric. A total of 249 pathways containing between 15 and 500 genes from the Gene Ontology Biological Processes compendium were downloaded from the Molecular Signatures Database (v5.0) [26]. The ranked list of genes and the pathways were used for gene set enrichment analysis (GSEA; v2-2.2.2.0) run to 10,000 permutations [26].

We tested for association between deleterious mutations and ovarian cancer risk using unconditional logistic regression adjusted for geographical region of origin (Australia, continental Europe, the United Kingdom and the USA). Odds ratios and associated 95 percent confidence intervals (95% CI) were also calculated.

We identified multiple missense variants that have an unknown functional effect on the proteins. We excluded all missense variants with a minor allele frequency of > 1 percent from further analyses as large-scale genome-wide association studies have shown that the relative risks conferred by common susceptibility allele are small (< 1.3) and thus unlikely to be detectable by the sample size of this study. The statistical power to detect single rare alleles by association, even if they confer larger risk (RR > 2) is still modest. We therefore used the rare admixture likelihood (RAML) burden test [27] to test for association on a gene-by-gene basis. The RAML combines the information across multiple rare variants to increase statistical power and allows for alleles associated with either an increased or a decreased risk. We classified variants with frequency ≤ 1 percent by whether or not they are predicted to have a damaging effect on protein function by more than one of the following prediction tools - SIFT (score <0.05), polyphen-2 (classified as probably damaging or damaging) and Provean (score<=-2.5). Only subjects with a call rate greater than 80 percent for missense variants and variants with a call rate greater than 80 percent with genotype frequencies consistent with Hardy-Weinberg equilibrium (P>10^-5^) were included in these analyses.

## SUPPLEMENTARY MATERIALS AND TABLES














