# Supplementary material for: Germline whole exome sequencing and large-scale replication identifies FANCM as a likely high grade serous ovarian cancer susceptibility gene
Source: Oncotarget. 2017 Mar 3;8(31):50930–40. doi: 10.18632/oncotarget.15871 (PMC5584218; doi:10.18632/oncotarget.15871)
Supplement: Supplementary file 1 [file oncotarget-08-50930-s001.pdf]

Germline whole exome sequencing and large-scale replication identifies *FANCM* as a likely high grade serous ovarian cancer susceptibility gene

SUPPLEMENTARY TABLES

Supplementary Table 1: Target regions for sequencing of 12 candidate genes identified from TCGA exome sequence data.

See Supplementary File 1

Supplementary Table 2a: List of genes with at least one protein truncating mutation in 412 HGSOC cases from TCGA.

See Supplementary File 2

Supplementary Table 2b: Details of protein truncating mutations identified in TCGA HGSOC cases.

See Supplementary File 2

Supplementary Table 3: Pathway analysis of genes harbouring protein truncating mutations in 412 HGSOC cases.

See Supplementary File 3

Supplementary Table 4: Germline coding sequence mutations identified in known ovarian cancer susceptibility genes *BRCA1*, *BRCA2*, *RAD51C*, *RAD51D* and *BRIP1*, from germline exome sequencing of 412 HGSOC cases

Gene ID	Pathway	Coding sequencing mutations		Other somatic Alterations	
		Germline N (%)	Somatic N (%)	Δ Hom. N (%)	LOH N (%)
BRCA1	HR	19 (4.61%)	14 (3.5 %)	<1 %	-77%
BRCA2	FANC	18 (4.37%)	13 (3.2 %)	<1	-58%
RAD51D	HR	4 (0.97%)	0	1	-85%
BRIP1	FANC	1 (0.24%)	<1	<1	-45%
RAD51C	FANC	1 (0.24%)	0	0	-48%

Also included are data for somatic variants identified in the same series of tumors including somatic coding sequence mutations, homozygous deletions (D HOM) and the frequency of loss of heterozygous (LOH) at the gene locus

Supplementary Table 5: Predicted deleterious truncating mutations identified in 11 candidate genes.

See Supplementary File 4

Supplementary Table 6: Predicted deleterious truncating mutations identified in *FANCM*.

See Supplementary File 5

Supplementary Table 7: Putative pathogenic missense variants identified in each gene based on *in silico* software programs SIFT, Polyphen-2 and Provean.

See Supplementary File 6

Supplementary Table 8: Number of missense variants detected in cases and controls by gene

Gene	Number variants	Controls		Cases	
		Number	%	Number	%
Predicted not damaging					
APEX1[a]	15	14	0.42	12	0.38
APLF[b]	25	52	1.54	81	1.8
APTX[b]	19	96	2.85	137	3.04
EME1[a]	33	48	1.43	50	1.6
FANCL[a]	20	22	0.65	15	0.48
FANCM[b]	113	151	4.48	223	4.95
PARP2[b]	25	25	0.74	31	0.69
PARP3[a]	43	43	1.28	55	1.75
POLN[a]	60	130	3.86	112	3.57
RAD54L[a]	25	57	1.69	42	1.34
SMUG1[a]	6	5	0.15	4	0.13
Predicted damaging					
APEX1[a]	11	8	0.24	9	0.29
APLF[b]	14	30	0.89	40	0.89
APTX[b]	7	11	0.33	18	0.4
EME1[a]	14	26	0.77	15	0.48
FANCL[a]	15	40	1.19	24	0.77
FANCM[b]	70	63	1.87	85	1.89
PARP2[c]	25	15	0.45	20	0.44
PARP3[a]	19	17	0.5	21	0.67
POLN[a]	24	12	0.36	18	0.57
RAD54L[a]	29	45	1.34	48	1.53
SMUG1[a]	15	14	0.42	28	0.89

a 3,368 controls and 3,134 cases; b 3,368 controls and 4,508 cases
