# Supplementary material for: Germline whole exome sequencing and large-scale replication identifies FANCM as a likely high grade serous ovarian cancer susceptibility gene
Source: Oncotarget. 2017 Mar 3;8(31):50930–40. doi: 10.18632/oncotarget.15871 (PMC5584218; doi:10.18632/oncotarget.15871)
Supplement: Supplementary file 2 [file oncotarget-08-50930-s002.docx]

**Supplementary Table 1:** Target regions for sequencing of 12 candidate genes identified from TCGA exome sequence data.

| **Exon** | **Target region start** | **Target region stop** | **Target size** | **Coverage** | **Number of Amplicons** |
| --- | --- | --- | --- | --- | --- |
| **APEX1 (NM_001244249.1), chromosome 14** | | | | | 11 |
| Exon 1 | 20923797 | 20923862 | 65 | 100% | 1 |
| Exon 2 | 20924072 | 20924260 | 188 | 100% | 2 |
| Exon 3 | 20924826 | 20925019 | 193 | 100% | 2 |
| Exon 4 | 20925149 | 20925668 | 519 | 100% | 6 |
| **APLF (NM_173545.2), chromosome 2** | | | | | 21 |
| Exon 1 | 68694856 | 68694959 | 103 | 100% | 1 |
| Exon 2 | 68717321 | 68717393 | 72 | 100% | 1 |
| Exon 3 | 68729862 | 68730035 | 173 | 89% | 2 |
| Exon 4 | 68740211 | 68740359 | 148 | 100% | 3 |
| Exon 5 | 68740679 | 68740812 | 133 | 100% | 2 |
| Exon 6 | 68753192 | 68753374 | 182 | 100% | 3 |
| Exon 7 | 68765003 | 68765359 | 356 | 100% | 4 |
| exon 8 | 68772318 | 68772444 | 126 | 100% | 2 |
| exon 9 | 68794472 | 68794519 | 47 | 100% | 1 |
| exon 10 | 68804951 | 68805155 | 204 | 100% | 2 |
| **APTX (NM_175073.2), chromosome 9** | | | | | 16 |
| exon 9 | 32973494 | 32973650 | 156 | 100% | 2 |
| exon 8 | 32973680 | 32973686 | 6 | 100% | 1 |
| exon 7 | 32974455 | 32974559 | 104 | 100% | 2 |
| exon 6 | 32984628 | 32984855 | 227 | 100% | 2 |
| exon 5 | 32985968 | 32986028 | 60 | 74% | 2 |
| exon 4 | 32987541 | 32987840 | 299 | 100% | 3 |
| exon 3 | 32988076 | 32988127 | 51 | 100% | 1 |
| exon 2 | 32989756 | 32989890 | 134 | 100% | 2 |
| exon 1 | 33001564 | 33001609 | 45 | 100% | 1 |
| **EME1 (NM_001166131.1), chromosome 17** | | | | | 21 |
| exon 1 | 48452562 | 48453344 | 782 | 100% | 9 |
| exon 2 | 48453426 | 48453554 | 128 | 100% | 2 |
| exon 3 | 48455955 | 48456042 | 87 | 100% | 1 |
| exon 4 | 48456134 | 48456256 | 122 | 100% | 2 |
| exon 5 | 48456428 | 48456585 | 157 | 100% | 2 |
| exon 6 | 48456812 | 48456928 | 116 | 100% | 2 |
| exon 7 | 48457672 | 48457862 | 190 | 100% | 2 |
| exon 8 | 48458123 | 48458301 | 178 | 100% | 2 |
| **FANCL (NM_001114636.1), chromosome 2** | | | | | 22 |
| exon 14 | 58386898 | 58386935 | 37 | 100% | 1 |
| exon 13 | 58387242 | 58387314 | 72 | 100% | 1 |
| exon 12 | 58388656 | 58388773 | 117 | 100% | 2 |
| exon 11 | 58390000 | 58390082 | 82 | 100% | 1 |
| exon 10 | 58390163 | 58390209 | 46 | 100% | 2 |
| exon 9 | 58390568 | 58390652 | 84 | 100% | 3 |
| exon 8 | 58392858 | 58393009 | 151 | 100% | 2 |
| exon 7 | 58425713 | 58425797 | 84 | 100% | 1 |
| exon 6 | 58431264 | 58431361 | 97 | 100% | 2 |
| exon 5 | 58449076 | 58449177 | 101 | 100% | 2 |
| exon 4 | 58453862 | 58453919 | 57 | 100% | 1 |
| exon 3 | 58456948 | 58457009 | 61 | 100% | 2 |
| exon 2 | 58459188 | 58459247 | 59 | 100% | 1 |
| exon 1 | 58468352 | 58468455 | 103 | 100% | 1 |
| **FANCM (NM_020937.2), chromosome 14** | | | | | 85 |
| exon 1 | 45605227 | 45605742 | 515 | 100% | 6 |
| exon 2 | 45606271 | 45606444 | 173 | 100% | 2 |
| exon 3 | 45609834 | 45609912 | 78 | 97% | 2 |
| exon 4 | 45618039 | 45618198 | 159 | 88% | 2 |
| exon 5 | 45620599 | 45620731 | 132 | 100% | 2 |
| exon 6 | 45623122 | 45623255 | 133 | 100% | 2 |
| exon 7 | 45623899 | 45624025 | 126 | 100% | 2 |
| exon 8 | 45624575 | 45624662 | 87 | 77% | 2 |
| exon 9 | 45628298 | 45628483 | 185 | 100% | 3 |
| exon 10 | 45633561 | 45633768 | 207 | 100% | 2 |
| exon 11 | 45636152 | 45636366 | 214 | 100% | 3 |
| exon 12 | 45639791 | 45639949 | 158 | 100% | 3 |
| exon 13 | 45642257 | 45642413 | 156 | 100% | 2 |
| exon 14 | 45644273 | 45646179 | 1906 | 100% | 22 |
| exon 15 | 45650632 | 45650727 | 95 | 100% | 2 |
| exon 16 | 45650839 | 45650908 | 69 | 100% | 2 |
| exon 17 | 45652976 | 45653105 | 129 | 95% | 2 |
| exon 18 | 45654419 | 45654576 | 157 | 100% | 3 |
| exon 19 | 45656983 | 45657090 | 107 | 92% | 2 |
| exon 20 | 45658004 | 45658565 | 561 | 100% | 6 |
| exon 21 | 45665374 | 45665750 | 376 | 100% | 6 |
| exon 22 | 45667846 | 45668138 | 292 | 100% | 5 |
| exon 23 | 45669072 | 45669212 | 140 | 100% | 2 |
| **MAD2L2 (NM_006341.3), chromosome 1** | | | | | 12 |
| exon 8 | 11734830 | 11734873 | 43 | 100% | 1 |
| exon 7 | 11735138 | 11735231 | 93 | 100% | 2 |
| exon 6 | 11735714 | 11735788 | 74 | 100% | 1 |
| exon 5 | 11736102 | 11736197 | 95 | 100% | 1 |
| exon 4 | 11736904 | 11737005 | 101 | 100% | 3 |
| exon 3 | 11737599 | 11737671 | 72 | 100% | 1 |
| exon 2 | 11740409 | 11740528 | 119 | 100% | 2 |
| exon 1 | 11740618 | 11740665 | 47 | 100% | 1 |
| **PARP2 (NM_005484.3), chromosome 14** | | | | | 26 |
| exon 1 | 20811793 | 20811846 | 53 | 100% | 1 |
| exon 2 | 20813090 | 20813285 | 195 | 100% | 2 |
| exon 3 | 20813552 | 20813623 | 71 | 100% | 2 |
| exon 4 | 20815021 | 20815072 | 51 | 100% | 1 |
| exon 5 | 20818684 | 20818781 | 97 | 100% | 2 |
| exon 6 | 20819205 | 20819281 | 76 | 100% | 1 |
| exon 7 | 20820403 | 20820506 | 103 | 100% | 2 |
| exon 8 | 20822243 | 20822406 | 163 | 100% | 2 |
| exon 9 | 20822967 | 20823106 | 139 | 100% | 1 |
| exon 10 | 20823910 | 20823974 | 64 | 100% | 1 |
| exon 11 | 20824055 | 20824190 | 135 | 100% | 2 |
| exon 12 | 20824465 | 20824593 | 128 | 100% | 2 |
| exon 13 | 20824748 | 20824848 | 100 | 100% | 2 |
| exon 14 | 20825209 | 20825308 | 99 | 100% | 1 |
| exon 15 | 20825554 | 20825679 | 125 | 100% | 2 |
| exon 16 | 20825796 | 20825957 | 161 | 100% | 2 |
| **PARP3 (NM_001003931.2), chromosome 3** | | | | | 21 |
| exon 1 | 51977363 | 51977554 | 191 | 100% | 2 |
| exon 2 | 51978104 | 51978233 | 129 | 100% | 2 |
| exon 3 | 51978405 | 51978594 | 189 | 100% | 2 |
| exon 4 | 51978793 | 51978926 | 133 | 100% | 2 |
| exon 5 | 51979013 | 51979240 | 227 | 100% | 3 |
| exon 6 | 51979510 | 51979660 | 150 | 100% | 2 |
| exon 7 | 51979868 | 51979955 | 87 | 100% | 2 |
| exon 8 | 51980181 | 51980359 | 178 | 100% | 2 |
| exon 9 | 51981755 | 51981911 | 156 | 100% | 2 |
| exon 10 | 51982326 | 51982497 | 171 | 100% | 2 |
| **POLN (NM_181808.2), chromosome 4** | | | | | 38 |
| exon 24 | 2073839 | 2074026 | 187 | 100% | 2 |
| exon 23 | 2074694 | 2074756 | 62 | 100% | 1 |
| exon 22 | 2077178 | 2077246 | 68 | 100% | 1 |
| exon 21 | 2082684 | 2082763 | 79 | 100% | 1 |
| exon 20 | 2083359 | 2083470 | 111 | 100% | 1 |
| exon 19 | 2087339 | 2087471 | 132 | 100% | 2 |
| exon 18 | 2097577 | 2097660 | 83 | 100% | 1 |
| exon 17 | 2129839 | 2129954 | 115 | 100% | 2 |
| exon 16 | 2130905 | 2130983 | 78 | 100% | 1 |
| exon 15 | 2132959 | 2133017 | 58 | 100% | 1 |
| exon 14 | 2158514 | 2158580 | 66 | 100% | 1 |
| exon 13 | 2159584 | 2159638 | 54 | 100% | 1 |
| exon 12 | 2160881 | 2160938 | 57 | 100% | 1 |
| exon 11 | 2172405 | 2172501 | 96 | 100% | 1 |
| exon 10 | 2172824 | 2172908 | 84 | 100% | 2 |
| exon 9 | 2175681 | 2175746 | 65 | 100% | 1 |
| exon 8 | 2176417 | 2176478 | 61 | 100% | 1 |
| exon 7 | 2177992 | 2178061 | 69 | 100% | 2 |
| exon 6 | 2181034 | 2181192 | 158 | 100% | 2 |
| exon 5 | 2194930 | 2195043 | 113 | 41% | 2 |
| exon 4 | 2200250 | 2200444 | 194 | 100% | 2 |
| exon 3 | 2209713 | 2210214 | 501 | 100% | 6 |
| exon 2 | 2214773 | 2214853 | 80 | 100% | 1 |
| exon 1 | 2230825 | 2230964 | 139 | 100% | 2 |
| **RAD54L (NM_003579.3), chromosome 1** | | | | | 31 |
| exon 1 | 46714070 | 46714080 | 10 | 100% | 1 |
| exon 2 | 46714183 | 46714270 | 87 | 100% | 1 |
| exon 3 | 46715671 | 46715791 | 120 | 100% | 2 |
| exon 4 | 46724357 | 46724418 | 61 | 100% | 1 |
| exon 5 | 46725635 | 46725771 | 136 | 100% | 2 |
| exon 6 | 46726213 | 46726283 | 70 | 100% | 1 |
| exon 7 | 46726398 | 46726687 | 289 | 100% | 3 |
| exon 8 | 46726932 | 46727057 | 125 | 100% | 2 |
| exon 9 | 46733130 | 46733281 | 151 | 100% | 2 |
| exon 10 | 46736330 | 46736457 | 127 | 100% | 2 |
| exon 11 | 46738137 | 46738212 | 75 | 100% | 1 |
| exon 12 | 46738343 | 46738474 | 131 | 100% | 2 |
| exon 13 | 46739026 | 46739137 | 111 | 100% | 1 |
| exon 14 | 46739295 | 46739419 | 124 | 100% | 2 |
| exon 15 | 46739809 | 46739888 | 79 | 100% | 2 |
| exon 16 | 46740209 | 46740389 | 180 | 100% | 2 |
| exon 17 | 46743488 | 46743652 | 164 | 100% | 2 |
| exon 18 | 46743743 | 46743955 | 212 | 100% | 2 |
| **SMUG1 (NM_014311.2), chromosome 12** | | | | | 9 |
| exon 1 | 54575878 | 54576407 | 529 | 100% | 6 |
| exon 2 | 54577439 | 54577731 | 292 | 100% | 3 |
| Total 313 Amplicons. Average amplicon size 187 bp. | | | | | |
