# Supplementary material for: Germline whole exome sequencing and large-scale replication identifies FANCM as a likely high grade serous ovarian cancer susceptibility gene
Source: Oncotarget. 2017 Mar 3;8(31):50930–40. doi: 10.18632/oncotarget.15871 (PMC5584218; doi:10.18632/oncotarget.15871)
Supplement: Supplementary file 3 [file oncotarget-08-50930-s003.docx]

**Supplementary Table 2a:** List of genes with at least one protein truncating mutation in 412 HGSOC cases from TCGA

| **gene** | **splice_**  **site** | **nonsense** | **stoploss** | **frameshift** | **total** | **total2** |
| --- | --- | --- | --- | --- | --- | --- |
| A1BG | 0 | 0 | 0 | 2 | 2 | 2 |
| A2M | 0 | 0 | 0 | 1 | 1 | 1 |
| A2ML1 | 1 | 0 | 0 | 1 | 2 | 1 |
| A4GALT | 0 | 0 | 0 | 2 | 2 | 2 |
| A4GNT | 0 | 2 | 0 | 0 | 2 | 2 |
| AACS | 0 | 1 | 0 | 1 | 2 | 2 |
| AADAC | 0 | 0 | 5 | 1 | 6 | 1 |
| AADACL2 | 0 | 1 | 0 | 0 | 1 | 1 |
| AADACL3 | 0 | 0 | 0 | 16 | 16 | 16 |
| AAGAB | 0 | 1 | 0 | 0 | 1 | 1 |
| AAK1 | 0 | 1 | 0 | 0 | 1 | 1 |
| AAMP | 0 | 0 | 0 | 1 | 1 | 1 |
| AARS2 | 0 | 0 | 0 | 3 | 3 | 3 |
| AARSD1 | 0 | 1 | 0 | 0 | 1 | 1 |
| AASDH | 0 | 0 | 0 | 4 | 4 | 4 |
| AATF | 1 | 0 | 0 | 0 | 1 | 0 |
| ABCA10 | 0 | 10 | 0 | 26 | 36 | 36 |
| ABCA13 | 2 | 20 | 36 | 2 | 60 | 22 |
| ABCA4 | 0 | 0 | 0 | 1 | 1 | 1 |
| ABCA5 | 0 | 1 | 0 | 0 | 1 | 1 |
| ABCA6 | 0 | 0 | 0 | 2 | 2 | 2 |
| ABCA8 | 0 | 2 | 0 | 1 | 3 | 3 |
| ABCA9 | 5 | 2 | 0 | 1 | 8 | 3 |
| ABCB10 | 0 | 1 | 0 | 2 | 3 | 3 |
| ABCB11 | 0 | 0 | 0 | 3 | 3 | 3 |
| ABCB5 | 12 | 2 | 0 | 0 | 14 | 2 |
| ABCB6 | 0 | 0 | 0 | 1 | 1 | 1 |
| ABCB7 | 13 | 0 | 0 | 0 | 13 | 0 |
| ABCB9 | 0 | 0 | 0 | 1 | 1 | 1 |
| ABCC1 | 1 | 4 | 0 | 1 | 6 | 5 |
| ABCC10 | 0 | 0 | 0 | 5 | 5 | 5 |
| ABCC11 | 7 | 3 | 0 | 0 | 10 | 3 |
| ABCC12 | 0 | 15 | 0 | 10 | 25 | 25 |
| ABCC2 | 0 | 2 | 0 | 0 | 2 | 2 |
| ABCC4 | 0 | 0 | 0 | 1 | 1 | 1 |
| ABCC5 | 0 | 1 | 0 | 5 | 6 | 6 |
| ABCC6 | 0 | 1 | 0 | 0 | 1 | 1 |
| ABCC9 | 2 | 0 | 0 | 0 | 2 | 0 |
| ABCD3 | 0 | 0 | 0 | 1 | 1 | 1 |
| ABCE1 | 0 | 0 | 0 | 3 | 3 | 3 |
| ABCF1 | 0 | 3 | 0 | 0 | 3 | 3 |
| ABCF3 | 1 | 0 | 0 | 2 | 3 | 2 |
| ABCG2 | 0 | 2 | 0 | 0 | 2 | 2 |
| ABCG4 | 0 | 0 | 0 | 1 | 1 | 1 |
| ABCG8 | 0 | 2 | 0 | 0 | 2 | 2 |
| ABHD12 | 0 | 1 | 0 | 0 | 1 | 1 |
| ABHD12B | 3 | 1 | 0 | 1 | 5 | 2 |
| ABHD14B | 0 | 1 | 0 | 3 | 4 | 4 |
| ABHD2 | 0 | 0 | 0 | 1 | 1 | 1 |
| ABHD3 | 1 | 0 | 0 | 1 | 2 | 1 |
| ABLIM1 | 1 | 0 | 0 | 0 | 1 | 0 |
| ABO | 0 | 0 | 0 | 3 | 3 | 3 |
| ABP1 | 0 | 0 | 0 | 1 | 1 | 1 |
| ABTB1 | 0 | 1 | 0 | 0 | 1 | 1 |
| ABTB2 | 0 | 1 | 0 | 1 | 2 | 2 |
| ACACA | 0 | 1 | 0 | 1 | 2 | 2 |
| ACACB | 0 | 1 | 0 | 5 | 6 | 6 |
| ACAD10 | 0 | 1 | 0 | 1 | 2 | 2 |
| ACAD11 | 10 | 9 | 0 | 5 | 24 | 14 |
| ACAD9 | 0 | 0 | 0 | 1 | 1 | 1 |
| ACADM | 0 | 0 | 0 | 1 | 1 | 1 |
| ACADVL | 1 | 0 | 0 | 0 | 1 | 0 |
| ACAN | 0 | 0 | 0 | 3 | 3 | 3 |
| ACBD4 | 0 | 1 | 0 | 0 | 1 | 1 |
| ACCN1 | 0 | 1 | 0 | 0 | 1 | 1 |
| ACCN2 | 0 | 8 | 0 | 0 | 8 | 8 |
| ACCN3 | 0 | 2 | 0 | 0 | 2 | 2 |
| ACE | 0 | 0 | 0 | 1 | 1 | 1 |
| ACIN1 | 0 | 0 | 0 | 11 | 11 | 11 |
| ACLY | 2 | 0 | 0 | 1 | 3 | 1 |
| ACOT1 | 0 | 0 | 0 | 1 | 1 | 1 |
| ACOT12 | 0 | 1 | 0 | 0 | 1 | 1 |
| ACOT13 | 1 | 0 | 0 | 0 | 1 | 0 |
| ACOT2 | 0 | 0 | 0 | 1 | 1 | 1 |
| ACOT4 | 0 | 0 | 0 | 8 | 8 | 8 |
| ACOT6 | 0 | 0 | 0 | 3 | 3 | 3 |
| ACOT8 | 0 | 0 | 0 | 4 | 4 | 4 |
| ACOX2 | 0 | 0 | 0 | 1 | 1 | 1 |
| ACOX3 | 0 | 1 | 0 | 0 | 1 | 1 |
| ACOXL | 0 | 3 | 0 | 0 | 3 | 3 |
| ACP6 | 0 | 0 | 0 | 1 | 1 | 1 |
| ACPL2 | 0 | 0 | 0 | 4 | 4 | 4 |
| ACPP | 0 | 0 | 0 | 2 | 2 | 2 |
| ACSBG1 | 0 | 0 | 0 | 1 | 1 | 1 |
| ACSBG2 | 3 | 0 | 0 | 2 | 5 | 2 |
| ACSF3 | 0 | 1 | 0 | 0 | 1 | 1 |
| ACSL5 | 1 | 0 | 0 | 0 | 1 | 0 |
| ACSL6 | 0 | 0 | 0 | 1 | 1 | 1 |
| ACSM2A | 0 | 16 | 0 | 0 | 16 | 16 |
| ACSM3 | 0 | 7 | 0 | 0 | 7 | 7 |
| ACSM4 | 1 | 3 | 0 | 1 | 5 | 4 |
| ACSM5 | 0 | 0 | 0 | 1 | 1 | 1 |
| ACSS2 | 0 | 0 | 0 | 11 | 11 | 11 |
| ACTA2 | 0 | 0 | 0 | 1 | 1 | 1 |
| ACTL6A | 0 | 0 | 0 | 1 | 1 | 1 |
| ACTL7B | 0 | 0 | 0 | 1 | 1 | 1 |
| ACTL9 | 0 | 0 | 0 | 2 | 2 | 2 |
| ACTN1 | 0 | 6 | 0 | 0 | 6 | 6 |
| ACTN3 | 0 | 1 | 0 | 0 | 1 | 1 |
| ACTR1B | 0 | 1 | 0 | 0 | 1 | 1 |
| ACTR3C | 0 | 4 | 0 | 0 | 4 | 4 |
| ACTR8 | 0 | 1 | 0 | 1 | 2 | 2 |
| ACTRT1 | 0 | 0 | 0 | 4 | 4 | 4 |
| ACVRL1 | 0 | 0 | 0 | 2 | 2 | 2 |
| ADA | 0 | 0 | 0 | 3 | 3 | 3 |
| ADAD1 | 1 | 0 | 0 | 0 | 1 | 0 |
| ADAM18 | 0 | 1 | 0 | 1 | 2 | 2 |
| ADAM19 | 0 | 1 | 0 | 2 | 3 | 3 |
| ADAM2 | 7 | 0 | 0 | 0 | 7 | 0 |
| ADAM20 | 0 | 0 | 0 | 1 | 1 | 1 |
| ADAM21 | 0 | 0 | 0 | 1 | 1 | 1 |
| ADAM22 | 0 | 0 | 0 | 1 | 1 | 1 |
| ADAM28 | 0 | 0 | 0 | 19 | 19 | 19 |
| ADAM29 | 0 | 1 | 3 | 0 | 4 | 1 |
| ADAM30 | 0 | 0 | 0 | 2 | 2 | 2 |
| ADAM33 | 0 | 0 | 0 | 1 | 1 | 1 |
| ADAM7 | 0 | 0 | 0 | 2 | 2 | 2 |
| ADAM8 | 0 | 0 | 0 | 8 | 8 | 8 |
| ADAM9 | 0 | 1 | 0 | 0 | 1 | 1 |
| ADAMDEC1 | 0 | 0 | 0 | 1 | 1 | 1 |
| ADAMTS12 | 1 | 0 | 0 | 0 | 1 | 0 |
| ADAMTS15 | 0 | 2 | 0 | 0 | 2 | 2 |
| ADAMTS16 | 0 | 1 | 0 | 0 | 1 | 1 |
| ADAMTS18 | 0 | 0 | 0 | 1 | 1 | 1 |
| ADAMTS20 | 1 | 0 | 0 | 0 | 1 | 0 |
| ADAMTS4 | 0 | 1 | 0 | 0 | 1 | 1 |
| ADAMTS7 | 0 | 2 | 0 | 0 | 2 | 2 |
| ADAMTS9 | 1 | 0 | 0 | 0 | 1 | 0 |
| ADAMTSL1 | 0 | 0 | 0 | 8 | 8 | 8 |
| ADAMTSL3 | 0 | 0 | 1 | 3 | 4 | 3 |
| ADAMTSL4 | 0 | 0 | 0 | 4 | 4 | 4 |
| ADAP1 | 0 | 1 | 0 | 0 | 1 | 1 |
| ADAP2 | 1 | 0 | 0 | 0 | 1 | 0 |
| ADARB1 | 0 | 0 | 0 | 1 | 1 | 1 |
| ADAT2 | 0 | 0 | 0 | 1 | 1 | 1 |
| ADCK2 | 1 | 0 | 0 | 0 | 1 | 0 |
| ADCY10 | 0 | 0 | 0 | 1 | 1 | 1 |
| ADCY2 | 0 | 0 | 0 | 1 | 1 | 1 |
| ADCY5 | 0 | 0 | 0 | 1 | 1 | 1 |
| ADCY7 | 0 | 0 | 0 | 2 | 2 | 2 |
| ADD3 | 0 | 1 | 0 | 1 | 2 | 2 |
| ADH1A | 1 | 0 | 0 | 0 | 1 | 0 |
| ADH5 | 0 | 0 | 0 | 1 | 1 | 1 |
| ADI1 | 0 | 4 | 0 | 0 | 4 | 4 |
| ADNP2 | 0 | 0 | 0 | 1 | 1 | 1 |
| ADPRH | 0 | 1 | 0 | 0 | 1 | 1 |
| ADPRHL1 | 0 | 0 | 0 | 1 | 1 | 1 |
| ADRB3 | 1 | 0 | 0 | 0 | 1 | 0 |
| ADRBK1 | 0 | 0 | 0 | 1 | 1 | 1 |
| ADRBK2 | 0 | 1 | 0 | 0 | 1 | 1 |
| ADSSL1 | 0 | 0 | 0 | 1 | 1 | 1 |
| AFAP1L1 | 0 | 1 | 0 | 0 | 1 | 1 |
| AFAP1L2 | 0 | 0 | 0 | 2 | 2 | 2 |
| AFM | 0 | 0 | 0 | 1 | 1 | 1 |
| AFMID | 1 | 0 | 0 | 0 | 1 | 0 |
| AGAP2 | 0 | 2 | 0 | 0 | 2 | 2 |
| AGAP5 | 0 | 3 | 0 | 0 | 3 | 3 |
| AGAP6 | 0 | 3 | 0 | 17 | 20 | 20 |
| AGBL1 | 5 | 1 | 0 | 0 | 6 | 1 |
| AGBL2 | 0 | 0 | 0 | 1 | 1 | 1 |
| AGER | 0 | 1 | 0 | 0 | 1 | 1 |
| AGL | 1 | 0 | 0 | 0 | 1 | 0 |
| AGMAT | 1 | 0 | 0 | 0 | 1 | 0 |
| AGMO | 0 | 0 | 0 | 2 | 2 | 2 |
| AGPAT3 | 0 | 0 | 0 | 1 | 1 | 1 |
| AGPAT4 | 0 | 6 | 0 | 0 | 6 | 6 |
| AGPAT6 | 0 | 0 | 4 | 3 | 7 | 3 |
| AGPAT9 | 0 | 1 | 0 | 0 | 1 | 1 |
| AGPHD1 | 0 | 4 | 0 | 0 | 4 | 4 |
| AGR2 | 0 | 1 | 0 | 0 | 1 | 1 |
| AGR3 | 1 | 0 | 0 | 0 | 1 | 0 |
| AGRP | 0 | 0 | 0 | 1 | 1 | 1 |
| AGTPBP1 | 0 | 0 | 0 | 2 | 2 | 2 |
| AGTR2 | 0 | 0 | 0 | 1 | 1 | 1 |
| AGXT2 | 2 | 0 | 0 | 0 | 2 | 0 |
| AHCTF1 | 6 | 0 | 0 | 0 | 6 | 0 |
| AHDC1 | 0 | 0 | 0 | 1 | 1 | 1 |
| AHI1 | 0 | 5 | 0 | 0 | 5 | 5 |
| AHNAK2 | 0 | 1 | 0 | 10 | 11 | 11 |
| AHR | 0 | 0 | 0 | 1 | 1 | 1 |
| AHSA1 | 0 | 0 | 1 | 0 | 1 | 0 |
| AHSA2 | 0 | 1 | 0 | 1 | 2 | 2 |
| AHSG | 0 | 1 | 0 | 0 | 1 | 1 |
| AHSP | 0 | 0 | 0 | 1 | 1 | 1 |
| AIFM3 | 0 | 0 | 0 | 1 | 1 | 1 |
| AIM1 | 0 | 0 | 0 | 2 | 2 | 2 |
| AIM2 | 0 | 0 | 2 | 12 | 14 | 12 |
| AIMP1 | 0 | 0 | 0 | 1 | 1 | 1 |
| AIP | 0 | 0 | 0 | 2 | 2 | 2 |
| AIPL1 | 1 | 0 | 0 | 0 | 1 | 0 |
| AK7 | 0 | 3 | 0 | 0 | 3 | 3 |
| AK8 | 0 | 0 | 0 | 1 | 1 | 1 |
| AKAP10 | 0 | 0 | 0 | 1 | 1 | 1 |
| AKAP13 | 0 | 2 | 0 | 0 | 2 | 2 |
| AKAP3 | 0 | 0 | 0 | 8 | 8 | 8 |
| AKAP7 | 1 | 0 | 0 | 0 | 1 | 0 |
| AKAP8 | 3 | 0 | 0 | 0 | 3 | 0 |
| AKD1 | 0 | 2 | 0 | 0 | 2 | 2 |
| AKIRIN2 | 0 | 0 | 0 | 1 | 1 | 1 |
| AKNA | 1 | 0 | 0 | 1 | 2 | 1 |
| AKNAD1 | 2 | 0 | 0 | 0 | 2 | 0 |
| AKR1B10 | 0 | 0 | 0 | 1 | 1 | 1 |
| AKR1C3 | 0 | 1 | 0 | 0 | 1 | 1 |
| AKR7A2 | 0 | 1 | 0 | 0 | 1 | 1 |
| AKT1 | 0 | 7 | 0 | 0 | 7 | 7 |
| AKT3 | 1 | 0 | 0 | 0 | 1 | 0 |
| ALAS1 | 0 | 0 | 0 | 8 | 8 | 8 |
| ALDH18A1 | 0 | 1 | 0 | 1 | 2 | 2 |
| ALDH1B1 | 0 | 1 | 0 | 8 | 9 | 9 |
| ALDH1L1 | 1 | 0 | 0 | 0 | 1 | 0 |
| ALDH1L2 | 0 | 2 | 0 | 0 | 2 | 2 |
| ALDH2 | 0 | 0 | 0 | 3 | 3 | 3 |
| ALDH3A2 | 0 | 1 | 0 | 0 | 1 | 1 |
| ALDH4A1 | 0 | 0 | 0 | 2 | 2 | 2 |
| ALDH5A1 | 1 | 0 | 0 | 1 | 2 | 1 |
| ALDH6A1 | 0 | 0 | 0 | 1 | 1 | 1 |
| ALDH9A1 | 0 | 1 | 0 | 0 | 1 | 1 |
| ALDOB | 0 | 2 | 0 | 0 | 2 | 2 |
| ALDOC | 0 | 0 | 0 | 1 | 1 | 1 |
| ALG1 | 0 | 1 | 0 | 0 | 1 | 1 |
| ALG10 | 0 | 0 | 0 | 1 | 1 | 1 |
| ALG10B | 0 | 1 | 0 | 0 | 1 | 1 |
| ALG1L2 | 0 | 1 | 0 | 0 | 1 | 1 |
| ALG8 | 0 | 1 | 0 | 0 | 1 | 1 |
| ALG9 | 0 | 0 | 0 | 1 | 1 | 1 |
| ALK | 0 | 1 | 0 | 0 | 1 | 1 |
| ALKBH3 | 0 | 1 | 0 | 2 | 3 | 3 |
| ALLC | 0 | 1 | 0 | 0 | 1 | 1 |
| ALOX12 | 0 | 1 | 0 | 0 | 1 | 1 |
| ALOX15B | 0 | 2 | 0 | 1 | 3 | 3 |
| ALOXE3 | 0 | 1 | 0 | 0 | 1 | 1 |
| ALPK1 | 0 | 1 | 0 | 0 | 1 | 1 |
| ALPK2 | 0 | 2 | 0 | 9 | 11 | 11 |
| ALPP | 0 | 1 | 0 | 1 | 2 | 2 |
| ALS2 | 2 | 0 | 0 | 0 | 2 | 0 |
| ALS2CL | 0 | 0 | 0 | 1 | 1 | 1 |
| ALS2CR11 | 0 | 1 | 0 | 1 | 2 | 2 |
| ALS2CR12 | 0 | 1 | 0 | 0 | 1 | 1 |
| ALS2CR8 | 0 | 0 | 0 | 4 | 4 | 4 |
| AMBN | 1 | 0 | 0 | 0 | 1 | 0 |
| AMHR2 | 0 | 0 | 0 | 3 | 3 | 3 |
| AMICA1 | 1 | 0 | 0 | 0 | 1 | 0 |
| AMOTL1 | 1 | 0 | 0 | 0 | 1 | 0 |
| AMPD2 | 1 | 0 | 0 | 0 | 1 | 0 |
| AMY2B | 1 | 0 | 0 | 0 | 1 | 0 |
| AMZ1 | 0 | 1 | 0 | 0 | 1 | 1 |
| AMZ2 | 0 | 0 | 0 | 2 | 2 | 2 |
| ANAPC4 | 0 | 1 | 0 | 0 | 1 | 1 |
| ANGEL2 | 0 | 0 | 0 | 3 | 3 | 3 |
| ANGPT2 | 0 | 1 | 0 | 0 | 1 | 1 |
| ANGPT4 | 0 | 0 | 0 | 1 | 1 | 1 |
| ANGPTL3 | 1 | 0 | 0 | 1 | 2 | 1 |
| ANGPTL4 | 0 | 0 | 0 | 1 | 1 | 1 |
| ANGPTL5 | 0 | 1 | 0 | 0 | 1 | 1 |
| ANGPTL6 | 0 | 1 | 0 | 0 | 1 | 1 |
| ANK1 | 0 | 0 | 0 | 8 | 8 | 8 |
| ANK3 | 2 | 1 | 0 | 0 | 3 | 1 |
| ANKFN1 | 1 | 0 | 0 | 2 | 3 | 2 |
| ANKHD1 | 0 | 2 | 0 | 0 | 2 | 2 |
| ANKLE1 | 0 | 0 | 0 | 1 | 1 | 1 |
| ANKMY2 | 0 | 1 | 0 | 0 | 1 | 1 |
| ANKRD1 | 1 | 0 | 0 | 0 | 1 | 0 |
| ANKRD16 | 0 | 0 | 0 | 1 | 1 | 1 |
| ANKRD18B | 0 | 1 | 0 | 0 | 1 | 1 |
| ANKRD20A4 | 0 | 0 | 0 | 12 | 12 | 12 |
| ANKRD22 | 1 | 0 | 0 | 0 | 1 | 0 |
| ANKRD23 | 0 | 0 | 0 | 1 | 1 | 1 |
| ANKRD24 | 0 | 0 | 0 | 1 | 1 | 1 |
| ANKRD27 | 0 | 0 | 0 | 1 | 1 | 1 |
| ANKRD30A | 6 | 18 | 0 | 25 | 49 | 43 |
| ANKRD35 | 0 | 3 | 1 | 1 | 5 | 4 |
| ANKRD36 | 3 | 0 | 0 | 31 | 34 | 31 |
| ANKRD36B | 1 | 0 | 0 | 4 | 5 | 4 |
| ANKRD37 | 0 | 0 | 0 | 1 | 1 | 1 |
| ANKRD6 | 1 | 0 | 0 | 0 | 1 | 0 |
| ANLN | 1 | 1 | 0 | 0 | 2 | 1 |
| ANO1 | 0 | 1 | 0 | 0 | 1 | 1 |
| ANO10 | 0 | 0 | 0 | 1 | 1 | 1 |
| ANO5 | 0 | 1 | 0 | 3 | 4 | 4 |
| ANO7 | 0 | 0 | 0 | 9 | 9 | 9 |
| ANO8 | 3 | 0 | 0 | 0 | 3 | 0 |
| ANPEP | 0 | 1 | 0 | 0 | 1 | 1 |
| ANTXR2 | 0 | 0 | 0 | 14 | 14 | 14 |
| ANUBL1 | 1 | 0 | 0 | 2 | 3 | 2 |
| ANXA10 | 1 | 4 | 0 | 0 | 5 | 4 |
| ANXA5 | 0 | 1 | 0 | 0 | 1 | 1 |
| ANXA7 | 0 | 1 | 0 | 0 | 1 | 1 |
| AOAH | 0 | 3 | 0 | 1 | 4 | 4 |
| AOC2 | 0 | 4 | 0 | 8 | 12 | 12 |
| AOX1 | 0 | 3 | 0 | 0 | 3 | 3 |
| AP1G2 | 1 | 0 | 0 | 0 | 1 | 0 |
| AP1M1 | 0 | 0 | 0 | 1 | 1 | 1 |
| AP1M2 | 2 | 0 | 0 | 9 | 11 | 9 |
| AP1S1 | 0 | 0 | 0 | 1 | 1 | 1 |
| AP2A1 | 0 | 0 | 0 | 1 | 1 | 1 |
| AP2A2 | 0 | 0 | 0 | 2 | 2 | 2 |
| AP2B1 | 1 | 0 | 0 | 2 | 3 | 2 |
| AP2M1 | 0 | 0 | 4 | 0 | 4 | 0 |
| AP3B2 | 0 | 0 | 0 | 6 | 6 | 6 |
| AP3M1 | 0 | 20 | 0 | 0 | 20 | 20 |
| AP3M2 | 0 | 0 | 0 | 3 | 3 | 3 |
| AP3S1 | 0 | 0 | 0 | 1 | 1 | 1 |
| AP4B1 | 0 | 0 | 0 | 1 | 1 | 1 |
| AP4E1 | 0 | 0 | 0 | 1 | 1 | 1 |
| AP4M1 | 0 | 1 | 0 | 0 | 1 | 1 |
| APBA1 | 0 | 0 | 0 | 1 | 1 | 1 |
| APC | 0 | 1 | 0 | 2 | 3 | 3 |
| APCS | 0 | 0 | 0 | 1 | 1 | 1 |
| APEX1 | 0 | 0 | 0 | 3 | 3 | 3 |
| APEX2 | 0 | 0 | 0 | 1 | 1 | 1 |
| APIP | 0 | 4 | 0 | 0 | 4 | 4 |
| APLF | 0 | 0 | 0 | 13 | 13 | 13 |
| APOA2 | 9 | 0 | 0 | 0 | 9 | 0 |
| APOB | 0 | 5 | 0 | 0 | 5 | 5 |
| APOBEC1 | 0 | 3 | 0 | 0 | 3 | 3 |
| APOBEC3A | 0 | 0 | 0 | 1 | 1 | 1 |
| APOBEC3B | 1 | 0 | 0 | 2 | 3 | 2 |
| APOBEC3G | 0 | 1 | 0 | 0 | 1 | 1 |
| APOL4 | 20 | 0 | 1 | 0 | 21 | 0 |
| APOL5 | 0 | 1 | 0 | 4 | 5 | 5 |
| APPBP2 | 0 | 0 | 0 | 1 | 1 | 1 |
| APPL1 | 3 | 5 | 0 | 1 | 9 | 6 |
| APTX | 7 | 2 | 0 | 0 | 9 | 2 |
| AQP10 | 0 | 0 | 0 | 1 | 1 | 1 |
| AQP12A | 0 | 0 | 0 | 5 | 5 | 5 |
| AQP12B | 0 | 1 | 0 | 0 | 1 | 1 |
| AQP6 | 0 | 0 | 0 | 2 | 2 | 2 |
| AQP7 | 0 | 8 | 0 | 2 | 10 | 10 |
| AQP9 | 0 | 0 | 0 | 2 | 2 | 2 |
| ARAP1 | 0 | 7 | 0 | 0 | 7 | 7 |
| ARF6 | 0 | 0 | 0 | 2 | 2 | 2 |
| ARHGAP10 | 1 | 0 | 0 | 3 | 4 | 3 |
| ARHGAP15 | 0 | 1 | 0 | 0 | 1 | 1 |
| ARHGAP25 | 1 | 0 | 0 | 0 | 1 | 0 |
| ARHGAP27 | 0 | 0 | 0 | 1 | 1 | 1 |
| ARHGAP29 | 0 | 0 | 0 | 2 | 2 | 2 |
| ARHGAP33 | 0 | 0 | 0 | 1 | 1 | 1 |
| ARHGAP36 | 0 | 10 | 0 | 0 | 10 | 10 |
| ARHGAP40 | 0 | 13 | 0 | 0 | 13 | 13 |
| ARHGAP44 | 0 | 0 | 0 | 2 | 2 | 2 |
| ARHGAP5 | 0 | 2 | 0 | 8 | 10 | 10 |
| ARHGAP8 | 0 | 0 | 0 | 1 | 1 | 1 |
| ARHGAP9 | 1 | 0 | 0 | 0 | 1 | 0 |
| ARHGDIA | 0 | 0 | 0 | 1 | 1 | 1 |
| ARHGEF15 | 0 | 0 | 0 | 1 | 1 | 1 |
| ARHGEF2 | 0 | 3 | 0 | 0 | 3 | 3 |
| ARHGEF35 | 0 | 0 | 0 | 2 | 2 | 2 |
| ARHGEF37 | 4 | 0 | 0 | 0 | 4 | 0 |
| ARHGEF4 | 2 | 0 | 0 | 1 | 3 | 1 |
| ARHGEF40 | 1 | 0 | 0 | 0 | 1 | 0 |
| ARHGEF5 | 0 | 1 | 0 | 0 | 1 | 1 |
| ARHGEF6 | 0 | 0 | 0 | 19 | 19 | 19 |
| ARID1A | 0 | 0 | 0 | 8 | 8 | 8 |
| ARID4A | 2 | 0 | 0 | 0 | 2 | 0 |
| ARL11 | 0 | 6 | 0 | 0 | 6 | 6 |
| ARL13B | 0 | 1 | 0 | 0 | 1 | 1 |
| ARL6IP1 | 0 | 0 | 0 | 1 | 1 | 1 |
| ARMC3 | 0 | 7 | 0 | 5 | 12 | 12 |
| ARMC5 | 0 | 0 | 0 | 1 | 1 | 1 |
| ARMC6 | 0 | 2 | 0 | 0 | 2 | 2 |
| ARMCX2 | 0 | 0 | 0 | 2 | 2 | 2 |
| ARMCX6 | 0 | 0 | 0 | 1 | 1 | 1 |
| ARMS2 | 0 | 0 | 0 | 1 | 1 | 1 |
| ARNTL2 | 1 | 0 | 0 | 0 | 1 | 0 |
| ARSD | 0 | 0 | 0 | 1 | 1 | 1 |
| ARSE | 0 | 1 | 0 | 0 | 1 | 1 |
| ARSJ | 0 | 0 | 0 | 1 | 1 | 1 |
| ART1 | 0 | 1 | 0 | 0 | 1 | 1 |
| ART3 | 0 | 2 | 0 | 0 | 2 | 2 |
| ART5 | 0 | 0 | 0 | 2 | 2 | 2 |
| ARV1 | 0 | 0 | 0 | 1 | 1 | 1 |
| ASAH1 | 1 | 0 | 0 | 1 | 2 | 1 |
| ASAP2 | 0 | 0 | 0 | 1 | 1 | 1 |
| ASB11 | 0 | 5 | 0 | 0 | 5 | 5 |
| ASB12 | 0 | 0 | 0 | 1 | 1 | 1 |
| ASB15 | 0 | 2 | 0 | 13 | 15 | 15 |
| ASB18 | 0 | 0 | 0 | 1 | 1 | 1 |
| ASB2 | 0 | 1 | 0 | 0 | 1 | 1 |
| ASB6 | 0 | 1 | 0 | 0 | 1 | 1 |
| ASB8 | 0 | 0 | 0 | 1 | 1 | 1 |
| ASCL3 | 0 | 3 | 0 | 2 | 5 | 5 |
| ASCL4 | 0 | 0 | 0 | 1 | 1 | 1 |
| ASH1L | 0 | 0 | 0 | 1 | 1 | 1 |
| ASPDH | 0 | 0 | 0 | 1 | 1 | 1 |
| ASPN | 0 | 1 | 0 | 0 | 1 | 1 |
| ASTE1 | 0 | 0 | 0 | 1 | 1 | 1 |
| ASTL | 0 | 0 | 0 | 3 | 3 | 3 |
| ASTN1 | 0 | 0 | 0 | 2 | 2 | 2 |
| ASXL1 | 0 | 0 | 0 | 1 | 1 | 1 |
| ATAD5 | 0 | 0 | 0 | 3 | 3 | 3 |
| ATF5 | 0 | 1 | 0 | 0 | 1 | 1 |
| ATF7IP | 1 | 0 | 0 | 0 | 1 | 0 |
| ATG3 | 0 | 0 | 0 | 7 | 7 | 7 |
| ATG4C | 0 | 0 | 0 | 3 | 3 | 3 |
| ATG4D | 0 | 1 | 0 | 0 | 1 | 1 |
| ATG7 | 0 | 1 | 0 | 1 | 2 | 2 |
| ATG9A | 0 | 0 | 0 | 2 | 2 | 2 |
| ATL1 | 0 | 1 | 0 | 1 | 2 | 2 |
| ATM | 1 | 2 | 0 | 0 | 3 | 2 |
| ATOH8 | 0 | 0 | 0 | 2 | 2 | 2 |
| ATP10B | 1 | 0 | 0 | 0 | 1 | 0 |
| ATP10D | 0 | 0 | 0 | 4 | 4 | 4 |
| ATP11A | 0 | 0 | 0 | 3 | 3 | 3 |
| ATP12A | 1 | 0 | 0 | 1 | 2 | 1 |
| ATP13A5 | 0 | 6 | 0 | 2 | 8 | 8 |
| ATP1A4 | 0 | 0 | 0 | 1 | 1 | 1 |
| ATP2B2 | 1 | 0 | 0 | 0 | 1 | 0 |
| ATP2B4 | 0 | 1 | 0 | 0 | 1 | 1 |
| ATP2C1 | 0 | 1 | 0 | 1 | 2 | 2 |
| ATP2C2 | 7 | 0 | 0 | 0 | 7 | 0 |
| ATP4A | 0 | 0 | 0 | 2 | 2 | 2 |
| ATP5J | 0 | 0 | 0 | 3 | 3 | 3 |
| ATP5L2 | 0 | 0 | 0 | 1 | 1 | 1 |
| ATP5O | 0 | 0 | 0 | 1 | 1 | 1 |
| ATP6V0A2 | 0 | 1 | 0 | 0 | 1 | 1 |
| ATP6V0D1 | 0 | 0 | 0 | 3 | 3 | 3 |
| ATP6V1C2 | 1 | 0 | 0 | 0 | 1 | 0 |
| ATP6V1G3 | 0 | 5 | 0 | 0 | 5 | 5 |
| ATP6V1H | 0 | 0 | 0 | 1 | 1 | 1 |
| ATP7B | 0 | 0 | 0 | 2 | 2 | 2 |
| ATP8B2 | 0 | 0 | 0 | 1 | 1 | 1 |
| ATP8B3 | 0 | 0 | 4 | 0 | 4 | 0 |
| ATP8B4 | 0 | 0 | 0 | 2 | 2 | 2 |
| ATP9B | 0 | 6 | 0 | 1 | 7 | 7 |
| ATPAF2 | 0 | 0 | 0 | 5 | 5 | 5 |
| ATR | 0 | 1 | 0 | 1 | 2 | 2 |
| ATRX | 0 | 1 | 0 | 6 | 7 | 7 |
| ATXN2 | 1 | 0 | 0 | 0 | 1 | 0 |
| ATXN3L | 0 | 1 | 0 | 0 | 1 | 1 |
| AURKB | 0 | 0 | 0 | 1 | 1 | 1 |
| AURKC | 0 | 0 | 0 | 1 | 1 | 1 |
| AVIL | 0 | 1 | 0 | 0 | 1 | 1 |
| AVPR1B | 0 | 1 | 0 | 1 | 2 | 2 |
| AWAT1 | 0 | 0 | 0 | 2 | 2 | 2 |
| AZI1 | 0 | 0 | 0 | 2 | 2 | 2 |
| AZU1 | 0 | 0 | 0 | 1 | 1 | 1 |
| B3GALNT2 | 0 | 0 | 0 | 1 | 1 | 1 |
| B3GNT4 | 0 | 4 | 0 | 2 | 6 | 6 |
| B3GNT6 | 0 | 0 | 0 | 2 | 2 | 2 |
| B3GNTL1 | 1 | 0 | 0 | 0 | 1 | 0 |
| B4GALNT3 | 0 | 1 | 0 | 0 | 1 | 1 |
| B4GALT3 | 0 | 0 | 0 | 3 | 3 | 3 |
| B7H6 | 0 | 0 | 0 | 5 | 5 | 5 |
| BACH2 | 0 | 0 | 0 | 1 | 1 | 1 |
| BAG4 | 0 | 1 | 0 | 0 | 1 | 1 |
| BAHD1 | 0 | 1 | 0 | 1 | 2 | 2 |
| BAI2 | 0 | 0 | 0 | 1 | 1 | 1 |
| BAI3 | 0 | 5 | 0 | 0 | 5 | 5 |
| BAIAP2L1 | 4 | 1 | 0 | 0 | 5 | 1 |
| BAIAP2L2 | 0 | 0 | 0 | 1 | 1 | 1 |
| BARHL1 | 0 | 0 | 0 | 1 | 1 | 1 |
| BARX2 | 0 | 0 | 0 | 1 | 1 | 1 |
| BAX | 0 | 0 | 0 | 1 | 1 | 1 |
| BAZ1A | 0 | 0 | 0 | 1 | 1 | 1 |
| BAZ1B | 0 | 4 | 0 | 5 | 9 | 9 |
| BAZ2A | 0 | 0 | 0 | 1 | 1 | 1 |
| BBOX1 | 0 | 1 | 0 | 0 | 1 | 1 |
| BBS10 | 0 | 0 | 0 | 1 | 1 | 1 |
| BBS12 | 0 | 0 | 0 | 5 | 5 | 5 |
| BBS7 | 0 | 0 | 0 | 1 | 1 | 1 |
| BBS9 | 1 | 0 | 0 | 0 | 1 | 0 |
| BCAP29 | 0 | 0 | 0 | 1 | 1 | 1 |
| BCAT1 | 0 | 1 | 0 | 0 | 1 | 1 |
| BCCIP | 1 | 0 | 0 | 1 | 2 | 1 |
| BCDIN3D | 0 | 0 | 0 | 1 | 1 | 1 |
| BCHE | 0 | 0 | 0 | 1 | 1 | 1 |
| BCL2L12 | 0 | 2 | 0 | 0 | 2 | 2 |
| BCL2L14 | 0 | 0 | 0 | 1 | 1 | 1 |
| BCL2L2-PABPN1 | 0 | 0 | 0 | 2 | 2 | 2 |
| BCL3 | 0 | 0 | 0 | 1 | 1 | 1 |
| BCL6 | 0 | 0 | 0 | 1 | 1 | 1 |
| BCL7C | 0 | 0 | 0 | 1 | 1 | 1 |
| BCL9 | 0 | 0 | 0 | 1 | 1 | 1 |
| BCL9L | 0 | 0 | 0 | 1 | 1 | 1 |
| BCLAF1 | 0 | 5 | 0 | 15 | 20 | 20 |
| BCO2 | 0 | 0 | 0 | 1 | 1 | 1 |
| BCOR | 0 | 0 | 0 | 1 | 1 | 1 |
| BCORL1 | 0 | 0 | 0 | 1 | 1 | 1 |
| BDKRB1 | 0 | 2 | 0 | 0 | 2 | 2 |
| BEND2 | 0 | 0 | 0 | 2 | 2 | 2 |
| BEND5 | 0 | 0 | 0 | 1 | 1 | 1 |
| BEND7 | 0 | 0 | 0 | 2 | 2 | 2 |
| BEST3 | 0 | 0 | 0 | 15 | 15 | 15 |
| BFSP1 | 1 | 0 | 0 | 4 | 5 | 4 |
| BFSP2 | 0 | 0 | 0 | 1 | 1 | 1 |
| BGLAP | 0 | 1 | 0 | 0 | 1 | 1 |
| BHMT2 | 1 | 0 | 0 | 0 | 1 | 0 |
| BICD2 | 0 | 0 | 0 | 1 | 1 | 1 |
| BIRC6 | 0 | 3 | 0 | 1 | 4 | 4 |
| BLM | 0 | 0 | 0 | 2 | 2 | 2 |
| BLVRA | 0 | 1 | 0 | 0 | 1 | 1 |
| BLZF1 | 0 | 2 | 0 | 0 | 2 | 2 |
| BMP2K | 0 | 6 | 0 | 0 | 6 | 6 |
| BMP7 | 0 | 0 | 0 | 1 | 1 | 1 |
| BMP8A | 1 | 0 | 0 | 0 | 1 | 0 |
| BMPR2 | 0 | 0 | 0 | 2 | 2 | 2 |
| BMX | 0 | 0 | 0 | 1 | 1 | 1 |
| BORA | 0 | 0 | 0 | 1 | 1 | 1 |
| BPGM | 0 | 0 | 1 | 0 | 1 | 0 |
| BPHL | 3 | 0 | 0 | 0 | 3 | 0 |
| BPI | 0 | 0 | 0 | 1 | 1 | 1 |
| BPIFA1 | 1 | 0 | 0 | 0 | 1 | 0 |
| BPIFA2 | 0 | 1 | 0 | 0 | 1 | 1 |
| BPIFA3 | 0 | 2 | 0 | 0 | 2 | 2 |
| BPIFB3 | 6 | 1 | 0 | 0 | 7 | 1 |
| BPIFB6 | 0 | 1 | 0 | 1 | 2 | 2 |
| BRAP | 0 | 0 | 0 | 1 | 1 | 1 |
| BRCA1 | 2 | 6 | 0 | 28 | 36 | 34 |
| BRCA2 | 1 | 6 | 0 | 19 | 26 | 25 |
| BRD4 | 0 | 2 | 0 | 0 | 2 | 2 |
| BRD9 | 0 | 0 | 0 | 1 | 1 | 1 |
| BRDT | 0 | 1 | 0 | 0 | 1 | 1 |
| BRIP1 | 0 | 0 | 0 | 3 | 3 | 3 |
| BRIX1 | 2 | 0 | 0 | 0 | 2 | 0 |
| BRMS1 | 1 | 0 | 0 | 0 | 1 | 0 |
| BROX | 0 | 0 | 0 | 12 | 12 | 12 |
| BRP44 | 0 | 0 | 0 | 2 | 2 | 2 |
| BRPF1 | 0 | 1 | 0 | 4 | 5 | 5 |
| BRSK1 | 1 | 0 | 0 | 3 | 4 | 3 |
| BRWD3 | 0 | 0 | 0 | 1 | 1 | 1 |
| BSN | 0 | 0 | 0 | 2 | 2 | 2 |
| BSPRY | 0 | 0 | 1 | 0 | 1 | 0 |
| BTAF1 | 0 | 3 | 0 | 0 | 3 | 3 |
| BTBD6 | 0 | 0 | 0 | 2 | 2 | 2 |
| BTBD7 | 0 | 0 | 0 | 2 | 2 | 2 |
| BTD | 0 | 4 | 0 | 0 | 4 | 4 |
| BTK | 0 | 1 | 0 | 1 | 2 | 2 |
| BTN2A1 | 0 | 2 | 0 | 0 | 2 | 2 |
| BTN2A2 | 1 | 0 | 0 | 0 | 1 | 0 |
| BTN3A2 | 0 | 1 | 0 | 0 | 1 | 1 |
| BTN3A3 | 0 | 6 | 0 | 0 | 6 | 6 |
| BTNL9 | 0 | 0 | 0 | 7 | 7 | 7 |
| BUD31 | 0 | 0 | 0 | 1 | 1 | 1 |
| BYSL | 0 | 1 | 0 | 0 | 1 | 1 |
| C10ORF10 | 0 | 1 | 0 | 0 | 1 | 1 |
| C10ORF11 | 0 | 0 | 0 | 3 | 3 | 3 |
| C10ORF113 | 1 | 0 | 0 | 0 | 1 | 0 |
| C10ORF12 | 0 | 0 | 0 | 2 | 2 | 2 |
| C10ORF120 | 0 | 1 | 0 | 0 | 1 | 1 |
| C10ORF128 | 0 | 2 | 0 | 0 | 2 | 2 |
| C10ORF131 | 0 | 1 | 0 | 0 | 1 | 1 |
| C10ORF27 | 0 | 3 | 0 | 3 | 6 | 6 |
| C10ORF32 | 2 | 0 | 0 | 0 | 2 | 0 |
| C10ORF53 | 0 | 2 | 0 | 1 | 3 | 3 |
| C10ORF62 | 0 | 1 | 0 | 0 | 1 | 1 |
| C10ORF68 | 0 | 1 | 0 | 10 | 11 | 11 |
| C10ORF71 | 0 | 0 | 0 | 2 | 2 | 2 |
| C10ORF90 | 0 | 1 | 0 | 0 | 1 | 1 |
| C10ORF91 | 0 | 0 | 0 | 3 | 3 | 3 |
| C10ORF92 | 0 | 1 | 0 | 0 | 1 | 1 |
| C11ORF1 | 0 | 11 | 0 | 0 | 11 | 11 |
| C11ORF16 | 0 | 0 | 0 | 2 | 2 | 2 |
| C11ORF40 | 4 | 0 | 0 | 0 | 4 | 0 |
| C11ORF54 | 0 | 1 | 0 | 1 | 2 | 2 |
| C11ORF63 | 0 | 0 | 0 | 1 | 1 | 1 |
| C11ORF65 | 0 | 3 | 0 | 0 | 3 | 3 |
| C11ORF71 | 0 | 2 | 0 | 0 | 2 | 2 |
| C11ORF82 | 0 | 0 | 0 | 1 | 1 | 1 |
| C11ORF84 | 1 | 0 | 0 | 0 | 1 | 0 |
| C12ORF26 | 0 | 0 | 0 | 3 | 3 | 3 |
| C12ORF35 | 0 | 1 | 0 | 0 | 1 | 1 |
| C12ORF39 | 1 | 0 | 0 | 0 | 1 | 0 |
| C12ORF4 | 0 | 1 | 0 | 0 | 1 | 1 |
| C12ORF48 | 0 | 0 | 0 | 1 | 1 | 1 |
| C12ORF49 | 0 | 1 | 0 | 0 | 1 | 1 |
| C12ORF50 | 0 | 1 | 0 | 0 | 1 | 1 |
| C12ORF56 | 0 | 1 | 0 | 1 | 2 | 2 |
| C12ORF65 | 0 | 0 | 0 | 1 | 1 | 1 |
| C12ORF69 | 0 | 1 | 0 | 0 | 1 | 1 |
| C12ORF74 | 0 | 1 | 0 | 0 | 1 | 1 |
| C13ORF16 | 0 | 0 | 0 | 2 | 2 | 2 |
| C14ORF105 | 0 | 1 | 0 | 0 | 1 | 1 |
| C14ORF118 | 0 | 3 | 0 | 0 | 3 | 3 |
| C14ORF133 | 0 | 1 | 0 | 5 | 6 | 6 |
| C14ORF135 | 0 | 0 | 0 | 1 | 1 | 1 |
| C14ORF176 | 0 | 0 | 0 | 1 | 1 | 1 |
| C14ORF182 | 0 | 0 | 0 | 2 | 2 | 2 |
| C14ORF183 | 0 | 0 | 0 | 1 | 1 | 1 |
| C14ORF39 | 1 | 0 | 0 | 0 | 1 | 0 |
| C14ORF93 | 1 | 0 | 0 | 0 | 1 | 0 |
| C15ORF32 | 0 | 1 | 0 | 0 | 1 | 1 |
| C15ORF33 | 0 | 2 | 0 | 0 | 2 | 2 |
| C15ORF59 | 0 | 0 | 0 | 1 | 1 | 1 |
| C16ORF55 | 0 | 0 | 0 | 3 | 3 | 3 |
| C16ORF62 | 0 | 3 | 0 | 0 | 3 | 3 |
| C16ORF89 | 0 | 1 | 0 | 0 | 1 | 1 |
| C16ORF93 | 0 | 0 | 0 | 1 | 1 | 1 |
| C17ORF101 | 5 | 0 | 0 | 0 | 5 | 0 |
| C17ORF104 | 0 | 3 | 0 | 0 | 3 | 3 |
| C17ORF28 | 2 | 0 | 0 | 0 | 2 | 0 |
| C17ORF56 | 1 | 0 | 0 | 0 | 1 | 0 |
| C17ORF57 | 12 | 12 | 0 | 2 | 26 | 14 |
| C17ORF58 | 0 | 0 | 5 | 0 | 5 | 0 |
| C17ORF63 | 0 | 1 | 0 | 0 | 1 | 1 |
| C17ORF66 | 0 | 2 | 0 | 0 | 2 | 2 |
| C17ORF74 | 0 | 0 | 0 | 1 | 1 | 1 |
| C17ORF77 | 0 | 7 | 0 | 0 | 7 | 7 |
| C17ORF80 | 2 | 0 | 0 | 0 | 2 | 0 |
| C17ORF81 | 0 | 2 | 0 | 0 | 2 | 2 |
| C17ORF85 | 0 | 3 | 0 | 0 | 3 | 3 |
| C18ORF21 | 0 | 2 | 0 | 0 | 2 | 2 |
| C18ORF34 | 2 | 0 | 0 | 3 | 5 | 3 |
| C19ORF21 | 1 | 1 | 0 | 0 | 2 | 1 |
| C19ORF47 | 0 | 0 | 0 | 3 | 3 | 3 |
| C19ORF48 | 0 | 1 | 0 | 0 | 1 | 1 |
| C19ORF55 | 0 | 0 | 0 | 3 | 3 | 3 |
| C19ORF57 | 0 | 0 | 0 | 1 | 1 | 1 |
| C19ORF59 | 0 | 4 | 0 | 0 | 4 | 4 |
| C19ORF75 | 1 | 0 | 0 | 1 | 2 | 1 |
| C1ORF105 | 0 | 0 | 0 | 1 | 1 | 1 |
| C1ORF112 | 0 | 1 | 0 | 1 | 2 | 2 |
| C1ORF122 | 0 | 0 | 0 | 1 | 1 | 1 |
| C1ORF127 | 9 | 0 | 0 | 0 | 9 | 0 |
| C1ORF135 | 0 | 0 | 0 | 6 | 6 | 6 |
| C1ORF141 | 0 | 0 | 0 | 5 | 5 | 5 |
| C1ORF158 | 1 | 1 | 0 | 0 | 2 | 1 |
| C1ORF168 | 4 | 6 | 0 | 3 | 13 | 9 |
| C1ORF173 | 0 | 0 | 0 | 1 | 1 | 1 |
| C1ORF177 | 0 | 2 | 0 | 5 | 7 | 7 |
| C1ORF187 | 0 | 0 | 0 | 2 | 2 | 2 |
| C1ORF189 | 0 | 1 | 0 | 6 | 7 | 7 |
| C1ORF210 | 0 | 1 | 0 | 0 | 1 | 1 |
| C1ORF216 | 0 | 1 | 0 | 1 | 2 | 2 |
| C1ORF227 | 0 | 4 | 0 | 0 | 4 | 4 |
| C1ORF50 | 0 | 1 | 0 | 0 | 1 | 1 |
| C1ORF51 | 0 | 0 | 0 | 1 | 1 | 1 |
| C1ORF85 | 1 | 0 | 0 | 0 | 1 | 0 |
| C1ORF87 | 0 | 1 | 0 | 0 | 1 | 1 |
| C1ORF88 | 0 | 0 | 0 | 1 | 1 | 1 |
| C1ORF9 | 0 | 1 | 0 | 0 | 1 | 1 |
| C1QB | 0 | 0 | 0 | 1 | 1 | 1 |
| C1QBP | 1 | 0 | 0 | 0 | 1 | 0 |
| C1QC | 0 | 0 | 0 | 2 | 2 | 2 |
| C1QTNF1 | 0 | 1 | 0 | 0 | 1 | 1 |
| C1QTNF2 | 0 | 1 | 0 | 1 | 2 | 2 |
| C1QTNF3 | 0 | 0 | 0 | 1 | 1 | 1 |
| C1QTNF5 | 0 | 0 | 0 | 2 | 2 | 2 |
| C1QTNF9 | 0 | 1 | 0 | 0 | 1 | 1 |
| C1R | 0 | 0 | 0 | 1 | 1 | 1 |
| C1RL | 0 | 0 | 0 | 1 | 1 | 1 |
| C20ORF106 | 0 | 1 | 0 | 0 | 1 | 1 |
| C20ORF107 | 0 | 0 | 0 | 1 | 1 | 1 |
| C20ORF118 | 0 | 0 | 0 | 1 | 1 | 1 |
| C20ORF132 | 0 | 0 | 0 | 6 | 6 | 6 |
| C20ORF141 | 0 | 0 | 0 | 2 | 2 | 2 |
| C20ORF152 | 0 | 1 | 0 | 0 | 1 | 1 |
| C20ORF26 | 0 | 1 | 0 | 1 | 2 | 2 |
| C20ORF4 | 0 | 0 | 0 | 1 | 1 | 1 |
| C20ORF85 | 0 | 0 | 0 | 1 | 1 | 1 |
| C20ORF94 | 0 | 0 | 0 | 2 | 2 | 2 |
| C20ORF96 | 1 | 0 | 0 | 0 | 1 | 0 |
| C21ORF2 | 0 | 0 | 0 | 1 | 1 | 1 |
| C21ORF33 | 0 | 0 | 0 | 1 | 1 | 1 |
| C21ORF59 | 0 | 1 | 0 | 0 | 1 | 1 |
| C22ORF15 | 0 | 4 | 0 | 0 | 4 | 4 |
| C22ORF23 | 0 | 0 | 0 | 1 | 1 | 1 |
| C22ORF25 | 0 | 1 | 0 | 0 | 1 | 1 |
| C22ORF29 | 0 | 1 | 0 | 1 | 2 | 2 |
| C22ORF31 | 0 | 0 | 0 | 1 | 1 | 1 |
| C22ORF33 | 2 | 1 | 0 | 0 | 3 | 1 |
| C22ORF42 | 1 | 0 | 0 | 5 | 6 | 5 |
| C2CD3 | 1 | 0 | 0 | 1 | 2 | 1 |
| C2ORF3 | 1 | 0 | 0 | 0 | 1 | 0 |
| C2ORF40 | 0 | 3 | 0 | 0 | 3 | 3 |
| C2ORF42 | 0 | 0 | 0 | 3 | 3 | 3 |
| C2ORF44 | 0 | 0 | 0 | 2 | 2 | 2 |
| C2ORF56 | 0 | 1 | 0 | 0 | 1 | 1 |
| C2ORF57 | 0 | 0 | 0 | 3 | 3 | 3 |
| C2ORF63 | 5 | 4 | 0 | 0 | 9 | 4 |
| C2ORF65 | 0 | 0 | 0 | 5 | 5 | 5 |
| C2ORF67 | 0 | 0 | 0 | 1 | 1 | 1 |
| C2ORF68 | 0 | 1 | 0 | 0 | 1 | 1 |
| C2ORF76 | 0 | 0 | 0 | 2 | 2 | 2 |
| C2ORF77 | 0 | 1 | 0 | 0 | 1 | 1 |
| C2ORF78 | 0 | 0 | 0 | 1 | 1 | 1 |
| C2ORF80 | 0 | 1 | 0 | 0 | 1 | 1 |
| C2ORF85 | 0 | 0 | 0 | 1 | 1 | 1 |
| C3ORF20 | 0 | 0 | 0 | 1 | 1 | 1 |
| C3ORF25 | 0 | 18 | 0 | 0 | 18 | 18 |
| C3ORF32 | 0 | 1 | 0 | 1 | 2 | 2 |
| C3ORF33 | 0 | 1 | 0 | 1 | 2 | 2 |
| C3ORF62 | 0 | 1 | 0 | 0 | 1 | 1 |
| C3ORF64 | 0 | 0 | 0 | 1 | 1 | 1 |
| C3ORF67 | 0 | 1 | 0 | 0 | 1 | 1 |
| C3ORF72 | 0 | 0 | 0 | 10 | 10 | 10 |
| C3ORF77 | 0 | 0 | 0 | 1 | 1 | 1 |
| C4BPA | 1 | 0 | 0 | 0 | 1 | 0 |
| C4ORF17 | 1 | 0 | 0 | 27 | 28 | 27 |
| C4ORF21 | 1 | 0 | 0 | 1 | 2 | 1 |
| C4ORF27 | 0 | 0 | 0 | 1 | 1 | 1 |
| C4ORF29 | 0 | 1 | 0 | 0 | 1 | 1 |
| C4ORF32 | 0 | 0 | 0 | 1 | 1 | 1 |
| C4ORF33 | 0 | 4 | 0 | 0 | 4 | 4 |
| C4ORF37 | 0 | 1 | 0 | 2 | 3 | 3 |
| C4ORF39 | 0 | 0 | 0 | 1 | 1 | 1 |
| C4ORF46 | 0 | 0 | 0 | 1 | 1 | 1 |
| C4ORF47 | 0 | 0 | 0 | 3 | 3 | 3 |
| C4ORF51 | 0 | 0 | 0 | 1 | 1 | 1 |
| C4ORF6 | 0 | 0 | 0 | 2 | 2 | 2 |
| C5 | 0 | 0 | 0 | 4 | 4 | 4 |
| C5ORF25 | 0 | 7 | 0 | 0 | 7 | 7 |
| C5ORF28 | 0 | 1 | 0 | 0 | 1 | 1 |
| C5ORF34 | 0 | 1 | 0 | 0 | 1 | 1 |
| C5ORF42 | 0 | 3 | 0 | 0 | 3 | 3 |
| C5ORF45 | 0 | 7 | 0 | 0 | 7 | 7 |
| C5ORF48 | 0 | 0 | 1 | 0 | 1 | 0 |
| C5ORF49 | 0 | 2 | 0 | 0 | 2 | 2 |
| C5ORF54 | 0 | 0 | 0 | 1 | 1 | 1 |
| C5ORF55 | 0 | 0 | 0 | 1 | 1 | 1 |
| C6 | 3 | 2 | 0 | 1 | 6 | 3 |
| C6ORF1 | 0 | 1 | 0 | 0 | 1 | 1 |
| C6ORF10 | 0 | 0 | 0 | 1 | 1 | 1 |
| C6ORF106 | 2 | 0 | 0 | 0 | 2 | 0 |
| C6ORF118 | 0 | 0 | 0 | 1 | 1 | 1 |
| C6ORF125 | 0 | 0 | 0 | 14 | 14 | 14 |
| C6ORF130 | 0 | 1 | 0 | 0 | 1 | 1 |
| C6ORF146 | 0 | 0 | 0 | 1 | 1 | 1 |
| C6ORF165 | 0 | 1 | 0 | 1 | 2 | 2 |
| C6ORF186 | 1 | 1 | 0 | 0 | 2 | 1 |
| C6ORF191 | 0 | 0 | 0 | 1 | 1 | 1 |
| C6ORF203 | 0 | 0 | 0 | 1 | 1 | 1 |
| C6ORF211 | 0 | 0 | 0 | 1 | 1 | 1 |
| C6ORF222 | 0 | 0 | 0 | 2 | 2 | 2 |
| C6ORF26 | 0 | 1 | 0 | 0 | 1 | 1 |
| C6ORF70 | 1 | 0 | 0 | 0 | 1 | 0 |
| C7 | 3 | 0 | 0 | 1 | 4 | 1 |
| C7ORF10 | 0 | 0 | 0 | 2 | 2 | 2 |
| C7ORF29 | 0 | 0 | 0 | 5 | 5 | 5 |
| C7ORF43 | 1 | 0 | 0 | 1 | 2 | 1 |
| C7ORF46 | 0 | 0 | 0 | 3 | 3 | 3 |
| C7ORF49 | 0 | 0 | 0 | 1 | 1 | 1 |
| C7ORF52 | 0 | 0 | 0 | 1 | 1 | 1 |
| C7ORF63 | 0 | 2 | 0 | 1 | 3 | 3 |
| C7ORF68 | 0 | 0 | 0 | 1 | 1 | 1 |
| C7ORF72 | 0 | 0 | 0 | 1 | 1 | 1 |
| C8B | 0 | 11 | 0 | 0 | 11 | 11 |
| C8ORF44 | 0 | 7 | 0 | 4 | 11 | 11 |
| C8ORF44-SGK3 | 6 | 0 | 0 | 0 | 6 | 0 |
| C8ORF74 | 0 | 0 | 0 | 2 | 2 | 2 |
| C8ORF80 | 0 | 0 | 0 | 2 | 2 | 2 |
| C8ORF85 | 0 | 1 | 0 | 0 | 1 | 1 |
| C9 | 0 | 1 | 0 | 1 | 2 | 2 |
| C9ORF102 | 0 | 0 | 0 | 1 | 1 | 1 |
| C9ORF117 | 0 | 0 | 1 | 0 | 1 | 0 |
| C9ORF128 | 0 | 1 | 0 | 0 | 1 | 1 |
| C9ORF129 | 0 | 3 | 0 | 0 | 3 | 3 |
| C9ORF131 | 0 | 0 | 0 | 2 | 2 | 2 |
| C9ORF135 | 0 | 1 | 0 | 0 | 1 | 1 |
| C9ORF152 | 0 | 0 | 0 | 14 | 14 | 14 |
| C9ORF153 | 0 | 1 | 0 | 0 | 1 | 1 |
| C9ORF21 | 1 | 0 | 0 | 0 | 1 | 0 |
| C9ORF25 | 0 | 0 | 0 | 1 | 1 | 1 |
| C9ORF3 | 1 | 0 | 0 | 2 | 3 | 2 |
| C9ORF30-TMEFF1 | 0 | 1 | 0 | 0 | 1 | 1 |
| C9ORF66 | 0 | 1 | 0 | 0 | 1 | 1 |
| C9ORF7 | 0 | 0 | 0 | 1 | 1 | 1 |
| C9ORF71 | 0 | 0 | 1 | 0 | 1 | 0 |
| C9ORF79 | 0 | 0 | 0 | 1 | 1 | 1 |
| C9ORF86 | 0 | 0 | 0 | 5 | 5 | 5 |
| C9ORF91 | 0 | 0 | 0 | 1 | 1 | 1 |
| C9ORF93 | 0 | 0 | 0 | 1 | 1 | 1 |
| CA1 | 1 | 0 | 0 | 0 | 1 | 0 |
| CA11 | 4 | 0 | 0 | 0 | 4 | 0 |
| CA3 | 0 | 0 | 0 | 1 | 1 | 1 |
| CA5A | 0 | 1 | 0 | 0 | 1 | 1 |
| CA5B | 1 | 0 | 0 | 1 | 2 | 1 |
| CAB39L | 0 | 0 | 0 | 1 | 1 | 1 |
| CABIN1 | 0 | 0 | 0 | 3 | 3 | 3 |
| CABYR | 0 | 0 | 0 | 2 | 2 | 2 |
| CACHD1 | 2 | 0 | 0 | 0 | 2 | 0 |
| CACNA1A | 0 | 6 | 0 | 0 | 6 | 6 |
| CACNA1B | 1 | 4 | 0 | 1 | 6 | 5 |
| CACNA1D | 0 | 1 | 0 | 6 | 7 | 7 |
| CACNA1E | 0 | 1 | 0 | 0 | 1 | 1 |
| CACNA1G | 1 | 0 | 0 | 3 | 4 | 3 |
| CACNA1S | 1 | 0 | 0 | 3 | 4 | 3 |
| CACNA2D2 | 0 | 0 | 0 | 1 | 1 | 1 |
| CACNA2D4 | 0 | 2 | 0 | 0 | 2 | 2 |
| CACNB1 | 0 | 0 | 0 | 1 | 1 | 1 |
| CACNG6 | 0 | 0 | 0 | 1 | 1 | 1 |
| CAD | 2 | 0 | 0 | 2 | 4 | 2 |
| CADM4 | 6 | 0 | 0 | 0 | 6 | 0 |
| CADPS | 0 | 3 | 0 | 0 | 3 | 3 |
| CAGE1 | 0 | 1 | 0 | 0 | 1 | 1 |
| CALCR | 0 | 0 | 0 | 1 | 1 | 1 |
| CALHM2 | 0 | 0 | 0 | 4 | 4 | 4 |
| CALM1 | 2 | 0 | 0 | 0 | 2 | 0 |
| CALML4 | 0 | 12 | 0 | 0 | 12 | 12 |
| CALML5 | 0 | 0 | 0 | 2 | 2 | 2 |
| CAMK1 | 0 | 0 | 0 | 1 | 1 | 1 |
| CAMK2A | 1 | 0 | 0 | 1 | 2 | 1 |
| CAMK2B | 0 | 0 | 0 | 1 | 1 | 1 |
| CAMKV | 0 | 0 | 0 | 1 | 1 | 1 |
| CAMLG | 0 | 0 | 1 | 0 | 1 | 0 |
| CAMSAP1 | 1 | 0 | 0 | 0 | 1 | 0 |
| CAMTA2 | 0 | 1 | 0 | 2 | 3 | 3 |
| CANT1 | 0 | 0 | 0 | 2 | 2 | 2 |
| CANX | 0 | 1 | 0 | 0 | 1 | 1 |
| CAPG | 0 | 0 | 0 | 3 | 3 | 3 |
| CAPN11 | 1 | 1 | 0 | 0 | 2 | 1 |
| CAPN14 | 0 | 1 | 0 | 0 | 1 | 1 |
| CAPN2 | 0 | 3 | 0 | 0 | 3 | 3 |
| CAPN5 | 0 | 0 | 0 | 1 | 1 | 1 |
| CAPN6 | 0 | 2 | 0 | 0 | 2 | 2 |
| CAPN7 | 0 | 0 | 0 | 5 | 5 | 5 |
| CAPN9 | 5 | 0 | 0 | 0 | 5 | 0 |
| CAPRIN2 | 0 | 0 | 0 | 1 | 1 | 1 |
| CAPS2 | 1 | 7 | 0 | 0 | 8 | 7 |
| CAPSL | 0 | 0 | 0 | 12 | 12 | 12 |
| CARD10 | 0 | 1 | 0 | 0 | 1 | 1 |
| CARD14 | 0 | 0 | 0 | 1 | 1 | 1 |
| CARD17 | 3 | 0 | 0 | 0 | 3 | 0 |
| CARM1 | 0 | 0 | 0 | 1 | 1 | 1 |
| CARS2 | 0 | 2 | 0 | 3 | 5 | 5 |
| CARTPT | 0 | 0 | 5 | 0 | 5 | 0 |
| CASKIN2 | 0 | 0 | 0 | 2 | 2 | 2 |
| CASP10 | 0 | 1 | 0 | 0 | 1 | 1 |
| CASP14 | 0 | 0 | 1 | 0 | 1 | 0 |
| CASP4 | 0 | 0 | 0 | 2 | 2 | 2 |
| CASP5 | 0 | 0 | 0 | 25 | 25 | 25 |
| CASP8 | 1 | 0 | 0 | 0 | 1 | 0 |
| CASP8AP2 | 0 | 0 | 0 | 1 | 1 | 1 |
| CASP9 | 0 | 7 | 0 | 1 | 8 | 8 |
| CASR | 0 | 0 | 0 | 2 | 2 | 2 |
| CATSPER1 | 1 | 0 | 0 | 0 | 1 | 0 |
| CATSPER2 | 1 | 1 | 0 | 0 | 2 | 1 |
| CATSPERG | 0 | 1 | 0 | 1 | 2 | 2 |
| CAV3 | 0 | 0 | 0 | 1 | 1 | 1 |
| CBFA2T2 | 0 | 2 | 0 | 0 | 2 | 2 |
| CBLC | 0 | 0 | 0 | 7 | 7 | 7 |
| CBX3 | 3 | 0 | 0 | 0 | 3 | 0 |
| CBX4 | 0 | 0 | 0 | 1 | 1 | 1 |
| CBX8 | 0 | 0 | 0 | 2 | 2 | 2 |
| CBY1 | 0 | 0 | 0 | 1 | 1 | 1 |
| CC2D1B | 0 | 0 | 0 | 2 | 2 | 2 |
| CC2D2B | 0 | 1 | 0 | 0 | 1 | 1 |
| CCBL2 | 0 | 0 | 0 | 1 | 1 | 1 |
| CCBP2 | 0 | 0 | 0 | 2 | 2 | 2 |
| CCDC102B | 0 | 1 | 0 | 0 | 1 | 1 |
| CCDC105 | 0 | 0 | 0 | 5 | 5 | 5 |
| CCDC108 | 0 | 1 | 0 | 4 | 5 | 5 |
| CCDC11 | 0 | 1 | 0 | 0 | 1 | 1 |
| CCDC111 | 0 | 0 | 0 | 3 | 3 | 3 |
| CCDC121 | 0 | 0 | 0 | 7 | 7 | 7 |
| CCDC122 | 0 | 1 | 0 | 0 | 1 | 1 |
| CCDC127 | 0 | 1 | 0 | 0 | 1 | 1 |
| CCDC129 | 0 | 0 | 0 | 2 | 2 | 2 |
| CCDC13 | 0 | 0 | 0 | 1 | 1 | 1 |
| CCDC132 | 4 | 0 | 0 | 0 | 4 | 0 |
| CCDC135 | 0 | 0 | 0 | 1 | 1 | 1 |
| CCDC14 | 0 | 1 | 0 | 0 | 1 | 1 |
| CCDC144A | 0 | 1 | 0 | 0 | 1 | 1 |
| CCDC144NL | 1 | 0 | 0 | 0 | 1 | 0 |
| CCDC146 | 0 | 0 | 0 | 1 | 1 | 1 |
| CCDC147 | 1 | 0 | 0 | 0 | 1 | 0 |
| CCDC148 | 2 | 0 | 0 | 1 | 3 | 1 |
| CCDC150 | 0 | 1 | 0 | 15 | 16 | 16 |
| CCDC153 | 0 | 1 | 0 | 0 | 1 | 1 |
| CCDC27 | 0 | 0 | 0 | 2 | 2 | 2 |
| CCDC30 | 0 | 1 | 0 | 0 | 1 | 1 |
| CCDC37 | 0 | 0 | 0 | 2 | 2 | 2 |
| CCDC38 | 0 | 1 | 0 | 0 | 1 | 1 |
| CCDC39 | 0 | 0 | 0 | 4 | 4 | 4 |
| CCDC40 | 0 | 0 | 0 | 2 | 2 | 2 |
| CCDC51 | 0 | 1 | 0 | 0 | 1 | 1 |
| CCDC57 | 0 | 0 | 0 | 1 | 1 | 1 |
| CCDC59 | 0 | 0 | 0 | 1 | 1 | 1 |
| CCDC60 | 0 | 0 | 0 | 1 | 1 | 1 |
| CCDC62 | 0 | 1 | 0 | 0 | 1 | 1 |
| CCDC63 | 0 | 0 | 0 | 1 | 1 | 1 |
| CCDC65 | 18 | 2 | 0 | 2 | 22 | 4 |
| CCDC66 | 0 | 3 | 0 | 8 | 11 | 11 |
| CCDC67 | 0 | 0 | 0 | 1 | 1 | 1 |
| CCDC7 | 0 | 1 | 0 | 18 | 19 | 19 |
| CCDC73 | 0 | 1 | 0 | 0 | 1 | 1 |
| CCDC77 | 0 | 2 | 0 | 2 | 4 | 4 |
| CCDC78 | 0 | 1 | 0 | 0 | 1 | 1 |
| CCDC8 | 0 | 0 | 0 | 1 | 1 | 1 |
| CCDC81 | 0 | 1 | 0 | 0 | 1 | 1 |
| CCDC83 | 1 | 0 | 0 | 0 | 1 | 0 |
| CCDC84 | 1 | 0 | 0 | 0 | 1 | 0 |
| CCDC85A | 0 | 0 | 0 | 2 | 2 | 2 |
| CCDC86 | 0 | 0 | 0 | 1 | 1 | 1 |
| CCDC89 | 0 | 1 | 0 | 1 | 2 | 2 |
| CCDC9 | 0 | 2 | 0 | 0 | 2 | 2 |
| CCDC90A | 0 | 0 | 0 | 1 | 1 | 1 |
| CCDC94 | 1 | 0 | 0 | 0 | 1 | 0 |
| CCDC96 | 0 | 0 | 0 | 1 | 1 | 1 |
| CCDC97 | 0 | 0 | 0 | 1 | 1 | 1 |
| CCDC99 | 0 | 0 | 0 | 1 | 1 | 1 |
| CCHCR1 | 4 | 0 | 0 | 1 | 5 | 1 |
| CCKBR | 0 | 0 | 0 | 2 | 2 | 2 |
| CCL25 | 0 | 0 | 8 | 0 | 8 | 0 |
| CCL26 | 0 | 5 | 0 | 0 | 5 | 5 |
| CCNB1IP1 | 0 | 0 | 0 | 1 | 1 | 1 |
| CCND2 | 0 | 0 | 0 | 1 | 1 | 1 |
| CCNDBP1 | 0 | 0 | 0 | 1 | 1 | 1 |
| CCNF | 0 | 0 | 0 | 1 | 1 | 1 |
| CCNI2 | 0 | 0 | 0 | 1 | 1 | 1 |
| CCR1 | 0 | 0 | 0 | 1 | 1 | 1 |
| CCR8 | 0 | 0 | 0 | 1 | 1 | 1 |
| CCR9 | 0 | 0 | 0 | 1 | 1 | 1 |
| CCRN4L | 0 | 1 | 0 | 0 | 1 | 1 |
| CCT5 | 0 | 3 | 0 | 0 | 3 | 3 |
| CCT6B | 1 | 0 | 2 | 0 | 3 | 0 |
| CCT8L2 | 0 | 5 | 0 | 0 | 5 | 5 |
| CD101 | 0 | 1 | 0 | 0 | 1 | 1 |
| CD109 | 0 | 1 | 0 | 2 | 3 | 3 |
| CD163L1 | 0 | 0 | 0 | 6 | 6 | 6 |
| CD164 | 1 | 0 | 0 | 0 | 1 | 0 |
| CD177 | 1 | 1 | 0 | 3 | 5 | 4 |
| CD180 | 0 | 0 | 0 | 1 | 1 | 1 |
| CD19 | 0 | 0 | 0 | 1 | 1 | 1 |
| CD1A | 0 | 2 | 0 | 0 | 2 | 2 |
| CD1B | 0 | 1 | 0 | 0 | 1 | 1 |
| CD200R1 | 0 | 0 | 0 | 2 | 2 | 2 |
| CD200R1L | 0 | 0 | 0 | 11 | 11 | 11 |
| CD207 | 0 | 2 | 0 | 0 | 2 | 2 |
| CD209 | 0 | 0 | 0 | 1 | 1 | 1 |
| CD22 | 3 | 0 | 0 | 0 | 3 | 0 |
| CD244 | 0 | 2 | 0 | 0 | 2 | 2 |
| CD247 | 0 | 0 | 0 | 1 | 1 | 1 |
| CD27 | 0 | 0 | 0 | 5 | 5 | 5 |
| CD300LB | 0 | 1 | 0 | 0 | 1 | 1 |
| CD300LG | 0 | 0 | 0 | 6 | 6 | 6 |
| CD320 | 0 | 0 | 0 | 1 | 1 | 1 |
| CD33 | 0 | 0 | 0 | 16 | 16 | 16 |
| CD36 | 4 | 10 | 0 | 3 | 17 | 13 |
| CD38 | 0 | 0 | 0 | 1 | 1 | 1 |
| CD3EAP | 0 | 0 | 0 | 1 | 1 | 1 |
| CD5 | 0 | 0 | 0 | 3 | 3 | 3 |
| CD5L | 0 | 3 | 0 | 1 | 4 | 4 |
| CD6 | 0 | 0 | 0 | 1 | 1 | 1 |
| CD63 | 0 | 0 | 0 | 1 | 1 | 1 |
| CD68 | 0 | 0 | 0 | 1 | 1 | 1 |
| CD72 | 0 | 5 | 0 | 2 | 7 | 7 |
| CD79A | 0 | 0 | 0 | 1 | 1 | 1 |
| CD97 | 0 | 0 | 0 | 1 | 1 | 1 |
| CDAN1 | 0 | 0 | 0 | 3 | 3 | 3 |
| CDC20B | 0 | 0 | 0 | 1 | 1 | 1 |
| CDC27 | 0 | 4 | 0 | 9 | 13 | 13 |
| CDC34 | 0 | 1 | 0 | 0 | 1 | 1 |
| CDC42BPB | 0 | 0 | 0 | 5 | 5 | 5 |
| CDC42BPG | 0 | 0 | 0 | 1 | 1 | 1 |
| CDC42EP1 | 0 | 0 | 0 | 1 | 1 | 1 |
| CDCA2 | 0 | 0 | 0 | 1 | 1 | 1 |
| CDCA3 | 0 | 0 | 0 | 2 | 2 | 2 |
| CDCP2 | 0 | 0 | 0 | 21 | 21 | 21 |
| CDH10 | 0 | 1 | 0 | 0 | 1 | 1 |
| CDH2 | 0 | 0 | 0 | 1 | 1 | 1 |
| CDH24 | 0 | 11 | 0 | 0 | 11 | 11 |
| CDH26 | 1 | 1 | 0 | 0 | 2 | 1 |
| CDH3 | 0 | 0 | 0 | 1 | 1 | 1 |
| CDH5 | 8 | 0 | 0 | 0 | 8 | 0 |
| CDH7 | 0 | 0 | 0 | 1 | 1 | 1 |
| CDHR3 | 0 | 0 | 0 | 2 | 2 | 2 |
| CDHR4 | 0 | 0 | 0 | 1 | 1 | 1 |
| CDHR5 | 0 | 0 | 0 | 1 | 1 | 1 |
| CDK10 | 0 | 0 | 0 | 1 | 1 | 1 |
| CDK11A | 0 | 1 | 0 | 2 | 3 | 3 |
| CDK12 | 1 | 0 | 0 | 0 | 1 | 0 |
| CDK13 | 0 | 1 | 0 | 0 | 1 | 1 |
| CDK15 | 1 | 0 | 0 | 0 | 1 | 0 |
| CDK19 | 1 | 0 | 0 | 0 | 1 | 0 |
| CDK2AP2 | 0 | 0 | 0 | 7 | 7 | 7 |
| CDK5RAP1 | 0 | 1 | 0 | 0 | 1 | 1 |
| CDK5RAP2 | 0 | 10 | 0 | 2 | 12 | 12 |
| CDK5RAP3 | 0 | 0 | 0 | 1 | 1 | 1 |
| CDKL2 | 5 | 0 | 0 | 0 | 5 | 0 |
| CDKL3 | 0 | 0 | 0 | 3 | 3 | 3 |
| CDKN2B | 0 | 0 | 0 | 1 | 1 | 1 |
| CDKN3 | 0 | 0 | 0 | 1 | 1 | 1 |
| CDON | 0 | 2 | 0 | 0 | 2 | 2 |
| CDR2 | 0 | 0 | 0 | 1 | 1 | 1 |
| CDRT1 | 0 | 0 | 0 | 2 | 2 | 2 |
| CDRT15 | 2 | 2 | 0 | 0 | 4 | 2 |
| CDRT4 | 0 | 0 | 0 | 1 | 1 | 1 |
| CDX2 | 1 | 0 | 0 | 1 | 2 | 1 |
| CEACAM1 | 0 | 0 | 0 | 3 | 3 | 3 |
| CEACAM19 | 0 | 0 | 0 | 1 | 1 | 1 |
| CEACAM20 | 0 | 1 | 0 | 1 | 2 | 2 |
| CEACAM4 | 1 | 0 | 0 | 0 | 1 | 0 |
| CEACAM7 | 1 | 2 | 0 | 0 | 3 | 2 |
| CECR1 | 1 | 0 | 0 | 0 | 1 | 0 |
| CECR2 | 0 | 4 | 0 | 1 | 5 | 5 |
| CELA1 | 0 | 0 | 0 | 30 | 30 | 30 |
| CELA3B | 0 | 0 | 0 | 2 | 2 | 2 |
| CELF3 | 0 | 0 | 0 | 1 | 1 | 1 |
| CELSR3 | 0 | 0 | 0 | 3 | 3 | 3 |
| CENPBD1 | 0 | 3 | 0 | 0 | 3 | 3 |
| CENPE | 1 | 0 | 0 | 4 | 5 | 4 |
| CENPJ | 0 | 1 | 0 | 0 | 1 | 1 |
| CEP128 | 0 | 0 | 0 | 1 | 1 | 1 |
| CEP135 | 0 | 0 | 0 | 1 | 1 | 1 |
| CEP152 | 0 | 0 | 0 | 1 | 1 | 1 |
| CEP164 | 0 | 0 | 0 | 2 | 2 | 2 |
| CEP192 | 2 | 0 | 0 | 0 | 2 | 0 |
| CEP250 | 0 | 0 | 0 | 4 | 4 | 4 |
| CEP290 | 14 | 2 | 0 | 1 | 17 | 3 |
| CEP44 | 0 | 0 | 0 | 1 | 1 | 1 |
| CEP55 | 0 | 1 | 0 | 0 | 1 | 1 |
| CEP57L1 | 0 | 0 | 0 | 1 | 1 | 1 |
| CEP68 | 0 | 1 | 0 | 1 | 2 | 2 |
| CEP70 | 0 | 1 | 0 | 0 | 1 | 1 |
| CEP78 | 1 | 0 | 0 | 0 | 1 | 0 |
| CEP85 | 0 | 4 | 0 | 0 | 4 | 4 |
| CEP89 | 0 | 2 | 0 | 0 | 2 | 2 |
| CEP95 | 0 | 0 | 0 | 1 | 1 | 1 |
| CER1 | 0 | 1 | 0 | 0 | 1 | 1 |
| CERCAM | 0 | 0 | 0 | 1 | 1 | 1 |
| CERKL | 0 | 1 | 0 | 0 | 1 | 1 |
| CERS5 | 1 | 0 | 0 | 0 | 1 | 0 |
| CES1 | 3 | 0 | 0 | 0 | 3 | 0 |
| CES2 | 0 | 0 | 0 | 1 | 1 | 1 |
| CES3 | 0 | 3 | 0 | 0 | 3 | 3 |
| CES5A | 0 | 7 | 0 | 2 | 9 | 9 |
| CETN3 | 0 | 0 | 0 | 3 | 3 | 3 |
| CFDP1 | 1 | 0 | 0 | 0 | 1 | 0 |
| CFHR1 | 1 | 1 | 0 | 0 | 2 | 1 |
| CFHR2 | 0 | 6 | 0 | 0 | 6 | 6 |
| CFHR3 | 1 | 0 | 0 | 1 | 2 | 1 |
| CFHR4 | 1 | 0 | 0 | 0 | 1 | 0 |
| CFHR5 | 0 | 1 | 0 | 7 | 8 | 8 |
| CFTR | 0 | 1 | 0 | 0 | 1 | 1 |
| CGB5 | 0 | 1 | 0 | 0 | 1 | 1 |
| CGN | 0 | 1 | 0 | 0 | 1 | 1 |
| CHAC2 | 0 | 0 | 0 | 1 | 1 | 1 |
| CHAF1A | 0 | 0 | 0 | 1 | 1 | 1 |
| CHAF1B | 1 | 0 | 0 | 0 | 1 | 0 |
| CHCHD4 | 0 | 1 | 0 | 1 | 2 | 2 |
| CHCHD6 | 0 | 0 | 0 | 1 | 1 | 1 |
| CHD1L | 2 | 1 | 0 | 0 | 3 | 1 |
| CHD2 | 0 | 0 | 0 | 2 | 2 | 2 |
| CHD3 | 0 | 1 | 0 | 1 | 2 | 2 |
| CHD5 | 0 | 0 | 0 | 1 | 1 | 1 |
| CHD6 | 0 | 4 | 0 | 0 | 4 | 4 |
| CHD9 | 0 | 0 | 0 | 2 | 2 | 2 |
| CHEK2 | 0 | 0 | 0 | 2 | 2 | 2 |
| CHERP | 0 | 0 | 0 | 2 | 2 | 2 |
| CHI3L1 | 0 | 2 | 0 | 0 | 2 | 2 |
| CHI3L2 | 0 | 0 | 0 | 1 | 1 | 1 |
| CHIC2 | 0 | 1 | 0 | 0 | 1 | 1 |
| CHIT1 | 1 | 1 | 0 | 2 | 4 | 3 |
| CHL1 | 0 | 0 | 0 | 1 | 1 | 1 |
| CHP2 | 0 | 3 | 0 | 2 | 5 | 5 |
| CHPF | 0 | 1 | 0 | 0 | 1 | 1 |
| CHPF2 | 0 | 0 | 0 | 1 | 1 | 1 |
| CHRFAM7A | 0 | 0 | 0 | 2 | 2 | 2 |
| CHRM1 | 0 | 0 | 0 | 1 | 1 | 1 |
| CHRNA1 | 1 | 0 | 0 | 1 | 2 | 1 |
| CHRNA2 | 0 | 0 | 0 | 1 | 1 | 1 |
| CHRNA4 | 0 | 0 | 0 | 1 | 1 | 1 |
| CHRNA6 | 0 | 0 | 0 | 1 | 1 | 1 |
| CHRNA7 | 0 | 0 | 0 | 3 | 3 | 3 |
| CHRNB3 | 1 | 0 | 0 | 1 | 2 | 1 |
| CHRNG | 1 | 0 | 0 | 0 | 1 | 0 |
| CHST15 | 0 | 0 | 0 | 1 | 1 | 1 |
| CHST5 | 0 | 0 | 0 | 1 | 1 | 1 |
| CHTOP | 0 | 0 | 0 | 1 | 1 | 1 |
| CIAO1 | 1 | 0 | 0 | 1 | 2 | 1 |
| CIB4 | 0 | 1 | 0 | 0 | 1 | 1 |
| CIDEA | 0 | 0 | 0 | 1 | 1 | 1 |
| CIDEB | 2 | 0 | 0 | 0 | 2 | 0 |
| CKAP5 | 0 | 0 | 0 | 1 | 1 | 1 |
| CKB | 0 | 0 | 0 | 3 | 3 | 3 |
| CKM | 0 | 0 | 1 | 0 | 1 | 0 |
| CLASP1 | 0 | 2 | 0 | 1 | 3 | 3 |
| CLCA2 | 0 | 1 | 0 | 3 | 4 | 4 |
| CLCA4 | 0 | 0 | 0 | 2 | 2 | 2 |
| CLCN1 | 1 | 0 | 0 | 0 | 1 | 0 |
| CLCN2 | 0 | 0 | 0 | 1 | 1 | 1 |
| CLCN3 | 0 | 0 | 0 | 4 | 4 | 4 |
| CLCN6 | 0 | 0 | 0 | 1 | 1 | 1 |
| CLCNKA | 0 | 0 | 0 | 3 | 3 | 3 |
| CLDN24 | 0 | 1 | 0 | 0 | 1 | 1 |
| CLDN5 | 0 | 1 | 0 | 0 | 1 | 1 |
| CLEC12B | 0 | 0 | 0 | 3 | 3 | 3 |
| CLEC16A | 0 | 2 | 0 | 0 | 2 | 2 |
| CLEC18B | 0 | 0 | 0 | 1 | 1 | 1 |
| CLEC1A | 0 | 0 | 0 | 1 | 1 | 1 |
| CLEC2D | 0 | 0 | 0 | 1 | 1 | 1 |
| CLEC3A | 0 | 0 | 0 | 1 | 1 | 1 |
| CLEC4F | 0 | 1 | 0 | 1 | 2 | 2 |
| CLEC6A | 0 | 1 | 0 | 0 | 1 | 1 |
| CLIC2 | 0 | 0 | 0 | 1 | 1 | 1 |
| CLLU1 | 0 | 0 | 0 | 8 | 8 | 8 |
| CLNS1A | 0 | 2 | 0 | 0 | 2 | 2 |
| CLP1 | 0 | 0 | 0 | 2 | 2 | 2 |
| CLPTM1 | 0 | 1 | 0 | 0 | 1 | 1 |
| CLRN1 | 0 | 0 | 0 | 1 | 1 | 1 |
| CLSTN2 | 0 | 1 | 0 | 0 | 1 | 1 |
| CLSTN3 | 0 | 0 | 0 | 1 | 1 | 1 |
| CLTB | 1 | 0 | 0 | 0 | 1 | 0 |
| CLTC | 0 | 1 | 0 | 0 | 1 | 1 |
| CLTCL1 | 0 | 0 | 0 | 2 | 2 | 2 |
| CLUAP1 | 0 | 0 | 0 | 7 | 7 | 7 |
| CLVS1 | 0 | 0 | 0 | 1 | 1 | 1 |
| CMA1 | 0 | 2 | 0 | 1 | 3 | 3 |
| CMAS | 0 | 0 | 0 | 13 | 13 | 13 |
| CMIP | 0 | 0 | 0 | 1 | 1 | 1 |
| CMPK2 | 0 | 0 | 0 | 2 | 2 | 2 |
| CMTM2 | 1 | 0 | 0 | 0 | 1 | 0 |
| CMYA5 | 0 | 3 | 0 | 2 | 5 | 5 |
| CNDP1 | 0 | 0 | 0 | 1 | 1 | 1 |
| CNDP2 | 0 | 0 | 0 | 1 | 1 | 1 |
| CNGA1 | 0 | 1 | 0 | 0 | 1 | 1 |
| CNGA3 | 1 | 0 | 0 | 1 | 2 | 1 |
| CNGB3 | 0 | 0 | 0 | 5 | 5 | 5 |
| CNKSR1 | 1 | 1 | 0 | 2 | 4 | 3 |
| CNKSR2 | 1 | 0 | 0 | 0 | 1 | 0 |
| CNKSR3 | 0 | 0 | 0 | 6 | 6 | 6 |
| CNNM2 | 0 | 0 | 0 | 2 | 2 | 2 |
| CNNM3 | 0 | 1 | 0 | 0 | 1 | 1 |
| CNOT3 | 0 | 0 | 0 | 8 | 8 | 8 |
| CNPY2 | 0 | 0 | 0 | 1 | 1 | 1 |
| CNTD2 | 0 | 0 | 0 | 1 | 1 | 1 |
| CNTF | 0 | 2 | 0 | 1 | 3 | 3 |
| CNTLN | 0 | 1 | 0 | 0 | 1 | 1 |
| CNTN1 | 0 | 0 | 0 | 5 | 5 | 5 |
| CNTN2 | 0 | 15 | 0 | 0 | 15 | 15 |
| CNTN3 | 0 | 0 | 0 | 1 | 1 | 1 |
| CNTN6 | 0 | 1 | 0 | 1 | 2 | 2 |
| CNTNAP1 | 9 | 0 | 0 | 1 | 10 | 1 |
| CNTNAP3 | 1 | 0 | 0 | 14 | 15 | 14 |
| CNTNAP3B | 0 | 0 | 0 | 2 | 2 | 2 |
| CNTNAP4 | 0 | 0 | 11 | 0 | 11 | 0 |
| CNTROB | 0 | 0 | 0 | 1 | 1 | 1 |
| COASY | 0 | 2 | 0 | 0 | 2 | 2 |
| COBLL1 | 0 | 1 | 0 | 0 | 1 | 1 |
| COG2 | 0 | 0 | 0 | 1 | 1 | 1 |
| COG4 | 0 | 0 | 0 | 1 | 1 | 1 |
| COG7 | 0 | 0 | 0 | 1 | 1 | 1 |
| COL11A2 | 0 | 6 | 0 | 0 | 6 | 6 |
| COL13A1 | 0 | 0 | 0 | 2 | 2 | 2 |
| COL14A1 | 0 | 0 | 0 | 1 | 1 | 1 |
| COL15A1 | 1 | 0 | 0 | 0 | 1 | 0 |
| COL16A1 | 3 | 0 | 0 | 1 | 4 | 1 |
| COL17A1 | 0 | 0 | 0 | 1 | 1 | 1 |
| COL19A1 | 0 | 0 | 0 | 1 | 1 | 1 |
| COL1A1 | 4 | 0 | 0 | 1 | 5 | 1 |
| COL22A1 | 0 | 0 | 0 | 2 | 2 | 2 |
| COL23A1 | 1 | 0 | 0 | 0 | 1 | 0 |
| COL24A1 | 0 | 2 | 0 | 3 | 5 | 5 |
| COL25A1 | 0 | 1 | 0 | 0 | 1 | 1 |
| COL28A1 | 0 | 3 | 0 | 0 | 3 | 3 |
| COL2A1 | 0 | 0 | 0 | 1 | 1 | 1 |
| COL4A1 | 0 | 1 | 0 | 2 | 3 | 3 |
| COL4A2 | 0 | 0 | 0 | 1 | 1 | 1 |
| COL4A4 | 0 | 0 | 0 | 1 | 1 | 1 |
| COL4A5 | 0 | 1 | 0 | 14 | 15 | 15 |
| COL4A6 | 0 | 0 | 0 | 3 | 3 | 3 |
| COL5A1 | 0 | 0 | 0 | 1 | 1 | 1 |
| COL5A2 | 0 | 0 | 0 | 1 | 1 | 1 |
| COL6A5 | 0 | 11 | 0 | 5 | 16 | 16 |
| COL7A1 | 0 | 2 | 0 | 0 | 2 | 2 |
| COL9A1 | 1 | 0 | 0 | 0 | 1 | 0 |
| COL9A2 | 1 | 2 | 0 | 0 | 3 | 2 |
| COLEC11 | 0 | 0 | 0 | 1 | 1 | 1 |
| COLEC12 | 0 | 0 | 0 | 1 | 1 | 1 |
| COMMD10 | 0 | 1 | 0 | 0 | 1 | 1 |
| COMMD2 | 0 | 1 | 0 | 0 | 1 | 1 |
| COPB1 | 0 | 0 | 0 | 1 | 1 | 1 |
| COPG | 0 | 0 | 0 | 1 | 1 | 1 |
| COPS7A | 0 | 2 | 0 | 0 | 2 | 2 |
| COPZ1 | 0 | 0 | 0 | 1 | 1 | 1 |
| COQ5 | 0 | 1 | 0 | 0 | 1 | 1 |
| COQ6 | 0 | 4 | 0 | 0 | 4 | 4 |
| CORO1C | 0 | 0 | 0 | 1 | 1 | 1 |
| CORO2A | 1 | 0 | 12 | 0 | 13 | 0 |
| CORO2B | 0 | 0 | 0 | 1 | 1 | 1 |
| COX6A1 | 0 | 0 | 0 | 2 | 2 | 2 |
| COX6B2 | 0 | 0 | 0 | 1 | 1 | 1 |
| CPA1 | 0 | 1 | 0 | 0 | 1 | 1 |
| CPA4 | 0 | 2 | 0 | 0 | 2 | 2 |
| CPB2 | 0 | 0 | 0 | 1 | 1 | 1 |
| CPD | 0 | 0 | 0 | 1 | 1 | 1 |
| CPEB3 | 0 | 0 | 0 | 1 | 1 | 1 |
| CPEB4 | 0 | 0 | 0 | 1 | 1 | 1 |
| CPNE1 | 0 | 0 | 0 | 1 | 1 | 1 |
| CPNE3 | 1 | 0 | 0 | 0 | 1 | 0 |
| CPNE7 | 0 | 1 | 0 | 0 | 1 | 1 |
| CPNE9 | 1 | 0 | 0 | 0 | 1 | 0 |
| CPO | 0 | 1 | 0 | 0 | 1 | 1 |
| CPS1 | 0 | 0 | 0 | 1 | 1 | 1 |
| CPSF1 | 0 | 0 | 0 | 5 | 5 | 5 |
| CPT2 | 0 | 0 | 0 | 1 | 1 | 1 |
| CPVL | 0 | 16 | 0 | 1 | 17 | 17 |
| CPXM1 | 0 | 0 | 1 | 1 | 2 | 1 |
| CPXM2 | 0 | 0 | 0 | 2 | 2 | 2 |
| CPZ | 0 | 1 | 0 | 0 | 1 | 1 |
| CR2 | 0 | 1 | 0 | 0 | 1 | 1 |
| CRAT | 0 | 0 | 0 | 1 | 1 | 1 |
| CRB1 | 0 | 0 | 0 | 2 | 2 | 2 |
| CRBN | 1 | 0 | 0 | 0 | 1 | 0 |
| CRCT1 | 0 | 1 | 0 | 0 | 1 | 1 |
| CREB3L1 | 2 | 0 | 0 | 0 | 2 | 0 |
| CREBBP | 0 | 0 | 0 | 3 | 3 | 3 |
| CREG2 | 0 | 1 | 0 | 0 | 1 | 1 |
| CRELD1 | 0 | 0 | 0 | 2 | 2 | 2 |
| CRHR2 | 0 | 1 | 0 | 4 | 5 | 5 |
| CRIPAK | 0 | 0 | 0 | 20 | 20 | 20 |
| CRISP1 | 0 | 1 | 0 | 0 | 1 | 1 |
| CRISPLD1 | 0 | 1 | 0 | 0 | 1 | 1 |
| CRISPLD2 | 1 | 0 | 0 | 0 | 1 | 0 |
| CRLF1 | 0 | 0 | 0 | 1 | 1 | 1 |
| CRNKL1 | 0 | 1 | 0 | 0 | 1 | 1 |
| CROCC | 0 | 1 | 0 | 1 | 2 | 2 |
| CROT | 0 | 0 | 0 | 2 | 2 | 2 |
| CRTC2 | 1 | 0 | 0 | 0 | 1 | 0 |
| CRX | 0 | 0 | 0 | 1 | 1 | 1 |
| CRY1 | 0 | 0 | 0 | 3 | 3 | 3 |
| CRYBA4 | 7 | 1 | 0 | 0 | 8 | 1 |
| CRYBB2 | 1 | 0 | 0 | 0 | 1 | 0 |
| CRYGA | 0 | 3 | 0 | 0 | 3 | 3 |
| CRYGC | 0 | 1 | 0 | 0 | 1 | 1 |
| CS | 0 | 1 | 0 | 0 | 1 | 1 |
| CSAG1 | 0 | 21 | 0 | 0 | 21 | 21 |
| CSDC2 | 0 | 0 | 0 | 1 | 1 | 1 |
| CSE1L | 0 | 0 | 0 | 1 | 1 | 1 |
| CSF1R | 0 | 1 | 0 | 10 | 11 | 11 |
| CSF2RB | 0 | 0 | 0 | 4 | 4 | 4 |
| CSF3 | 0 | 0 | 0 | 1 | 1 | 1 |
| CSGALNACT2 | 0 | 3 | 0 | 0 | 3 | 3 |
| CSH1 | 0 | 0 | 0 | 1 | 1 | 1 |
| CSH2 | 0 | 0 | 2 | 0 | 2 | 0 |
| CSHL1 | 0 | 0 | 0 | 3 | 3 | 3 |
| CSMD1 | 0 | 7 | 10 | 0 | 17 | 7 |
| CSMD2 | 0 | 0 | 0 | 1 | 1 | 1 |
| CSMD3 | 0 | 2 | 0 | 1 | 3 | 3 |
| CSN1S1 | 4 | 0 | 0 | 0 | 4 | 0 |
| CSPG4 | 0 | 0 | 0 | 4 | 4 | 4 |
| CSPP1 | 0 | 0 | 0 | 1 | 1 | 1 |
| CST2 | 0 | 2 | 0 | 0 | 2 | 2 |
| CST3 | 0 | 1 | 0 | 1 | 2 | 2 |
| CST4 | 0 | 0 | 0 | 1 | 1 | 1 |
| CST7 | 0 | 0 | 0 | 1 | 1 | 1 |
| CST8 | 0 | 2 | 0 | 0 | 2 | 2 |
| CST9 | 0 | 0 | 0 | 1 | 1 | 1 |
| CSTL1 | 1 | 0 | 0 | 0 | 1 | 0 |
| CT62 | 0 | 1 | 0 | 0 | 1 | 1 |
| CTAGE9 | 0 | 0 | 0 | 1 | 1 | 1 |
| CTBP2 | 0 | 14 | 0 | 7 | 21 | 21 |
| CTC1 | 1 | 0 | 0 | 0 | 1 | 0 |
| CTCFL | 0 | 0 | 0 | 1 | 1 | 1 |
| CTDSP2 | 0 | 0 | 0 | 11 | 11 | 11 |
| CTF1 | 1 | 0 | 0 | 0 | 1 | 0 |
| CTH | 0 | 0 | 0 | 2 | 2 | 2 |
| CTNNA2 | 0 | 1 | 0 | 0 | 1 | 1 |
| CTNNA3 | 0 | 0 | 0 | 1 | 1 | 1 |
| CTNNAL1 | 0 | 3 | 0 | 0 | 3 | 3 |
| CTNND2 | 15 | 0 | 0 | 0 | 15 | 0 |
| CTPS | 1 | 0 | 0 | 0 | 1 | 0 |
| CTR9 | 0 | 0 | 0 | 1 | 1 | 1 |
| CTSA | 0 | 0 | 0 | 2 | 2 | 2 |
| CTSB | 0 | 2 | 0 | 0 | 2 | 2 |
| CTSC | 0 | 0 | 0 | 1 | 1 | 1 |
| CTSE | 2 | 1 | 0 | 0 | 3 | 1 |
| CTSW | 1 | 0 | 0 | 1 | 2 | 1 |
| CUBN | 0 | 1 | 0 | 1 | 2 | 2 |
| CUL4B | 0 | 0 | 0 | 1 | 1 | 1 |
| CUL7 | 1 | 1 | 0 | 0 | 2 | 1 |
| CUL9 | 0 | 0 | 0 | 3 | 3 | 3 |
| CUTA | 0 | 0 | 1 | 0 | 1 | 0 |
| CUTC | 0 | 0 | 0 | 1 | 1 | 1 |
| CWC22 | 2 | 0 | 0 | 0 | 2 | 0 |
| CWC25 | 1 | 1 | 0 | 5 | 7 | 6 |
| CWH43 | 0 | 0 | 0 | 13 | 13 | 13 |
| CX3CR1 | 0 | 0 | 0 | 1 | 1 | 1 |
| CXADR | 0 | 0 | 0 | 1 | 1 | 1 |
| CXCL5 | 0 | 0 | 0 | 1 | 1 | 1 |
| CXCL6 | 0 | 0 | 0 | 7 | 7 | 7 |
| CXCL9 | 0 | 1 | 0 | 0 | 1 | 1 |
| CXCR2 | 0 | 0 | 0 | 1 | 1 | 1 |
| CXORF22 | 0 | 1 | 0 | 1 | 2 | 2 |
| CXORF23 | 0 | 1 | 0 | 0 | 1 | 1 |
| CXORF40B | 0 | 0 | 0 | 1 | 1 | 1 |
| CXORF56 | 0 | 0 | 0 | 2 | 2 | 2 |
| CXORF58 | 7 | 0 | 0 | 0 | 7 | 0 |
| CYB561D2 | 0 | 2 | 0 | 0 | 2 | 2 |
| CYB5D1 | 1 | 0 | 0 | 0 | 1 | 0 |
| CYB5R1 | 1 | 0 | 0 | 0 | 1 | 0 |
| CYB5R4 | 0 | 1 | 0 | 1 | 2 | 2 |
| CYFIP1 | 0 | 0 | 0 | 2 | 2 | 2 |
| CYFIP2 | 0 | 2 | 0 | 1 | 3 | 3 |
| CYP11B2 | 1 | 0 | 0 | 2 | 3 | 2 |
| CYP19A1 | 0 | 0 | 0 | 17 | 17 | 17 |
| CYP1A1 | 0 | 1 | 0 | 1 | 2 | 2 |
| CYP1B1 | 0 | 0 | 0 | 1 | 1 | 1 |
| CYP24A1 | 0 | 1 | 0 | 1 | 2 | 2 |
| CYP26C1 | 0 | 0 | 0 | 1 | 1 | 1 |
| CYP2A13 | 0 | 3 | 0 | 1 | 4 | 4 |
| CYP2A6 | 0 | 1 | 0 | 0 | 1 | 1 |
| CYP2C18 | 0 | 5 | 0 | 0 | 5 | 5 |
| CYP2C19 | 0 | 1 | 0 | 0 | 1 | 1 |
| CYP2C9 | 1 | 0 | 0 | 2 | 3 | 2 |
| CYP2D6 | 0 | 0 | 0 | 12 | 12 | 12 |
| CYP2E1 | 2 | 0 | 0 | 0 | 2 | 0 |
| CYP2J2 | 0 | 0 | 0 | 1 | 1 | 1 |
| CYP2S1 | 0 | 1 | 0 | 0 | 1 | 1 |
| CYP3A4 | 0 | 0 | 0 | 2 | 2 | 2 |
| CYP3A43 | 0 | 1 | 0 | 0 | 1 | 1 |
| CYP3A5 | 0 | 0 | 0 | 14 | 14 | 14 |
| CYP46A1 | 1 | 0 | 0 | 0 | 1 | 0 |
| CYP4A22 | 0 | 1 | 0 | 1 | 2 | 2 |
| CYP4B1 | 0 | 1 | 0 | 0 | 1 | 1 |
| CYP4F11 | 0 | 0 | 0 | 1 | 1 | 1 |
| CYP4F12 | 2 | 0 | 0 | 0 | 2 | 0 |
| CYP4F3 | 0 | 1 | 0 | 0 | 1 | 1 |
| CYP4F8 | 1 | 0 | 0 | 0 | 1 | 0 |
| CYP7A1 | 0 | 1 | 0 | 0 | 1 | 1 |
| CYP8B1 | 0 | 0 | 0 | 1 | 1 | 1 |
| CYTH1 | 1 | 0 | 0 | 0 | 1 | 0 |
| CYYR1 | 1 | 0 | 0 | 0 | 1 | 0 |
| DAB2 | 0 | 0 | 0 | 2 | 2 | 2 |
| DAB2IP | 0 | 0 | 0 | 2 | 2 | 2 |
| DAGLB | 0 | 0 | 0 | 1 | 1 | 1 |
| DAK | 0 | 0 | 0 | 1 | 1 | 1 |
| DAPL1 | 0 | 5 | 0 | 0 | 5 | 5 |
| DARS2 | 1 | 0 | 0 | 0 | 1 | 0 |
| DAXX | 0 | 0 | 0 | 1 | 1 | 1 |
| DAZAP1 | 0 | 0 | 0 | 1 | 1 | 1 |
| DBF4 | 0 | 0 | 0 | 1 | 1 | 1 |
| DBF4B | 4 | 0 | 0 | 0 | 4 | 0 |
| DBT | 0 | 0 | 0 | 1 | 1 | 1 |
| DCAF11 | 0 | 2 | 0 | 0 | 2 | 2 |
| DCAF15 | 0 | 0 | 0 | 1 | 1 | 1 |
| DCAF4L2 | 0 | 0 | 0 | 22 | 22 | 22 |
| DCAF6 | 0 | 0 | 0 | 1 | 1 | 1 |
| DCAKD | 0 | 0 | 0 | 7 | 7 | 7 |
| DCD | 0 | 0 | 0 | 1 | 1 | 1 |
| DCDC2B | 0 | 0 | 0 | 7 | 7 | 7 |
| DCDC5 | 0 | 1 | 0 | 0 | 1 | 1 |
| DCHS1 | 0 | 0 | 0 | 1 | 1 | 1 |
| DCLRE1A | 0 | 3 | 0 | 0 | 3 | 3 |
| DCP1A | 0 | 0 | 0 | 1 | 1 | 1 |
| DCST2 | 0 | 0 | 0 | 2 | 2 | 2 |
| DCTN1 | 0 | 0 | 0 | 4 | 4 | 4 |
| DCTN6 | 1 | 0 | 0 | 0 | 1 | 0 |
| DCUN1D2 | 0 | 1 | 0 | 0 | 1 | 1 |
| DDB1 | 0 | 0 | 0 | 1 | 1 | 1 |
| DDIT3 | 0 | 0 | 0 | 2 | 2 | 2 |
| DDIT4 | 0 | 0 | 0 | 1 | 1 | 1 |
| DDO | 0 | 1 | 0 | 0 | 1 | 1 |
| DDOST | 0 | 0 | 0 | 2 | 2 | 2 |
| DDTL | 0 | 0 | 0 | 6 | 6 | 6 |
| DDX25 | 1 | 0 | 0 | 0 | 1 | 0 |
| DDX27 | 0 | 1 | 0 | 1 | 2 | 2 |
| DDX39A | 0 | 0 | 0 | 1 | 1 | 1 |
| DDX41 | 0 | 0 | 0 | 1 | 1 | 1 |
| DDX42 | 1 | 0 | 0 | 0 | 1 | 0 |
| DDX49 | 0 | 0 | 0 | 2 | 2 | 2 |
| DDX54 | 0 | 8 | 0 | 0 | 8 | 8 |
| DDX55 | 0 | 0 | 0 | 2 | 2 | 2 |
| DDX58 | 0 | 0 | 0 | 1 | 1 | 1 |
| DDX59 | 0 | 0 | 0 | 1 | 1 | 1 |
| DDX60 | 0 | 1 | 0 | 1 | 2 | 2 |
| DDX60L | 2 | 1 | 0 | 1 | 4 | 2 |
| DEFA6 | 0 | 4 | 0 | 0 | 4 | 4 |
| DEFB108B | 0 | 0 | 0 | 1 | 1 | 1 |
| DEFB126 | 0 | 0 | 0 | 2 | 2 | 2 |
| DEFB128 | 0 | 0 | 0 | 17 | 17 | 17 |
| DEFB132 | 0 | 0 | 2 | 0 | 2 | 0 |
| DEM1 | 0 | 0 | 0 | 17 | 17 | 17 |
| DENND1B | 0 | 10 | 0 | 0 | 10 | 10 |
| DENND1C | 0 | 0 | 0 | 2 | 2 | 2 |
| DENND2D | 0 | 1 | 0 | 0 | 1 | 1 |
| DENND4B | 0 | 0 | 0 | 1 | 1 | 1 |
| DENND4C | 0 | 1 | 0 | 1 | 2 | 2 |
| DEPDC1 | 0 | 0 | 0 | 1 | 1 | 1 |
| DEPDC7 | 0 | 0 | 0 | 1 | 1 | 1 |
| DEPTOR | 0 | 0 | 0 | 1 | 1 | 1 |
| DERL3 | 0 | 0 | 0 | 1 | 1 | 1 |
| DFFA | 0 | 0 | 0 | 1 | 1 | 1 |
| DFNA5 | 0 | 0 | 0 | 6 | 6 | 6 |
| DGAT2 | 0 | 16 | 0 | 0 | 16 | 16 |
| DGAT2L6 | 0 | 1 | 0 | 0 | 1 | 1 |
| DGCR14 | 0 | 1 | 0 | 0 | 1 | 1 |
| DGCR2 | 0 | 17 | 0 | 0 | 17 | 17 |
| DGCR8 | 0 | 0 | 0 | 4 | 4 | 4 |
| DHCR24 | 0 | 1 | 0 | 1 | 2 | 2 |
| DHCR7 | 3 | 1 | 0 | 0 | 4 | 1 |
| DHDH | 0 | 1 | 0 | 0 | 1 | 1 |
| DHRS12 | 0 | 0 | 0 | 1 | 1 | 1 |
| DHRS2 | 0 | 1 | 0 | 1 | 2 | 2 |
| DHRS3 | 0 | 0 | 0 | 2 | 2 | 2 |
| DHRS4L2 | 0 | 0 | 0 | 6 | 6 | 6 |
| DHRS7B | 0 | 0 | 0 | 1 | 1 | 1 |
| DHTKD1 | 1 | 1 | 0 | 0 | 2 | 1 |
| DHX30 | 0 | 1 | 0 | 1 | 2 | 2 |
| DHX32 | 1 | 0 | 0 | 1 | 2 | 1 |
| DHX37 | 0 | 0 | 0 | 2 | 2 | 2 |
| DHX40 | 0 | 1 | 0 | 0 | 1 | 1 |
| DHX58 | 1 | 1 | 0 | 1 | 3 | 2 |
| DIAPH1 | 0 | 0 | 0 | 2 | 2 | 2 |
| DIAPH3 | 0 | 0 | 0 | 1 | 1 | 1 |
| DIDO1 | 0 | 0 | 0 | 3 | 3 | 3 |
| DIMT1 | 2 | 0 | 0 | 18 | 20 | 18 |
| DIP2A | 0 | 0 | 0 | 1 | 1 | 1 |
| DIP2C | 0 | 3 | 0 | 0 | 3 | 3 |
| DIS3 | 0 | 1 | 3 | 0 | 4 | 1 |
| DISP2 | 0 | 0 | 0 | 1 | 1 | 1 |
| DIXDC1 | 1 | 0 | 0 | 0 | 1 | 0 |
| DKK3 | 0 | 1 | 0 | 0 | 1 | 1 |
| DKKL1 | 0 | 0 | 0 | 1 | 1 | 1 |
| DLC1 | 0 | 0 | 0 | 1 | 1 | 1 |
| DLEC1 | 1 | 1 | 0 | 1 | 3 | 2 |
| DLGAP3 | 0 | 0 | 0 | 2 | 2 | 2 |
| DLGAP5 | 0 | 1 | 0 | 0 | 1 | 1 |
| DLL3 | 1 | 0 | 0 | 0 | 1 | 0 |
| DLX3 | 0 | 1 | 0 | 1 | 2 | 2 |
| DMBT1 | 0 | 1 | 0 | 1 | 2 | 2 |
| DMC1 | 0 | 0 | 0 | 1 | 1 | 1 |
| DMD | 1 | 0 | 0 | 2 | 3 | 2 |
| DMGDH | 0 | 4 | 0 | 0 | 4 | 4 |
| DMKN | 0 | 0 | 0 | 1 | 1 | 1 |
| DMRTA2 | 0 | 0 | 1 | 0 | 1 | 0 |
| DMXL1 | 0 | 0 | 0 | 18 | 18 | 18 |
| DNAH1 | 0 | 0 | 0 | 3 | 3 | 3 |
| DNAH10 | 0 | 3 | 0 | 2 | 5 | 5 |
| DNAH11 | 1 | 10 | 2 | 0 | 13 | 10 |
| DNAH14 | 0 | 2 | 0 | 7 | 9 | 9 |
| DNAH17 | 1 | 0 | 0 | 1 | 2 | 1 |
| DNAH2 | 0 | 1 | 0 | 5 | 6 | 6 |
| DNAH3 | 0 | 7 | 0 | 0 | 7 | 7 |
| DNAH5 | 1 | 1 | 0 | 2 | 4 | 3 |
| DNAH6 | 0 | 1 | 0 | 0 | 1 | 1 |
| DNAH7 | 0 | 9 | 0 | 2 | 11 | 11 |
| DNAH8 | 3 | 1 | 0 | 4 | 8 | 5 |
| DNAH9 | 1 | 1 | 0 | 0 | 2 | 1 |
| DNAI1 | 1 | 0 | 0 | 4 | 5 | 4 |
| DNAJB1 | 0 | 0 | 0 | 1 | 1 | 1 |
| DNAJB3 | 0 | 0 | 0 | 1 | 1 | 1 |
| DNAJB4 | 0 | 3 | 0 | 0 | 3 | 3 |
| DNAJB7 | 0 | 0 | 0 | 15 | 15 | 15 |
| DNAJC10 | 0 | 0 | 0 | 2 | 2 | 2 |
| DNAJC11 | 0 | 0 | 0 | 2 | 2 | 2 |
| DNAJC12 | 0 | 0 | 0 | 1 | 1 | 1 |
| DNAJC13 | 0 | 0 | 0 | 3 | 3 | 3 |
| DNAJC14 | 0 | 2 | 0 | 0 | 2 | 2 |
| DNAJC15 | 1 | 0 | 0 | 0 | 1 | 0 |
| DNAJC16 | 0 | 1 | 0 | 0 | 1 | 1 |
| DNAJC18 | 0 | 4 | 0 | 0 | 4 | 4 |
| DNAJC21 | 0 | 0 | 0 | 1 | 1 | 1 |
| DNAJC28 | 0 | 1 | 0 | 0 | 1 | 1 |
| DNAJC5 | 0 | 0 | 0 | 1 | 1 | 1 |
| DNASE2 | 0 | 0 | 0 | 1 | 1 | 1 |
| DNASE2B | 0 | 1 | 0 | 0 | 1 | 1 |
| DNHD1 | 1 | 2 | 0 | 1 | 4 | 3 |
| DNM2 | 0 | 0 | 0 | 1 | 1 | 1 |
| DNMBP | 0 | 0 | 0 | 1 | 1 | 1 |
| DNMT3A | 0 | 0 | 0 | 1 | 1 | 1 |
| DNMT3B | 0 | 0 | 0 | 1 | 1 | 1 |
| DOCK1 | 1 | 0 | 0 | 0 | 1 | 0 |
| DOCK10 | 0 | 1 | 0 | 0 | 1 | 1 |
| DOCK3 | 0 | 0 | 0 | 3 | 3 | 3 |
| DOCK5 | 0 | 0 | 0 | 1 | 1 | 1 |
| DOCK6 | 2 | 0 | 0 | 1 | 3 | 1 |
| DOCK7 | 0 | 1 | 0 | 1 | 2 | 2 |
| DOCK8 | 0 | 4 | 0 | 1 | 5 | 5 |
| DOK1 | 0 | 1 | 0 | 0 | 1 | 1 |
| DOK5 | 0 | 0 | 0 | 1 | 1 | 1 |
| DOLK | 0 | 0 | 0 | 2 | 2 | 2 |
| DOM3Z | 0 | 0 | 0 | 2 | 2 | 2 |
| DONSON | 1 | 0 | 0 | 0 | 1 | 0 |
| DOPEY2 | 0 | 0 | 0 | 11 | 11 | 11 |
| DPEP2 | 0 | 2 | 0 | 0 | 2 | 2 |
| DPP3 | 1 | 0 | 0 | 0 | 1 | 0 |
| DPP6 | 1 | 0 | 0 | 1 | 2 | 1 |
| DPP7 | 0 | 0 | 0 | 3 | 3 | 3 |
| DPP8 | 0 | 0 | 0 | 10 | 10 | 10 |
| DPP9 | 1 | 5 | 0 | 3 | 9 | 8 |
| DPPA2 | 0 | 0 | 0 | 2 | 2 | 2 |
| DPPA3 | 0 | 0 | 0 | 1 | 1 | 1 |
| DPPA4 | 5 | 0 | 0 | 1 | 6 | 1 |
| DPRX | 0 | 1 | 0 | 0 | 1 | 1 |
| DPY19L2 | 0 | 1 | 0 | 0 | 1 | 1 |
| DPY19L4 | 0 | 0 | 0 | 1 | 1 | 1 |
| DPYD | 3 | 0 | 0 | 1 | 4 | 1 |
| DQX1 | 0 | 4 | 0 | 0 | 4 | 4 |
| DRAM2 | 0 | 1 | 0 | 0 | 1 | 1 |
| DRD3 | 1 | 0 | 0 | 0 | 1 | 0 |
| DRD5 | 0 | 2 | 0 | 0 | 2 | 2 |
| DROSHA | 0 | 0 | 0 | 1 | 1 | 1 |
| DSC2 | 0 | 0 | 0 | 7 | 7 | 7 |
| DSCAM | 0 | 0 | 0 | 2 | 2 | 2 |
| DSCAML1 | 0 | 0 | 0 | 20 | 20 | 20 |
| DSCC1 | 1 | 0 | 0 | 0 | 1 | 0 |
| DSCR3 | 0 | 0 | 0 | 2 | 2 | 2 |
| DSCR6 | 0 | 2 | 0 | 0 | 2 | 2 |
| DSEL | 0 | 0 | 0 | 1 | 1 | 1 |
| DSG4 | 0 | 0 | 0 | 1 | 1 | 1 |
| DSN1 | 0 | 1 | 0 | 0 | 1 | 1 |
| DSP | 1 | 0 | 0 | 0 | 1 | 0 |
| DST | 0 | 0 | 0 | 1 | 1 | 1 |
| DTNBP1 | 4 | 1 | 0 | 0 | 5 | 1 |
| DTWD1 | 0 | 1 | 0 | 0 | 1 | 1 |
| DTX3L | 0 | 1 | 0 | 0 | 1 | 1 |
| DUOX1 | 0 | 1 | 0 | 2 | 3 | 3 |
| DUOX2 | 2 | 0 | 0 | 1 | 3 | 1 |
| DUOXA1 | 0 | 0 | 0 | 1 | 1 | 1 |
| DUSP1 | 0 | 0 | 0 | 3 | 3 | 3 |
| DUSP11 | 0 | 0 | 0 | 1 | 1 | 1 |
| DUSP12 | 0 | 0 | 0 | 1 | 1 | 1 |
| DUSP16 | 0 | 0 | 0 | 4 | 4 | 4 |
| DUSP18 | 0 | 1 | 0 | 3 | 4 | 4 |
| DUSP6 | 0 | 2 | 0 | 0 | 2 | 2 |
| DUXA | 0 | 0 | 0 | 4 | 4 | 4 |
| DVL3 | 0 | 1 | 0 | 0 | 1 | 1 |
| DYDC2 | 3 | 0 | 0 | 1 | 4 | 1 |
| DYNC1H1 | 0 | 0 | 0 | 1 | 1 | 1 |
| DYNC1I1 | 0 | 0 | 0 | 1 | 1 | 1 |
| DYNC1LI1 | 0 | 1 | 0 | 0 | 1 | 1 |
| DYNC1LI2 | 0 | 0 | 0 | 2 | 2 | 2 |
| DYNC2H1 | 0 | 16 | 0 | 2 | 18 | 18 |
| DYNC2LI1 | 1 | 0 | 0 | 1 | 2 | 1 |
| DYRK1A | 0 | 2 | 0 | 0 | 2 | 2 |
| DYRK2 | 0 | 0 | 0 | 1 | 1 | 1 |
| DYTN | 0 | 0 | 0 | 2 | 2 | 2 |
| DYX1C1 | 0 | 1 | 0 | 0 | 1 | 1 |
| DZANK1 | 1 | 0 | 0 | 0 | 1 | 0 |
| E2F8 | 0 | 1 | 0 | 0 | 1 | 1 |
| EBF4 | 0 | 0 | 0 | 1 | 1 | 1 |
| EBI3 | 1 | 0 | 0 | 0 | 1 | 0 |
| EBLN2 | 0 | 10 | 0 | 0 | 10 | 10 |
| ECD | 0 | 0 | 0 | 1 | 1 | 1 |
| ECE1 | 0 | 0 | 0 | 1 | 1 | 1 |
| ECEL1 | 0 | 1 | 0 | 0 | 1 | 1 |
| ECHDC2 | 0 | 0 | 0 | 1 | 1 | 1 |
| ECHDC3 | 1 | 1 | 0 | 0 | 2 | 1 |
| ECM2 | 0 | 0 | 0 | 1 | 1 | 1 |
| ECT2 | 0 | 1 | 0 | 3 | 4 | 4 |
| ECT2L | 1 | 5 | 0 | 0 | 6 | 5 |
| EDA | 0 | 0 | 0 | 1 | 1 | 1 |
| EDA2R | 0 | 0 | 0 | 6 | 6 | 6 |
| EDC4 | 0 | 0 | 0 | 2 | 2 | 2 |
| EDDM3B | 0 | 1 | 0 | 0 | 1 | 1 |
| EDEM1 | 1 | 0 | 0 | 0 | 1 | 0 |
| EDN1 | 0 | 0 | 0 | 1 | 1 | 1 |
| EDN3 | 0 | 2 | 0 | 0 | 2 | 2 |
| EDNRB | 0 | 0 | 0 | 1 | 1 | 1 |
| EEF2 | 0 | 0 | 0 | 1 | 1 | 1 |
| EEF2K | 0 | 2 | 0 | 0 | 2 | 2 |
| EFCAB3 | 0 | 2 | 0 | 0 | 2 | 2 |
| EFCAB5 | 3 | 1 | 0 | 0 | 4 | 1 |
| EFCAB6 | 1 | 0 | 0 | 8 | 9 | 8 |
| EFCAB7 | 0 | 1 | 0 | 1 | 2 | 2 |
| EFEMP2 | 0 | 0 | 0 | 2 | 2 | 2 |
| EFHA1 | 0 | 3 | 0 | 1 | 4 | 4 |
| EFHB | 0 | 0 | 0 | 1 | 1 | 1 |
| EFHC1 | 0 | 0 | 0 | 1 | 1 | 1 |
| EFNB1 | 0 | 0 | 0 | 18 | 18 | 18 |
| EFR3A | 0 | 0 | 0 | 2 | 2 | 2 |
| EGF | 0 | 0 | 0 | 5 | 5 | 5 |
| EGFL8 | 0 | 4 | 0 | 0 | 4 | 4 |
| EGFR | 1 | 0 | 0 | 3 | 4 | 3 |
| EGLN2 | 0 | 0 | 0 | 3 | 3 | 3 |
| EHBP1L1 | 0 | 0 | 0 | 1 | 1 | 1 |
| EHD3 | 0 | 0 | 0 | 2 | 2 | 2 |
| EHD4 | 0 | 0 | 0 | 1 | 1 | 1 |
| EIF2A | 0 | 0 | 0 | 4 | 4 | 4 |
| EIF2AK3 | 0 | 0 | 0 | 1 | 1 | 1 |
| EIF2AK4 | 4 | 0 | 0 | 1 | 5 | 1 |
| EIF2B2 | 2 | 0 | 0 | 0 | 2 | 0 |
| EIF2B3 | 0 | 0 | 0 | 2 | 2 | 2 |
| EIF2B5 | 0 | 0 | 11 | 1 | 12 | 1 |
| EIF2D | 1 | 0 | 0 | 0 | 1 | 0 |
| EIF3I | 0 | 8 | 0 | 0 | 8 | 8 |
| EIF3L | 0 | 0 | 0 | 1 | 1 | 1 |
| EIF4E1B | 1 | 0 | 0 | 0 | 1 | 0 |
| ELAC2 | 1 | 0 | 0 | 1 | 2 | 1 |
| ELFN2 | 0 | 0 | 0 | 2 | 2 | 2 |
| ELMO3 | 0 | 1 | 0 | 2 | 3 | 3 |
| ELMOD2 | 0 | 0 | 0 | 1 | 1 | 1 |
| ELN | 0 | 0 | 0 | 1 | 1 | 1 |
| EME1 | 0 | 9 | 0 | 1 | 10 | 10 |
| EMILIN2 | 0 | 1 | 0 | 1 | 2 | 2 |
| EML3 | 0 | 0 | 0 | 1 | 1 | 1 |
| EML5 | 0 | 0 | 0 | 3 | 3 | 3 |
| EML6 | 0 | 4 | 0 | 0 | 4 | 4 |
| EMR1 | 0 | 1 | 0 | 0 | 1 | 1 |
| ENAH | 7 | 0 | 0 | 0 | 7 | 0 |
| ENAM | 0 | 0 | 0 | 1 | 1 | 1 |
| ENC1 | 0 | 0 | 0 | 1 | 1 | 1 |
| ENDOD1 | 0 | 1 | 0 | 0 | 1 | 1 |
| ENDOU | 1 | 0 | 0 | 1 | 2 | 1 |
| ENDOV | 8 | 0 | 0 | 0 | 8 | 0 |
| ENGASE | 0 | 0 | 0 | 7 | 7 | 7 |
| ENO3 | 0 | 0 | 0 | 1 | 1 | 1 |
| ENOSF1 | 0 | 2 | 0 | 1 | 3 | 3 |
| ENPEP | 0 | 8 | 0 | 1 | 9 | 9 |
| ENPP3 | 0 | 1 | 0 | 0 | 1 | 1 |
| ENPP7 | 1 | 0 | 0 | 0 | 1 | 0 |
| ENTHD1 | 0 | 0 | 0 | 4 | 4 | 4 |
| ENTPD2 | 2 | 0 | 0 | 0 | 2 | 0 |
| ENTPD7 | 0 | 0 | 0 | 1 | 1 | 1 |
| EP300 | 2 | 0 | 0 | 0 | 2 | 0 |
| EP400 | 0 | 0 | 0 | 1 | 1 | 1 |
| EPB41L1 | 0 | 4 | 0 | 0 | 4 | 4 |
| EPB41L3 | 0 | 1 | 0 | 1 | 2 | 2 |
| EPB41L5 | 0 | 15 | 0 | 0 | 15 | 15 |
| EPG5 | 0 | 1 | 0 | 2 | 3 | 3 |
| EPHA1 | 1 | 0 | 0 | 0 | 1 | 0 |
| EPHA10 | 1 | 1 | 0 | 0 | 2 | 1 |
| EPHA2 | 0 | 0 | 0 | 1 | 1 | 1 |
| EPHA5 | 0 | 1 | 0 | 0 | 1 | 1 |
| EPHB1 | 0 | 0 | 0 | 20 | 20 | 20 |
| EPHB2 | 0 | 0 | 0 | 1 | 1 | 1 |
| EPHX2 | 0 | 1 | 0 | 0 | 1 | 1 |
| EPHX3 | 0 | 0 | 0 | 2 | 2 | 2 |
| EPN3 | 0 | 0 | 0 | 3 | 3 | 3 |
| EPO | 0 | 0 | 0 | 1 | 1 | 1 |
| EPOR | 1 | 0 | 0 | 0 | 1 | 0 |
| EPRS | 0 | 2 | 0 | 0 | 2 | 2 |
| EPS8L3 | 1 | 0 | 0 | 1 | 2 | 1 |
| EPSTI1 | 0 | 2 | 0 | 0 | 2 | 2 |
| EPT1 | 0 | 0 | 0 | 1 | 1 | 1 |
| EPX | 2 | 14 | 0 | 0 | 16 | 14 |
| ERAP1 | 0 | 0 | 0 | 1 | 1 | 1 |
| ERAP2 | 1 | 0 | 0 | 0 | 1 | 0 |
| ERBB3 | 0 | 1 | 0 | 1 | 2 | 2 |
| ERC1 | 0 | 7 | 0 | 0 | 7 | 7 |
| ERC2 | 0 | 0 | 0 | 1 | 1 | 1 |
| ERCC2 | 0 | 0 | 0 | 1 | 1 | 1 |
| ERCC3 | 0 | 2 | 0 | 0 | 2 | 2 |
| ERG | 1 | 0 | 0 | 0 | 1 | 0 |
| ERICH1 | 0 | 0 | 0 | 2 | 2 | 2 |
| ERLEC1 | 0 | 0 | 0 | 1 | 1 | 1 |
| ERMAP | 0 | 1 | 0 | 0 | 1 | 1 |
| ERMP1 | 1 | 0 | 0 | 3 | 4 | 3 |
| ERN2 | 1 | 0 | 0 | 0 | 1 | 0 |
| ERO1LB | 0 | 0 | 0 | 1 | 1 | 1 |
| ERV3-1 | 0 | 1 | 0 | 1 | 2 | 2 |
| ERVMER34-1 | 0 | 1 | 0 | 0 | 1 | 1 |
| ESF1 | 0 | 0 | 0 | 7 | 7 | 7 |
| ESR2 | 2 | 0 | 0 | 0 | 2 | 0 |
| ESRP2 | 0 | 0 | 0 | 1 | 1 | 1 |
| ESX1 | 0 | 0 | 0 | 2 | 2 | 2 |
| ESYT1 | 1 | 0 | 0 | 1 | 2 | 1 |
| ETNK2 | 0 | 0 | 0 | 1 | 1 | 1 |
| ETV1 | 0 | 0 | 0 | 1 | 1 | 1 |
| EVPL | 0 | 0 | 0 | 1 | 1 | 1 |
| EWSR1 | 0 | 0 | 0 | 1 | 1 | 1 |
| EXO1 | 1 | 1 | 0 | 0 | 2 | 1 |
| EXOC4 | 1 | 0 | 0 | 0 | 1 | 0 |
| EXOC8 | 0 | 1 | 0 | 0 | 1 | 1 |
| EXOG | 1 | 0 | 0 | 2 | 3 | 2 |
| EXOSC9 | 0 | 0 | 0 | 1 | 1 | 1 |
| EXTL3 | 0 | 2 | 0 | 0 | 2 | 2 |
| EYA4 | 0 | 1 | 0 | 0 | 1 | 1 |
| EZH2 | 2 | 3 | 0 | 2 | 7 | 5 |
| F11 | 0 | 1 | 0 | 0 | 1 | 1 |
| F13A1 | 1 | 0 | 0 | 0 | 1 | 0 |
| F2 | 1 | 0 | 0 | 0 | 1 | 0 |
| F2RL1 | 1 | 0 | 0 | 0 | 1 | 0 |
| F5 | 0 | 0 | 0 | 1 | 1 | 1 |
| F7 | 0 | 0 | 0 | 2 | 2 | 2 |
| FAAH | 0 | 0 | 0 | 1 | 1 | 1 |
| FAAH2 | 0 | 2 | 0 | 0 | 2 | 2 |
| FABP2 | 1 | 0 | 0 | 0 | 1 | 0 |
| FABP4 | 1 | 0 | 0 | 0 | 1 | 0 |
| FABP5 | 0 | 0 | 0 | 1 | 1 | 1 |
| FAH | 0 | 0 | 0 | 1 | 1 | 1 |
| FAHD2B | 0 | 1 | 0 | 0 | 1 | 1 |
| FAIM2 | 0 | 0 | 0 | 1 | 1 | 1 |
| FAIM3 | 0 | 0 | 0 | 1 | 1 | 1 |
| FAM101B | 0 | 17 | 0 | 0 | 17 | 17 |
| FAM102B | 0 | 0 | 0 | 1 | 1 | 1 |
| FAM108A1 | 0 | 0 | 0 | 1 | 1 | 1 |
| FAM111B | 0 | 1 | 0 | 2 | 3 | 3 |
| FAM114A1 | 0 | 1 | 0 | 1 | 2 | 2 |
| FAM115C | 0 | 0 | 0 | 7 | 7 | 7 |
| FAM117A | 0 | 0 | 0 | 1 | 1 | 1 |
| FAM118B | 0 | 0 | 0 | 1 | 1 | 1 |
| FAM123B | 0 | 0 | 0 | 1 | 1 | 1 |
| FAM124A | 0 | 1 | 0 | 0 | 1 | 1 |
| FAM124B | 18 | 0 | 0 | 0 | 18 | 0 |
| FAM126A | 0 | 1 | 0 | 0 | 1 | 1 |
| FAM129A | 6 | 0 | 0 | 0 | 6 | 0 |
| FAM129C | 0 | 0 | 0 | 1 | 1 | 1 |
| FAM134A | 1 | 0 | 0 | 0 | 1 | 0 |
| FAM134C | 0 | 0 | 0 | 1 | 1 | 1 |
| FAM13A | 1 | 1 | 0 | 0 | 2 | 1 |
| FAM13C | 0 | 0 | 0 | 2 | 2 | 2 |
| FAM151A | 1 | 3 | 0 | 5 | 9 | 8 |
| FAM154A | 0 | 2 | 0 | 0 | 2 | 2 |
| FAM154B | 0 | 0 | 0 | 7 | 7 | 7 |
| FAM155A | 0 | 1 | 0 | 0 | 1 | 1 |
| FAM159A | 0 | 2 | 0 | 2 | 4 | 4 |
| FAM160A2 | 0 | 1 | 0 | 2 | 3 | 3 |
| FAM160B1 | 0 | 0 | 0 | 1 | 1 | 1 |
| FAM160B2 | 0 | 0 | 0 | 4 | 4 | 4 |
| FAM161A | 0 | 1 | 0 | 0 | 1 | 1 |
| FAM161B | 0 | 0 | 0 | 2 | 2 | 2 |
| FAM162A | 1 | 0 | 0 | 0 | 1 | 0 |
| FAM164C | 0 | 0 | 0 | 1 | 1 | 1 |
| FAM166A | 0 | 1 | 0 | 1 | 2 | 2 |
| FAM168B | 0 | 1 | 0 | 0 | 1 | 1 |
| FAM171A1 | 0 | 0 | 0 | 1 | 1 | 1 |
| FAM173B | 0 | 1 | 0 | 1 | 2 | 2 |
| FAM174A | 0 | 8 | 0 | 0 | 8 | 8 |
| FAM175B | 0 | 0 | 0 | 1 | 1 | 1 |
| FAM177B | 0 | 1 | 0 | 0 | 1 | 1 |
| FAM178A | 0 | 2 | 0 | 0 | 2 | 2 |
| FAM179A | 1 | 2 | 0 | 1 | 4 | 3 |
| FAM179B | 0 | 9 | 0 | 0 | 9 | 9 |
| FAM180A | 0 | 0 | 0 | 1 | 1 | 1 |
| FAM183A | 0 | 2 | 0 | 0 | 2 | 2 |
| FAM187B | 0 | 17 | 0 | 0 | 17 | 17 |
| FAM188B | 0 | 1 | 0 | 0 | 1 | 1 |
| FAM194A | 0 | 0 | 0 | 3 | 3 | 3 |
| FAM194B | 0 | 0 | 0 | 13 | 13 | 13 |
| FAM205A | 0 | 0 | 0 | 1 | 1 | 1 |
| FAM207A | 0 | 0 | 0 | 1 | 1 | 1 |
| FAM208A | 0 | 0 | 0 | 2 | 2 | 2 |
| FAM208B | 0 | 2 | 0 | 0 | 2 | 2 |
| FAM21C | 0 | 0 | 0 | 1 | 1 | 1 |
| FAM3B | 0 | 0 | 0 | 1 | 1 | 1 |
| FAM3C | 0 | 1 | 0 | 0 | 1 | 1 |
| FAM40B | 0 | 0 | 0 | 1 | 1 | 1 |
| FAM47A | 0 | 0 | 0 | 1 | 1 | 1 |
| FAM47C | 0 | 1 | 0 | 0 | 1 | 1 |
| FAM47E | 1 | 0 | 0 | 0 | 1 | 0 |
| FAM48B1 | 0 | 0 | 0 | 1 | 1 | 1 |
| FAM48B2 | 0 | 0 | 0 | 1 | 1 | 1 |
| FAM58A | 0 | 1 | 0 | 0 | 1 | 1 |
| FAM58BP | 0 | 1 | 0 | 0 | 1 | 1 |
| FAM59A | 0 | 0 | 0 | 10 | 10 | 10 |
| FAM5C | 0 | 0 | 0 | 1 | 1 | 1 |
| FAM63A | 0 | 0 | 0 | 1 | 1 | 1 |
| FAM63B | 0 | 0 | 0 | 3 | 3 | 3 |
| FAM65B | 0 | 0 | 0 | 1 | 1 | 1 |
| FAM70B | 0 | 1 | 0 | 0 | 1 | 1 |
| FAM71A | 0 | 6 | 0 | 0 | 6 | 6 |
| FAM71C | 0 | 0 | 0 | 1 | 1 | 1 |
| FAM71D | 0 | 0 | 0 | 15 | 15 | 15 |
| FAM71F2 | 0 | 1 | 0 | 0 | 1 | 1 |
| FAM73A | 1 | 1 | 0 | 0 | 2 | 1 |
| FAM75A3 | 0 | 0 | 0 | 2 | 2 | 2 |
| FAM75C1 | 0 | 0 | 0 | 1 | 1 | 1 |
| FAM75C2 | 1 | 1 | 0 | 0 | 2 | 1 |
| FAM75D1 | 0 | 0 | 0 | 1 | 1 | 1 |
| FAM78A | 0 | 0 | 0 | 1 | 1 | 1 |
| FAM81B | 0 | 2 | 0 | 4 | 6 | 6 |
| FAM82A1 | 3 | 3 | 0 | 0 | 6 | 3 |
| FAM82A2 | 1 | 0 | 0 | 0 | 1 | 0 |
| FAM83A | 0 | 3 | 0 | 1 | 4 | 4 |
| FAM83D | 0 | 0 | 0 | 1 | 1 | 1 |
| FAM83E | 0 | 0 | 0 | 2 | 2 | 2 |
| FAM83H | 0 | 1 | 0 | 0 | 1 | 1 |
| FAM86B1 | 0 | 0 | 0 | 1 | 1 | 1 |
| FAM86B2 | 0 | 0 | 0 | 1 | 1 | 1 |
| FAM90A1 | 0 | 0 | 0 | 3 | 3 | 3 |
| FAM98C | 0 | 0 | 0 | 1 | 1 | 1 |
| FAM9C | 0 | 2 | 0 | 0 | 2 | 2 |
| FAN1 | 0 | 1 | 0 | 0 | 1 | 1 |
| FANCA | 2 | 0 | 0 | 0 | 2 | 0 |
| FANCD2 | 1 | 2 | 0 | 22 | 25 | 24 |
| FANCE | 0 | 0 | 0 | 1 | 1 | 1 |
| FANCI | 0 | 0 | 0 | 1 | 1 | 1 |
| FANCL | 0 | 0 | 0 | 4 | 4 | 4 |
| FANCM | 0 | 1 | 0 | 0 | 1 | 1 |
| FARP2 | 0 | 1 | 0 | 1 | 2 | 2 |
| FARSB | 0 | 1 | 0 | 0 | 1 | 1 |
| FASLG | 0 | 0 | 0 | 2 | 2 | 2 |
| FASTKD1 | 0 | 5 | 0 | 9 | 14 | 14 |
| FASTKD3 | 0 | 0 | 0 | 1 | 1 | 1 |
| FAT3 | 0 | 0 | 0 | 1 | 1 | 1 |
| FAT4 | 0 | 0 | 0 | 1 | 1 | 1 |
| FBF1 | 0 | 0 | 0 | 1 | 1 | 1 |
| FBLIM1 | 0 | 0 | 0 | 1 | 1 | 1 |
| FBLN1 | 0 | 0 | 0 | 1 | 1 | 1 |
| FBLN2 | 0 | 1 | 2 | 7 | 10 | 8 |
| FBLN7 | 0 | 0 | 0 | 1 | 1 | 1 |
| FBN1 | 0 | 3 | 0 | 0 | 3 | 3 |
| FBN2 | 3 | 0 | 0 | 0 | 3 | 0 |
| FBP2 | 0 | 0 | 5 | 0 | 5 | 0 |
| FBXL13 | 0 | 0 | 0 | 1 | 1 | 1 |
| FBXL21 | 0 | 0 | 0 | 13 | 13 | 13 |
| FBXO10 | 0 | 0 | 0 | 2 | 2 | 2 |
| FBXO22 | 0 | 0 | 1 | 0 | 1 | 0 |
| FBXO24 | 0 | 1 | 0 | 0 | 1 | 1 |
| FBXO28 | 0 | 1 | 0 | 0 | 1 | 1 |
| FBXO3 | 0 | 1 | 0 | 0 | 1 | 1 |
| FBXO38 | 0 | 0 | 0 | 1 | 1 | 1 |
| FBXW10 | 0 | 0 | 0 | 3 | 3 | 3 |
| FBXW4 | 0 | 2 | 0 | 0 | 2 | 2 |
| FBXW8 | 0 | 0 | 0 | 2 | 2 | 2 |
| FCER2 | 2 | 0 | 0 | 1 | 3 | 1 |
| FCGBP | 1 | 1 | 0 | 2 | 4 | 3 |
| FCGR1A | 0 | 4 | 0 | 0 | 4 | 4 |
| FCGR1B | 0 | 0 | 0 | 3 | 3 | 3 |
| FCGR2C | 0 | 0 | 1 | 0 | 1 | 0 |
| FCGR3A | 0 | 0 | 0 | 1 | 1 | 1 |
| FCHSD1 | 0 | 1 | 0 | 1 | 2 | 2 |
| FCHSD2 | 0 | 0 | 12 | 4 | 16 | 4 |
| FCN2 | 0 | 0 | 0 | 2 | 2 | 2 |
| FCN3 | 2 | 0 | 0 | 8 | 10 | 8 |
| FCRL1 | 0 | 1 | 0 | 0 | 1 | 1 |
| FCRL2 | 0 | 0 | 0 | 1 | 1 | 1 |
| FCRL3 | 0 | 2 | 0 | 0 | 2 | 2 |
| FCRL5 | 0 | 0 | 0 | 1 | 1 | 1 |
| FDFT1 | 0 | 1 | 0 | 0 | 1 | 1 |
| FECH | 1 | 0 | 0 | 1 | 2 | 1 |
| FER1L5 | 1 | 0 | 0 | 0 | 1 | 0 |
| FERMT1 | 0 | 0 | 0 | 18 | 18 | 18 |
| FETUB | 0 | 0 | 0 | 3 | 3 | 3 |
| FGD1 | 0 | 0 | 0 | 3 | 3 | 3 |
| FGD6 | 0 | 5 | 0 | 2 | 7 | 7 |
| FGF19 | 0 | 3 | 0 | 0 | 3 | 3 |
| FGF6 | 0 | 0 | 0 | 1 | 1 | 1 |
| FGGY | 3 | 0 | 0 | 1 | 4 | 1 |
| FGL1 | 1 | 0 | 0 | 0 | 1 | 0 |
| FH | 0 | 0 | 0 | 2 | 2 | 2 |
| FHDC1 | 0 | 0 | 0 | 1 | 1 | 1 |
| FHOD1 | 0 | 0 | 0 | 1 | 1 | 1 |
| FIG4 | 0 | 11 | 0 | 0 | 11 | 11 |
| FIGNL1 | 0 | 0 | 0 | 2 | 2 | 2 |
| FKBP14 | 0 | 0 | 1 | 1 | 2 | 1 |
| FKBP15 | 0 | 0 | 0 | 1 | 1 | 1 |
| FKBP2 | 0 | 0 | 0 | 1 | 1 | 1 |
| FKTN | 0 | 4 | 0 | 1 | 5 | 5 |
| FLG | 0 | 15 | 0 | 9 | 24 | 24 |
| FLG2 | 0 | 1 | 0 | 6 | 7 | 7 |
| FLI1 | 0 | 0 | 0 | 1 | 1 | 1 |
| FLII | 0 | 1 | 0 | 1 | 2 | 2 |
| FLNA | 3 | 0 | 0 | 3 | 6 | 3 |
| FLNB | 0 | 0 | 0 | 2 | 2 | 2 |
| FLNC | 0 | 0 | 0 | 2 | 2 | 2 |
| FLOT2 | 0 | 0 | 0 | 2 | 2 | 2 |
| FLRT2 | 0 | 0 | 0 | 2 | 2 | 2 |
| FLT3 | 1 | 0 | 0 | 0 | 1 | 0 |
| FLT3LG | 0 | 0 | 0 | 1 | 1 | 1 |
| FMNL2 | 0 | 7 | 0 | 0 | 7 | 7 |
| FMO2 | 0 | 1 | 4 | 0 | 5 | 1 |
| FMO4 | 0 | 0 | 0 | 2 | 2 | 2 |
| FMO5 | 0 | 1 | 0 | 0 | 1 | 1 |
| FNDC3B | 0 | 0 | 0 | 2 | 2 | 2 |
| FNDC4 | 0 | 0 | 0 | 1 | 1 | 1 |
| FNDC7 | 0 | 2 | 0 | 0 | 2 | 2 |
| FNDC9 | 0 | 0 | 0 | 1 | 1 | 1 |
| FOLR1 | 4 | 0 | 0 | 0 | 4 | 0 |
| FOLR3 | 0 | 2 | 0 | 0 | 2 | 2 |
| FOLR4 | 1 | 0 | 0 | 0 | 1 | 0 |
| FOXB2 | 0 | 0 | 0 | 1 | 1 | 1 |
| FOXC2 | 0 | 0 | 0 | 1 | 1 | 1 |
| FOXD4 | 0 | 0 | 0 | 4 | 4 | 4 |
| FOXD4L5 | 0 | 0 | 0 | 1 | 1 | 1 |
| FOXRED2 | 0 | 0 | 0 | 2 | 2 | 2 |
| FRA10AC1 | 1 | 0 | 0 | 0 | 1 | 0 |
| FRAS1 | 3 | 1 | 0 | 0 | 4 | 1 |
| FREM1 | 0 | 0 | 0 | 5 | 5 | 5 |
| FRG2B | 0 | 0 | 0 | 3 | 3 | 3 |
| FRG2C | 0 | 1 | 0 | 9 | 10 | 10 |
| FRMD4A | 0 | 1 | 0 | 1 | 2 | 2 |
| FRMPD2 | 1 | 0 | 0 | 9 | 10 | 9 |
| FRMPD4 | 0 | 8 | 0 | 0 | 8 | 8 |
| FRS3 | 0 | 0 | 0 | 2 | 2 | 2 |
| FRY | 0 | 0 | 0 | 4 | 4 | 4 |
| FSCB | 0 | 2 | 0 | 0 | 2 | 2 |
| FSCN3 | 1 | 0 | 0 | 0 | 1 | 0 |
| FSD2 | 0 | 1 | 0 | 0 | 1 | 1 |
| FSIP1 | 0 | 1 | 0 | 0 | 1 | 1 |
| FST | 0 | 0 | 0 | 1 | 1 | 1 |
| FSTL4 | 0 | 0 | 0 | 1 | 1 | 1 |
| FTHL17 | 0 | 2 | 0 | 0 | 2 | 2 |
| FTSJD2 | 0 | 3 | 0 | 0 | 3 | 3 |
| FUBP1 | 5 | 0 | 0 | 0 | 5 | 0 |
| FUCA1 | 1 | 0 | 0 | 0 | 1 | 0 |
| FUS | 0 | 0 | 0 | 2 | 2 | 2 |
| FUT10 | 0 | 0 | 0 | 1 | 1 | 1 |
| FUT6 | 0 | 1 | 0 | 0 | 1 | 1 |
| FXC1 | 0 | 0 | 1 | 0 | 1 | 0 |
| FXYD4 | 2 | 0 | 0 | 0 | 2 | 0 |
| FYCO1 | 0 | 0 | 0 | 4 | 4 | 4 |
| FZD1 | 0 | 0 | 0 | 3 | 3 | 3 |
| FZD4 | 0 | 0 | 0 | 1 | 1 | 1 |
| G0S2 | 0 | 0 | 1 | 0 | 1 | 0 |
| G3BP1 | 0 | 3 | 0 | 1 | 4 | 4 |
| GAB2 | 0 | 0 | 0 | 1 | 1 | 1 |
| GAB4 | 1 | 0 | 0 | 0 | 1 | 0 |
| GABBR1 | 0 | 0 | 0 | 1 | 1 | 1 |
| GABPA | 1 | 0 | 0 | 0 | 1 | 0 |
| GABRB1 | 0 | 1 | 0 | 0 | 1 | 1 |
| GABRB3 | 0 | 1 | 0 | 0 | 1 | 1 |
| GAD1 | 0 | 12 | 0 | 0 | 12 | 12 |
| GAK | 0 | 0 | 0 | 1 | 1 | 1 |
| GAL3ST2 | 3 | 0 | 0 | 0 | 3 | 0 |
| GAL3ST3 | 0 | 1 | 0 | 0 | 1 | 1 |
| GAL3ST4 | 0 | 1 | 0 | 0 | 1 | 1 |
| GALC | 0 | 1 | 0 | 0 | 1 | 1 |
| GALNT1 | 0 | 0 | 0 | 1 | 1 | 1 |
| GALNT14 | 0 | 1 | 0 | 1 | 2 | 2 |
| GALNT2 | 0 | 0 | 0 | 1 | 1 | 1 |
| GALNT3 | 0 | 0 | 0 | 1 | 1 | 1 |
| GALNT6 | 0 | 0 | 0 | 1 | 1 | 1 |
| GALNTL5 | 3 | 0 | 0 | 0 | 3 | 0 |
| GALT | 0 | 0 | 0 | 1 | 1 | 1 |
| GANC | 1 | 2 | 0 | 1 | 4 | 3 |
| GAPVD1 | 0 | 0 | 0 | 2 | 2 | 2 |
| GARNL3 | 0 | 0 | 0 | 4 | 4 | 4 |
| GART | 0 | 0 | 0 | 8 | 8 | 8 |
| GAS2 | 0 | 0 | 0 | 2 | 2 | 2 |
| GAS2L2 | 0 | 1 | 0 | 0 | 1 | 1 |
| GATM | 0 | 1 | 0 | 0 | 1 | 1 |
| GBA2 | 0 | 1 | 0 | 0 | 1 | 1 |
| GBAS | 1 | 0 | 0 | 0 | 1 | 0 |
| GBE1 | 1 | 0 | 0 | 0 | 1 | 0 |
| GBF1 | 0 | 0 | 0 | 2 | 2 | 2 |
| GBGT1 | 0 | 13 | 0 | 1 | 14 | 14 |
| GBP3 | 1 | 3 | 0 | 0 | 4 | 3 |
| GBP4 | 6 | 1 | 0 | 0 | 7 | 1 |
| GBP5 | 1 | 3 | 0 | 2 | 6 | 5 |
| GBP7 | 2 | 1 | 0 | 2 | 5 | 3 |
| GCA | 0 | 1 | 0 | 0 | 1 | 1 |
| GCC2 | 0 | 16 | 0 | 0 | 16 | 16 |
| GCKR | 0 | 1 | 0 | 3 | 4 | 4 |
| GCNT1 | 0 | 11 | 0 | 0 | 11 | 11 |
| GCNT3 | 0 | 3 | 0 | 0 | 3 | 3 |
| GCNT4 | 0 | 1 | 0 | 0 | 1 | 1 |
| GCNT7 | 0 | 0 | 5 | 0 | 5 | 0 |
| GDF3 | 0 | 0 | 0 | 2 | 2 | 2 |
| GDF9 | 0 | 1 | 0 | 0 | 1 | 1 |
| GDPD3 | 0 | 0 | 0 | 4 | 4 | 4 |
| GDPD4 | 0 | 0 | 0 | 5 | 5 | 5 |
| GEMIN4 | 0 | 1 | 0 | 0 | 1 | 1 |
| GEMIN5 | 0 | 0 | 0 | 2 | 2 | 2 |
| GFI1 | 7 | 0 | 0 | 0 | 7 | 0 |
| GFM1 | 0 | 0 | 0 | 2 | 2 | 2 |
| GFM2 | 0 | 0 | 0 | 1 | 1 | 1 |
| GGCX | 0 | 1 | 0 | 0 | 1 | 1 |
| GGN | 0 | 8 | 0 | 0 | 8 | 8 |
| GGT1 | 8 | 1 | 0 | 0 | 9 | 1 |
| GGT5 | 0 | 0 | 0 | 1 | 1 | 1 |
| GH2 | 0 | 3 | 0 | 1 | 4 | 4 |
| GHDC | 0 | 0 | 1 | 0 | 1 | 0 |
| GHITM | 0 | 1 | 0 | 0 | 1 | 1 |
| GHR | 0 | 0 | 0 | 1 | 1 | 1 |
| GHRHR | 0 | 6 | 0 | 0 | 6 | 6 |
| GHRL | 0 | 0 | 0 | 1 | 1 | 1 |
| GIMAP2 | 0 | 1 | 0 | 0 | 1 | 1 |
| GIMAP6 | 1 | 0 | 0 | 0 | 1 | 0 |
| GIMAP7 | 0 | 0 | 0 | 2 | 2 | 2 |
| GIMAP8 | 0 | 0 | 0 | 2 | 2 | 2 |
| GINS3 | 0 | 0 | 0 | 3 | 3 | 3 |
| GJA1 | 0 | 0 | 0 | 6 | 6 | 6 |
| GJA10 | 0 | 4 | 0 | 0 | 4 | 4 |
| GJA3 | 0 | 0 | 0 | 2 | 2 | 2 |
| GJA4 | 0 | 3 | 0 | 0 | 3 | 3 |
| GJA8 | 0 | 0 | 0 | 2 | 2 | 2 |
| GJA9 | 0 | 0 | 1 | 1 | 2 | 1 |
| GJB2 | 0 | 1 | 0 | 4 | 5 | 5 |
| GJB4 | 0 | 3 | 0 | 2 | 5 | 5 |
| GJB7 | 0 | 0 | 0 | 3 | 3 | 3 |
| GJD4 | 0 | 0 | 0 | 1 | 1 | 1 |
| GKN2 | 1 | 0 | 0 | 0 | 1 | 0 |
| GLB1L | 0 | 1 | 0 | 0 | 1 | 1 |
| GLB1L2 | 1 | 1 | 0 | 0 | 2 | 1 |
| GLCE | 0 | 0 | 0 | 1 | 1 | 1 |
| GLDC | 0 | 1 | 0 | 1 | 2 | 2 |
| GLG1 | 0 | 0 | 0 | 1 | 1 | 1 |
| GLI1 | 0 | 0 | 0 | 3 | 3 | 3 |
| GLIPR1 | 0 | 1 | 0 | 1 | 2 | 2 |
| GLIPR1L2 | 0 | 0 | 0 | 1 | 1 | 1 |
| GLO1 | 0 | 0 | 0 | 1 | 1 | 1 |
| GLRA3 | 0 | 0 | 0 | 10 | 10 | 10 |
| GLRA4 | 0 | 1 | 0 | 0 | 1 | 1 |
| GLS2 | 2 | 0 | 0 | 0 | 2 | 0 |
| GLT1D1 | 0 | 3 | 0 | 0 | 3 | 3 |
| GLT8D1 | 0 | 0 | 0 | 1 | 1 | 1 |
| GLT8D2 | 0 | 0 | 0 | 1 | 1 | 1 |
| GLTSCR2 | 0 | 0 | 0 | 1 | 1 | 1 |
| GLUD2 | 0 | 1 | 0 | 1 | 2 | 2 |
| GLYR1 | 0 | 0 | 0 | 17 | 17 | 17 |
| GMEB2 | 0 | 0 | 0 | 4 | 4 | 4 |
| GML | 1 | 0 | 0 | 0 | 1 | 0 |
| GMPR | 0 | 0 | 0 | 5 | 5 | 5 |
| GMPR2 | 0 | 0 | 0 | 1 | 1 | 1 |
| GNAI3 | 0 | 3 | 0 | 3 | 6 | 6 |
| GNAL | 0 | 0 | 0 | 3 | 3 | 3 |
| GNAT3 | 0 | 0 | 0 | 1 | 1 | 1 |
| GNB1 | 0 | 2 | 0 | 0 | 2 | 2 |
| GNB2 | 0 | 0 | 0 | 2 | 2 | 2 |
| GNB5 | 0 | 1 | 0 | 0 | 1 | 1 |
| GNL1 | 0 | 2 | 0 | 1 | 3 | 3 |
| GNLY | 2 | 3 | 0 | 2 | 7 | 5 |
| GNPTAB | 0 | 0 | 0 | 2 | 2 | 2 |
| GNPTG | 0 | 0 | 0 | 1 | 1 | 1 |
| GNRH2 | 0 | 0 | 0 | 3 | 3 | 3 |
| GNS | 1 | 0 | 0 | 0 | 1 | 0 |
| GOLGA1 | 0 | 0 | 0 | 2 | 2 | 2 |
| GOLGA3 | 0 | 0 | 0 | 3 | 3 | 3 |
| GOLGA6A | 0 | 1 | 0 | 0 | 1 | 1 |
| GOLGA6B | 0 | 0 | 0 | 2 | 2 | 2 |
| GOLGA6C | 0 | 5 | 0 | 0 | 5 | 5 |
| GOLGA8B | 0 | 1 | 0 | 0 | 1 | 1 |
| GOLGB1 | 0 | 2 | 0 | 0 | 2 | 2 |
| GOLPH3L | 0 | 0 | 0 | 1 | 1 | 1 |
| GON4L | 0 | 0 | 0 | 1 | 1 | 1 |
| GOT1L1 | 0 | 0 | 0 | 21 | 21 | 21 |
| GP1BA | 0 | 1 | 0 | 0 | 1 | 1 |
| GP2 | 1 | 0 | 0 | 0 | 1 | 0 |
| GP6 | 0 | 0 | 0 | 12 | 12 | 12 |
| GPATCH3 | 0 | 2 | 0 | 0 | 2 | 2 |
| GPC4 | 0 | 0 | 0 | 1 | 1 | 1 |
| GPC6 | 0 | 4 | 0 | 0 | 4 | 4 |
| GPHN | 0 | 0 | 0 | 7 | 7 | 7 |
| GPLD1 | 0 | 0 | 0 | 5 | 5 | 5 |
| GPN3 | 0 | 4 | 0 | 0 | 4 | 4 |
| GPNMB | 2 | 6 | 0 | 1 | 9 | 7 |
| GPR108 | 0 | 0 | 0 | 1 | 1 | 1 |
| GPR111 | 0 | 0 | 0 | 1 | 1 | 1 |
| GPR112 | 0 | 0 | 0 | 2 | 2 | 2 |
| GPR113 | 0 | 3 | 0 | 0 | 3 | 3 |
| GPR114 | 0 | 0 | 0 | 1 | 1 | 1 |
| GPR115 | 0 | 1 | 0 | 0 | 1 | 1 |
| GPR116 | 0 | 0 | 1 | 0 | 1 | 0 |
| GPR125 | 0 | 0 | 0 | 11 | 11 | 11 |
| GPR128 | 0 | 0 | 0 | 9 | 9 | 9 |
| GPR133 | 0 | 0 | 0 | 2 | 2 | 2 |
| GPR135 | 0 | 0 | 0 | 1 | 1 | 1 |
| GPR142 | 0 | 0 | 0 | 10 | 10 | 10 |
| GPR149 | 0 | 2 | 0 | 0 | 2 | 2 |
| GPR151 | 0 | 3 | 0 | 3 | 6 | 6 |
| GPR155 | 0 | 1 | 0 | 0 | 1 | 1 |
| GPR156 | 0 | 0 | 0 | 1 | 1 | 1 |
| GPR17 | 0 | 1 | 0 | 0 | 1 | 1 |
| GPR172B | 0 | 0 | 0 | 1 | 1 | 1 |
| GPR173 | 0 | 0 | 0 | 2 | 2 | 2 |
| GPR179 | 0 | 1 | 0 | 1 | 2 | 2 |
| GPR183 | 0 | 7 | 0 | 0 | 7 | 7 |
| GPR4 | 0 | 0 | 0 | 1 | 1 | 1 |
| GPR84 | 0 | 1 | 0 | 1 | 2 | 2 |
| GPR87 | 0 | 0 | 0 | 1 | 1 | 1 |
| GPR89A | 0 | 0 | 0 | 2 | 2 | 2 |
| GPR89B | 0 | 0 | 0 | 2 | 2 | 2 |
| GPR97 | 0 | 0 | 0 | 1 | 1 | 1 |
| GPR98 | 1 | 1 | 0 | 2 | 4 | 3 |
| GPRASP1 | 0 | 0 | 0 | 1 | 1 | 1 |
| GPRC5A | 0 | 0 | 0 | 4 | 4 | 4 |
| GPRC5C | 0 | 0 | 0 | 1 | 1 | 1 |
| GPRC5D | 0 | 0 | 0 | 5 | 5 | 5 |
| GPRIN1 | 0 | 1 | 0 | 1 | 2 | 2 |
| GPSM3 | 0 | 0 | 0 | 1 | 1 | 1 |
| GRAMD1A | 2 | 0 | 0 | 0 | 2 | 0 |
| GRAMD1B | 0 | 0 | 0 | 1 | 1 | 1 |
| GRAMD2 | 0 | 0 | 0 | 3 | 3 | 3 |
| GRAMD4 | 0 | 0 | 0 | 1 | 1 | 1 |
| GRB7 | 0 | 0 | 0 | 1 | 1 | 1 |
| GREB1 | 0 | 0 | 0 | 1 | 1 | 1 |
| GRHL2 | 0 | 1 | 0 | 0 | 1 | 1 |
| GRIA1 | 4 | 0 | 0 | 0 | 4 | 0 |
| GRIK3 | 0 | 0 | 0 | 1 | 1 | 1 |
| GRIN2B | 0 | 1 | 0 | 0 | 1 | 1 |
| GRIN3B | 0 | 1 | 0 | 0 | 1 | 1 |
| GRIP1 | 1 | 0 | 0 | 0 | 1 | 0 |
| GRIP2 | 0 | 5 | 0 | 4 | 9 | 9 |
| GRIPAP1 | 0 | 0 | 0 | 1 | 1 | 1 |
| GRM2 | 0 | 0 | 0 | 1 | 1 | 1 |
| GRM3 | 0 | 0 | 0 | 1 | 1 | 1 |
| GRM5 | 0 | 1 | 0 | 0 | 1 | 1 |
| GRN | 0 | 0 | 0 | 1 | 1 | 1 |
| GRXCR2 | 0 | 0 | 0 | 4 | 4 | 4 |
| GSC | 0 | 1 | 0 | 0 | 1 | 1 |
| GSG1 | 0 | 7 | 0 | 0 | 7 | 7 |
| GSG2 | 0 | 0 | 0 | 2 | 2 | 2 |
| GSS | 0 | 0 | 0 | 1 | 1 | 1 |
| GSTA1 | 0 | 0 | 0 | 1 | 1 | 1 |
| GSTA3 | 0 | 0 | 0 | 1 | 1 | 1 |
| GSTA5 | 1 | 0 | 0 | 0 | 1 | 0 |
| GSTM4 | 2 | 0 | 0 | 3 | 5 | 3 |
| GSTO2 | 1 | 0 | 0 | 0 | 1 | 0 |
| GSTT2 | 0 | 1 | 0 | 0 | 1 | 1 |
| GTF2B | 0 | 0 | 0 | 1 | 1 | 1 |
| GTF2F1 | 2 | 0 | 0 | 0 | 2 | 0 |
| GTF2H4 | 0 | 0 | 0 | 2 | 2 | 2 |
| GTF2I | 0 | 0 | 0 | 2 | 2 | 2 |
| GTF2IRD2 | 0 | 1 | 0 | 0 | 1 | 1 |
| GTF3C1 | 0 | 0 | 0 | 3 | 3 | 3 |
| GTF3C5 | 0 | 0 | 0 | 1 | 1 | 1 |
| GTPBP10 | 0 | 1 | 0 | 0 | 1 | 1 |
| GTPBP5 | 0 | 0 | 0 | 1 | 1 | 1 |
| GTPBP8 | 1 | 0 | 0 | 0 | 1 | 0 |
| GTSE1 | 1 | 0 | 0 | 1 | 2 | 1 |
| GUCA1C | 0 | 7 | 0 | 0 | 7 | 7 |
| GUCA2A | 0 | 1 | 0 | 0 | 1 | 1 |
| GUCY2C | 0 | 1 | 0 | 0 | 1 | 1 |
| GUCY2D | 1 | 0 | 0 | 1 | 2 | 1 |
| GUCY2F | 1 | 0 | 0 | 5 | 6 | 5 |
| GUF1 | 2 | 0 | 0 | 2 | 4 | 2 |
| GUSB | 0 | 1 | 0 | 0 | 1 | 1 |
| GYG2 | 1 | 0 | 0 | 0 | 1 | 0 |
| GYLTL1B | 0 | 2 | 0 | 0 | 2 | 2 |
| GZMA | 0 | 1 | 0 | 0 | 1 | 1 |
| GZMH | 0 | 0 | 1 | 0 | 1 | 0 |
| H2AFY2 | 0 | 0 | 0 | 1 | 1 | 1 |
| H6PD | 0 | 1 | 0 | 0 | 1 | 1 |
| HAAO | 0 | 1 | 0 | 0 | 1 | 1 |
| HABP2 | 0 | 2 | 0 | 0 | 2 | 2 |
| HACL1 | 0 | 0 | 0 | 1 | 1 | 1 |
| HADH | 0 | 0 | 0 | 1 | 1 | 1 |
| HADHB | 0 | 0 | 0 | 7 | 7 | 7 |
| HAO1 | 0 | 1 | 0 | 0 | 1 | 1 |
| HAO2 | 0 | 0 | 0 | 1 | 1 | 1 |
| HAP1 | 0 | 4 | 0 | 0 | 4 | 4 |
| HAPLN4 | 0 | 1 | 0 | 0 | 1 | 1 |
| HAS2 | 0 | 0 | 0 | 1 | 1 | 1 |
| HAUS1 | 0 | 1 | 0 | 0 | 1 | 1 |
| HAUS3 | 0 | 1 | 0 | 1 | 2 | 2 |
| HAUS4 | 1 | 0 | 0 | 0 | 1 | 0 |
| HAVCR1 | 0 | 0 | 0 | 1 | 1 | 1 |
| HAVCR2 | 0 | 0 | 0 | 1 | 1 | 1 |
| HBB | 0 | 1 | 0 | 0 | 1 | 1 |
| HCAR3 | 0 | 0 | 0 | 4 | 4 | 4 |
| HCFC1 | 0 | 0 | 0 | 1 | 1 | 1 |
| HCN3 | 0 | 0 | 0 | 1 | 1 | 1 |
| HCN4 | 0 | 4 | 0 | 0 | 4 | 4 |
| HDAC1 | 0 | 0 | 0 | 2 | 2 | 2 |
| HDAC4 | 0 | 0 | 0 | 1 | 1 | 1 |
| HDGFRP3 | 14 | 0 | 0 | 0 | 14 | 0 |
| HEATR1 | 3 | 1 | 0 | 0 | 4 | 1 |
| HEATR7A | 0 | 0 | 0 | 1 | 1 | 1 |
| HEATR7B2 | 0 | 2 | 0 | 5 | 7 | 7 |
| HEATR8 | 0 | 1 | 0 | 0 | 1 | 1 |
| HECTD1 | 4 | 0 | 0 | 0 | 4 | 0 |
| HECW1 | 0 | 0 | 0 | 1 | 1 | 1 |
| HECW2 | 0 | 3 | 0 | 0 | 3 | 3 |
| HEG1 | 0 | 0 | 0 | 1 | 1 | 1 |
| HELB | 0 | 0 | 0 | 21 | 21 | 21 |
| HEPACAM2 | 0 | 7 | 0 | 0 | 7 | 7 |
| HEPHL1 | 1 | 2 | 0 | 1 | 4 | 3 |
| HERC1 | 0 | 0 | 0 | 2 | 2 | 2 |
| HERC6 | 0 | 0 | 4 | 1 | 5 | 1 |
| HERPUD1 | 0 | 0 | 0 | 1 | 1 | 1 |
| HESX1 | 1 | 0 | 0 | 0 | 1 | 0 |
| HEXA | 0 | 0 | 0 | 1 | 1 | 1 |
| HFM1 | 1 | 5 | 0 | 1 | 7 | 6 |
| HGSNAT | 0 | 1 | 0 | 0 | 1 | 1 |
| HHATL | 0 | 0 | 0 | 1 | 1 | 1 |
| HHIP | 13 | 0 | 0 | 0 | 13 | 0 |
| HHIPL2 | 0 | 0 | 0 | 4 | 4 | 4 |
| HHLA2 | 1 | 1 | 0 | 1 | 3 | 2 |
| HIAT1 | 0 | 0 | 0 | 1 | 1 | 1 |
| HIBCH | 0 | 0 | 0 | 1 | 1 | 1 |
| HIC1 | 0 | 0 | 0 | 1 | 1 | 1 |
| HIGD1B | 0 | 0 | 1 | 0 | 1 | 0 |
| HINT3 | 0 | 1 | 0 | 0 | 1 | 1 |
| HIP1R | 0 | 0 | 0 | 1 | 1 | 1 |
| HIPK1 | 0 | 1 | 0 | 0 | 1 | 1 |
| HIPK3 | 0 | 0 | 0 | 1 | 1 | 1 |
| HIST1H1T | 0 | 1 | 0 | 1 | 2 | 2 |
| HIST1H2AA | 0 | 0 | 0 | 1 | 1 | 1 |
| HIST1H2AB | 0 | 0 | 0 | 1 | 1 | 1 |
| HIST1H2AK | 0 | 0 | 0 | 1 | 1 | 1 |
| HIST1H2AL | 0 | 0 | 0 | 1 | 1 | 1 |
| HIST1H2BE | 0 | 0 | 0 | 2 | 2 | 2 |
| HIST1H2BG | 0 | 1 | 0 | 0 | 1 | 1 |
| HIST1H3A | 0 | 1 | 0 | 0 | 1 | 1 |
| HIST1H4B | 0 | 4 | 0 | 0 | 4 | 4 |
| HIST1H4D | 0 | 0 | 0 | 1 | 1 | 1 |
| HIST1H4K | 0 | 0 | 0 | 1 | 1 | 1 |
| HIST1H4L | 0 | 0 | 0 | 1 | 1 | 1 |
| HIST3H2BB | 0 | 0 | 0 | 1 | 1 | 1 |
| HIVEP2 | 0 | 0 | 0 | 1 | 1 | 1 |
| HIVEP3 | 0 | 3 | 0 | 0 | 3 | 3 |
| HJURP | 1 | 0 | 0 | 0 | 1 | 0 |
| HK3 | 0 | 1 | 0 | 0 | 1 | 1 |
| HKDC1 | 0 | 1 | 0 | 1 | 2 | 2 |
| HKR1 | 0 | 2 | 0 | 1 | 3 | 3 |
| HLA-A | 0 | 0 | 0 | 1 | 1 | 1 |
| HLA-B | 0 | 0 | 0 | 68 | 68 | 68 |
| HLA-DPA1 | 0 | 1 | 0 | 0 | 1 | 1 |
| HLA-DQB1 | 0 | 1 | 0 | 6 | 7 | 7 |
| HLA-DRB1 | 5 | 0 | 0 | 45 | 50 | 45 |
| HLA-G | 0 | 0 | 0 | 12 | 12 | 12 |
| HLCS | 0 | 0 | 0 | 1 | 1 | 1 |
| HLX | 0 | 0 | 0 | 1 | 1 | 1 |
| HMCN1 | 0 | 0 | 0 | 1 | 1 | 1 |
| HMGXB3 | 0 | 0 | 0 | 4 | 4 | 4 |
| HMMR | 0 | 6 | 0 | 0 | 6 | 6 |
| HNF1A | 0 | 0 | 0 | 5 | 5 | 5 |
| HNRNPA1L2 | 0 | 0 | 0 | 8 | 8 | 8 |
| HNRNPD | 1 | 0 | 0 | 0 | 1 | 0 |
| HNRPLL | 1 | 0 | 0 | 0 | 1 | 0 |
| HOMER2 | 0 | 1 | 0 | 0 | 1 | 1 |
| HOOK2 | 0 | 0 | 0 | 1 | 1 | 1 |
| HOOK3 | 0 | 1 | 0 | 0 | 1 | 1 |
| HORMAD1 | 0 | 6 | 0 | 0 | 6 | 6 |
| HORMAD2 | 1 | 0 | 0 | 0 | 1 | 0 |
| HOXA1 | 0 | 0 | 0 | 2 | 2 | 2 |
| HOXA10 | 1 | 0 | 0 | 0 | 1 | 0 |
| HOXA11 | 0 | 0 | 0 | 2 | 2 | 2 |
| HOXA6 | 0 | 0 | 0 | 9 | 9 | 9 |
| HPGD | 0 | 0 | 0 | 1 | 1 | 1 |
| HPS4 | 0 | 0 | 0 | 1 | 1 | 1 |
| HPSE | 0 | 3 | 0 | 0 | 3 | 3 |
| HPSE2 | 0 | 0 | 0 | 1 | 1 | 1 |
| HRASLS | 0 | 1 | 0 | 0 | 1 | 1 |
| HRNR | 0 | 4 | 0 | 0 | 4 | 4 |
| HS6ST1 | 0 | 1 | 0 | 0 | 1 | 1 |
| HS6ST2 | 0 | 0 | 0 | 4 | 4 | 4 |
| HSD11B2 | 0 | 0 | 0 | 1 | 1 | 1 |
| HSD17B13 | 0 | 1 | 0 | 9 | 10 | 10 |
| HSD17B14 | 1 | 1 | 0 | 2 | 4 | 3 |
| HSD17B2 | 0 | 0 | 3 | 0 | 3 | 0 |
| HSD17B4 | 0 | 0 | 0 | 3 | 3 | 3 |
| HSF2 | 0 | 0 | 0 | 1 | 1 | 1 |
| HSF4 | 0 | 0 | 0 | 1 | 1 | 1 |
| HSH2D | 0 | 1 | 0 | 2 | 3 | 3 |
| HSP90B1 | 1 | 0 | 0 | 0 | 1 | 0 |
| HSPA13 | 0 | 0 | 0 | 1 | 1 | 1 |
| HSPA6 | 0 | 5 | 0 | 0 | 5 | 5 |
| HSPA9 | 0 | 0 | 0 | 1 | 1 | 1 |
| HSPG2 | 1 | 0 | 0 | 4 | 5 | 4 |
| HTN3 | 0 | 3 | 0 | 0 | 3 | 3 |
| HTR1A | 0 | 0 | 0 | 1 | 1 | 1 |
| HTR1E | 0 | 0 | 0 | 1 | 1 | 1 |
| HTR3A | 1 | 0 | 0 | 0 | 1 | 0 |
| HTR3E | 0 | 1 | 0 | 0 | 1 | 1 |
| HTRA4 | 1 | 0 | 0 | 0 | 1 | 0 |
| HYDIN | 1 | 5 | 0 | 2 | 8 | 7 |
| IBSP | 0 | 0 | 0 | 5 | 5 | 5 |
| ICK | 0 | 1 | 0 | 0 | 1 | 1 |
| IDH3A | 1 | 0 | 0 | 0 | 1 | 0 |
| IDH3G | 0 | 0 | 0 | 1 | 1 | 1 |
| IDI2 | 0 | 0 | 11 | 1 | 12 | 1 |
| IFI27L1 | 1 | 0 | 0 | 0 | 1 | 0 |
| IFI35 | 0 | 0 | 0 | 1 | 1 | 1 |
| IFI44 | 0 | 1 | 0 | 0 | 1 | 1 |
| IFI44L | 0 | 3 | 0 | 0 | 3 | 3 |
| IFIH1 | 11 | 4 | 0 | 1 | 16 | 5 |
| IFIT1 | 0 | 1 | 0 | 0 | 1 | 1 |
| IFIT3 | 0 | 1 | 0 | 2 | 3 | 3 |
| IFLTD1 | 0 | 1 | 0 | 0 | 1 | 1 |
| IFNA10 | 0 | 1 | 0 | 1 | 2 | 2 |
| IFNA5 | 0 | 4 | 0 | 0 | 4 | 4 |
| IFNA6 | 0 | 1 | 0 | 0 | 1 | 1 |
| IFNAR1 | 0 | 0 | 0 | 1 | 1 | 1 |
| IFNB1 | 0 | 1 | 0 | 0 | 1 | 1 |
| IFNE | 0 | 5 | 0 | 0 | 5 | 5 |
| IFNK | 0 | 0 | 0 | 19 | 19 | 19 |
| IFNW1 | 0 | 0 | 0 | 1 | 1 | 1 |
| IFT122 | 0 | 1 | 0 | 1 | 2 | 2 |
| IFT140 | 0 | 0 | 0 | 1 | 1 | 1 |
| IFT172 | 2 | 0 | 0 | 0 | 2 | 0 |
| IFT43 | 1 | 0 | 0 | 0 | 1 | 0 |
| IFT74 | 3 | 0 | 0 | 0 | 3 | 0 |
| IFT80 | 0 | 0 | 0 | 1 | 1 | 1 |
| IGDCC3 | 0 | 0 | 0 | 2 | 2 | 2 |
| IGDCC4 | 0 | 4 | 0 | 1 | 5 | 5 |
| IGF2BP2 | 0 | 0 | 3 | 0 | 3 | 0 |
| IGFBP1 | 0 | 0 | 0 | 1 | 1 | 1 |
| IGFBPL1 | 0 | 0 | 0 | 2 | 2 | 2 |
| IGFLR1 | 0 | 0 | 0 | 4 | 4 | 4 |
| IGHMBP2 | 0 | 0 | 0 | 3 | 3 | 3 |
| IGLL1 | 0 | 0 | 0 | 2 | 2 | 2 |
| IGSF1 | 0 | 0 | 0 | 1 | 1 | 1 |
| IGSF10 | 0 | 3 | 0 | 4 | 7 | 7 |
| IGSF22 | 4 | 1 | 0 | 0 | 5 | 1 |
| IGSF3 | 0 | 0 | 0 | 5 | 5 | 5 |
| IKBKAP | 0 | 1 | 0 | 5 | 6 | 6 |
| IKBKE | 1 | 0 | 0 | 0 | 1 | 0 |
| IKZF2 | 0 | 4 | 0 | 0 | 4 | 4 |
| IKZF4 | 0 | 0 | 0 | 2 | 2 | 2 |
| IL12B | 0 | 0 | 0 | 1 | 1 | 1 |
| IL12RB1 | 0 | 1 | 0 | 0 | 1 | 1 |
| IL17RB | 2 | 0 | 0 | 1 | 3 | 1 |
| IL18RAP | 0 | 0 | 0 | 2 | 2 | 2 |
| IL19 | 1 | 0 | 0 | 0 | 1 | 0 |
| IL1R1 | 0 | 0 | 0 | 5 | 5 | 5 |
| IL20 | 1 | 0 | 0 | 3 | 4 | 3 |
| IL21R | 0 | 0 | 0 | 1 | 1 | 1 |
| IL23R | 0 | 0 | 0 | 1 | 1 | 1 |
| IL28A | 0 | 1 | 0 | 0 | 1 | 1 |
| IL4R | 0 | 0 | 0 | 1 | 1 | 1 |
| IL6ST | 0 | 0 | 0 | 1 | 1 | 1 |
| IL8 | 0 | 4 | 0 | 0 | 4 | 4 |
| IL9 | 0 | 0 | 0 | 1 | 1 | 1 |
| ILVBL | 0 | 1 | 0 | 0 | 1 | 1 |
| IMMT | 0 | 1 | 0 | 0 | 1 | 1 |
| IMP3 | 0 | 0 | 1 | 0 | 1 | 0 |
| IMP5 | 0 | 1 | 0 | 0 | 1 | 1 |
| IMPA1 | 5 | 0 | 0 | 0 | 5 | 0 |
| IMPA2 | 0 | 6 | 0 | 0 | 6 | 6 |
| INADL | 0 | 1 | 0 | 1 | 2 | 2 |
| INCA1 | 0 | 5 | 0 | 1 | 6 | 6 |
| INCENP | 0 | 0 | 0 | 3 | 3 | 3 |
| INHBC | 0 | 0 | 0 | 3 | 3 | 3 |
| INMT | 0 | 2 | 16 | 0 | 18 | 2 |
| INPP1 | 3 | 0 | 0 | 0 | 3 | 0 |
| INPP4B | 0 | 1 | 0 | 0 | 1 | 1 |
| INPP5D | 0 | 1 | 0 | 0 | 1 | 1 |
| INPP5J | 1 | 1 | 0 | 0 | 2 | 1 |
| INPP5K | 0 | 0 | 0 | 1 | 1 | 1 |
| INPPL1 | 0 | 0 | 0 | 1 | 1 | 1 |
| INSC | 0 | 4 | 0 | 1 | 5 | 5 |
| INSR | 0 | 0 | 0 | 1 | 1 | 1 |
| INSRR | 1 | 1 | 0 | 2 | 4 | 3 |
| INTS1 | 0 | 0 | 0 | 1 | 1 | 1 |
| INTS3 | 0 | 7 | 0 | 0 | 7 | 7 |
| INTU | 0 | 0 | 0 | 1 | 1 | 1 |
| IP6K2 | 0 | 1 | 0 | 0 | 1 | 1 |
| IPO11 | 1 | 0 | 0 | 0 | 1 | 0 |
| IPO13 | 0 | 0 | 0 | 2 | 2 | 2 |
| IPO5 | 3 | 2 | 0 | 0 | 5 | 2 |
| IPO7 | 0 | 0 | 0 | 3 | 3 | 3 |
| IPO8 | 0 | 1 | 0 | 0 | 1 | 1 |
| IQCA1 | 0 | 0 | 0 | 1 | 1 | 1 |
| IQCE | 1 | 0 | 0 | 4 | 5 | 4 |
| IQCF1 | 0 | 1 | 1 | 0 | 2 | 1 |
| IQCF3 | 0 | 9 | 0 | 0 | 9 | 9 |
| IQCG | 0 | 1 | 0 | 0 | 1 | 1 |
| IQCH | 0 | 2 | 0 | 0 | 2 | 2 |
| IQGAP1 | 0 | 0 | 0 | 12 | 12 | 12 |
| IQGAP2 | 0 | 1 | 0 | 0 | 1 | 1 |
| IQGAP3 | 2 | 0 | 0 | 1 | 3 | 1 |
| IRAK3 | 3 | 0 | 0 | 0 | 3 | 0 |
| IRAK4 | 0 | 1 | 0 | 0 | 1 | 1 |
| IRS1 | 0 | 0 | 0 | 3 | 3 | 3 |
| IRS2 | 0 | 1 | 0 | 0 | 1 | 1 |
| IRS4 | 0 | 0 | 0 | 1 | 1 | 1 |
| IRX1 | 0 | 0 | 0 | 2 | 2 | 2 |
| IRX3 | 0 | 0 | 0 | 1 | 1 | 1 |
| IRX4 | 0 | 0 | 0 | 1 | 1 | 1 |
| ISCU | 0 | 0 | 0 | 1 | 1 | 1 |
| ISM2 | 0 | 2 | 0 | 0 | 2 | 2 |
| ISX | 1 | 0 | 0 | 0 | 1 | 0 |
| ISYNA1 | 0 | 1 | 0 | 0 | 1 | 1 |
| ITGA1 | 0 | 0 | 0 | 1 | 1 | 1 |
| ITGA10 | 0 | 1 | 0 | 1 | 2 | 2 |
| ITGA2 | 2 | 0 | 0 | 0 | 2 | 0 |
| ITGA2B | 0 | 0 | 0 | 1 | 1 | 1 |
| ITGA4 | 0 | 0 | 0 | 1 | 1 | 1 |
| ITGA5 | 0 | 8 | 0 | 0 | 8 | 8 |
| ITGA6 | 0 | 1 | 0 | 0 | 1 | 1 |
| ITGAD | 0 | 1 | 0 | 0 | 1 | 1 |
| ITGAE | 2 | 0 | 0 | 0 | 2 | 0 |
| ITGAL | 3 | 0 | 0 | 0 | 3 | 0 |
| ITGAV | 0 | 2 | 0 | 0 | 2 | 2 |
| ITGB1BP2 | 0 | 1 | 0 | 0 | 1 | 1 |
| ITGB3 | 0 | 0 | 0 | 2 | 2 | 2 |
| ITGB3BP | 0 | 0 | 0 | 1 | 1 | 1 |
| ITGB6 | 1 | 2 | 0 | 1 | 4 | 3 |
| ITGB7 | 0 | 0 | 0 | 1 | 1 | 1 |
| ITGB8 | 0 | 1 | 0 | 0 | 1 | 1 |
| ITIH1 | 0 | 2 | 0 | 1 | 3 | 3 |
| ITIH2 | 1 | 0 | 0 | 0 | 1 | 0 |
| ITIH6 | 14 | 0 | 0 | 1 | 15 | 1 |
| ITM2A | 0 | 0 | 0 | 1 | 1 | 1 |
| ITPKC | 2 | 0 | 0 | 0 | 2 | 0 |
| ITPR3 | 1 | 0 | 0 | 1 | 2 | 1 |
| ITPRIPL1 | 0 | 2 | 0 | 0 | 2 | 2 |
| ITSN1 | 0 | 0 | 0 | 8 | 8 | 8 |
| ITSN2 | 4 | 0 | 0 | 0 | 4 | 0 |
| JAKMIP1 | 0 | 1 | 0 | 0 | 1 | 1 |
| JAKMIP3 | 0 | 0 | 0 | 2 | 2 | 2 |
| JMJD4 | 1 | 0 | 0 | 0 | 1 | 0 |
| JUP | 0 | 0 | 0 | 5 | 5 | 5 |
| KAAG1 | 0 | 1 | 0 | 0 | 1 | 1 |
| KAL1 | 0 | 1 | 0 | 0 | 1 | 1 |
| KANK1 | 0 | 1 | 0 | 0 | 1 | 1 |
| KANK2 | 0 | 0 | 0 | 1 | 1 | 1 |
| KANK4 | 5 | 0 | 0 | 0 | 5 | 0 |
| KAT2A | 0 | 0 | 0 | 1 | 1 | 1 |
| KAT6A | 0 | 2 | 0 | 0 | 2 | 2 |
| KAT7 | 0 | 1 | 0 | 1 | 2 | 2 |
| KATNA1 | 0 | 0 | 1 | 0 | 1 | 0 |
| KATNAL2 | 0 | 1 | 0 | 0 | 1 | 1 |
| KAZN | 0 | 1 | 0 | 0 | 1 | 1 |
| KBTBD12 | 0 | 1 | 0 | 1 | 2 | 2 |
| KBTBD3 | 0 | 0 | 0 | 1 | 1 | 1 |
| KBTBD7 | 0 | 0 | 0 | 2 | 2 | 2 |
| KCMF1 | 0 | 0 | 0 | 2 | 2 | 2 |
| KCNA1 | 0 | 0 | 0 | 2 | 2 | 2 |
| KCNA3 | 0 | 0 | 0 | 1 | 1 | 1 |
| KCNA6 | 0 | 0 | 0 | 1 | 1 | 1 |
| KCNAB3 | 1 | 1 | 0 | 0 | 2 | 1 |
| KCND1 | 0 | 0 | 0 | 1 | 1 | 1 |
| KCND2 | 0 | 3 | 0 | 0 | 3 | 3 |
| KCNE1L | 0 | 6 | 0 | 0 | 6 | 6 |
| KCNE2 | 0 | 0 | 0 | 1 | 1 | 1 |
| KCNH1 | 1 | 0 | 0 | 0 | 1 | 0 |
| KCNH3 | 0 | 0 | 0 | 2 | 2 | 2 |
| KCNIP2 | 0 | 1 | 0 | 0 | 1 | 1 |
| KCNJ11 | 0 | 0 | 0 | 1 | 1 | 1 |
| KCNJ15 | 0 | 1 | 0 | 1 | 2 | 2 |
| KCNJ18 | 0 | 0 | 0 | 1 | 1 | 1 |
| KCNK1 | 0 | 2 | 0 | 0 | 2 | 2 |
| KCNK16 | 0 | 3 | 0 | 1 | 4 | 4 |
| KCNK18 | 0 | 0 | 0 | 1 | 1 | 1 |
| KCNK4 | 3 | 0 | 0 | 0 | 3 | 0 |
| KCNK5 | 0 | 0 | 0 | 1 | 1 | 1 |
| KCNMA1 | 0 | 0 | 0 | 2 | 2 | 2 |
| KCNMB1 | 0 | 0 | 0 | 1 | 1 | 1 |
| KCNQ4 | 0 | 0 | 0 | 1 | 1 | 1 |
| KCNQ5 | 0 | 2 | 0 | 0 | 2 | 2 |
| KCNRG | 0 | 0 | 0 | 2 | 2 | 2 |
| KCNT1 | 0 | 0 | 0 | 1 | 1 | 1 |
| KCNT2 | 0 | 7 | 0 | 0 | 7 | 7 |
| KCNU1 | 0 | 0 | 0 | 1 | 1 | 1 |
| KCTD11 | 0 | 0 | 0 | 1 | 1 | 1 |
| KCTD12 | 0 | 0 | 1 | 0 | 1 | 0 |
| KCTD17 | 1 | 0 | 0 | 0 | 1 | 0 |
| KCTD18 | 0 | 0 | 0 | 8 | 8 | 8 |
| KDELC1 | 0 | 0 | 0 | 1 | 1 | 1 |
| KDELR3 | 0 | 0 | 0 | 1 | 1 | 1 |
| KDM1B | 0 | 2 | 0 | 0 | 2 | 2 |
| KDM2B | 0 | 0 | 0 | 2 | 2 | 2 |
| KDM3A | 0 | 2 | 0 | 1 | 3 | 3 |
| KDM3B | 0 | 0 | 0 | 1 | 1 | 1 |
| KDM5C | 0 | 2 | 0 | 2 | 4 | 4 |
| KDM6A | 0 | 0 | 0 | 5 | 5 | 5 |
| KEAP1 | 0 | 0 | 0 | 1 | 1 | 1 |
| KEL | 0 | 0 | 0 | 1 | 1 | 1 |
| KHDC1 | 0 | 0 | 0 | 2 | 2 | 2 |
| KIAA0020 | 1 | 0 | 0 | 0 | 1 | 0 |
| KIAA0090 | 1 | 0 | 0 | 0 | 1 | 0 |
| KIAA0146 | 0 | 0 | 0 | 1 | 1 | 1 |
| KIAA0182 | 0 | 1 | 0 | 0 | 1 | 1 |
| KIAA0195 | 0 | 0 | 0 | 4 | 4 | 4 |
| KIAA0196 | 0 | 1 | 0 | 0 | 1 | 1 |
| KIAA0319 | 0 | 0 | 0 | 1 | 1 | 1 |
| KIAA0368 | 0 | 0 | 0 | 1 | 1 | 1 |
| KIAA0408 | 0 | 1 | 0 | 1 | 2 | 2 |
| KIAA0415 | 1 | 0 | 0 | 0 | 1 | 0 |
| KIAA0430 | 0 | 0 | 0 | 1 | 1 | 1 |
| KIAA0513 | 0 | 12 | 0 | 0 | 12 | 12 |
| KIAA0528 | 1 | 16 | 0 | 0 | 17 | 16 |
| KIAA0556 | 0 | 0 | 0 | 1 | 1 | 1 |
| KIAA0564 | 1 | 2 | 0 | 2 | 5 | 4 |
| KIAA0586 | 1 | 0 | 0 | 4 | 5 | 4 |
| KIAA0664 | 0 | 0 | 0 | 1 | 1 | 1 |
| KIAA0753 | 0 | 2 | 0 | 1 | 3 | 3 |
| KIAA0895 | 0 | 0 | 0 | 5 | 5 | 5 |
| KIAA0907 | 0 | 0 | 0 | 3 | 3 | 3 |
| KIAA0913 | 0 | 0 | 0 | 6 | 6 | 6 |
| KIAA0922 | 1 | 0 | 0 | 0 | 1 | 0 |
| KIAA1199 | 0 | 0 | 0 | 1 | 1 | 1 |
| KIAA1211 | 0 | 0 | 0 | 2 | 2 | 2 |
| KIAA1239 | 0 | 7 | 0 | 0 | 7 | 7 |
| KIAA1257 | 0 | 0 | 0 | 2 | 2 | 2 |
| KIAA1279 | 0 | 1 | 0 | 0 | 1 | 1 |
| KIAA1324L | 0 | 0 | 0 | 2 | 2 | 2 |
| KIAA1370 | 0 | 0 | 0 | 2 | 2 | 2 |
| KIAA1407 | 0 | 0 | 0 | 20 | 20 | 20 |
| KIAA1429 | 2 | 1 | 0 | 0 | 3 | 1 |
| KIAA1530 | 0 | 0 | 0 | 1 | 1 | 1 |
| KIAA1549 | 0 | 0 | 0 | 2 | 2 | 2 |
| KIAA1586 | 0 | 0 | 0 | 2 | 2 | 2 |
| KIAA1609 | 1 | 0 | 0 | 0 | 1 | 0 |
| KIAA1614 | 0 | 0 | 0 | 2 | 2 | 2 |
| KIAA1731 | 0 | 0 | 0 | 1 | 1 | 1 |
| KIAA1755 | 1 | 13 | 0 | 0 | 14 | 13 |
| KIAA1797 | 1 | 0 | 0 | 0 | 1 | 0 |
| KIAA1841 | 0 | 1 | 0 | 1 | 2 | 2 |
| KIAA1958 | 0 | 0 | 0 | 4 | 4 | 4 |
| KIAA2018 | 0 | 0 | 0 | 2 | 2 | 2 |
| KIF12 | 0 | 1 | 0 | 0 | 1 | 1 |
| KIF15 | 0 | 1 | 0 | 0 | 1 | 1 |
| KIF18A | 0 | 1 | 0 | 0 | 1 | 1 |
| KIF18B | 1 | 0 | 0 | 0 | 1 | 0 |
| KIF1A | 0 | 0 | 0 | 2 | 2 | 2 |
| KIF1B | 0 | 2 | 0 | 0 | 2 | 2 |
| KIF20A | 0 | 0 | 0 | 1 | 1 | 1 |
| KIF20B | 0 | 1 | 0 | 2 | 3 | 3 |
| KIF24 | 1 | 3 | 0 | 1 | 5 | 4 |
| KIF26B | 0 | 0 | 0 | 4 | 4 | 4 |
| KIF3A | 0 | 1 | 0 | 0 | 1 | 1 |
| KIF4A | 2 | 0 | 0 | 0 | 2 | 0 |
| KIF4B | 0 | 0 | 0 | 1 | 1 | 1 |
| KIF5A | 0 | 0 | 0 | 4 | 4 | 4 |
| KIF5C | 7 | 0 | 0 | 0 | 7 | 0 |
| KIF6 | 7 | 0 | 0 | 1 | 8 | 1 |
| KIF7 | 0 | 1 | 0 | 0 | 1 | 1 |
| KIFC3 | 0 | 0 | 0 | 1 | 1 | 1 |
| KIR2DL1 | 0 | 2 | 0 | 8 | 10 | 10 |
| KIR2DL4 | 0 | 0 | 0 | 10 | 10 | 10 |
| KIR2DS4 | 1 | 0 | 0 | 14 | 15 | 14 |
| KIR3DL1 | 0 | 2 | 0 | 0 | 2 | 2 |
| KIR3DL2 | 0 | 0 | 0 | 1 | 1 | 1 |
| KIRREL2 | 0 | 1 | 0 | 0 | 1 | 1 |
| KIRREL3 | 0 | 0 | 0 | 1 | 1 | 1 |
| KIT | 0 | 0 | 0 | 2 | 2 | 2 |
| KLHDC1 | 0 | 1 | 0 | 0 | 1 | 1 |
| KLHDC4 | 0 | 1 | 0 | 0 | 1 | 1 |
| KLHDC7A | 0 | 3 | 0 | 0 | 3 | 3 |
| KLHDC8B | 0 | 0 | 0 | 1 | 1 | 1 |
| KLHL1 | 0 | 0 | 0 | 1 | 1 | 1 |
| KLHL13 | 0 | 0 | 0 | 1 | 1 | 1 |
| KLHL25 | 0 | 0 | 0 | 1 | 1 | 1 |
| KLHL29 | 0 | 0 | 0 | 5 | 5 | 5 |
| KLHL32 | 2 | 2 | 0 | 0 | 4 | 2 |
| KLHL38 | 0 | 0 | 0 | 2 | 2 | 2 |
| KLK14 | 2 | 0 | 0 | 0 | 2 | 0 |
| KLK3 | 0 | 0 | 1 | 0 | 1 | 0 |
| KLK4 | 0 | 1 | 2 | 0 | 3 | 1 |
| KLK7 | 0 | 0 | 0 | 1 | 1 | 1 |
| KLKB1 | 0 | 0 | 0 | 2 | 2 | 2 |
| KLRC2 | 0 | 0 | 0 | 3 | 3 | 3 |
| KLRC3 | 3 | 0 | 0 | 0 | 3 | 0 |
| KLRC4-KLRK1 | 1 | 0 | 0 | 0 | 1 | 0 |
| KLRK1 | 0 | 0 | 0 | 1 | 1 | 1 |
| KNG1 | 1 | 0 | 0 | 0 | 1 | 0 |
| KNTC1 | 0 | 0 | 0 | 2 | 2 | 2 |
| KPNA1 | 0 | 0 | 0 | 1 | 1 | 1 |
| KPNA4 | 1 | 0 | 0 | 0 | 1 | 0 |
| KPNA5 | 0 | 1 | 0 | 0 | 1 | 1 |
| KPNA7 | 0 | 3 | 0 | 0 | 3 | 3 |
| KPRP | 0 | 0 | 1 | 2 | 3 | 2 |
| KPTN | 0 | 0 | 0 | 1 | 1 | 1 |
| KRBA1 | 0 | 0 | 0 | 8 | 8 | 8 |
| KRBA2 | 0 | 0 | 0 | 13 | 13 | 13 |
| KRI1 | 0 | 0 | 0 | 1 | 1 | 1 |
| KRT14 | 0 | 0 | 0 | 2 | 2 | 2 |
| KRT15 | 0 | 1 | 0 | 1 | 2 | 2 |
| KRT20 | 0 | 0 | 0 | 1 | 1 | 1 |
| KRT222 | 0 | 1 | 0 | 1 | 2 | 2 |
| KRT23 | 0 | 1 | 0 | 0 | 1 | 1 |
| KRT24 | 0 | 1 | 0 | 0 | 1 | 1 |
| KRT25 | 0 | 1 | 0 | 0 | 1 | 1 |
| KRT27 | 1 | 0 | 0 | 0 | 1 | 0 |
| KRT28 | 0 | 1 | 0 | 0 | 1 | 1 |
| KRT31 | 18 | 0 | 0 | 0 | 18 | 0 |
| KRT33A | 0 | 0 | 0 | 1 | 1 | 1 |
| KRT34 | 0 | 1 | 0 | 0 | 1 | 1 |
| KRT35 | 1 | 1 | 0 | 0 | 2 | 1 |
| KRT36 | 0 | 1 | 0 | 0 | 1 | 1 |
| KRT38 | 0 | 4 | 0 | 1 | 5 | 5 |
| KRT4 | 0 | 0 | 0 | 7 | 7 | 7 |
| KRT6A | 0 | 0 | 0 | 2 | 2 | 2 |
| KRT6B | 0 | 0 | 0 | 1 | 1 | 1 |
| KRT6C | 1 | 0 | 0 | 0 | 1 | 0 |
| KRT71 | 0 | 0 | 0 | 1 | 1 | 1 |
| KRT72 | 0 | 0 | 0 | 1 | 1 | 1 |
| KRT74 | 2 | 0 | 0 | 0 | 2 | 0 |
| KRT75 | 0 | 0 | 0 | 1 | 1 | 1 |
| KRT76 | 0 | 10 | 0 | 0 | 10 | 10 |
| KRT78 | 0 | 0 | 0 | 3 | 3 | 3 |
| KRT79 | 0 | 0 | 0 | 2 | 2 | 2 |
| KRT81 | 0 | 2 | 0 | 0 | 2 | 2 |
| KRT82 | 0 | 3 | 0 | 0 | 3 | 3 |
| KRT83 | 0 | 24 | 0 | 2 | 26 | 26 |
| KRT85 | 0 | 1 | 0 | 0 | 1 | 1 |
| KRT86 | 0 | 0 | 0 | 2 | 2 | 2 |
| KRTAP1-1 | 0 | 1 | 0 | 3 | 4 | 4 |
| KRTAP1-5 | 0 | 0 | 0 | 14 | 14 | 14 |
| KRTAP10-11 | 0 | 0 | 0 | 1 | 1 | 1 |
| KRTAP10-4 | 0 | 0 | 10 | 0 | 10 | 0 |
| KRTAP10-6 | 0 | 0 | 0 | 14 | 14 | 14 |
| KRTAP10-7 | 0 | 1 | 0 | 0 | 1 | 1 |
| KRTAP10-8 | 0 | 1 | 0 | 0 | 1 | 1 |
| KRTAP12-1 | 0 | 0 | 0 | 1 | 1 | 1 |
| KRTAP13-1 | 0 | 4 | 1 | 2 | 7 | 6 |
| KRTAP13-2 | 0 | 6 | 0 | 0 | 6 | 6 |
| KRTAP15-1 | 0 | 13 | 0 | 0 | 13 | 13 |
| KRTAP19-3 | 0 | 0 | 0 | 2 | 2 | 2 |
| KRTAP21-2 | 0 | 2 | 0 | 0 | 2 | 2 |
| KRTAP24-1 | 0 | 0 | 0 | 1 | 1 | 1 |
| KRTAP27-1 | 0 | 1 | 0 | 1 | 2 | 2 |
| KRTAP4-2 | 0 | 1 | 0 | 0 | 1 | 1 |
| KRTAP4-5 | 0 | 1 | 0 | 0 | 1 | 1 |
| KRTAP5-1 | 0 | 4 | 0 | 0 | 4 | 4 |
| KRTAP5-10 | 0 | 0 | 0 | 1 | 1 | 1 |
| KRTAP5-4 | 0 | 0 | 0 | 1 | 1 | 1 |
| KRTAP6-2 | 0 | 0 | 0 | 3 | 3 | 3 |
| KRTAP9-1 | 0 | 1 | 0 | 1 | 2 | 2 |
| KSR1 | 0 | 0 | 0 | 1 | 1 | 1 |
| L1TD1 | 0 | 0 | 0 | 8 | 8 | 8 |
| LACC1 | 0 | 1 | 0 | 0 | 1 | 1 |
| LACE1 | 0 | 0 | 0 | 2 | 2 | 2 |
| LAD1 | 0 | 1 | 0 | 25 | 26 | 26 |
| LAG3 | 0 | 0 | 0 | 1 | 1 | 1 |
| LAIR2 | 0 | 18 | 0 | 0 | 18 | 18 |
| LAMA2 | 1 | 0 | 0 | 2 | 3 | 2 |
| LAMA4 | 0 | 0 | 0 | 1 | 1 | 1 |
| LAMB1 | 4 | 2 | 0 | 1 | 7 | 3 |
| LAMB2 | 0 | 1 | 0 | 0 | 1 | 1 |
| LAMB3 | 0 | 2 | 0 | 1 | 3 | 3 |
| LAMB4 | 2 | 0 | 0 | 7 | 9 | 7 |
| LAMC3 | 1 | 0 | 0 | 0 | 1 | 0 |
| LANCL2 | 0 | 1 | 0 | 0 | 1 | 1 |
| LAPTM4B | 0 | 0 | 0 | 1 | 1 | 1 |
| LARGE | 0 | 0 | 0 | 2 | 2 | 2 |
| LARP1 | 0 | 0 | 0 | 1 | 1 | 1 |
| LARP1B | 0 | 0 | 0 | 1 | 1 | 1 |
| LARP4 | 2 | 0 | 0 | 0 | 2 | 0 |
| LARP4B | 1 | 0 | 0 | 0 | 1 | 0 |
| LARP7 | 0 | 0 | 0 | 2 | 2 | 2 |
| LARS2 | 6 | 0 | 0 | 0 | 6 | 0 |
| LAT | 0 | 0 | 0 | 1 | 1 | 1 |
| LAX1 | 0 | 0 | 0 | 1 | 1 | 1 |
| LBP | 0 | 1 | 0 | 0 | 1 | 1 |
| LCA5L | 0 | 0 | 0 | 1 | 1 | 1 |
| LCE1D | 0 | 0 | 18 | 0 | 18 | 0 |
| LCE3C | 0 | 0 | 0 | 1 | 1 | 1 |
| LCE3E | 0 | 0 | 0 | 1 | 1 | 1 |
| LCE4A | 0 | 3 | 0 | 0 | 3 | 3 |
| LCN2 | 1 | 0 | 0 | 0 | 1 | 0 |
| LCORL | 0 | 0 | 0 | 1 | 1 | 1 |
| LCT | 0 | 0 | 0 | 2 | 2 | 2 |
| LDHB | 0 | 0 | 0 | 1 | 1 | 1 |
| LDHC | 0 | 0 | 0 | 1 | 1 | 1 |
| LDHD | 0 | 1 | 0 | 0 | 1 | 1 |
| LDLR | 1 | 0 | 0 | 1 | 2 | 1 |
| LDLRAD1 | 0 | 1 | 0 | 0 | 1 | 1 |
| LDLRAP1 | 0 | 0 | 0 | 1 | 1 | 1 |
| LECT1 | 0 | 1 | 0 | 0 | 1 | 1 |
| LEFTY1 | 0 | 0 | 0 | 1 | 1 | 1 |
| LEO1 | 0 | 0 | 0 | 1 | 1 | 1 |
| LEPRE1 | 0 | 1 | 0 | 0 | 1 | 1 |
| LETM2 | 0 | 1 | 0 | 1 | 2 | 2 |
| LFNG | 0 | 0 | 0 | 6 | 6 | 6 |
| LGALS12 | 0 | 0 | 0 | 1 | 1 | 1 |
| LGALS9B | 0 | 0 | 0 | 1 | 1 | 1 |
| LGI1 | 0 | 0 | 0 | 1 | 1 | 1 |
| LGI2 | 0 | 0 | 0 | 1 | 1 | 1 |
| LHFPL1 | 0 | 1 | 0 | 0 | 1 | 1 |
| LHFPL2 | 0 | 0 | 0 | 1 | 1 | 1 |
| LHX2 | 0 | 0 | 0 | 1 | 1 | 1 |
| LHX5 | 0 | 0 | 0 | 1 | 1 | 1 |
| LIG4 | 0 | 0 | 0 | 1 | 1 | 1 |
| LILRA1 | 0 | 1 | 0 | 1 | 2 | 2 |
| LILRA2 | 4 | 1 | 0 | 2 | 7 | 3 |
| LILRA3 | 1 | 0 | 0 | 4 | 5 | 4 |
| LILRA4 | 0 | 0 | 0 | 2 | 2 | 2 |
| LILRA5 | 1 | 0 | 0 | 0 | 1 | 0 |
| LILRA6 | 0 | 2 | 0 | 0 | 2 | 2 |
| LILRB1 | 2 | 1 | 0 | 1 | 4 | 2 |
| LILRB3 | 1 | 0 | 0 | 0 | 1 | 0 |
| LILRB4 | 10 | 0 | 0 | 0 | 10 | 0 |
| LIMA1 | 1 | 1 | 0 | 0 | 2 | 1 |
| LIMCH1 | 0 | 3 | 0 | 0 | 3 | 3 |
| LIMS1 | 5 | 1 | 0 | 0 | 6 | 1 |
| LIMS2 | 0 | 0 | 0 | 1 | 1 | 1 |
| LIMS3L | 0 | 6 | 0 | 0 | 6 | 6 |
| LINS | 0 | 8 | 0 | 0 | 8 | 8 |
| LIPC | 0 | 0 | 0 | 3 | 3 | 3 |
| LIPE | 0 | 0 | 0 | 1 | 1 | 1 |
| LIPJ | 14 | 0 | 0 | 0 | 14 | 0 |
| LIPT1 | 0 | 0 | 0 | 2 | 2 | 2 |
| LLGL1 | 0 | 1 | 0 | 0 | 1 | 1 |
| LLGL2 | 1 | 0 | 0 | 0 | 1 | 0 |
| LLPH | 0 | 0 | 0 | 1 | 1 | 1 |
| LMAN2 | 0 | 0 | 0 | 1 | 1 | 1 |
| LMBRD1 | 0 | 0 | 0 | 4 | 4 | 4 |
| LMBRD2 | 0 | 1 | 0 | 0 | 1 | 1 |
| LMF1 | 0 | 0 | 0 | 1 | 1 | 1 |
| LMNB1 | 1 | 0 | 0 | 0 | 1 | 0 |
| LMO7 | 7 | 0 | 0 | 4 | 11 | 4 |
| LMTK3 | 0 | 0 | 0 | 1 | 1 | 1 |
| LNX1 | 0 | 1 | 0 | 0 | 1 | 1 |
| LOC100132247 | 0 | 0 | 0 | 1 | 1 | 1 |
| LOC100293516 | 0 | 1 | 0 | 0 | 1 | 1 |
| LOC100507203 | 0 | 0 | 0 | 1 | 1 | 1 |
| LOC100509575 | 0 | 6 | 0 | 0 | 6 | 6 |
| LOC120824 | 0 | 0 | 0 | 1 | 1 | 1 |
| LOC388946 | 0 | 0 | 0 | 1 | 1 | 1 |
| LOC399939 | 2 | 0 | 0 | 0 | 2 | 0 |
| LOC440563 | 0 | 1 | 0 | 0 | 1 | 1 |
| LOC649330 | 0 | 0 | 0 | 3 | 3 | 3 |
| LOC729020 | 0 | 3 | 0 | 0 | 3 | 3 |
| LOH12CR1 | 0 | 0 | 0 | 1 | 1 | 1 |
| LONP2 | 1 | 3 | 0 | 0 | 4 | 3 |
| LOXHD1 | 0 | 0 | 0 | 1 | 1 | 1 |
| LOXL3 | 0 | 0 | 0 | 1 | 1 | 1 |
| LOXL4 | 0 | 0 | 0 | 4 | 4 | 4 |
| LPA | 4 | 3 | 0 | 1 | 8 | 4 |
| LPHN1 | 0 | 0 | 0 | 1 | 1 | 1 |
| LPHN2 | 0 | 0 | 0 | 1 | 1 | 1 |
| LPIN1 | 4 | 0 | 0 | 0 | 4 | 0 |
| LPIN2 | 0 | 0 | 0 | 1 | 1 | 1 |
| LPIN3 | 0 | 1 | 0 | 1 | 2 | 2 |
| LPO | 0 | 0 | 0 | 2 | 2 | 2 |
| LRCH3 | 0 | 2 | 0 | 0 | 2 | 2 |
| LRCH4 | 0 | 0 | 0 | 3 | 3 | 3 |
| LRGUK | 2 | 0 | 0 | 0 | 2 | 0 |
| LRIF1 | 0 | 0 | 0 | 1 | 1 | 1 |
| LRIG1 | 0 | 7 | 0 | 1 | 8 | 8 |
| LRMP | 0 | 0 | 0 | 1 | 1 | 1 |
| LRP1 | 0 | 0 | 0 | 7 | 7 | 7 |
| LRP1B | 11 | 1 | 0 | 0 | 12 | 1 |
| LRP2 | 0 | 0 | 0 | 4 | 4 | 4 |
| LRP5 | 0 | 1 | 0 | 0 | 1 | 1 |
| LRP5L | 0 | 2 | 0 | 0 | 2 | 2 |
| LRP8 | 0 | 0 | 0 | 1 | 1 | 1 |
| LRR1 | 0 | 1 | 0 | 1 | 2 | 2 |
| LRRC10 | 0 | 0 | 0 | 1 | 1 | 1 |
| LRRC16B | 4 | 1 | 0 | 1 | 6 | 2 |
| LRRC19 | 0 | 0 | 0 | 1 | 1 | 1 |
| LRRC3 | 0 | 2 | 0 | 0 | 2 | 2 |
| LRRC30 | 0 | 0 | 0 | 1 | 1 | 1 |
| LRRC31 | 1 | 0 | 0 | 1 | 2 | 1 |
| LRRC34 | 0 | 2 | 0 | 2 | 4 | 4 |
| LRRC37A | 0 | 0 | 0 | 1 | 1 | 1 |
| LRRC37A3 | 0 | 0 | 0 | 1 | 1 | 1 |
| LRRC39 | 0 | 1 | 0 | 0 | 1 | 1 |
| LRRC42 | 1 | 0 | 0 | 0 | 1 | 0 |
| LRRC46 | 3 | 0 | 0 | 2 | 5 | 2 |
| LRRC48 | 0 | 0 | 0 | 1 | 1 | 1 |
| LRRC52 | 0 | 1 | 0 | 0 | 1 | 1 |
| LRRC55 | 0 | 1 | 0 | 0 | 1 | 1 |
| LRRC56 | 0 | 0 | 0 | 1 | 1 | 1 |
| LRRC69 | 0 | 0 | 0 | 3 | 3 | 3 |
| LRRC70 | 0 | 1 | 0 | 0 | 1 | 1 |
| LRRC71 | 1 | 0 | 0 | 0 | 1 | 0 |
| LRRC8A | 0 | 1 | 0 | 0 | 1 | 1 |
| LRRC8B | 0 | 0 | 0 | 1 | 1 | 1 |
| LRRC8C | 0 | 1 | 0 | 0 | 1 | 1 |
| LRRC8E | 0 | 0 | 0 | 2 | 2 | 2 |
| LRRCC1 | 0 | 0 | 1 | 2 | 3 | 2 |
| LRRFIP2 | 0 | 0 | 0 | 1 | 1 | 1 |
| LRRIQ1 | 0 | 2 | 0 | 1 | 3 | 3 |
| LRRIQ3 | 2 | 0 | 0 | 0 | 2 | 0 |
| LRRN2 | 0 | 5 | 0 | 0 | 5 | 5 |
| LRTM1 | 0 | 2 | 0 | 1 | 3 | 3 |
| LSM14A | 0 | 0 | 0 | 2 | 2 | 2 |
| LSR | 0 | 0 | 0 | 1 | 1 | 1 |
| LTBP2 | 0 | 3 | 0 | 0 | 3 | 3 |
| LTF | 0 | 0 | 0 | 1 | 1 | 1 |
| LTK | 0 | 5 | 0 | 2 | 7 | 7 |
| LUM | 0 | 0 | 0 | 1 | 1 | 1 |
| LUZP4 | 0 | 0 | 0 | 1 | 1 | 1 |
| LY6G5C | 0 | 0 | 0 | 1 | 1 | 1 |
| LY75 | 1 | 0 | 0 | 0 | 1 | 0 |
| LY75-CD302 | 0 | 2 | 0 | 2 | 4 | 4 |
| LYPD3 | 1 | 0 | 0 | 0 | 1 | 0 |
| LYRM2 | 0 | 1 | 0 | 0 | 1 | 1 |
| LYSMD1 | 1 | 0 | 0 | 0 | 1 | 0 |
| LYST | 0 | 7 | 0 | 0 | 7 | 7 |
| LYVE1 | 0 | 1 | 0 | 0 | 1 | 1 |
| LZTR1 | 1 | 0 | 0 | 0 | 1 | 0 |
| LZTS2 | 0 | 0 | 0 | 1 | 1 | 1 |
| MAB21L3 | 0 | 1 | 0 | 1 | 2 | 2 |
| MACF1 | 0 | 2 | 0 | 2 | 4 | 4 |
| MAD2L2 | 0 | 0 | 0 | 2 | 2 | 2 |
| MADD | 5 | 1 | 0 | 0 | 6 | 1 |
| MAGEA10 | 0 | 0 | 0 | 1 | 1 | 1 |
| MAGEA11 | 0 | 0 | 0 | 2 | 2 | 2 |
| MAGEA3 | 0 | 2 | 0 | 0 | 2 | 2 |
| MAGEA4 | 0 | 0 | 0 | 1 | 1 | 1 |
| MAGEB16 | 0 | 0 | 0 | 1 | 1 | 1 |
| MAGEC1 | 0 | 2 | 0 | 0 | 2 | 2 |
| MAGEC3 | 0 | 1 | 0 | 3 | 4 | 4 |
| MAGEE2 | 0 | 0 | 0 | 2 | 2 | 2 |
| MAGEF1 | 0 | 0 | 0 | 2 | 2 | 2 |
| MAK16 | 0 | 0 | 0 | 4 | 4 | 4 |
| MAMDC2 | 1 | 0 | 0 | 0 | 1 | 0 |
| MAMSTR | 0 | 0 | 0 | 3 | 3 | 3 |
| MAN1B1 | 0 | 0 | 0 | 1 | 1 | 1 |
| MAN2A2 | 1 | 0 | 0 | 2 | 3 | 2 |
| MAN2B1 | 1 | 0 | 0 | 0 | 1 | 0 |
| MAN2B2 | 0 | 0 | 0 | 4 | 4 | 4 |
| MAN2C1 | 0 | 0 | 0 | 1 | 1 | 1 |
| MAP1B | 0 | 0 | 0 | 1 | 1 | 1 |
| MAP3K1 | 0 | 0 | 0 | 1 | 1 | 1 |
| MAP3K11 | 0 | 0 | 0 | 2 | 2 | 2 |
| MAP3K12 | 0 | 0 | 0 | 1 | 1 | 1 |
| MAP3K15 | 0 | 7 | 0 | 1 | 8 | 8 |
| MAP3K3 | 0 | 2 | 0 | 0 | 2 | 2 |
| MAP3K5 | 5 | 1 | 0 | 9 | 15 | 10 |
| MAP3K6 | 1 | 4 | 0 | 0 | 5 | 4 |
| MAP4K2 | 0 | 0 | 0 | 1 | 1 | 1 |
| MAP7 | 0 | 0 | 0 | 1 | 1 | 1 |
| MAP7D1 | 0 | 0 | 0 | 1 | 1 | 1 |
| MAP7D3 | 0 | 2 | 0 | 0 | 2 | 2 |
| MAPK1 | 0 | 0 | 0 | 1 | 1 | 1 |
| MAPK13 | 0 | 1 | 0 | 0 | 1 | 1 |
| MAPK15 | 1 | 0 | 0 | 2 | 3 | 2 |
| MAPK1IP1L | 0 | 0 | 0 | 4 | 4 | 4 |
| MAPKAP1 | 0 | 0 | 0 | 1 | 1 | 1 |
| MAPKAPK5 | 0 | 6 | 0 | 0 | 6 | 6 |
| MAPKBP1 | 0 | 1 | 0 | 0 | 1 | 1 |
| MAPRE3 | 0 | 0 | 0 | 4 | 4 | 4 |
| Mar-04 | 0 | 0 | 0 | 1 | 1 | 1 |
| Mar-10 | 0 | 2 | 0 | 0 | 2 | 2 |
| MARCO | 0 | 1 | 0 | 0 | 1 | 1 |
| MARK3 | 0 | 3 | 0 | 0 | 3 | 3 |
| MARS2 | 0 | 0 | 0 | 1 | 1 | 1 |
| MASP2 | 2 | 0 | 0 | 0 | 2 | 0 |
| MAST1 | 2 | 0 | 0 | 0 | 2 | 0 |
| MAST2 | 0 | 0 | 0 | 3 | 3 | 3 |
| MAST3 | 0 | 0 | 0 | 2 | 2 | 2 |
| MAST4 | 0 | 0 | 0 | 3 | 3 | 3 |
| MASTL | 1 | 0 | 0 | 0 | 1 | 0 |
| MATK | 0 | 2 | 0 | 0 | 2 | 2 |
| MATN4 | 0 | 2 | 0 | 0 | 2 | 2 |
| MATR3 | 5 | 0 | 0 | 0 | 5 | 0 |
| MAU2 | 0 | 0 | 0 | 1 | 1 | 1 |
| MB21D2 | 0 | 0 | 0 | 2 | 2 | 2 |
| MBD3 | 0 | 0 | 0 | 1 | 1 | 1 |
| MBD4 | 1 | 0 | 0 | 3 | 4 | 3 |
| MBNL3 | 0 | 0 | 2 | 0 | 2 | 0 |
| MC1R | 0 | 0 | 0 | 1 | 1 | 1 |
| MC5R | 0 | 0 | 0 | 1 | 1 | 1 |
| MCAM | 0 | 0 | 0 | 1 | 1 | 1 |
| MCC | 0 | 1 | 0 | 1 | 2 | 2 |
| MCCC1 | 0 | 0 | 0 | 1 | 1 | 1 |
| MCEE | 0 | 1 | 0 | 0 | 1 | 1 |
| MCF2 | 0 | 0 | 0 | 1 | 1 | 1 |
| MCF2L | 0 | 0 | 0 | 2 | 2 | 2 |
| MCF2L2 | 0 | 2 | 0 | 0 | 2 | 2 |
| MCHR1 | 0 | 0 | 0 | 1 | 1 | 1 |
| MCM2 | 0 | 0 | 0 | 1 | 1 | 1 |
| MCM3 | 0 | 4 | 0 | 0 | 4 | 4 |
| MCM3AP | 0 | 0 | 0 | 2 | 2 | 2 |
| MCM5 | 7 | 0 | 0 | 1 | 8 | 1 |
| MCM8 | 0 | 0 | 0 | 1 | 1 | 1 |
| MCM9 | 0 | 0 | 0 | 1 | 1 | 1 |
| MCOLN1 | 1 | 0 | 0 | 0 | 1 | 0 |
| MCTP1 | 0 | 10 | 0 | 0 | 10 | 10 |
| MCTP2 | 0 | 1 | 0 | 0 | 1 | 1 |
| MCU | 0 | 0 | 1 | 0 | 1 | 0 |
| MDC1 | 0 | 0 | 0 | 4 | 4 | 4 |
| MDH1 | 0 | 0 | 0 | 1 | 1 | 1 |
| MDN1 | 0 | 0 | 0 | 1 | 1 | 1 |
| ME1 | 5 | 0 | 0 | 0 | 5 | 0 |
| ME2 | 0 | 1 | 0 | 0 | 1 | 1 |
| MECP2 | 0 | 0 | 0 | 1 | 1 | 1 |
| MED12 | 2 | 0 | 0 | 3 | 5 | 3 |
| MED12L | 0 | 0 | 0 | 4 | 4 | 4 |
| MED13 | 0 | 0 | 0 | 1 | 1 | 1 |
| MED13L | 0 | 1 | 0 | 0 | 1 | 1 |
| MED17 | 0 | 1 | 0 | 1 | 2 | 2 |
| MED22 | 0 | 0 | 0 | 1 | 1 | 1 |
| MED23 | 0 | 5 | 0 | 0 | 5 | 5 |
| MED25 | 0 | 0 | 0 | 1 | 1 | 1 |
| MED31 | 0 | 0 | 0 | 1 | 1 | 1 |
| MED4 | 0 | 1 | 0 | 0 | 1 | 1 |
| MEF2B | 0 | 0 | 0 | 1 | 1 | 1 |
| MEGF10 | 7 | 1 | 0 | 0 | 8 | 1 |
| MEGF8 | 0 | 0 | 0 | 1 | 1 | 1 |
| MEI1 | 0 | 12 | 0 | 0 | 12 | 12 |
| MEOX1 | 2 | 1 | 0 | 0 | 3 | 1 |
| MEOX2 | 0 | 0 | 0 | 2 | 2 | 2 |
| MEP1B | 0 | 3 | 0 | 0 | 3 | 3 |
| MEPE | 0 | 0 | 0 | 5 | 5 | 5 |
| MET | 0 | 0 | 0 | 6 | 6 | 6 |
| METAP1D | 1 | 0 | 0 | 0 | 1 | 0 |
| METTL1 | 8 | 0 | 0 | 0 | 8 | 0 |
| METTL13 | 0 | 1 | 0 | 0 | 1 | 1 |
| METTL17 | 0 | 1 | 0 | 0 | 1 | 1 |
| METTL18 | 0 | 0 | 0 | 1 | 1 | 1 |
| METTL20 | 2 | 1 | 0 | 0 | 3 | 1 |
| METTL21A | 0 | 0 | 1 | 0 | 1 | 0 |
| METTL2A | 0 | 3 | 0 | 0 | 3 | 3 |
| METTL3 | 0 | 1 | 0 | 0 | 1 | 1 |
| METTL7A | 0 | 0 | 0 | 1 | 1 | 1 |
| METTL7B | 0 | 2 | 0 | 0 | 2 | 2 |
| MFHAS1 | 0 | 3 | 0 | 1 | 4 | 4 |
| MFN1 | 0 | 0 | 0 | 1 | 1 | 1 |
| MFNG | 1 | 0 | 0 | 2 | 3 | 2 |
| MFSD4 | 1 | 0 | 0 | 0 | 1 | 0 |
| MFSD5 | 0 | 7 | 0 | 1 | 8 | 8 |
| MFSD6 | 0 | 1 | 0 | 0 | 1 | 1 |
| MFSD6L | 0 | 1 | 0 | 2 | 3 | 3 |
| MFSD9 | 0 | 1 | 0 | 0 | 1 | 1 |
| MGAM | 0 | 0 | 0 | 1 | 1 | 1 |
| MGAT4A | 0 | 0 | 0 | 1 | 1 | 1 |
| MGST2 | 0 | 2 | 0 | 0 | 2 | 2 |
| MIA2 | 0 | 0 | 0 | 1 | 1 | 1 |
| MIA3 | 0 | 0 | 0 | 2 | 2 | 2 |
| MIB1 | 0 | 0 | 0 | 1 | 1 | 1 |
| MICA | 13 | 0 | 0 | 26 | 39 | 26 |
| MICAL2 | 16 | 0 | 0 | 0 | 16 | 0 |
| MICALCL | 0 | 0 | 0 | 2 | 2 | 2 |
| MICB | 0 | 2 | 0 | 1 | 3 | 3 |
| MID1 | 9 | 0 | 0 | 1 | 10 | 1 |
| MID1IP1 | 0 | 0 | 0 | 3 | 3 | 3 |
| MIEN1 | 0 | 0 | 0 | 1 | 1 | 1 |
| MIF4GD | 0 | 1 | 0 | 0 | 1 | 1 |
| MILR1 | 0 | 0 | 0 | 1 | 1 | 1 |
| MINK1 | 0 | 1 | 0 | 2 | 3 | 3 |
| MIS18BP1 | 0 | 0 | 0 | 4 | 4 | 4 |
| MKI67 | 0 | 1 | 0 | 1 | 2 | 2 |
| MKI67IP | 0 | 0 | 0 | 4 | 4 | 4 |
| MKNK1 | 0 | 0 | 0 | 1 | 1 | 1 |
| MKNK2 | 0 | 0 | 0 | 1 | 1 | 1 |
| MKRN2 | 0 | 0 | 0 | 2 | 2 | 2 |
| MLF1 | 0 | 0 | 0 | 1 | 1 | 1 |
| MLF2 | 0 | 0 | 1 | 0 | 1 | 0 |
| MLH1 | 0 | 0 | 0 | 6 | 6 | 6 |
| MLH3 | 0 | 0 | 0 | 1 | 1 | 1 |
| MLKL | 0 | 1 | 0 | 5 | 6 | 6 |
| MLL | 15 | 2 | 0 | 1 | 18 | 3 |
| MLL2 | 0 | 0 | 0 | 1 | 1 | 1 |
| MLL3 | 1 | 0 | 0 | 0 | 1 | 0 |
| MLL4 | 0 | 0 | 0 | 14 | 14 | 14 |
| MLLT10 | 0 | 0 | 0 | 1 | 1 | 1 |
| MLLT6 | 0 | 0 | 0 | 1 | 1 | 1 |
| MLNR | 0 | 0 | 0 | 7 | 7 | 7 |
| MLPH | 0 | 0 | 0 | 2 | 2 | 2 |
| MMAA | 0 | 3 | 0 | 1 | 4 | 4 |
| MMAB | 0 | 1 | 0 | 0 | 1 | 1 |
| MMACHC | 0 | 0 | 0 | 2 | 2 | 2 |
| MME | 1 | 1 | 0 | 13 | 15 | 14 |
| MMEL1 | 0 | 1 | 0 | 0 | 1 | 1 |
| MMP1 | 1 | 1 | 0 | 14 | 16 | 15 |
| MMP10 | 17 | 2 | 0 | 0 | 19 | 2 |
| MMP12 | 0 | 0 | 0 | 9 | 9 | 9 |
| MMP15 | 0 | 3 | 0 | 0 | 3 | 3 |
| MMP19 | 1 | 1 | 0 | 0 | 2 | 1 |
| MMP20 | 1 | 0 | 0 | 1 | 2 | 1 |
| MMP27 | 0 | 2 | 0 | 0 | 2 | 2 |
| MMP3 | 0 | 0 | 0 | 1 | 1 | 1 |
| MMP8 | 2 | 7 | 0 | 0 | 9 | 7 |
| MMRN1 | 0 | 1 | 0 | 0 | 1 | 1 |
| MMS19 | 1 | 0 | 0 | 1 | 2 | 1 |
| MMS22L | 0 | 0 | 0 | 14 | 14 | 14 |
| MNDA | 0 | 1 | 0 | 0 | 1 | 1 |
| MNS1 | 0 | 0 | 0 | 11 | 11 | 11 |
| MOB1B | 0 | 0 | 0 | 1 | 1 | 1 |
| MOCOS | 1 | 0 | 0 | 2 | 3 | 2 |
| MOCS1 | 0 | 0 | 0 | 1 | 1 | 1 |
| MOCS2 | 0 | 1 | 0 | 0 | 1 | 1 |
| MOGAT1 | 1 | 0 | 0 | 14 | 15 | 14 |
| MOGAT2 | 0 | 0 | 0 | 1 | 1 | 1 |
| MOGS | 0 | 0 | 0 | 2 | 2 | 2 |
| MOK | 0 | 18 | 0 | 1 | 19 | 19 |
| MON1B | 1 | 0 | 0 | 0 | 1 | 0 |
| MON2 | 1 | 0 | 0 | 0 | 1 | 0 |
| MORN3 | 0 | 0 | 0 | 1 | 1 | 1 |
| MOS | 0 | 1 | 0 | 0 | 1 | 1 |
| MOSC1 | 0 | 1 | 0 | 0 | 1 | 1 |
| MOV10 | 2 | 0 | 0 | 3 | 5 | 3 |
| MPHOSPH9 | 0 | 0 | 0 | 2 | 2 | 2 |
| MPL | 1 | 0 | 0 | 0 | 1 | 0 |
| MPO | 2 | 0 | 0 | 0 | 2 | 0 |
| MPP1 | 2 | 3 | 0 | 0 | 5 | 3 |
| MPPE1 | 0 | 0 | 0 | 2 | 2 | 2 |
| MPV17L2 | 0 | 0 | 0 | 1 | 1 | 1 |
| MPZL2 | 0 | 0 | 0 | 1 | 1 | 1 |
| MPZL3 | 0 | 0 | 0 | 1 | 1 | 1 |
| MRAS | 2 | 0 | 0 | 0 | 2 | 0 |
| MRC2 | 0 | 0 | 0 | 1 | 1 | 1 |
| MRGPRX3 | 0 | 8 | 0 | 0 | 8 | 8 |
| MRGPRX4 | 0 | 0 | 0 | 1 | 1 | 1 |
| MRM1 | 0 | 1 | 0 | 0 | 1 | 1 |
| MRO | 0 | 0 | 0 | 9 | 9 | 9 |
| MRPL1 | 0 | 1 | 0 | 0 | 1 | 1 |
| MRPL14 | 0 | 0 | 0 | 1 | 1 | 1 |
| MRPL2 | 0 | 0 | 0 | 1 | 1 | 1 |
| MRPL20 | 1 | 0 | 0 | 0 | 1 | 0 |
| MRPL28 | 0 | 1 | 0 | 0 | 1 | 1 |
| MRPL32 | 0 | 2 | 0 | 0 | 2 | 2 |
| MRPL46 | 0 | 1 | 0 | 1 | 2 | 2 |
| MRPL50 | 0 | 0 | 0 | 1 | 1 | 1 |
| MRPS18C | 1 | 0 | 0 | 0 | 1 | 0 |
| MRPS2 | 0 | 1 | 0 | 0 | 1 | 1 |
| MRPS27 | 0 | 0 | 13 | 0 | 13 | 0 |
| MRPS33 | 0 | 1 | 0 | 0 | 1 | 1 |
| MRPS7 | 0 | 0 | 0 | 1 | 1 | 1 |
| MRTO4 | 0 | 0 | 0 | 1 | 1 | 1 |
| MS4A12 | 3 | 0 | 0 | 3 | 6 | 3 |
| MS4A14 | 0 | 3 | 0 | 0 | 3 | 3 |
| MS4A2 | 0 | 1 | 0 | 0 | 1 | 1 |
| MS4A4A | 0 | 0 | 0 | 1 | 1 | 1 |
| MS4A5 | 0 | 1 | 0 | 0 | 1 | 1 |
| MS4A6A | 2 | 0 | 0 | 3 | 5 | 3 |
| MSH4 | 0 | 1 | 0 | 0 | 1 | 1 |
| MSH6 | 0 | 0 | 0 | 3 | 3 | 3 |
| MSL2 | 0 | 1 | 0 | 0 | 1 | 1 |
| MSLNL | 0 | 1 | 0 | 1 | 2 | 2 |
| MSMO1 | 0 | 2 | 0 | 0 | 2 | 2 |
| MSR1 | 0 | 9 | 0 | 0 | 9 | 9 |
| MSRB3 | 1 | 0 | 0 | 0 | 1 | 0 |
| MST1 | 3 | 10 | 0 | 0 | 13 | 10 |
| MST1R | 0 | 0 | 0 | 2 | 2 | 2 |
| MT1X | 1 | 0 | 0 | 0 | 1 | 0 |
| MTA2 | 0 | 16 | 0 | 0 | 16 | 16 |
| MTCP1 | 0 | 1 | 0 | 0 | 1 | 1 |
| MTERF | 0 | 1 | 0 | 0 | 1 | 1 |
| MTERFD3 | 0 | 0 | 0 | 1 | 1 | 1 |
| MTF2 | 1 | 0 | 0 | 0 | 1 | 0 |
| MTHFD1 | 0 | 1 | 0 | 0 | 1 | 1 |
| MTHFD1L | 0 | 1 | 0 | 0 | 1 | 1 |
| MTHFR | 0 | 1 | 0 | 2 | 3 | 3 |
| MTIF2 | 0 | 0 | 0 | 2 | 2 | 2 |
| MTL5 | 0 | 1 | 0 | 0 | 1 | 1 |
| MTMR10 | 0 | 1 | 0 | 0 | 1 | 1 |
| MTMR11 | 0 | 0 | 0 | 1 | 1 | 1 |
| MTOR | 0 | 1 | 0 | 0 | 1 | 1 |
| MTRR | 1 | 0 | 0 | 0 | 1 | 0 |
| MTUS1 | 0 | 0 | 0 | 1 | 1 | 1 |
| MUC1 | 0 | 0 | 0 | 1 | 1 | 1 |
| MUC16 | 1 | 2 | 0 | 18 | 21 | 20 |
| MUC17 | 1 | 5 | 0 | 3 | 9 | 8 |
| MUC2 | 0 | 0 | 0 | 4 | 4 | 4 |
| MUC20 | 17 | 0 | 0 | 0 | 17 | 0 |
| MUC4 | 0 | 0 | 0 | 1 | 1 | 1 |
| MUC5B | 0 | 0 | 0 | 4 | 4 | 4 |
| MUC6 | 0 | 0 | 0 | 23 | 23 | 23 |
| MUC7 | 0 | 0 | 0 | 1 | 1 | 1 |
| MUL1 | 0 | 0 | 0 | 1 | 1 | 1 |
| MUM1 | 0 | 0 | 0 | 1 | 1 | 1 |
| MUS81 | 0 | 0 | 0 | 1 | 1 | 1 |
| MUSK | 0 | 1 | 0 | 0 | 1 | 1 |
| MVD | 0 | 0 | 0 | 1 | 1 | 1 |
| MVP | 0 | 0 | 0 | 1 | 1 | 1 |
| MX2 | 0 | 0 | 0 | 1 | 1 | 1 |
| MXD3 | 0 | 0 | 0 | 1 | 1 | 1 |
| MXI1 | 0 | 1 | 0 | 0 | 1 | 1 |
| MXRA5 | 0 | 0 | 0 | 1 | 1 | 1 |
| MYBBP1A | 0 | 0 | 0 | 3 | 3 | 3 |
| MYBPC2 | 0 | 1 | 0 | 2 | 3 | 3 |
| MYBPHL | 0 | 2 | 0 | 0 | 2 | 2 |
| MYCBP2 | 0 | 0 | 0 | 4 | 4 | 4 |
| MYCBPAP | 0 | 0 | 0 | 11 | 11 | 11 |
| MYEF2 | 6 | 0 | 0 | 0 | 6 | 0 |
| MYEOV | 1 | 0 | 0 | 1 | 2 | 1 |
| MYH1 | 0 | 1 | 0 | 1 | 2 | 2 |
| MYH10 | 0 | 1 | 0 | 1 | 2 | 2 |
| MYH11 | 1 | 0 | 0 | 0 | 1 | 0 |
| MYH13 | 0 | 0 | 0 | 2 | 2 | 2 |
| MYH15 | 1 | 6 | 0 | 0 | 7 | 6 |
| MYH3 | 0 | 10 | 0 | 1 | 11 | 11 |
| MYH4 | 1 | 0 | 0 | 2 | 3 | 2 |
| MYH7 | 0 | 1 | 0 | 1 | 2 | 2 |
| MYH7B | 0 | 1 | 0 | 2 | 3 | 3 |
| MYH8 | 0 | 3 | 0 | 1 | 4 | 4 |
| MYH9 | 0 | 17 | 0 | 2 | 19 | 19 |
| MYL1 | 0 | 0 | 0 | 6 | 6 | 6 |
| MYL7 | 0 | 1 | 0 | 0 | 1 | 1 |
| MYLK | 0 | 1 | 0 | 0 | 1 | 1 |
| MYNN | 0 | 2 | 0 | 0 | 2 | 2 |
| MYO10 | 0 | 0 | 0 | 2 | 2 | 2 |
| MYO15A | 0 | 1 | 0 | 0 | 1 | 1 |
| MYO16 | 0 | 1 | 0 | 0 | 1 | 1 |
| MYO1A | 0 | 1 | 0 | 0 | 1 | 1 |
| MYO1C | 0 | 1 | 0 | 0 | 1 | 1 |
| MYO1F | 0 | 1 | 0 | 0 | 1 | 1 |
| MYO1H | 1 | 0 | 0 | 1 | 2 | 1 |
| MYO3B | 1 | 1 | 0 | 0 | 2 | 1 |
| MYO6 | 1 | 0 | 0 | 1 | 2 | 1 |
| MYO7A | 0 | 0 | 0 | 3 | 3 | 3 |
| MYO7B | 1 | 0 | 0 | 2 | 3 | 2 |
| MYO9B | 0 | 0 | 0 | 1 | 1 | 1 |
| MYOC | 0 | 2 | 0 | 0 | 2 | 2 |
| MYOCD | 0 | 0 | 0 | 1 | 1 | 1 |
| MYOF | 0 | 4 | 0 | 0 | 4 | 4 |
| MYOM2 | 0 | 0 | 0 | 1 | 1 | 1 |
| MYOM3 | 0 | 0 | 0 | 2 | 2 | 2 |
| MYT1 | 0 | 1 | 0 | 4 | 5 | 5 |
| MYT1L | 0 | 3 | 0 | 0 | 3 | 3 |
| MZB1 | 0 | 0 | 0 | 2 | 2 | 2 |
| N4BP2L2 | 0 | 0 | 0 | 1 | 1 | 1 |
| N4BP3 | 0 | 0 | 0 | 3 | 3 | 3 |
| N6AMT1 | 1 | 0 | 0 | 0 | 1 | 0 |
| NAA16 | 0 | 3 | 0 | 17 | 20 | 20 |
| NAA20 | 0 | 2 | 0 | 0 | 2 | 2 |
| NAAA | 1 | 0 | 0 | 0 | 1 | 0 |
| NAALADL2 | 0 | 1 | 0 | 0 | 1 | 1 |
| NACA2 | 0 | 1 | 0 | 1 | 2 | 2 |
| NACC2 | 0 | 0 | 0 | 1 | 1 | 1 |
| NADK | 0 | 0 | 0 | 1 | 1 | 1 |
| NAF1 | 0 | 1 | 0 | 0 | 1 | 1 |
| NAGLU | 0 | 1 | 0 | 1 | 2 | 2 |
| NAIP | 0 | 0 | 0 | 1 | 1 | 1 |
| NANOG | 0 | 0 | 0 | 5 | 5 | 5 |
| NAP1L1 | 2 | 0 | 0 | 0 | 2 | 0 |
| NAP1L4 | 0 | 2 | 0 | 0 | 2 | 2 |
| NARF | 0 | 0 | 0 | 1 | 1 | 1 |
| NAT1 | 0 | 2 | 0 | 0 | 2 | 2 |
| NAT10 | 1 | 0 | 0 | 3 | 4 | 3 |
| NAT2 | 0 | 1 | 0 | 0 | 1 | 1 |
| NAT6 | 0 | 0 | 0 | 1 | 1 | 1 |
| NAT8B | 0 | 0 | 0 | 1 | 1 | 1 |
| NBAS | 0 | 2 | 0 | 1 | 3 | 3 |
| NBEAL1 | 1 | 0 | 0 | 0 | 1 | 0 |
| NBN | 0 | 0 | 0 | 1 | 1 | 1 |
| NBPF1 | 0 | 9 | 0 | 0 | 9 | 9 |
| NBPF10 | 0 | 1 | 0 | 0 | 1 | 1 |
| NBPF14 | 4 | 0 | 0 | 2 | 6 | 2 |
| NBPF15 | 0 | 1 | 0 | 2 | 3 | 3 |
| NBPF3 | 0 | 0 | 0 | 38 | 38 | 38 |
| NBPF7 | 3 | 1 | 0 | 1 | 5 | 2 |
| NBPF9 | 0 | 1 | 10 | 1 | 12 | 2 |
| NCAM1 | 0 | 0 | 0 | 1 | 1 | 1 |
| NCAPG2 | 0 | 0 | 0 | 1 | 1 | 1 |
| NCAPH | 0 | 0 | 0 | 1 | 1 | 1 |
| NCBP1 | 0 | 4 | 0 | 0 | 4 | 4 |
| NCF1 | 0 | 1 | 0 | 0 | 1 | 1 |
| NCKIPSD | 0 | 0 | 0 | 1 | 1 | 1 |
| NCL | 0 | 0 | 0 | 29 | 29 | 29 |
| NCOA3 | 0 | 1 | 0 | 0 | 1 | 1 |
| NCOA6 | 0 | 0 | 0 | 1 | 1 | 1 |
| NCOR1 | 2 | 13 | 0 | 2 | 17 | 15 |
| NCOR2 | 0 | 0 | 0 | 5 | 5 | 5 |
| NCR1 | 0 | 0 | 0 | 1 | 1 | 1 |
| NCS1 | 1 | 0 | 0 | 0 | 1 | 0 |
| NDRG2 | 0 | 0 | 0 | 1 | 1 | 1 |
| NDUFA4L2 | 0 | 0 | 0 | 2 | 2 | 2 |
| NDUFA8 | 0 | 0 | 0 | 2 | 2 | 2 |
| NDUFAF3 | 0 | 0 | 0 | 1 | 1 | 1 |
| NDUFAF4 | 0 | 1 | 2 | 0 | 3 | 1 |
| NDUFB10 | 0 | 0 | 0 | 1 | 1 | 1 |
| NDUFB11 | 0 | 0 | 0 | 2 | 2 | 2 |
| NDUFB3 | 0 | 1 | 0 | 0 | 1 | 1 |
| NDUFS2 | 0 | 0 | 0 | 1 | 1 | 1 |
| NDUFV3 | 1 | 0 | 0 | 1 | 2 | 1 |
| NEB | 0 | 1 | 0 | 1 | 2 | 2 |
| NECAB2 | 0 | 6 | 0 | 1 | 7 | 7 |
| NECAB3 | 0 | 0 | 0 | 1 | 1 | 1 |
| NEDD8 | 0 | 0 | 0 | 2 | 2 | 2 |
| NEFH | 0 | 0 | 0 | 1 | 1 | 1 |
| NEFM | 0 | 0 | 0 | 1 | 1 | 1 |
| NEIL1 | 5 | 0 | 0 | 0 | 5 | 0 |
| NEIL3 | 0 | 0 | 0 | 1 | 1 | 1 |
| NEK10 | 0 | 1 | 0 | 0 | 1 | 1 |
| NEK11 | 1 | 0 | 0 | 0 | 1 | 0 |
| NEK8 | 0 | 0 | 1 | 0 | 1 | 0 |
| NEK9 | 1 | 2 | 0 | 0 | 3 | 2 |
| NENF | 0 | 1 | 0 | 0 | 1 | 1 |
| NEO1 | 0 | 0 | 0 | 1 | 1 | 1 |
| NES | 0 | 2 | 0 | 1 | 3 | 3 |
| NEU3 | 0 | 0 | 1 | 0 | 1 | 0 |
| NEURL | 0 | 0 | 0 | 8 | 8 | 8 |
| NEURL2 | 0 | 0 | 0 | 1 | 1 | 1 |
| NEURL4 | 0 | 0 | 0 | 1 | 1 | 1 |
| NEUROD1 | 0 | 0 | 0 | 1 | 1 | 1 |
| NEUROD6 | 0 | 1 | 0 | 0 | 1 | 1 |
| NEUROG3 | 0 | 1 | 0 | 0 | 1 | 1 |
| NEXN | 1 | 0 | 0 | 1 | 2 | 1 |
| NF1 | 0 | 0 | 0 | 1 | 1 | 1 |
| NFATC3 | 0 | 0 | 0 | 14 | 14 | 14 |
| NFKBIZ | 0 | 0 | 0 | 1 | 1 | 1 |
| NFXL1 | 1 | 0 | 0 | 0 | 1 | 0 |
| NGDN | 0 | 1 | 0 | 0 | 1 | 1 |
| NGEF | 0 | 0 | 0 | 1 | 1 | 1 |
| NGLY1 | 0 | 1 | 0 | 0 | 1 | 1 |
| NHLRC1 | 0 | 0 | 0 | 1 | 1 | 1 |
| NID2 | 0 | 0 | 0 | 1 | 1 | 1 |
| NIF3L1 | 0 | 0 | 0 | 2 | 2 | 2 |
| NIN | 0 | 7 | 0 | 0 | 7 | 7 |
| NINL | 0 | 0 | 0 | 1 | 1 | 1 |
| NIPA2 | 0 | 0 | 0 | 4 | 4 | 4 |
| NIPBL | 2 | 0 | 0 | 10 | 12 | 10 |
| NIPSNAP1 | 0 | 0 | 0 | 1 | 1 | 1 |
| NIPSNAP3A | 0 | 0 | 0 | 2 | 2 | 2 |
| NISCH | 0 | 0 | 0 | 4 | 4 | 4 |
| NKAPL | 0 | 0 | 0 | 3 | 3 | 3 |
| NKTR | 0 | 0 | 0 | 5 | 5 | 5 |
| NKX1-2 | 0 | 0 | 0 | 3 | 3 | 3 |
| NLGN4X | 0 | 1 | 0 | 0 | 1 | 1 |
| NLRC4 | 0 | 1 | 0 | 0 | 1 | 1 |
| NLRP10 | 0 | 1 | 0 | 0 | 1 | 1 |
| NLRP12 | 0 | 2 | 0 | 0 | 2 | 2 |
| NLRP13 | 0 | 0 | 0 | 14 | 14 | 14 |
| NLRP5 | 0 | 0 | 0 | 1 | 1 | 1 |
| NLRP8 | 0 | 1 | 0 | 3 | 4 | 4 |
| NLRX1 | 0 | 0 | 0 | 1 | 1 | 1 |
| NME1 | 0 | 0 | 0 | 1 | 1 | 1 |
| NMRAL1 | 0 | 1 | 0 | 1 | 2 | 2 |
| NOBOX | 0 | 0 | 0 | 1 | 1 | 1 |
| NOC2L | 0 | 0 | 0 | 2 | 2 | 2 |
| NOC3L | 0 | 1 | 0 | 1 | 2 | 2 |
| NOD2 | 0 | 0 | 0 | 15 | 15 | 15 |
| NOL10 | 0 | 3 | 0 | 1 | 4 | 4 |
| NOL3 | 1 | 0 | 0 | 2 | 3 | 2 |
| NOL6 | 0 | 2 | 0 | 0 | 2 | 2 |
| NOMO2 | 0 | 0 | 0 | 7 | 7 | 7 |
| NOMO3 | 0 | 1 | 0 | 1 | 2 | 2 |
| NONO | 0 | 0 | 0 | 1 | 1 | 1 |
| NOP14 | 0 | 0 | 0 | 2 | 2 | 2 |
| NOP58 | 0 | 1 | 0 | 0 | 1 | 1 |
| NOS1 | 0 | 3 | 0 | 0 | 3 | 3 |
| NOS2 | 0 | 0 | 0 | 1 | 1 | 1 |
| NOSTRIN | 0 | 0 | 0 | 1 | 1 | 1 |
| NOTCH2 | 1 | 1 | 0 | 1 | 3 | 2 |
| NOTCH2NL | 0 | 3 | 0 | 0 | 3 | 3 |
| NOX3 | 1 | 1 | 0 | 1 | 3 | 2 |
| NOX4 | 0 | 0 | 0 | 1 | 1 | 1 |
| NOX5 | 1 | 2 | 0 | 2 | 5 | 4 |
| NPAS1 | 0 | 1 | 0 | 0 | 1 | 1 |
| NPAT | 0 | 9 | 0 | 1 | 10 | 10 |
| NPC1 | 0 | 1 | 0 | 0 | 1 | 1 |
| NPC1L1 | 1 | 0 | 0 | 0 | 1 | 0 |
| NPC2 | 5 | 0 | 0 | 0 | 5 | 0 |
| NPFFR1 | 6 | 0 | 0 | 0 | 6 | 0 |
| NPFFR2 | 0 | 0 | 0 | 1 | 1 | 1 |
| NPHP1 | 4 | 2 | 0 | 0 | 6 | 2 |
| NPHP3 | 0 | 1 | 0 | 1 | 2 | 2 |
| NPHS2 | 0 | 0 | 0 | 1 | 1 | 1 |
| NPIP | 0 | 0 | 0 | 1 | 1 | 1 |
| NPM2 | 2 | 0 | 0 | 2 | 4 | 2 |
| NPR1 | 0 | 0 | 0 | 15 | 15 | 15 |
| NPVF | 0 | 0 | 0 | 1 | 1 | 1 |
| NPY2R | 0 | 1 | 0 | 0 | 1 | 1 |
| NQO1 | 1 | 0 | 0 | 2 | 3 | 2 |
| NR2E3 | 1 | 0 | 0 | 1 | 2 | 1 |
| NR2F1 | 0 | 0 | 0 | 1 | 1 | 1 |
| NR3C1 | 0 | 1 | 0 | 0 | 1 | 1 |
| NR4A2 | 0 | 0 | 0 | 2 | 2 | 2 |
| NRAP | 1 | 0 | 0 | 0 | 1 | 0 |
| NRF1 | 0 | 0 | 0 | 1 | 1 | 1 |
| NRG4 | 0 | 0 | 0 | 1 | 1 | 1 |
| NRIP1 | 0 | 1 | 0 | 0 | 1 | 1 |
| NRK | 0 | 2 | 0 | 3 | 5 | 5 |
| NRP1 | 0 | 0 | 0 | 1 | 1 | 1 |
| NRP2 | 0 | 1 | 0 | 0 | 1 | 1 |
| NRSN1 | 0 | 2 | 0 | 0 | 2 | 2 |
| NRXN1 | 0 | 0 | 0 | 1 | 1 | 1 |
| NRXN2 | 11 | 0 | 0 | 8 | 19 | 8 |
| NSD1 | 4 | 0 | 0 | 1 | 5 | 1 |
| NSF | 0 | 0 | 0 | 3 | 3 | 3 |
| NSMCE2 | 0 | 0 | 0 | 1 | 1 | 1 |
| NSUN2 | 0 | 0 | 0 | 1 | 1 | 1 |
| NSUN4 | 0 | 1 | 0 | 0 | 1 | 1 |
| NSUN6 | 0 | 0 | 0 | 2 | 2 | 2 |
| NT5C | 0 | 1 | 0 | 0 | 1 | 1 |
| NT5C1B | 0 | 11 | 0 | 0 | 11 | 11 |
| NT5DC2 | 0 | 0 | 0 | 1 | 1 | 1 |
| NTM | 0 | 0 | 0 | 1 | 1 | 1 |
| NTPCR | 4 | 0 | 0 | 0 | 4 | 0 |
| NTRK1 | 0 | 0 | 0 | 1 | 1 | 1 |
| NUAK2 | 1 | 0 | 0 | 0 | 1 | 0 |
| NUB1 | 0 | 0 | 0 | 6 | 6 | 6 |
| NUBP1 | 1 | 0 | 0 | 0 | 1 | 0 |
| NUCB1 | 0 | 0 | 0 | 1 | 1 | 1 |
| NUDT12 | 0 | 1 | 0 | 1 | 2 | 2 |
| NUDT13 | 0 | 0 | 0 | 13 | 13 | 13 |
| NUDT16L1 | 0 | 0 | 4 | 0 | 4 | 0 |
| NUDT17 | 0 | 0 | 0 | 35 | 35 | 35 |
| NUDT18 | 0 | 0 | 1 | 0 | 1 | 0 |
| NUDT22 | 0 | 0 | 0 | 1 | 1 | 1 |
| NUDT6 | 0 | 0 | 0 | 1 | 1 | 1 |
| NUF2 | 3 | 0 | 0 | 0 | 3 | 0 |
| NUMBL | 0 | 0 | 0 | 1 | 1 | 1 |
| NUP107 | 1 | 0 | 0 | 0 | 1 | 0 |
| NUP133 | 0 | 0 | 0 | 1 | 1 | 1 |
| NUP155 | 1 | 0 | 0 | 0 | 1 | 0 |
| NUP210 | 1 | 1 | 0 | 0 | 2 | 1 |
| NUP210L | 0 | 0 | 0 | 1 | 1 | 1 |
| NUP214 | 0 | 0 | 0 | 11 | 11 | 11 |
| NUP98 | 0 | 0 | 0 | 2 | 2 | 2 |
| NWD1 | 0 | 2 | 0 | 0 | 2 | 2 |
| NXF3 | 1 | 0 | 0 | 1 | 2 | 1 |
| OAS1 | 0 | 0 | 0 | 1 | 1 | 1 |
| OAS2 | 0 | 1 | 0 | 0 | 1 | 1 |
| OAS3 | 0 | 7 | 0 | 0 | 7 | 7 |
| OASL | 0 | 3 | 0 | 0 | 3 | 3 |
| OAZ1 | 0 | 0 | 0 | 1 | 1 | 1 |
| OAZ2 | 0 | 0 | 0 | 1 | 1 | 1 |
| OBFC2B | 0 | 2 | 0 | 0 | 2 | 2 |
| OBP2B | 0 | 0 | 0 | 1 | 1 | 1 |
| OBSCN | 1 | 2 | 0 | 0 | 3 | 2 |
| OBSL1 | 0 | 3 | 0 | 0 | 3 | 3 |
| OC90 | 0 | 0 | 0 | 1 | 1 | 1 |
| OCIAD2 | 1 | 0 | 0 | 0 | 1 | 0 |
| OCM | 0 | 3 | 0 | 0 | 3 | 3 |
| ODAM | 0 | 0 | 0 | 1 | 1 | 1 |
| ODF1 | 0 | 0 | 0 | 1 | 1 | 1 |
| ODF3L1 | 0 | 2 | 0 | 1 | 3 | 3 |
| ODF3L2 | 0 | 2 | 0 | 1 | 3 | 3 |
| ODZ1 | 0 | 0 | 0 | 13 | 13 | 13 |
| ODZ2 | 0 | 0 | 0 | 1 | 1 | 1 |
| ODZ4 | 0 | 0 | 0 | 1 | 1 | 1 |
| OFD1 | 0 | 7 | 0 | 0 | 7 | 7 |
| OGFOD1 | 0 | 0 | 0 | 1 | 1 | 1 |
| OGFRL1 | 0 | 0 | 0 | 2 | 2 | 2 |
| OGG1 | 0 | 1 | 0 | 1 | 2 | 2 |
| OIT3 | 0 | 0 | 0 | 14 | 14 | 14 |
| OLFM4 | 0 | 6 | 0 | 10 | 16 | 16 |
| OLFML2A | 0 | 0 | 0 | 1 | 1 | 1 |
| OLFML3 | 0 | 1 | 0 | 2 | 3 | 3 |
| OLR1 | 0 | 1 | 0 | 0 | 1 | 1 |
| OMP | 0 | 1 | 0 | 0 | 1 | 1 |
| OPA1 | 0 | 1 | 0 | 0 | 1 | 1 |
| OPN3 | 18 | 0 | 0 | 0 | 18 | 0 |
| OPN4 | 0 | 0 | 0 | 1 | 1 | 1 |
| OPRD1 | 0 | 0 | 0 | 4 | 4 | 4 |
| OPRK1 | 0 | 1 | 0 | 0 | 1 | 1 |
| OPTC | 0 | 0 | 0 | 1 | 1 | 1 |
| OPTN | 1 | 0 | 0 | 0 | 1 | 0 |
| OR10A4 | 0 | 0 | 0 | 1 | 1 | 1 |
| OR10A6 | 0 | 5 | 0 | 0 | 5 | 5 |
| OR10A7 | 0 | 0 | 0 | 1 | 1 | 1 |
| OR10AD1 | 0 | 1 | 0 | 4 | 5 | 5 |
| OR10C1 | 0 | 11 | 0 | 0 | 11 | 11 |
| OR10G2 | 0 | 0 | 0 | 1 | 1 | 1 |
| OR10G7 | 0 | 1 | 0 | 0 | 1 | 1 |
| OR10H4 | 0 | 1 | 0 | 1 | 2 | 2 |
| OR10J3 | 0 | 0 | 0 | 1 | 1 | 1 |
| OR10J5 | 0 | 0 | 18 | 0 | 18 | 0 |
| OR10P1 | 0 | 0 | 0 | 4 | 4 | 4 |
| OR10Q1 | 0 | 0 | 0 | 1 | 1 | 1 |
| OR10R2 | 0 | 4 | 0 | 0 | 4 | 4 |
| OR10S1 | 0 | 0 | 0 | 1 | 1 | 1 |
| OR10V1 | 0 | 17 | 0 | 0 | 17 | 17 |
| OR10Z1 | 0 | 1 | 0 | 0 | 1 | 1 |
| OR11G2 | 0 | 0 | 0 | 1 | 1 | 1 |
| OR11H1 | 0 | 0 | 0 | 2 | 2 | 2 |
| OR11H12 | 0 | 0 | 0 | 1 | 1 | 1 |
| OR11H2 | 0 | 0 | 0 | 1 | 1 | 1 |
| OR11H4 | 0 | 0 | 0 | 1 | 1 | 1 |
| OR11H6 | 0 | 0 | 0 | 2 | 2 | 2 |
| OR13A1 | 0 | 1 | 0 | 2 | 3 | 3 |
| OR13C4 | 0 | 0 | 0 | 1 | 1 | 1 |
| OR13C5 | 0 | 1 | 0 | 18 | 19 | 19 |
| OR13C8 | 0 | 1 | 0 | 0 | 1 | 1 |
| OR13C9 | 0 | 0 | 0 | 4 | 4 | 4 |
| OR13D1 | 0 | 1 | 0 | 1 | 2 | 2 |
| OR13G1 | 0 | 0 | 0 | 1 | 1 | 1 |
| OR13J1 | 0 | 0 | 0 | 1 | 1 | 1 |
| OR14C36 | 0 | 1 | 0 | 0 | 1 | 1 |
| OR1A2 | 0 | 0 | 0 | 1 | 1 | 1 |
| OR1D5 | 0 | 0 | 0 | 3 | 3 | 3 |
| OR1E2 | 0 | 0 | 0 | 10 | 10 | 10 |
| OR1F1 | 0 | 11 | 0 | 0 | 11 | 11 |
| OR1J1 | 0 | 11 | 0 | 1 | 12 | 12 |
| OR1J2 | 0 | 1 | 0 | 2 | 3 | 3 |
| OR1N2 | 0 | 0 | 0 | 11 | 11 | 11 |
| OR1S1 | 0 | 1 | 0 | 1 | 2 | 2 |
| OR1S2 | 0 | 10 | 0 | 1 | 11 | 11 |
| OR2A2 | 0 | 0 | 0 | 1 | 1 | 1 |
| OR2A5 | 0 | 1 | 0 | 0 | 1 | 1 |
| OR2AG2 | 0 | 0 | 0 | 1 | 1 | 1 |
| OR2B11 | 0 | 5 | 0 | 3 | 8 | 8 |
| OR2B2 | 0 | 0 | 0 | 1 | 1 | 1 |
| OR2C3 | 0 | 0 | 0 | 1 | 1 | 1 |
| OR2D2 | 0 | 2 | 0 | 4 | 6 | 6 |
| OR2F1 | 0 | 0 | 0 | 1 | 1 | 1 |
| OR2G6 | 0 | 0 | 0 | 7 | 7 | 7 |
| OR2L2 | 0 | 2 | 0 | 0 | 2 | 2 |
| OR2L3 | 0 | 2 | 0 | 0 | 2 | 2 |
| OR2M3 | 0 | 0 | 1 | 0 | 1 | 0 |
| OR2S2 | 0 | 1 | 0 | 0 | 1 | 1 |
| OR2T1 | 0 | 2 | 0 | 1 | 3 | 3 |
| OR2T10 | 0 | 1 | 0 | 0 | 1 | 1 |
| OR2T11 | 0 | 2 | 0 | 2 | 4 | 4 |
| OR2T12 | 0 | 1 | 0 | 1 | 2 | 2 |
| OR2T2 | 0 | 0 | 0 | 6 | 6 | 6 |
| OR2T27 | 0 | 5 | 1 | 10 | 16 | 15 |
| OR2T3 | 0 | 1 | 0 | 1 | 2 | 2 |
| OR2T35 | 0 | 1 | 0 | 15 | 16 | 16 |
| OR2T4 | 0 | 0 | 0 | 4 | 4 | 4 |
| OR2T5 | 0 | 6 | 0 | 0 | 6 | 6 |
| OR2T8 | 0 | 0 | 0 | 3 | 3 | 3 |
| OR2W3 | 0 | 8 | 0 | 0 | 8 | 8 |
| OR2W5 | 0 | 2 | 0 | 0 | 2 | 2 |
| OR3A1 | 0 | 5 | 0 | 1 | 6 | 6 |
| OR4A5 | 0 | 2 | 0 | 1 | 3 | 3 |
| OR4C15 | 0 | 3 | 0 | 0 | 3 | 3 |
| OR4C16 | 0 | 0 | 0 | 2 | 2 | 2 |
| OR4C3 | 0 | 0 | 0 | 1 | 1 | 1 |
| OR4C46 | 0 | 0 | 0 | 4 | 4 | 4 |
| OR4D1 | 0 | 0 | 5 | 3 | 8 | 3 |
| OR4D10 | 0 | 20 | 0 | 0 | 20 | 20 |
| OR4D11 | 0 | 0 | 0 | 1 | 1 | 1 |
| OR4D6 | 0 | 0 | 0 | 10 | 10 | 10 |
| OR4F17 | 0 | 0 | 0 | 4 | 4 | 4 |
| OR4F21 | 0 | 1 | 0 | 0 | 1 | 1 |
| OR4F4 | 0 | 0 | 0 | 1 | 1 | 1 |
| OR4F6 | 0 | 0 | 0 | 2 | 2 | 2 |
| OR4L1 | 0 | 0 | 0 | 4 | 4 | 4 |
| OR4M2 | 0 | 10 | 0 | 2 | 12 | 12 |
| OR4N2 | 0 | 0 | 0 | 4 | 4 | 4 |
| OR4N4 | 0 | 2 | 0 | 9 | 11 | 11 |
| OR4Q3 | 0 | 3 | 0 | 0 | 3 | 3 |
| OR4S2 | 0 | 2 | 0 | 0 | 2 | 2 |
| OR51B2 | 0 | 0 | 0 | 1 | 1 | 1 |
| OR51B5 | 0 | 1 | 0 | 0 | 1 | 1 |
| OR51F1 | 0 | 1 | 0 | 0 | 1 | 1 |
| OR51G1 | 0 | 2 | 0 | 8 | 10 | 10 |
| OR51I1 | 0 | 16 | 0 | 0 | 16 | 16 |
| OR51I2 | 0 | 0 | 0 | 13 | 13 | 13 |
| OR51M1 | 0 | 1 | 0 | 0 | 1 | 1 |
| OR51S1 | 0 | 1 | 0 | 0 | 1 | 1 |
| OR51T1 | 0 | 0 | 0 | 4 | 4 | 4 |
| OR51V1 | 0 | 0 | 0 | 2 | 2 | 2 |
| OR52A1 | 0 | 0 | 0 | 1 | 1 | 1 |
| OR52B2 | 0 | 0 | 0 | 3 | 3 | 3 |
| OR52E6 | 0 | 0 | 0 | 1 | 1 | 1 |
| OR52E8 | 0 | 1 | 0 | 0 | 1 | 1 |
| OR52H1 | 0 | 1 | 0 | 0 | 1 | 1 |
| OR52I2 | 0 | 0 | 0 | 5 | 5 | 5 |
| OR52J3 | 0 | 6 | 0 | 0 | 6 | 6 |
| OR52K1 | 0 | 2 | 0 | 2 | 4 | 4 |
| OR52K2 | 0 | 20 | 0 | 2 | 22 | 22 |
| OR52M1 | 0 | 0 | 0 | 4 | 4 | 4 |
| OR52N1 | 0 | 0 | 0 | 1 | 1 | 1 |
| OR52N2 | 0 | 0 | 0 | 1 | 1 | 1 |
| OR52N4 | 0 | 1 | 0 | 0 | 1 | 1 |
| OR52N5 | 0 | 0 | 0 | 1 | 1 | 1 |
| OR52W1 | 0 | 0 | 0 | 1 | 1 | 1 |
| OR56A3 | 0 | 1 | 0 | 0 | 1 | 1 |
| OR56B1 | 0 | 0 | 0 | 1 | 1 | 1 |
| OR5A2 | 0 | 1 | 0 | 0 | 1 | 1 |
| OR5AC2 | 0 | 1 | 0 | 9 | 10 | 10 |
| OR5AN1 | 0 | 0 | 0 | 1 | 1 | 1 |
| OR5AR1 | 0 | 0 | 0 | 1 | 1 | 1 |
| OR5B12 | 0 | 0 | 0 | 3 | 3 | 3 |
| OR5B17 | 0 | 3 | 0 | 0 | 3 | 3 |
| OR5D14 | 0 | 0 | 0 | 1 | 1 | 1 |
| OR5D16 | 0 | 0 | 0 | 5 | 5 | 5 |
| OR5H1 | 0 | 1 | 0 | 1 | 2 | 2 |
| OR5H14 | 0 | 1 | 0 | 0 | 1 | 1 |
| OR5H15 | 0 | 4 | 0 | 11 | 15 | 15 |
| OR5H2 | 0 | 0 | 0 | 1 | 1 | 1 |
| OR5L2 | 0 | 1 | 0 | 2 | 3 | 3 |
| OR5M3 | 0 | 7 | 0 | 0 | 7 | 7 |
| OR5M8 | 0 | 0 | 0 | 1 | 1 | 1 |
| OR5P2 | 0 | 0 | 0 | 5 | 5 | 5 |
| OR5R1 | 0 | 0 | 0 | 1 | 1 | 1 |
| OR6A2 | 0 | 0 | 0 | 2 | 2 | 2 |
| OR6C1 | 0 | 0 | 0 | 17 | 17 | 17 |
| OR6C3 | 0 | 0 | 0 | 1 | 1 | 1 |
| OR6C4 | 0 | 1 | 0 | 9 | 10 | 10 |
| OR6C6 | 0 | 2 | 0 | 0 | 2 | 2 |
| OR6C68 | 0 | 0 | 0 | 2 | 2 | 2 |
| OR6C70 | 0 | 0 | 0 | 3 | 3 | 3 |
| OR6C76 | 0 | 0 | 0 | 4 | 4 | 4 |
| OR6F1 | 0 | 0 | 0 | 2 | 2 | 2 |
| OR6K2 | 0 | 0 | 0 | 5 | 5 | 5 |
| OR6Q1 | 0 | 0 | 0 | 1 | 1 | 1 |
| OR7C1 | 0 | 0 | 0 | 1 | 1 | 1 |
| OR7G1 | 0 | 0 | 0 | 6 | 6 | 6 |
| OR7G3 | 0 | 7 | 0 | 0 | 7 | 7 |
| OR8A1 | 0 | 0 | 0 | 2 | 2 | 2 |
| OR8B3 | 0 | 0 | 0 | 15 | 15 | 15 |
| OR8B4 | 0 | 0 | 0 | 1 | 1 | 1 |
| OR8D4 | 0 | 1 | 0 | 2 | 3 | 3 |
| OR8G1 | 0 | 0 | 0 | 4 | 4 | 4 |
| OR8H2 | 0 | 0 | 0 | 1 | 1 | 1 |
| OR8H3 | 0 | 0 | 0 | 1 | 1 | 1 |
| OR8K3 | 0 | 11 | 0 | 0 | 11 | 11 |
| OR8U1 | 0 | 0 | 0 | 1 | 1 | 1 |
| OR8U8 | 0 | 1 | 0 | 0 | 1 | 1 |
| OR9A4 | 0 | 0 | 0 | 1 | 1 | 1 |
| OR9K2 | 0 | 1 | 0 | 10 | 11 | 11 |
| OR9Q1 | 0 | 0 | 0 | 1 | 1 | 1 |
| ORAI2 | 0 | 1 | 0 | 0 | 1 | 1 |
| ORC1 | 0 | 0 | 0 | 1 | 1 | 1 |
| ORC4 | 2 | 0 | 0 | 0 | 2 | 0 |
| OSBP | 0 | 15 | 0 | 0 | 15 | 15 |
| OSBPL10 | 0 | 0 | 0 | 3 | 3 | 3 |
| OSBPL1A | 0 | 2 | 0 | 1 | 3 | 3 |
| OSBPL7 | 0 | 0 | 0 | 1 | 1 | 1 |
| OSGEP | 0 | 1 | 0 | 0 | 1 | 1 |
| OTOA | 1 | 0 | 0 | 0 | 1 | 0 |
| OTOF | 0 | 0 | 0 | 2 | 2 | 2 |
| OTOGL | 1 | 16 | 0 | 0 | 17 | 16 |
| OTOL1 | 1 | 0 | 0 | 0 | 1 | 0 |
| OTOP2 | 0 | 0 | 0 | 1 | 1 | 1 |
| OTOP3 | 0 | 1 | 0 | 0 | 1 | 1 |
| OTUD4 | 4 | 0 | 0 | 0 | 4 | 0 |
| OTUD6B | 1 | 0 | 0 | 0 | 1 | 0 |
| OVCH1 | 0 | 0 | 0 | 1 | 1 | 1 |
| OVGP1 | 0 | 0 | 0 | 19 | 19 | 19 |
| OVOL2 | 2 | 1 | 0 | 0 | 3 | 1 |
| OXA1L | 0 | 0 | 0 | 1 | 1 | 1 |
| OXGR1 | 0 | 0 | 0 | 3 | 3 | 3 |
| OXSM | 0 | 0 | 6 | 0 | 6 | 0 |
| P2RX2 | 0 | 0 | 0 | 1 | 1 | 1 |
| P2RX5 | 0 | 2 | 0 | 0 | 2 | 2 |
| P2RX6 | 0 | 3 | 0 | 0 | 3 | 3 |
| P2RX7 | 4 | 0 | 0 | 0 | 4 | 0 |
| P2RY11 | 0 | 1 | 0 | 1 | 2 | 2 |
| P2RY13 | 0 | 0 | 0 | 2 | 2 | 2 |
| P2RY4 | 0 | 16 | 0 | 0 | 16 | 16 |
| P4HA2 | 0 | 0 | 0 | 1 | 1 | 1 |
| PABPC1 | 0 | 2 | 0 | 6 | 8 | 8 |
| PABPC3 | 0 | 0 | 0 | 34 | 34 | 34 |
| PADI1 | 1 | 0 | 0 | 0 | 1 | 0 |
| PADI2 | 1 | 2 | 0 | 0 | 3 | 2 |
| PADI4 | 0 | 0 | 0 | 1 | 1 | 1 |
| PADI6 | 0 | 0 | 0 | 1 | 1 | 1 |
| PAF1 | 0 | 3 | 0 | 0 | 3 | 3 |
| PAFAH1B1 | 1 | 0 | 0 | 0 | 1 | 0 |
| PAFAH2 | 0 | 0 | 0 | 2 | 2 | 2 |
| PAH | 2 | 0 | 0 | 0 | 2 | 0 |
| PALB2 | 0 | 1 | 0 | 3 | 4 | 4 |
| PALLD | 0 | 0 | 0 | 1 | 1 | 1 |
| PANX1 | 0 | 1 | 0 | 0 | 1 | 1 |
| PANX3 | 0 | 0 | 0 | 1 | 1 | 1 |
| PAPD7 | 0 | 0 | 0 | 1 | 1 | 1 |
| PAPLN | 3 | 1 | 0 | 1 | 5 | 2 |
| PAPOLB | 0 | 0 | 0 | 1 | 1 | 1 |
| PAPPA2 | 0 | 0 | 0 | 1 | 1 | 1 |
| PAPSS1 | 0 | 1 | 0 | 0 | 1 | 1 |
| PAQR6 | 0 | 0 | 0 | 1 | 1 | 1 |
| PARD3 | 2 | 1 | 0 | 0 | 3 | 1 |
| PARD6B | 0 | 0 | 0 | 1 | 1 | 1 |
| PARK7 | 0 | 0 | 0 | 2 | 2 | 2 |
| PARP1 | 0 | 0 | 0 | 1 | 1 | 1 |
| PARP11 | 0 | 0 | 0 | 1 | 1 | 1 |
| PARP16 | 0 | 1 | 0 | 0 | 1 | 1 |
| PARP2 | 0 | 1 | 0 | 0 | 1 | 1 |
| PARP3 | 0 | 2 | 0 | 0 | 2 | 2 |
| PARP4 | 3 | 0 | 0 | 1 | 4 | 1 |
| PARP8 | 0 | 0 | 0 | 1 | 1 | 1 |
| PASK | 0 | 1 | 0 | 1 | 2 | 2 |
| PATE1 | 0 | 1 | 0 | 0 | 1 | 1 |
| PATE4 | 16 | 0 | 0 | 0 | 16 | 0 |
| PATZ1 | 0 | 0 | 0 | 10 | 10 | 10 |
| PAX6 | 0 | 0 | 0 | 1 | 1 | 1 |
| PAX8 | 0 | 0 | 0 | 1 | 1 | 1 |
| PBLD | 0 | 0 | 0 | 6 | 6 | 6 |
| PBX3 | 0 | 0 | 0 | 1 | 1 | 1 |
| PBX4 | 0 | 1 | 0 | 0 | 1 | 1 |
| PCCA | 0 | 0 | 0 | 1 | 1 | 1 |
| PCDH15 | 1 | 1 | 0 | 0 | 2 | 1 |
| PCDH20 | 0 | 0 | 0 | 1 | 1 | 1 |
| PCDHA11 | 0 | 0 | 0 | 2 | 2 | 2 |
| PCDHA12 | 0 | 2 | 0 | 0 | 2 | 2 |
| PCDHA13 | 0 | 0 | 0 | 4 | 4 | 4 |
| PCDHA2 | 0 | 0 | 0 | 1 | 1 | 1 |
| PCDHA3 | 0 | 0 | 0 | 12 | 12 | 12 |
| PCDHA4 | 0 | 0 | 0 | 1 | 1 | 1 |
| PCDHA9 | 0 | 1 | 0 | 0 | 1 | 1 |
| PCDHAC1 | 0 | 1 | 0 | 0 | 1 | 1 |
| PCDHB1 | 0 | 1 | 0 | 0 | 1 | 1 |
| PCDHB10 | 0 | 1 | 0 | 2 | 3 | 3 |
| PCDHB11 | 0 | 0 | 0 | 1 | 1 | 1 |
| PCDHB13 | 0 | 0 | 0 | 1 | 1 | 1 |
| PCDHB14 | 0 | 1 | 0 | 2 | 3 | 3 |
| PCDHB16 | 0 | 0 | 0 | 1 | 1 | 1 |
| PCDHB3 | 0 | 0 | 0 | 3 | 3 | 3 |
| PCDHB4 | 0 | 0 | 0 | 3 | 3 | 3 |
| PCDHB6 | 0 | 0 | 0 | 7 | 7 | 7 |
| PCDHB7 | 0 | 9 | 0 | 1 | 10 | 10 |
| PCDHB8 | 0 | 0 | 0 | 1 | 1 | 1 |
| PCDHB9 | 0 | 0 | 0 | 1 | 1 | 1 |
| PCDHGA11 | 0 | 0 | 0 | 8 | 8 | 8 |
| PCDHGA2 | 0 | 1 | 0 | 1 | 2 | 2 |
| PCDHGA3 | 0 | 1 | 0 | 1 | 2 | 2 |
| PCDHGA5 | 0 | 0 | 0 | 1 | 1 | 1 |
| PCDHGB1 | 0 | 0 | 0 | 1 | 1 | 1 |
| PCDHGB3 | 0 | 0 | 0 | 2 | 2 | 2 |
| PCDHGB6 | 0 | 0 | 0 | 1 | 1 | 1 |
| PCDHGC5 | 0 | 1 | 0 | 1 | 2 | 2 |
| PCGF1 | 0 | 0 | 0 | 9 | 9 | 9 |
| PCIF1 | 0 | 0 | 0 | 1 | 1 | 1 |
| PCK1 | 0 | 0 | 0 | 1 | 1 | 1 |
| PCK2 | 1 | 4 | 0 | 3 | 8 | 7 |
| PCNT | 0 | 1 | 0 | 2 | 3 | 3 |
| PCNX | 0 | 0 | 0 | 1 | 1 | 1 |
| PCNXL2 | 0 | 0 | 0 | 1 | 1 | 1 |
| PCOLCE | 0 | 0 | 0 | 2 | 2 | 2 |
| PCOLCE2 | 0 | 1 | 0 | 0 | 1 | 1 |
| PCSK1 | 0 | 1 | 0 | 0 | 1 | 1 |
| PCSK4 | 0 | 1 | 0 | 0 | 1 | 1 |
| PCSK5 | 0 | 0 | 0 | 1 | 1 | 1 |
| PDAP1 | 0 | 0 | 0 | 8 | 8 | 8 |
| PDCD11 | 2 | 1 | 0 | 0 | 3 | 1 |
| PDCD1LG2 | 0 | 0 | 0 | 4 | 4 | 4 |
| PDCD2L | 1 | 0 | 0 | 0 | 1 | 0 |
| PDCD5 | 0 | 1 | 0 | 0 | 1 | 1 |
| PDCD6IP | 1 | 0 | 0 | 1 | 2 | 1 |
| PDE11A | 1 | 6 | 0 | 3 | 10 | 9 |
| PDE3B | 0 | 4 | 0 | 0 | 4 | 4 |
| PDE4B | 0 | 1 | 0 | 1 | 2 | 2 |
| PDE4DIP | 1 | 1 | 0 | 1 | 3 | 2 |
| PDE5A | 0 | 3 | 0 | 0 | 3 | 3 |
| PDE6B | 0 | 0 | 0 | 1 | 1 | 1 |
| PDGFRB | 0 | 1 | 0 | 1 | 2 | 2 |
| PDIA2 | 1 | 2 | 0 | 0 | 3 | 2 |
| PDIA6 | 0 | 0 | 0 | 1 | 1 | 1 |
| PDLIM2 | 0 | 0 | 0 | 1 | 1 | 1 |
| PDP2 | 0 | 0 | 0 | 1 | 1 | 1 |
| PDPR | 0 | 1 | 0 | 0 | 1 | 1 |
| PDSS2 | 0 | 0 | 0 | 14 | 14 | 14 |
| PDX1 | 0 | 1 | 0 | 0 | 1 | 1 |
| PDXDC1 | 0 | 0 | 0 | 1 | 1 | 1 |
| PDZD2 | 0 | 0 | 0 | 4 | 4 | 4 |
| PDZD3 | 0 | 0 | 0 | 1 | 1 | 1 |
| PDZD7 | 0 | 0 | 0 | 2 | 2 | 2 |
| PDZD9 | 0 | 0 | 0 | 2 | 2 | 2 |
| PDZK1 | 0 | 0 | 0 | 1 | 1 | 1 |
| PDZRN3 | 2 | 0 | 0 | 1 | 3 | 1 |
| PEG3 | 0 | 0 | 0 | 1 | 1 | 1 |
| PELI1 | 0 | 0 | 0 | 1 | 1 | 1 |
| PELP1 | 0 | 0 | 0 | 1 | 1 | 1 |
| PENK | 0 | 0 | 0 | 2 | 2 | 2 |
| PER1 | 0 | 1 | 0 | 1 | 2 | 2 |
| PER2 | 0 | 2 | 0 | 1 | 3 | 3 |
| PER3 | 6 | 0 | 0 | 0 | 6 | 0 |
| PET112 | 0 | 1 | 0 | 0 | 1 | 1 |
| PEX1 | 0 | 0 | 0 | 4 | 4 | 4 |
| PEX11G | 0 | 1 | 0 | 0 | 1 | 1 |
| PEX12 | 0 | 0 | 0 | 1 | 1 | 1 |
| PEX6 | 0 | 0 | 0 | 1 | 1 | 1 |
| PFAS | 0 | 1 | 0 | 1 | 2 | 2 |
| PFKFB1 | 0 | 0 | 0 | 2 | 2 | 2 |
| PFKFB4 | 0 | 0 | 0 | 1 | 1 | 1 |
| PFKP | 0 | 0 | 0 | 1 | 1 | 1 |
| PGD | 1 | 0 | 0 | 0 | 1 | 0 |
| PGK1 | 0 | 0 | 0 | 1 | 1 | 1 |
| PGK2 | 0 | 0 | 0 | 1 | 1 | 1 |
| PGLYRP1 | 0 | 0 | 1 | 0 | 1 | 0 |
| PGLYRP4 | 0 | 1 | 0 | 0 | 1 | 1 |
| PGM2 | 0 | 1 | 1 | 0 | 2 | 1 |
| PGM3 | 0 | 0 | 0 | 1 | 1 | 1 |
| PGPEP1L | 0 | 0 | 0 | 2 | 2 | 2 |
| PGS1 | 0 | 0 | 0 | 1 | 1 | 1 |
| PHACTR4 | 0 | 0 | 0 | 2 | 2 | 2 |
| PHB | 0 | 0 | 0 | 1 | 1 | 1 |
| PHC3 | 0 | 0 | 0 | 15 | 15 | 15 |
| PHF13 | 0 | 0 | 0 | 1 | 1 | 1 |
| PHF15 | 0 | 0 | 0 | 4 | 4 | 4 |
| PHF21A | 0 | 0 | 0 | 5 | 5 | 5 |
| PHF7 | 1 | 0 | 0 | 0 | 1 | 0 |
| PHGDH | 11 | 0 | 0 | 1 | 12 | 1 |
| PHKA1 | 0 | 1 | 0 | 2 | 3 | 3 |
| PHKB | 0 | 1 | 0 | 0 | 1 | 1 |
| PHLDB1 | 0 | 0 | 0 | 1 | 1 | 1 |
| PHLDB2 | 0 | 1 | 0 | 0 | 1 | 1 |
| PHLPP2 | 0 | 0 | 0 | 2 | 2 | 2 |
| PHTF1 | 0 | 15 | 0 | 0 | 15 | 15 |
| PHYHD1 | 0 | 1 | 0 | 0 | 1 | 1 |
| PHYHIP | 0 | 0 | 0 | 1 | 1 | 1 |
| PI15 | 0 | 1 | 0 | 0 | 1 | 1 |
| PI4KA | 5 | 0 | 0 | 0 | 5 | 0 |
| PI4KB | 0 | 0 | 0 | 7 | 7 | 7 |
| PIEZO2 | 0 | 0 | 0 | 2 | 2 | 2 |
| PIGM | 0 | 0 | 0 | 1 | 1 | 1 |
| PIGN | 0 | 1 | 0 | 0 | 1 | 1 |
| PIGU | 0 | 0 | 0 | 3 | 3 | 3 |
| PIK3C2A | 0 | 1 | 0 | 0 | 1 | 1 |
| PIK3C2B | 0 | 0 | 0 | 2 | 2 | 2 |
| PIK3C2G | 6 | 14 | 8 | 6 | 34 | 20 |
| PIK3C3 | 0 | 11 | 0 | 0 | 11 | 11 |
| PIK3CD | 0 | 0 | 0 | 1 | 1 | 1 |
| PIK3R3 | 6 | 0 | 0 | 0 | 6 | 0 |
| PIK3R4 | 0 | 0 | 0 | 1 | 1 | 1 |
| PIK3R6 | 11 | 1 | 0 | 0 | 12 | 1 |
| PIKFYVE | 0 | 1 | 0 | 0 | 1 | 1 |
| PIM2 | 10 | 0 | 0 | 0 | 10 | 0 |
| PION | 0 | 0 | 0 | 1 | 1 | 1 |
| PIP | 0 | 0 | 0 | 1 | 1 | 1 |
| PIR | 0 | 0 | 0 | 1 | 1 | 1 |
| PITRM1 | 0 | 1 | 0 | 0 | 1 | 1 |
| PIWIL2 | 0 | 0 | 0 | 1 | 1 | 1 |
| PIWIL3 | 7 | 2 | 0 | 3 | 12 | 5 |
| PKD1L1 | 1 | 3 | 0 | 1 | 5 | 4 |
| PKD1L2 | 2 | 0 | 3 | 2 | 7 | 2 |
| PKD1L3 | 0 | 15 | 0 | 0 | 15 | 15 |
| PKD2 | 0 | 0 | 0 | 1 | 1 | 1 |
| PKD2L1 | 0 | 9 | 0 | 0 | 9 | 9 |
| PKHD1 | 0 | 6 | 0 | 5 | 11 | 11 |
| PKHD1L1 | 30 | 6 | 0 | 1 | 37 | 7 |
| PKM2 | 1 | 0 | 0 | 0 | 1 | 0 |
| PKN3 | 0 | 0 | 0 | 1 | 1 | 1 |
| PKP1 | 0 | 0 | 0 | 1 | 1 | 1 |
| PKP4 | 2 | 0 | 0 | 0 | 2 | 0 |
| PLA2G2E | 1 | 0 | 0 | 0 | 1 | 0 |
| PLA2G3 | 0 | 0 | 0 | 7 | 7 | 7 |
| PLA2G4C | 0 | 0 | 0 | 9 | 9 | 9 |
| PLA2G4D | 7 | 0 | 0 | 0 | 7 | 0 |
| PLA2G4F | 0 | 1 | 0 | 3 | 4 | 4 |
| PLA2G7 | 0 | 1 | 0 | 0 | 1 | 1 |
| PLA2R1 | 1 | 2 | 0 | 4 | 7 | 6 |
| PLAC1L | 1 | 0 | 0 | 0 | 1 | 0 |
| PLAC4 | 0 | 0 | 0 | 1 | 1 | 1 |
| PLAT | 0 | 0 | 0 | 9 | 9 | 9 |
| PLB1 | 0 | 0 | 0 | 1 | 1 | 1 |
| PLCB2 | 0 | 0 | 0 | 2 | 2 | 2 |
| PLCB3 | 0 | 0 | 0 | 2 | 2 | 2 |
| PLCD3 | 0 | 0 | 0 | 1 | 1 | 1 |
| PLCD4 | 0 | 0 | 0 | 1 | 1 | 1 |
| PLCL1 | 0 | 0 | 0 | 1 | 1 | 1 |
| PLCL2 | 0 | 1 | 0 | 4 | 5 | 5 |
| PLEC | 0 | 0 | 0 | 3 | 3 | 3 |
| PLEK2 | 0 | 1 | 0 | 0 | 1 | 1 |
| PLEKHA1 | 0 | 10 | 0 | 1 | 11 | 11 |
| PLEKHA8 | 0 | 0 | 0 | 1 | 1 | 1 |
| PLEKHB2 | 0 | 1 | 0 | 0 | 1 | 1 |
| PLEKHF1 | 0 | 0 | 0 | 1 | 1 | 1 |
| PLEKHG1 | 0 | 0 | 0 | 1 | 1 | 1 |
| PLEKHG2 | 1 | 0 | 0 | 1 | 2 | 1 |
| PLEKHG4 | 0 | 1 | 0 | 0 | 1 | 1 |
| PLEKHG4B | 0 | 0 | 0 | 1 | 1 | 1 |
| PLEKHG5 | 0 | 0 | 0 | 2 | 2 | 2 |
| PLEKHG6 | 0 | 0 | 0 | 2 | 2 | 2 |
| PLEKHH1 | 0 | 8 | 0 | 1 | 9 | 9 |
| PLEKHH2 | 0 | 1 | 0 | 1 | 2 | 2 |
| PLEKHH3 | 0 | 0 | 0 | 1 | 1 | 1 |
| PLEKHM2 | 0 | 0 | 0 | 4 | 4 | 4 |
| PLIN4 | 0 | 0 | 0 | 4 | 4 | 4 |
| PLK1S1 | 0 | 3 | 0 | 14 | 17 | 17 |
| PLK3 | 0 | 0 | 0 | 2 | 2 | 2 |
| PLOD3 | 7 | 0 | 0 | 1 | 8 | 1 |
| PLS1 | 0 | 1 | 0 | 0 | 1 | 1 |
| PLSCR1 | 0 | 2 | 0 | 0 | 2 | 2 |
| PLSCR5 | 0 | 1 | 0 | 11 | 12 | 12 |
| PLXDC2 | 0 | 2 | 0 | 0 | 2 | 2 |
| PLXNA1 | 0 | 0 | 0 | 1 | 1 | 1 |
| PLXNA2 | 0 | 0 | 0 | 2 | 2 | 2 |
| PLXNB3 | 0 | 0 | 0 | 1 | 1 | 1 |
| PM20D1 | 0 | 0 | 0 | 2 | 2 | 2 |
| PMEL | 0 | 1 | 0 | 3 | 4 | 4 |
| PMF1 | 0 | 1 | 0 | 1 | 2 | 2 |
| PMFBP1 | 0 | 1 | 0 | 1 | 2 | 2 |
| PML | 0 | 1 | 0 | 2 | 3 | 3 |
| PMS1 | 0 | 0 | 0 | 1 | 1 | 1 |
| PMS2 | 0 | 0 | 0 | 1 | 1 | 1 |
| PMVK | 0 | 1 | 0 | 0 | 1 | 1 |
| PNCK | 0 | 1 | 0 | 1 | 2 | 2 |
| PNLDC1 | 0 | 2 | 0 | 0 | 2 | 2 |
| PNLIPRP1 | 0 | 1 | 0 | 1 | 2 | 2 |
| PNLIPRP2 | 0 | 0 | 16 | 4 | 20 | 4 |
| PNLIPRP3 | 0 | 1 | 0 | 1 | 2 | 2 |
| PNMT | 0 | 4 | 0 | 1 | 5 | 5 |
| PNPLA1 | 0 | 12 | 0 | 0 | 12 | 12 |
| PNPLA7 | 0 | 1 | 0 | 3 | 4 | 4 |
| PODXL2 | 0 | 0 | 0 | 1 | 1 | 1 |
| POF1B | 0 | 2 | 0 | 0 | 2 | 2 |
| POFUT1 | 0 | 0 | 0 | 1 | 1 | 1 |
| POLD1 | 0 | 0 | 0 | 1 | 1 | 1 |
| POLDIP2 | 0 | 0 | 0 | 1 | 1 | 1 |
| POLDIP3 | 0 | 0 | 0 | 1 | 1 | 1 |
| POLE | 2 | 0 | 0 | 7 | 9 | 7 |
| POLE2 | 1 | 0 | 0 | 1 | 2 | 1 |
| POLG | 1 | 0 | 0 | 0 | 1 | 0 |
| POLG2 | 0 | 4 | 0 | 4 | 8 | 8 |
| POLI | 0 | 6 | 0 | 0 | 6 | 6 |
| POLK | 0 | 1 | 0 | 2 | 3 | 3 |
| POLN | 0 | 1 | 0 | 3 | 4 | 4 |
| POLQ | 9 | 0 | 0 | 0 | 9 | 0 |
| POLR1E | 0 | 0 | 0 | 1 | 1 | 1 |
| POLR2A | 0 | 0 | 0 | 3 | 3 | 3 |
| POLR2I | 0 | 0 | 3 | 0 | 3 | 0 |
| POLR2J2 | 4 | 0 | 0 | 0 | 4 | 0 |
| POLR3A | 0 | 1 | 0 | 1 | 2 | 2 |
| POLR3C | 0 | 0 | 0 | 1 | 1 | 1 |
| POLR3D | 0 | 0 | 0 | 1 | 1 | 1 |
| POLR3F | 0 | 0 | 0 | 3 | 3 | 3 |
| POLR3G | 0 | 0 | 0 | 1 | 1 | 1 |
| POM121L12 | 0 | 2 | 0 | 0 | 2 | 2 |
| POM121L2 | 0 | 1 | 0 | 0 | 1 | 1 |
| POMGNT1 | 0 | 0 | 0 | 2 | 2 | 2 |
| POMT1 | 1 | 0 | 0 | 1 | 2 | 1 |
| PON2 | 0 | 0 | 0 | 1 | 1 | 1 |
| POP1 | 0 | 0 | 0 | 1 | 1 | 1 |
| POP5 | 0 | 0 | 1 | 0 | 1 | 0 |
| POTEA | 2 | 6 | 0 | 1 | 9 | 7 |
| POTEH | 1 | 7 | 0 | 0 | 8 | 7 |
| POU1F1 | 1 | 0 | 0 | 0 | 1 | 0 |
| POU4F2 | 0 | 0 | 0 | 1 | 1 | 1 |
| POU6F1 | 0 | 0 | 0 | 1 | 1 | 1 |
| POU6F2 | 0 | 1 | 0 | 0 | 1 | 1 |
| PPAN-P2RY11 | 0 | 0 | 0 | 2 | 2 | 2 |
| PPARGC1B | 0 | 0 | 0 | 1 | 1 | 1 |
| PPCDC | 0 | 0 | 0 | 1 | 1 | 1 |
| PPEF2 | 0 | 3 | 0 | 2 | 5 | 5 |
| PPFIA3 | 8 | 0 | 0 | 4 | 12 | 4 |
| PPIC | 0 | 0 | 0 | 10 | 10 | 10 |
| PPID | 0 | 0 | 0 | 1 | 1 | 1 |
| PPIE | 0 | 1 | 0 | 0 | 1 | 1 |
| PPIL2 | 0 | 0 | 0 | 1 | 1 | 1 |
| PPIP5K2 | 0 | 1 | 0 | 0 | 1 | 1 |
| PPL | 0 | 2 | 0 | 0 | 2 | 2 |
| PPM1F | 0 | 0 | 0 | 2 | 2 | 2 |
| PPM1G | 0 | 0 | 0 | 3 | 3 | 3 |
| PPM1J | 1 | 0 | 0 | 1 | 2 | 1 |
| PPM1M | 2 | 0 | 0 | 0 | 2 | 0 |
| PPP1R12A | 0 | 0 | 1 | 0 | 1 | 0 |
| PPP1R13B | 0 | 1 | 0 | 0 | 1 | 1 |
| PPP1R15A | 0 | 2 | 0 | 1 | 3 | 3 |
| PPP1R26 | 0 | 0 | 0 | 3 | 3 | 3 |
| PPP1R32 | 1 | 0 | 0 | 0 | 1 | 0 |
| PPP1R3A | 0 | 0 | 0 | 11 | 11 | 11 |
| PPP1R42 | 1 | 0 | 1 | 0 | 2 | 0 |
| PPP2R3A | 0 | 1 | 0 | 0 | 1 | 1 |
| PPP2R5D | 2 | 0 | 0 | 0 | 2 | 0 |
| PPP6R3 | 1 | 0 | 0 | 0 | 1 | 0 |
| PPRC1 | 0 | 1 | 0 | 0 | 1 | 1 |
| PQLC3 | 1 | 0 | 0 | 1 | 2 | 1 |
| PRAME | 0 | 0 | 0 | 1 | 1 | 1 |
| PRAMEF1 | 0 | 13 | 0 | 0 | 13 | 13 |
| PRAMEF10 | 0 | 0 | 0 | 1 | 1 | 1 |
| PRAMEF11 | 0 | 4 | 0 | 4 | 8 | 8 |
| PRAMEF12 | 0 | 1 | 0 | 0 | 1 | 1 |
| PRAMEF2 | 0 | 8 | 0 | 0 | 8 | 8 |
| PRAMEF4 | 0 | 1 | 0 | 0 | 1 | 1 |
| PRB4 | 0 | 2 | 0 | 1 | 3 | 3 |
| PRC1 | 0 | 0 | 0 | 1 | 1 | 1 |
| PRCP | 0 | 0 | 0 | 1 | 1 | 1 |
| PRDM15 | 0 | 0 | 0 | 1 | 1 | 1 |
| PRDM4 | 0 | 0 | 0 | 1 | 1 | 1 |
| PRDM9 | 0 | 2 | 0 | 1 | 3 | 3 |
| PREPL | 1 | 0 | 0 | 0 | 1 | 0 |
| PREX2 | 0 | 0 | 0 | 1 | 1 | 1 |
| PRH1 | 0 | 0 | 0 | 1 | 1 | 1 |
| PRIM2 | 0 | 0 | 0 | 1 | 1 | 1 |
| PRKAB1 | 0 | 0 | 0 | 1 | 1 | 1 |
| PRKACA | 0 | 0 | 0 | 1 | 1 | 1 |
| PRKAR2A | 0 | 1 | 0 | 0 | 1 | 1 |
| PRKCG | 0 | 0 | 0 | 2 | 2 | 2 |
| PRKD3 | 0 | 0 | 0 | 5 | 5 | 5 |
| PRKDC | 0 | 0 | 0 | 2 | 2 | 2 |
| PRKG1 | 0 | 0 | 0 | 2 | 2 | 2 |
| PRKRA | 19 | 0 | 0 | 0 | 19 | 0 |
| PRMT6 | 0 | 0 | 0 | 1 | 1 | 1 |
| PRMT7 | 0 | 8 | 0 | 0 | 8 | 8 |
| PRODH | 0 | 16 | 0 | 0 | 16 | 16 |
| PROKR1 | 0 | 5 | 0 | 0 | 5 | 5 |
| PROKR2 | 0 | 0 | 0 | 3 | 3 | 3 |
| PROM2 | 0 | 0 | 0 | 1 | 1 | 1 |
| PROX2 | 0 | 0 | 0 | 1 | 1 | 1 |
| PROZ | 1 | 0 | 0 | 1 | 2 | 1 |
| PRPF31 | 1 | 0 | 0 | 0 | 1 | 0 |
| PRPF40B | 0 | 0 | 0 | 1 | 1 | 1 |
| PRPF8 | 0 | 13 | 0 | 3 | 16 | 16 |
| PRPH | 3 | 0 | 0 | 0 | 3 | 0 |
| PRPS1L1 | 0 | 0 | 0 | 5 | 5 | 5 |
| PRPSAP1 | 0 | 1 | 0 | 0 | 1 | 1 |
| PRR14 | 0 | 0 | 0 | 1 | 1 | 1 |
| PRR19 | 0 | 0 | 0 | 2 | 2 | 2 |
| PRR4 | 0 | 0 | 1 | 0 | 1 | 0 |
| PRRC1 | 0 | 0 | 0 | 1 | 1 | 1 |
| PRRC2B | 0 | 0 | 0 | 1 | 1 | 1 |
| PRRC2C | 0 | 0 | 0 | 2 | 2 | 2 |
| PRRT2 | 0 | 0 | 0 | 2 | 2 | 2 |
| PRSS12 | 0 | 0 | 0 | 3 | 3 | 3 |
| PRSS3 | 1 | 0 | 0 | 0 | 1 | 0 |
| PRSS36 | 0 | 1 | 0 | 0 | 1 | 1 |
| PRSS37 | 0 | 0 | 0 | 2 | 2 | 2 |
| PRSS45 | 0 | 1 | 0 | 0 | 1 | 1 |
| PRSS53 | 1 | 0 | 0 | 0 | 1 | 0 |
| PRSS57 | 0 | 1 | 0 | 0 | 1 | 1 |
| PRSS58 | 0 | 0 | 0 | 2 | 2 | 2 |
| PRSS8 | 0 | 0 | 0 | 2 | 2 | 2 |
| PRUNE2 | 1 | 0 | 0 | 1 | 2 | 1 |
| PSAPL1 | 0 | 0 | 0 | 17 | 17 | 17 |
| PSCA | 0 | 11 | 0 | 0 | 11 | 11 |
| PSD3 | 0 | 0 | 0 | 1 | 1 | 1 |
| PSD4 | 1 | 0 | 0 | 0 | 1 | 0 |
| PSEN2 | 0 | 0 | 0 | 1 | 1 | 1 |
| PSG1 | 0 | 1 | 0 | 0 | 1 | 1 |
| PSG11 | 0 | 1 | 0 | 0 | 1 | 1 |
| PSG5 | 0 | 1 | 0 | 0 | 1 | 1 |
| PSG6 | 0 | 2 | 0 | 2 | 4 | 4 |
| PSG8 | 1 | 0 | 0 | 0 | 1 | 0 |
| PSG9 | 0 | 4 | 0 | 0 | 4 | 4 |
| PSKH2 | 0 | 7 | 0 | 0 | 7 | 7 |
| PSMB1 | 0 | 0 | 0 | 1 | 1 | 1 |
| PSMB11 | 0 | 0 | 0 | 3 | 3 | 3 |
| PSMC3IP | 1 | 0 | 0 | 0 | 1 | 0 |
| PSMC5 | 0 | 0 | 0 | 1 | 1 | 1 |
| PSMD11 | 0 | 0 | 0 | 1 | 1 | 1 |
| PSMD12 | 4 | 0 | 0 | 0 | 4 | 0 |
| PSPC1 | 0 | 0 | 0 | 1 | 1 | 1 |
| PSPH | 0 | 1 | 0 | 0 | 1 | 1 |
| PSRC1 | 0 | 0 | 0 | 1 | 1 | 1 |
| PSTPIP2 | 0 | 0 | 0 | 1 | 1 | 1 |
| PTBP2 | 0 | 0 | 0 | 1 | 1 | 1 |
| PTCD3 | 0 | 1 | 0 | 0 | 1 | 1 |
| PTCH2 | 0 | 0 | 0 | 1 | 1 | 1 |
| PTCHD3 | 0 | 2 | 0 | 23 | 25 | 25 |
| PTDSS2 | 0 | 0 | 0 | 1 | 1 | 1 |
| PTGDR | 0 | 1 | 0 | 1 | 2 | 2 |
| PTGER2 | 0 | 1 | 0 | 0 | 1 | 1 |
| PTGES2 | 0 | 0 | 0 | 2 | 2 | 2 |
| PTGIS | 0 | 1 | 0 | 0 | 1 | 1 |
| PTH1R | 1 | 0 | 0 | 0 | 1 | 0 |
| PTH2R | 0 | 12 | 0 | 0 | 12 | 12 |
| PTK6 | 0 | 0 | 0 | 1 | 1 | 1 |
| PTK7 | 0 | 0 | 0 | 1 | 1 | 1 |
| PTPLAD1 | 0 | 1 | 0 | 0 | 1 | 1 |
| PTPN11 | 0 | 1 | 0 | 0 | 1 | 1 |
| PTPN13 | 1 | 0 | 0 | 0 | 1 | 0 |
| PTPN18 | 0 | 1 | 0 | 0 | 1 | 1 |
| PTPN21 | 0 | 3 | 0 | 0 | 3 | 3 |
| PTPN22 | 0 | 0 | 0 | 1 | 1 | 1 |
| PTPN5 | 0 | 0 | 0 | 1 | 1 | 1 |
| PTPN7 | 0 | 1 | 0 | 1 | 2 | 2 |
| PTPN9 | 0 | 0 | 0 | 2 | 2 | 2 |
| PTPRA | 0 | 0 | 0 | 1 | 1 | 1 |
| PTPRD | 0 | 0 | 0 | 1 | 1 | 1 |
| PTPRF | 0 | 0 | 0 | 3 | 3 | 3 |
| PTPRH | 0 | 4 | 0 | 4 | 8 | 8 |
| PTPRM | 2 | 1 | 0 | 0 | 3 | 1 |
| PTPRN2 | 1 | 0 | 0 | 1 | 2 | 1 |
| PTPRQ | 0 | 0 | 0 | 1 | 1 | 1 |
| PTPRR | 0 | 2 | 0 | 0 | 2 | 2 |
| PTPRT | 0 | 0 | 0 | 8 | 8 | 8 |
| PTPRU | 0 | 0 | 0 | 3 | 3 | 3 |
| PTS | 1 | 0 | 0 | 0 | 1 | 0 |
| PTTG2 | 0 | 0 | 0 | 12 | 12 | 12 |
| PUF60 | 0 | 0 | 0 | 2 | 2 | 2 |
| PUM2 | 0 | 0 | 0 | 1 | 1 | 1 |
| PVRIG | 0 | 1 | 0 | 2 | 3 | 3 |
| PWP2 | 0 | 1 | 0 | 0 | 1 | 1 |
| PXDNL | 0 | 17 | 0 | 3 | 20 | 20 |
| PXMP2 | 0 | 0 | 0 | 1 | 1 | 1 |
| PYGB | 0 | 0 | 0 | 2 | 2 | 2 |
| PYGL | 0 | 1 | 0 | 0 | 1 | 1 |
| PYGM | 0 | 2 | 0 | 1 | 3 | 3 |
| PYHIN1 | 0 | 0 | 0 | 1 | 1 | 1 |
| PYROXD1 | 0 | 0 | 0 | 1 | 1 | 1 |
| PYROXD2 | 0 | 0 | 0 | 1 | 1 | 1 |
| PZP | 1 | 3 | 0 | 1 | 5 | 4 |
| QARS | 0 | 0 | 0 | 1 | 1 | 1 |
| QPCT | 0 | 1 | 0 | 0 | 1 | 1 |
| QPRT | 0 | 1 | 0 | 0 | 1 | 1 |
| QRFPR | 0 | 0 | 0 | 1 | 1 | 1 |
| QRICH1 | 0 | 0 | 0 | 2 | 2 | 2 |
| QSOX2 | 0 | 1 | 0 | 0 | 1 | 1 |
| R3HDML | 0 | 0 | 0 | 5 | 5 | 5 |
| RAB11A | 0 | 0 | 1 | 0 | 1 | 0 |
| RAB11FIP5 | 0 | 0 | 0 | 1 | 1 | 1 |
| RAB37 | 0 | 0 | 0 | 1 | 1 | 1 |
| RAB38 | 0 | 0 | 0 | 1 | 1 | 1 |
| RAB3GAP2 | 0 | 0 | 0 | 1 | 1 | 1 |
| RAB3IP | 0 | 6 | 0 | 0 | 6 | 6 |
| RAB41 | 0 | 0 | 0 | 1 | 1 | 1 |
| RAB42 | 0 | 0 | 0 | 1 | 1 | 1 |
| RAB5C | 4 | 0 | 0 | 0 | 4 | 0 |
| RABEP1 | 0 | 0 | 0 | 1 | 1 | 1 |
| RABEPK | 0 | 0 | 0 | 8 | 8 | 8 |
| RABIF | 0 | 0 | 0 | 1 | 1 | 1 |
| RABL2A | 0 | 1 | 0 | 9 | 10 | 10 |
| RABL2B | 0 | 0 | 0 | 1 | 1 | 1 |
| RAD1 | 1 | 1 | 0 | 0 | 2 | 1 |
| RAD51AP1 | 0 | 0 | 0 | 1 | 1 | 1 |
| RAD51AP2 | 0 | 0 | 0 | 1 | 1 | 1 |
| RAD51C | 1 | 1 | 0 | 0 | 2 | 1 |
| RAD51D | 0 | 2 | 0 | 0 | 2 | 2 |
| RAD52 | 0 | 26 | 0 | 1 | 27 | 27 |
| RAD54L | 0 | 1 | 0 | 0 | 1 | 1 |
| RAD9B | 0 | 0 | 0 | 3 | 3 | 3 |
| RAET1E | 0 | 4 | 0 | 0 | 4 | 4 |
| RAG1 | 0 | 1 | 0 | 2 | 3 | 3 |
| RAG2 | 0 | 1 | 0 | 0 | 1 | 1 |
| RAI1 | 0 | 0 | 0 | 1 | 1 | 1 |
| RAI14 | 0 | 0 | 0 | 1 | 1 | 1 |
| RALBP1 | 0 | 0 | 0 | 1 | 1 | 1 |
| RALGAPA2 | 0 | 0 | 0 | 3 | 3 | 3 |
| RALY | 0 | 0 | 0 | 1 | 1 | 1 |
| RANBP1 | 0 | 0 | 0 | 1 | 1 | 1 |
| RANBP17 | 1 | 0 | 0 | 1 | 2 | 1 |
| RANBP3L | 1 | 0 | 0 | 0 | 1 | 0 |
| RANGRF | 0 | 5 | 0 | 0 | 5 | 5 |
| RAP1GAP | 1 | 0 | 0 | 0 | 1 | 0 |
| RAP2C | 0 | 0 | 0 | 2 | 2 | 2 |
| RAPGEF3 | 0 | 0 | 0 | 1 | 1 | 1 |
| RAPGEF4 | 4 | 0 | 0 | 0 | 4 | 0 |
| RAPGEF6 | 0 | 3 | 0 | 0 | 3 | 3 |
| RAPH1 | 0 | 2 | 0 | 0 | 2 | 2 |
| RAPSN | 0 | 0 | 0 | 1 | 1 | 1 |
| RARA | 0 | 0 | 0 | 1 | 1 | 1 |
| RARRES3 | 0 | 0 | 0 | 1 | 1 | 1 |
| RARS | 0 | 1 | 0 | 0 | 1 | 1 |
| RASAL2 | 0 | 0 | 1 | 1 | 2 | 1 |
| RASD1 | 0 | 1 | 0 | 0 | 1 | 1 |
| RASGEF1A | 0 | 1 | 0 | 0 | 1 | 1 |
| RASGRF1 | 0 | 0 | 0 | 1 | 1 | 1 |
| RASGRP3 | 0 | 1 | 0 | 0 | 1 | 1 |
| RASGRP4 | 0 | 1 | 0 | 0 | 1 | 1 |
| RASSF6 | 1 | 0 | 0 | 0 | 1 | 0 |
| RASSF9 | 0 | 1 | 0 | 0 | 1 | 1 |
| RBBP8 | 0 | 0 | 0 | 2 | 2 | 2 |
| RBCK1 | 1 | 0 | 0 | 0 | 1 | 0 |
| RBFOX1 | 1 | 0 | 0 | 0 | 1 | 0 |
| RBKS | 7 | 0 | 0 | 0 | 7 | 0 |
| RBL1 | 0 | 0 | 0 | 2 | 2 | 2 |
| RBM15B | 0 | 0 | 0 | 4 | 4 | 4 |
| RBM19 | 0 | 0 | 0 | 1 | 1 | 1 |
| RBM23 | 1 | 0 | 0 | 0 | 1 | 0 |
| RBM27 | 0 | 3 | 0 | 0 | 3 | 3 |
| RBM43 | 0 | 0 | 0 | 3 | 3 | 3 |
| RBM44 | 0 | 0 | 0 | 2 | 2 | 2 |
| RBM6 | 0 | 0 | 0 | 1 | 1 | 1 |
| RBMS1 | 2 | 0 | 0 | 0 | 2 | 0 |
| RBMS3 | 1 | 0 | 0 | 0 | 1 | 0 |
| RBMXL1 | 0 | 0 | 0 | 10 | 10 | 10 |
| RBP3 | 0 | 0 | 0 | 1 | 1 | 1 |
| RBPMS | 0 | 0 | 0 | 1 | 1 | 1 |
| RC3H1 | 0 | 0 | 0 | 5 | 5 | 5 |
| RCC1 | 0 | 2 | 0 | 0 | 2 | 2 |
| RCCD1 | 0 | 0 | 0 | 1 | 1 | 1 |
| RCN3 | 0 | 0 | 0 | 1 | 1 | 1 |
| RCOR2 | 0 | 1 | 0 | 0 | 1 | 1 |
| RCOR3 | 0 | 1 | 0 | 0 | 1 | 1 |
| RCSD1 | 0 | 1 | 0 | 0 | 1 | 1 |
| RDH12 | 1 | 1 | 0 | 0 | 2 | 1 |
| RDH13 | 0 | 0 | 0 | 1 | 1 | 1 |
| RDH16 | 0 | 1 | 0 | 0 | 1 | 1 |
| RDM1 | 0 | 0 | 0 | 2 | 2 | 2 |
| RDX | 8 | 1 | 0 | 0 | 9 | 1 |
| RECQL | 2 | 0 | 0 | 1 | 3 | 1 |
| RECQL5 | 0 | 0 | 0 | 1 | 1 | 1 |
| REEP2 | 12 | 0 | 0 | 0 | 12 | 0 |
| REEP3 | 0 | 0 | 0 | 1 | 1 | 1 |
| REG1B | 0 | 0 | 0 | 1 | 1 | 1 |
| REG3G | 0 | 1 | 0 | 0 | 1 | 1 |
| RELA | 0 | 11 | 0 | 0 | 11 | 11 |
| RELN | 0 | 5 | 0 | 2 | 7 | 7 |
| RELT | 0 | 0 | 0 | 1 | 1 | 1 |
| RERGL | 0 | 1 | 0 | 1 | 2 | 2 |
| RESP18 | 1 | 0 | 0 | 0 | 1 | 0 |
| RETSAT | 4 | 2 | 0 | 4 | 10 | 6 |
| REV1 | 0 | 0 | 0 | 1 | 1 | 1 |
| REV3L | 0 | 0 | 0 | 1 | 1 | 1 |
| RFC5 | 0 | 0 | 0 | 1 | 1 | 1 |
| RFFL | 0 | 0 | 0 | 1 | 1 | 1 |
| RFPL1 | 0 | 2 | 0 | 0 | 2 | 2 |
| RFPL2 | 2 | 0 | 0 | 0 | 2 | 0 |
| RFT1 | 0 | 0 | 0 | 5 | 5 | 5 |
| RFX3 | 0 | 0 | 0 | 2 | 2 | 2 |
| RFX8 | 2 | 0 | 0 | 0 | 2 | 0 |
| RG9MTD2 | 1 | 1 | 0 | 0 | 2 | 1 |
| RG9MTD3 | 1 | 0 | 0 | 1 | 2 | 1 |
| RGAG1 | 0 | 0 | 0 | 2 | 2 | 2 |
| RGAG4 | 0 | 0 | 0 | 3 | 3 | 3 |
| RGL2 | 0 | 0 | 0 | 4 | 4 | 4 |
| RGL3 | 0 | 1 | 0 | 1 | 2 | 2 |
| RGL4 | 0 | 0 | 0 | 2 | 2 | 2 |
| RGPD3 | 0 | 1 | 0 | 2 | 3 | 3 |
| RGS12 | 1 | 0 | 0 | 0 | 1 | 0 |
| RGS18 | 0 | 0 | 0 | 2 | 2 | 2 |
| RGS6 | 2 | 0 | 0 | 0 | 2 | 0 |
| RGS7 | 0 | 1 | 0 | 0 | 1 | 1 |
| RGS9 | 0 | 1 | 0 | 0 | 1 | 1 |
| RHBDD3 | 0 | 2 | 0 | 0 | 2 | 2 |
| RHBDF1 | 0 | 0 | 0 | 1 | 1 | 1 |
| RHBDL2 | 1 | 0 | 0 | 0 | 1 | 0 |
| RHBG | 0 | 1 | 0 | 2 | 3 | 3 |
| RHCG | 0 | 0 | 0 | 1 | 1 | 1 |
| RHO | 0 | 0 | 0 | 1 | 1 | 1 |
| RHOBTB3 | 0 | 1 | 0 | 1 | 2 | 2 |
| RICTOR | 0 | 0 | 0 | 1 | 1 | 1 |
| RILP | 0 | 0 | 0 | 1 | 1 | 1 |
| RIMBP2 | 0 | 0 | 0 | 1 | 1 | 1 |
| RIMBP3 | 0 | 0 | 0 | 3 | 3 | 3 |
| RIMS1 | 3 | 0 | 0 | 0 | 3 | 0 |
| RIMS2 | 0 | 2 | 0 | 0 | 2 | 2 |
| RINT1 | 0 | 3 | 0 | 0 | 3 | 3 |
| RIOK2 | 0 | 1 | 0 | 0 | 1 | 1 |
| RIPK1 | 0 | 0 | 0 | 1 | 1 | 1 |
| RIPK3 | 0 | 3 | 0 | 4 | 7 | 7 |
| RIPK4 | 0 | 0 | 0 | 2 | 2 | 2 |
| RLBP1 | 0 | 0 | 0 | 2 | 2 | 2 |
| RLN1 | 0 | 0 | 0 | 1 | 1 | 1 |
| RLN3 | 0 | 2 | 0 | 0 | 2 | 2 |
| RLTPR | 0 | 0 | 0 | 3 | 3 | 3 |
| RMND5B | 1 | 1 | 0 | 0 | 2 | 1 |
| RNASE2 | 0 | 1 | 0 | 0 | 1 | 1 |
| RNASE9 | 0 | 0 | 0 | 1 | 1 | 1 |
| RNASEH2A | 5 | 0 | 0 | 0 | 5 | 0 |
| RNASEL | 0 | 4 | 0 | 3 | 7 | 7 |
| RNF112 | 0 | 2 | 0 | 0 | 2 | 2 |
| RNF123 | 0 | 7 | 0 | 1 | 8 | 8 |
| RNF141 | 0 | 0 | 6 | 0 | 6 | 0 |
| RNF145 | 0 | 0 | 0 | 1 | 1 | 1 |
| RNF168 | 0 | 0 | 0 | 1 | 1 | 1 |
| RNF180 | 0 | 0 | 0 | 2 | 2 | 2 |
| RNF181 | 0 | 0 | 0 | 1 | 1 | 1 |
| RNF186 | 0 | 2 | 0 | 0 | 2 | 2 |
| RNF187 | 0 | 0 | 0 | 1 | 1 | 1 |
| RNF19A | 0 | 1 | 0 | 0 | 1 | 1 |
| RNF20 | 0 | 1 | 0 | 0 | 1 | 1 |
| RNF214 | 0 | 0 | 0 | 1 | 1 | 1 |
| RNF217 | 0 | 1 | 0 | 0 | 1 | 1 |
| RNF25 | 0 | 2 | 0 | 2 | 4 | 4 |
| RNF34 | 0 | 0 | 0 | 1 | 1 | 1 |
| RNLS | 0 | 0 | 0 | 1 | 1 | 1 |
| RNMTL1 | 0 | 1 | 0 | 0 | 1 | 1 |
| RNPEP | 2 | 0 | 0 | 0 | 2 | 0 |
| RNPS1 | 0 | 0 | 0 | 1 | 1 | 1 |
| ROBO3 | 0 | 0 | 0 | 1 | 1 | 1 |
| ROCK1 | 0 | 1 | 0 | 0 | 1 | 1 |
| ROM1 | 0 | 0 | 0 | 3 | 3 | 3 |
| ROPN1B | 0 | 1 | 0 | 0 | 1 | 1 |
| ROPN1L | 0 | 1 | 0 | 0 | 1 | 1 |
| ROR2 | 0 | 4 | 0 | 0 | 4 | 4 |
| ROS1 | 0 | 1 | 0 | 0 | 1 | 1 |
| RP1L1 | 0 | 0 | 0 | 2 | 2 | 2 |
| RP9 | 0 | 0 | 5 | 0 | 5 | 0 |
| RPAIN | 0 | 0 | 0 | 1 | 1 | 1 |
| RPAP2 | 0 | 0 | 0 | 1 | 1 | 1 |
| RPF1 | 0 | 0 | 0 | 2 | 2 | 2 |
| RPGRIP1 | 0 | 0 | 0 | 1 | 1 | 1 |
| RPGRIP1L | 0 | 0 | 0 | 3 | 3 | 3 |
| RPL10 | 0 | 0 | 0 | 1 | 1 | 1 |
| RPL3 | 0 | 0 | 0 | 1 | 1 | 1 |
| RPL30 | 0 | 0 | 0 | 1 | 1 | 1 |
| RPL3L | 0 | 0 | 0 | 3 | 3 | 3 |
| RPS27L | 0 | 1 | 0 | 0 | 1 | 1 |
| RPS6KA3 | 0 | 0 | 0 | 1 | 1 | 1 |
| RPS6KB2 | 0 | 0 | 0 | 1 | 1 | 1 |
| RPTN | 0 | 2 | 0 | 0 | 2 | 2 |
| RPTOR | 0 | 0 | 0 | 1 | 1 | 1 |
| RPUSD3 | 0 | 0 | 1 | 0 | 1 | 0 |
| RPUSD4 | 1 | 0 | 0 | 0 | 1 | 0 |
| RRAS | 0 | 3 | 0 | 0 | 3 | 3 |
| RRH | 0 | 0 | 0 | 1 | 1 | 1 |
| RRM1 | 0 | 2 | 0 | 0 | 2 | 2 |
| RRN3 | 0 | 0 | 0 | 6 | 6 | 6 |
| RRP1 | 0 | 0 | 0 | 1 | 1 | 1 |
| RRP12 | 4 | 0 | 0 | 0 | 4 | 0 |
| RRP15 | 0 | 0 | 0 | 5 | 5 | 5 |
| RRP36 | 1 | 0 | 0 | 0 | 1 | 0 |
| RRP8 | 1 | 0 | 0 | 1 | 2 | 1 |
| RSBN1 | 0 | 0 | 0 | 1 | 1 | 1 |
| RSF1 | 0 | 14 | 0 | 0 | 14 | 14 |
| RSPH1 | 2 | 0 | 0 | 0 | 2 | 0 |
| RSPO3 | 0 | 0 | 0 | 1 | 1 | 1 |
| RTF1 | 0 | 0 | 0 | 1 | 1 | 1 |
| RTL1 | 0 | 0 | 0 | 1 | 1 | 1 |
| RTN4IP1 | 0 | 0 | 0 | 2 | 2 | 2 |
| RTP2 | 0 | 0 | 0 | 1 | 1 | 1 |
| RTTN | 0 | 0 | 0 | 1 | 1 | 1 |
| RUFY3 | 0 | 0 | 0 | 2 | 2 | 2 |
| RUFY4 | 0 | 1 | 0 | 0 | 1 | 1 |
| RUSC1-AS1 | 0 | 8 | 0 | 0 | 8 | 8 |
| RUSC2 | 0 | 1 | 0 | 1 | 2 | 2 |
| RWDD2B | 1 | 0 | 0 | 1 | 2 | 1 |
| RXFP4 | 0 | 1 | 0 | 0 | 1 | 1 |
| RXRA | 0 | 1 | 0 | 0 | 1 | 1 |
| RYR1 | 0 | 1 | 0 | 4 | 5 | 5 |
| RYR2 | 0 | 1 | 0 | 3 | 4 | 4 |
| RYR3 | 0 | 0 | 0 | 1 | 1 | 1 |
| S100A3 | 0 | 0 | 0 | 6 | 6 | 6 |
| S1PR1 | 0 | 1 | 0 | 0 | 1 | 1 |
| S1PR2 | 0 | 1 | 0 | 0 | 1 | 1 |
| SAA1 | 0 | 0 | 0 | 2 | 2 | 2 |
| SAAL1 | 1 | 0 | 0 | 0 | 1 | 0 |
| SACM1L | 0 | 1 | 0 | 0 | 1 | 1 |
| SACS | 0 | 2 | 0 | 3 | 5 | 5 |
| SAFB2 | 0 | 0 | 0 | 5 | 5 | 5 |
| SALL2 | 0 | 0 | 0 | 1 | 1 | 1 |
| SAMD11 | 0 | 0 | 0 | 2 | 2 | 2 |
| SAMD12 | 0 | 0 | 0 | 1 | 1 | 1 |
| SAMD15 | 0 | 2 | 0 | 1 | 3 | 3 |
| SAMD3 | 1 | 1 | 0 | 1 | 3 | 2 |
| SAMD4A | 9 | 0 | 0 | 0 | 9 | 0 |
| SAMD9 | 0 | 0 | 0 | 6 | 6 | 6 |
| SAMD9L | 0 | 1 | 0 | 3 | 4 | 4 |
| SAP130 | 0 | 2 | 0 | 1 | 3 | 3 |
| SARDH | 0 | 2 | 0 | 0 | 2 | 2 |
| SARM1 | 0 | 0 | 0 | 8 | 8 | 8 |
| SARS2 | 0 | 1 | 0 | 0 | 1 | 1 |
| SASH1 | 0 | 0 | 0 | 6 | 6 | 6 |
| SASH3 | 1 | 0 | 0 | 0 | 1 | 0 |
| SATL1 | 1 | 3 | 0 | 0 | 4 | 3 |
| SBDS | 2 | 0 | 0 | 0 | 2 | 0 |
| SBF2 | 1 | 0 | 0 | 0 | 1 | 0 |
| SC5DL | 0 | 1 | 0 | 0 | 1 | 1 |
| SCAF1 | 0 | 0 | 0 | 1 | 1 | 1 |
| SCAF11 | 1 | 0 | 0 | 2 | 3 | 2 |
| SCAF8 | 0 | 1 | 0 | 0 | 1 | 1 |
| SCAMP1 | 0 | 1 | 0 | 0 | 1 | 1 |
| SCAP | 0 | 0 | 0 | 1 | 1 | 1 |
| SCAPER | 0 | 1 | 0 | 1 | 2 | 2 |
| SCARA3 | 0 | 1 | 0 | 2 | 3 | 3 |
| SCEL | 1 | 1 | 0 | 0 | 2 | 1 |
| SCFD1 | 0 | 1 | 0 | 0 | 1 | 1 |
| SCG2 | 0 | 0 | 0 | 2 | 2 | 2 |
| SCGB1A1 | 0 | 0 | 0 | 2 | 2 | 2 |
| SCGB1D2 | 0 | 1 | 0 | 0 | 1 | 1 |
| SCGN | 0 | 0 | 0 | 1 | 1 | 1 |
| SCIN | 0 | 1 | 0 | 0 | 1 | 1 |
| SCML4 | 0 | 1 | 0 | 0 | 1 | 1 |
| SCN10A | 0 | 0 | 0 | 3 | 3 | 3 |
| SCN11A | 0 | 0 | 0 | 1 | 1 | 1 |
| SCN1A | 0 | 3 | 0 | 0 | 3 | 3 |
| SCN2A | 0 | 9 | 0 | 0 | 9 | 9 |
| SCN3A | 0 | 15 | 0 | 0 | 15 | 15 |
| SCN3B | 0 | 0 | 0 | 2 | 2 | 2 |
| SCN4A | 0 | 2 | 0 | 1 | 3 | 3 |
| SCN5A | 2 | 0 | 0 | 11 | 13 | 11 |
| SCN7A | 3 | 0 | 0 | 1 | 4 | 1 |
| SCNM1 | 4 | 0 | 0 | 0 | 4 | 0 |
| SCRN1 | 0 | 0 | 0 | 1 | 1 | 1 |
| SCRN2 | 0 | 0 | 0 | 1 | 1 | 1 |
| SCRN3 | 0 | 1 | 0 | 0 | 1 | 1 |
| SCUBE2 | 1 | 0 | 0 | 0 | 1 | 0 |
| SCYL1 | 1 | 0 | 0 | 0 | 1 | 0 |
| SDC2 | 0 | 0 | 0 | 5 | 5 | 5 |
| SDCCAG8 | 4 | 0 | 0 | 0 | 4 | 0 |
| SDHA | 0 | 1 | 0 | 2 | 3 | 3 |
| SDK2 | 0 | 0 | 0 | 1 | 1 | 1 |
| SDR39U1 | 0 | 0 | 0 | 1 | 1 | 1 |
| SDSL | 0 | 3 | 0 | 3 | 6 | 6 |
| SEC14L4 | 0 | 3 | 0 | 0 | 3 | 3 |
| SEC22B | 0 | 1 | 0 | 0 | 1 | 1 |
| SEC22C | 1 | 0 | 0 | 0 | 1 | 0 |
| SEC23A | 0 | 3 | 0 | 0 | 3 | 3 |
| SEC23B | 0 | 1 | 0 | 1 | 2 | 2 |
| SEC31B | 0 | 1 | 0 | 1 | 2 | 2 |
| SEC61A2 | 0 | 1 | 0 | 2 | 3 | 3 |
| SEL1L2 | 1 | 0 | 0 | 1 | 2 | 1 |
| SELL | 18 | 0 | 0 | 0 | 18 | 0 |
| SELP | 0 | 1 | 0 | 6 | 7 | 7 |
| SELPLG | 0 | 0 | 0 | 1 | 1 | 1 |
| SEMA5B | 0 | 0 | 0 | 2 | 2 | 2 |
| SEMA6A | 0 | 3 | 0 | 0 | 3 | 3 |
| SEMA6C | 0 | 0 | 0 | 1 | 1 | 1 |
| SEMG2 | 0 | 3 | 0 | 0 | 3 | 3 |
| SENP5 | 0 | 1 | 0 | 0 | 1 | 1 |
| Sep-01 | 1 | 0 | 0 | 0 | 1 | 0 |
| Sep-02 | 0 | 0 | 0 | 1 | 1 | 1 |
| Sep-06 | 0 | 1 | 0 | 0 | 1 | 1 |
| Sep-07 | 0 | 1 | 0 | 0 | 1 | 1 |
| Sep-10 | 0 | 1 | 0 | 0 | 1 | 1 |
| Sep-14 | 0 | 1 | 0 | 0 | 1 | 1 |
| SEPSECS | 0 | 0 | 0 | 1 | 1 | 1 |
| SERAC1 | 1 | 1 | 0 | 4 | 6 | 5 |
| SERGEF | 0 | 3 | 0 | 0 | 3 | 3 |
| SERHL2 | 5 | 0 | 0 | 1 | 6 | 1 |
| SERINC5 | 1 | 0 | 0 | 0 | 1 | 0 |
| SERPINA1 | 1 | 0 | 0 | 0 | 1 | 0 |
| SERPINA10 | 0 | 8 | 0 | 0 | 8 | 8 |
| SERPINA12 | 0 | 5 | 0 | 0 | 5 | 5 |
| SERPINA3 | 0 | 0 | 0 | 2 | 2 | 2 |
| SERPINA4 | 0 | 1 | 0 | 1 | 2 | 2 |
| SERPINA9 | 3 | 6 | 0 | 0 | 9 | 6 |
| SERPINB10 | 0 | 1 | 0 | 1 | 2 | 2 |
| SERPINB11 | 1 | 0 | 1 | 0 | 2 | 0 |
| SERPINB13 | 0 | 2 | 0 | 0 | 2 | 2 |
| SERPINB3 | 0 | 0 | 0 | 5 | 5 | 5 |
| SERPINB4 | 0 | 0 | 0 | 1 | 1 | 1 |
| SERPINB7 | 0 | 1 | 0 | 0 | 1 | 1 |
| SERPINC1 | 0 | 0 | 0 | 1 | 1 | 1 |
| SERPINE2 | 0 | 1 | 0 | 0 | 1 | 1 |
| SERPINH1 | 0 | 4 | 0 | 1 | 5 | 5 |
| SERPINI1 | 0 | 0 | 0 | 2 | 2 | 2 |
| SESN2 | 0 | 0 | 0 | 1 | 1 | 1 |
| SESN3 | 1 | 0 | 0 | 0 | 1 | 0 |
| SESTD1 | 0 | 0 | 0 | 15 | 15 | 15 |
| SETD2 | 10 | 0 | 0 | 0 | 10 | 0 |
| SETD4 | 0 | 1 | 0 | 0 | 1 | 1 |
| SETD5 | 0 | 0 | 0 | 1 | 1 | 1 |
| SETD6 | 0 | 0 | 0 | 1 | 1 | 1 |
| SETDB1 | 0 | 0 | 0 | 1 | 1 | 1 |
| SF3A3 | 0 | 0 | 0 | 2 | 2 | 2 |
| SF3B3 | 0 | 0 | 0 | 17 | 17 | 17 |
| SF3B4 | 0 | 0 | 0 | 1 | 1 | 1 |
| SFI1 | 0 | 0 | 0 | 1 | 1 | 1 |
| SFN | 0 | 1 | 0 | 1 | 2 | 2 |
| SFT2D1 | 0 | 0 | 0 | 1 | 1 | 1 |
| SFXN3 | 0 | 0 | 0 | 1 | 1 | 1 |
| SFXN4 | 3 | 0 | 0 | 0 | 3 | 0 |
| SGCB | 0 | 0 | 0 | 1 | 1 | 1 |
| SGK1 | 0 | 1 | 0 | 0 | 1 | 1 |
| SGK223 | 0 | 1 | 0 | 0 | 1 | 1 |
| SGK494 | 1 | 0 | 0 | 0 | 1 | 0 |
| SGPP2 | 2 | 0 | 0 | 0 | 2 | 0 |
| SGSH | 0 | 0 | 0 | 1 | 1 | 1 |
| SGSM2 | 0 | 0 | 0 | 2 | 2 | 2 |
| SGSM3 | 1 | 0 | 0 | 0 | 1 | 0 |
| SH2D2A | 0 | 0 | 0 | 1 | 1 | 1 |
| SH2D4A | 1 | 0 | 0 | 0 | 1 | 0 |
| SH2D6 | 4 | 0 | 0 | 0 | 4 | 0 |
| SH3BP2 | 0 | 0 | 0 | 3 | 3 | 3 |
| SH3BP4 | 0 | 4 | 0 | 0 | 4 | 4 |
| SH3D21 | 0 | 1 | 0 | 0 | 1 | 1 |
| SH3GLB2 | 2 | 0 | 0 | 0 | 2 | 0 |
| SH3PXD2B | 0 | 0 | 0 | 2 | 2 | 2 |
| SH3TC1 | 5 | 0 | 0 | 1 | 6 | 1 |
| SH3TC2 | 0 | 1 | 0 | 0 | 1 | 1 |
| SHANK1 | 0 | 0 | 0 | 1 | 1 | 1 |
| SHANK2 | 0 | 8 | 1 | 0 | 9 | 8 |
| SHBG | 0 | 0 | 0 | 3 | 3 | 3 |
| SHC4 | 1 | 0 | 0 | 1 | 2 | 1 |
| SHCBP1 | 0 | 0 | 0 | 3 | 3 | 3 |
| SHE | 0 | 0 | 0 | 1 | 1 | 1 |
| SHF | 0 | 0 | 0 | 1 | 1 | 1 |
| SHKBP1 | 1 | 0 | 0 | 1 | 2 | 1 |
| SHPK | 0 | 1 | 0 | 1 | 2 | 2 |
| SHROOM1 | 1 | 0 | 0 | 0 | 1 | 0 |
| SHROOM2 | 0 | 6 | 0 | 1 | 7 | 7 |
| SIDT1 | 1 | 1 | 0 | 0 | 2 | 1 |
| SIDT2 | 0 | 0 | 0 | 1 | 1 | 1 |
| SIGLEC1 | 0 | 5 | 0 | 0 | 5 | 5 |
| SIGLEC10 | 1 | 0 | 0 | 0 | 1 | 0 |
| SIGLEC12 | 0 | 1 | 0 | 0 | 1 | 1 |
| SIGLEC5 | 0 | 8 | 0 | 10 | 18 | 18 |
| SIGLEC8 | 0 | 0 | 0 | 2 | 2 | 2 |
| SIK2 | 0 | 0 | 0 | 1 | 1 | 1 |
| SIK3 | 0 | 1 | 0 | 0 | 1 | 1 |
| SIKE1 | 0 | 0 | 0 | 1 | 1 | 1 |
| SIN3B | 0 | 0 | 0 | 3 | 3 | 3 |
| SIPA1L2 | 0 | 0 | 0 | 2 | 2 | 2 |
| SIRPB1 | 0 | 0 | 0 | 12 | 12 | 12 |
| SIRPG | 0 | 1 | 0 | 0 | 1 | 1 |
| SIRT3 | 1 | 0 | 0 | 0 | 1 | 0 |
| SIRT4 | 0 | 0 | 0 | 1 | 1 | 1 |
| SKA3 | 0 | 10 | 0 | 25 | 35 | 35 |
| SKIV2L | 1 | 1 | 0 | 0 | 2 | 1 |
| SLA | 0 | 0 | 0 | 2 | 2 | 2 |
| SLA2 | 0 | 1 | 0 | 1 | 2 | 2 |
| SLAIN1 | 0 | 0 | 0 | 16 | 16 | 16 |
| SLAMF1 | 0 | 0 | 0 | 1 | 1 | 1 |
| SLAMF8 | 0 | 1 | 0 | 0 | 1 | 1 |
| SLBP | 0 | 0 | 0 | 1 | 1 | 1 |
| SLC10A1 | 0 | 2 | 0 | 0 | 2 | 2 |
| SLC10A4 | 0 | 0 | 0 | 1 | 1 | 1 |
| SLC12A1 | 0 | 1 | 0 | 0 | 1 | 1 |
| SLC12A2 | 8 | 0 | 0 | 0 | 8 | 0 |
| SLC12A5 | 0 | 0 | 0 | 14 | 14 | 14 |
| SLC12A6 | 0 | 1 | 0 | 0 | 1 | 1 |
| SLC12A7 | 0 | 0 | 0 | 1 | 1 | 1 |
| SLC12A8 | 0 | 1 | 0 | 1 | 2 | 2 |
| SLC12A9 | 0 | 0 | 0 | 1 | 1 | 1 |
| SLC13A1 | 0 | 5 | 0 | 0 | 5 | 5 |
| SLC13A2 | 0 | 1 | 0 | 0 | 1 | 1 |
| SLC13A5 | 0 | 0 | 0 | 4 | 4 | 4 |
| SLC14A1 | 0 | 0 | 0 | 21 | 21 | 21 |
| SLC15A1 | 0 | 0 | 0 | 1 | 1 | 1 |
| SLC16A12 | 0 | 0 | 0 | 1 | 1 | 1 |
| SLC17A4 | 0 | 8 | 0 | 2 | 10 | 10 |
| SLC17A5 | 0 | 0 | 0 | 2 | 2 | 2 |
| SLC17A7 | 0 | 0 | 0 | 1 | 1 | 1 |
| SLC17A9 | 0 | 0 | 0 | 1 | 1 | 1 |
| SLC18A1 | 0 | 0 | 0 | 1 | 1 | 1 |
| SLC18A2 | 2 | 0 | 0 | 0 | 2 | 0 |
| SLC19A3 | 0 | 0 | 0 | 1 | 1 | 1 |
| SLC1A1 | 0 | 1 | 0 | 0 | 1 | 1 |
| SLC1A5 | 0 | 0 | 0 | 1 | 1 | 1 |
| SLC1A7 | 12 | 0 | 0 | 0 | 12 | 0 |
| SLC22A10 | 0 | 1 | 0 | 2 | 3 | 3 |
| SLC22A11 | 0 | 1 | 0 | 0 | 1 | 1 |
| SLC22A14 | 4 | 0 | 0 | 21 | 25 | 21 |
| SLC22A15 | 0 | 0 | 0 | 1 | 1 | 1 |
| SLC22A25 | 8 | 0 | 0 | 4 | 12 | 4 |
| SLC22A3 | 0 | 3 | 0 | 0 | 3 | 3 |
| SLC22A9 | 0 | 0 | 0 | 5 | 5 | 5 |
| SLC23A1 | 1 | 0 | 0 | 1 | 2 | 1 |
| SLC23A2 | 0 | 0 | 0 | 3 | 3 | 3 |
| SLC24A6 | 1 | 0 | 0 | 0 | 1 | 0 |
| SLC25A13 | 0 | 0 | 0 | 1 | 1 | 1 |
| SLC25A2 | 0 | 12 | 0 | 2 | 14 | 14 |
| SLC25A21 | 0 | 1 | 0 | 0 | 1 | 1 |
| SLC25A22 | 0 | 0 | 0 | 1 | 1 | 1 |
| SLC25A24 | 0 | 1 | 0 | 2 | 3 | 3 |
| SLC25A31 | 0 | 6 | 0 | 0 | 6 | 6 |
| SLC25A32 | 0 | 0 | 0 | 1 | 1 | 1 |
| SLC25A33 | 0 | 0 | 0 | 1 | 1 | 1 |
| SLC25A45 | 0 | 1 | 0 | 0 | 1 | 1 |
| SLC25A48 | 0 | 1 | 0 | 0 | 1 | 1 |
| SLC25A5 | 0 | 0 | 0 | 7 | 7 | 7 |
| SLC26A10 | 4 | 4 | 0 | 0 | 8 | 4 |
| SLC26A2 | 0 | 0 | 0 | 2 | 2 | 2 |
| SLC26A4 | 2 | 0 | 0 | 0 | 2 | 0 |
| SLC26A7 | 0 | 0 | 0 | 1 | 1 | 1 |
| SLC26A8 | 1 | 1 | 0 | 1 | 3 | 2 |
| SLC27A3 | 0 | 0 | 0 | 3 | 3 | 3 |
| SLC27A6 | 0 | 0 | 0 | 2 | 2 | 2 |
| SLC28A3 | 0 | 1 | 0 | 0 | 1 | 1 |
| SLC29A4 | 0 | 0 | 0 | 1 | 1 | 1 |
| SLC2A11 | 0 | 0 | 0 | 6 | 6 | 6 |
| SLC2A5 | 0 | 0 | 0 | 1 | 1 | 1 |
| SLC2A9 | 0 | 1 | 0 | 0 | 1 | 1 |
| SLC30A2 | 0 | 0 | 1 | 0 | 1 | 0 |
| SLC30A8 | 0 | 1 | 0 | 1 | 2 | 2 |
| SLC34A1 | 0 | 0 | 0 | 1 | 1 | 1 |
| SLC34A2 | 1 | 7 | 0 | 2 | 10 | 9 |
| SLC35B3 | 0 | 0 | 0 | 1 | 1 | 1 |
| SLC35C2 | 0 | 0 | 0 | 2 | 2 | 2 |
| SLC35F5 | 0 | 0 | 0 | 4 | 4 | 4 |
| SLC35G3 | 0 | 0 | 0 | 1 | 1 | 1 |
| SLC36A2 | 0 | 0 | 0 | 1 | 1 | 1 |
| SLC36A4 | 6 | 0 | 0 | 1 | 7 | 1 |
| SLC37A2 | 0 | 0 | 0 | 1 | 1 | 1 |
| SLC37A4 | 0 | 11 | 1 | 14 | 26 | 25 |
| SLC38A10 | 0 | 0 | 0 | 2 | 2 | 2 |
| SLC38A6 | 1 | 0 | 0 | 0 | 1 | 0 |
| SLC39A2 | 0 | 1 | 0 | 0 | 1 | 1 |
| SLC39A4 | 0 | 0 | 0 | 1 | 1 | 1 |
| SLC39A5 | 0 | 1 | 0 | 1 | 2 | 2 |
| SLC3A1 | 0 | 1 | 0 | 0 | 1 | 1 |
| SLC43A3 | 0 | 0 | 0 | 1 | 1 | 1 |
| SLC44A3 | 0 | 0 | 0 | 1 | 1 | 1 |
| SLC44A4 | 0 | 0 | 0 | 1 | 1 | 1 |
| SLC45A2 | 0 | 0 | 0 | 2 | 2 | 2 |
| SLC45A3 | 0 | 0 | 0 | 2 | 2 | 2 |
| SLC46A1 | 0 | 0 | 0 | 6 | 6 | 6 |
| SLC47A1 | 0 | 0 | 0 | 1 | 1 | 1 |
| SLC4A10 | 0 | 1 | 0 | 0 | 1 | 1 |
| SLC4A2 | 0 | 0 | 0 | 1 | 1 | 1 |
| SLC4A3 | 0 | 0 | 0 | 6 | 6 | 6 |
| SLC4A5 | 0 | 0 | 0 | 1 | 1 | 1 |
| SLC4A7 | 0 | 1 | 0 | 0 | 1 | 1 |
| SLC4A9 | 0 | 0 | 0 | 1 | 1 | 1 |
| SLC5A10 | 0 | 3 | 0 | 0 | 3 | 3 |
| SLC5A12 | 0 | 0 | 0 | 1 | 1 | 1 |
| SLC5A2 | 0 | 1 | 0 | 3 | 4 | 4 |
| SLC5A4 | 0 | 8 | 0 | 7 | 15 | 15 |
| SLC5A6 | 0 | 1 | 0 | 1 | 2 | 2 |
| SLC6A16 | 0 | 2 | 0 | 0 | 2 | 2 |
| SLC6A18 | 0 | 1 | 0 | 1 | 2 | 2 |
| SLC6A19 | 0 | 0 | 0 | 3 | 3 | 3 |
| SLC6A5 | 0 | 0 | 0 | 1 | 1 | 1 |
| SLC6A6 | 0 | 0 | 0 | 1 | 1 | 1 |
| SLC7A13 | 1 | 0 | 0 | 0 | 1 | 0 |
| SLC7A2 | 0 | 0 | 0 | 1 | 1 | 1 |
| SLC8A1 | 0 | 0 | 0 | 13 | 13 | 13 |
| SLC9A1 | 0 | 2 | 0 | 0 | 2 | 2 |
| SLC9A11 | 0 | 0 | 0 | 1 | 1 | 1 |
| SLC9A4 | 0 | 0 | 0 | 2 | 2 | 2 |
| SLC9A5 | 0 | 0 | 0 | 1 | 1 | 1 |
| SLC9A9 | 1 | 0 | 0 | 0 | 1 | 0 |
| SLCO1A2 | 0 | 0 | 0 | 2 | 2 | 2 |
| SLCO1B1 | 0 | 2 | 0 | 19 | 21 | 21 |
| SLCO1B3 | 0 | 0 | 0 | 2 | 2 | 2 |
| SLCO2B1 | 0 | 0 | 0 | 1 | 1 | 1 |
| SLCO4C1 | 0 | 0 | 1 | 0 | 1 | 0 |
| SLCO5A1 | 0 | 1 | 0 | 1 | 2 | 2 |
| SLCO6A1 | 0 | 4 | 0 | 0 | 4 | 4 |
| SLFN11 | 0 | 1 | 0 | 0 | 1 | 1 |
| SLFN12 | 0 | 0 | 0 | 1 | 1 | 1 |
| SLFN12L | 0 | 0 | 0 | 8 | 8 | 8 |
| SLFN13 | 0 | 1 | 0 | 1 | 2 | 2 |
| SLFN5 | 0 | 2 | 0 | 2 | 4 | 4 |
| SLFNL1 | 0 | 2 | 0 | 5 | 7 | 7 |
| SLIT1 | 0 | 1 | 0 | 0 | 1 | 1 |
| SLIT3 | 0 | 1 | 0 | 0 | 1 | 1 |
| SLITRK3 | 0 | 9 | 0 | 0 | 9 | 9 |
| SLITRK5 | 0 | 0 | 0 | 2 | 2 | 2 |
| SLITRK6 | 0 | 1 | 0 | 0 | 1 | 1 |
| SLMAP | 0 | 2 | 0 | 1 | 3 | 3 |
| SMAD7 | 0 | 0 | 0 | 1 | 1 | 1 |
| SMAD9 | 0 | 2 | 0 | 0 | 2 | 2 |
| SMAP1 | 0 | 1 | 0 | 5 | 6 | 6 |
| SMAP2 | 2 | 0 | 0 | 0 | 2 | 0 |
| SMARCA4 | 0 | 0 | 0 | 3 | 3 | 3 |
| SMARCC2 | 0 | 0 | 0 | 1 | 1 | 1 |
| SMC1B | 0 | 0 | 0 | 2 | 2 | 2 |
| SMC5 | 0 | 0 | 0 | 1 | 1 | 1 |
| SMC6 | 0 | 1 | 0 | 0 | 1 | 1 |
| SMCHD1 | 0 | 0 | 0 | 1 | 1 | 1 |
| SMCR8 | 0 | 1 | 0 | 1 | 2 | 2 |
| SMEK1 | 0 | 0 | 0 | 1 | 1 | 1 |
| SMEK2 | 1 | 0 | 0 | 0 | 1 | 0 |
| SMG1 | 1 | 0 | 0 | 1 | 2 | 1 |
| SMG5 | 0 | 0 | 0 | 2 | 2 | 2 |
| SMG6 | 0 | 6 | 0 | 1 | 7 | 7 |
| SMOC2 | 0 | 0 | 0 | 3 | 3 | 3 |
| SMOX | 0 | 0 | 0 | 1 | 1 | 1 |
| SMPD3 | 0 | 0 | 0 | 1 | 1 | 1 |
| SMPD4 | 0 | 7 | 0 | 2 | 9 | 9 |
| SMPDL3B | 0 | 0 | 0 | 3 | 3 | 3 |
| SMR3B | 0 | 0 | 0 | 1 | 1 | 1 |
| SMTN | 0 | 0 | 0 | 2 | 2 | 2 |
| SMTNL1 | 0 | 1 | 0 | 0 | 1 | 1 |
| SMTNL2 | 1 | 0 | 0 | 0 | 1 | 0 |
| SMUG1 | 0 | 1 | 0 | 0 | 1 | 1 |
| SMYD4 | 1 | 3 | 0 | 0 | 4 | 3 |
| SMYD5 | 0 | 0 | 0 | 2 | 2 | 2 |
| SNAP29 | 0 | 1 | 0 | 0 | 1 | 1 |
| SNAPC1 | 0 | 0 | 0 | 5 | 5 | 5 |
| SNAPC2 | 0 | 0 | 0 | 1 | 1 | 1 |
| SNAPC5 | 0 | 1 | 0 | 0 | 1 | 1 |
| SNAPIN | 0 | 1 | 0 | 0 | 1 | 1 |
| SNED1 | 0 | 0 | 0 | 2 | 2 | 2 |
| SNRNP200 | 0 | 0 | 0 | 3 | 3 | 3 |
| SNRNP35 | 0 | 0 | 0 | 1 | 1 | 1 |
| SNRPB | 0 | 5 | 0 | 0 | 5 | 5 |
| SNRPB2 | 1 | 0 | 0 | 0 | 1 | 0 |
| SNRPC | 20 | 0 | 0 | 0 | 20 | 0 |
| SNTB1 | 0 | 1 | 0 | 0 | 1 | 1 |
| SNTG2 | 0 | 1 | 0 | 2 | 3 | 3 |
| SNUPN | 0 | 0 | 0 | 1 | 1 | 1 |
| SNX14 | 0 | 1 | 0 | 0 | 1 | 1 |
| SNX17 | 0 | 2 | 0 | 0 | 2 | 2 |
| SNX18 | 0 | 1 | 0 | 0 | 1 | 1 |
| SNX19 | 0 | 1 | 0 | 0 | 1 | 1 |
| SNX25 | 0 | 0 | 0 | 1 | 1 | 1 |
| SNX29 | 0 | 1 | 0 | 1 | 2 | 2 |
| SNX31 | 0 | 0 | 0 | 2 | 2 | 2 |
| SNX32 | 0 | 0 | 0 | 1 | 1 | 1 |
| SNX5 | 2 | 0 | 0 | 0 | 2 | 0 |
| SOAT2 | 0 | 3 | 0 | 0 | 3 | 3 |
| SOCS5 | 0 | 1 | 0 | 0 | 1 | 1 |
| SORBS1 | 0 | 3 | 0 | 0 | 3 | 3 |
| SORD | 1 | 0 | 0 | 0 | 1 | 0 |
| SORL1 | 0 | 0 | 0 | 7 | 7 | 7 |
| SOS1 | 0 | 0 | 0 | 10 | 10 | 10 |
| SOS2 | 0 | 0 | 0 | 1 | 1 | 1 |
| SOSTDC1 | 0 | 0 | 0 | 3 | 3 | 3 |
| SOX10 | 0 | 1 | 0 | 0 | 1 | 1 |
| SOX11 | 0 | 0 | 0 | 1 | 1 | 1 |
| SP100 | 1 | 0 | 0 | 0 | 1 | 0 |
| SP110 | 0 | 1 | 0 | 0 | 1 | 1 |
| SPAG16 | 0 | 1 | 0 | 0 | 1 | 1 |
| SPAG17 | 0 | 0 | 0 | 1 | 1 | 1 |
| SPAG4 | 0 | 0 | 0 | 1 | 1 | 1 |
| SPAG8 | 0 | 2 | 0 | 0 | 2 | 2 |
| SPANXN2 | 0 | 0 | 0 | 7 | 7 | 7 |
| SPATA16 | 0 | 2 | 0 | 0 | 2 | 2 |
| SPATA20 | 0 | 0 | 0 | 3 | 3 | 3 |
| SPATA21 | 0 | 1 | 0 | 0 | 1 | 1 |
| SPATA3 | 0 | 0 | 0 | 1 | 1 | 1 |
| SPATA4 | 0 | 0 | 0 | 1 | 1 | 1 |
| SPATA5 | 0 | 1 | 0 | 1 | 2 | 2 |
| SPATA7 | 0 | 1 | 0 | 0 | 1 | 1 |
| SPATA8 | 3 | 0 | 0 | 1 | 4 | 1 |
| SPDYE1 | 0 | 0 | 0 | 2 | 2 | 2 |
| SPDYE3 | 0 | 0 | 0 | 1 | 1 | 1 |
| SPECC1 | 0 | 0 | 0 | 6 | 6 | 6 |
| SPEF2 | 0 | 0 | 0 | 1 | 1 | 1 |
| SPEN | 0 | 0 | 0 | 1 | 1 | 1 |
| SPERT | 0 | 1 | 0 | 0 | 1 | 1 |
| SPG20 | 0 | 0 | 1 | 0 | 1 | 0 |
| SPG7 | 1 | 0 | 0 | 0 | 1 | 0 |
| SPHKAP | 0 | 0 | 0 | 4 | 4 | 4 |
| SPICE1 | 0 | 1 | 0 | 1 | 2 | 2 |
| SPIN2A | 0 | 0 | 0 | 1 | 1 | 1 |
| SPIN3 | 0 | 0 | 0 | 4 | 4 | 4 |
| SPINK14 | 0 | 0 | 0 | 9 | 9 | 9 |
| SPINK7 | 1 | 0 | 0 | 0 | 1 | 0 |
| SPINT4 | 0 | 0 | 0 | 1 | 1 | 1 |
| SPNS3 | 0 | 2 | 0 | 1 | 3 | 3 |
| SPOCD1 | 0 | 0 | 0 | 2 | 2 | 2 |
| SPOCK3 | 0 | 0 | 0 | 1 | 1 | 1 |
| SPON1 | 0 | 1 | 0 | 0 | 1 | 1 |
| SPPL2A | 1 | 0 | 0 | 0 | 1 | 0 |
| SPR | 0 | 1 | 0 | 0 | 1 | 1 |
| SPRED3 | 0 | 1 | 0 | 0 | 1 | 1 |
| SPRR1B | 0 | 0 | 0 | 1 | 1 | 1 |
| SPRR2G | 0 | 0 | 1 | 0 | 1 | 0 |
| SPRY1 | 0 | 0 | 0 | 1 | 1 | 1 |
| SPRYD5 | 1 | 0 | 0 | 0 | 1 | 0 |
| SPTAN1 | 0 | 0 | 0 | 1 | 1 | 1 |
| SPTBN1 | 0 | 0 | 0 | 1 | 1 | 1 |
| SPTBN5 | 0 | 1 | 0 | 2 | 3 | 3 |
| SPTLC1 | 0 | 1 | 0 | 1 | 2 | 2 |
| SPTLC2 | 0 | 0 | 0 | 1 | 1 | 1 |
| SPZ1 | 0 | 0 | 0 | 7 | 7 | 7 |
| SRBD1 | 0 | 1 | 0 | 0 | 1 | 1 |
| SRCAP | 0 | 0 | 0 | 2 | 2 | 2 |
| SRD5A2 | 1 | 0 | 0 | 0 | 1 | 0 |
| SRD5A3 | 0 | 0 | 0 | 3 | 3 | 3 |
| SREBF2 | 0 | 0 | 0 | 1 | 1 | 1 |
| SREK1 | 0 | 0 | 0 | 1 | 1 | 1 |
| SREK1IP1 | 0 | 0 | 0 | 3 | 3 | 3 |
| SRFBP1 | 0 | 0 | 0 | 4 | 4 | 4 |
| SRGAP2 | 0 | 4 | 0 | 0 | 4 | 4 |
| SRGAP3 | 0 | 0 | 0 | 1 | 1 | 1 |
| SRL | 0 | 1 | 0 | 0 | 1 | 1 |
| SRP54 | 0 | 0 | 0 | 1 | 1 | 1 |
| SRPK2 | 1 | 0 | 0 | 0 | 1 | 0 |
| SRPRB | 0 | 0 | 0 | 1 | 1 | 1 |
| SRPX | 0 | 0 | 0 | 1 | 1 | 1 |
| SRPX2 | 0 | 2 | 0 | 0 | 2 | 2 |
| SRR | 0 | 0 | 0 | 1 | 1 | 1 |
| SRRD | 1 | 0 | 0 | 1 | 2 | 1 |
| SRRM3 | 0 | 0 | 0 | 7 | 7 | 7 |
| SRRM5 | 0 | 0 | 0 | 1 | 1 | 1 |
| SRRT | 0 | 0 | 0 | 2 | 2 | 2 |
| SS18 | 0 | 4 | 0 | 0 | 4 | 4 |
| SSH1 | 0 | 0 | 0 | 1 | 1 | 1 |
| SSH3 | 0 | 0 | 0 | 1 | 1 | 1 |
| SSPO | 0 | 2 | 0 | 26 | 28 | 28 |
| SSTR1 | 0 | 0 | 0 | 1 | 1 | 1 |
| SSU72 | 0 | 1 | 0 | 0 | 1 | 1 |
| SSX5 | 1 | 0 | 0 | 0 | 1 | 0 |
| ST14 | 0 | 0 | 0 | 1 | 1 | 1 |
| ST18 | 0 | 0 | 0 | 1 | 1 | 1 |
| ST20 | 0 | 0 | 0 | 3 | 3 | 3 |
| ST3GAL2 | 0 | 1 | 0 | 1 | 2 | 2 |
| ST3GAL5 | 0 | 0 | 0 | 1 | 1 | 1 |
| ST3GAL6 | 0 | 0 | 0 | 1 | 1 | 1 |
| ST6GALNAC1 | 1 | 0 | 0 | 1 | 2 | 1 |
| ST6GALNAC6 | 0 | 0 | 0 | 1 | 1 | 1 |
| ST7L | 0 | 0 | 0 | 1 | 1 | 1 |
| STAB1 | 0 | 0 | 0 | 2 | 2 | 2 |
| STAB2 | 0 | 0 | 0 | 2 | 2 | 2 |
| STAC | 0 | 0 | 0 | 1 | 1 | 1 |
| STAC3 | 0 | 0 | 0 | 1 | 1 | 1 |
| STAG1 | 0 | 2 | 0 | 0 | 2 | 2 |
| STAG3 | 0 | 5 | 0 | 1 | 6 | 6 |
| STAP2 | 0 | 1 | 0 | 0 | 1 | 1 |
| STARD13 | 0 | 0 | 0 | 7 | 7 | 7 |
| STARD4 | 14 | 2 | 0 | 0 | 16 | 2 |
| STARD6 | 0 | 18 | 0 | 0 | 18 | 18 |
| STAT2 | 0 | 0 | 0 | 1 | 1 | 1 |
| STAT3 | 0 | 0 | 0 | 1 | 1 | 1 |
| STAT4 | 0 | 0 | 0 | 1 | 1 | 1 |
| STAT5A | 0 | 0 | 0 | 1 | 1 | 1 |
| STATH | 1 | 0 | 0 | 0 | 1 | 0 |
| STBD1 | 0 | 1 | 0 | 3 | 4 | 4 |
| STEAP4 | 0 | 3 | 0 | 0 | 3 | 3 |
| STIL | 0 | 0 | 0 | 1 | 1 | 1 |
| STIP1 | 2 | 0 | 0 | 0 | 2 | 0 |
| STK19 | 0 | 1 | 0 | 0 | 1 | 1 |
| STK31 | 0 | 2 | 0 | 0 | 2 | 2 |
| STK32A | 0 | 1 | 0 | 0 | 1 | 1 |
| STK32B | 0 | 0 | 0 | 1 | 1 | 1 |
| STK33 | 0 | 0 | 0 | 12 | 12 | 12 |
| STK36 | 0 | 0 | 0 | 1 | 1 | 1 |
| STMN3 | 0 | 0 | 0 | 1 | 1 | 1 |
| STOML3 | 0 | 1 | 0 | 0 | 1 | 1 |
| STON1-GTF2A1L | 0 | 0 | 0 | 2 | 2 | 2 |
| STOX1 | 0 | 2 | 0 | 0 | 2 | 2 |
| STRC | 1 | 0 | 0 | 0 | 1 | 0 |
| STRN4 | 7 | 0 | 0 | 1 | 8 | 1 |
| STX18 | 3 | 0 | 0 | 0 | 3 | 0 |
| STX3 | 0 | 1 | 0 | 0 | 1 | 1 |
| STX5 | 0 | 0 | 0 | 1 | 1 | 1 |
| STX6 | 1 | 1 | 0 | 0 | 2 | 1 |
| STXBP3 | 0 | 1 | 0 | 0 | 1 | 1 |
| STXBP5L | 0 | 1 | 0 | 0 | 1 | 1 |
| STYXL1 | 0 | 0 | 0 | 1 | 1 | 1 |
| SULF1 | 0 | 0 | 0 | 2 | 2 | 2 |
| SULT1A1 | 1 | 0 | 0 | 0 | 1 | 0 |
| SULT1C2 | 0 | 1 | 0 | 0 | 1 | 1 |
| SULT1C3 | 0 | 10 | 0 | 0 | 10 | 10 |
| SULT1C4 | 0 | 7 | 0 | 0 | 7 | 7 |
| SULT1E1 | 0 | 0 | 0 | 1 | 1 | 1 |
| SULT2A1 | 0 | 1 | 0 | 0 | 1 | 1 |
| SUMF2 | 0 | 0 | 0 | 1 | 1 | 1 |
| SUN1 | 0 | 0 | 0 | 2 | 2 | 2 |
| SUN2 | 0 | 1 | 0 | 0 | 1 | 1 |
| SUOX | 0 | 0 | 1 | 0 | 1 | 0 |
| SUPT3H | 0 | 0 | 0 | 1 | 1 | 1 |
| SUPT6H | 0 | 8 | 0 | 0 | 8 | 8 |
| SUSD2 | 0 | 3 | 0 | 4 | 7 | 7 |
| SUSD3 | 1 | 1 | 0 | 0 | 2 | 1 |
| SUV420H1 | 0 | 0 | 0 | 1 | 1 | 1 |
| SUZ12 | 0 | 0 | 0 | 1 | 1 | 1 |
| SV2A | 0 | 0 | 0 | 1 | 1 | 1 |
| SVEP1 | 0 | 1 | 0 | 0 | 1 | 1 |
| SVIL | 0 | 1 | 0 | 1 | 2 | 2 |
| SVOPL | 0 | 6 | 0 | 0 | 6 | 6 |
| SYCP1 | 0 | 0 | 0 | 2 | 2 | 2 |
| SYCP2 | 1 | 2 | 0 | 0 | 3 | 2 |
| SYCP2L | 0 | 1 | 0 | 1 | 2 | 2 |
| SYDE2 | 0 | 0 | 0 | 2 | 2 | 2 |
| SYN3 | 0 | 1 | 0 | 0 | 1 | 1 |
| SYNC | 0 | 0 | 0 | 1 | 1 | 1 |
| SYNE1 | 0 | 18 | 0 | 6 | 24 | 24 |
| SYNE2 | 0 | 14 | 0 | 0 | 14 | 14 |
| SYNGR4 | 1 | 0 | 0 | 1 | 2 | 1 |
| SYNM | 0 | 3 | 0 | 1 | 4 | 4 |
| SYNPO2L | 0 | 0 | 0 | 2 | 2 | 2 |
| SYT12 | 0 | 0 | 0 | 1 | 1 | 1 |
| SYT14 | 0 | 0 | 0 | 2 | 2 | 2 |
| SYT15 | 1 | 0 | 0 | 0 | 1 | 0 |
| SYT17 | 0 | 2 | 0 | 0 | 2 | 2 |
| SYT5 | 0 | 0 | 0 | 1 | 1 | 1 |
| SYTL1 | 1 | 0 | 0 | 0 | 1 | 0 |
| SYTL2 | 1 | 0 | 0 | 0 | 1 | 0 |
| SYTL3 | 0 | 0 | 1 | 0 | 1 | 0 |
| SZT2 | 1 | 0 | 0 | 5 | 6 | 5 |
| TAAR2 | 0 | 19 | 0 | 0 | 19 | 19 |
| TAAR8 | 0 | 0 | 0 | 1 | 1 | 1 |
| TAAR9 | 0 | 0 | 0 | 1 | 1 | 1 |
| TAC4 | 0 | 0 | 0 | 4 | 4 | 4 |
| TACC2 | 0 | 1 | 0 | 1 | 2 | 2 |
| TACR2 | 0 | 0 | 0 | 1 | 1 | 1 |
| TADA2B | 0 | 0 | 0 | 2 | 2 | 2 |
| TAF1A | 4 | 0 | 0 | 0 | 4 | 0 |
| TAF1B | 0 | 0 | 0 | 1 | 1 | 1 |
| TAF1C | 0 | 1 | 0 | 0 | 1 | 1 |
| TAF2 | 1 | 0 | 0 | 0 | 1 | 0 |
| TAF3 | 0 | 1 | 0 | 0 | 1 | 1 |
| TAF6 | 0 | 0 | 0 | 3 | 3 | 3 |
| TAF8 | 0 | 1 | 0 | 0 | 1 | 1 |
| TALDO1 | 0 | 0 | 0 | 1 | 1 | 1 |
| TANC2 | 0 | 11 | 0 | 0 | 11 | 11 |
| TAOK3 | 0 | 0 | 0 | 1 | 1 | 1 |
| TARBP1 | 1 | 1 | 0 | 1 | 3 | 2 |
| TARBP2 | 1 | 0 | 0 | 0 | 1 | 0 |
| TARSL2 | 0 | 0 | 0 | 2 | 2 | 2 |
| TAS1R2 | 0 | 1 | 0 | 0 | 1 | 1 |
| TAS2R14 | 0 | 0 | 0 | 1 | 1 | 1 |
| TAS2R16 | 0 | 0 | 0 | 3 | 3 | 3 |
| TAS2R19 | 0 | 7 | 0 | 0 | 7 | 7 |
| TAS2R30 | 0 | 1 | 0 | 0 | 1 | 1 |
| TAS2R31 | 0 | 0 | 0 | 1 | 1 | 1 |
| TAS2R41 | 0 | 1 | 0 | 1 | 2 | 2 |
| TAS2R46 | 0 | 1 | 0 | 7 | 8 | 8 |
| TAS2R60 | 0 | 0 | 3 | 0 | 3 | 0 |
| TAS2R7 | 0 | 1 | 0 | 0 | 1 | 1 |
| TAS2R8 | 0 | 2 | 0 | 17 | 19 | 19 |
| TATDN3 | 19 | 0 | 0 | 0 | 19 | 0 |
| TAZ | 3 | 0 | 0 | 0 | 3 | 0 |
| TBC1D15 | 0 | 1 | 0 | 0 | 1 | 1 |
| TBC1D17 | 0 | 0 | 0 | 1 | 1 | 1 |
| TBC1D24 | 0 | 0 | 0 | 1 | 1 | 1 |
| TBC1D28 | 2 | 1 | 0 | 0 | 3 | 1 |
| TBC1D29 | 19 | 0 | 0 | 0 | 19 | 0 |
| TBC1D3H | 0 | 0 | 0 | 1 | 1 | 1 |
| TBC1D5 | 0 | 1 | 0 | 0 | 1 | 1 |
| TBC1D8 | 0 | 0 | 0 | 2 | 2 | 2 |
| TBC1D9B | 0 | 1 | 0 | 0 | 1 | 1 |
| TBCC | 0 | 0 | 0 | 2 | 2 | 2 |
| TBCCD1 | 0 | 0 | 0 | 2 | 2 | 2 |
| TBCD | 0 | 1 | 0 | 1 | 2 | 2 |
| TBCK | 1 | 0 | 0 | 3 | 4 | 3 |
| TBKBP1 | 0 | 1 | 0 | 0 | 1 | 1 |
| TBR1 | 0 | 0 | 0 | 2 | 2 | 2 |
| TBX10 | 0 | 0 | 0 | 1 | 1 | 1 |
| TBX19 | 0 | 1 | 0 | 0 | 1 | 1 |
| TBX6 | 0 | 0 | 0 | 1 | 1 | 1 |
| TCEANC | 0 | 1 | 0 | 0 | 1 | 1 |
| TCEB3 | 0 | 0 | 0 | 1 | 1 | 1 |
| TCEB3B | 0 | 1 | 0 | 1 | 2 | 2 |
| TCEB3CL | 0 | 0 | 0 | 23 | 23 | 23 |
| TCF12 | 0 | 2 | 0 | 0 | 2 | 2 |
| TCF3 | 0 | 0 | 0 | 1 | 1 | 1 |
| TCHH | 0 | 7 | 0 | 2 | 9 | 9 |
| TCHHL1 | 0 | 5 | 0 | 1 | 6 | 6 |
| TCHP | 1 | 0 | 0 | 0 | 1 | 0 |
| TCIRG1 | 1 | 0 | 0 | 0 | 1 | 0 |
| TCN1 | 0 | 0 | 0 | 1 | 1 | 1 |
| TCN2 | 0 | 2 | 0 | 0 | 2 | 2 |
| TCOF1 | 1 | 0 | 0 | 0 | 1 | 0 |
| TCP1 | 0 | 0 | 0 | 1 | 1 | 1 |
| TCP10L2 | 0 | 23 | 0 | 0 | 23 | 23 |
| TCP11 | 1 | 0 | 0 | 0 | 1 | 0 |
| TCP11L2 | 1 | 0 | 0 | 0 | 1 | 0 |
| TCTN1 | 0 | 0 | 0 | 2 | 2 | 2 |
| TDG | 0 | 0 | 0 | 3 | 3 | 3 |
| TDP1 | 1 | 0 | 0 | 0 | 1 | 0 |
| TDRD5 | 0 | 0 | 0 | 1 | 1 | 1 |
| TDRD6 | 0 | 4 | 0 | 0 | 4 | 4 |
| TEAD2 | 0 | 0 | 0 | 4 | 4 | 4 |
| TECTA | 0 | 0 | 0 | 1 | 1 | 1 |
| TEKT1 | 1 | 0 | 0 | 2 | 3 | 2 |
| TEKT3 | 0 | 1 | 0 | 0 | 1 | 1 |
| TEKT4 | 0 | 2 | 0 | 1 | 3 | 3 |
| TEN1 | 0 | 4 | 0 | 0 | 4 | 4 |
| TENC1 | 0 | 0 | 0 | 6 | 6 | 6 |
| TEP1 | 0 | 0 | 0 | 2 | 2 | 2 |
| TERF2IP | 0 | 1 | 0 | 0 | 1 | 1 |
| TEX101 | 1 | 0 | 0 | 0 | 1 | 0 |
| TEX13B | 1 | 5 | 0 | 0 | 6 | 5 |
| TEX14 | 3 | 1 | 0 | 0 | 4 | 1 |
| TEX15 | 0 | 3 | 0 | 1 | 4 | 4 |
| TEX2 | 0 | 0 | 0 | 1 | 1 | 1 |
| TF | 1 | 0 | 0 | 2 | 3 | 2 |
| TFDP1 | 1 | 0 | 0 | 0 | 1 | 0 |
| TFDP3 | 0 | 3 | 0 | 0 | 3 | 3 |
| TFR2 | 0 | 1 | 0 | 4 | 5 | 5 |
| TGFBR2 | 0 | 0 | 0 | 14 | 14 | 14 |
| TGM1 | 1 | 0 | 0 | 0 | 1 | 0 |
| TGM3 | 0 | 0 | 0 | 14 | 14 | 14 |
| TGM4 | 0 | 5 | 0 | 0 | 5 | 5 |
| TGM5 | 0 | 0 | 0 | 1 | 1 | 1 |
| TGM6 | 0 | 0 | 0 | 3 | 3 | 3 |
| TGM7 | 1 | 0 | 0 | 0 | 1 | 0 |
| TGOLN2 | 0 | 0 | 0 | 1 | 1 | 1 |
| TGS1 | 0 | 3 | 0 | 0 | 3 | 3 |
| TH1L | 0 | 0 | 0 | 1 | 1 | 1 |
| THADA | 0 | 1 | 0 | 2 | 3 | 3 |
| THAP5 | 0 | 0 | 0 | 1 | 1 | 1 |
| THAP6 | 0 | 0 | 0 | 1 | 1 | 1 |
| THAP9 | 0 | 0 | 0 | 1 | 1 | 1 |
| THBS1 | 0 | 0 | 0 | 1 | 1 | 1 |
| THBS2 | 0 | 0 | 0 | 1 | 1 | 1 |
| THNSL1 | 0 | 1 | 0 | 1 | 2 | 2 |
| THNSL2 | 2 | 0 | 0 | 3 | 5 | 3 |
| THOC5 | 1 | 0 | 0 | 0 | 1 | 0 |
| THRAP3 | 0 | 2 | 0 | 0 | 2 | 2 |
| THSD1 | 0 | 0 | 0 | 2 | 2 | 2 |
| THSD4 | 0 | 0 | 0 | 1 | 1 | 1 |
| THSD7A | 1 | 0 | 0 | 0 | 1 | 0 |
| THSD7B | 16 | 1 | 0 | 0 | 17 | 1 |
| THUMPD2 | 1 | 0 | 0 | 0 | 1 | 0 |
| THY1 | 0 | 0 | 0 | 1 | 1 | 1 |
| TIAL1 | 0 | 16 | 0 | 0 | 16 | 16 |
| TIAM1 | 0 | 0 | 0 | 1 | 1 | 1 |
| TIAM2 | 1 | 1 | 0 | 0 | 2 | 1 |
| TICAM2 | 0 | 0 | 0 | 1 | 1 | 1 |
| TIE1 | 0 | 4 | 0 | 8 | 12 | 12 |
| TIGD4 | 0 | 3 | 0 | 1 | 4 | 4 |
| TIGD6 | 0 | 0 | 0 | 12 | 12 | 12 |
| TIMD4 | 0 | 0 | 0 | 2 | 2 | 2 |
| TINAG | 0 | 1 | 0 | 0 | 1 | 1 |
| TIPIN | 0 | 1 | 0 | 0 | 1 | 1 |
| TJAP1 | 0 | 1 | 0 | 0 | 1 | 1 |
| TK2 | 0 | 0 | 0 | 1 | 1 | 1 |
| TKTL2 | 0 | 0 | 0 | 1 | 1 | 1 |
| TLCD1 | 0 | 0 | 0 | 2 | 2 | 2 |
| TLE1 | 0 | 1 | 0 | 0 | 1 | 1 |
| TLE2 | 0 | 0 | 0 | 1 | 1 | 1 |
| TLE3 | 0 | 0 | 0 | 1 | 1 | 1 |
| TLN2 | 0 | 0 | 0 | 1 | 1 | 1 |
| TLR1 | 0 | 0 | 0 | 2 | 2 | 2 |
| TLR10 | 0 | 1 | 0 | 1 | 2 | 2 |
| TLR2 | 0 | 3 | 0 | 0 | 3 | 3 |
| TLR5 | 0 | 1 | 0 | 0 | 1 | 1 |
| TLR7 | 0 | 4 | 0 | 0 | 4 | 4 |
| TM4SF4 | 0 | 0 | 0 | 1 | 1 | 1 |
| TM7SF2 | 2 | 0 | 0 | 0 | 2 | 0 |
| TMBIM4 | 0 | 0 | 0 | 3 | 3 | 3 |
| TMC2 | 5 | 1 | 0 | 0 | 6 | 1 |
| TMC3 | 1 | 11 | 0 | 1 | 13 | 12 |
| TMC7 | 0 | 4 | 0 | 0 | 4 | 4 |
| TMCO2 | 0 | 0 | 0 | 1 | 1 | 1 |
| TMCO3 | 0 | 9 | 0 | 0 | 9 | 9 |
| TMCO4 | 0 | 0 | 0 | 1 | 1 | 1 |
| TMED3 | 2 | 0 | 0 | 0 | 2 | 0 |
| TMED4 | 1 | 0 | 0 | 0 | 1 | 0 |
| TMEM104 | 3 | 1 | 0 | 0 | 4 | 1 |
| TMEM115 | 0 | 0 | 0 | 1 | 1 | 1 |
| TMEM120A | 0 | 0 | 4 | 0 | 4 | 0 |
| TMEM132B | 0 | 2 | 0 | 0 | 2 | 2 |
| TMEM132E | 0 | 0 | 0 | 1 | 1 | 1 |
| TMEM133 | 0 | 0 | 0 | 2 | 2 | 2 |
| TMEM144 | 1 | 0 | 0 | 0 | 1 | 0 |
| TMEM145 | 0 | 0 | 0 | 2 | 2 | 2 |
| TMEM146 | 1 | 1 | 0 | 0 | 2 | 1 |
| TMEM150A | 0 | 0 | 0 | 1 | 1 | 1 |
| TMEM150C | 0 | 3 | 0 | 4 | 7 | 7 |
| TMEM159 | 0 | 0 | 0 | 2 | 2 | 2 |
| TMEM161B | 0 | 0 | 0 | 1 | 1 | 1 |
| TMEM168 | 0 | 1 | 0 | 0 | 1 | 1 |
| TMEM170A | 0 | 0 | 0 | 1 | 1 | 1 |
| TMEM171 | 0 | 1 | 0 | 0 | 1 | 1 |
| TMEM176B | 1 | 1 | 0 | 0 | 2 | 1 |
| TMEM177 | 0 | 9 | 0 | 1 | 10 | 10 |
| TMEM179B | 0 | 1 | 0 | 2 | 3 | 3 |
| TMEM18 | 1 | 0 | 0 | 0 | 1 | 0 |
| TMEM180 | 0 | 0 | 0 | 3 | 3 | 3 |
| TMEM194A | 0 | 0 | 0 | 1 | 1 | 1 |
| TMEM198 | 0 | 0 | 0 | 2 | 2 | 2 |
| TMEM200A | 0 | 0 | 0 | 2 | 2 | 2 |
| TMEM206 | 0 | 0 | 0 | 3 | 3 | 3 |
| TMEM209 | 2 | 0 | 0 | 0 | 2 | 0 |
| TMEM214 | 0 | 1 | 0 | 1 | 2 | 2 |
| TMEM27 | 0 | 1 | 0 | 0 | 1 | 1 |
| TMEM31 | 0 | 0 | 0 | 1 | 1 | 1 |
| TMEM38A | 0 | 0 | 0 | 1 | 1 | 1 |
| TMEM44 | 1 | 0 | 0 | 0 | 1 | 0 |
| TMEM48 | 0 | 0 | 0 | 3 | 3 | 3 |
| TMEM54 | 0 | 0 | 0 | 2 | 2 | 2 |
| TMEM59 | 1 | 1 | 0 | 0 | 2 | 1 |
| TMEM60 | 0 | 0 | 0 | 1 | 1 | 1 |
| TMEM61 | 0 | 1 | 0 | 0 | 1 | 1 |
| TMEM62 | 0 | 0 | 0 | 2 | 2 | 2 |
| TMEM63B | 0 | 0 | 0 | 1 | 1 | 1 |
| TMEM63C | 0 | 0 | 0 | 1 | 1 | 1 |
| TMEM71 | 0 | 0 | 0 | 1 | 1 | 1 |
| TMEM72 | 0 | 1 | 0 | 0 | 1 | 1 |
| TMEM79 | 0 | 0 | 0 | 1 | 1 | 1 |
| TMEM81 | 0 | 2 | 0 | 0 | 2 | 2 |
| TMEM82 | 0 | 0 | 0 | 2 | 2 | 2 |
| TMEM85 | 0 | 0 | 0 | 1 | 1 | 1 |
| TMEM87A | 1 | 0 | 0 | 0 | 1 | 0 |
| TMEM87B | 0 | 0 | 0 | 1 | 1 | 1 |
| TMEM8C | 0 | 0 | 0 | 1 | 1 | 1 |
| TMEM9 | 0 | 0 | 0 | 5 | 5 | 5 |
| TMEM97 | 0 | 0 | 1 | 2 | 3 | 2 |
| TMEM99 | 0 | 0 | 0 | 1 | 1 | 1 |
| TMIE | 1 | 0 | 0 | 0 | 1 | 0 |
| TMLHE | 0 | 2 | 0 | 0 | 2 | 2 |
| TMOD2 | 0 | 0 | 0 | 1 | 1 | 1 |
| TMOD3 | 1 | 2 | 0 | 0 | 3 | 2 |
| TMPRSS11A | 0 | 0 | 0 | 1 | 1 | 1 |
| TMPRSS11B | 1 | 2 | 0 | 0 | 3 | 2 |
| TMPRSS11D | 0 | 1 | 0 | 0 | 1 | 1 |
| TMPRSS12 | 0 | 1 | 0 | 0 | 1 | 1 |
| TMPRSS13 | 0 | 0 | 0 | 6 | 6 | 6 |
| TMPRSS15 | 1 | 2 | 0 | 0 | 3 | 2 |
| TMPRSS3 | 0 | 0 | 0 | 1 | 1 | 1 |
| TMPRSS4 | 0 | 1 | 0 | 0 | 1 | 1 |
| TMPRSS6 | 0 | 0 | 0 | 1 | 1 | 1 |
| TMPRSS7 | 3 | 2 | 0 | 0 | 5 | 2 |
| TMPRSS9 | 0 | 0 | 0 | 1 | 1 | 1 |
| TMTC1 | 0 | 0 | 0 | 2 | 2 | 2 |
| TNC | 1 | 0 | 0 | 0 | 1 | 0 |
| TNF | 0 | 0 | 0 | 1 | 1 | 1 |
| TNFAIP3 | 0 | 1 | 0 | 0 | 1 | 1 |
| TNFAIP6 | 0 | 0 | 1 | 0 | 1 | 0 |
| TNFAIP8L3 | 0 | 1 | 0 | 0 | 1 | 1 |
| TNFRSF10A | 0 | 0 | 2 | 0 | 2 | 0 |
| TNFRSF10B | 2 | 0 | 0 | 0 | 2 | 0 |
| TNFRSF10C | 0 | 0 | 0 | 3 | 3 | 3 |
| TNFRSF10D | 0 | 1 | 0 | 0 | 1 | 1 |
| TNFRSF11B | 0 | 1 | 0 | 0 | 1 | 1 |
| TNFRSF13B | 0 | 0 | 0 | 2 | 2 | 2 |
| TNFRSF1A | 0 | 0 | 0 | 1 | 1 | 1 |
| TNFRSF25 | 1 | 0 | 0 | 0 | 1 | 0 |
| TNFRSF8 | 0 | 0 | 0 | 1 | 1 | 1 |
| TNFSF15 | 0 | 0 | 0 | 4 | 4 | 4 |
| TNFSF18 | 0 | 3 | 0 | 2 | 5 | 5 |
| TNIP3 | 1 | 0 | 0 | 0 | 1 | 0 |
| TNK1 | 16 | 1 | 0 | 1 | 18 | 2 |
| TNKS | 1 | 1 | 0 | 0 | 2 | 1 |
| TNNC2 | 0 | 0 | 0 | 1 | 1 | 1 |
| TNNI2 | 0 | 0 | 1 | 0 | 1 | 0 |
| TNNT2 | 5 | 0 | 0 | 0 | 5 | 0 |
| TNRC18 | 0 | 0 | 0 | 1 | 1 | 1 |
| TNRC6A | 1 | 0 | 0 | 0 | 1 | 0 |
| TNRC6C | 0 | 0 | 0 | 1 | 1 | 1 |
| TNS1 | 0 | 1 | 0 | 0 | 1 | 1 |
| TNS4 | 8 | 0 | 0 | 0 | 8 | 0 |
| TOB1 | 0 | 0 | 0 | 1 | 1 | 1 |
| TOE1 | 0 | 0 | 0 | 1 | 1 | 1 |
| TOM1 | 0 | 0 | 0 | 17 | 17 | 17 |
| TOM1L1 | 0 | 0 | 0 | 2 | 2 | 2 |
| TONSL | 0 | 0 | 0 | 1 | 1 | 1 |
| TOP2A | 0 | 1 | 0 | 11 | 12 | 12 |
| TOP2B | 1 | 0 | 0 | 0 | 1 | 0 |
| TOP3B | 0 | 0 | 0 | 1 | 1 | 1 |
| TOPORS | 0 | 0 | 0 | 1 | 1 | 1 |
| TOR1AIP1 | 1 | 0 | 0 | 2 | 3 | 2 |
| TOR1AIP2 | 0 | 0 | 0 | 1 | 1 | 1 |
| TOR3A | 0 | 0 | 0 | 1 | 1 | 1 |
| TP53AIP1 | 0 | 1 | 0 | 5 | 6 | 6 |
| TP53BP1 | 0 | 0 | 0 | 1 | 1 | 1 |
| TP63 | 1 | 0 | 0 | 0 | 1 | 0 |
| TPCN1 | 0 | 0 | 0 | 1 | 1 | 1 |
| TPCN2 | 0 | 0 | 0 | 1 | 1 | 1 |
| TPD52L3 | 0 | 0 | 0 | 2 | 2 | 2 |
| TPH2 | 0 | 0 | 0 | 3 | 3 | 3 |
| TPP1 | 1 | 0 | 0 | 0 | 1 | 0 |
| TPPP2 | 0 | 4 | 0 | 1 | 5 | 5 |
| TPR | 0 | 0 | 0 | 1 | 1 | 1 |
| TPRG1 | 10 | 0 | 0 | 0 | 10 | 0 |
| TPRG1L | 0 | 1 | 0 | 0 | 1 | 1 |
| TPTE | 6 | 4 | 0 | 0 | 10 | 4 |
| TPTE2 | 1 | 1 | 0 | 0 | 2 | 1 |
| TRAF5 | 0 | 0 | 0 | 1 | 1 | 1 |
| TRAFD1 | 0 | 1 | 0 | 0 | 1 | 1 |
| TRAK1 | 0 | 0 | 0 | 4 | 4 | 4 |
| TRAK2 | 0 | 1 | 0 | 1 | 2 | 2 |
| TRANK1 | 0 | 0 | 0 | 5 | 5 | 5 |
| TRAPPC10 | 0 | 0 | 0 | 1 | 1 | 1 |
| TRAPPC8 | 1 | 0 | 0 | 0 | 1 | 0 |
| TRAPPC9 | 0 | 0 | 0 | 1 | 1 | 1 |
| TREH | 1 | 0 | 0 | 0 | 1 | 0 |
| TREML1 | 0 | 1 | 0 | 0 | 1 | 1 |
| TREML4 | 0 | 0 | 0 | 4 | 4 | 4 |
| TREX1 | 0 | 0 | 0 | 1 | 1 | 1 |
| TRIM11 | 0 | 0 | 0 | 1 | 1 | 1 |
| TRIM13 | 0 | 0 | 0 | 1 | 1 | 1 |
| TRIM16 | 0 | 1 | 0 | 0 | 1 | 1 |
| TRIM17 | 0 | 0 | 0 | 1 | 1 | 1 |
| TRIM24 | 0 | 2 | 0 | 0 | 2 | 2 |
| TRIM26 | 0 | 0 | 0 | 1 | 1 | 1 |
| TRIM31 | 0 | 4 | 0 | 2 | 6 | 6 |
| TRIM33 | 1 | 0 | 0 | 0 | 1 | 0 |
| TRIM34 | 0 | 2 | 0 | 0 | 2 | 2 |
| TRIM38 | 0 | 4 | 0 | 0 | 4 | 4 |
| TRIM40 | 11 | 0 | 0 | 0 | 11 | 0 |
| TRIM42 | 0 | 0 | 0 | 4 | 4 | 4 |
| TRIM43 | 0 | 1 | 0 | 0 | 1 | 1 |
| TRIM45 | 0 | 1 | 0 | 0 | 1 | 1 |
| TRIM48 | 1 | 0 | 19 | 0 | 20 | 0 |
| TRIM5 | 0 | 1 | 0 | 0 | 1 | 1 |
| TRIM52 | 0 | 0 | 0 | 1 | 1 | 1 |
| TRIM54 | 1 | 1 | 0 | 0 | 2 | 1 |
| TRIM55 | 1 | 0 | 0 | 0 | 1 | 0 |
| TRIM58 | 0 | 0 | 6 | 1 | 7 | 1 |
| TRIM59 | 0 | 0 | 0 | 9 | 9 | 9 |
| TRIM60 | 0 | 1 | 0 | 0 | 1 | 1 |
| TRIM61 | 0 | 0 | 0 | 1 | 1 | 1 |
| TRIM63 | 1 | 0 | 0 | 0 | 1 | 0 |
| TRIM7 | 0 | 0 | 1 | 1 | 2 | 1 |
| TRIM77P | 0 | 0 | 0 | 1 | 1 | 1 |
| TRIML1 | 0 | 0 | 0 | 1 | 1 | 1 |
| TRIO | 0 | 11 | 0 | 0 | 11 | 11 |
| TRIP11 | 0 | 1 | 0 | 5 | 6 | 6 |
| TRIP13 | 0 | 9 | 0 | 0 | 9 | 9 |
| TRMT11 | 0 | 1 | 0 | 1 | 2 | 2 |
| TRMT2A | 0 | 0 | 0 | 3 | 3 | 3 |
| TRMT5 | 0 | 0 | 0 | 2 | 2 | 2 |
| TRMT61B | 0 | 1 | 0 | 1 | 2 | 2 |
| TRNAU1AP | 0 | 0 | 0 | 2 | 2 | 2 |
| TRNT1 | 0 | 1 | 0 | 2 | 3 | 3 |
| TROVE2 | 0 | 0 | 0 | 1 | 1 | 1 |
| TRPC1 | 0 | 1 | 0 | 2 | 3 | 3 |
| TRPC4AP | 1 | 0 | 0 | 0 | 1 | 0 |
| TRPC6 | 3 | 0 | 0 | 1 | 4 | 1 |
| TRPM2 | 0 | 2 | 0 | 0 | 2 | 2 |
| TRPM3 | 0 | 0 | 0 | 8 | 8 | 8 |
| TRPM4 | 1 | 2 | 0 | 0 | 3 | 2 |
| TRPM8 | 0 | 1 | 0 | 0 | 1 | 1 |
| TRPV3 | 0 | 0 | 0 | 1 | 1 | 1 |
| TRPV4 | 0 | 0 | 0 | 1 | 1 | 1 |
| TRPV5 | 0 | 0 | 0 | 1 | 1 | 1 |
| TRUB2 | 0 | 0 | 0 | 1 | 1 | 1 |
| TSC2 | 0 | 0 | 0 | 2 | 2 | 2 |
| TSC22D1 | 1 | 0 | 0 | 0 | 1 | 0 |
| TSGA10IP | 0 | 1 | 0 | 1 | 2 | 2 |
| TSGA13 | 0 | 1 | 0 | 0 | 1 | 1 |
| TSHR | 0 | 1 | 0 | 0 | 1 | 1 |
| TSHZ1 | 0 | 0 | 0 | 1 | 1 | 1 |
| TSHZ3 | 0 | 0 | 0 | 1 | 1 | 1 |
| TSNARE1 | 0 | 0 | 0 | 1 | 1 | 1 |
| TSNAXIP1 | 0 | 1 | 0 | 0 | 1 | 1 |
| TSPAN10 | 0 | 0 | 0 | 19 | 19 | 19 |
| TSPAN33 | 0 | 1 | 0 | 0 | 1 | 1 |
| TSPAN4 | 0 | 1 | 0 | 0 | 1 | 1 |
| TSPAN8 | 0 | 0 | 1 | 0 | 1 | 0 |
| TSPEAR | 0 | 0 | 0 | 3 | 3 | 3 |
| TSPYL2 | 0 | 0 | 0 | 2 | 2 | 2 |
| TSPYL6 | 0 | 0 | 0 | 1 | 1 | 1 |
| TSR1 | 1 | 0 | 0 | 0 | 1 | 0 |
| TSSC1 | 0 | 1 | 0 | 0 | 1 | 1 |
| TSSK1B | 0 | 0 | 0 | 2 | 2 | 2 |
| TSSK2 | 0 | 1 | 0 | 0 | 1 | 1 |
| TSSK3 | 0 | 1 | 0 | 0 | 1 | 1 |
| TSTD2 | 1 | 1 | 0 | 0 | 2 | 1 |
| TTBK2 | 0 | 1 | 0 | 0 | 1 | 1 |
| TTC12 | 0 | 1 | 0 | 0 | 1 | 1 |
| TTC13 | 0 | 1 | 0 | 0 | 1 | 1 |
| TTC16 | 0 | 0 | 0 | 2 | 2 | 2 |
| TTC18 | 7 | 0 | 0 | 0 | 7 | 0 |
| TTC21A | 0 | 2 | 0 | 1 | 3 | 3 |
| TTC22 | 0 | 1 | 0 | 0 | 1 | 1 |
| TTC23L | 0 | 0 | 0 | 2 | 2 | 2 |
| TTC24 | 0 | 0 | 0 | 8 | 8 | 8 |
| TTC26 | 0 | 1 | 0 | 0 | 1 | 1 |
| TTC29 | 0 | 0 | 0 | 1 | 1 | 1 |
| TTC33 | 0 | 1 | 0 | 0 | 1 | 1 |
| TTC37 | 0 | 1 | 0 | 1 | 2 | 2 |
| TTC7A | 0 | 0 | 0 | 1 | 1 | 1 |
| TTC9B | 0 | 0 | 0 | 1 | 1 | 1 |
| TTC9C | 0 | 10 | 0 | 0 | 10 | 10 |
| TTF2 | 0 | 0 | 0 | 3 | 3 | 3 |
| TTK | 1 | 0 | 1 | 0 | 2 | 0 |
| TTLL11 | 0 | 0 | 0 | 1 | 1 | 1 |
| TTLL12 | 0 | 0 | 0 | 1 | 1 | 1 |
| TTLL3 | 0 | 1 | 0 | 8 | 9 | 9 |
| TTLL4 | 0 | 0 | 0 | 1 | 1 | 1 |
| TTLL5 | 1 | 0 | 0 | 1 | 2 | 1 |
| TTLL6 | 0 | 0 | 0 | 3 | 3 | 3 |
| TTN | 2 | 59 | 3 | 5 | 69 | 64 |
| TTYH3 | 0 | 0 | 0 | 1 | 1 | 1 |
| TUB | 0 | 4 | 0 | 0 | 4 | 4 |
| TUBA3D | 0 | 0 | 0 | 2 | 2 | 2 |
| TUBA3E | 0 | 0 | 0 | 1 | 1 | 1 |
| TUBB1 | 0 | 3 | 0 | 0 | 3 | 3 |
| TUBB2B | 0 | 0 | 0 | 1 | 1 | 1 |
| TUBG1 | 0 | 1 | 0 | 0 | 1 | 1 |
| TUBGCP2 | 0 | 0 | 0 | 1 | 1 | 1 |
| TULP2 | 0 | 6 | 0 | 1 | 7 | 7 |
| TULP3 | 3 | 0 | 0 | 0 | 3 | 0 |
| TULP4 | 0 | 1 | 0 | 0 | 1 | 1 |
| TUSC3 | 0 | 0 | 0 | 1 | 1 | 1 |
| TWISTNB | 0 | 4 | 0 | 1 | 5 | 5 |
| TXLNG | 0 | 0 | 0 | 1 | 1 | 1 |
| TXNDC11 | 0 | 3 | 0 | 0 | 3 | 3 |
| TXNDC16 | 4 | 0 | 0 | 0 | 4 | 0 |
| TXNRD3NB | 0 | 0 | 0 | 3 | 3 | 3 |
| TYK2 | 0 | 0 | 0 | 4 | 4 | 4 |
| TYROBP | 1 | 0 | 0 | 1 | 2 | 1 |
| TYRP1 | 0 | 2 | 0 | 0 | 2 | 2 |
| TYW1B | 0 | 1 | 0 | 0 | 1 | 1 |
| TYW3 | 0 | 0 | 0 | 1 | 1 | 1 |
| U2AF1L4 | 0 | 0 | 0 | 1 | 1 | 1 |
| UBA3 | 0 | 0 | 0 | 1 | 1 | 1 |
| UBA7 | 0 | 1 | 0 | 1 | 2 | 2 |
| UBAP2 | 0 | 0 | 0 | 15 | 15 | 15 |
| UBE2D1 | 7 | 0 | 0 | 0 | 7 | 0 |
| UBE2E2 | 0 | 1 | 0 | 0 | 1 | 1 |
| UBE2U | 0 | 1 | 0 | 0 | 1 | 1 |
| UBE3C | 0 | 0 | 0 | 1 | 1 | 1 |
| UBE4B | 0 | 0 | 0 | 2 | 2 | 2 |
| UBL4B | 0 | 2 | 0 | 0 | 2 | 2 |
| UBN1 | 0 | 0 | 0 | 2 | 2 | 2 |
| UBQLNL | 0 | 3 | 0 | 0 | 3 | 3 |
| UBR2 | 0 | 0 | 0 | 1 | 1 | 1 |
| UBR3 | 0 | 1 | 0 | 0 | 1 | 1 |
| UBR4 | 0 | 0 | 0 | 7 | 7 | 7 |
| UBTF | 0 | 1 | 0 | 0 | 1 | 1 |
| UBXN10 | 0 | 1 | 0 | 0 | 1 | 1 |
| UBXN11 | 0 | 0 | 0 | 22 | 22 | 22 |
| UBXN7 | 0 | 1 | 0 | 0 | 1 | 1 |
| UBXN8 | 0 | 1 | 0 | 1 | 2 | 2 |
| UCK1 | 0 | 0 | 0 | 1 | 1 | 1 |
| UCP3 | 2 | 0 | 0 | 0 | 2 | 0 |
| UFSP1 | 0 | 0 | 1 | 0 | 1 | 0 |
| UGGT1 | 0 | 0 | 0 | 1 | 1 | 1 |
| UGGT2 | 1 | 0 | 0 | 2 | 3 | 2 |
| UGT1A1 | 0 | 0 | 0 | 1 | 1 | 1 |
| UGT1A10 | 0 | 1 | 0 | 0 | 1 | 1 |
| UGT1A3 | 0 | 0 | 0 | 2 | 2 | 2 |
| UGT1A5 | 0 | 0 | 0 | 1 | 1 | 1 |
| UGT1A7 | 1 | 0 | 0 | 0 | 1 | 0 |
| UGT1A9 | 0 | 1 | 0 | 0 | 1 | 1 |
| UGT2B17 | 0 | 0 | 0 | 1 | 1 | 1 |
| UGT2B28 | 0 | 0 | 0 | 3 | 3 | 3 |
| UGT2B4 | 0 | 0 | 0 | 1 | 1 | 1 |
| UGT2B7 | 0 | 1 | 0 | 0 | 1 | 1 |
| UGT8 | 0 | 0 | 0 | 1 | 1 | 1 |
| UHMK1 | 0 | 0 | 0 | 1 | 1 | 1 |
| UHRF1 | 0 | 4 | 0 | 0 | 4 | 4 |
| UHRF1BP1 | 0 | 1 | 0 | 1 | 2 | 2 |
| ULBP3 | 0 | 2 | 0 | 0 | 2 | 2 |
| ULK1 | 0 | 0 | 0 | 1 | 1 | 1 |
| ULK4 | 0 | 1 | 0 | 1 | 2 | 2 |
| UMOD | 0 | 1 | 0 | 0 | 1 | 1 |
| UMODL1 | 1 | 1 | 0 | 0 | 2 | 1 |
| UMPS | 0 | 0 | 0 | 1 | 1 | 1 |
| UNC13A | 14 | 0 | 0 | 0 | 14 | 0 |
| UNC13B | 0 | 0 | 0 | 1 | 1 | 1 |
| UNC13C | 0 | 2 | 0 | 5 | 7 | 7 |
| UNC13D | 2 | 0 | 0 | 1 | 3 | 1 |
| UNC45A | 1 | 0 | 0 | 0 | 1 | 0 |
| UNC5CL | 1 | 0 | 0 | 0 | 1 | 0 |
| UNC80 | 0 | 2 | 0 | 2 | 4 | 4 |
| UNC93A | 9 | 3 | 0 | 0 | 12 | 3 |
| UPB1 | 4 | 0 | 0 | 1 | 5 | 1 |
| UPF1 | 0 | 0 | 0 | 1 | 1 | 1 |
| UPK2 | 1 | 0 | 0 | 0 | 1 | 0 |
| UPK3A | 1 | 2 | 0 | 0 | 3 | 2 |
| UPP2 | 0 | 0 | 0 | 4 | 4 | 4 |
| UQCRB | 0 | 1 | 0 | 0 | 1 | 1 |
| URB1 | 0 | 0 | 0 | 1 | 1 | 1 |
| URB2 | 0 | 0 | 0 | 1 | 1 | 1 |
| USH1C | 0 | 14 | 0 | 1 | 15 | 15 |
| USH2A | 2 | 18 | 0 | 1 | 21 | 19 |
| USHBP1 | 0 | 0 | 0 | 1 | 1 | 1 |
| USP17L2 | 0 | 0 | 0 | 2 | 2 | 2 |
| USP19 | 0 | 0 | 0 | 2 | 2 | 2 |
| USP20 | 0 | 0 | 0 | 1 | 1 | 1 |
| USP21 | 0 | 0 | 0 | 1 | 1 | 1 |
| USP25 | 1 | 0 | 0 | 0 | 1 | 0 |
| USP27X | 0 | 0 | 0 | 1 | 1 | 1 |
| USP29 | 0 | 3 | 0 | 0 | 3 | 3 |
| USP32 | 0 | 1 | 0 | 1 | 2 | 2 |
| USP34 | 1 | 0 | 0 | 1 | 2 | 1 |
| USP35 | 0 | 1 | 0 | 1 | 2 | 2 |
| USP38 | 0 | 0 | 0 | 1 | 1 | 1 |
| USP4 | 0 | 1 | 0 | 0 | 1 | 1 |
| USP40 | 0 | 1 | 1 | 2 | 4 | 3 |
| USP42 | 0 | 0 | 0 | 2 | 2 | 2 |
| USP45 | 11 | 3 | 0 | 1 | 15 | 4 |
| USP50 | 0 | 0 | 0 | 1 | 1 | 1 |
| USP54 | 0 | 1 | 0 | 0 | 1 | 1 |
| USP6 | 1 | 2 | 0 | 1 | 4 | 3 |
| USP9X | 0 | 1 | 0 | 0 | 1 | 1 |
| UTP14C | 0 | 3 | 0 | 1 | 4 | 4 |
| UTP20 | 2 | 0 | 0 | 0 | 2 | 0 |
| UTRN | 1 | 1 | 0 | 7 | 9 | 8 |
| UTS2D | 0 | 1 | 0 | 1 | 2 | 2 |
| VAC14 | 0 | 3 | 0 | 0 | 3 | 3 |
| VARS2 | 0 | 0 | 0 | 2 | 2 | 2 |
| VAV3 | 0 | 1 | 0 | 0 | 1 | 1 |
| VAX1 | 0 | 0 | 0 | 1 | 1 | 1 |
| VAX2 | 0 | 1 | 0 | 0 | 1 | 1 |
| VCL | 0 | 0 | 2 | 0 | 2 | 0 |
| VCPIP1 | 0 | 0 | 0 | 1 | 1 | 1 |
| VEGFB | 0 | 0 | 0 | 1 | 1 | 1 |
| VENTX | 0 | 1 | 0 | 0 | 1 | 1 |
| VEPH1 | 0 | 1 | 0 | 0 | 1 | 1 |
| VGLL1 | 0 | 6 | 0 | 0 | 6 | 6 |
| VGLL3 | 0 | 0 | 0 | 1 | 1 | 1 |
| VIL1 | 9 | 0 | 0 | 2 | 11 | 2 |
| VILL | 0 | 1 | 0 | 1 | 2 | 2 |
| VIM | 0 | 0 | 4 | 0 | 4 | 0 |
| VIP | 0 | 1 | 0 | 1 | 2 | 2 |
| VIT | 0 | 13 | 0 | 1 | 14 | 14 |
| VN1R4 | 0 | 0 | 0 | 10 | 10 | 10 |
| VNN1 | 0 | 1 | 4 | 0 | 5 | 1 |
| VPRBP | 1 | 0 | 0 | 0 | 1 | 0 |
| VPREB1 | 1 | 0 | 1 | 0 | 2 | 0 |
| VPREB3 | 0 | 0 | 1 | 0 | 1 | 0 |
| VPS11 | 0 | 5 | 0 | 0 | 5 | 5 |
| VPS13A | 0 | 1 | 0 | 0 | 1 | 1 |
| VPS13B | 0 | 1 | 0 | 2 | 3 | 3 |
| VPS13C | 0 | 1 | 0 | 3 | 4 | 4 |
| VPS16 | 0 | 0 | 0 | 1 | 1 | 1 |
| VPS35 | 0 | 1 | 0 | 1 | 2 | 2 |
| VPS39 | 0 | 0 | 0 | 1 | 1 | 1 |
| VPS4A | 0 | 0 | 0 | 1 | 1 | 1 |
| VPS4B | 1 | 0 | 0 | 0 | 1 | 0 |
| VPS8 | 0 | 0 | 0 | 1 | 1 | 1 |
| VRK3 | 0 | 1 | 0 | 0 | 1 | 1 |
| VRTN | 0 | 0 | 0 | 2 | 2 | 2 |
| VSIG10L | 0 | 0 | 0 | 11 | 11 | 11 |
| VSTM1 | 0 | 0 | 1 | 0 | 1 | 0 |
| VWA3A | 1 | 2 | 0 | 0 | 3 | 2 |
| VWA3B | 0 | 1 | 0 | 2 | 3 | 3 |
| VWCE | 0 | 1 | 0 | 0 | 1 | 1 |
| VWDE | 0 | 14 | 0 | 0 | 14 | 14 |
| VWF | 0 | 0 | 0 | 1 | 1 | 1 |
| WASL | 0 | 0 | 0 | 1 | 1 | 1 |
| WBSCR28 | 0 | 2 | 0 | 0 | 2 | 2 |
| WDFY3 | 0 | 0 | 0 | 6 | 6 | 6 |
| WDFY4 | 0 | 1 | 0 | 0 | 1 | 1 |
| WDHD1 | 0 | 1 | 0 | 0 | 1 | 1 |
| WDR11 | 0 | 0 | 0 | 1 | 1 | 1 |
| WDR16 | 1 | 0 | 0 | 1 | 2 | 1 |
| WDR17 | 1 | 0 | 0 | 5 | 6 | 5 |
| WDR18 | 0 | 0 | 0 | 1 | 1 | 1 |
| WDR27 | 9 | 0 | 0 | 7 | 16 | 7 |
| WDR3 | 0 | 1 | 0 | 2 | 3 | 3 |
| WDR31 | 0 | 1 | 0 | 0 | 1 | 1 |
| WDR33 | 1 | 0 | 0 | 0 | 1 | 0 |
| WDR35 | 2 | 1 | 0 | 0 | 3 | 1 |
| WDR49 | 0 | 0 | 0 | 1 | 1 | 1 |
| WDR5B | 0 | 0 | 0 | 1 | 1 | 1 |
| WDR6 | 0 | 0 | 0 | 2 | 2 | 2 |
| WDR62 | 0 | 5 | 0 | 0 | 5 | 5 |
| WDR63 | 1 | 0 | 0 | 0 | 1 | 0 |
| WDR64 | 0 | 0 | 0 | 1 | 1 | 1 |
| WDR65 | 0 | 0 | 0 | 1 | 1 | 1 |
| WDR66 | 1 | 2 | 0 | 0 | 3 | 2 |
| WDR67 | 0 | 1 | 0 | 1 | 2 | 2 |
| WDR72 | 0 | 8 | 0 | 0 | 8 | 8 |
| WDR78 | 0 | 1 | 0 | 3 | 4 | 4 |
| WDR83 | 0 | 0 | 0 | 3 | 3 | 3 |
| WDR85 | 0 | 0 | 0 | 3 | 3 | 3 |
| WDR87 | 0 | 1 | 0 | 0 | 1 | 1 |
| WDR89 | 0 | 1 | 0 | 0 | 1 | 1 |
| WDR91 | 0 | 1 | 0 | 1 | 2 | 2 |
| WDR93 | 1 | 0 | 0 | 1 | 2 | 1 |
| WDR96 | 0 | 3 | 0 | 0 | 3 | 3 |
| WDTC1 | 0 | 0 | 0 | 1 | 1 | 1 |
| WFDC8 | 0 | 0 | 0 | 2 | 2 | 2 |
| WHSC1 | 0 | 0 | 0 | 1 | 1 | 1 |
| WIPI1 | 0 | 1 | 0 | 0 | 1 | 1 |
| WNK1 | 0 | 0 | 0 | 4 | 4 | 4 |
| WNK3 | 0 | 3 | 0 | 0 | 3 | 3 |
| WNK4 | 0 | 0 | 0 | 5 | 5 | 5 |
| WNT10A | 0 | 1 | 0 | 0 | 1 | 1 |
| WNT11 | 0 | 1 | 0 | 0 | 1 | 1 |
| WNT16 | 0 | 0 | 0 | 1 | 1 | 1 |
| WRAP53 | 0 | 0 | 0 | 11 | 11 | 11 |
| WRAP73 | 0 | 1 | 0 | 0 | 1 | 1 |
| WRN | 0 | 2 | 0 | 0 | 2 | 2 |
| WSB2 | 0 | 0 | 0 | 1 | 1 | 1 |
| WT1 | 1 | 0 | 0 | 0 | 1 | 0 |
| WTH3DI | 0 | 0 | 0 | 2 | 2 | 2 |
| WWC1 | 1 | 0 | 0 | 0 | 1 | 0 |
| WWC3 | 0 | 0 | 0 | 4 | 4 | 4 |
| XAF1 | 0 | 2 | 0 | 0 | 2 | 2 |
| XDH | 2 | 0 | 0 | 0 | 2 | 0 |
| XG | 0 | 1 | 0 | 0 | 1 | 1 |
| XIRP1 | 0 | 1 | 0 | 0 | 1 | 1 |
| XK | 0 | 0 | 0 | 1 | 1 | 1 |
| XKR6 | 0 | 3 | 0 | 0 | 3 | 3 |
| XKR8 | 0 | 1 | 0 | 0 | 1 | 1 |
| XKR9 | 0 | 1 | 0 | 0 | 1 | 1 |
| XKRX | 13 | 0 | 0 | 0 | 13 | 0 |
| XPC | 1 | 0 | 0 | 7 | 8 | 7 |
| XPNPEP3 | 0 | 0 | 0 | 2 | 2 | 2 |
| XPO1 | 0 | 0 | 0 | 1 | 1 | 1 |
| XPOT | 0 | 0 | 0 | 2 | 2 | 2 |
| XRRA1 | 0 | 1 | 0 | 1 | 2 | 2 |
| XYLT2 | 1 | 0 | 0 | 0 | 1 | 0 |
| YARS2 | 2 | 0 | 0 | 2 | 4 | 2 |
| YBEY | 0 | 0 | 0 | 1 | 1 | 1 |
| YBX2 | 0 | 0 | 0 | 1 | 1 | 1 |
| YEATS2 | 0 | 0 | 0 | 4 | 4 | 4 |
| YES1 | 0 | 0 | 0 | 5 | 5 | 5 |
| YIF1B | 0 | 0 | 0 | 1 | 1 | 1 |
| YIPF1 | 2 | 0 | 0 | 1 | 3 | 1 |
| YIPF7 | 0 | 1 | 0 | 0 | 1 | 1 |
| YSK4 | 1 | 0 | 0 | 0 | 1 | 0 |
| YTHDF1 | 0 | 2 | 0 | 1 | 3 | 3 |
| YTHDF2 | 0 | 0 | 0 | 1 | 1 | 1 |
| YTHDF3 | 0 | 0 | 0 | 1 | 1 | 1 |
| YWHAB | 0 | 0 | 0 | 1 | 1 | 1 |
| YY1AP1 | 0 | 0 | 0 | 1 | 1 | 1 |
| ZACN | 0 | 0 | 0 | 1 | 1 | 1 |
| ZAN | 5 | 23 | 0 | 7 | 35 | 30 |
| ZBBX | 1 | 0 | 0 | 1 | 2 | 1 |
| ZBP1 | 0 | 3 | 0 | 1 | 4 | 4 |
| ZBTB2 | 0 | 0 | 0 | 2 | 2 | 2 |
| ZBTB37 | 0 | 0 | 0 | 1 | 1 | 1 |
| ZBTB39 | 0 | 0 | 0 | 1 | 1 | 1 |
| ZBTB4 | 0 | 0 | 0 | 3 | 3 | 3 |
| ZBTB41 | 0 | 1 | 0 | 0 | 1 | 1 |
| ZBTB45 | 0 | 0 | 0 | 4 | 4 | 4 |
| ZBTB7C | 0 | 0 | 0 | 1 | 1 | 1 |
| ZBTB9 | 0 | 1 | 0 | 0 | 1 | 1 |
| ZC3H10 | 0 | 0 | 0 | 1 | 1 | 1 |
| ZC3H11A | 0 | 1 | 0 | 0 | 1 | 1 |
| ZC3H12A | 0 | 0 | 0 | 1 | 1 | 1 |
| ZC3H12C | 0 | 0 | 0 | 1 | 1 | 1 |
| ZC3H12D | 0 | 0 | 0 | 1 | 1 | 1 |
| ZC3H14 | 0 | 0 | 0 | 1 | 1 | 1 |
| ZC3H15 | 0 | 0 | 0 | 1 | 1 | 1 |
| ZC3H18 | 0 | 0 | 0 | 2 | 2 | 2 |
| ZC3H6 | 0 | 0 | 0 | 2 | 2 | 2 |
| ZCCHC13 | 0 | 0 | 0 | 1 | 1 | 1 |
| ZCCHC16 | 0 | 1 | 0 | 0 | 1 | 1 |
| ZCCHC18 | 0 | 0 | 0 | 1 | 1 | 1 |
| ZCCHC4 | 0 | 0 | 1 | 3 | 4 | 3 |
| ZCRB1 | 0 | 1 | 0 | 1 | 2 | 2 |
| ZCWPW2 | 0 | 0 | 0 | 1 | 1 | 1 |
| ZDBF2 | 0 | 0 | 0 | 1 | 1 | 1 |
| ZDHHC1 | 0 | 0 | 0 | 1 | 1 | 1 |
| ZDHHC13 | 1 | 0 | 0 | 0 | 1 | 0 |
| ZDHHC4 | 1 | 0 | 0 | 0 | 1 | 0 |
| ZDHHC8 | 0 | 0 | 0 | 2 | 2 | 2 |
| ZEB2 | 0 | 11 | 0 | 0 | 11 | 11 |
| ZFHX3 | 0 | 2 | 0 | 0 | 2 | 2 |
| ZFHX4 | 0 | 0 | 0 | 1 | 1 | 1 |
| ZFP37 | 0 | 1 | 0 | 1 | 2 | 2 |
| ZFP41 | 0 | 0 | 1 | 0 | 1 | 0 |
| ZFP91 | 1 | 0 | 0 | 0 | 1 | 0 |
| ZFR | 0 | 0 | 2 | 1 | 3 | 1 |
| ZFR2 | 0 | 0 | 0 | 1 | 1 | 1 |
| ZFYVE16 | 0 | 0 | 0 | 1 | 1 | 1 |
| ZFYVE19 | 0 | 0 | 0 | 11 | 11 | 11 |
| ZFYVE20 | 0 | 0 | 0 | 1 | 1 | 1 |
| ZFYVE26 | 0 | 1 | 0 | 1 | 2 | 2 |
| ZFYVE9 | 0 | 11 | 0 | 0 | 11 | 11 |
| ZHX2 | 0 | 0 | 0 | 3 | 3 | 3 |
| ZHX3 | 0 | 0 | 0 | 3 | 3 | 3 |
| ZIK1 | 0 | 0 | 0 | 2 | 2 | 2 |
| ZIM3 | 0 | 11 | 0 | 1 | 12 | 12 |
| ZKSCAN1 | 0 | 1 | 0 | 0 | 1 | 1 |
| ZKSCAN3 | 0 | 0 | 0 | 1 | 1 | 1 |
| ZKSCAN4 | 0 | 0 | 0 | 4 | 4 | 4 |
| ZMIZ1 | 0 | 1 | 0 | 1 | 2 | 2 |
| ZMIZ2 | 0 | 0 | 0 | 1 | 1 | 1 |
| ZMYM1 | 0 | 1 | 0 | 0 | 1 | 1 |
| ZMYM3 | 0 | 2 | 0 | 0 | 2 | 2 |
| ZMYM4 | 3 | 0 | 0 | 0 | 3 | 0 |
| ZMYM5 | 0 | 2 | 0 | 0 | 2 | 2 |
| ZMYND10 | 0 | 2 | 0 | 0 | 2 | 2 |
| ZMYND12 | 0 | 1 | 0 | 2 | 3 | 3 |
| ZMYND15 | 3 | 0 | 0 | 1 | 4 | 1 |
| ZMYND17 | 0 | 0 | 0 | 4 | 4 | 4 |
| ZMYND19 | 0 | 0 | 0 | 1 | 1 | 1 |
| ZNF100 | 0 | 8 | 0 | 1 | 9 | 9 |
| ZNF107 | 0 | 0 | 0 | 7 | 7 | 7 |
| ZNF114 | 0 | 0 | 0 | 1 | 1 | 1 |
| ZNF133 | 0 | 0 | 0 | 1 | 1 | 1 |
| ZNF135 | 0 | 0 | 0 | 1 | 1 | 1 |
| ZNF140 | 0 | 0 | 0 | 4 | 4 | 4 |
| ZNF142 | 0 | 0 | 0 | 2 | 2 | 2 |
| ZNF148 | 0 | 1 | 0 | 0 | 1 | 1 |
| ZNF154 | 0 | 7 | 0 | 0 | 7 | 7 |
| ZNF155 | 0 | 0 | 0 | 1 | 1 | 1 |
| ZNF175 | 0 | 0 | 0 | 3 | 3 | 3 |
| ZNF182 | 0 | 0 | 0 | 1 | 1 | 1 |
| ZNF19 | 0 | 0 | 0 | 1 | 1 | 1 |
| ZNF2 | 0 | 0 | 0 | 2 | 2 | 2 |
| ZNF211 | 0 | 2 | 0 | 0 | 2 | 2 |
| ZNF212 | 0 | 2 | 0 | 0 | 2 | 2 |
| ZNF215 | 1 | 0 | 0 | 0 | 1 | 0 |
| ZNF221 | 0 | 0 | 0 | 2 | 2 | 2 |
| ZNF222 | 0 | 0 | 0 | 1 | 1 | 1 |
| ZNF223 | 0 | 4 | 0 | 0 | 4 | 4 |
| ZNF224 | 0 | 1 | 0 | 1 | 2 | 2 |
| ZNF227 | 0 | 0 | 0 | 2 | 2 | 2 |
| ZNF229 | 0 | 1 | 0 | 3 | 4 | 4 |
| ZNF233 | 0 | 1 | 0 | 0 | 1 | 1 |
| ZNF238 | 0 | 1 | 0 | 0 | 1 | 1 |
| ZNF253 | 0 | 2 | 0 | 0 | 2 | 2 |
| ZNF254 | 0 | 1 | 0 | 0 | 1 | 1 |
| ZNF256 | 1 | 0 | 0 | 0 | 1 | 0 |
| ZNF260 | 0 | 0 | 0 | 1 | 1 | 1 |
| ZNF267 | 0 | 1 | 0 | 0 | 1 | 1 |
| ZNF273 | 0 | 1 | 0 | 2 | 3 | 3 |
| ZNF277 | 0 | 0 | 0 | 1 | 1 | 1 |
| ZNF28 | 0 | 1 | 0 | 7 | 8 | 8 |
| ZNF280A | 0 | 0 | 0 | 1 | 1 | 1 |
| ZNF281 | 0 | 0 | 0 | 1 | 1 | 1 |
| ZNF286A | 0 | 0 | 0 | 1 | 1 | 1 |
| ZNF286B | 0 | 4 | 0 | 0 | 4 | 4 |
| ZNF292 | 0 | 0 | 0 | 2 | 2 | 2 |
| ZNF295 | 0 | 0 | 0 | 1 | 1 | 1 |
| ZNF300 | 0 | 0 | 0 | 1 | 1 | 1 |
| ZNF302 | 0 | 1 | 0 | 0 | 1 | 1 |
| ZNF318 | 0 | 0 | 0 | 1 | 1 | 1 |
| ZNF323 | 0 | 0 | 0 | 2 | 2 | 2 |
| ZNF334 | 0 | 0 | 0 | 1 | 1 | 1 |
| ZNF335 | 0 | 0 | 0 | 3 | 3 | 3 |
| ZNF33B | 0 | 0 | 0 | 1 | 1 | 1 |
| ZNF341 | 0 | 0 | 0 | 1 | 1 | 1 |
| ZNF343 | 3 | 0 | 0 | 0 | 3 | 0 |
| ZNF345 | 0 | 2 | 0 | 0 | 2 | 2 |
| ZNF347 | 0 | 0 | 0 | 17 | 17 | 17 |
| ZNF35 | 0 | 0 | 0 | 1 | 1 | 1 |
| ZNF417 | 0 | 4 | 0 | 0 | 4 | 4 |
| ZNF423 | 0 | 0 | 0 | 1 | 1 | 1 |
| ZNF425 | 0 | 1 | 0 | 1 | 2 | 2 |
| ZNF429 | 0 | 2 | 0 | 8 | 10 | 10 |
| ZNF431 | 1 | 0 | 0 | 0 | 1 | 0 |
| ZNF433 | 0 | 1 | 0 | 1 | 2 | 2 |
| ZNF434 | 0 | 1 | 0 | 0 | 1 | 1 |
| ZNF439 | 1 | 2 | 0 | 0 | 3 | 2 |
| ZNF440 | 0 | 5 | 0 | 1 | 6 | 6 |
| ZNF442 | 0 | 1 | 1 | 0 | 2 | 1 |
| ZNF443 | 0 | 1 | 0 | 0 | 1 | 1 |
| ZNF446 | 0 | 0 | 0 | 1 | 1 | 1 |
| ZNF45 | 0 | 1 | 0 | 1 | 2 | 2 |
| ZNF461 | 0 | 0 | 0 | 1 | 1 | 1 |
| ZNF462 | 0 | 6 | 0 | 0 | 6 | 6 |
| ZNF471 | 0 | 0 | 0 | 1 | 1 | 1 |
| ZNF473 | 0 | 1 | 0 | 0 | 1 | 1 |
| ZNF479 | 0 | 0 | 0 | 1 | 1 | 1 |
| ZNF486 | 0 | 4 | 0 | 1 | 5 | 5 |
| ZNF488 | 0 | 0 | 0 | 2 | 2 | 2 |
| ZNF491 | 0 | 3 | 0 | 0 | 3 | 3 |
| ZNF496 | 0 | 1 | 0 | 2 | 3 | 3 |
| ZNF501 | 0 | 0 | 0 | 1 | 1 | 1 |
| ZNF506 | 0 | 0 | 0 | 5 | 5 | 5 |
| ZNF513 | 0 | 0 | 0 | 3 | 3 | 3 |
| ZNF514 | 0 | 1 | 0 | 0 | 1 | 1 |
| ZNF516 | 0 | 0 | 0 | 1 | 1 | 1 |
| ZNF519 | 0 | 0 | 0 | 1 | 1 | 1 |
| ZNF521 | 0 | 0 | 0 | 1 | 1 | 1 |
| ZNF526 | 0 | 0 | 0 | 1 | 1 | 1 |
| ZNF528 | 0 | 2 | 0 | 1 | 3 | 3 |
| ZNF530 | 0 | 0 | 0 | 1 | 1 | 1 |
| ZNF534 | 0 | 1 | 0 | 1 | 2 | 2 |
| ZNF543 | 1 | 5 | 0 | 0 | 6 | 5 |
| ZNF544 | 1 | 12 | 0 | 0 | 13 | 12 |
| ZNF546 | 0 | 3 | 0 | 2 | 5 | 5 |
| ZNF547 | 0 | 1 | 0 | 0 | 1 | 1 |
| ZNF549 | 0 | 1 | 0 | 0 | 1 | 1 |
| ZNF551 | 0 | 1 | 0 | 0 | 1 | 1 |
| ZNF552 | 0 | 3 | 0 | 0 | 3 | 3 |
| ZNF554 | 0 | 0 | 0 | 1 | 1 | 1 |
| ZNF556 | 1 | 1 | 0 | 0 | 2 | 1 |
| ZNF558 | 0 | 0 | 0 | 1 | 1 | 1 |
| ZNF561 | 0 | 4 | 0 | 1 | 5 | 5 |
| ZNF563 | 0 | 5 | 0 | 0 | 5 | 5 |
| ZNF567 | 0 | 0 | 0 | 1 | 1 | 1 |
| ZNF568 | 0 | 1 | 0 | 0 | 1 | 1 |
| ZNF569 | 0 | 0 | 0 | 1 | 1 | 1 |
| ZNF57 | 0 | 1 | 0 | 0 | 1 | 1 |
| ZNF571 | 0 | 1 | 0 | 0 | 1 | 1 |
| ZNF572 | 0 | 0 | 0 | 1 | 1 | 1 |
| ZNF573 | 0 | 0 | 0 | 1 | 1 | 1 |
| ZNF582 | 0 | 0 | 0 | 1 | 1 | 1 |
| ZNF587 | 0 | 4 | 0 | 2 | 6 | 6 |
| ZNF594 | 0 | 2 | 0 | 4 | 6 | 6 |
| ZNF595 | 0 | 0 | 0 | 1 | 1 | 1 |
| ZNF596 | 0 | 1 | 0 | 0 | 1 | 1 |
| ZNF599 | 0 | 2 | 1 | 9 | 12 | 11 |
| ZNF607 | 0 | 1 | 0 | 0 | 1 | 1 |
| ZNF608 | 0 | 0 | 0 | 1 | 1 | 1 |
| ZNF611 | 0 | 0 | 0 | 1 | 1 | 1 |
| ZNF613 | 0 | 0 | 0 | 1 | 1 | 1 |
| ZNF616 | 0 | 2 | 0 | 0 | 2 | 2 |
| ZNF618 | 0 | 0 | 0 | 2 | 2 | 2 |
| ZNF619 | 0 | 1 | 0 | 0 | 1 | 1 |
| ZNF620 | 0 | 0 | 0 | 1 | 1 | 1 |
| ZNF622 | 0 | 1 | 0 | 0 | 1 | 1 |
| ZNF642 | 2 | 0 | 0 | 0 | 2 | 0 |
| ZNF643 | 0 | 1 | 0 | 0 | 1 | 1 |
| ZNF645 | 0 | 0 | 0 | 1 | 1 | 1 |
| ZNF646 | 0 | 0 | 0 | 1 | 1 | 1 |
| ZNF648 | 0 | 0 | 0 | 1 | 1 | 1 |
| ZNF655 | 0 | 1 | 0 | 0 | 1 | 1 |
| ZNF660 | 0 | 0 | 0 | 1 | 1 | 1 |
| ZNF662 | 0 | 1 | 0 | 0 | 1 | 1 |
| ZNF664 | 0 | 1 | 0 | 0 | 1 | 1 |
| ZNF665 | 0 | 1 | 0 | 0 | 1 | 1 |
| ZNF667 | 0 | 0 | 0 | 1 | 1 | 1 |
| ZNF674 | 0 | 1 | 0 | 2 | 3 | 3 |
| ZNF675 | 0 | 0 | 0 | 2 | 2 | 2 |
| ZNF676 | 1 | 0 | 0 | 0 | 1 | 0 |
| ZNF677 | 1 | 0 | 0 | 1 | 2 | 1 |
| ZNF678 | 0 | 2 | 0 | 1 | 3 | 3 |
| ZNF681 | 0 | 1 | 0 | 2 | 3 | 3 |
| ZNF682 | 0 | 0 | 0 | 1 | 1 | 1 |
| ZNF683 | 0 | 0 | 0 | 1 | 1 | 1 |
| ZNF687 | 0 | 8 | 0 | 0 | 8 | 8 |
| ZNF689 | 0 | 1 | 0 | 1 | 2 | 2 |
| ZNF691 | 0 | 0 | 0 | 4 | 4 | 4 |
| ZNF709 | 0 | 0 | 0 | 1 | 1 | 1 |
| ZNF714 | 0 | 0 | 0 | 1 | 1 | 1 |
| ZNF717 | 0 | 26 | 0 | 91 | 117 | 117 |
| ZNF718 | 0 | 0 | 0 | 2 | 2 | 2 |
| ZNF721 | 0 | 0 | 0 | 3 | 3 | 3 |
| ZNF727 | 4 | 2 | 0 | 0 | 6 | 2 |
| ZNF732 | 0 | 1 | 0 | 0 | 1 | 1 |
| ZNF736 | 0 | 1 | 0 | 0 | 1 | 1 |
| ZNF737 | 0 | 0 | 0 | 1 | 1 | 1 |
| ZNF749 | 0 | 0 | 0 | 5 | 5 | 5 |
| ZNF75A | 0 | 1 | 0 | 0 | 1 | 1 |
| ZNF75D | 0 | 2 | 0 | 1 | 3 | 3 |
| ZNF77 | 0 | 0 | 0 | 1 | 1 | 1 |
| ZNF772 | 0 | 5 | 0 | 0 | 5 | 5 |
| ZNF773 | 1 | 0 | 0 | 0 | 1 | 0 |
| ZNF774 | 0 | 0 | 0 | 1 | 1 | 1 |
| ZNF776 | 1 | 0 | 0 | 1 | 2 | 1 |
| ZNF777 | 0 | 0 | 0 | 1 | 1 | 1 |
| ZNF778 | 0 | 0 | 0 | 1 | 1 | 1 |
| ZNF780B | 8 | 0 | 0 | 0 | 8 | 0 |
| ZNF782 | 0 | 0 | 0 | 1 | 1 | 1 |
| ZNF785 | 0 | 0 | 0 | 2 | 2 | 2 |
| ZNF786 | 0 | 1 | 0 | 0 | 1 | 1 |
| ZNF79 | 0 | 1 | 0 | 0 | 1 | 1 |
| ZNF790 | 0 | 7 | 0 | 1 | 8 | 8 |
| ZNF792 | 0 | 1 | 0 | 5 | 6 | 6 |
| ZNF793 | 0 | 0 | 0 | 1 | 1 | 1 |
| ZNF80 | 0 | 3 | 0 | 5 | 8 | 8 |
| ZNF804A | 0 | 0 | 0 | 4 | 4 | 4 |
| ZNF808 | 0 | 3 | 0 | 1 | 4 | 4 |
| ZNF812 | 0 | 2 | 0 | 5 | 7 | 7 |
| ZNF813 | 0 | 0 | 0 | 8 | 8 | 8 |
| ZNF816 | 0 | 0 | 0 | 1 | 1 | 1 |
| ZNF816-ZNF321P | 0 | 0 | 0 | 2 | 2 | 2 |
| ZNF823 | 0 | 1 | 0 | 0 | 1 | 1 |
| ZNF827 | 0 | 0 | 0 | 1 | 1 | 1 |
| ZNF83 | 0 | 3 | 0 | 0 | 3 | 3 |
| ZNF835 | 0 | 1 | 0 | 4 | 5 | 5 |
| ZNF839 | 0 | 0 | 0 | 1 | 1 | 1 |
| ZNF844 | 0 | 1 | 0 | 9 | 10 | 10 |
| ZNF846 | 0 | 1 | 0 | 1 | 2 | 2 |
| ZNF85 | 0 | 0 | 0 | 1 | 1 | 1 |
| ZNF860 | 0 | 18 | 0 | 0 | 18 | 18 |
| ZNF862 | 0 | 1 | 0 | 0 | 1 | 1 |
| ZNF879 | 3 | 0 | 0 | 0 | 3 | 0 |
| ZNF883 | 0 | 1 | 0 | 1 | 2 | 2 |
| ZNF90 | 0 | 0 | 0 | 2 | 2 | 2 |
| ZNF93 | 0 | 0 | 0 | 1 | 1 | 1 |
| ZNF99 | 0 | 2 | 0 | 1 | 3 | 3 |
| ZNHIT3 | 0 | 0 | 0 | 1 | 1 | 1 |
| ZNHIT6 | 1 | 0 | 0 | 0 | 1 | 0 |
| ZP1 | 0 | 0 | 0 | 3 | 3 | 3 |
| ZP2 | 0 | 1 | 0 | 0 | 1 | 1 |
| ZP3 | 0 | 0 | 0 | 10 | 10 | 10 |
| ZP4 | 0 | 1 | 0 | 0 | 1 | 1 |
| ZRANB3 | 5 | 0 | 0 | 0 | 5 | 0 |
| ZSCAN1 | 0 | 0 | 0 | 1 | 1 | 1 |
| ZSCAN16 | 0 | 1 | 0 | 0 | 1 | 1 |
| ZSCAN22 | 0 | 1 | 0 | 1 | 2 | 2 |
| ZSCAN5B | 0 | 0 | 0 | 1 | 1 | 1 |
| ZSWIM1 | 0 | 5 | 0 | 0 | 5 | 5 |
| ZSWIM2 | 0 | 1 | 0 | 0 | 1 | 1 |
| ZSWIM3 | 0 | 0 | 0 | 1 | 1 | 1 |
| ZSWIM4 | 2 | 0 | 0 | 0 | 2 | 0 |
| ZSWIM6 | 0 | 0 | 0 | 1 | 1 | 1 |
| ZSWIM7 | 0 | 0 | 0 | 1 | 1 | 1 |
| ZUFSP | 0 | 1 | 0 | 0 | 1 | 1 |
| ZWINT | 0 | 0 | 0 | 2 | 2 | 2 |
| ZXDA | 0 | 1 | 0 | 0 | 1 | 1 |
| ZXDB | 0 | 0 | 0 | 1 | 1 | 1 |
| ZXDC | 1 | 0 | 0 | 0 | 1 | 0 |

**Supplementary Table 2b:** Details of protein truncating mutations identified in TCGA HGSOC cases

| **Frequency** | **Gene** | **exon** | **Type** | **cDNA** |
| --- | --- | --- | --- | --- |
| 1 | A1BG | Exon5 | frameshift deletion | c.760delA |
| 1 | A1BG | Exon5 | frameshift deletion | c.751delC |
| 1 | A2ML1 | Exon4 | splicing | c.462+1G>C |
| 1 | A2ML1 | Exon12 | frameshift deletion | c.1444_1445delAG |
| 1 | A2M | Exon2 | frameshift deletion | c.202delA |
| 1 | A4GALT | Exon3 | frameshift deletion | c.845delA |
| 1 | A4GALT | Exon3 | frameshift insertion | c.839_840insT |
| 2 | A4GNT | Exon3 | stopgain SNV | c.796C>T |
| 1 | AACS | Exon6 | stopgain SNV | c.670C>T |
| 1 | AACS | Exon17 | frameshift deletion | c.1723delT |
| 1 | AADACL2 | Exon4 | stopgain SNV | c.559G>T |
| 16 | AADACL3 | Exon3 | frameshift deletion | c.374delG |
| 1 | AADAC | Exon5 | frameshift deletion | c.720delT |
| 5 | AADAC | Exon5 | stoploss SNV | c.1198T>C |
| 1 | AAGAB | Exon10 | stopgain SNV | c.887G>A |
| 1 | AAK1 | Exon16 | stopgain SNV | c.2176G>T |
| 1 | AAMP | Exon10 | frameshift deletion | c.1090delC |
| 3 | AARS2 | Exon2 | frameshift insertion | c.267dupC |
| 1 | AARSD1 | Exon7 | stopgain SNV | c.646C>T |
| 3 | AASDH | Exon8 | frameshift deletion | c.1319delT |
| 1 | AASDH | Exon3 | frameshift deletion | c._del |
| 1 | AATF | Exon4 | splicing | c.832+2dupT |
| 10 | ABCA10 | Exon32 | frameshift insertion | c.3689_3696dupTTCCAGGT |
| 2 | ABCA10 | Exon31 | frameshift deletion | c.3685_3686delGT |
| 1 | ABCA10 | Exon20 | frameshift deletion | c.2170delA |
| 2 | ABCA10 | Exon17 | stopgain SNV | c.1843C>T |
| 3 | ABCA10 | Exon16 | stopgain SNV | c.1729C>T |
| 13 | ABCA10 | Exon14 | frameshift deletion | c.1357_1358delAT |
| 5 | ABCA10 | Exon9 | stopgain SNV | c.783T>A |
| 1 | ABCA13 | Exon6 | stoploss SNV | c.631delT |
| 4 | ABCA13 | Exon10 | stoploss SNV | c.1288T>A |
| 1 | ABCA13 | Exon11 | stopgain SNV | c.1336C>T |
| 9 | ABCA13 | Exon15 | stoploss SNV | c.3830A>G |
| 9 | ABCA13 | Exon15 | stoploss SNV | c.5503T>A |
| 1 | ABCA13 | Exon15 | stoploss SNV | c.5503T>G |
| 8 | ABCA13 | Exon15 | stopgain SNV | c.6298C>T |
| 8 | ABCA13 | Exon15 | stoploss SNV | c.6472T>C |
| 1 | ABCA13 | Exon16 | stoploss SNV | c.7546T>C |
| 1 | ABCA13 | Exon16 | frameshift deletion | c.7880_7881delAG |
| 1 | ABCA13 | Exon16 | frameshift insertion | c.7884_7885insT |
| 3 | ABCA13 | Exon21 | stoploss SNV | c.9058T>A |
| 1 | ABCA13 | Exon22 | stopgain SNV | c.9225C>A |
| 1 | ABCA13 | Exon30 | splicing | c.10526-2A>G |
| 1 | ABCA13 | Exon39 | splicing | c.12296+1G>T |
| 9 | ABCA13 | Exon48 | stopgain SNV | c.13227C>A |
| 1 | ABCA13 | Exon52 | stopgain SNV | c.14032A>T |
| 1 | ABCA4 | Exon23 | frameshift insertion | c.3395dupT |
| 1 | ABCA5 | Exon6 | stopgain SNV | c.742delA |
| 1 | ABCA6 | Exon13 | frameshift deletion | c.1712_1715delTCAC |
| 1 | ABCA6 | Exon4 | frameshift deletion | c.321_322delAC |
| 1 | ABCA8 | Exon27 | frameshift deletion | c.3576delT |
| 2 | ABCA8 | Exon17 | stopgain SNV | c.2219_2220dupTG |
| 1 | ABCA9 | Exon38 | splicing | c.4641-1G>A |
| 1 | ABCA9 | Exon9 | frameshift deletion | c.1275delC |
| 4 | ABCA9 | Exon8 | splicing | c.942+2T>C |
| 1 | ABCA9 | Exon6 | stopgain SNV | c.784C>T |
| 1 | ABCA9 | Exon6 | stopgain SNV | c.739C>T |
| 1 | ABCB10 | Exon10 | frameshift deletion | c.1821_1822delTG |
| 1 | ABCB10 | Exon9 | stopgain SNV | c.1723C>T |
| 1 | ABCB10 | Exon8 | frameshift insertion | c.1572_1573insG |
| 3 | ABCB11 | Exon25 | frameshift deletion | c.3244_3245delGA |
| 12 | ABCB5 | Exon3 | splicing | c.108+1G>C |
| 1 | ABCB5 | Exon6 | stopgain SNV | c.400C>T |
| 1 | ABCB5 | Exon28 | stopgain SNV | c.3583C>T |
| 1 | ABCB6 | Exon7 | frameshift deletion | c.1369_1370delCT |
| 13 | ABCB7 | Exon3 | splicing | c.249+1G>A |
| 1 | ABCB9 | Exon6 | frameshift deletion | c.1228delA |
| 5 | ABCC10 | Exon3 | frameshift deletion | c.1257delA |
| 7 | ABCC11 | Exon29 | splicing | c.3778-1G>A |
| 3 | ABCC11 | Exon21 | stopgain SNV | c.2813C>G |
| 12 | ABCC12 | Exon22 | stopgain SNV | c.3071G>A |
| 1 | ABCC12 | Exon20 | frameshift deletion | c.2704_2708delACGAC |
| 2 | ABCC12 | Exon19 | stopgain SNV | c.2491C>T |
| 4 | ABCC12 | Exon18 | frameshift deletion | c.2425delG |
| 2 | ABCC12 | Exon13 | frameshift deletion | c.1888_1889delCT |
| 1 | ABCC12 | Exon4 | stopgain SNV | c.490G>T |
| 3 | ABCC12 | Exon2 | frameshift deletion | c.197_198delAA |
| 4 | ABCC1 | Exon20 | stopgain SNV | c.2592T>G |
| 1 | ABCC1 | Exon20 | frameshift insertion | c.2610dupG |
| 1 | ABCC1 | Exon23 | splicing | c.3223-2_3223-2delA |
| 2 | ABCC2 | Exon28 | stopgain SNV | c.3928C>T |
| 1 | ABCC4 | Exon3 | frameshift insertion | c.305_306insC |
| 5 | ABCC5 | Exon20 | frameshift deletion | c.2855_2856delTC |
| 1 | ABCC5 | Exon16 | stopgain SNV | c.2318T>G |
| 1 | ABCC6 | Exon8 | stopgain SNV | c.913C>T |
| 2 | ABCC9 | Exon19 | splicing | c.2238-1G>A |
| 1 | ABCD3 | Exon3 | frameshift deletion | c.163delG |
| 3 | ABCE1 | Exon9 | frameshift deletion | c.750delG |
| 3 | ABCF1 | Exon15 | stopgain SNV | c.1418T>A |
| 1 | ABCF3 | Exon17 | frameshift insertion | c.1585_1586insA |
| 1 | ABCF3 | Exon17 | frameshift insertion | c.1587dupT |
| 1 | ABCF3 | Exon19 | splicing | c.1883+2T>G |
| 1 | ABCG2 | Exon7 | stopgain SNV | c.736C>T |
| 1 | ABCG2 | Exon4 | stopgain SNV | c.337C>T |
| 1 | ABCG4 | Exon3 | frameshift deletion | c.239delG |
| 2 | ABCG8 | Exon7 | stopgain SNV | c.1083G>A |
| 3 | ABHD12B | Exon3 | splicing | c.255+1G>T |
| 1 | ABHD12B | Exon4 | stopgain SNV | c.297C>G |
| 1 | ABHD12B | Exon8 | frameshift deletion | c.589delG |
| 1 | ABHD12 | Exon12 | stopgain SNV | c.1063C>T |
| 3 | ABHD14B | Exon3 | frameshift insertion | c.314dupC |
| 1 | ABHD14B | Exon2 | stopgain SNV | c.49C>T |
| 1 | ABHD2 | Exon8 | frameshift insertion | c.323dupC |
| 1 | ABHD3 | Exon7 | splicing | c.669-1G>A |
| 1 | ABHD3 | Exon4 | frameshift deletion | c.544delG |
| 1 | ABLIM1 | Exon5 | splicing | c.493+-1_493+1dupCAG |
| 2 | ABO | Exon7 | frameshift deletion | c.1060delC |
| 1 | ABO | Exon5 | frameshift deletion | c.223_224delAA |
| 1 | ABP1 | Exon4 | frameshift deletion | c.1878_1879delGT |
| 1 | ABTB1 | Exon10 | stopgain SNV | c.916C>T |
| 1 | ABTB2 | Exon9 | stopgain SNV | c.1860T>A |
| 1 | ABTB2 | Exon2 | frameshift deletion | c.895delC |
| 1 | ACACA | Exon22 | stopgain SNV | c.2107G>T |
| 1 | ACACA | Exon16 | frameshift insertion | c.1364_1365insG |
| 3 | ACACB | Exon18 | frameshift deletion | c.2812delG |
| 1 | ACACB | Exon24 | frameshift insertion | c.3581dupC |
| 1 | ACACB | Exon45 | frameshift deletion | c.6287delT |
| 1 | ACACB | Exon51 | stopgain SNV | c.7125G>A |
| 1 | ACAD10 | Exon4 | frameshift deletion | c.450delT |
| 1 | ACAD10 | Exon11 | stopgain SNV | c.1381C>T |
| 1 | ACAD11 | Exon16 | stopgain SNV | c.1819C>T |
| 7 | ACAD11 | Exon12 | splicing | c.1414+1G>T |
| 3 | ACAD11 | Exon10 | splicing | c.1071-1G>A |
| 1 | ACAD11 | Exon8 | stopgain SNV | c.1069C>T |
| 1 | ACAD11 | Exon7 | frameshift deletion | c.937_943delTATTTTA |
| 7 | ACAD11 | Exon3 | stopgain SNV | c.321C>G |
| 4 | ACAD11 | Exon2 | frameshift deletion | c.247delC |
| 1 | ACAD9 | Exon14 | frameshift deletion | c.1430delG |
| 1 | ACADM | Exon4 | frameshift insertion | c.244dupT |
| 1 | ACADVL | Exon11 | splicing | c.1182+1G>A |
| 1 | ACAN | Exon6 | frameshift deletion | c.907_908delGC |
| 2 | ACAN | Exon12 | frameshift deletion | c.5912delC |
| 1 | ACBD4 | Exon5 | stopgain SNV | c.135C>G |
| 1 | ACCN1 | Exon6 | stopgain SNV | c.1057A>T |
| 3 | ACCN2 | Exon4 | stopgain SNV | c.676C>T |
| 5 | ACCN2 | Exon5 | stopgain SNV | c.826C>T |
| 1 | ACCN3 | Exon1 | stopgain SNV | c.235C>T |
| 1 | ACCN3 | Exon7 | stopgain SNV | c.1260delT |
| 1 | ACE | Exon22 | frameshift deletion | c.3347delA |
| 10 | ACIN1 | Exon6 | frameshift deletion | c.1339delG |
| 1 | ACIN1 | Exon6 | frameshift deletion | c.1232delA |
| 1 | ACLY | Exon21 | frameshift deletion | c.2414delA |
| 2 | ACLY | Exon3 | splicing | c.159+2T>G |
| 1 | ACOT12 | Exon10 | stopgain SNV | c.1003G>T |
| 1 | ACOT13 | Exon3 | splicing | c.197+2dupT |
| 1 | ACOT1 | Exon2 | frameshift deletion | c.605delC |
| 1 | ACOT2 | Exon1 | frameshift insertion | c.65_66insA |
| 1 | ACOT4 | Exon1 | frameshift insertion | c.364dupC |
| 7 | ACOT4 | Exon2 | frameshift insertion | c.563_564insTCAA |
| 1 | ACOT6 | Exon2 | frameshift deletion | c.215_216delAA |
| 1 | ACOT6 | Exon2 | frameshift deletion | c.234delT |
| 1 | ACOT6 | Exon2 | frameshift deletion | c.340delA |
| 3 | ACOT8 | Exon2 | frameshift insertion | c.207_208dupGT |
| 1 | ACOT8 | Exon1 | frameshift insertion | c.-1_2dupAGAT |
| 1 | ACOX2 | Exon4 | frameshift deletion | c.461_464delCAGA |
| 1 | ACOX3 | Exon3 | stopgain SNV | c.241C>T |
| 3 | ACOXL | Exon16 | stopgain SNV | c.1407T>A |
| 1 | ACP6 | Exon3 | frameshift deletion | c.372delG |
| 4 | ACPL2 | Exon8 | frameshift deletion | c.580_581delGA |
| 2 | ACPP | Exon6 | frameshift deletion | c.620_632del  GTAAAGTCTACGA |
| 1 | ACSBG1 | Exon9 | frameshift deletion | c.1084delG |
| 3 | ACSBG2 | Exon3 | splicing | c.297+1G>A |
| 1 | ACSBG2 | Exon8 | frameshift deletion | c.775_779delAAACT |
| 1 | ACSBG2 | Exon10 | frameshift deletion | c.1305delT |
| 1 | ACSF3 | Exon4 | stopgain SNV | c.689G>A |
| 1 | ACSL5 | Exon17 | splicing | c.1477-13_1477-4del  GTCTGCTGTG |
| 1 | ACSL6 | Exon6 | frameshift deletion | c.526delC |
| 2 | ACSM2A | Exon3 | stopgain SNV | c.72C>G |
| 6 | ACSM2A | Exon4 | stopgain SNV | c.343C>T |
| 8 | ACSM2A | Exon4 | stopgain SNV | c.360G>A |
| 1 | ACSM3 | Exon6 | stopgain SNV | c.875G>A |
| 6 | ACSM3 | Exon10 | stopgain SNV | c.1323C>G |
| 1 | ACSM4 | Exon1 | frameshift deletion | c.56_72delCTGGCCGGCGCTTACAC |
| 1 | ACSM4 | Exon2 | stopgain SNV | c.208G>T |
| 1 | ACSM4 | Exon2 | stopgain SNV | c.295C>T |
| 1 | ACSM4 | Exon3 | splicing | c.413-1G>A |
| 1 | ACSM4 | Exon3 | stopgain SNV | c.472C>T |
| 1 | ACSM5 | Exon2 | frameshift deletion | c.54_57delATTC |
| 11 | ACSS2 | Exon8 | frameshift insertion | c.579_580dupGT |
| 1 | ACTA2 | Exon2 | frameshift insertion | c.105dupC |
| 1 | ACTL6A | Exon5 | frameshift insertion | c.471_472insAT |
| 1 | ACTL7B | Exon1 | frameshift deletion | c.736_739delGACG |
| 1 | ACTL9 | Exon1 | frameshift insertion | c.902dupT |
| 1 | ACTL9 | Exon1 | frameshift deletion | c.110delT |
| 1 | ACTN1 | Exon18 | stopgain SNV | c.2155C>T |
| 5 | ACTN1 | Exon18 | stopgain SNV | c.2152G>T |
| 1 | ACTN3 | Exon21 | stopgain SNV | c.2566G>T |
| 1 | ACTR1B | Exon9 | stopgain SNV | c.937C>T |
| 1 | ACTR3C | Exon6 | stopgain SNV | c.526C>T |
| 3 | ACTR3C | Exon5 | stopgain SNV | c.361C>T |
| 1 | ACTR8 | Exon6 | frameshift deletion | c.725delA |
| 1 | ACTR8 | Exon4 | stopgain SNV | c.430C>T |
| 4 | ACTRT1 | Exon1 | frameshift insertion | c.547dupA |
| 1 | ACVRL1 | Exon7 | frameshift deletion | c.913delT |
| 1 | ACVRL1 | Exon7 | frameshift deletion | c.916delG |
| 1 | ADAD1 | Exon10 | splicing | c.1249+2T>C |
| 1 | ADAM18 | Exon6 | stopgain SNV | c.501C>G |
| 1 | ADAM18 | Exon8 | frameshift deletion | c.600_604delGGGAT |
| 1 | ADAM19 | Exon21 | frameshift deletion | c.2493delC |
| 1 | ADAM19 | Exon21 | frameshift insertion | c.2400_2401insAC |
| 1 | ADAM19 | Exon2 | stopgain SNV | c.118A>T |
| 1 | ADAM20 | Exon2 | frameshift insertion | c.688dupA |
| 1 | ADAM21 | Exon2 | frameshift deletion | c.151delA |
| 1 | ADAM22 | Exon19 | frameshift insertion | c.1593dupT |
| 19 | ADAM28 | Exon6 | frameshift deletion | c.536delA |
| 1 | ADAM29 | Exon5 | stopgain SNV | c.2020A>T |
| 3 | ADAM29 | Exon5 | stoploss SNV | c.2461T>C |
| 7 | ADAM2 | Exon9 | splicing | c.571-2A>T |
| 1 | ADAM30 | Exon1 | frameshift insertion | c.1865dupA |
| 1 | ADAM30 | Exon1 | frameshift insertion | c.844dupA |
| 1 | ADAM33 | Exon3 | frameshift deletion | c.237delT |
| 1 | ADAM7 | Exon10 | frameshift insertion | c.943dupT |
| 1 | ADAM7 | Exon15 | frameshift deletion | c.1562_1565delAGAG |
| 8 | ADAM8 | Exon23 | frameshift insertion | c.2456dupA |
| 1 | ADAM9 | Exon4 | stopgain SNV | c.291C>G |
| 1 | ADAMDEC1 | Exon8 | frameshift deletion | c.620delC |
| 1 | ADAMTS12 | Exon10 | splicing | c.1335-1G>C |
| 1 | ADAMTS15 | Exon1 | stopgain SNV | c.120C>A |
| 1 | ADAMTS15 | Exon5 | stopgain SNV | c.1557G>A |
| 1 | ADAMTS16 | Exon3 | stopgain SNV | c.400A>T |
| 1 | ADAMTS18 | Exon3 | frameshift deletion | c.446_447delTC |
| 1 | ADAMTS20 | Exon28 | splicing | c.4107+2T>A |
| 1 | ADAMTS4 | Exon5 | stopgain SNV | c.1348C>T |
| 2 | ADAMTS7 | Exon7 | stopgain SNV | c.1072G>T |
| 1 | ADAMTS9 | Exon11 | splicing | c.1464-15_1464-6del  TGTGTGTTTC |
| 8 | ADAMTSL1 | Exon11 | frameshift insertion | c.1184dupG |
| 1 | ADAMTSL3 | Exon7 | frameshift deletion | c.679delT |
| 1 | ADAMTSL3 | Exon21 | frameshift deletion | c.3355_3356delCC |
| 1 | ADAMTSL3 | Exon21 | frameshift deletion | c.3371delT |
| 1 | ADAMTSL3 | Exon30 | stoploss SNV | c.5074T>C |
| 2 | ADAMTSL4 | Exon6 | frameshift deletion | c.767_786del  AGGCCTCTGGCACAGAGCCC |
| 1 | ADAMTSL4 | Exon12 | frameshift insertion | c.1914_1915insT |
| 1 | ADAMTSL4 | Exon15 | frameshift deletion | c.2554delT |
| 1 | ADA | Exon10 | frameshift deletion | c.956_960delAAGAG |
| 1 | ADA | Exon4 | frameshift deletion | c.318delG |
| 1 | ADA | Exon4 | frameshift deletion | c.317delT |
| 1 | ADAP1 | Exon10 | stopgain SNV | c.918C>A |
| 1 | ADAP2 | Exon2 | splicing | c.225+1G>A |
| 1 | ADARB1 | Exon6 | frameshift deletion | c.1079_1080delGG |
| 1 | ADAT2 | Exon4 | frameshift deletion | c.409_410delGT |
| 1 | ADCK2 | Exon1 | splicing | c.933+1G>C |
| 1 | ADCY10 | Exon17 | frameshift deletion | c.2206delT |
| 1 | ADCY2 | Exon17 | frameshift deletion | c.2202delA |
| 1 | ADCY5 | Exon8 | frameshift insertion | c.1956_1957insGC |
| 1 | ADCY7 | Exon20 | frameshift insertion | c.2533_2534insAG |
| 1 | ADCY7 | Exon25 | frameshift insertion | c.3113_3114insT |
| 1 | ADD3 | Exon14 | stopgain SNV | c.1987delC |
| 1 | ADD3 | Exon14 | frameshift insertion | c.2006dupA |
| 1 | ADH1A | Exon5 | splicing | c.347+1G>C |
| 1 | ADH5 | Exon5 | frameshift deletion | c.503_504delAA |
| 4 | ADI1 | Exon4 | stopgain SNV | c.508C>T |
| 1 | ADNP2 | Exon4 | frameshift insertion | c.2904dupG |
| 1 | ADPRHL1 | Exon6 | frameshift insertion | c.863_864insC |
| 1 | ADPRH | Exon5 | stopgain SNV | c.798G>A |
| 1 | ADRB3 | Exon3 | splicing | c.1206-2A>C |
| 1 | ADRBK1 | Exon13 | frameshift deletion | c.1074delT |
| 1 | ADRBK2 | Exon6 | stopgain SNV | c.454G>T |
| 1 | ADSSL1 | Exon9 | frameshift deletion | c.914delT |
| 1 | AFAP1L1 | Exon9 | stopgain SNV | c.934C>T |
| 1 | AFAP1L2 | Exon15 | frameshift insertion | c.1954dupG |
| 1 | AFAP1L2 | Exon15 | frameshift deletion | c.1886delC |
| 1 | AFMID | Exon6 | splicing | c.395-2A>G |
| 1 | AFM | Exon1 | frameshift insertion | c.47dupT |
| 2 | AGAP2 | Exon2 | stopgain SNV | c.185G>A |
| 3 | AGAP5 | Exon8 | stopgain SNV | c.1957C>T |
| 7 | AGAP6 | Exon1 | frameshift insertion | c.55dupC |
| 10 | AGAP6 | Exon1 | frameshift deletion | c.208delC |
| 3 | AGAP6 | Exon8 | stopgain SNV | c.1861G>T |
| 1 | AGBL1 | Exon22 | stopgain SNV | c.3082C>T |
| 1 | AGBL1 | Exon23 | splicing | c.3185+1G>T |
| 4 | AGBL1 | Exon23 | splicing | c.3185+2dupT |
| 1 | AGBL2 | Exon18 | frameshift insertion | c.2474dupA |
| 1 | AGER | Exon2 | stopgain SNV | c.153G>A |
| 1 | AGL | Exon28 | splicing | c.3836+1G>A |
| 1 | AGMAT | Exon8 | splicing | c.986-2A>T |
| 2 | AGMO | Exon1 | frameshift insertion | c.60_61dupTT |
| 1 | AGPAT3 | Exon6 | frameshift deletion | c.649delT |
| 6 | AGPAT4 | Exon4 | stopgain SNV | c.439A>T |
| 1 | AGPAT6 | Exon4 | frameshift insertion | c.418_419insAT |
| 1 | AGPAT6 | Exon12 | frameshift deletion | c.1244delG |
| 1 | AGPAT6 | Exon12 | frameshift deletion | c.1248delT |
| 4 | AGPAT6 | Exon13 | stoploss SNV | c.1370G>C |
| 1 | AGPAT9 | Exon3 | stopgain SNV | c.259C>T |
| 4 | AGPHD1 | Exon3 | stopgain SNV | c.435T>A |
| 1 | AGR2 | Exon2 | stopgain SNV | c.51C>G |
| 1 | AGR3 | Exon6 | splicing | c.303+2T>C |
| 1 | AGRP | Exon3 | frameshift insertion | c.131dupG |
| 1 | AGTPBP1 | Exon14 | frameshift deletion | c.1862delA |
| 1 | AGTPBP1 | Exon14 | frameshift deletion | c.1853delC |
| 1 | AGTR2 | Exon3 | frameshift deletion | c.402delT |
| 1 | AGXT2 | Exon5 | splicing | c.363-1G>T |
| 1 | AGXT2 | Exon3 | splicing | c.177+2T>C |
| 1 | AHCTF1 | Exon27 | splicing | c.3374+1G>T |
| 5 | AHCTF1 | Exon15 | splicing | c.1742-1G>A |
| 1 | AHDC1 | Exon6 | frameshift insertion | c.1785_1786insG |
| 1 | AHI1 | Exon8 | stopgain SNV | c.1267C>T |
| 4 | AHI1 | Exon8 | stopgain SNV | c.1244C>G |
| 1 | AHNAK2 | Exon7 | frameshift insertion | c.16956_16957insA |
| 2 | AHNAK2 | Exon7 | frameshift insertion | c.16843_16844insT |
| 1 | AHNAK2 | Exon7 | stopgain SNV | c.9316C>T |
| 1 | AHNAK2 | Exon7 | frameshift deletion | c.7840delA |
| 3 | AHNAK2 | Exon7 | frameshift insertion | c.3335_3336insA |
| 3 | AHNAK2 | Exon7 | frameshift deletion | c.3330delG |
| 1 | AHR | Exon10 | frameshift deletion | c.1165delG |
| 1 | AHSA1 | Exon9 | stoploss SNV | c.1015T>A |
| 1 | AHSA2 | Exon6 | stopgain SNV | c.295G>T |
| 1 | AHSA2 | Exon6 | frameshift insertion | c.320_323dupCTAT |
| 1 | AHSG | Exon1 | stopgain SNV | c.4A>T |
| 1 | AHSP | gene | frameshift deletion | wholegene |
| 1 | AIFM3 | Exon9 | frameshift deletion | c.807_807del |
| 1 | AIM1 | Exon2 | frameshift insertion | c.2008dupA |
| 1 | AIM1 | Exon9 | frameshift deletion | c.3759_3760delTG |
| 2 | AIM2 | Exon6 | stoploss SNV | c.1030T>G |
| 11 | AIM2 | Exon6 | frameshift deletion | c.1027delA |
| 1 | AIM2 | Exon6 | frameshift insertion | c.1027dupA |
| 1 | AIMP1 | Exon7 | frameshift deletion | c.828delT |
| 1 | AIPL1 | Exon3 | splicing | c.97-2A>G |
| 1 | AIP | Exon2 | frameshift deletion | c.209delT |
| 1 | AIP | Exon2 | frameshift deletion | c.213delT |
| 3 | AK7 | Exon3 | stopgain SNV | c.336T>A |
| 1 | AK8 | Exon8 | frameshift deletion | c.605delA |
| 1 | AKAP10 | Exon4 | frameshift deletion | c.775delC |
| 2 | AKAP13 | Exon34 | stopgain SNV | c.7618C>T |
| 4 | AKAP3 | Exon4 | frameshift insertion | c.2102dupG |
| 4 | AKAP3 | Exon4 | frameshift deletion | c.2098delT |
| 1 | AKAP7 | Exon4 | splicing | c.428+1G>A |
| 3 | AKAP8 | Exon11 | splicing | c.1302+2T>G |
| 1 | AKD1 | Exon23 | stopgain SNV | c.2570T>G |
| 1 | AKD1 | Exon10 | stopgain SNV | c.847C>T |
| 1 | AKIRIN2 | Exon2 | frameshift deletion | c.260_261delAA |
| 1 | AKNAD1 | Exon14 | splicing | c.2167+1G>A |
| 1 | AKNAD1 | Exon9 | splicing | c.1664+1G>A |
| 1 | AKNA | Exon18 | frameshift insertion | c.3630_3631insAA |
| 1 | AKNA | Exon9 | splicing | c.1916+1G>A |
| 1 | AKR1B10 | Exon1 | frameshift deletion | c.37delA |
| 1 | AKR1C3 | Exon9 | stopgain SNV | c.969T>G |
| 1 | AKR7A2 | Exon7 | stopgain SNV | c.1033C>T |
| 7 | AKT1 | Exon4 | stopgain SNV | c.235C>T |
| 1 | AKT3 | Exon9 | splicing | c.819+2_819+2delT |
| 8 | ALAS1 | Exon6 | frameshift deletion | c.840delT |
| 1 | ALDH18A1 | Exon14 | stopgain SNV | c.1648A>T |
| 1 | ALDH18A1 | Exon4 | frameshift insertion | c.438_439dupCC |
| 1 | ALDH1B1 | Exon2 | frameshift insertion | c.479_480insGA |
| 1 | ALDH1B1 | Exon2 | frameshift insertion | c.482dupT |
| 2 | ALDH1B1 | Exon2 | frameshift deletion | c.578delG |
| 1 | ALDH1B1 | Exon2 | stopgain SNV | c.1132C>T |
| 4 | ALDH1B1 | Exon2 | frameshift deletion | c.1161delC |
| 1 | ALDH1L1 | Exon15 | splicing | c.1694+1G>T |
| 1 | ALDH1L2 | Exon3 | stopgain SNV | c.381T>A |
| 1 | ALDH1L2 | Exon2 | stopgain SNV | c.139C>T |
| 1 | ALDH2 | Exon7 | frameshift deletion | c.670delC |
| 1 | ALDH2 | Exon7 | frameshift deletion | c.671delA |
| 1 | ALDH2 | Exon7 | frameshift insertion | c.723dupG |
| 1 | ALDH3A2 | Exon3 | stopgain SNV | c.462T>G |
| 1 | ALDH4A1 | Exon7 | frameshift deletion | c.614delC |
| 1 | ALDH4A1 | Exon7 | frameshift deletion | c.609_610delCG |
| 1 | ALDH5A1 | Exon3 | frameshift insertion | c.454_455insTA |
| 1 | ALDH5A1 | Exon6 | splicing | c.766-1G>T |
| 1 | ALDH6A1 | Exon8 | frameshift insertion | c.1003dupG |
| 1 | ALDH9A1 | Exon5 | stopgain SNV | c.732T>A |
| 2 | ALDOB | Exon3 | stopgain SNV | c.178C>T |
| 1 | ALDOC | Exon3 | frameshift deletion | c.236delT |
| 1 | ALG10B | Exon2 | stopgain SNV | c.355C>T |
| 1 | ALG10 | Exon2 | frameshift deletion | c._del |
| 1 | ALG1L2 | Exon3 | stopgain SNV | c.248G>A |
| 1 | ALG1 | Exon11 | stopgain SNV | c.1095T>A |
| 1 | ALG8 | Exon9 | stopgain SNV | c.902T>A |
| 1 | ALG9 | Exon8 | frameshift deletion | c.876delC |
| 1 | ALKBH3 | Exon2 | frameshift insertion | c.13dupA |
| 1 | ALKBH3 | Exon4 | stopgain SNV | c.208C>T |
| 1 | ALKBH3 | Exon6 | frameshift deletion | c.368_369delAG |
| 1 | ALK | Exon29 | stopgain SNV | c.4849C>T |
| 1 | ALLC | Exon5 | stopgain SNV | c.265C>T |
| 1 | ALOX12 | Exon11 | stopgain SNV | c.1450C>T |
| 1 | ALOX15B | Exon2 | frameshift deletion | c.279delG |
| 2 | ALOX15B | Exon10 | stopgain SNV | c.1387C>T |
| 1 | ALOXE3 | Exon7 | stopgain SNV | c.1096C>T |
| 1 | ALPK1 | Exon3 | stopgain SNV | c.46C>T |
| 2 | ALPK2 | Exon6 | frameshift deletion | c.5373delG |
| 2 | ALPK2 | Exon5 | frameshift deletion | c.3017delA |
| 2 | ALPK2 | Exon5 | stopgain SNV | c.2737A>T |
| 4 | ALPK2 | Exon5 | frameshift insertion | c.2045dupC |
| 1 | ALPK2 | Exon4 | frameshift insertion | c.506_509dupACCA |
| 1 | ALPP | Exon4 | frameshift insertion | c.378_379insGT |
| 1 | ALPP | Exon7 | stopgain SNV | c.804T>G |
| 1 | ALS2CL | Exon26 | frameshift deletion | c._del |
| 1 | ALS2CR11 | Exon10 | stopgain SNV | c.997G>T |
| 1 | ALS2CR11 | Exon1 | frameshift insertion | c.51dupC |
| 1 | ALS2CR12 | Exon15 | stopgain SNV | c.1265C>G |
| 4 | ALS2CR8 | Exon16 | frameshift insertion | c.2146dupA |
| 2 | ALS2 | Exon16 | splicing | c.2841+1G>C |
| 1 | AMBN | Exon7 | splicing | c.532-1G>A |
| 1 | AMHR2 | Exon3 | frameshift deletion | c.403_404delCC |
| 1 | AMHR2 | Exon3 | frameshift deletion | c.407delG |
| 1 | AMHR2 | Exon3 | frameshift deletion | c.414_418delGGCTG |
| 1 | AMICA1 | Exon10 | splicing | c.1006-1G>C |
| 1 | AMOTL1 | Exon10 | splicing | c.2261+2T>C |
| 1 | AMPD2 | Exon1 | splicing | c.10+2_10+8delTGAGTGT |
| 1 | AMY2B | Exon7 | splicing | c.745-2A>T |
| 1 | AMZ1 | Exon7 | stopgain SNV | c.1441C>T |
| 2 | AMZ2 | Exon7 | frameshift deletion | c.937_941delAGGTG |
| 1 | ANAPC4 | Exon27 | stopgain SNV | c.1924C>T |
| 3 | ANGEL2 | Exon2 | frameshift deletion | c.197_206delACCCATACTT |
| 1 | ANGPT2 | Exon3 | stopgain SNV | c.497C>A |
| 1 | ANGPT4 | Exon9 | frameshift deletion | c.1366delG |
| 1 | ANGPTL3 | Exon1 | frameshift deletion | c.361_365delAACTC |
| 1 | ANGPTL3 | Exon6 | splicing | c.932-2A>G |
| 1 | ANGPTL4 | Exon6 | frameshift insertion | c.1101dupC |
| 1 | ANGPTL5 | Exon9 | stopgain SNV | c.1158T>G |
| 1 | ANGPTL6 | Exon4 | stopgain SNV | c.851C>G |
| 7 | ANK1 | Exon33 | frameshift deletion | c.4106_4107delAG |
| 1 | ANK1 | Exon15 | frameshift insertion | c.1722_1723dupCG |
| 1 | ANK3 | Exon17 | splicing | c.1672-1_1672-1delG |
| 1 | ANK3 | Exon17 | splicing | c.1672-1G>A |
| 1 | ANK3 | Exon15 | stopgain SNV | c.1528C>T |
| 1 | ANKFN1 | Exon2 | splicing | c.62+2T>C |
| 1 | ANKFN1 | Exon10 | frameshift deletion | c.1155_1156delTG |
| 1 | ANKFN1 | Exon11 | frameshift deletion | c.1290delG |
| 2 | ANKHD1 | Exon21 | stopgain SNV | c.3799G>T |
| 1 | ANKLE1 | Exon5 | frameshift deletion | c.931delA |
| 1 | ANKMY2 | Exon9 | stopgain SNV | c.1036C>T |
| 1 | ANKRD16 | Exon4 | frameshift deletion | c.661delG |
| 1 | ANKRD18B | Exon3 | stopgain SNV | c.443C>A |
| 1 | ANKRD1 | Exon4 | splicing | c.208-1G>C |
| 5 | ANKRD20A4 | Exon6 | frameshift deletion | c.775_778delCTTA |
| 7 | ANKRD20A4 | Exon15 | frameshift deletion | c.2319_2322delGAAA |
| 1 | ANKRD22 | Exon6 | splicing | c.498+1G>A |
| 1 | ANKRD23 | Exon4 | frameshift deletion | c.334_343delCTGGAGATGT |
| 1 | ANKRD24 | Exon12 | frameshift deletion | c.895delC |
| 1 | ANKRD27 | Exon27 | frameshift deletion | c._del |
| 1 | ANKRD30A | Exon1 | stopgain SNV | c.46C>T |
| 17 | ANKRD30A | Exon3 | stopgain SNV | c.328G>T |
| 6 | ANKRD30A | Exon25 | splicing | c.2344+1G>T |
| 20 | ANKRD30A | Exon27 | frameshift deletion | c.2443delG |
| 1 | ANKRD30A | Exon32 | frameshift deletion | c.2869_2872delTTAG |
| 4 | ANKRD30A | Exon34 | frameshift deletion | c.3771_3772delTG |
| 2 | ANKRD35 | Exon10 | stopgain SNV | c.1510C>T |
| 1 | ANKRD35 | Exon10 | frameshift insertion | c.1804dupG |
| 1 | ANKRD35 | Exon10 | stopgain SNV | c.2770C>T |
| 1 | ANKRD35 | Exon13 | stoploss SNV | c.3004T>A |
| 3 | ANKRD36B | Exon41 | frameshift deletion | c.3883delA |
| 1 | ANKRD36B | Exon39 | frameshift deletion | c.3390delA |
| 1 | ANKRD36B | Exon26 | splicing | c.1710+1_1710+1delG |
| 12 | ANKRD36 | Exon1 | frameshift deletion | c.12delC |
| 1 | ANKRD36 | Exon40 | splicing | c.2479-1G>A |
| 2 | ANKRD36 | Exon59 | splicing | c.3498+1_3498+1delG |
| 1 | ANKRD36 | Exon69 | frameshift insertion | c.4270_4271insA |
| 1 | ANKRD36 | Exon71 | frameshift deletion | c.5154delA |
| 5 | ANKRD36 | Exon73 | frameshift deletion | c.5647delA |
| 12 | ANKRD36 | Exon75 | frameshift insertion | c.5809dupC |
| 1 | ANKRD37 | Exon2 | frameshift insertion | c.83_84insCC |
| 1 | ANKRD6 | Exon5 | splicing | c.319-2A>G |
| 1 | ANLN | Exon2 | stopgain SNV | c.31C>T |
| 1 | ANLN | Exon4 | splicing | c.873+2_873+2delT |
| 1 | ANO10 | Exon2 | frameshift insertion | c.132dupA |
| 1 | ANO1 | Exon18 | stopgain SNV | c.1798A>T |
| 3 | ANO5 | Exon5 | frameshift insertion | c.191dupA |
| 1 | ANO5 | Exon10 | stopgain SNV | c.892G>T |
| 9 | ANO7 | Exon24 | frameshift deletion | c.2741_2742delCA |
| 3 | ANO8 | Exon15 | splicing | c.2418+2T>G |
| 1 | ANPEP | Exon18 | stopgain SNV | c.2449C>T |
| 4 | ANTXR2 | Exon13 | frameshift insertion | c.1073dupC |
| 10 | ANTXR2 | Exon13 | frameshift deletion | c.1069delG |
| 1 | ANUBL1 | Exon9 | frameshift deletion | c.2010_2011delTG |
| 1 | ANUBL1 | Exon8 | splicing | c.1872+1G>T |
| 1 | ANUBL1 | Exon7 | frameshift deletion | c.1064_1065delCT |
| 1 | ANXA10 | Exon1 | splicing | c.18+2T>C |
| 4 | ANXA10 | Exon2 | stopgain SNV | c.70C>T |
| 1 | ANXA5 | Exon9 | stopgain SNV | c.601C>T |
| 1 | ANXA7 | Exon14 | stopgain SNV | c.1435C>T |
| 3 | AOAH | Exon19 | stopgain SNV | c.1453G>T |
| 1 | AOAH | Exon18 | frameshift insertion | c.1363dupA |
| 1 | AOC2 | Exon1 | frameshift deletion | c.114delC |
| 4 | AOC2 | Exon1 | stopgain SNV | c.140G>A |
| 1 | AOC2 | Exon1 | frameshift insertion | c.559_560insT |
| 1 | AOC2 | Exon1 | frameshift insertion | c.764dupT |
| 1 | AOC2 | Exon1 | frameshift deletion | c.770delA |
| 1 | AOC2 | Exon1 | frameshift deletion | c.1102_1108delGCCGATT |
| 1 | AOC2 | Exon1 | frameshift deletion | c.1244_1245delCA |
| 1 | AOC2 | Exon1 | frameshift insertion | c.1254_1255insA |
| 1 | AOC2 | Exon1 | frameshift deletion | c.1254delG |
| 1 | AOX1 | Exon16 | stopgain SNV | c.1626T>G |
| 2 | AOX1 | Exon26 | stopgain SNV | c.2896C>T |
| 1 | AP1G2 | Exon12 | splicing | c.1091+1G>A |
| 1 | AP1M1 | Exon9 | frameshift insertion | c.909_910insT |
| 7 | AP1M2 | Exon10 | frameshift deletion | c.1127_1128delTC |
| 1 | AP1M2 | Exon10 | frameshift deletion | c.1111delG |
| 1 | AP1M2 | Exon3 | frameshift deletion | c.200delT |
| 2 | AP1M2 | Exon4 | splicing | c.200-1G>T |
| 1 | AP1S1 | Exon4 | frameshift insertion | c.364dupG |
| 1 | AP2A1 | Exon12 | frameshift insertion | c.1547_1548insT |
| 1 | AP2A2 | Exon14 | frameshift deletion | c.1825delT |
| 1 | AP2A2 | Exon14 | frameshift deletion | c.1944delT |
| 1 | AP2B1 | Exon2 | splicing | c.37+2T>C |
| 2 | AP2B1 | Exon6 | frameshift deletion | c.532_533delGC |
| 4 | AP2M1 | Exon11 | stoploss SNV | c.1294T>C |
| 6 | AP3B2 | Exon10 | frameshift insertion | c.1166dupA |
| 20 | AP3M1 | Exon6 | stopgain SNV | c.772C>T |
| 2 | AP3M2 | Exon5 | frameshift insertion | c.636dupG |
| 1 | AP3M2 | Exon6 | frameshift insertion | c.716dupT |
| 1 | AP3S1 | Exon2 | frameshift deletion | c.121_124delAAGA |
| 1 | AP4B1 | Exon7 | frameshift deletion | c.1160_1161delCA |
| 1 | AP4E1 | Exon6 | frameshift deletion | c.567delT |
| 1 | AP4M1 | Exon13 | stopgain SNV | c.1012C>T |
| 1 | APBA1 | Exon2 | frameshift deletion | c.15_18delGGGG |
| 1 | APC | Exon9 | stopgain SNV | c.1316C>G |
| 1 | APC | Exon14 | frameshift deletion | c.3772delT |
| 1 | APC | Exon14 | frameshift insertion | c.4638dupA |
| 1 | APCS | Exon2 | frameshift insertion | c.482dupG |
| 1 | APEX1 | Exon4 | frameshift deletion | c.285delA |
| 1 | APEX1 | Exon4 | frameshift deletion | c.301_307delGAGAACA |
| 1 | APEX1 | Exon5 | frameshift insertion | c.648dupA |
| 1 | APEX2 | Exon6 | frameshift insertion | c.661_662insT |
| 4 | APIP | Exon3 | stopgain SNV | c.199C>T |
| 13 | APLF | Exon10 | frameshift deletion | c.1528delA |
| 1 | APOA2 | Exon4 | splicing | c.53-15_53-6del  TGTGTGTGTG |
| 3 | APOA2 | Exon4 | splicing | c.53-17_53-6del  TGTGTGTGTGTG |
| 5 | APOA2 | Exon4 | splicing | c.53-11_53-6del  TGTGTG |
| 3 | APOBEC1 | Exon3 | stopgain SNV | c.62G>A |
| 1 | APOBEC3A | Exon3 | frameshift deletion | c.235delG |
| 1 | APOBEC3B | Exon2 | splicing | c.174+1G>T |
| 2 | APOBEC3B | Exon6 | frameshift deletion | c.784delG |
| 1 | APOBEC3G | Exon3 | stopgain SNV | c.394C>T |
| 1 | APOB | Exon26 | stopgain SNV | c.7564C>T |
| 4 | APOB | Exon26 | stopgain SNV | c.6086T>G |
| 1 | APOL4 | Exon6 | stoploss SNV | c.693A>G |
| 20 | APOL4 | Exon4 | splicing | c.35+2T>G |
| 1 | APOL5 | Exon3 | stopgain SNV | c.343C>T |
| 4 | APOL5 | Exon4 | frameshift deletion | c.1250delC |
| 1 | APPBP2 | Exon1 | frameshift deletion | c.39_40delCT |
| 3 | APPL1 | Exon6 | splicing | c.374-1G>A |
| 5 | APPL1 | Exon15 | stopgain SNV | c.1423G>T |
| 1 | APPL1 | Exon21 | frameshift deletion | c.1930_1933delAGAG |
| 1 | APTX | Exon6 | stopgain SNV | c.781C>T |
| 1 | APTX | Exon5 | stopgain SNV | c.554delT |
| 7 | APTX | Exon7 | splicing | c.484-25_484-5delGTTTTTTTTTTTGTTTTTTTT |
| 1 | AQP10 | Exon6 | frameshift insertion | c.812_813insT |
| 5 | AQP12A | Exon2 | frameshift deletion | c.419delG |
| 1 | AQP12B | Exon1 | stopgain SNV | c.405G>A |
| 2 | AQP6 | Exon4 | frameshift deletion | c.781delG |
| 1 | AQP7 | Exon6 | stopgain SNV | c.506G>A |
| 2 | AQP7 | Exon4 | frameshift deletion | c.191delA |
| 7 | AQP7 | Exon3 | stopgain SNV | c.88_89insGAGGAAGATG |
| 2 | AQP9 | Exon5 | frameshift deletion | c.497delT |
| 7 | ARAP1 | Exon29 | stopgain SNV | c.3932T>A |
| 1 | ARF6 | Exon2 | frameshift deletion | c.218delT |
| 1 | ARF6 | Exon2 | frameshift deletion | c.220delT |
| 1 | ARHGAP10 | Exon8 | splicing | c.703-2A>G |
| 1 | ARHGAP10 | Exon9 | frameshift insertion | c.926dupC |
| 1 | ARHGAP10 | Exon9 | frameshift insertion | c.927dupT |
| 1 | ARHGAP10 | Exon17 | frameshift deletion | c.1512delG |
| 1 | ARHGAP15 | Exon14 | stopgain SNV | c.1415C>G |
| 1 | ARHGAP25 | Exon6 | splicing | c.688-2A>T |
| 1 | ARHGAP27 | Exon5 | frameshift deletion | c.350delA |
| 2 | ARHGAP29 | Exon5 | frameshift deletion | c.491delC |
| 1 | ARHGAP33 | Exon21 | frameshift deletion | c.2195delC |
| 10 | ARHGAP36 | Exon7 | stopgain SNV | c.938C>G |
| 13 | ARHGAP40 | Exon3 | stopgain SNV | c.415C>T |
| 2 | ARHGAP44 | Exon14 | frameshift deletion | c.1158delC |
| 1 | ARHGAP5 | Exon2 | stopgain SNV | c.1438C>T |
| 1 | ARHGAP5 | Exon2 | stopgain SNV | c.1441G>T |
| 8 | ARHGAP5 | Exon2 | frameshift deletion | c.2628delC |
| 1 | ARHGAP8 | Exon11 | frameshift insertion | c.898_899insA |
| 1 | ARHGAP9 | Exon13 | splicing | c.1552+1G>T |
| 1 | ARHGDIA | Exon2 | frameshift deletion | c.128_129delAG |
| 1 | ARHGEF15 | Exon6 | frameshift deletion | c.1136delC |
| 3 | ARHGEF2 | Exon6 | stopgain SNV | c.515C>G |
| 2 | ARHGEF35 | Exon2 | frameshift insertion | c.1275dupC |
| 4 | ARHGEF37 | Exon4 | splicing | c.458+1G>A |
| 1 | ARHGEF40 | Exon3 | splicing | c.1450+2T>G |
| 2 | ARHGEF4 | Exon4 | splicing | c.427+2T>C |
| 1 | ARHGEF4 | Exon5 | frameshift deletion | c.548_549delAG |
| 1 | ARHGEF5 | Exon2 | stopgain SNV | c.616C>T |
| 10 | ARHGEF6 | Exon18 | frameshift deletion | c.1892delA |
| 9 | ARHGEF6 | Exon18 | frameshift deletion | c.1877_1878delTG |
| 8 | ARID1A | Exon14 | frameshift deletion | c.3701delG |
| 2 | ARID4A | Exon20 | splicing | c.3211+1G>A |
| 6 | ARL11 | Exon2 | stopgain SNV | c.446G>A |
| 1 | ARL13B | Exon10 | stopgain SNV | c.1252C>T |
| 1 | ARL6IP1 | Exon3 | frameshift deletion | c.198_202delTCTGT |
| 1 | ARMC3 | Exon2 | frameshift deletion | c.14delT |
| 4 | ARMC3 | Exon6 | frameshift deletion | c.406_407delAT |
| 7 | ARMC3 | Exon16 | stopgain SNV | c.1982C>A |
| 1 | ARMC5 | Exon4 | frameshift deletion | c.2061delG |
| 2 | ARMC6 | Exon5 | stopgain SNV | c.859G>T |
| 1 | ARMCX2 | Exon6 | frameshift deletion | c.1436delC |
| 1 | ARMCX2 | Exon6 | frameshift deletion | c.1297delA |
| 1 | ARMCX6 | Exon4 | frameshift deletion | c.233delC |
| 1 | ARMS2 | Exon1 | frameshift deletion | c.246_247delTC |
| 1 | ARNTL2 | Exon9 | splicing | c.776-1G>A |
| 1 | ARSD | Exon9 | frameshift insertion | c.1302dupG |
| 1 | ARSE | Exon7 | stopgain SNV | c.991G>T |
| 1 | ARSJ | Exon2 | frameshift insertion | c.1611dupC |
| 1 | ART1 | Exon3 | stopgain SNV | c.225T>G |
| 2 | ART3 | Exon10 | stopgain SNV | c.1027A>T |
| 1 | ART5 | Exon3 | frameshift insertion | c.70_71insA |
| 1 | ART5 | Exon3 | frameshift insertion | c.69_70insAC |
| 1 | ARV1 | Exon4 | frameshift deletion | c.518delA |
| 1 | ASAH1 | Exon13 | splicing | c.900-2A>G |
| 1 | ASAH1 | Exon1 | frameshift deletion | c.108_114delCTTTGCT |
| 1 | ASAP2 | Exon22 | frameshift insertion | c.2357dupC |
| 5 | ASB11 | Exon2 | stopgain SNV | c.223C>T |
| 1 | ASB12 | Exon3 | frameshift insertion | c.880_881dupCA |
| 11 | ASB15 | Exon7 | frameshift deletion | c.721delG |
| 2 | ASB15 | Exon7 | frameshift deletion | c.761delG |
| 1 | ASB15 | Exon7 | stopgain SNV | c.844C>T |
| 1 | ASB15 | Exon10 | stopgain SNV | c.1734T>A |
| 1 | ASB18 | Exon2 | frameshift deletion | c.296delA |
| 1 | ASB2 | Exon10 | stopgain SNV | c.1801C>T |
| 1 | ASB6 | Exon5 | stopgain SNV | c.566G>A |
| 1 | ASB8 | Exon4 | frameshift deletion | c.375delA |
| 2 | ASCL3 | Exon2 | frameshift insertion | c.475_476dupAA |
| 3 | ASCL3 | Exon2 | stopgain SNV | c.382C>T |
| 1 | ASCL4 | Exon1 | frameshift deletion | c.515delG |
| 1 | ASH1L | Exon3 | frameshift insertion | c.2574dupG |
| 1 | ASPDH | Exon1 | frameshift deletion | c.20_23delGTAG |
| 1 | ASPN | Exon3 | stopgain SNV | c.313C>T |
| 1 | ASTE1 | Exon6 | frameshift deletion | c.1894delA |
| 3 | ASTL | Exon9 | frameshift deletion | c.1282delA |
| 2 | ASTN1 | Exon15 | frameshift deletion | c.2421delG |
| 1 | ASXL1 | Exon8 | frameshift insertion | c.868_869insC |
| 1 | ATAD5 | Exon1 | frameshift deletion | c.39delG |
| 2 | ATAD5 | Exon8 | frameshift insertion | c.2775dupC |
| 1 | ATF5 | Exon4 | stopgain SNV | c.619C>T |
| 1 | ATF7IP | Exon14 | splicing | c.3281-2A>G |
| 7 | ATG3 | Exon4 | frameshift deletion | c.212_213delAA |
| 3 | ATG4C | Exon8 | frameshift deletion | c.959_960delTG |
| 1 | ATG4D | Exon7 | stopgain SNV | c.990C>A |
| 1 | ATG7 | Exon6 | frameshift deletion | c.488delA |
| 1 | ATG7 | Exon18 | stopgain SNV | c.1975C>T |
| 1 | ATG9A | Exon13 | frameshift deletion | c.2204delG |
| 1 | ATG9A | Exon7 | frameshift deletion | c.540delG |
| 1 | ATL1 | Exon10 | frameshift deletion | c.871delG |
| 1 | ATL1 | Exon13 | stopgain SNV | c.1177C>T |
| 1 | ATM | Exon7 | splicing | c.901+1G>A |
| 2 | ATM | Exon22 | stopgain SNV | c.3169A>T |
| 2 | ATOH8 | Exon2 | frameshift deletion | c.922delC |
| 1 | ATP10B | Exon16 | splicing | c.1813-2A>G |
| 2 | ATP10D | Exon8 | frameshift deletion | c.1039delT |
| 1 | ATP10D | Exon9 | frameshift deletion | c.1166_1167delAT |
| 1 | ATP10D | Exon23 | frameshift deletion | c.4193_4194delTT |
| 1 | ATP11A | Exon28 | frameshift deletion | c.3225delC |
| 2 | ATP11A | Exon28 | frameshift insertion | c.3229dupC |
| 1 | ATP12A | Exon8 | frameshift insertion | c.912dupC |
| 1 | ATP12A | Exon13 | splicing | c.1724-2A>G |
| 1 | ATP13A5 | Exon30 | frameshift deletion | c.3653delT |
| 1 | ATP13A5 | Exon15 | frameshift deletion | c.1709_1710delTT |
| 6 | ATP13A5 | Exon2 | stopgain SNV | c.171delG |
| 1 | ATP1A4 | Exon1 | frameshift insertion | c.72dupG |
| 1 | ATP2B2 | Exon4 | splicing | c.397+1G>A |
| 1 | ATP2B4 | Exon2 | stopgain SNV | c.144delT |
| 1 | ATP2C1 | Exon24 | stopgain SNV | c.2380C>T |
| 1 | ATP2C1 | Exon25 | frameshift deletion | c.2584_2585delGT |
| 1 | ATP2C2 | Exon18 | splicing | c.1661-2A>T |
| 6 | ATP2C2 | Exon25 | splicing | c.2482-2A>C |
| 2 | ATP4A | Exon8 | frameshift deletion | c.1223_1224delTC |
| 3 | ATP5J | Exon3 | frameshift deletion | c.274delA |
| 1 | ATP5L2 | Exon1 | frameshift deletion | c.171_174delCAGT |
| 1 | ATP5O | Exon5 | frameshift deletion | c.410delG |
| 1 | ATP6V0A2 | Exon17 | stopgain SNV | c.2064C>A |
| 3 | ATP6V0D1 | Exon2 | frameshift insertion | c.203dupC |
| 1 | ATP6V1C2 | Exon10 | splicing | c.825+2_825+3delTG |
| 5 | ATP6V1G3 | Exon3 | stopgain SNV | c.106C>T |
| 1 | ATP6V1H | Exon4 | frameshift deletion | c.281delT |
| 1 | ATP7B | Exon2 | frameshift deletion | c.533delT |
| 1 | ATP7B | Exon1 | frameshift deletion | c.19_20delCA |
| 1 | ATP8B2 | Exon28 | frameshift deletion | c.3632delG |
| 4 | ATP8B3 | Exon29 | stoploss SNV | c.3903A>G |
| 2 | ATP8B4 | Exon21 | frameshift insertion | c.2210dupA |
| 6 | ATP9B | Exon5 | stopgain SNV | c.640C>T |
| 1 | ATP9B | Exon16 | frameshift deletion | c.1896delC |
| 1 | ATPAF2 | Exon8 | frameshift deletion | c.794delG |
| 1 | ATPAF2 | Exon4 | frameshift deletion | c.385_386delCG |
| 3 | ATPAF2 | Exon4 | frameshift deletion | c.382delA |
| 1 | ATR | Exon47 | stopgain SNV | c.7816C>T |
| 1 | ATR | Exon25 | frameshift deletion | c.4436delA |
| 1 | ATRX | Exon11 | stopgain SNV | c.3863C>G |
| 6 | ATRX | Exon11 | frameshift deletion | c.3862delT |
| 1 | ATXN2 | Exon18 | splicing | c.2785-1G>A |
| 1 | ATXN3L | Exon1 | stopgain SNV | c.76G>T |
| 1 | AURKB | Exon3 | frameshift insertion | c.49dupG |
| 1 | AURKC | Exon6 | frameshift insertion | c.606_607insA |
| 1 | AVIL | Exon6 | stopgain SNV | c.595C>T |
| 1 | AVPR1B | Exon1 | frameshift deletion | c.888_889delCT |
| 1 | AVPR1B | Exon1 | stopgain SNV | c.917G>A |
| 2 | AWAT1 | Exon4 | frameshift deletion | c.314delA |
| 1 | AZI1 | Exon13 | frameshift insertion | c.1627_1628insTC |
| 1 | AZI1 | Exon13 | frameshift insertion | c.1625_1626insG |
| 1 | AZU1 | Exon5 | frameshift deletion | c.695delT |
| 1 | B3GALNT2 | Exon7 | frameshift insertion | c.824_825dupTT |
| 3 | B3GNT4 | Exon2 | stopgain SNV | c.34G>T |
| 1 | B3GNT4 | Exon3 | stopgain SNV | c.688G>T |
| 2 | B3GNT4 | Exon3 | frameshift deletion | c.703_713delTGGGACCCAGC |
| 2 | B3GNT6 | Exon2 | frameshift deletion | c.1010delT |
| 1 | B3GNTL1 | Exon6 | splicing | c.408+1G>A |
| 1 | B4GALNT3 | Exon9 | stopgain SNV | c.790C>T |
| 3 | B4GALT3 | Exon3 | frameshift deletion | c.200delG |
| 5 | B7H6 | Exon5 | frameshift deletion | c.1346_1347delTT |
| 1 | BACH2 | Exon5 | frameshift insertion | c.1186_1187insT |
| 1 | BAG4 | Exon2 | stopgain SNV | c.308G>A |
| 1 | BAHD1 | Exon2 | stopgain SNV | c.58C>T |
| 1 | BAHD1 | Exon2 | frameshift deletion | c.183delA |
| 1 | BAI2 | Exon4 | frameshift deletion | c.262delC |
| 5 | BAI3 | Exon29 | stopgain SNV | c.3724G>T |
| 4 | BAIAP2L1 | Exon5 | splicing | c.215-1G>A |
| 1 | BAIAP2L1 | Exon3 | stopgain SNV | c.203C>G |
| 1 | BAIAP2L2 | Exon13 | frameshift insertion | c.1493dupC |
| 1 | BARHL1 | Exon3 | frameshift insertion | c.819_820insAA |
| 1 | BARX2 | Exon4 | frameshift insertion | c.627_628insT |
| 1 | BAX | Exon3 | frameshift insertion | c.121dupG |
| 1 | BAZ1A | Exon13 | frameshift insertion | c.1522dupG |
| 5 | BAZ1B | Exon10 | frameshift deletion | c.2937delA |
| 4 | BAZ1B | Exon6 | stopgain SNV | c.787C>T |
| 1 | BAZ2A | Exon24 | frameshift deletion | c.4870_4871delGG |
| 1 | BBOX1 | Exon3 | stopgain SNV | c.103A>T |
| 1 | BBS10 | Exon2 | frameshift deletion | c.2119_2120delGT |
| 1 | BBS12 | Exon3 | frameshift deletion | c.854_855delTT |
| 4 | BBS12 | Exon3 | frameshift deletion | c.1549delG |
| 1 | BBS7 | Exon7 | frameshift deletion | c.712_715delAGAG |
| 1 | BBS9 | Exon11 | splicing | c.1199-1G>A |
| 1 | BCAP29 | Exon4 | frameshift deletion | c.316delA |
| 1 | BCAT1 | Exon3 | stopgain SNV | c.244C>T |
| 1 | BCCIP | Exon1 | frameshift insertion | c.24dupT |
| 1 | BCCIP | Exon5 | splicing | c.599+1G>A |
| 1 | BCDIN3D | Exon2 | frameshift deletion | c.377delC |
| 1 | BCHE | Exon2 | frameshift insertion | c.435_436insG |
| 2 | BCL2L12 | Exon4 | stopgain SNV | c.556C>T |
| 1 | BCL2L14 | Exon2 | frameshift deletion | c.402_405delTTCT |
| 2 | BCL2L2-PABPN1 | Exon3 | frameshift insertion | c.364_365insC |
| 1 | BCL3 | Exon6 | frameshift deletion | c.831delC |
| 1 | BCL6 | Exon5 | frameshift deletion | c.406_407delAT |
| 1 | BCL7C | Exon6 | frameshift insertion | c.618dupA |
| 1 | BCL9L | Exon7 | frameshift insertion | c.3380dupC |
| 1 | BCL9 | Exon8 | frameshift insertion | c.1547dupC |
| 6 | BCLAF1 | Exon12 | frameshift deletion | c._del |
| 1 | BCLAF1 | Exon11 | frameshift deletion | c.2528delG |
| 5 | BCLAF1 | Exon5 | stopgain SNV | c.1207G>T |
| 4 | BCLAF1 | Exon4 | frameshift insertion | c.109_110insCA |
| 4 | BCLAF1 | Exon4 | frameshift deletion | c.108delT |
| 1 | BCO2 | Exon5 | frameshift insertion | c.653_654insG |
| 1 | BCORL1 | Exon3 | frameshift insertion | c.3243_3244insTT |
| 1 | BCOR | Exon4 | frameshift deletion | c.2414_2415delAC |
| 2 | BDKRB1 | Exon3 | stopgain SNV | c.844C>T |
| 1 | BEND2 | Exon2 | frameshift deletion | c.213delT |
| 1 | BEND2 | Exon2 | frameshift deletion | c.39_40delAA |
| 1 | BEND5 | Exon4 | frameshift deletion | c.850_854delGCTCC |
| 2 | BEND7 | Exon9 | frameshift deletion | c.1404_1405delCT |
| 1 | BEST3 | Exon4 | frameshift deletion | c.285_288delCCAG |
| 1 | BEST3 | Exon4 | frameshift deletion | c.279_282delGTGG |
| 13 | BEST3 | Exon3 | frameshift deletion | c.177delA |
| 2 | BFSP1 | Exon8 | frameshift deletion | c.1621_1622delTA |
| 2 | BFSP1 | Exon8 | frameshift deletion | c.1620_1621delTT |
| 1 | BFSP1 | Exon3 | splicing | c.63+1G>A |
| 1 | BFSP2 | Exon1 | frameshift deletion | c.482delG |
| 1 | BGLAP | Exon3 | stopgain SNV | c.156C>G |
| 1 | BHMT2 | Exon4 | splicing | c.406+1G>A |
| 1 | BICD2 | Exon7 | frameshift deletion | c.2435_2436delAA |
| 1 | BIRC6 | Exon10 | frameshift deletion | c.2768delT |
| 3 | BIRC6 | Exon42 | stopgain SNV | c.7988T>A |
| 1 | BLM | Exon3 | frameshift deletion | c.772_773delCT |
| 1 | BLM | Exon8 | frameshift deletion | c.1980delT |
| 1 | BLVRA | Exon5 | stopgain SNV | c.332G>A |
| 1 | BLZF1 | Exon3 | stopgain SNV | c.424C>T |
| 1 | BLZF1 | Exon7 | stopgain SNV | c.1123C>T |
| 6 | BMP2K | Exon16 | stopgain SNV | c.3481C>T |
| 1 | BMP7 | Exon2 | frameshift deletion | c.607_608delGG |
| 1 | BMP8A | Exon7 | splicing | c.1060-2A>T |
| 2 | BMPR2 | Exon12 | frameshift deletion | c.2420_2421delAA |
| 1 | BMX | Exon2 | frameshift deletion | c.93delT |
| 1 | BORA | Exon11 | frameshift deletion | c.1575_1576delAT |
| 1 | BPGM | Exon4 | stoploss SNV | c.778delT |
| 3 | BPHL | Exon3 | splicing | c.378+2T>C |
| 1 | BPIFA1 | Exon5 | splicing | c.581+1G>C |
| 1 | BPIFA2 | Exon7 | stopgain SNV | c.697C>T |
| 1 | BPIFA3 | Exon3 | stopgain SNV | c.326C>A |
| 1 | BPIFA3 | Exon4 | stopgain SNV | c.495C>A |
| 1 | BPIFB3 | Exon1 | stopgain SNV | c.64C>T |
| 6 | BPIFB3 | Exon4 | splicing | c.387-1G>T |
| 1 | BPIFB6 | Exon1 | stopgain SNV | c.49C>T |
| 1 | BPIFB6 | Exon8 | frameshift deletion | c.796delG |
| 1 | BPI | Exon13 | frameshift insertion | c.1330dupG |
| 1 | BRAP | Exon12 | frameshift insertion | c.1574_1575insC |
| 1 | BRCA1 | Exon24 | stopgain SNV | c.5508G>A |
| 1 | BRCA1 | Exon23 | frameshift deletion | c.5398delC |
| 7 | BRCA1 | Exon21 | frameshift insertion | c.5329dupC |
| 1 | BRCA1 | Exon20 | frameshift deletion | c.5240_5243delGAAA |
| 1 | BRCA1 | Exon18 | frameshift deletion | c.5093_5096delCTAA |
| 1 | BRCA1 | Exon12 | frameshift insertion | c.4182_4183dupTC |
| 2 | BRCA1 | Exon11 | frameshift deletion | c.4035delA |
| 1 | BRCA1 | Exon11 | frameshift deletion | c.3756_3759delGTCT |
| 1 | BRCA1 | Exon11 | stopgain SNV | c.3403C>T |
| 2 | BRCA1 | Exon11 | frameshift deletion | c.2475delC |
| 1 | BRCA1 | Exon11 | frameshift deletion | c.2457delC |
| 1 | BRCA1 | Exon11 | stopgain SNV | c.2389G>T |
| 1 | BRCA1 | Exon11 | stopgain SNV | c.2138C>G |
| 1 | BRCA1 | Exon11 | frameshift deletion | c.1961delA |
| 1 | BRCA1 | Exon11 | frameshift deletion | c.1510delC |
| 1 | BRCA1 | Exon11 | stopgain SNV | c.1499_1508dup  ATAAATTAAA |
| 1 | BRCA1 | Exon11 | stopgain SNV | c.1115G>A |
| 1 | BRCA1 | Exon10 | splicing | c.594-2A>C |
| 1 | BRCA1 | Exon8 | frameshift deletion | c.514delC |
| 1 | BRCA1 | Exon5 | splicing | c.135-1G>T |
| 8 | BRCA1 | Exon2 | frameshift deletion | c.68_69delAG |
| 1 | BRCA2 | Exon7 | splicing | c.631+2T>G |
| 1 | BRCA2 | Exon9 | frameshift deletion | c.771_775delTCAAA |
| 1 | BRCA2 | Exon10 | stopgain SNV | c.880G>T |
| 1 | BRCA2 | Exon10 | frameshift deletion | c.1029delA |
| 1 | BRCA2 | Exon10 | frameshift insertion | c.1813dupA |
| 1 | BRCA2 | Exon11 | frameshift deletion | c.3847_3848delGT |
| 1 | BRCA2 | Exon11 | frameshift deletion | c.4563_4564delAT |
| 1 | BRCA2 | Exon11 | stopgain SNV | c.4889C>G |
| 1 | BRCA2 | Exon11 | frameshift deletion | c.5116_5119delAATA |
| 1 | BRCA2 | Exon11 | frameshift deletion | c.5130_5133delTGTA |
| 1 | BRCA2 | Exon11 | frameshift deletion | c.5213_5216delCTTA |
| 1 | BRCA2 | Exon11 | frameshift deletion | c.5351_5352delAA |
| 1 | BRCA2 | Exon11 | frameshift deletion | c.5569_5573delGAAAC |
| 8 | BRCA2 | Exon11 | frameshift deletion | c.5946delT |
| 1 | BRCA2 | Exon11 | stopgain SNV | c.6037A>T |
| 1 | BRCA2 | Exon11 | frameshift deletion | c.6405_6409delCTTAA |
| 1 | BRCA2 | Exon14 | stopgain SNV | c.7180A>T |
| 1 | BRCA2 | Exon15 | stopgain SNV | c.7558C>T |
| 1 | BRCA2 | Exon27 | stopgain SNV | c.9976A>T |
| 2 | BRD4 | Exon2 | stopgain SNV | c.250C>T |
| 1 | BRD9 | Exon15 | frameshift deletion | c.1681delC |
| 1 | BRDT | Exon12 | stopgain SNV | c.1943C>G |
| 1 | BRIP1 | Exon17 | frameshift deletion | c.2489delG |
| 1 | BRIP1 | Exon15 | frameshift insertion | c.2108_2109insCC |
| 1 | BRIP1 | Exon9 | frameshift deletion | c.1236delA |
| 2 | BRIX1 | Exon10 | splicing | c.793-2dupA |
| 1 | BRMS1 | Exon6 | splicing | c.359-2A>G |
| 5 | BROX | Exon2 | frameshift deletion | c.27delA |
| 2 | BROX | Exon8 | frameshift deletion | c.583delA |
| 5 | BROX | Exon8 | frameshift deletion | c.595delG |
| 2 | BRP44 | Exon3 | frameshift deletion | c.126delC |
| 1 | BRPF1 | Exon3 | stopgain SNV | c.1145G>A |
| 4 | BRPF1 | Exon6 | frameshift insertion | c.1917_1918insT |
| 1 | BRSK1 | Exon8 | frameshift deletion | c.712delC |
| 1 | BRSK1 | Exon8 | frameshift insertion | c.792_793insG |
| 1 | BRSK1 | Exon8 | frameshift deletion | c.799_800delGA |
| 1 | BRSK1 | Exon18 | splicing | c.2090-2dupA |
| 1 | BRWD3 | Exon17 | frameshift deletion | c.1761delA |
| 1 | BSN | Exon5 | frameshift deletion | c.3018delT |
| 1 | BSN | Exon6 | frameshift deletion | c.9234_9237delCCAG |
| 1 | BSPRY | Exon6 | stoploss SNV | c.1207T>G |
| 3 | BTAF1 | Exon12 | stopgain SNV | c.1361T>A |
| 1 | BTBD6 | Exon5 | frameshift insertion | c.547_548insG |
| 1 | BTBD6 | Exon5 | frameshift insertion | c.841dupT |
| 1 | BTBD7 | Exon3 | frameshift deletion | c.192delC |
| 1 | BTBD7 | Exon3 | frameshift insertion | c.184_185insT |
| 4 | BTD | Exon4 | stopgain SNV | c.1505C>G |
| 1 | BTK | Exon17 | frameshift deletion | c.1701delA |
| 1 | BTK | Exon15 | stopgain SNV | c.1396A>T |
| 2 | BTN2A1 | Exon4 | stopgain SNV | c.442A>T |
| 1 | BTN2A2 | Exon3 | splicing | c.95-1G>A |
| 1 | BTN3A2 | Exon5 | stopgain SNV | c.646G>T |
| 6 | BTN3A3 | Exon5 | stopgain SNV | c.513G>A |
| 7 | BTNL9 | Exon4 | frameshift deletion | c.622delC |
| 1 | BUD31 | Exon4 | frameshift insertion | c.169_170insT |
| 1 | BYSL | Exon6 | stopgain SNV | c.910G>T |
| 1 | C10ORF10 | Exon2 | stopgain SNV | c.184C>T |
| 1 | C10ORF113 | Exon3 | splicing | c.168-1G>A |
| 3 | C10ORF11 | Exon1 | frameshift deletion | c.21delC |
| 1 | C10ORF120 | Exon3 | stopgain SNV | c.628C>T |
| 2 | C10ORF128 | Exon5 | stopgain SNV | c.271C>T |
| 1 | C10ORF12 | Exon1 | frameshift insertion | c.2244_2245insG |
| 1 | C10ORF12 | Exon1 | frameshift deletion | c.3411delC |
| 1 | C10ORF131 | Exon7 | stopgain SNV | c.257C>G |
| 3 | C10ORF27 | Exon7 | frameshift deletion | c.676delG |
| 3 | C10ORF27 | Exon4 | stopgain SNV | c.148G>T |
| 2 | C10ORF32 | Exon1 | splicing | c.141+1G>C |
| 2 | C10ORF53 | Exon2 | stopgain SNV | c.195C>A |
| 1 | C10ORF53 | Exon2 | frameshift insertion | c.201dupT |
| 1 | C10ORF62 | Exon1 | stopgain SNV | c.457C>T |
| 1 | C10ORF68 | Exon3 | stopgain SNV | c.21C>G |
| 10 | C10ORF68 | Exon9 | frameshift deletion | c.675_678delAAAT |
| 1 | C10ORF71 | Exon3 | frameshift deletion | c.1655delC |
| 1 | C10ORF71 | Exon3 | frameshift insertion | c.1923_1924insTA |
| 1 | C10ORF90 | Exon4 | stopgain SNV | c.1414C>T |
| 1 | C10ORF91 | Exon3 | frameshift insertion | c.310_311insT |
| 1 | C10ORF91 | Exon3 | frameshift insertion | c.390_391insG |
| 1 | C10ORF91 | Exon3 | frameshift deletion | c.400_401delGT |
| 1 | C10ORF92 | Exon22 | stopgain SNV | c.2584C>T |
| 2 | C11ORF16 | Exon6 | frameshift deletion | c.1377_1378delAT |
| 11 | C11ORF1 | Exon4 | stopgain SNV | c.443C>A |
| 1 | C11ORF40 | Exon3 | splicing | c.308+1G>C |
| 3 | C11ORF40 | Exon3 | splicing | c.142-1G>A |
| 1 | C11ORF54 | Exon4 | stopgain SNV | c.201C>G |
| 1 | C11ORF54 | Exon8 | frameshift insertion | c.641_642insCAAA |
| 1 | C11ORF63 | Exon5 | frameshift deletion | c.1396_1403delGTTAATAA |
| 2 | C11ORF65 | Exon9 | stopgain SNV | c.876C>A |
| 1 | C11ORF65 | Exon4 | stopgain SNV | c.178G>T |
| 2 | C11ORF71 | Exon1 | stopgain SNV | c.57C>G |
| 1 | C11ORF82 | Exon6 | frameshift deletion | c.2709_2718delGAAACTGAAA |
| 1 | C11ORF84 | Exon4 | splicing | c.733+2T>G |
| 3 | C12ORF26 | Exon2 | frameshift deletion | c.295delT |
| 1 | C12ORF35 | Exon4 | stopgain SNV | c.4739T>G |
| 1 | C12ORF39 | Exon3 | splicing | c.88-2A>C |
| 1 | C12ORF48 | Exon3 | frameshift deletion | c.376_377delAC |
| 1 | C12ORF49 | Exon5 | stopgain SNV | c.585T>G |
| 1 | C12ORF4 | Exon7 | stopgain SNV | c.793G>T |
| 1 | C12ORF50 | Exon12 | stopgain SNV | c.1171C>T |
| 1 | C12ORF56 | Exon10 | stopgain SNV | c.1179C>A |
| 1 | C12ORF56 | Exon10 | frameshift deletion | c.1156delT |
| 1 | C12ORF65 | Exon2 | frameshift insertion | c.96_99dupATCC |
| 1 | C12ORF69 | Exon2 | stopgain SNV | c.172A>T |
| 1 | C12ORF74 | Exon2 | stopgain SNV | c.490C>T |
| 1 | C13ORF16 | Exon5 | frameshift deletion | c.240delA |
| 1 | C13ORF16 | Exon5 | frameshift deletion | c.253delA |
| 1 | C14ORF105 | Exon3 | stopgain SNV | c.331C>T |
| 3 | C14ORF118 | Exon7 | stopgain SNV | c.1084C>T |
| 5 | C14ORF133 | Exon18 | frameshift deletion | c.1332delC |
| 1 | C14ORF133 | Exon7 | stopgain SNV | c.484C>T |
| 1 | C14ORF135 | Exon8 | frameshift deletion | c.1730_1733delACAG |
| 1 | C14ORF176 | Exon6 | frameshift deletion | c.305delT |
| 2 | C14ORF182 | Exon1 | frameshift deletion | c.22delC |
| 1 | C14ORF183 | Exon2 | frameshift deletion | c.206delG |
| 1 | C14ORF39 | Exon14 | splicing | c.1059-1G>T |
| 1 | C14ORF93 | Exon7 | splicing | c.1197+1G>T |
| 1 | C15ORF32 | Exon1 | stopgain SNV | c.88A>T |
| 1 | C15ORF33 | Exon13 | stopgain SNV | c.1122_1125dupTAGT |
| 1 | C15ORF33 | Exon8 | stopgain SNV | c.621G>A |
| 1 | C15ORF59 | Exon2 | frameshift insertion | c.866_867insG |
| 3 | C16ORF55 | Exon3 | frameshift insertion | c.406_407insTG |
| 1 | C16ORF62 | Exon3 | stopgain SNV | c.403A>T |
| 2 | C16ORF62 | Exon4 | stopgain SNV | c.602C>A |
| 1 | C16ORF89 | Exon2 | stopgain SNV | c.241C>T |
| 1 | C16ORF93 | Exon8 | frameshift deletion | c.779_780delAG |
| 2 | C17ORF101 | Exon5 | splicing | c.423+1G>A |
| 3 | C17ORF101 | Exon4 | splicing | c.380+1G>A |
| 3 | C17ORF104 | Exon8 | stopgain SNV | c.2770C>T |
| 2 | C17ORF28 | Exon5 | splicing | c.388-1G>T |
| 1 | C17ORF56 | Exon5 | splicing | c.214-2_214-1insAA |
| 1 | C17ORF57 | Exon9 | frameshift insertion | c.650_651insG |
| 5 | C17ORF57 | Exon10 | stopgain SNV | c.706C>T |
| 12 | C17ORF57 | Exon11 | splicing | c.806-1G>A |
| 7 | C17ORF57 | Exon12 | stopgain SNV | c.1297A>T |
| 1 | C17ORF57 | Exon18 | frameshift insertion | c.1947dupA |
| 5 | C17ORF58 | Exon2 | stoploss SNV | c.226T>C |
| 1 | C17ORF63 | Exon4 | stopgain SNV | c.754C>T |
| 2 | C17ORF66 | Exon4 | stopgain SNV | c.400C>T |
| 1 | C17ORF74 | Exon3 | frameshift deletion | c.427delC |
| 7 | C17ORF77 | Exon3 | stopgain SNV | c.253G>T |
| 2 | C17ORF80 | Exon4 | splicing | c.1621+1G>C |
| 1 | C17ORF81 | Exon5 | stopgain SNV | c.405C>A |
| 1 | C17ORF81 | Exon7 | stopgain SNV | c.682C>T |
| 3 | C17ORF85 | Exon12 | stopgain SNV | c.569T>G |
| 2 | C18ORF21 | Exon5 | stopgain SNV | c.622A>T |
| 1 | C18ORF34 | Exon17 | frameshift insertion | c.1662dupA |
| 2 | C18ORF34 | Exon10 | frameshift deletion | c.874delA |
| 2 | C18ORF34 | Exon9 | splicing | c.457+2T>C |
| 1 | C19ORF21 | Exon2 | stopgain SNV | c.922C>T |
| 1 | C19ORF21 | Exon3 | splicing | c.1911+2T>A |
| 3 | C19ORF47 | Exon8 | frameshift deletion | c.531_532delCC |
| 1 | C19ORF48 | Exon5 | stopgain SNV | c.238G>T |
| 3 | C19ORF55 | Exon9 | frameshift deletion | c.1193delG |
| 1 | C19ORF57 | Exon8 | frameshift insertion | c.1847_1850dupCCCT |
| 2 | C19ORF59 | Exon3 | stopgain SNV | c.236T>A |
| 2 | C19ORF59 | Exon6 | stopgain SNV | c.547C>T |
| 1 | C19ORF75 | Exon3 | frameshift insertion | c.120dupG |
| 1 | C19ORF75 | Exon4 | splicing | c.410+1G>A |
| 1 | C1ORF105 | Exon7 | frameshift deletion | c.510_511delCT |
| 1 | C1ORF112 | Exon12 | frameshift insertion | c.1102dupA |
| 1 | C1ORF112 | Exon13 | stopgain SNV | c.1201C>T |
| 1 | C1ORF122 | Exon3 | frameshift insertion | c.268dupC |
| 9 | C1ORF127 | Exon12 | splicing | c.1290+2C>T |
| 3 | C1ORF135 | Exon3 | frameshift deletion | c.780_789delATCAGTGAAA |
| 1 | C1ORF135 | Exon3 | frameshift deletion | c.703delG |
| 1 | C1ORF135 | Exon2 | frameshift deletion | c.117_118delCC |
| 1 | C1ORF135 | Exon2 | frameshift deletion | c.115delC |
| 5 | C1ORF141 | Exon7 | frameshift deletion | c.1017_1021delAATGA |
| 1 | C1ORF158 | Exon2 | stopgain SNV | c.139C>T |
| 1 | C1ORF158 | Exon3 | splicing | c.403+1G>A |
| 1 | C1ORF168 | Exon17 | frameshift insertion | c.1905_1906dupAA |
| 1 | C1ORF168 | Exon13 | splicing | c.1544-2A>G |
| 6 | C1ORF168 | Exon10 | stopgain SNV | c.1420G>T |
| 2 | C1ORF168 | Exon7 | splicing | c.1098+2T>A |
| 1 | C1ORF168 | Exon6 | splicing | c.954-1G>A |
| 1 | C1ORF168 | Exon3 | frameshift insertion | c.850dupA |
| 1 | C1ORF168 | Exon2 | frameshift deletion | c.728_729delAT |
| 1 | C1ORF173 | Exon12 | frameshift deletion | c.2092delG |
| 5 | C1ORF177 | Exon7 | frameshift insertion | c.770dupA |
| 2 | C1ORF177 | Exon10 | stopgain SNV | c.1203G>A |
| 2 | C1ORF187 | Exon3 | frameshift deletion | c.528delC |
| 6 | C1ORF189 | Exon4 | frameshift deletion | c.299_300delGA |
| 1 | C1ORF189 | Exon4 | stopgain SNV | c.271C>T |
| 1 | C1ORF210 | Exon3 | stopgain SNV | c.180C>A |
| 1 | C1ORF216 | Exon2 | frameshift deletion | c.467delT |
| 1 | C1ORF216 | Exon2 | stopgain SNV | c.256G>T |
| 4 | C1ORF227 | Exon2 | stopgain SNV | c.109C>T |
| 1 | C1ORF50 | Exon3 | stopgain SNV | c.211C>T |
| 1 | C1ORF51 | Exon1 | frameshift deletion | c.285_292delAAGATCAA |
| 1 | C1ORF85 | Exon6 | splicing | c.799-2A>G |
| 1 | C1ORF87 | Exon6 | stopgain SNV | c.760A>T |
| 1 | C1ORF88 | Exon4 | frameshift insertion | c.293dupT |
| 1 | C1ORF9 | Exon24 | stopgain SNV | c.3610C>T |
| 1 | C1QB | Exon3 | frameshift deletion | c.596delA |
| 1 | C1QBP | Exon6 | splicing | c.699+1G>A |
| 1 | C1QC | Exon3 | frameshift insertion | c.357_358insC |
| 1 | C1QC | Exon3 | frameshift deletion | c.494delT |
| 1 | C1QTNF1 | Exon4 | stopgain SNV | c.721C>T |
| 1 | C1QTNF2 | Exon2 | frameshift insertion | c.316_317insCA |
| 1 | C1QTNF2 | Exon2 | stopgain SNV | c.310C>T |
| 1 | C1QTNF3 | Exon2 | frameshift insertion | c.182dupC |
| 2 | C1QTNF5 | Exon15 | frameshift insertion | c.583dupG |
| 1 | C1QTNF9 | Exon3 | stopgain SNV | c.226C>T |
| 1 | C1RL | Exon6 | frameshift deletion | c.982delT |
| 1 | C1R | Exon2 | frameshift deletion | c.152_153delCC |
| 1 | C20ORF106 | Exon1 | stopgain SNV | c.97C>T |
| 1 | C20ORF107 | Exon2 | frameshift insertion | c.311dupA |
| 1 | C20ORF118 | Exon3 | frameshift deletion | c.299delG |
| 2 | C20ORF132 | Exon21 | frameshift deletion | c.2825delT |
| 2 | C20ORF132 | Exon15 | frameshift deletion | c.2076_2077delGA |
| 2 | C20ORF132 | Exon15 | frameshift deletion | c.2076delG |
| 2 | C20ORF141 | Exon1 | frameshift deletion | c.176_179delTGAC |
| 1 | C20ORF152 | Exon1 | stopgain SNV | c.30G>A |
| 1 | C20ORF26 | Exon6 | stopgain SNV | c.544C>T |
| 1 | C20ORF26 | Exon27 | frameshift deletion | c.3522_3526delTCTTC |
| 1 | C20ORF4 | Exon2 | frameshift insertion | c.352dupC |
| 1 | C20ORF85 | Exon3 | frameshift insertion | c.248dupT |
| 1 | C20ORF94 | Exon8 | frameshift deletion | c.921_924delCCAC |
| 1 | C20ORF94 | Exon8 | frameshift deletion | c.1110_1111delTT |
| 1 | C20ORF96 | Exon7 | splicing | c.565+1G>A |
| 1 | C21ORF2 | Exon6 | frameshift deletion | c.620_621delCC |
| 1 | C21ORF33 | Exon4 | frameshift insertion | c.349dupA |
| 1 | C21ORF59 | Exon6 | stopgain SNV | c.735C>G |
| 2 | C22ORF15 | Exon4 | stopgain SNV | c.253G>T |
| 2 | C22ORF15 | Exon5 | stopgain SNV | c.394C>T |
| 1 | C22ORF23 | Exon4 | frameshift deletion | c.360_363delAAAG |
| 1 | C22ORF25 | Exon3 | stopgain SNV | c.120G>A |
| 1 | C22ORF29 | Exon3 | frameshift insertion | c.555dupT |
| 1 | C22ORF29 | Exon3 | stopgain SNV | c.451C>T |
| 1 | C22ORF31 | Exon2 | frameshift deletion | c.288_291delTGGA |
| 1 | C22ORF33 | Exon4 | stopgain SNV | c.376C>T |
| 2 | C22ORF33 | Exon4 | splicing | c.234+2T>C |
| 1 | C22ORF42 | Exon5 | splicing | c.400+1G>A |
| 1 | C22ORF42 | Exon1 | frameshift deletion | c.109delG |
| 4 | C22ORF42 | Exon1 | frameshift insertion | c.102dupG |
| 1 | C2CD3 | Exon27 | frameshift insertion | c.5176_5177insG |
| 1 | C2CD3 | Exon9 | splicing | c.1218-2A>G |
| 1 | C2ORF3 | Exon3 | splicing | c.266-2A>G |
| 1 | C2ORF40 | Exon3 | stopgain SNV | c.241C>T |
| 2 | C2ORF40 | Exon3 | stopgain SNV | c.256C>T |
| 1 | C2ORF42 | Exon7 | frameshift deletion | c.1182delA |
| 1 | C2ORF42 | Exon3 | frameshift deletion | c.681_693del  GACTCTGAAATCG |
| 1 | C2ORF42 | Exon3 | frameshift deletion | c.138_139delAT |
| 1 | C2ORF44 | Exon2 | frameshift deletion | c.1031delT |
| 1 | C2ORF44 | Exon2 | frameshift insertion | c.213_214insC |
| 1 | C2ORF56 | Exon8 | stopgain SNV | c.960T>A |
| 3 | C2ORF57 | Exon1 | frameshift deletion | c.428_431delGAGA |
| 5 | C2ORF63 | Exon10 | splicing | c.1018+2T>A |
| 4 | C2ORF63 | Exon7 | stopgain SNV | c.610C>T |
| 1 | C2ORF65 | Exon11 | frameshift deletion | c.1477_1484delCCCCTGCC |
| 4 | C2ORF65 | Exon5 | frameshift insertion | c.676dupT |
| 1 | C2ORF67 | Exon2 | frameshift insertion | c.238_239insAG |
| 1 | C2ORF68 | Exon3 | stopgain SNV | c.316C>T |
| 2 | C2ORF76 | Exon4 | frameshift deletion | c.134_143del |
| 1 | C2ORF77 | Exon7 | stopgain SNV | c.1030G>T |
| 1 | C2ORF78 | Exon3 | frameshift insertion | c.1993dupC |
| 1 | C2ORF80 | Exon5 | stopgain SNV | c.253C>T |
| 1 | C2ORF85 | Exon2 | frameshift deletion | c.1620delC |
| 1 | C3ORF20 | Exon3 | frameshift deletion | c.10delC |
| 18 | C3ORF25 | Exon5 | stopgain SNV | c.843T>G |
| 1 | C3ORF32 | Exon8 | stopgain SNV | c.604C>T |
| 1 | C3ORF32 | Exon6 | frameshift insertion | c.382dupG |
| 1 | C3ORF33 | Exon6 | frameshift deletion | c.664delT |
| 1 | C3ORF33 | Exon6 | stopgain SNV | c.394A>T |
| 1 | C3ORF62 | Exon3 | stopgain SNV | c.786dupT |
| 1 | C3ORF64 | Exon14 | frameshift deletion | c.1097delA |
| 1 | C3ORF67 | Exon13 | stopgain SNV | c.1231C>T |
| 10 | C3ORF72 | Exon2 | frameshift deletion | c.200_209del  ATTCGAAGGC |
| 1 | C3ORF77 | Exon2 | frameshift deletion | c.730delC |
| 1 | C4BPA | Exon7 | splicing | c.889+1G>A |
| 14 | C4ORF17 | Exon3 | frameshift deletion | c.253delT |
| 13 | C4ORF17 | Exon3 | frameshift insertion | c.255_256insG |
| 1 | C4ORF17 | Exon8 | splicing | c.880+2T>A |
| 1 | C4ORF21 | Exon12 | frameshift deletion | c.3562_3565delCAGA |
| 1 | C4ORF21 | Exon7 | splicing | c.2602+1G>A |
| 1 | C4ORF27 | Exon5 | frameshift deletion | c.516delA |
| 1 | C4ORF29 | Exon11 | stopgain SNV | c.1138C>T |
| 1 | C4ORF32 | Exon2 | frameshift deletion | c.248delT |
| 4 | C4ORF33 | Exon5 | stopgain SNV | c.430C>T |
| 2 | C4ORF37 | Exon10 | frameshift insertion | c.1282dupG |
| 1 | C4ORF37 | Exon4 | stopgain SNV | c.484C>T |
| 1 | C4ORF39 | Exon1 | frameshift deletion | c.307delA |
| 1 | C4ORF46 | Exon1 | frameshift deletion | c.2delT |
| 1 | C4ORF47 | Exon1 | frameshift deletion | c.98delA |
| 2 | C4ORF47 | Exon6 | frameshift deletion | c.775delG |
| 1 | C4ORF51 | Exon1 | frameshift insertion | c.165dupA |
| 1 | C4ORF6 | Exon1 | frameshift deletion | c.68delT |
| 1 | C4ORF6 | Exon1 | frameshift insertion | c.70dupC |
| 1 | C5 | Exon27 | frameshift deletion | c.3454delA |
| 3 | C5 | Exon25 | frameshift deletion | c.3215delG |
| 1 | C5ORF25 | Exon7 | stopgain SNV | c.920delT |
| 6 | C5ORF25 | Exon9 | stopgain SNV | c.1298T>A |
| 1 | C5ORF28 | Exon3 | stopgain SNV | c.331C>T |
| 1 | C5ORF34 | Exon3 | stopgain SNV | c.208C>T |
| 3 | C5ORF42 | Exon43 | stopgain SNV | c.8385T>G |
| 1 | C5ORF45 | Exon7 | stopgain SNV | c.808C>T |
| 1 | C5ORF45 | Exon7 | stopgain SNV | c.640G>T |
| 5 | C5ORF45 | Exon2 | stopgain SNV | c.112C>T |
| 1 | C5ORF48 | Exon3 | stoploss SNV | c.403T>G |
| 2 | C5ORF49 | Exon2 | stopgain SNV | c.214C>T |
| 1 | C5ORF54 | Exon2 | frameshift deletion | c.591_594delAAGA |
| 1 | C5ORF55 | Exon1 | frameshift deletion | c.196delC |
| 1 | C6 | Exon17 | splicing | c.2381+2T>C |
| 1 | C6 | Exon16 | splicing | c.2290+1G>T |
| 1 | C6 | Exon13 | frameshift deletion | c.1879delG |
| 1 | C6 | Exon13 | splicing | c.1856+2T>G |
| 1 | C6 | Exon12 | stopgain SNV | c.1786C>T |
| 1 | C6 | Exon12 | stopgain SNV | c.1751C>A |
| 2 | C6ORF106 | Exon3 | splicing | c.313+2T>C |
| 1 | C6ORF10 | Exon22 | frameshift deletion | c._del |
| 1 | C6ORF118 | Exon2 | frameshift deletion | c.384delG |
| 14 | C6ORF125 | Exon1 | frameshift deletion | c.10delA |
| 1 | C6ORF130 | Exon4 | stopgain SNV | c.226C>T |
| 1 | C6ORF146 | Exon7 | frameshift deletion | c.1291delG |
| 1 | C6ORF165 | Exon2 | stopgain SNV | c.43C>T |
| 1 | C6ORF165 | Exon4 | frameshift deletion | c.241_242delAT |
| 1 | C6ORF186 | Exon3 | stopgain SNV | c.483G>A |
| 1 | C6ORF186 | Exon4 | splicing | c.418-1G>A |
| 1 | C6ORF191 | Exon4 | frameshift deletion | c.228_231delTTGT |
| 1 | C6ORF1 | Exon5 | stopgain SNV | c.163G>T |
| 1 | C6ORF203 | Exon4 | frameshift insertion | c.647_648dupCT |
| 1 | C6ORF211 | Exon5 | frameshift deletion | c.727delG |
| 1 | C6ORF222 | Exon2 | frameshift insertion | c.571_572insC |
| 1 | C6ORF222 | Exon2 | frameshift deletion | c.568delG |
| 1 | C6ORF26 | Exon2 | stopgain SNV | c.226C>T |
| 1 | C6ORF70 | Exon13 | splicing | c.1317+1G>A |
| 1 | C7 | Exon6 | frameshift deletion | c.502delG |
| 1 | C7 | Exon17 | splicing | c.2350+1_2350+1delG |
| 2 | C7 | Exon17 | splicing | c.2350+2T>C |
| 2 | C7ORF10 | Exon15 | frameshift deletion | c.1194delG |
| 5 | C7ORF29 | Exon1 | frameshift deletion | c.666delC |
| 1 | C7ORF43 | Exon11 | splicing | c.1589+2T>A |
| 1 | C7ORF43 | Exon8 | frameshift insertion | c.1079dupT |
| 3 | C7ORF46 | Exon4 | frameshift deletion | c.633delG |
| 1 | C7ORF49 | Exon2 | frameshift deletion | c.12delA |
| 1 | C7ORF52 | Exon3 | frameshift deletion | c.408delC |
| 2 | C7ORF63 | Exon22 | stopgain SNV | c.2548C>T |
| 1 | C7ORF63 | Exon23 | frameshift insertion | c.2691dupA |
| 1 | C7ORF68 | Exon2 | frameshift insertion | c.168dupA |
| 1 | C7ORF72 | Exon1 | frameshift deletion | c.168delT |
| 1 | C8B | Exon12 | stopgain SNV | c.1653G>A |
| 4 | C8B | Exon9 | stopgain SNV | c.1282C>T |
| 6 | C8B | Exon6 | stopgain SNV | c.694A>T |
| 4 | C8ORF44 | Exon2 | frameshift deletion | c.82delG |
| 5 | C8ORF44 | Exon2 | stopgain SNV | c.207G>A |
| 2 | C8ORF44 | Exon2 | stopgain SNV | c.208C>T |
| 6 | C8ORF44-SGK3 | Exon17 | splicing | c.1075-2_1075-1delAG |
| 2 | C8ORF74 | Exon4 | frameshift insertion | c.844dupC |
| 2 | C8ORF80 | Exon19 | frameshift insertion | c.2376dupC |
| 1 | C8ORF85 | Exon2 | stopgain SNV | c.340C>T |
| 1 | C9 | Exon10 | frameshift deletion | c.1615delT |
| 1 | C9 | Exon4 | stopgain SNV | c.460C>T |
| 1 | C9ORF102 | Exon6 | frameshift deletion | c.1084_1087delGCCA |
| 1 | C9ORF117 | Exon9 | stoploss SNV | c.1561T>C |
| 1 | C9ORF128 | Exon2 | stopgain SNV | c.109G>T |
| 3 | C9ORF129 | Exon5 | stopgain SNV | c.508C>T |
| 1 | C9ORF131 | Exon3 | frameshift insertion | c.2015dupC |
| 1 | C9ORF131 | Exon3 | frameshift deletion | c.2673delA |
| 1 | C9ORF135 | Exon5 | stopgain SNV | c.468C>G |
| 14 | C9ORF152 | Exon1 | frameshift deletion | c.115delA |
| 1 | C9ORF153 | Exon3 | stopgain SNV | c.119C>A |
| 1 | C9ORF21 | Exon6 | splicing | c.422-2A>G |
| 1 | C9ORF25 | Exon6 | frameshift insertion | c.489dupC |
| 1 | C9ORF30-TMEFF1 | Exon1 | stopgain SNV | c.118C>T |
| 1 | C9ORF3 | Exon6 | frameshift deletion | c.1447delA |
| 1 | C9ORF3 | Exon11 | splicing | c.1744-1G>A |
| 1 | C9ORF3 | Exon12 | frameshift deletion | c.1845delT |
| 1 | C9ORF66 | Exon1 | stopgain SNV | c.57G>A |
| 1 | C9ORF71 | Exon2 | stoploss SNV | c.511T>C |
| 1 | C9ORF79 | Exon4 | frameshift deletion | c.884_894delGCCTGGGGTGC |
| 1 | C9ORF7 | Exon3 | frameshift deletion | c.287_288delCT |
| 1 | C9ORF86 | Exon7 | frameshift deletion | c.669_670delTT |
| 1 | C9ORF86 | Exon7 | frameshift deletion | c.674delA |
| 1 | C9ORF86 | Exon9 | frameshift insertion | c.826_827insGA |
| 1 | C9ORF86 | Exon9 | frameshift deletion | c.830delC |
| 1 | C9ORF86 | Exon9 | frameshift deletion | c.833delG |
| 1 | C9ORF91 | Exon2 | frameshift insertion | c.59dupC |
| 1 | C9ORF93 | Exon17 | frameshift deletion | c.2133delG |
| 4 | CA11 | Exon5 | splicing | c.471+1G>C |
| 1 | CA1 | Exon8 | splicing | c.451-2A>C |
| 1 | CA3 | Exon1 | frameshift insertion | c.17dupG |
| 1 | CA5A | Exon5 | stopgain SNV | c.580C>T |
| 1 | CA5B | Exon4 | splicing | c.459+1G>A |
| 1 | CA5B | Exon8 | frameshift deletion | c.951delC |
| 1 | CAB39L | Exon5 | frameshift insertion | c.245dupT |
| 1 | CABIN1 | Exon20 | frameshift deletion | c.2795delC |
| 2 | CABIN1 | Exon23 | frameshift deletion | c.3289delC |
| 2 | CABYR | Exon5 | frameshift deletion | c.664_665delTG |
| 2 | CACHD1 | Exon21 | splicing | c.2736-1G>A |
| 6 | CACNA1A | Exon46 | stopgain SNV | c.6619C>T |
| 1 | CACNA1B | Exon2 | splicing | c.285-2_285-2delA |
| 4 | CACNA1B | Exon6 | stopgain SNV | c.853G>T |
| 1 | CACNA1B | Exon46 | frameshift deletion | c.6928_6931delGTGC |
| 6 | CACNA1D | Exon28 | frameshift deletion | c.3435delC |
| 1 | CACNA1D | Exon46 | stopgain SNV | c.5661C>A |
| 1 | CACNA1E | Exon8 | stopgain SNV | c.1117C>T |
| 1 | CACNA1G | Exon12 | splicing | c.2754+1G>C |
| 2 | CACNA1G | Exon33 | frameshift deletion | c.5859delG |
| 1 | CACNA1G | Exon33 | frameshift deletion | c.5868_5869delTT |
| 2 | CACNA1S | Exon44 | frameshift deletion | c.5490_5497delAGAGCTAC |
| 1 | CACNA1S | Exon25 | frameshift deletion | c.3124delG |
| 1 | CACNA1S | Exon3 | splicing | c.258+2T>C |
| 1 | CACNA2D2 | Exon30 | frameshift deletion | c.2562delG |
| 1 | CACNA2D4 | Exon25 | stopgain SNV | c.2406C>A |
| 1 | CACNA2D4 | Exon19 | stopgain SNV | c.1882C>T |
| 1 | CACNB1 | Exon14 | frameshift insertion | c.1407_1408insT |
| 1 | CACNG6 | Exon3 | frameshift deletion | c.468_469delAG |
| 6 | CADM4 | Exon7 | splicing | c.755+2T>A |
| 2 | CAD | Exon14 | splicing | c.2156+2T>A |
| 1 | CAD | Exon32 | frameshift insertion | c.5105_5106insG |
| 1 | CAD | Exon40 | frameshift deletion | c.6098delT |
| 2 | CADPS | Exon20 | stopgain SNV | c.2810T>G |
| 1 | CADPS | Exon3 | stopgain SNV | c.710G>A |
| 1 | CAGE1 | Exon4 | stopgain SNV | c.469A>T |
| 1 | CALCR | Exon14 | frameshift deletion | c.1358delA |
| 4 | CALHM2 | Exon3 | frameshift deletion | c.341_342delCT |
| 2 | CALM1 | Exon2 | splicing | c.4-1G>C |
| 12 | CALML4 | Exon1 | stopgain SNV | c.118C>T |
| 2 | CALML5 | Exon1 | frameshift deletion | c.219delG |
| 1 | CAMK1 | Exon5 | frameshift deletion | c.293delT |
| 1 | CAMK2A | Exon17 | frameshift deletion | c.1207delT |
| 1 | CAMK2A | Exon18 | splicing | c.1205-1_1205-1delG |
| 1 | CAMK2B | Exon3 | frameshift deletion | c.199delC |
| 1 | CAMKV | Exon7 | frameshift deletion | c.609delC |
| 1 | CAMLG | Exon4 | stoploss SNV | c.889T>C |
| 1 | CAMSAP1 | Exon17 | splicing | c.4506+2C>T |
| 2 | CAMTA2 | Exon15 | frameshift insertion | c.2603dupC |
| 1 | CAMTA2 | Exon4 | stopgain SNV | c.279G>A |
| 2 | CANT1 | Exon4 | frameshift deletion | c.708delT |
| 1 | CANX | Exon3 | stopgain SNV | c.242C>G |
| 3 | CAPG | Exon4 | frameshift deletion | c.332delC |
| 1 | CAPN11 | Exon11 | stopgain SNV | c.1196G>A |
| 1 | CAPN11 | Exon13 | splicing | c.1417-1G>A |
| 1 | CAPN14 | Exon2 | stopgain SNV | c.32G>A |
| 3 | CAPN2 | Exon3 | stopgain SNV | c.84G>A |
| 1 | CAPN5 | Exon2 | frameshift insertion | c.55_56insC |
| 2 | CAPN6 | Exon7 | stopgain SNV | c.931A>T |
| 5 | CAPN7 | Exon12 | frameshift deletion | c.1331delT |
| 2 | CAPN9 | Exon3 | splicing | c.284-1G>C |
| 2 | CAPN9 | Exon4 | splicing | c.403-2A>G |
| 1 | CAPN9 | Exon13 | splicing | c.1579+2T>G |
| 1 | CAPRIN2 | Exon1 | frameshift deletion | c.234_237delAGAG |
| 6 | CAPS2 | Exon13 | stopgain SNV | c.1196_1203dupATCAAATA |
| 1 | CAPS2 | Exon13 | stopgain SNV | c.1180C>T |
| 1 | CAPS2 | Exon11 | splicing | c.911+2T>A |
| 1 | CAPSL | Exon5 | frameshift deletion | c.553delT |
| 11 | CAPSL | Exon5 | frameshift deletion | c.536delA |
| 1 | CARD10 | Exon14 | stopgain SNV | c.2197C>T |
| 1 | CARD14 | Exon5 | frameshift deletion | c.832delA |
| 3 | CARD17 | Exon3 | splicing | c.8-1G>C |
| 1 | CARM1 | Exon7 | frameshift deletion | c.848_849delGA |
| 3 | CARS2 | Exon15 | frameshift insertion | c.1683dupA |
| 2 | CARS2 | Exon12 | stopgain SNV | c.1207A>T |
| 5 | CARTPT | Exon3 | stoploss SNV | c.349T>C |
| 1 | CASKIN2 | Exon11 | frameshift deletion | c.1060delG |
| 1 | CASKIN2 | Exon11 | frameshift insertion | c.1052_1053insG |
| 1 | CASP10 | Exon2 | stopgain SNV | c.325C>T |
| 1 | CASP14 | Exon7 | stoploss SNV | c.727T>G |
| 1 | CASP4 | Exon3 | frameshift deletion | c.360_363delAAGA |
| 1 | CASP4 | Exon2 | frameshift deletion | c.231delT |
| 2 | CASP5 | Exon3 | frameshift deletion | c.241delA |
| 4 | CASP5 | Exon3 | frameshift insertion | c.241dupA |
| 19 | CASP5 | Exon2 | frameshift deletion | c.67delA |
| 1 | CASP8AP2 | Exon8 | frameshift deletion | c.3550delG |
| 1 | CASP8 | Exon8 | splicing | c.661-1G>A |
| 7 | CASP9 | Exon6 | stopgain SNV | c.532G>T |
| 1 | CASP9 | Exon3 | frameshift deletion | c.178delG |
| 1 | CASR | Exon4 | frameshift deletion | c.1103delT |
| 1 | CASR | Exon7 | frameshift deletion | c.2244delG |
| 1 | CATSPER1 | Exon10 | splicing | c.2065-2A>G |
| 1 | CATSPER2 | Exon8 | splicing | c.842+1G>A |
| 1 | CATSPER2 | Exon2 | stopgain SNV | c.40C>T |
| 1 | CATSPERG | Exon10 | stopgain SNV | c.1168C>T |
| 1 | CATSPERG | Exon12 | frameshift insertion | c.1354_1355insC |
| 1 | CAV3 | Exon2 | frameshift insertion | c.197_198dupCT |
| 1 | CBFA2T2 | Exon11 | stopgain SNV | c.1551C>A |
| 1 | CBFA2T2 | Exon11 | stopgain SNV | c.1552_1553insA |
| 7 | CBLC | Exon8 | frameshift insertion | c.1255dupC |
| 3 | CBX3 | Exon4 | splicing | c.168-1_168-0insA |
| 1 | CBX4 | Exon5 | frameshift insertion | c.690dupC |
| 1 | CBX8 | Exon5 | frameshift insertion | c.956dupG |
| 1 | CBX8 | Exon3 | frameshift deletion | c.114_114del |
| 1 | CBY1 | Exon5 | frameshift insertion | c.378dupA |
| 1 | CC2D1B | Exon7 | frameshift deletion | c.620_623delTAGC |
| 1 | CC2D1B | Exon2 | frameshift deletion | c.45_46delCC |
| 1 | CC2D2B | Exon9 | stopgain SNV | c.958C>T |
| 1 | CCBL2 | Exon14 | frameshift deletion | c.1305delA |
| 2 | CCBP2 | Exon3 | frameshift deletion | c.895_896delAG |
| 1 | CCDC102B | Exon6 | stopgain SNV | c.890G>A |
| 1 | CCDC105 | Exon1 | frameshift deletion | c.56_57delCC |
| 4 | CCDC105 | Exon6 | frameshift deletion | c.1204delC |
| 1 | CCDC108 | Exon26 | frameshift deletion | c.4211_4212delGC |
| 1 | CCDC108 | Exon26 | frameshift deletion | c.4190delT |
| 1 | CCDC108 | Exon24 | frameshift deletion | c.3893_3894delAC |
| 1 | CCDC108 | Exon24 | frameshift deletion | c.3882delG |
| 1 | CCDC108 | Exon15 | stopgain SNV | c.2543G>A |
| 3 | CCDC111 | Exon7 | frameshift deletion | c.558delT |
| 1 | CCDC11 | Exon2 | stopgain SNV | c.292C>T |
| 7 | CCDC121 | Exon2 | frameshift deletion | c.737_738delGA |
| 1 | CCDC122 | Exon7 | stopgain SNV | c.745C>T |
| 1 | CCDC127 | Exon3 | stopgain SNV | c.367G>T |
| 2 | CCDC129 | Exon11 | frameshift deletion | c.2277delT |
| 4 | CCDC132 | Exon18 | splicing | c.1453-1G>C |
| 1 | CCDC135 | Exon15 | frameshift deletion | c.2175_2176delCA |
| 1 | CCDC13 | Exon13 | frameshift deletion | c.1620delA |
| 1 | CCDC144A | Exon13 | stopgain SNV | c.3409C>T |
| 1 | CCDC144NL | Exon4 | splicing | c.416-1G>A |
| 1 | CCDC146 | Exon15 | frameshift insertion | c.2058dupT |
| 1 | CCDC147 | Exon8 | splicing | c.1091-2A>C |
| 1 | CCDC148 | Exon12 | frameshift deletion | c.1458_1459delAG |
| 2 | CCDC148 | Exon5 | splicing | c.252-1G>C |
| 1 | CCDC14 | Exon7 | stopgain SNV | c.952C>T |
| 13 | CCDC150 | Exon3 | frameshift deletion | c.291_292delTG |
| 1 | CCDC150 | Exon3 | stopgain SNV | c.307C>T |
| 2 | CCDC150 | Exon7 | frameshift deletion | c.849delA |
| 1 | CCDC153 | Exon6 | stopgain SNV | c.403G>T |
| 2 | CCDC27 | Exon12 | frameshift deletion | c.1916delT |
| 1 | CCDC30 | Exon4 | stopgain SNV | c.418G>T |
| 1 | CCDC37 | Exon9 | frameshift insertion | c.891_892insG |
| 1 | CCDC37 | Exon15 | frameshift deletion | c.1617_1620delGCGG |
| 1 | CCDC38 | Exon2 | stopgain SNV | c.14T>G |
| 4 | CCDC39 | Exon19 | frameshift insertion | c.2660dupT |
| 1 | CCDC40 | Exon3 | frameshift insertion | c.310dupG |
| 1 | CCDC40 | Exon13 | frameshift insertion | c.2205dupG |
| 1 | CCDC51 | Exon4 | stopgain SNV | c.961C>T |
| 1 | CCDC57 | Exon12 | frameshift deletion | c.1772delA |
| 1 | CCDC59 | Exon2 | frameshift deletion | c.196delA |
| 1 | CCDC60 | Exon3 | frameshift deletion | c.333delC |
| 1 | CCDC62 | Exon8 | stopgain SNV | c.931C>T |
| 1 | CCDC63 | Exon5 | frameshift deletion | c.391delA |
| 1 | CCDC65 | Exon3 | splicing | c.301-2A>C |
| 1 | CCDC65 | Exon3 | splicing | c.301-2A>G |
| 16 | CCDC65 | Exon3 | splicing | c.301-1G>A |
| 1 | CCDC65 | Exon3 | stopgain SNV | c.439G>T |
| 1 | CCDC65 | Exon5 | frameshift insertion | c.649_650insC |
| 1 | CCDC65 | Exon6 | frameshift deletion | c.876_877delTA |
| 1 | CCDC65 | Exon7 | stopgain SNV | c.1101T>G |
| 3 | CCDC66 | Exon10 | stopgain SNV | c.1381C>T |
| 4 | CCDC66 | Exon14 | frameshift insertion | c.2319dupG |
| 1 | CCDC66 | Exon17 | frameshift deletion | c.2649delA |
| 3 | CCDC66 | Exon18 | frameshift insertion | c.2823_2826dupAGAG |
| 1 | CCDC67 | Exon12 | frameshift deletion | c.1510delC |
| 1 | CCDC73 | Exon16 | stopgain SNV | c.1642G>T |
| 2 | CCDC77 | Exon4 | stopgain SNV | c.390T>G |
| 1 | CCDC77 | Exon6 | frameshift deletion | c.491delC |
| 1 | CCDC77 | Exon6 | frameshift deletion | c.496_497delAA |
| 1 | CCDC78 | Exon5 | stopgain SNV | c.472C>T |
| 17 | CCDC7 | Exon2 | frameshift insertion | c.230dupT |
| 1 | CCDC7 | Exon9 | stopgain SNV | c.796C>T |
| 1 | CCDC7 | Exon15 | frameshift insertion | c.1185dupA |
| 1 | CCDC81 | Exon9 | stopgain SNV | c.994C>T |
| 1 | CCDC83 | Exon12 | splicing | c.1174-1G>A |
| 1 | CCDC84 | Exon9 | splicing | c.805+2dupT |
| 1 | CCDC85A | Exon2 | frameshift deletion | c.734delG |
| 1 | CCDC85A | Exon2 | frameshift deletion | c.742delC |
| 1 | CCDC86 | Exon1 | frameshift deletion | c.568delC |
| 1 | CCDC89 | Exon1 | frameshift deletion | c.773_774delGC |
| 1 | CCDC89 | Exon1 | stopgain SNV | c.234C>A |
| 1 | CCDC8 | Exon1 | frameshift deletion | c.1252delA |
| 1 | CCDC90A | Exon3 | frameshift deletion | c.619_620delAT |
| 1 | CCDC94 | Exon4 | splicing | c.271-1G>T |
| 1 | CCDC96 | Exon1 | frameshift insertion | c.1450_1451insTT |
| 1 | CCDC97 | Exon2 | frameshift insertion | c.393dupT |
| 1 | CCDC99 | Exon11 | frameshift deletion | c.1426delG |
| 2 | CCDC9 | Exon12 | stopgain SNV | c.1578G>A |
| 1 | CCHCR1 | Exon16 | frameshift insertion | c.1954_1955insT |
| 4 | CCHCR1 | Exon15 | splicing | c.1610-2dupA |
| 2 | CCKBR | Exon5 | frameshift insertion | c.1029dupT |
| 8 | CCL25 | Exon6 | stoploss SNV | c.452G>C |
| 5 | CCL26 | Exon3 | stopgain SNV | c.132G>A |
| 1 | CCNB1IP1 | Exon5 | frameshift deletion | c.467delA |
| 1 | CCND2 | Exon1 | frameshift deletion | c.83delA |
| 1 | CCNDBP1 | Exon8 | frameshift insertion | c.746_747dupGA |
| 1 | CCNF | Exon8 | frameshift deletion | c.756_757delCC |
| 1 | CCNI2 | Exon5 | frameshift deletion | c.895delC |
| 1 | CCR1 | Exon2 | frameshift deletion | c.407delT |
| 1 | CCR8 | Exon2 | frameshift deletion | c.67delA |
| 1 | CCR9 | Exon3 | frameshift deletion | c.65delC |
| 1 | CCRN4L | Exon3 | stopgain SNV | c.1030delC |
| 3 | CCT5 | Exon4 | stopgain SNV | c.412G>T |
| 2 | CCT6B | Exon13 | stoploss SNV | c.1457G>T |
| 1 | CCT6B | Exon6 | splicing | c.480-2A>G |
| 5 | CCT8L2 | Exon1 | stopgain SNV | c.1094G>A |
| 1 | CD101 | Exon2 | stopgain SNV | c.352G>T |
| 1 | CD109 | Exon26 | stopgain SNV | c.3149C>G |
| 1 | CD109 | Exon27 | frameshift deletion | c.3376delA |
| 1 | CD109 | Exon32 | frameshift deletion | c.3949delA |
| 6 | CD163L1 | Exon8 | frameshift deletion | c.2007delT |
| 1 | CD164 | Exon7 | splicing | c.428-1G>C |
| 1 | CD177 | Exon4 | splicing | c.380-1G>C |
| 1 | CD177 | Exon4 | stopgain SNV | c.392T>A |
| 1 | CD177 | Exon5 | frameshift deletion | c.598_599delAC |
| 2 | CD177 | Exon7 | frameshift deletion | c.786_787delCT |
| 1 | CD180 | Exon3 | frameshift deletion | c.796delG |
| 1 | CD19 | Exon1 | frameshift deletion | c.14_21delGCCTCCTC |
| 1 | CD1A | Exon2 | stopgain SNV | c.93G>A |
| 1 | CD1A | Exon4 | stopgain SNV | c.745C>T |
| 1 | CD1B | Exon3 | stopgain SNV | c.561T>A |
| 11 | CD200R1L | Exon5 | frameshift deletion | c.546delC |
| 2 | CD200R1 | Exon4 | frameshift insertion | c.432_433insA |
| 1 | CD207 | Exon5 | stopgain SNV | c.791G>A |
| 1 | CD207 | Exon1 | stopgain SNV | c.70C>T |
| 1 | CD209 | Exon4 | frameshift deletion | c.387delG |
| 3 | CD22 | Exon4 | splicing | c.413-1G>A |
| 2 | CD244 | Exon4 | stopgain SNV | c.764C>G |
| 1 | CD247 | Exon6 | frameshift deletion | c.355_370del  GCGGAGGCCTACAGTG |
| 4 | CD27 | Exon4 | frameshift deletion | c.497delT |
| 1 | CD27 | Exon4 | frameshift deletion | c.514_515delCC |
| 1 | CD300LB | Exon2 | stopgain SNV | c.262C>T |
| 4 | CD300LG | Exon2 | frameshift deletion | c.345delT |
| 2 | CD300LG | Exon4 | frameshift insertion | c.651dupC |
| 1 | CD320 | Exon2 | frameshift deletion | c.301delG |
| 16 | CD33 | Exon3 | frameshift deletion | c.466_469delGGCC |
| 1 | CD36 | Exon4 | stopgain SNV | c.220C>T |
| 1 | CD36 | Exon5 | frameshift insertion | c.378_381dupATCA |
| 4 | CD36 | Exon7 | splicing | c.701+1_701+4dupGTAA |
| 6 | CD36 | Exon10 | stopgain SNV | c.975T>G |
| 3 | CD36 | Exon11 | stopgain SNV | c.1079T>G |
| 1 | CD36 | Exon13 | frameshift deletion | c.1202_1205delTATT |
| 1 | CD36 | Exon14 | frameshift insertion | c.1379_1382dupTGAT |
| 1 | CD38 | Exon1 | frameshift deletion | c.109delG |
| 1 | CD3EAP | Exon3 | frameshift deletion | c.1246delG |
| 3 | CD5L | Exon3 | stopgain SNV | c.328G>T |
| 1 | CD5L | Exon3 | frameshift insertion | c.105_106insA |
| 1 | CD5 | Exon5 | frameshift deletion | c.558delC |
| 1 | CD5 | Exon5 | frameshift insertion | c.564_565insT |
| 1 | CD5 | Exon6 | frameshift insertion | c.988_989insA |
| 1 | CD63 | Exon3 | frameshift insertion | c.110_111insA |
| 1 | CD68 | Exon5 | frameshift insertion | c.923_924insC |
| 1 | CD6 | Exon10 | frameshift insertion | c.1575_1576insGAGA |
| 1 | CD72 | Exon8 | frameshift insertion | c.983_984insGG |
| 1 | CD72 | Exon8 | frameshift insertion | c.982_983insAGCTTTGGTCATGG |
| 4 | CD72 | Exon7 | stopgain SNV | c.851T>G |
| 1 | CD72 | Exon6 | stopgain SNV | c.724A>T |
| 1 | CD79A | Exon2 | frameshift insertion | c.193_194insAA |
| 1 | CD97 | Exon11 | frameshift deletion | c.1252delG |
| 1 | CDAN1 | Exon5 | frameshift deletion | c.969_970delGG |
| 1 | CDAN1 | Exon5 | frameshift deletion | c.962delT |
| 1 | CDAN1 | Exon5 | frameshift deletion | c.953delT |
| 1 | CDC20B | Exon8 | frameshift deletion | c.898delT |
| 4 | CDC27 | Exon13 | frameshift insertion | c.1702_1703insCC |
| 1 | CDC27 | Exon13 | frameshift insertion | c.1699_1702dupCCAG |
| 4 | CDC27 | Exon5 | stopgain SNV | c.431T>G |
| 1 | CDC27 | Exon4 | frameshift deletion | c.271delA |
| 3 | CDC27 | Exon2 | frameshift insertion | c.59dupC |
| 1 | CDC34 | Exon5 | stopgain SNV | c.682G>T |
| 1 | CDC42BPB | Exon29 | frameshift insertion | c.3797_3798dupAG |
| 1 | CDC42BPB | Exon28 | frameshift insertion | c.3553_3554insAG |
| 1 | CDC42BPB | Exon28 | frameshift insertion | c.3543_3544insA |
| 1 | CDC42BPB | Exon26 | frameshift insertion | c.3398_3399insCC |
| 1 | CDC42BPB | Exon15 | frameshift insertion | c.2149_2150insG |
| 1 | CDC42BPG | Exon37 | frameshift deletion | c.4628delT |
| 1 | CDC42EP1 | Exon2 | frameshift insertion | c.34_35insG |
| 1 | CDCA2 | Exon15 | frameshift insertion | c.2979dupA |
| 1 | CDCA3 | Exon5 | frameshift deletion | c.607delA |
| 1 | CDCA3 | Exon3 | frameshift insertion | c.138dupG |
| 4 | CDCP2 | Exon4 | frameshift insertion | c.1224_1225insGC |
| 2 | CDCP2 | Exon4 | frameshift deletion | c.1224delC |
| 14 | CDCP2 | Exon4 | frameshift insertion | c.1223_1224dupCC |
| 1 | CDCP2 | Exon1 | frameshift deletion | c.50_51delGC |
| 1 | CDH10 | Exon11 | stopgain SNV | c.1657G>T |
| 11 | CDH24 | Exon2 | stopgain SNV | c.138G>A |
| 1 | CDH26 | Exon4 | splicing | c.232-2A>C |
| 1 | CDH26 | Exon13 | stopgain SNV | c.1909C>T |
| 1 | CDH2 | Exon13 | frameshift deletion | c.2175delC |
| 1 | CDH3 | Exon16 | frameshift insertion | c.2462dupA |
| 8 | CDH5 | Exon9 | splicing | c.1485+2T>A |
| 1 | CDH7 | Exon12 | frameshift deletion | c.2087delC |
| 1 | CDHR3 | Exon9 | frameshift deletion | c.1154delT |
| 1 | CDHR3 | Exon10 | frameshift insertion | c.1316dupC |
| 1 | CDHR4 | Exon11 | frameshift deletion | c.1456delA |
| 1 | CDHR5 | Exon11 | frameshift deletion | c.1184delT |
| 1 | CDK10 | Exon11 | frameshift deletion | c.581_582delGC |
| 2 | CDK11A | Exon16 | frameshift insertion | c.1735dupT |
| 1 | CDK11A | Exon5 | stopgain SNV | c.415C>T |
| 1 | CDK12 | Exon9 | splicing | c.2846+1G>A |
| 1 | CDK13 | Exon14 | stopgain SNV | c.3886G>T |
| 1 | CDK15 | Exon5 | splicing | c.390+2T>C |
| 1 | CDK19 | Exon3 | splicing | c.129-1G>C |
| 3 | CDK2AP2 | Exon4 | frameshift deletion | c.345delG |
| 3 | CDK2AP2 | Exon4 | frameshift deletion | c.343delG |
| 1 | CDK2AP2 | Exon4 | frameshift deletion | c.325_331delGCCCGGG |
| 1 | CDK5RAP1 | Exon5 | stopgain SNV | c.457C>T |
| 1 | CDK5RAP2 | Exon31 | frameshift deletion | c.4670_4671delTC |
| 9 | CDK5RAP2 | Exon30 | stopgain SNV | c.4584delC |
| 1 | CDK5RAP2 | Exon30 | frameshift deletion | c.4576delG |
| 1 | CDK5RAP2 | Exon27 | stopgain SNV | c.4096G>T |
| 1 | CDK5RAP3 | Exon8 | frameshift deletion | c.726_727delGA |
| 5 | CDKL2 | Exon11 | splicing | c.1323-1G>A |
| 3 | CDKL3 | Exon11 | frameshift insertion | c.1512_1513dupGG |
| 1 | CDKN2B | Exon1 | frameshift deletion | c.120delC |
| 1 | CDKN3 | Exon5 | frameshift deletion | c.372delA |
| 2 | CDON | Exon10 | stopgain SNV | c.1925T>G |
| 1 | CDR2 | Exon3 | frameshift deletion | c.204delG |
| 2 | CDRT15 | Exon2 | splicing | c.262+1G>A |
| 2 | CDRT15 | Exon1 | stopgain SNV | c.70C>T |
| 1 | CDRT1 | Exon6 | frameshift deletion | c.1224_1227delCTCT |
| 1 | CDRT1 | Exon1 | frameshift deletion | c.152_158delTATCACA |
| 1 | CDRT4 | Exon4 | frameshift insertion | c.432_433insC |
| 1 | CDX2 | Exon3 | splicing | c.687+2T>G |
| 1 | CDX2 | Exon2 | frameshift deletion | c.680_683delAGAG |
| 1 | CEACAM19 | Exon2 | frameshift deletion | c.386_389delAATG |
| 1 | CEACAM1 | Exon4 | frameshift deletion | c.767delG |
| 1 | CEACAM1 | Exon3 | frameshift insertion | c.553_554insAGGC |
| 1 | CEACAM1 | Exon2 | frameshift deletion | c.207delA |
| 1 | CEACAM20 | Exon11 | frameshift deletion | c.1623delC |
| 1 | CEACAM20 | Exon5 | stopgain SNV | c.908C>G |
| 1 | CEACAM4 | Exon2 | splicing | c.64+1G>C |
| 2 | CEACAM7 | Exon2 | stopgain SNV | c.250A>T |
| 1 | CEACAM7 | Exon2 | splicing | c.64+1G>T |
| 1 | CECR1 | Exon7 | splicing | c.973-2A>G |
| 1 | CECR2 | Exon13 | frameshift deletion | c.1625delC |
| 2 | CECR2 | Exon16 | stopgain SNV | c.3666G>A |
| 2 | CECR2 | Exon16 | stopgain SNV | c.4210C>T |
| 1 | CELA1 | Exon6 | frameshift insertion | c.590dupG |
| 7 | CELA1 | Exon1 | frameshift insertion | c.12_13insCAACACC |
| 2 | CELA1 | Exon1 | frameshift deletion | c.8delT |
| 20 | CELA1 | Exon1 | frameshift insertion | c.6_7insC |
| 2 | CELA3B | Exon5 | frameshift deletion | c.439delG |
| 1 | CELF3 | Exon11 | frameshift insertion | c.1133dupA |
| 1 | CELSR3 | Exon21 | frameshift deletion | c.7229delT |
| 1 | CELSR3 | Exon21 | frameshift deletion | c.7224delC |
| 1 | CELSR3 | Exon1 | frameshift deletion | c.3058delC |
| 3 | CENPBD1 | Exon1 | stopgain SNV | c.102dupT |
| 4 | CENPE | Exon33 | frameshift deletion | c.4898delT |
| 1 | CENPE | Exon16 | splicing | c.1391-2A>G |
| 1 | CENPJ | Exon7 | stopgain SNV | c.1586C>G |
| 1 | CEP128 | Exon3 | frameshift deletion | c.221delG |
| 1 | CEP135 | Exon21 | frameshift insertion | c.2743_2744dupGA |
| 1 | CEP152 | Exon26 | frameshift deletion | c.4210_4211delGT |
| 2 | CEP164 | Exon5 | frameshift deletion | c.347delA |
| 1 | CEP192 | Exon19 | splicing | c.3190-1G>C |
| 1 | CEP192 | Exon30 | splicing | c.5616+1G>C |
| 3 | CEP250 | Exon17 | frameshift deletion | c.1943delC |
| 1 | CEP250 | Exon27 | frameshift insertion | c.3681dupT |
| 1 | CEP290 | Exon49 | splicing | c.6645+1G>A |
| 1 | CEP290 | Exon42 | stopgain SNV | c.5824C>T |
| 1 | CEP290 | Exon39 | frameshift deletion | c.5267delC |
| 1 | CEP290 | Exon39 | stopgain SNV | c.5263delA |
| 13 | CEP290 | Exon27 | splicing | c.2818-1G>T |
| 1 | CEP44 | Exon7 | frameshift deletion | c.589delG |
| 1 | CEP55 | Exon3 | stopgain SNV | c.256C>T |
| 1 | CEP57L1 | Exon10 | frameshift deletion | c.810delT |
| 1 | CEP68 | Exon3 | stopgain SNV | c.925delC |
| 1 | CEP68 | Exon3 | frameshift deletion | c.942delG |
| 1 | CEP70 | Exon16 | stopgain SNV | c.1648C>T |
| 1 | CEP78 | Exon15 | splicing | c.1801-1G>C |
| 4 | CEP85 | Exon14 | stopgain SNV | c.2243C>G |
| 1 | CEP89 | Exon19 | stopgain SNV | c.2200G>T |
| 1 | CEP89 | Exon15 | stopgain SNV | c.1714C>T |
| 1 | CEP95 | Exon10 | frameshift insertion | c.1095dupA |
| 1 | CER1 | Exon1 | stopgain SNV | c.19C>T |
| 1 | CERCAM | Exon12 | frameshift insertion | c.1632_1633insATCT |
| 1 | CERKL | Exon5 | stopgain SNV | c.715C>T |
| 1 | CERS5 | Exon6 | splicing | c.543+2T>C |
| 3 | CES1 | Exon8 | splicing | c.903+1G>T |
| 1 | CES2 | Exon2 | frameshift deletion | c.382delG |
| 2 | CES3 | Exon8 | stopgain SNV | c.1050G>A |
| 1 | CES3 | Exon10 | stopgain SNV | c.1288C>T |
| 7 | CES5A | Exon5 | stopgain SNV | c.607C>T |
| 2 | CES5A | Exon5 | frameshift deletion | c.568_571delGCCC |
| 3 | CETN3 | Exon2 | frameshift deletion | c.43_44delAA |
| 1 | CFDP1 | Exon5 | splicing | c.403-1G>C |
| 1 | CFHR1 | Exon1 | stopgain SNV | c.19delG |
| 1 | CFHR1 | Exon5 | splicing | c.790+1G>A |
| 1 | CFHR2 | Exon1 | stopgain SNV | c.19delG |
| 5 | CFHR2 | Exon4 | stopgain SNV | c.595G>T |
| 1 | CFHR3 | Exon4 | splicing | c.613+2T>C |
| 1 | CFHR3 | Exon6 | frameshift deletion | c.839_840delTA |
| 1 | CFHR4 | Exon3 | splicing | c.439+2T>C |
| 3 | CFHR5 | Exon4 | frameshift insertion | c.485_486dupAA |
| 3 | CFHR5 | Exon4 | frameshift insertion | c.486dupA |
| 1 | CFHR5 | Exon8 | frameshift deletion | c.1225delG |
| 1 | CFHR5 | Exon10 | stopgain SNV | c.1704T>A |
| 1 | CFTR | Exon23 | stopgain SNV | c.3846G>A |
| 1 | CGB5 | Exon3 | stopgain SNV | c.473C>A |
| 1 | CGN | Exon14 | stopgain SNV | c.2704G>T |
| 1 | CHAC2 | Exon1 | frameshift deletion | c.116delA |
| 1 | CHAF1A | Exon13 | frameshift insertion | c.2212dupC |
| 1 | CHAF1B | Exon13 | splicing | c.1494-2_1494-2delA |
| 1 | CHCHD4 | Exon3 | frameshift deletion | c.351_352delGA |
| 1 | CHCHD4 | Exon2 | stopgain SNV | c.31C>T |
| 1 | CHCHD6 | Exon4 | frameshift deletion | c.344delA |
| 1 | CHD1L | Exon8 | stopgain SNV | c.781C>T |
| 2 | CHD1L | Exon14 | splicing | c.1386-2A>G |
| 1 | CHD2 | Exon22 | frameshift deletion | c._del |
| 1 | CHD2 | Exon22 | frameshift insertion | c.2741_2742insA |
| 1 | CHD3 | Exon38 | frameshift deletion | c.5769delT |
| 1 | CHD3 | Exon38 | stopgain SNV | c.5770delC |
| 1 | CHD5 | Exon9 | frameshift insertion | c.1284_1285insA |
| 4 | CHD6 | Exon18 | stopgain SNV | c.2739C>A |
| 1 | CHD9 | Exon31 | frameshift deletion | c.6531delA |
| 1 | CHD9 | Exon31 | frameshift insertion | c.6538_6539insA |
| 2 | CHEK2 | Exon11 | frameshift deletion | c.1100delC |
| 1 | CHERP | Exon17 | frameshift deletion | c.2740delG |
| 1 | CHERP | Exon17 | frameshift deletion | c.2729delA |
| 1 | CHI3L1 | Exon8 | stopgain SNV | c.865A>T |
| 1 | CHI3L1 | Exon7 | stopgain SNV | c.670C>T |
| 1 | CHI3L2 | Exon2 | frameshift deletion | c.240delC |
| 1 | CHIC2 | Exon1 | stopgain SNV | c.24T>G |
| 2 | CHIT1 | Exon10 | frameshift deletion | c._del |
| 1 | CHIT1 | Exon10 | stopgain SNV | c.1125C>A |
| 1 | CHIT1 | Exon5 | splicing | c.314+1G>T |
| 1 | CHL1 | Exon15 | frameshift deletion | c.1683_1684delAA |
| 1 | CHP2 | Exon4 | frameshift deletion | c.275delC |
| 1 | CHP2 | Exon5 | frameshift deletion | c.387delG |
| 3 | CHP2 | Exon7 | stopgain SNV | c.562C>T |
| 1 | CHPF2 | Exon4 | frameshift deletion | c.1330_1331delTA |
| 1 | CHPF | Exon4 | stopgain SNV | c.1111C>T |
| 1 | CHRFAM7A | Exon9 | frameshift insertion | c.948_949insA |
| 1 | CHRFAM7A | Exon9 | frameshift insertion | c.947_948insA |
| 1 | CHRM1 | Exon2 | frameshift deletion | c.1148delT |
| 1 | CHRNA1 | Exon6 | frameshift deletion | c.524delT |
| 1 | CHRNA1 | Exon5 | splicing | c.309+1G>T |
| 1 | CHRNA2 | Exon6 | frameshift deletion | c.1293delC |
| 1 | CHRNA4 | Exon4 | frameshift insertion | c.307_308insATAG |
| 1 | CHRNA6 | Exon4 | frameshift deletion | c.433delA |
| 1 | CHRNA7 | Exon10 | frameshift insertion | c.1389_1390insG |
| 1 | CHRNA7 | Exon10 | frameshift insertion | c.1577_1578insA |
| 1 | CHRNA7 | Exon10 | frameshift insertion | c.1578_1579insA |
| 1 | CHRNB3 | Exon1 | splicing | c.52+1G>A |
| 1 | CHRNB3 | Exon5 | frameshift deletion | c.515delG |
| 1 | CHRNG | Exon5 | splicing | c.351-2A>G |
| 1 | CHST15 | Exon2 | frameshift deletion | c.9delC |
| 1 | CHST5 | Exon3 | frameshift deletion | c.361_364delATCT |
| 1 | CHTOP | Exon6 | frameshift deletion | c.581delG |
| 1 | CIAO1 | Exon5 | splicing | c.691+2dupT |
| 1 | CIAO1 | Exon7 | frameshift deletion | c.792_793delAG |
| 1 | CIB4 | Exon1 | stopgain SNV | c.21T>A |
| 1 | CIDEA | Exon5 | frameshift insertion | c.590_591insC |
| 1 | CIDEB | Exon7 | splicing | c.337-2A>G |
| 1 | CIDEB | Exon6 | splicing | c.336+1G>A |
| 1 | CKAP5 | Exon3 | frameshift deletion | c.171delC |
| 1 | CKB | Exon8 | frameshift deletion | c.1068delA |
| 1 | CKB | Exon8 | frameshift deletion | c.1065_1066delCG |
| 1 | CKB | Exon8 | frameshift deletion | c.1054_1055delAT |
| 1 | CKM | Exon8 | stoploss SNV | c.1144T>C |
| 1 | CLASP1 | Exon34 | frameshift insertion | c.3692dupG |
| 2 | CLASP1 | Exon32 | stopgain SNV | c.3380T>G |
| 1 | CLCA2 | Exon1 | stopgain SNV | c.148C>T |
| 2 | CLCA2 | Exon1 | frameshift deletion | c.162delT |
| 1 | CLCA2 | Exon7 | frameshift deletion | c.993delA |
| 2 | CLCA4 | Exon6 | frameshift insertion | c.760dupA |
| 1 | CLCN1 | Exon12 | splicing | c.1401+1G>T |
| 1 | CLCN2 | Exon15 | frameshift deletion | c.1607_1608delAT |
| 1 | CLCN3 | Exon8 | frameshift deletion | c.990delT |
| 3 | CLCN3 | Exon12 | frameshift deletion | c.2247delG |
| 1 | CLCN6 | Exon15 | frameshift deletion | c.1439_1440delAC |
| 1 | CLCNKA | Exon2 | frameshift deletion | c.85delC |
| 2 | CLCNKA | Exon2 | frameshift insertion | c.85_86insGA |
| 1 | CLDN24 | Exon1 | stopgain SNV | c.607C>T |
| 1 | CLDN5 | Exon1 | stopgain SNV | c.109C>T |
| 3 | CLEC12B | Exon2 | frameshift insertion | c.176dupC |
| 2 | CLEC16A | Exon18 | stopgain SNV | c.2063T>A |
| 1 | CLEC18B | Exon5 | frameshift deletion | c.603_604delTG |
| 1 | CLEC1A | Exon5 | frameshift insertion | c.655dupT |
| 1 | CLEC2D | Exon5 | frameshift deletion | c.529delT |
| 1 | CLEC3A | Exon1 | frameshift deletion | c.4_5delGG |
| 1 | CLEC4F | Exon4 | stopgain SNV | c.586C>T |
| 1 | CLEC4F | Exon2 | frameshift deletion | c.74_75delTG |
| 1 | CLEC6A | Exon4 | stopgain SNV | c.249G>A |
| 1 | CLIC2 | Exon5 | frameshift deletion | c.451_454delAACA |
| 8 | CLLU1 | Exon1 | frameshift insertion | c.122dupA |
| 2 | CLNS1A | Exon1 | stopgain SNV | c.114C>G |
| 1 | CLP1 | Exon3 | frameshift insertion | c.577_578insA |
| 1 | CLP1 | Exon3 | frameshift insertion | c.583_584insT |
| 1 | CLPTM1 | Exon8 | stopgain SNV | c.970A>T |
| 1 | CLRN1 | Exon1 | frameshift insertion | c.148_149insTGTC |
| 1 | CLSTN2 | Exon7 | stopgain SNV | c.1151G>A |
| 1 | CLSTN3 | Exon7 | frameshift deletion | c.1066delC |
| 1 | CLTB | Exon5 | splicing | c.353-2A>T |
| 1 | CLTCL1 | Exon29 | frameshift insertion | c.4576dupC |
| 1 | CLTCL1 | Exon29 | frameshift deletion | c.4550_4559del  ATGACCTGCT |
| 1 | CLTC | Exon11 | stopgain SNV | c.1648delG |
| 7 | CLUAP1 | Exon7 | frameshift deletion | c.708delT |
| 1 | CLVS1 | Exon3 | frameshift deletion | c.588delA |
| 1 | CMA1 | Exon5 | frameshift deletion | c.740delA |
| 2 | CMA1 | Exon2 | stopgain SNV | c.143C>G |
| 13 | CMAS | Exon8 | frameshift deletion | c.1223_1224delAA |
| 1 | CMIP | Exon18 | frameshift deletion | c.2035delG |
| 2 | CMPK2 | Exon2 | frameshift deletion | c.714_715delCC |
| 1 | CMTM2 | Exon1 | splicing | c.285+2_285+2delT |
| 2 | CMYA5 | Exon2 | stopgain SNV | c.925A>T |
| 1 | CMYA5 | Exon2 | stopgain SNV | c.3679G>T |
| 1 | CMYA5 | Exon2 | frameshift deletion | c.9614_9617delAAAA |
| 1 | CMYA5 | Exon2 | frameshift deletion | c.10496delA |
| 1 | CNDP1 | Exon2 | frameshift insertion | c.40_44dupGCTGT |
| 1 | CNDP2 | Exon9 | frameshift deletion | c.969delC |
| 1 | CNGA1 | Exon9 | stopgain SNV | c.540delG |
| 1 | CNGA3 | Exon2 | splicing | c.101+1G>A |
| 1 | CNGA3 | Exon7 | frameshift deletion | c.669_670delGG |
| 1 | CNGB3 | Exon17 | frameshift deletion | c.2073delA |
| 4 | CNGB3 | Exon10 | frameshift deletion | c.1148delC |
| 1 | CNKSR1 | Exon5 | splicing | c.519+1G>A |
| 1 | CNKSR1 | Exon7 | frameshift deletion | c.642delC |
| 1 | CNKSR1 | Exon17 | stopgain SNV | c.1492C>T |
| 1 | CNKSR1 | Exon17 | frameshift deletion | c.1514delC |
| 1 | CNKSR2 | Exon4 | splicing | c.519+1G>A |
| 6 | CNKSR3 | Exon12 | frameshift deletion | c.1346delG |
| 1 | CNNM2 | Exon1 | frameshift insertion | c.1620_1621insCT |
| 1 | CNNM2 | Exon1 | frameshift insertion | c.1621_1622insCT |
| 1 | CNNM3 | Exon6 | stopgain SNV | c.1816C>T |
| 8 | CNOT3 | Exon9 | frameshift deletion | c.729delT |
| 1 | CNPY2 | Exon4 | frameshift deletion | c.301delC |
| 1 | CNTD2 | Exon3 | frameshift deletion | c.442delC |
| 2 | CNTF | Exon2 | stopgain SNV | c.191G>A |
| 1 | CNTF | Exon2 | frameshift insertion | c.565_566insCC |
| 1 | CNTLN | Exon7 | stopgain SNV | c.1087C>T |
| 5 | CNTN1 | Exon9 | frameshift deletion | c.845delT |
| 15 | CNTN2 | Exon10 | stopgain SNV | c.1237C>T |
| 1 | CNTN3 | Exon12 | frameshift deletion | c.1580delC |
| 1 | CNTN6 | Exon8 | stopgain SNV | c.814A>T |
| 1 | CNTN6 | Exon13 | frameshift deletion | c.1581_1582delCC |
| 1 | CNTNAP1 | Exon3 | frameshift deletion | c.180delG |
| 9 | CNTNAP1 | Exon17 | splicing | c.2531-2A>T |
| 2 | CNTNAP3B | Exon14 | frameshift deletion | c.2128_2129delAC |
| 14 | CNTNAP3 | Exon10 | frameshift deletion | c.1599delG |
| 1 | CNTNAP3 | Exon2 | splicing | c.85+1G>A |
| 10 | CNTNAP4 | Exon5 | stoploss SNV | c.630A>G |
| 1 | CNTNAP4 | Exon12 | stoploss SNV | c.1877G>C |
| 1 | CNTROB | Exon8 | frameshift insertion | c.1058_1059insGT |
| 1 | COASY | Exon3 | stopgain SNV | c.507T>A |
| 1 | COASY | Exon10 | stopgain SNV | c.1654C>T |
| 1 | COBLL1 | Exon14 | stopgain SNV | c.3428delC |
| 1 | COG2 | Exon7 | frameshift deletion | c.657delT |
| 1 | COG4 | Exon1 | frameshift deletion | c._del |
| 1 | COG7 | Exon12 | frameshift insertion | c.1498dupT |
| 6 | COL11A2 | Exon14 | stopgain SNV | c.1482C>A |
| 1 | COL13A1 | Exon27 | frameshift insertion | c.1407dupT |
| 1 | COL13A1 | Exon27 | frameshift deletion | c.1431delT |
| 1 | COL14A1 | Exon7 | frameshift deletion | c.689_690delAT |
| 1 | COL15A1 | Exon12 | splicing | c.1701+1G>T |
| 1 | COL16A1 | Exon55 | splicing | c.3403-2_3403-1delAG |
| 1 | COL16A1 | Exon25 | frameshift insertion | c.1708_1709insG |
| 2 | COL16A1 | Exon25 | splicing | c.1656+1G>T |
| 1 | COL17A1 | Exon43 | frameshift insertion | c.2870dupG |
| 1 | COL19A1 | Exon51 | frameshift deletion | c.3339delA |
| 4 | COL1A1 | Exon43 | splicing | c.3099+1G>A |
| 1 | COL1A1 | Exon31 | frameshift deletion | c.2103delT |
| 1 | COL22A1 | Exon65 | frameshift deletion | c.4852delG |
| 1 | COL22A1 | Exon64 | frameshift deletion | c.4646delC |
| 1 | COL23A1 | Exon14 | splicing | c.774+2T>C |
| 2 | COL24A1 | Exon36 | stopgain SNV | c.3157G>T |
| 3 | COL24A1 | Exon36 | frameshift deletion | c.3152delC |
| 1 | COL25A1 | Exon12 | stopgain SNV | c.766delA |
| 1 | COL28A1 | Exon30 | stopgain SNV | c.2293C>T |
| 2 | COL28A1 | Exon29 | stopgain SNV | c.2263G>T |
| 1 | COL2A1 | Exon52 | frameshift deletion | c.4103delG |
| 1 | COL4A1 | Exon51 | stopgain SNV | c.4874dupA |
| 1 | COL4A1 | Exon46 | frameshift deletion | c.4088delG |
| 1 | COL4A1 | Exon37 | frameshift deletion | c.3102_3103delAG |
| 1 | COL4A2 | Exon42 | frameshift insertion | c.3897_3898insCT |
| 1 | COL4A4 | Exon48 | frameshift deletion | c.4932delC |
| 13 | COL4A5 | Exon8 | frameshift deletion | c.459delT |
| 1 | COL4A5 | Exon8 | stopgain SNV | c.460delA |
| 1 | COL4A5 | Exon44 | frameshift insertion | c.4006_4007insA |
| 1 | COL4A6 | Exon43 | frameshift insertion | c.4499_4500insT |
| 2 | COL4A6 | Exon29 | frameshift insertion | c.2724_2725insC |
| 1 | COL5A1 | Exon2 | frameshift insertion | c.202dupT |
| 1 | COL5A2 | Exon1 | frameshift deletion | c.7delG |
| 5 | COL6A5 | Exon8 | frameshift deletion | c.3452delT |
| 1 | COL6A5 | Exon35 | stopgain SNV | c.5854C>T |
| 2 | COL6A5 | Exon35 | stopgain SNV | c.6148C>T |
| 8 | COL6A5 | Exon38 | stopgain SNV | c.6814G>T |
| 1 | COL7A1 | Exon3 | stopgain SNV | c.337A>T |
| 1 | COL7A1 | Exon1 | stopgain SNV | c.49G>T |
| 1 | COL9A1 | Exon3 | splicing | c.147+2T>A |
| 1 | COL9A2 | Exon31 | splicing | c.1604-10_1604-3del  CTCCCTCC |
| 2 | COL9A2 | Exon19 | stopgain SNV | c.976C>T |
| 1 | COLEC11 | Exon6 | frameshift deletion | c.300delT |
| 1 | COLEC12 | Exon5 | frameshift deletion | c.761delA |
| 1 | COMMD10 | Exon5 | stopgain SNV | c.481C>T |
| 1 | COMMD2 | Exon4 | stopgain SNV | c.268G>T |
| 1 | COPB1 | Exon15 | frameshift deletion | c.1753delG |
| 1 | COPG | Exon17 | frameshift deletion | c.1654delA |
| 2 | COPS7A | Exon8 | stopgain SNV | c.805A>T |
| 1 | COPZ1 | Exon4 | frameshift deletion | c.243delC |
| 1 | COQ5 | Exon5 | stopgain SNV | c.736G>T |
| 4 | COQ6 | Exon1 | stopgain SNV | c.41G>A |
| 1 | CORO1C | Exon11 | frameshift deletion | c.1311delT |
| 1 | CORO2A | Exon12 | stoploss SNV | c.1577dupG |
| 11 | CORO2A | Exon12 | stoploss SNV | c.1576T>A |
| 1 | CORO2A | Exon6 | splicing | c.469-1G>C |
| 1 | CORO2B | Exon5 | frameshift deletion | c.606_609delGAAG |
| 2 | COX6A1 | Exon1 | frameshift deletion | c.59delT |
| 1 | COX6B2 | Exon3 | frameshift insertion | c.194dupC |
| 1 | CPA1 | Exon2 | stopgain SNV | c.79C>T |
| 2 | CPA4 | Exon7 | stopgain SNV | c.678G>A |
| 1 | CPB2 | Exon2 | frameshift deletion | c.128delA |
| 1 | CPD | Exon2 | frameshift deletion | c.868delT |
| 1 | CPEB3 | Exon10 | frameshift insertion | c.1881_1882insA |
| 1 | CPEB4 | Exon1 | frameshift deletion | c.325delG |
| 1 | CPNE1 | Exon7 | frameshift deletion | c.463delC |
| 1 | CPNE3 | Exon13 | splicing | c.1068+2T>G |
| 1 | CPNE7 | Exon8 | stopgain SNV | c.784C>T |
| 1 | CPNE9 | Exon6 | splicing | c.377+1G>A |
| 1 | CPO | Exon3 | stopgain SNV | c.177G>A |
| 1 | CPS1 | Exon15 | frameshift deletion | c.1547delG |
| 1 | CPSF1 | Exon25 | frameshift deletion | c.2823_2824delAG |
| 1 | CPSF1 | Exon25 | frameshift deletion | c.2741_2754delAAGGTGGCGGCGCA |
| 2 | CPSF1 | Exon4 | frameshift deletion | c.205delG |
| 1 | CPSF1 | Exon3 | frameshift insertion | c.155_156insT |
| 1 | CPT2 | Exon4 | frameshift deletion | c.1239_1240delGA |
| 3 | CPVL | Exon13 | stopgain SNV | c.1422T>A |
| 1 | CPVL | Exon11 | frameshift deletion | c.1030delG |
| 13 | CPVL | Exon10 | stopgain SNV | c.933T>A |
| 1 | CPXM1 | Exon14 | stoploss SNV | c.1982G>C |
| 1 | CPXM1 | Exon5 | frameshift deletion | c.623delT |
| 1 | CPXM2 | Exon9 | frameshift deletion | c.1149delG |
| 1 | CPXM2 | Exon6 | frameshift deletion | c.802delG |
| 1 | CPZ | Exon10 | stopgain SNV | c.1641C>G |
| 1 | CR2 | Exon4 | stopgain SNV | c.658C>T |
| 1 | CRAT | Exon10 | frameshift deletion | c.1325delA |
| 1 | CRB1 | Exon4 | frameshift insertion | c.1745_1746dupAC |
| 1 | CRB1 | Exon10 | frameshift deletion | c.3703delA |
| 1 | CRBN | Exon5 | splicing | c.527+1_527+1delG |
| 1 | CRCT1 | Exon2 | stopgain SNV | c.13C>T |
| 1 | CREB3L1 | Exon10 | splicing | c.1132-1G>A |
| 1 | CREB3L1 | Exon11 | splicing | c.1523+2_1523+2delT |
| 3 | CREBBP | Exon31 | frameshift deletion | c.5605delA |
| 1 | CREG2 | Exon4 | stopgain SNV | c.809G>A |
| 1 | CRELD1 | Exon7 | frameshift deletion | c.785delG |
| 1 | CRELD1 | Exon7 | frameshift insertion | c.796dupG |
| 1 | CRHR2 | Exon13 | frameshift deletion | c.1254delG |
| 3 | CRHR2 | Exon13 | frameshift deletion | c.1241delT |
| 1 | CRHR2 | Exon13 | stopgain SNV | c.1231C>T |
| 6 | CRIPAK | Exon1 | frameshift insertion | c.77_78dupCA |
| 2 | CRIPAK | Exon1 | frameshift deletion | c.264_265delAT |
| 1 | CRIPAK | Exon1 | frameshift deletion | c.272_276delATGTG |
| 10 | CRIPAK | Exon1 | frameshift insertion | c.324_325dupCA |
| 1 | CRIPAK | Exon1 | frameshift deletion | c.1160_1163delACAC |
| 1 | CRISP1 | Exon6 | stopgain SNV | c.502C>T |
| 1 | CRISPLD1 | Exon2 | stopgain SNV | c.9T>A |
| 1 | CRISPLD2 | Exon11 | splicing | c.1113-1_1113-1delG |
| 1 | CRLF1 | Exon5 | frameshift deletion | c.845_846delTG |
| 1 | CRNKL1 | Exon2 | stopgain SNV | c.148C>T |
| 1 | CROCC | Exon10 | frameshift insertion | c.1207_1208insA |
| 1 | CROCC | Exon33 | stopgain SNV | c.5383C>T |
| 2 | CROT | Exon18 | frameshift insertion | c.1761dupA |
| 1 | CRTC2 | Exon6 | splicing | c.435-1G>A |
| 1 | CRX | Exon4 | frameshift deletion | c.466_467delGG |
| 1 | CRY1 | Exon10 | frameshift deletion | c.1565delC |
| 1 | CRY1 | Exon6 | frameshift insertion | c.801_802insG |
| 1 | CRY1 | Exon6 | frameshift deletion | c.797_798delAC |
| 7 | CRYBA4 | Exon3 | splicing | c.40-1G>C |
| 1 | CRYBA4 | Exon3 | stopgain SNV | c.67C>T |
| 1 | CRYBB2 | Exon5 | splicing | c.449+1G>A |
| 1 | CRYGA | Exon3 | stopgain SNV | c.295C>T |
| 2 | CRYGA | Exon2 | stopgain SNV | c.128G>A |
| 1 | CRYGC | Exon2 | stopgain SNV | c.128G>A |
| 20 | CSAG1 | Exon4 | stopgain SNV | c.84C>G |
| 1 | CSAG1 | Exon4 | stopgain SNV | c.121C>T |
| 1 | CSDC2 | Exon4 | frameshift insertion | c.377_378dupAG |
| 1 | CSE1L | Exon6 | frameshift insertion | c.485dupA |
| 10 | CSF1R | Exon16 | frameshift deletion | c.2168_2169delAT |
| 1 | CSF1R | Exon6 | stopgain SNV | c.789_790insT |
| 1 | CSF2RB | Exon7 | frameshift insertion | c.747_748insGG |
| 3 | CSF2RB | Exon14 | frameshift deletion | c.2124delC |
| 1 | CSF3 | Exon3 | frameshift deletion | c.198delA |
| 3 | CSGALNACT2 | Exon5 | stopgain SNV | c.1039C>T |
| 1 | CSH1 | Exon4 | frameshift deletion | c.345delC |
| 2 | CSH2 | Exon3 | stoploss SNV | c.367T>C |
| 1 | CSHL1 | Exon3 | frameshift insertion | c.189_190insCA |
| 2 | CSHL1 | Exon3 | frameshift deletion | c.146_149delTGGT |
| 5 | CSMD1 | Exon58 | stopgain SNV | c.8936G>A |
| 10 | CSMD1 | Exon41 | stoploss SNV | c.6196T>C |
| 2 | CSMD1 | Exon15 | stopgain SNV | c.2079G>A |
| 1 | CSMD2 | Exon65 | frameshift deletion | c.9947_9948delTG |
| 1 | CSMD3 | Exon44 | stopgain SNV | c.6708G>A |
| 1 | CSMD3 | Exon30 | stopgain SNV | c.4780C>T |
| 1 | CSMD3 | Exon1 | frameshift deletion | c.115_118delGGGA |
| 4 | CSN1S1 | Exon7 | splicing | c.154-1G>C |
| 1 | CS | Exon6 | stopgain SNV | c.423G>A |
| 1 | CSPG4 | Exon10 | frameshift deletion | c.6392delA |
| 1 | CSPG4 | Exon10 | frameshift insertion | c.6384dupC |
| 1 | CSPG4 | Exon10 | frameshift deletion | c.5997delC |
| 1 | CSPG4 | Exon5 | frameshift deletion | c.4305delC |
| 1 | CSPP1 | Exon19 | frameshift deletion | c.2506_2509delATTG |
| 2 | CST2 | Exon2 | stopgain SNV | c.271C>T |
| 1 | CST3 | Exon3 | stopgain SNV | c.397C>T |
| 1 | CST3 | Exon2 | frameshift deletion | c.277_296del  GAGCTGGGCCGAACCACGTG |
| 1 | CST4 | Exon2 | frameshift insertion | c.241dupG |
| 1 | CST7 | Exon2 | frameshift deletion | c.97delC |
| 1 | CST8 | Exon3 | stopgain SNV | c.252C>A |
| 1 | CST8 | Exon3 | stopgain SNV | c.322C>T |
| 1 | CST9 | Exon2 | frameshift deletion | c.390delT |
| 1 | CSTL1 | Exon3 | splicing | c.330+2T>C |
| 1 | CT62 | Exon4 | stopgain SNV | c.207T>A |
| 1 | CTAGE9 | Exon1 | frameshift deletion | c.1462_1465delCAAA |
| 14 | CTBP2 | Exon11 | stopgain SNV | c.1333C>T |
| 7 | CTBP2 | Exon11 | frameshift deletion | c.1291delC |
| 1 | CTC1 | Exon21 | splicing | c.3221+1G>A |
| 1 | CTCFL | Exon2 | frameshift insertion | c.382_383insCG |
| 5 | CTDSP2 | Exon7 | frameshift deletion | c.639_640delCC |
| 6 | CTDSP2 | Exon7 | frameshift deletion | c.636delG |
| 1 | CTF1 | Exon2 | splicing | c.26-2A>T |
| 2 | CTH | Exon11 | frameshift deletion | c.1064delC |
| 1 | CTNNA2 | Exon18 | stopgain SNV | c.2690T>G |
| 1 | CTNNA3 | Exon15 | frameshift deletion | c.2104_2105delAT |
| 3 | CTNNAL1 | Exon11 | stopgain SNV | c.1547C>G |
| 15 | CTNND2 | Exon22 | splicing | c.3417+2_3417+5dupTAAA |
| 1 | CTPS | Exon18 | splicing | c.1692-1G>C |
| 1 | CTR9 | Exon4 | frameshift deletion | c.429delC |
| 1 | CTSA | Exon2 | frameshift deletion | c.51delG |
| 1 | CTSA | Exon2 | frameshift deletion | c.53_54delTG |
| 1 | CTSB | Exon6 | stopgain SNV | c.423C>A |
| 1 | CTSB | Exon5 | stopgain SNV | c.265C>T |
| 1 | CTSC | Exon7 | frameshift deletion | c.1122delT |
| 1 | CTSE | Exon6 | splicing | c.785+2T>C |
| 1 | CTSE | Exon9 | splicing | c.1027-2_1027-2delA |
| 1 | CTSE | Exon9 | stopgain SNV | c.1126C>T |
| 1 | CTSW | Exon3 | frameshift deletion | c.247delA |
| 1 | CTSW | Exon10 | splicing | c.1021-1G>C |
| 1 | CUBN | Exon66 | stopgain SNV | c.10612G>T |
| 1 | CUBN | Exon14 | frameshift deletion | c.1690_1693delCTCT |
| 1 | CUL4B | Exon3 | frameshift insertion | c.294_295insA |
| 1 | CUL7 | Exon25 | stopgain SNV | c.4969C>T |
| 1 | CUL7 | Exon12 | splicing | c.2650-2A>C |
| 1 | CUL9 | Exon4 | frameshift deletion | c.963delG |
| 1 | CUL9 | Exon4 | frameshift deletion | c.972_976delCGAAC |
| 1 | CUL9 | Exon26 | frameshift deletion | c.5165delC |
| 1 | CUTA | Exon5 | stoploss SNV | c.470G>C |
| 1 | CUTC | Exon8 | frameshift insertion | c.668dupG |
| 2 | CWC22 | Exon11 | splicing | c.1147+2dupT |
| 1 | CWC25 | Exon11 | splicing | c.1164-10_1164-2del  CTCTTCCTA |
| 1 | CWC25 | Exon8 | stopgain SNV | c.979C>T |
| 5 | CWC25 | Exon3 | frameshift deletion | c.262delC |
| 9 | CWH43 | Exon12 | frameshift deletion | c.1596delA |
| 4 | CWH43 | Exon16 | frameshift deletion | c.2088delA |
| 1 | CX3CR1 | Exon2 | frameshift deletion | c.984delT |
| 1 | CXADR | Exon4 | frameshift deletion | c.544_545delCA |
| 1 | CXCL5 | Exon1 | frameshift deletion | c.30delT |
| 7 | CXCL6 | Exon2 | frameshift insertion | c.239dupT |
| 1 | CXCL9 | Exon4 | stopgain SNV | c.340C>T |
| 1 | CXCR2 | Exon4 | frameshift deletion | c.674delT |
| 1 | CXORF22 | Exon11 | stopgain SNV | c.1936A>T |
| 1 | CXORF22 | Exon16 | frameshift deletion | c.2857delC |
| 1 | CXORF23 | Exon2 | stopgain SNV | c.366C>G |
| 1 | CXORF40B | Exon4 | frameshift deletion | c.110_111delGT |
| 2 | CXORF56 | Exon2 | frameshift insertion | c.155_156insT |
| 7 | CXORF58 | Exon4 | splicing | c.311+1G>A |
| 2 | CYB561D2 | Exon4 | stopgain SNV | c.661C>T |
| 1 | CYB5D1 | Exon2 | splicing | c.237+1G>C |
| 1 | CYB5R1 | Exon6 | splicing | c.346-1G>T |
| 1 | CYB5R4 | Exon1 | frameshift insertion | c.66dupG |
| 1 | CYB5R4 | Exon3 | stopgain SNV | c.255T>G |
| 1 | CYFIP1 | Exon29 | frameshift deletion | c.3315delT |
| 1 | CYFIP1 | Exon29 | frameshift deletion | c.3325delT |
| 1 | CYFIP2 | Exon11 | frameshift deletion | c.1096delT |
| 2 | CYFIP2 | Exon12 | stopgain SNV | c.1204A>T |
| 1 | CYP11B2 | Exon3 | frameshift deletion | c.510_514delGGCCC |
| 1 | CYP11B2 | Exon3 | splicing | c.395+1G>A |
| 1 | CYP11B2 | Exon1 | frameshift deletion | c.63_64delAC |
| 1 | CYP19A1 | Exon11 | frameshift deletion | c.1386delA |
| 16 | CYP19A1 | Exon5 | frameshift deletion | c.411delT |
| 1 | CYP1A1 | Exon7 | stopgain SNV | c.1371delT |
| 1 | CYP1A1 | Exon2 | frameshift deletion | c.316delC |
| 1 | CYP1B1 | Exon3 | frameshift insertion | c.1200_1209dup  TCATGCCACC |
| 1 | CYP24A1 | Exon6 | stopgain SNV | c.803G>A |
| 1 | CYP24A1 | Exon2 | frameshift deletion | c.351_352delGC |
| 1 | CYP26C1 | Exon4 | frameshift insertion | c.845_851dupCCATGCA |
| 3 | CYP2A13 | Exon2 | stopgain SNV | c.301C>T |
| 1 | CYP2A13 | Exon8 | frameshift insertion | c.1228dupG |
| 1 | CYP2A6 | Exon9 | stopgain SNV | c.1400C>A |
| 5 | CYP2C18 | Exon2 | stopgain SNV | c.204T>A |
| 1 | CYP2C19 | Exon4 | stopgain SNV | c.636G>A |
| 1 | CYP2C9 | Exon4 | splicing | c.642+2T>A |
| 2 | CYP2C9 | Exon5 | frameshift deletion | c.818delA |
| 11 | CYP2D6 | Exon5 | frameshift deletion | c.775delA |
| 1 | CYP2D6 | Exon3 | frameshift deletion | c.454delT |
| 2 | CYP2E1 | Exon4 | splicing | c.648+1G>A |
| 1 | CYP2J2 | Exon2 | frameshift deletion | c.232delC |
| 1 | CYP2S1 | Exon4 | stopgain SNV | c.595C>T |
| 1 | CYP3A43 | Exon3 | stopgain SNV | c.204C>G |
| 1 | CYP3A4 | Exon13 | frameshift insertion | c.1461dupA |
| 1 | CYP3A4 | Exon8 | frameshift insertion | c.719dupT |
| 2 | CYP3A5 | Exon12 | frameshift deletion | c.1372delG |
| 5 | CYP3A5 | Exon11 | frameshift insertion | c.1035dupT |
| 4 | CYP3A5 | Exon4 | frameshift deletion | c.258delT |
| 1 | CYP3A5 | Exon4 | frameshift insertion | c.247dupG |
| 2 | CYP3A5 | Exon2 | frameshift insertion | c.92dupG |
| 1 | CYP46A1 | Exon5 | splicing | c.443+1_443+2insGCT |
| 1 | CYP4A22 | Exon5 | stopgain SNV | c.559C>T |
| 1 | CYP4A22 | Exon8 | frameshift deletion | c.901delG |
| 1 | CYP4B1 | Exon2 | stopgain SNV | c.285T>A |
| 1 | CYP4F11 | Exon1 | frameshift deletion | c.53delC |
| 2 | CYP4F12 | Exon8 | splicing | c.985+1G>A |
| 1 | CYP4F3 | Exon3 | stopgain SNV | c.269G>A |
| 1 | CYP4F8 | Exon12 | splicing | c.1314-1G>C |
| 1 | CYP7A1 | Exon3 | stopgain SNV | c.592C>T |
| 1 | CYP8B1 | Exon1 | frameshift deletion | c.638delT |
| 1 | CYTH1 | Exon11 | splicing | c.813-2G>T |
| 1 | CYYR1 | Exon3 | splicing | c.176+1G>A |
| 1 | DAB2IP | Exon11 | frameshift deletion | c.1955delC |
| 1 | DAB2IP | Exon13 | frameshift insertion | c.2989dupC |
| 1 | DAB2 | Exon12 | frameshift insertion | c.1627_1628insT |
| 1 | DAB2 | Exon12 | frameshift deletion | c.1595delT |
| 1 | DAGLB | Exon3 | frameshift deletion | c.268_269delCC |
| 1 | DAK | Exon3 | frameshift insertion | c.63_64insA |
| 5 | DAPL1 | Exon4 | stopgain SNV | c.313C>T |
| 1 | DARS2 | Exon5 | splicing | c.492+2T>C |
| 1 | DAXX | Exon3 | frameshift insertion | c.589dupC |
| 1 | DAZAP1 | Exon3 | frameshift deletion | c.211delA |
| 4 | DBF4B | Exon1 | splicing | c.19+1G>T |
| 1 | DBF4 | Exon5 | frameshift deletion | c.500delT |
| 1 | DBT | Exon2 | frameshift deletion | c.75_76delAT |
| 2 | DCAF11 | Exon15 | stopgain SNV | c.1536C>A |
| 1 | DCAF15 | Exon7 | frameshift deletion | c.851delC |
| 1 | DCAF4L2 | Exon1 | frameshift deletion | c.1115_1116delTC |
| 2 | DCAF4L2 | Exon1 | frameshift deletion | c.145delC |
| 18 | DCAF4L2 | Exon1 | frameshift insertion | c.131dupG |
| 1 | DCAF4L2 | Exon1 | frameshift deletion | c.49_50delAA |
| 1 | DCAF6 | Exon19 | frameshift deletion | c.2504delT |
| 7 | DCAKD | Exon2 | frameshift deletion | c.21_22delAG |
| 7 | DCDC2B | Exon1 | frameshift insertion | c.52dupG |
| 1 | DCDC5 | Exon11 | stopgain SNV | c.1240G>T |
| 1 | DCD | Exon4 | frameshift insertion | c.225dupA |
| 1 | DCHS1 | Exon2 | frameshift deletion | c.1463delC |
| 1 | DCLRE1A | Exon2 | stopgain SNV | c.572C>A |
| 2 | DCLRE1A | Exon1 | stopgain SNV | c.412C>T |
| 1 | DCP1A | Exon7 | frameshift deletion | c.780_781delTC |
| 1 | DCST2 | Exon8 | frameshift deletion | c.1213delT |
| 1 | DCST2 | Exon4 | frameshift deletion | c.726delT |
| 1 | DCTN1 | Exon18 | frameshift deletion | c.2332delG |
| 3 | DCTN1 | Exon6 | frameshift deletion | c.707delA |
| 1 | DCTN6 | Exon3 | splicing | c.194+1_194+2insTC |
| 1 | DCUN1D2 | Exon3 | stopgain SNV | c.262C>T |
| 1 | DDB1 | Exon6 | frameshift insertion | c.685dupG |
| 2 | DDIT3 | Exon4 | frameshift insertion | c.332_333insTG |
| 1 | DDIT4 | Exon3 | frameshift deletion | c.275delT |
| 1 | DDO | Exon5 | stopgain SNV | c.632delC |
| 1 | DDOST | Exon5 | frameshift deletion | c.597delT |
| 1 | DDOST | Exon5 | frameshift deletion | c.591delT |
| 3 | DDTL | Exon3 | frameshift deletion | c.341_342delGA |
| 3 | DDTL | Exon3 | frameshift deletion | c.345_346delGA |
| 1 | DDX25 | Exon7 | splicing | c.622+1G>T |
| 1 | DDX27 | Exon14 | frameshift deletion | c.1658delG |
| 1 | DDX27 | Exon20 | stopgain SNV | c.2344C>T |
| 1 | DDX39A | Exon10 | frameshift insertion | c.1131_1132insTT |
| 1 | DDX41 | Exon9 | frameshift deletion | c.825delC |
| 1 | DDX42 | Exon15 | splicing | c.1902+1_1902+1delG |
| 1 | DDX49 | Exon11 | frameshift deletion | c.1166delA |
| 1 | DDX49 | Exon13 | frameshift deletion | c.1273delC |
| 8 | DDX54 | Exon18 | stopgain SNV | c.2274C>G |
| 2 | DDX55 | Exon14 | frameshift insertion | c.1775_1776dupGG |
| 1 | DDX58 | Exon18 | frameshift insertion | c.2587_2590dupTTCT |
| 1 | DDX59 | Exon2 | frameshift deletion | c.110delG |
| 1 | DDX60L | Exon30 | stopgain SNV | c.4063C>T |
| 2 | DDX60L | Exon18 | splicing | c.2245-1G>A |
| 1 | DDX60L | Exon4 | frameshift deletion | c._del |
| 1 | DDX60 | Exon36 | frameshift deletion | c.4941delA |
| 1 | DDX60 | Exon5 | stopgain SNV | c.358C>T |
| 4 | DEFA6 | Exon1 | stopgain SNV | c.121C>T |
| 1 | DEFB108B | Exon1 | frameshift deletion | c.33delC |
| 1 | DEFB126 | Exon2 | frameshift deletion | c.222_225delGACA |
| 1 | DEFB126 | Exon2 | frameshift deletion | c.313_314delAC |
| 17 | DEFB128 | Exon2 | frameshift insertion | c.183dupA |
| 2 | DEFB132 | Exon2 | stoploss SNV | c.287A>C |
| 2 | DEM1 | Exon3 | frameshift deletion | c.794_795delTG |
| 15 | DEM1 | Exon3 | frameshift insertion | c.1029_1030insG |
| 10 | DENND1B | Exon21 | stopgain SNV | c.1525A>T |
| 1 | DENND1C | Exon3 | frameshift insertion | c.124dupC |
| 1 | DENND1C | Exon3 | frameshift insertion | c.114_115insA |
| 1 | DENND2D | Exon1 | stopgain SNV | c.61C>T |
| 1 | DENND4B | Exon3 | frameshift deletion | c.533delC |
| 1 | DENND4C | Exon18 | stopgain SNV | c.3452C>G |
| 1 | DENND4C | Exon21 | frameshift deletion | c.3864delC |
| 1 | DEPDC1 | Exon10 | frameshift deletion | c.2065_2068delTTAC |
| 1 | DEPDC7 | Exon2 | frameshift deletion | c.430_433delTTAG |
| 1 | DEPTOR | Exon2 | frameshift deletion | c.284_288delGGGGC |
| 1 | DERL3 | Exon4 | frameshift deletion | c.314delG |
| 1 | DFFA | Exon4 | frameshift deletion | c.477_480delGGAA |
| 6 | DFNA5 | Exon2 | frameshift insertion | c.119dupA |
| 1 | DGAT2L6 | Exon4 | stopgain SNV | c.451C>T |
| 16 | DGAT2 | Exon3 | stopgain SNV | c.355A>T |
| 1 | DGCR14 | Exon10 | stopgain SNV | c.1418C>A |
| 17 | DGCR2 | Exon8 | stopgain SNV | c.1123G>T |
| 1 | DGCR8 | Exon2 | frameshift insertion | c.65_66insT |
| 1 | DGCR8 | Exon2 | frameshift deletion | c.126_127delGT |
| 1 | DGCR8 | Exon2 | frameshift insertion | c.131_132insGG |
| 1 | DGCR8 | Exon2 | frameshift deletion | c.376delG |
| 1 | DHCR24 | Exon5 | frameshift insertion | c.717_718insA |
| 1 | DHCR24 | Exon5 | stopgain SNV | c.711C>A |
| 3 | DHCR7 | Exon10 | splicing | c.964-1G>C |
| 1 | DHCR7 | Exon6 | stopgain SNV | c.452G>A |
| 1 | DHDH | Exon2 | stopgain SNV | c.139C>T |
| 1 | DHRS12 | Exon7 | frameshift deletion | c.478delG |
| 1 | DHRS2 | Exon3 | stopgain SNV | c.208C>T |
| 1 | DHRS2 | Exon7 | frameshift deletion | c.666delC |
| 1 | DHRS3 | Exon2 | frameshift deletion | c.331delC |
| 1 | DHRS3 | Exon2 | frameshift deletion | c.326_327delCC |
| 6 | DHRS4L2 | Exon4 | frameshift insertion | c.203_204insA |
| 1 | DHRS7B | Exon4 | frameshift deletion | c.425delA |
| 1 | DHTKD1 | Exon7 | stopgain SNV | c.1246C>T |
| 1 | DHTKD1 | Exon7 | splicing | c.1358+1G>A |
| 1 | DHX30 | Exon6 | frameshift deletion | c.353delG |
| 1 | DHX30 | Exon6 | stopgain SNV | c.354C>A |
| 1 | DHX32 | Exon11 | frameshift deletion | c.2156_2157delCT |
| 1 | DHX32 | Exon7 | splicing | c.1193-1G>C |
| 1 | DHX37 | Exon10 | frameshift insertion | c.1294_1295insC |
| 1 | DHX37 | Exon10 | frameshift insertion | c.1293_1294insT |
| 1 | DHX40 | Exon14 | stopgain SNV | c.1627C>T |
| 1 | DHX58 | Exon10 | frameshift deletion | c.1340delT |
| 1 | DHX58 | Exon6 | splicing | c.561+1_561+2delGT |
| 1 | DHX58 | Exon5 | stopgain SNV | c.472C>T |
| 2 | DIAPH1 | Exon15 | frameshift insertion | c.2081dupC |
| 1 | DIAPH3 | Exon21 | frameshift deletion | c.2546delA |
| 1 | DIDO1 | Exon16 | frameshift deletion | c.5384delC |
| 1 | DIDO1 | Exon3 | frameshift deletion | c.137delA |
| 1 | DIDO1 | Exon3 | frameshift deletion | c.133delG |
| 18 | DIMT1 | Exon11 | frameshift deletion | c.876delC |
| 2 | DIMT1 | Exon9 | splicing | c.571-2A>G |
| 1 | DIP2A | Exon5 | frameshift deletion | c.489delC |
| 3 | DIP2C | Exon31 | stopgain SNV | c.3805G>T |
| 3 | DIS3 | Exon21 | stoploss SNV | c.2875T>C |
| 1 | DIS3 | Exon2 | stopgain SNV | c.274C>T |
| 1 | DISP2 | Exon8 | frameshift deletion | c.2411delG |
| 1 | DIXDC1 | Exon15 | splicing | c.1526+1G>A |
| 1 | DKK3 | Exon3 | stopgain SNV | c.337C>T |
| 1 | DKKL1 | Exon5 | frameshift insertion | c.270_271insT |
| 1 | DLC1 | Exon13 | frameshift insertion | c.3730_3731insG |
| 1 | DLEC1 | Exon25 | frameshift insertion | c.3654_3655insTG |
| 1 | DLEC1 | Exon27 | splicing | c.3786-1G>A |
| 1 | DLEC1 | Exon36 | stopgain SNV | c.5088C>A |
| 1 | DLGAP3 | Exon1 | frameshift deletion | c.566delC |
| 1 | DLGAP3 | Exon1 | frameshift insertion | c.254dupG |
| 1 | DLGAP5 | Exon19 | stopgain SNV | c.2491C>T |
| 1 | DLL3 | Exon7 | splicing | c.1673+1G>A |
| 1 | DLX3 | Exon1 | frameshift insertion | c.181_182insCCCA |
| 1 | DLX3 | Exon1 | stopgain SNV | c.79A>T |
| 1 | DMBT1 | Exon7 | frameshift deletion | c.575delG |
| 1 | DMBT1 | Exon40 | stopgain SNV | c.4942C>T |
| 1 | DMC1 | Exon9 | frameshift deletion | c.579delA |
| 2 | DMD | Exon41 | frameshift deletion | c.5851delA |
| 1 | DMD | Exon8 | splicing | c.507-11_507-6delATGTGT |
| 3 | DMGDH | Exon6 | stopgain SNV | c.972G>A |
| 1 | DMGDH | Exon6 | stopgain SNV | c.856C>T |
| 1 | DMKN | Exon2 | frameshift deletion | c.571delG |
| 1 | DMRTA2 | Exon3 | stoploss SNV | c.892T>A |
| 17 | DMXL1 | Exon18 | frameshift deletion | c.4445delG |
| 1 | DMXL1 | Exon18 | frameshift deletion | c.4457delC |
| 1 | DNAH10 | Exon9 | stopgain SNV | c.1135C>T |
| 1 | DNAH10 | Exon49 | frameshift deletion | c.8272delC |
| 1 | DNAH10 | Exon73 | stopgain SNV | c.12520C>T |
| 1 | DNAH10 | Exon76 | frameshift deletion | c.13032delG |
| 1 | DNAH10 | Exon77 | stopgain SNV | c.13163G>A |
| 7 | DNAH11 | Exon1 | stopgain SNV | c.100G>T |
| 1 | DNAH11 | Exon11 | stopgain SNV | c.1915C>T |
| 1 | DNAH11 | Exon37 | stopgain SNV | c.6286C>T |
| 2 | DNAH11 | Exon61 | stoploss SNV | c.9957A>T |
| 1 | DNAH11 | Exon63 | splicing | c.10354+2T>C |
| 1 | DNAH11 | Exon71 | stopgain SNV | c.11692C>T |
| 1 | DNAH14 | Exon2 | stopgain SNV | c.55G>T |
| 1 | DNAH14 | Exon6 | stopgain SNV | c.604A>T |
| 1 | DNAH14 | Exon15 | frameshift deletion | c.1867delT |
| 2 | DNAH14 | Exon19 | frameshift deletion | c.2973delC |
| 1 | DNAH14 | Exon30 | frameshift insertion | c.4872dupA |
| 3 | DNAH14 | Exon37 | frameshift deletion | c.5773_5776delAAAG |
| 1 | DNAH17 | Exon55 | frameshift insertion | c.8771_8772insT |
| 1 | DNAH17 | Exon26 | splicing | c.3864+1_3864+3delGTG |
| 1 | DNAH1 | Exon65 | frameshift deletion | c.10468_10471delAGAG |
| 1 | DNAH1 | Exon71 | frameshift deletion | c.11275delC |
| 1 | DNAH1 | Exon72 | frameshift deletion | c.11464_11476del  TGCTTGTTCCATG |
| 1 | DNAH2 | Exon8 | frameshift deletion | c.1331delG |
| 3 | DNAH2 | Exon45 | frameshift deletion | c.7068delG |
| 1 | DNAH2 | Exon65 | stopgain SNV | c.10026G>A |
| 1 | DNAH2 | Exon82 | frameshift deletion | c.12806delG |
| 1 | DNAH3 | Exon53 | stopgain SNV | c.9134T>G |
| 5 | DNAH3 | Exon14 | stopgain SNV | c.2028T>A |
| 1 | DNAH3 | Exon7 | stopgain SNV | c.1017G>A |
| 1 | DNAH5 | Exon62 | stopgain SNV | c.10441C>T |
| 1 | DNAH5 | Exon58 | splicing | c.9606-2A>C |
| 1 | DNAH5 | Exon56 | frameshift insertion | c.9431_9432insT |
| 1 | DNAH5 | Exon48 | frameshift deletion | c.7946delG |
| 1 | DNAH6 | Exon11 | stopgain SNV | c.1801A>T |
| 3 | DNAH7 | Exon65 | stopgain SNV | c.11934T>A |
| 1 | DNAH7 | Exon40 | stopgain SNV | c.6429dupT |
| 1 | DNAH7 | Exon35 | stopgain SNV | c.5600T>A |
| 2 | DNAH7 | Exon30 | stopgain SNV | c.4787dupA |
| 1 | DNAH7 | Exon27 | stopgain SNV | c.4309C>T |
| 2 | DNAH7 | Exon24 | frameshift deletion | c.3833delA |
| 1 | DNAH7 | Exon14 | stopgain SNV | c.1687C>T |
| 2 | DNAH8 | Exon12 | splicing | c.1764+2T>C |
| 3 | DNAH8 | Exon18 | frameshift deletion | c.2457delT |
| 1 | DNAH8 | Exon50 | stopgain SNV | c.7084C>T |
| 1 | DNAH8 | Exon77 | splicing | c.11563+1G>T |
| 1 | DNAH8 | Exon90 | frameshift deletion | c.13393delA |
| 1 | DNAH9 | Exon20 | splicing | c.4614+2T>A |
| 1 | DNAH9 | Exon46 | stopgain SNV | c.8761delG |
| 1 | DNAI1 | Exon1 | splicing | c.48+2dupT |
| 1 | DNAI1 | Exon13 | frameshift deletion | c.1200delA |
| 3 | DNAI1 | Exon13 | frameshift deletion | c.1203delA |
| 1 | DNAJB1 | Exon1 | frameshift deletion | c.170_171delAC |
| 1 | DNAJB3 | Exon1 | frameshift deletion | c.375delG |
| 3 | DNAJB4 | Exon2 | stopgain SNV | c.215T>A |
| 15 | DNAJB7 | Exon1 | frameshift insertion | c.164dupT |
| 2 | DNAJC10 | Exon13 | frameshift deletion | c.1133delA |
| 2 | DNAJC11 | Exon14 | frameshift deletion | c.1510delA |
| 1 | DNAJC12 | Exon2 | frameshift deletion | c.85delC |
| 1 | DNAJC13 | Exon6 | frameshift insertion | c.431_432insA |
| 2 | DNAJC13 | Exon6 | frameshift deletion | c.448_449delTC |
| 2 | DNAJC14 | Exon2 | stopgain SNV | c.832C>T |
| 1 | DNAJC15 | Exon4 | splicing | c.311+1G>A |
| 1 | DNAJC16 | Exon7 | stopgain SNV | c.1021C>T |
| 4 | DNAJC18 | Exon5 | stopgain SNV | c.606C>G |
| 1 | DNAJC21 | Exon12 | frameshift deletion | c.1494delT |
| 1 | DNAJC28 | Exon2 | stopgain SNV | c.928C>T |
| 1 | DNAJC5 | Exon4 | frameshift deletion | c.478delC |
| 1 | DNASE2B | Exon6 | stopgain SNV | c.1051C>T |
| 1 | DNASE2 | Exon6 | frameshift deletion | c.956_957delAG |
| 1 | DNHD1 | Exon10 | splicing | c.1837+2T>C |
| 1 | DNHD1 | Exon25 | frameshift insertion | c.7644_7645insT |
| 2 | DNHD1 | Exon36 | stopgain SNV | c.12142C>T |
| 1 | DNM2 | Exon18 | frameshift deletion | c.2035delA |
| 1 | DNMBP | Exon4 | frameshift deletion | c.1341_1359del  TCCCCTAGAAGCAAGGACT |
| 1 | DNMT3A | Exon20 | frameshift insertion | c.2357_2360dupCAGC |
| 1 | DNMT3B | Exon9 | frameshift insertion | c.1047dupA |
| 1 | DOCK10 | Exon43 | stopgain SNV | c.4727C>G |
| 1 | DOCK1 | Exon45 | splicing | c.4629+2T>C |
| 3 | DOCK3 | Exon24 | frameshift deletion | c.2440_2441delGT |
| 1 | DOCK5 | Exon17 | frameshift deletion | c.1672delG |
| 1 | DOCK6 | Exon45 | frameshift insertion | c.5755_5756insT |
| 2 | DOCK6 | Exon33 | splicing | c.4022-2dupA |
| 1 | DOCK7 | Exon48 | frameshift deletion | c.6145_6146delAA |
| 1 | DOCK7 | Exon20 | stopgain SNV | c.2263C>T |
| 1 | DOCK8 | Exon12 | frameshift deletion | c.1371_1372delTG |
| 4 | DOCK8 | Exon47 | stopgain SNV | c.6207C>A |
| 1 | DOK1 | Exon5 | stopgain SNV | c.714T>A |
| 1 | DOK5 | Exon7 | frameshift deletion | c.844delT |
| 2 | DOLK | Exon1 | frameshift insertion | c.1dupA |
| 1 | DOM3Z | Exon3 | frameshift deletion | c.544_548delCGGGA |
| 1 | DOM3Z | Exon2 | frameshift deletion | c.61_64delAAAC |
| 1 | DONSON | Exon10 | splicing | c.1563+1G>A |
| 11 | DOPEY2 | Exon30 | frameshift deletion | c.5853delC |
| 2 | DPEP2 | Exon4 | stopgain SNV | c.489T>G |
| 1 | DPP3 | Exon7 | splicing | c.798+1_798+1delG |
| 1 | DPP6 | Exon8 | splicing | c.571-1G>A |
| 1 | DPP6 | Exon19 | frameshift deletion | c.1662delC |
| 3 | DPP7 | Exon13 | frameshift insertion | c.1461dupG |
| 10 | DPP8 | Exon17 | frameshift deletion | c.2136delT |
| 1 | DPP9 | Exon17 | frameshift deletion | c.1961delT |
| 1 | DPP9 | Exon17 | frameshift deletion | c.1959delG |
| 1 | DPP9 | Exon12 | frameshift deletion | c.1225delC |
| 5 | DPP9 | Exon8 | stopgain SNV | c.879G>A |
| 1 | DPP9 | Exon6 | splicing | c.314-1G>A |
| 1 | DPPA2 | Exon6 | frameshift deletion | c.646_647delAT |
| 1 | DPPA2 | Exon4 | frameshift insertion | c.244_245insAATT |
| 1 | DPPA3 | Exon3 | frameshift deletion | c.341_344delCAAC |
| 1 | DPPA4 | Exon3 | frameshift deletion | c.180delG |
| 5 | DPPA4 | Exon4 | splicing | c.179-1_179-1delG |
| 1 | DPRX | Exon3 | stopgain SNV | c.466C>T |
| 1 | DPY19L2 | Exon21 | stopgain SNV | c.2038A>T |
| 1 | DPY19L4 | Exon12 | frameshift deletion | c.1272_1273delCT |
| 1 | DPYD | Exon20 | frameshift deletion | c.2579delA |
| 3 | DPYD | Exon15 | splicing | c.1905+1G>A |
| 1 | DQX1 | Exon10 | stopgain SNV | c.1714C>T |
| 1 | DQX1 | Exon10 | stopgain SNV | c.1635G>A |
| 1 | DQX1 | Exon6 | stopgain SNV | c.1063C>T |
| 1 | DQX1 | Exon4 | stopgain SNV | c.637C>T |
| 1 | DRAM2 | Exon6 | stopgain SNV | c.494G>A |
| 1 | DRD3 | Exon8 | splicing | c.1007-2A>G |
| 2 | DRD5 | Exon1 | stopgain SNV | c.1005C>A |
| 1 | DROSHA | Exon4 | frameshift deletion | c.115_116delCT |
| 6 | DSC2 | Exon16 | frameshift insertion | c.2686_2687dupGA |
| 1 | DSC2 | Exon16 | frameshift deletion | c.2655delT |
| 15 | DSCAML1 | Exon20 | frameshift deletion | c.3883delC |
| 5 | DSCAML1 | Exon20 | frameshift deletion | c.3879delT |
| 1 | DSCAM | Exon17 | frameshift insertion | c.3203dupA |
| 1 | DSCAM | Exon9 | frameshift deletion | c.1899delT |
| 1 | DSCC1 | Exon6 | splicing | c.716+1G>A |
| 2 | DSCR3 | Exon3 | frameshift deletion | c.258delC |
| 2 | DSCR6 | Exon4 | stopgain SNV | c.292C>T |
| 1 | DSEL | Exon2 | frameshift deletion | c.2653delA |
| 1 | DSG4 | Exon16 | frameshift insertion | c.2476dupG |
| 1 | DSN1 | Exon10 | stopgain SNV | c.703C>T |
| 1 | DSP | Exon17 | splicing | c.2436+2T>C |
| 1 | DST | Exon1 | frameshift deletion | c.22delT |
| 4 | DTNBP1 | Exon9 | splicing | c.667+2C>T |
| 1 | DTNBP1 | Exon8 | stopgain SNV | c.664C>T |
| 1 | DTWD1 | Exon3 | stopgain SNV | c.169A>T |
| 1 | DTX3L | Exon3 | stopgain SNV | c.1209T>G |
| 1 | DUOX1 | Exon18 | stopgain SNV | c.1948C>T |
| 1 | DUOX1 | Exon28 | frameshift deletion | c.3552delG |
| 1 | DUOX1 | Exon31 | frameshift insertion | c.3982dupC |
| 1 | DUOX2 | Exon19 | frameshift insertion | c.2406_2407insCTAC |
| 2 | DUOX2 | Exon11 | splicing | c.1041-2A>C |
| 1 | DUOXA1 | Exon6 | frameshift deletion | c.294delT |
| 1 | DUSP11 | Exon1 | frameshift deletion | c.51_52delTT |
| 1 | DUSP12 | Exon6 | frameshift insertion | c.956_957insC |
| 2 | DUSP16 | Exon7 | frameshift deletion | c.1085_1089delGCGTG |
| 2 | DUSP16 | Exon7 | frameshift deletion | c.1080_1083delGCCC |
| 3 | DUSP18 | Exon2 | frameshift deletion | c.491_492delTG |
| 1 | DUSP18 | Exon2 | stopgain SNV | c.40C>T |
| 1 | DUSP1 | Exon4 | frameshift deletion | c.1081delA |
| 1 | DUSP1 | Exon4 | frameshift deletion | c.738_739delCA |
| 1 | DUSP1 | Exon3 | frameshift insertion | c.626_627insAG |
| 2 | DUSP6 | Exon3 | stopgain SNV | c.899C>A |
| 4 | DUXA | Exon6 | frameshift deletion | c.573delC |
| 1 | DVL3 | Exon5 | stopgain SNV | c.502C>T |
| 3 | DYDC2 | Exon4 | splicing | c.148-2A>G |
| 1 | DYDC2 | Exon5 | frameshift deletion | c.368delC |
| 1 | DYNC1H1 | Exon8 | frameshift deletion | c.2093delT |
| 1 | DYNC1I1 | Exon14 | frameshift deletion | c.1556delG |
| 1 | DYNC1LI1 | Exon8 | stopgain SNV | c.1012G>T |
| 2 | DYNC1LI2 | Exon13 | frameshift deletion | c.1379delG |
| 8 | DYNC2H1 | Exon23 | stopgain SNV | c.3381T>A |
| 1 | DYNC2H1 | Exon47 | frameshift deletion | c.7705delT |
| 4 | DYNC2H1 | Exon47 | stopgain SNV | c.7706C>A |
| 4 | DYNC2H1 | Exon49 | stopgain SNV | c.8055T>G |
| 1 | DYNC2H1 | Exon70 | frameshift deletion | c.10527_10528delGT |
| 1 | DYNC2LI1 | Exon6 | frameshift deletion | c.420delA |
| 1 | DYNC2LI1 | Exon7 | splicing | c.508-1G>A |
| 2 | DYRK1A | Exon6 | stopgain SNV | c.340A>T |
| 1 | DYRK2 | Exon3 | frameshift deletion | c.1081delA |
| 2 | DYTN | Exon11 | frameshift insertion | c.1281_1282dupGG |
| 1 | DYX1C1 | Exon5 | stopgain SNV | c.583delA |
| 1 | DZANK1 | Exon19 | splicing | c.1890+1dupG |
| 1 | E2F8 | Exon13 | stopgain SNV | c.2569C>T |
| 1 | EBF4 | Exon3 | frameshift deletion | c.242delT |
| 1 | EBI3 | Exon3 | splicing | c.379+1_379+1delG |
| 10 | EBLN2 | Exon1 | stopgain SNV | c.492T>G |
| 1 | ECD | Exon10 | frameshift deletion | c.1241delT |
| 1 | ECE1 | Exon15 | frameshift insertion | c.1702_1703dupCA |
| 1 | ECEL1 | Exon14 | stopgain SNV | c.1920C>G |
| 1 | ECHDC2 | Exon5 | frameshift insertion | c.399dupG |
| 1 | ECHDC3 | Exon4 | splicing | c.591+2_591+2delT |
| 1 | ECHDC3 | Exon5 | stopgain SNV | c.781C>T |
| 1 | ECM2 | Exon4 | frameshift deletion | c.888_900del  CGGGTGCTCTCTG |
| 1 | ECT2L | Exon16 | splicing | c.2028+1_2028+9del  GTAAATGAG |
| 5 | ECT2L | Exon22 | stopgain SNV | c.2593A>T |
| 1 | ECT2 | Exon8 | frameshift deletion | c.797delG |
| 2 | ECT2 | Exon8 | frameshift deletion | c.798delA |
| 1 | ECT2 | Exon10 | stopgain SNV | c.1039_1040insGTTA |
| 5 | EDA2R | Exon6 | frameshift deletion | c.716delG |
| 1 | EDA2R | Exon6 | frameshift deletion | c.670delT |
| 1 | EDA | Exon2 | frameshift deletion | c.452delG |
| 1 | EDC4 | Exon5 | frameshift deletion | c.508_509delAC |
| 1 | EDC4 | Exon6 | frameshift deletion | c.689_690delAC |
| 1 | EDDM3B | Exon2 | stopgain SNV | c.268C>T |
| 1 | EDEM1 | Exon10 | splicing | c.1680+1G>C |
| 1 | EDN1 | Exon2 | frameshift deletion | c.76delG |
| 1 | EDN3 | Exon2 | stopgain SNV | c.306C>G |
| 1 | EDN3 | Exon3 | stopgain SNV | c.400G>T |
| 1 | EDNRB | Exon2 | frameshift insertion | c.360_361insGA |
| 1 | EEF2K | Exon6 | stopgain SNV | c.616C>T |
| 1 | EEF2K | Exon16 | stopgain SNV | c.1852C>T |
| 1 | EEF2 | Exon2 | frameshift deletion | c.209delT |
| 2 | EFCAB3 | Exon6 | stopgain SNV | c.451C>T |
| 2 | EFCAB5 | Exon1 | splicing | c.42+1G>A |
| 1 | EFCAB5 | Exon10 | stopgain SNV | c.1852C>T |
| 1 | EFCAB5 | Exon12 | splicing | c.2273-2_2273-1insT |
| 8 | EFCAB6 | Exon27 | frameshift deletion | c.3563_3564delAG |
| 1 | EFCAB6 | Exon25 | splicing | c.2773-2A>G |
| 1 | EFCAB7 | Exon4 | frameshift deletion | c.474delT |
| 1 | EFCAB7 | Exon12 | stopgain SNV | c.1528G>T |
| 2 | EFEMP2 | Exon5 | frameshift insertion | c.468dupC |
| 3 | EFHA1 | Exon9 | stopgain SNV | c.916A>T |
| 1 | EFHA1 | Exon1 | frameshift insertion | c.190dupA |
| 1 | EFHB | Exon5 | frameshift deletion | c.1287delA |
| 1 | EFHC1 | Exon4 | frameshift deletion | c.682_692delGACTTTGATCA |
| 18 | EFNB1 | Exon2 | frameshift deletion | c.251delA |
| 1 | EFR3A | Exon4 | frameshift deletion | c.310delG |
| 1 | EFR3A | Exon6 | frameshift deletion | c.489delG |
| 4 | EGFL8 | Exon3 | stopgain SNV | c.222C>G |
| 5 | EGF | Exon23 | frameshift insertion | c.3280dupC |
| 1 | EGFR | Exon3 | frameshift deletion | c.287delT |
| 1 | EGFR | Exon21 | frameshift deletion | c.2509delG |
| 1 | EGFR | Exon27 | splicing | c.3163-1_3163-1delG |
| 1 | EGFR | Exon27 | frameshift deletion | c.3165delG |
| 3 | EGLN2 | Exon4 | frameshift insertion | c.990_991insCT |
| 1 | EHBP1L1 | Exon14 | frameshift deletion | c.4036delA |
| 1 | EHD3 | Exon2 | frameshift deletion | c.271delG |
| 1 | EHD3 | Exon4 | frameshift deletion | c.679delC |
| 1 | EHD4 | Exon4 | frameshift deletion | c.690delG |
| 1 | EIF2AK3 | Exon17 | frameshift deletion | c.3268_3271delCAGA |
| 1 | EIF2AK4 | Exon9 | frameshift deletion | c.1532delA |
| 4 | EIF2AK4 | Exon34 | splicing | c.4561+2T>G |
| 4 | EIF2A | Exon8 | frameshift deletion | c.573_574delTA |
| 2 | EIF2B2 | Exon2 | splicing | c.284+2T>G |
| 2 | EIF2B3 | Exon4 | frameshift deletion | c.450delA |
| 1 | EIF2B5 | Exon4 | frameshift insertion | c.602dupT |
| 11 | EIF2B5 | Exon16 | stoploss SNV | c.2164T>A |
| 1 | EIF2D | Exon11 | splicing | c.921-2A>G |
| 8 | EIF3I | Exon11 | stopgain SNV | c.939C>G |
| 1 | EIF3L | Exon7 | frameshift deletion | c.761_762delAG |
| 1 | EIF4E1B | Exon8 | splicing | c.614+1G>T |
| 1 | ELAC2 | Exon15 | frameshift insertion | c.1334_1335insC |
| 1 | ELAC2 | Exon8 | splicing | c.560-2A>G |
| 1 | ELFN2 | Exon3 | frameshift deletion | c.1365delC |
| 1 | ELFN2 | Exon3 | frameshift insertion | c.1357_1358insGGAG |
| 1 | ELMO3 | Exon10 | frameshift deletion | c.984delC |
| 1 | ELMO3 | Exon10 | frameshift deletion | c.991delA |
| 1 | ELMO3 | Exon13 | stopgain SNV | c.1342A>T |
| 1 | ELMOD2 | Exon2 | frameshift insertion | c.1dupA |
| 1 | ELN | Exon26 | frameshift deletion | c.1776delA |
| 1 | EME1 | Exon2 | frameshift insertion | c.735dupA |
| 1 | EME1 | Exon8 | stopgain SNV | c.1484G>A |
| 8 | EME1 | Exon8 | stopgain SNV | c.1510C>T |
| 1 | EMILIN2 | Exon4 | stopgain SNV | c.553C>T |
| 1 | EMILIN2 | Exon8 | frameshift deletion | c.2887_2888delGA |
| 1 | EML3 | Exon10 | frameshift deletion | c.1175delG |
| 3 | EML5 | Exon17 | frameshift deletion | c.2493delA |
| 4 | EML6 | Exon25 | stopgain SNV | c.3516G>A |
| 1 | EMR1 | Exon5 | stopgain SNV | c.430G>T |
| 7 | ENAH | Exon6 | splicing | c.435-1G>C |
| 1 | ENAM | Exon9 | frameshift deletion | c.2080_2084delAATCA |
| 1 | ENC1 | Exon2 | frameshift deletion | c.1409delA |
| 1 | ENDOD1 | Exon2 | stopgain SNV | c.1273C>T |
| 1 | ENDOU | Exon10 | splicing | c.1115+1G>C |
| 1 | ENDOU | Exon8 | frameshift insertion | c.879dupA |
| 8 | ENDOV | Exon4 | splicing | c.364-2A>G |
| 4 | ENGASE | Exon4 | frameshift deletion | c.513delT |
| 3 | ENGASE | Exon4 | frameshift insertion | c.516_517insT |
| 1 | ENO3 | Exon9 | frameshift insertion | c.1065dupC |
| 1 | ENOSF1 | Exon16 | frameshift insertion | c.1279dupC |
| 1 | ENOSF1 | Exon15 | stopgain SNV | c.1195delG |
| 1 | ENOSF1 | Exon13 | stopgain SNV | c.990C>A |
| 7 | ENPEP | Exon6 | stopgain SNV | c.1238G>A |
| 1 | ENPEP | Exon9 | frameshift deletion | c.1558delT |
| 1 | ENPEP | Exon16 | stopgain SNV | c.2282T>A |
| 1 | ENPP3 | Exon2 | stopgain SNV | c.116T>G |
| 1 | ENPP7 | Exon2 | splicing | c.399+2T>C |
| 4 | ENTHD1 | Exon6 | frameshift deletion | c.1104_1105delTC |
| 2 | ENTPD2 | Exon7 | splicing | c.1029+2T>C |
| 1 | ENTPD7 | Exon10 | frameshift deletion | c.1311delG |
| 1 | EP300 | Exon13 | splicing | c.2242-2_2242-1delAG |
| 1 | EP300 | Exon13 | splicing | c.2242-1_2242-1delG |
| 1 | EP400 | Exon30 | frameshift insertion | c.5924_5925insT |
| 4 | EPB41L1 | Exon6 | stopgain SNV | c.397C>T |
| 1 | EPB41L3 | Exon22 | stopgain SNV | c.3256G>T |
| 1 | EPB41L3 | Exon12 | frameshift deletion | c.1354delC |
| 15 | EPB41L5 | Exon7 | stopgain SNV | c.502C>T |
| 1 | EPG5 | Exon37 | stopgain SNV | c.6314G>A |
| 1 | EPG5 | Exon30 | frameshift deletion | c.5230delA |
| 1 | EPG5 | Exon14 | frameshift deletion | c.2691delA |
| 1 | EPHA10 | Exon17 | stopgain SNV | c.2989C>T |
| 1 | EPHA10 | Exon13 | splicing | c.2208+1G>A |
| 1 | EPHA1 | Exon17 | splicing | c.2696+2T>A |
| 1 | EPHA2 | Exon13 | frameshift deletion | c.2263delG |
| 1 | EPHA5 | Exon2 | stopgain SNV | c.224G>A |
| 20 | EPHB1 | Exon3 | frameshift deletion | c.614delT |
| 1 | EPHB2 | Exon11 | frameshift deletion | c.2090delT |
| 1 | EPHX2 | Exon16 | stopgain SNV | c.1399C>T |
| 1 | EPHX3 | Exon7 | frameshift insertion | c.807dupA |
| 1 | EPHX3 | Exon4 | frameshift deletion | c.439delA |
| 2 | EPN3 | Exon7 | frameshift deletion | c.1189_1202del  AAACTCCCCAGCAC |
| 1 | EPN3 | Exon9 | frameshift deletion | c.1568_1575delGGAACCCC |
| 1 | EPO | Exon5 | frameshift deletion | c.454_455delGG |
| 1 | EPOR | Exon3 | splicing | c.251+1G>A |
| 2 | EPRS | Exon24 | stopgain SNV | c.3394A>T |
| 1 | EPS8L3 | Exon15 | frameshift deletion | c.1263_1266delAGCC |
| 1 | EPS8L3 | Exon7 | splicing | c.461+1G>A |
| 2 | EPSTI1 | Exon7 | stopgain SNV | c.564C>G |
| 1 | EPT1 | Exon4 | frameshift deletion | c.261delC |
| 1 | EPX | Exon6 | stopgain SNV | c.658C>T |
| 13 | EPX | Exon6 | stopgain SNV | c.771C>A |
| 2 | EPX | Exon6 | splicing | c.801+1G>A |
| 1 | ERAP1 | Exon13 | frameshift deletion | c.1861_1864delACTG |
| 1 | ERAP2 | Exon9 | splicing | c.1503+1G>A |
| 1 | ERBB3 | Exon24 | stopgain SNV | c.2843G>A |
| 1 | ERBB3 | Exon28 | frameshift deletion | c.4029_4305del |
| 7 | ERC1 | Exon9 | stopgain SNV | c.1792G>T |
| 1 | ERC2 | Exon4 | frameshift insertion | c.1077dupA |
| 1 | ERCC2 | Exon18 | frameshift deletion | c.1703_1704delTT |
| 2 | ERCC3 | Exon3 | stopgain SNV | c.325C>T |
| 1 | ERG | Exon11 | splicing | c.892+1G>T |
| 1 | ERICH1 | Exon5 | frameshift deletion | c.1218delT |
| 1 | ERICH1 | Exon3 | frameshift insertion | c.258_259insCC |
| 1 | ERLEC1 | Exon4 | frameshift deletion | c.389_390delAC |
| 1 | ERMAP | Exon5 | stopgain SNV | c.476_483dupCTGTGATC |
| 1 | ERMP1 | Exon14 | splicing | c.2386+1G>T |
| 2 | ERMP1 | Exon8 | frameshift insertion | c.1335dupC |
| 1 | ERMP1 | Exon7 | frameshift deletion | c.1297_1298delGG |
| 1 | ERN2 | Exon17 | splicing | c.1945-1G>A |
| 1 | ERO1LB | Exon16 | frameshift insertion | c.1385dupT |
| 1 | ERV3-1 | Exon2 | stopgain SNV | c.1279C>T |
| 1 | ERV3-1 | Exon2 | frameshift deletion | c.848_849delTA |
| 1 | ERVMER34-1 | Exon3 | stopgain SNV | c.1637C>G |
| 7 | ESF1 | Exon14 | frameshift deletion | c.2519_2520delAG |
| 2 | ESR2 | Exon3 | splicing | c.362+2T>A |
| 1 | ESRP2 | Exon10 | frameshift insertion | c.1287dupC |
| 1 | ESX1 | Exon4 | frameshift deletion | c.1171delA |
| 1 | ESX1 | Exon2 | frameshift deletion | c.282_285delGGAG |
| 1 | ESYT1 | Exon17 | frameshift insertion | c.1863dupT |
| 1 | ESYT1 | Exon29 | splicing | c.3225+1G>A |
| 1 | ETNK2 | Exon3 | frameshift deletion | c.566_567delGC |
| 1 | ETV1 | Exon7 | frameshift insertion | c.360dupC |
| 1 | EVPL | Exon22 | frameshift insertion | c.2963_2964insT |
| 1 | EWSR1 | Exon13 | frameshift deletion | c.1380_1381delTG |
| 1 | EXO1 | Exon14 | stopgain SNV | c.2152C>T |
| 1 | EXO1 | Exon15 | splicing | c.2212-1G>C |
| 1 | EXOC4 | Exon13 | splicing | c.1872-2A>T |
| 1 | EXOC8 | Exon1 | stopgain SNV | c.2042T>G |
| 1 | EXOG | Exon3 | splicing | c.304-1G>A |
| 2 | EXOG | Exon5 | frameshift deletion | c.608delA |
| 1 | EXOSC9 | Exon7 | frameshift deletion | c.656delG |
| 2 | EXTL3 | Exon3 | stopgain SNV | c.925A>T |
| 1 | EYA4 | Exon6 | stopgain SNV | c.321C>A |
| 3 | EZH2 | Exon17 | stopgain SNV | c.1975A>T |
| 1 | EZH2 | Exon18 | splicing | c.1898-1G>A |
| 1 | EZH2 | Exon6 | frameshift deletion | c._del |
| 1 | EZH2 | Exon3 | frameshift deletion | c.119delG |
| 1 | EZH2 | Exon4 | splicing | c.118-2_118-2delA |
| 1 | F11 | Exon5 | stopgain SNV | c.403G>T |
| 1 | F13A1 | Exon7 | splicing | c.691-1G>A |
| 1 | F2 | Exon8 | splicing | c.1003+2T>G |
| 1 | F2RL1 | Exon1 | splicing | c.82+1G>A |
| 1 | F5 | Exon15 | frameshift insertion | c.5037dupA |
| 1 | F7 | Exon1 | frameshift deletion | c.23delT |
| 1 | F7 | Exon8 | frameshift deletion | c.1196delT |
| 2 | FAAH2 | Exon10 | stopgain SNV | c.1294G>T |
| 1 | FAAH | Exon2 | frameshift deletion | c.226delG |
| 1 | FABP2 | Exon4 | splicing | c.241-10_241-5delTATTAT |
| 1 | FABP4 | Exon4 | splicing | c.348+2T>C |
| 1 | FABP5 | Exon3 | frameshift deletion | c.255delT |
| 1 | FAHD2B | Exon8 | stopgain SNV | c.913G>T |
| 1 | FAH | Exon9 | frameshift deletion | c.744delG |
| 1 | FAIM2 | Exon2 | frameshift deletion | c.57_58delGC |
| 1 | FAIM3 | Exon3 | frameshift insertion | c.317_318insA |
| 17 | FAM101B | Exon2 | stopgain SNV | c.253C>T |
| 1 | FAM102B | Exon11 | frameshift insertion | c.1083dupG |
| 1 | FAM108A1 | Exon3 | frameshift deletion | c.363_364delCC |
| 2 | FAM111B | Exon4 | frameshift insertion | c.816dupA |
| 1 | FAM111B | Exon4 | stopgain SNV | c.1515G>A |
| 1 | FAM114A1 | Exon4 | frameshift deletion | c.392delG |
| 1 | FAM114A1 | Exon12 | stopgain SNV | c.1360delG |
| 7 | FAM115C | Exon3 | frameshift deletion | c.1256_1257delCT |
| 1 | FAM117A | Exon7 | frameshift deletion | c.1029delC |
| 1 | FAM118B | Exon4 | frameshift deletion | c.266delA |
| 1 | FAM123B | Exon2 | frameshift deletion | c.3191delC |
| 1 | FAM124A | Exon3 | stopgain SNV | c.175C>T |
| 18 | FAM124B | Exon3 | splicing | c.733-1G>A |
| 1 | FAM126A | Exon6 | stopgain SNV | c.523C>T |
| 5 | FAM129A | Exon9 | splicing | c.823-1G>C |
| 1 | FAM129A | Exon6 | splicing | c.434-1G>T |
| 1 | FAM129C | Exon11 | frameshift deletion | c.1359_1371del  AGCACCGTTTGGC |
| 1 | FAM134A | Exon2 | splicing | c.282-1_282-0insC |
| 1 | FAM134C | Exon9 | frameshift insertion | c.1236_1237insT |
| 1 | FAM13A | Exon4 | stopgain SNV | c.498C>A |
| 1 | FAM13A | Exon5 | splicing | c.428-2A>G |
| 2 | FAM13C | Exon12 | frameshift deletion | c.1013delT |
| 1 | FAM151A | Exon8 | stopgain SNV | c.1693C>T |
| 1 | FAM151A | Exon8 | frameshift insertion | c.1146dupG |
| 4 | FAM151A | Exon7 | frameshift deletion | c.1003_1031del  CCTGGGGATGACGGT  CTGAATGTGGAGTG |
| 2 | FAM151A | Exon6 | stopgain SNV | c.818G>A |
| 1 | FAM151A | Exon2 | splicing | c.118+1G>C |
| 1 | FAM154A | Exon4 | stopgain SNV | c.823C>T |
| 1 | FAM154A | Exon4 | stopgain SNV | c.538A>T |
| 1 | FAM154B | Exon3 | frameshift insertion | c.317dupC |
| 6 | FAM154B | Exon3 | frameshift deletion | c.528_529delAC |
| 1 | FAM155A | Exon1 | stopgain SNV | c.97G>T |
| 1 | FAM159A | Exon2 | frameshift insertion | c.295_296insTT |
| 1 | FAM159A | Exon2 | frameshift insertion | c.300_301insA |
| 2 | FAM159A | Exon2 | stopgain SNV | c.313C>T |
| 1 | FAM160A2 | Exon12 | frameshift insertion | c.2934_2935insCA |
| 1 | FAM160A2 | Exon12 | frameshift deletion | c.2887delT |
| 1 | FAM160A2 | Exon3 | stopgain SNV | c.336G>A |
| 1 | FAM160B1 | Exon9 | frameshift insertion | c.1146dupT |
| 4 | FAM160B2 | Exon7 | frameshift insertion | c.887dupA |
| 1 | FAM161A | Exon3 | stopgain SNV | c.493C>T |
| 2 | FAM161B | Exon9 | frameshift deletion | c.2119delG |
| 1 | FAM162A | Exon3 | splicing | c.263+1G>A |
| 1 | FAM164C | Exon2 | frameshift insertion | c.46dupA |
| 1 | FAM166A | Exon7 | frameshift deletion | c.886_887delCT |
| 1 | FAM166A | Exon5 | stopgain SNV | c.703C>T |
| 1 | FAM168B | Exon5 | stopgain SNV | c.316C>T |
| 1 | FAM171A1 | Exon8 | frameshift deletion | c.1090_1091delTT |
| 1 | FAM173B | Exon5 | stopgain SNV | c.544C>T |
| 1 | FAM173B | Exon3 | frameshift deletion | c.347delA |
| 8 | FAM174A | Exon2 | stopgain SNV | c.565C>T |
| 1 | FAM175B | Exon5 | frameshift insertion | c.338dupA |
| 1 | FAM177B | Exon4 | stopgain SNV | c.189G>A |
| 1 | FAM178A | Exon5 | stopgain SNV | c.1369C>T |
| 1 | FAM178A | Exon11 | stopgain SNV | c.2543_2544insA |
| 1 | FAM179A | Exon4 | frameshift deletion | c.231_240delGGACACCCCT |
| 1 | FAM179A | Exon9 | stopgain SNV | c.1192G>T |
| 1 | FAM179A | Exon14 | splicing | c.2012+2T>C |
| 1 | FAM179A | Exon19 | stopgain SNV | c.2668C>T |
| 9 | FAM179B | Exon9 | stopgain SNV | c.3410C>A |
| 1 | FAM180A | Exon3 | frameshift deletion | c.249delG |
| 2 | FAM183A | Exon4 | stopgain SNV | c.377G>A |
| 16 | FAM187B | Exon1 | stopgain SNV | c.693G>A |
| 1 | FAM187B | Exon1 | stopgain SNV | c.94C>T |
| 1 | FAM188B | Exon3 | stopgain SNV | c.307A>T |
| 2 | FAM194A | Exon12 | frameshift deletion | c.1371delT |
| 1 | FAM194A | Exon1 | frameshift insertion | c.158_159insC |
| 13 | FAM194B | Exon3 | frameshift deletion | c.404delA |
| 1 | FAM205A | Exon4 | frameshift insertion | c.490dupT |
| 1 | FAM207A | Exon2 | frameshift deletion | c.208delG |
| 1 | FAM208A | Exon23 | frameshift deletion | c.4323delG |
| 1 | FAM208A | Exon23 | frameshift deletion | c._del |
| 2 | FAM208B | Exon15 | stopgain SNV | c.3941T>G |
| 1 | FAM21C | Exon3 | frameshift deletion | c.267_270delCTCT |
| 1 | FAM3B | Exon7 | frameshift deletion | c.582delA |
| 1 | FAM3C | Exon10 | stopgain SNV | c.658G>T |
| 1 | FAM40B | Exon21 | frameshift insertion | c.2455_2456dupGA |
| 1 | FAM47A | Exon1 | frameshift deletion | c.375_376delAA |
| 1 | FAM47C | Exon1 | stopgain SNV | c.1920C>G |
| 1 | FAM47E | Exon3 | splicing | c.221+1G>A |
| 1 | FAM48B1 | Exon1 | frameshift insertion | c.2019_2020insAA |
| 1 | FAM48B2 | Exon1 | frameshift deletion | c.1376_1377delAA |
| 1 | FAM58A | Exon4 | stopgain SNV | c.625C>T |
| 1 | FAM58BP | Exon1 | stopgain SNV | c.22G>T |
| 10 | FAM59A | Exon6 | frameshift deletion | c.2013delT |
| 1 | FAM5C | Exon3 | frameshift deletion | c.409delC |
| 1 | FAM63A | Exon10 | frameshift insertion | c.1436_1437insT |
| 3 | FAM63B | Exon4 | frameshift deletion | c.998delT |
| 1 | FAM65B | Exon14 | frameshift deletion | c.1770delT |
| 1 | FAM70B | Exon5 | stopgain SNV | c.373C>T |
| 6 | FAM71A | Exon1 | stopgain SNV | c.1663A>T |
| 1 | FAM71C | Exon1 | frameshift insertion | c.234dupC |
| 15 | FAM71D | Exon5 | frameshift insertion | c.588_589insT |
| 1 | FAM71F2 | Exon3 | stopgain SNV | c.384C>G |
| 1 | FAM73A | Exon8 | splicing | c.896-1G>A |
| 1 | FAM73A | Exon16 | stopgain SNV | c.1723A>T |
| 1 | FAM75A3 | Exon4 | frameshift deletion | c.2963delG |
| 1 | FAM75A3 | Exon4 | frameshift insertion | c.4021dupT |
| 1 | FAM75C1 | Exon4 | frameshift deletion | c.3402_3403delCA |
| 1 | FAM75C2 | Exon4 | stopgain SNV | c.3004C>T |
| 1 | FAM75C2 | Exon3 | splicing | c.265+1G>A |
| 1 | FAM75D1 | Exon4 | frameshift deletion | c.1958delC |
| 1 | FAM78A | Exon1 | frameshift deletion | c.276_277delGG |
| 1 | FAM81B | Exon1 | frameshift deletion | c.92delA |
| 1 | FAM81B | Exon5 | stopgain SNV | c.640C>T |
| 3 | FAM81B | Exon7 | frameshift insertion | c.854_855insC |
| 1 | FAM81B | Exon8 | stopgain SNV | c.948G>A |
| 3 | FAM82A1 | Exon2 | stopgain SNV | c.46C>T |
| 2 | FAM82A1 | Exon2 | splicing | c.986+1G>A |
| 1 | FAM82A1 | Exon5 | splicing | c.1265-2A>C |
| 1 | FAM82A2 | Exon8 | splicing | c.911-2A>C |
| 3 | FAM83A | Exon1 | stopgain SNV | c.256G>T |
| 1 | FAM83A | Exon1 | frameshift deletion | c.366delC |
| 1 | FAM83D | Exon4 | frameshift deletion | c.1750_1753delCTCT |
| 2 | FAM83E | Exon4 | frameshift insertion | c.889dupC |
| 1 | FAM83H | Exon3 | stopgain SNV | c.601C>T |
| 1 | FAM86B1 | Exon7 | frameshift deletion | c.801delG |
| 1 | FAM86B2 | Exon8 | frameshift deletion | c.903delG |
| 1 | FAM90A1 | Exon4 | frameshift deletion | c.61delC |
| 2 | FAM90A1 | Exon4 | frameshift deletion | c.10delC |
| 1 | FAM98C | Exon8 | frameshift insertion | c.950dupG |
| 2 | FAM9C | Exon5 | stopgain SNV | c.319C>T |
| 1 | FAN1 | Exon13 | stopgain SNV | c.2854C>T |
| 1 | FANCA | Exon40 | splicing | c.3934+2T>C |
| 1 | FANCA | Exon4 | splicing | c.190-1G>C |
| 1 | FANCD2 | Exon28 | splicing | c.2715+1G>A |
| 2 | FANCD2 | Exon30 | stopgain SNV | c.2920C>T |
| 1 | FANCD2 | Exon32 | frameshift deletion | c.3185_3188delATCA |
| 18 | FANCD2 | Exon43 | frameshift deletion | c.4240delG |
| 3 | FANCD2 | Exon43 | frameshift deletion | c.4251delA |
| 1 | FANCE | Exon2 | frameshift deletion | c.387delA |
| 1 | FANCI | Exon23 | frameshift deletion | c.2345_2346delCT |
| 4 | FANCL | Exon14 | frameshift insertion | c.1096_1099dupATTA |
| 1 | FANCM | Exon22 | stopgain SNV | c.5791C>T |
| 1 | FARP2 | Exon2 | stopgain SNV | c.61C>T |
| 1 | FARP2 | Exon16 | frameshift deletion | c.1720delA |
| 1 | FARSB | Exon3 | stopgain SNV | c.215dupA |
| 2 | FASLG | Exon2 | frameshift deletion | c.373delT |
| 9 | FASTKD1 | Exon14 | frameshift insertion | c.2406dupA |
| 5 | FASTKD1 | Exon13 | stopgain SNV | c.2303C>G |
| 1 | FASTKD3 | Exon2 | frameshift insertion | c.457_458insC |
| 1 | FAT3 | Exon9 | frameshift insertion | c.6569_6570insA |
| 1 | FAT4 | Exon5 | frameshift deletion | c.5941delT |
| 1 | FBF1 | Exon22 | frameshift deletion | c.2457_2458delGC |
| 1 | FBLIM1 | Exon6 | frameshift insertion | c.595_596insA |
| 1 | FBLN1 | Exon10 | frameshift insertion | c.1080_1081insCA |
| 7 | FBLN2 | Exon2 | frameshift deletion | c.939delC |
| 1 | FBLN2 | Exon16 | stopgain SNV | c.3163C>T |
| 2 | FBLN2 | Exon17 | stoploss SNV | c.3376T>C |
| 1 | FBLN7 | Exon7 | frameshift deletion | c.1162delG |
| 3 | FBN1 | Exon35 | stopgain SNV | c.4245T>A |
| 3 | FBN2 | Exon66 | splicing | c.8365-1G>A |
| 5 | FBP2 | Exon7 | stoploss SNV | c.1019A>G |
| 1 | FBXL13 | Exon3 | frameshift deletion | c.38delA |
| 13 | FBXL21 | Exon8 | frameshift deletion | c.1190_1191delCT |
| 1 | FBXO10 | Exon11 | frameshift deletion | c.2781delG |
| 1 | FBXO10 | Exon4 | frameshift deletion | c.1497_1498delTG |
| 1 | FBXO22 | Exon7 | stoploss SNV | c.1212A>T |
| 1 | FBXO24 | Exon6 | stopgain SNV | c.820delG |
| 1 | FBXO28 | Exon4 | stopgain SNV | c.592C>T |
| 1 | FBXO38 | Exon11 | frameshift deletion | c.1290delT |
| 1 | FBXO3 | Exon9 | stopgain SNV | c.1024G>T |
| 1 | FBXW10 | Exon1 | frameshift deletion | c.293_294delAG |
| 1 | FBXW10 | Exon6 | frameshift deletion | c.1224_1227delCTCT |
| 1 | FBXW10 | Exon14 | frameshift deletion | c.2898_2899delAA |
| 2 | FBXW4 | Exon8 | stopgain SNV | c.1023C>G |
| 2 | FBXW8 | Exon8 | frameshift deletion | c.1312_1313delGT |
| 1 | FCER2 | Exon9 | frameshift deletion | c.489_493delCCCTG |
| 2 | FCER2 | Exon8 | splicing | c.317-1G>C |
| 1 | FCGBP | Exon28 | stopgain SNV | c.12851C>A |
| 1 | FCGBP | Exon10 | frameshift deletion | c.4846_4847delGG |
| 1 | FCGBP | Exon8 | splicing | c.3990+1G>A |
| 1 | FCGBP | Exon2 | frameshift deletion | c.887_888delAG |
| 4 | FCGR1A | Exon3 | stopgain SNV | c.274C>T |
| 1 | FCGR1B | Exon5 | frameshift insertion | c.583dupG |
| 2 | FCGR1B | Exon3 | frameshift deletion | c.168_169delGT |
| 1 | FCGR2C | Exon3 | stoploss SNV | c.169T>C |
| 1 | FCGR3A | Exon4 | frameshift deletion | c.160delG |
| 1 | FCHSD1 | Exon14 | frameshift deletion | c.1333_1336delGACT |
| 1 | FCHSD1 | Exon11 | stopgain SNV | c.1003C>T |
| 12 | FCHSD2 | Exon20 | stoploss SNV | c.2223A>G |
| 1 | FCHSD2 | Exon11 | frameshift insertion | c.1009_1010insTC |
| 1 | FCHSD2 | Exon11 | frameshift insertion | c.1008_1009dupAC |
| 2 | FCHSD2 | Exon2 | frameshift deletion | c.114delT |
| 2 | FCN2 | Exon8 | frameshift deletion | c.791delC |
| 8 | FCN3 | Exon4 | frameshift deletion | c.316delC |
| 2 | FCN3 | Exon4 | splicing | c.232+1G>A |
| 1 | FCRL1 | Exon4 | stopgain SNV | c.481C>T |
| 1 | FCRL2 | Exon3 | frameshift deletion | c.80_81delCT |
| 2 | FCRL3 | Exon5 | stopgain SNV | c.361C>T |
| 1 | FCRL5 | Exon11 | frameshift deletion | c.2502_2503delAC |
| 1 | FDFT1 | Exon4 | stopgain SNV | c.496C>T |
| 1 | FECH | Exon8 | frameshift insertion | c.825_826insAA |
| 1 | FECH | Exon4 | splicing | c.314+2T>C |
| 1 | FER1L5 | Exon10 | splicing | c.843+1G>C |
| 11 | FERMT1 | Exon12 | frameshift deletion | c.1577delG |
| 4 | FERMT1 | Exon12 | frameshift insertion | c.1574_1575insG |
| 3 | FERMT1 | Exon6 | frameshift deletion | c.795delT |
| 1 | FETUB | Exon1 | frameshift deletion | c.188_189delGA |
| 1 | FETUB | Exon4 | frameshift deletion | c.437delA |
| 1 | FETUB | Exon7 | frameshift insertion | c.872dupC |
| 1 | FGD1 | Exon4 | frameshift insertion | c.832_833insC |
| 1 | FGD1 | Exon3 | frameshift insertion | c.596dupC |
| 1 | FGD1 | Exon3 | frameshift insertion | c.527dupC |
| 5 | FGD6 | Exon2 | stopgain SNV | c.2419G>T |
| 1 | FGD6 | Exon2 | frameshift deletion | c.2420delG |
| 1 | FGD6 | Exon2 | frameshift deletion | c.2416delC |
| 3 | FGF19 | Exon3 | stopgain SNV | c.360T>A |
| 1 | FGF6 | Exon2 | frameshift deletion | c.353delT |
| 1 | FGGY | Exon4 | frameshift insertion | c.418dupG |
| 2 | FGGY | Exon11 | splicing | c.1221+2T>C |
| 1 | FGGY | Exon15 | splicing | c.1490-1G>C |
| 1 | FGL1 | Exon10 | splicing | c.780-1G>C |
| 1 | FHDC1 | Exon9 | frameshift deletion | c.1204delG |
| 2 | FH | Exon5 | frameshift deletion | c.691delA |
| 1 | FHOD1 | Exon13 | frameshift insertion | c.1754dupC |
| 11 | FIG4 | Exon16 | stopgain SNV | c.1824G>A |
| 1 | FIGNL1 | Exon4 | frameshift deletion | c.1519_1523delTCTCA |
| 1 | FIGNL1 | Exon4 | frameshift insertion | c.605_606insG |
| 1 | FKBP14 | Exon4 | stoploss SNV | c.634T>C |
| 1 | FKBP14 | Exon3 | frameshift insertion | c.362dupC |
| 1 | FKBP15 | Exon26 | frameshift deletion | c.3183delA |
| 1 | FKBP2 | Exon2 | frameshift deletion | c.67delA |
| 4 | FKTN | Exon5 | stopgain SNV | c.351T>G |
| 1 | FKTN | Exon7 | frameshift deletion | c.773_774delTT |
| 1 | FLG2 | Exon3 | frameshift deletion | c.6852_6855delACAG |
| 1 | FLG2 | Exon3 | frameshift deletion | c.5722delC |
| 1 | FLG2 | Exon3 | stopgain SNV | c.4687C>T |
| 2 | FLG2 | Exon3 | frameshift insertion | c.3939_3940dupTA |
| 2 | FLG2 | Exon3 | frameshift deletion | c.3101_3104delAATA |
| 1 | FLG | Exon3 | stopgain SNV | c.10828C>T |
| 1 | FLG | Exon3 | stopgain SNV | c.10255C>T |
| 1 | FLG | Exon3 | stopgain SNV | c.9947C>G |
| 1 | FLG | Exon3 | stopgain SNV | c.7661C>G |
| 2 | FLG | Exon3 | stopgain SNV | c.7339C>T |
| 9 | FLG | Exon3 | frameshift deletion | c.2282_2285delCAGT |
| 8 | FLG | Exon3 | stopgain SNV | c.1501C>T |
| 1 | FLG | Exon3 | stopgain SNV | c.1123C>T |
| 1 | FLI1 | Exon10 | frameshift deletion | c.1119_1122delCTCC |
| 1 | FLII | Exon30 | frameshift insertion | c.3796_3800dupAAGGC |
| 1 | FLII | Exon24 | stopgain SNV | c.3154C>T |
| 1 | FLNA | Exon47 | frameshift insertion | c.7906dupG |
| 1 | FLNA | Exon46 | frameshift deletion | c.7543delA |
| 1 | FLNA | Exon46 | frameshift deletion | c.7535_7536delGT |
| 3 | FLNA | Exon9 | splicing | c.1228+2T>G |
| 2 | FLNB | Exon29 | frameshift deletion | c.4973delC |
| 1 | FLNC | Exon38 | frameshift deletion | c.6351delG |
| 1 | FLNC | Exon38 | frameshift deletion | c.6355delG |
| 1 | FLOT2 | Exon6 | frameshift deletion | c.508_509delAC |
| 1 | FLOT2 | Exon5 | frameshift deletion | c.410delT |
| 2 | FLRT2 | Exon2 | frameshift deletion | c.1512delA |
| 1 | FLT3LG | Exon6 | frameshift insertion | c.351dupC |
| 1 | FLT3 | Exon14 | splicing | c.1704+1_1704+1delG |
| 7 | FMNL2 | Exon21 | stopgain SNV | c.2661T>G |
| 1 | FMO2 | Exon6 | stopgain SNV | c.745C>T |
| 4 | FMO2 | Exon9 | stoploss SNV | c.1414T>C |
| 1 | FMO4 | Exon10 | frameshift deletion | c.1495delA |
| 1 | FMO4 | Exon10 | frameshift deletion | c.1551delC |
| 1 | FMO5 | Exon5 | stopgain SNV | c.531T>G |
| 2 | FNDC3B | Exon17 | frameshift deletion | c.1914delA |
| 1 | FNDC4 | Exon2 | frameshift insertion | c.30dupC |
| 2 | FNDC7 | Exon7 | stopgain SNV | c.1279G>T |
| 1 | FNDC9 | Exon2 | frameshift deletion | c.532delC |
| 4 | FOLR1 | Exon5 | splicing | c.493+2T>C |
| 2 | FOLR3 | Exon5 | stopgain SNV | c.595C>T |
| 1 | FOLR4 | Exon2 | splicing | c.139-2A>G |
| 1 | FOXB2 | Exon1 | frameshift deletion | c.188_198delACAACCTCTCC |
| 1 | FOXC2 | Exon1 | frameshift insertion | c.438dupG |
| 1 | FOXD4L5 | Exon1 | frameshift insertion | c.815dupT |
| 2 | FOXD4 | Exon1 | frameshift insertion | c.1271dupT |
| 2 | FOXD4 | Exon1 | frameshift insertion | c.756dupG |
| 1 | FOXRED2 | Exon8 | frameshift deletion | c.1721delC |
| 1 | FOXRED2 | Exon5 | frameshift deletion | c.1098_1099delGA |
| 1 | FRA10AC1 | Exon13 | splicing | c.826+1G>C |
| 1 | FRAS1 | Exon10 | stopgain SNV | c.988G>T |
| 2 | FRAS1 | Exon65 | splicing | c.10174+1G>A |
| 1 | FRAS1 | Exon67 | splicing | c.10540+2_10540+2delT |
| 1 | FREM1 | Exon38 | frameshift deletion | c.6429delA |
| 4 | FREM1 | Exon16 | frameshift deletion | c.2630delT |
| 3 | FRG2B | Exon4 | frameshift deletion | c.480delG |
| 9 | FRG2C | Exon1 | frameshift deletion | c.129_130delAG |
| 1 | FRG2C | Exon4 | stopgain SNV | c.793C>T |
| 1 | FRMD4A | Exon23 | frameshift insertion | c.3014dupC |
| 1 | FRMD4A | Exon17 | stopgain SNV | c.1281T>G |
| 8 | FRMPD2 | Exon26 | frameshift deletion | c.3251delA |
| 1 | FRMPD2 | Exon25 | frameshift deletion | c.3183delC |
| 1 | FRMPD2 | Exon6 | splicing | c.567+2_567+2delT |
| 8 | FRMPD4 | Exon15 | stopgain SNV | c.2067C>G |
| 1 | FRS3 | Exon7 | frameshift deletion | c.783delC |
| 1 | FRS3 | Exon4 | frameshift deletion | c.97delC |
| 2 | FRY | Exon9 | frameshift deletion | c.899delA |
| 1 | FRY | Exon47 | frameshift deletion | c.6692delT |
| 1 | FRY | Exon47 | frameshift insertion | c.6698_6699insC |
| 1 | FSCB | Exon1 | stopgain SNV | c.595C>T |
| 1 | FSCB | Exon1 | stopgain SNV | c.220C>T |
| 1 | FSCN3 | Exon5 | splicing | c.1121-1G>C |
| 1 | FSD2 | Exon12 | stopgain SNV | c.1855C>T |
| 1 | FSIP1 | Exon9 | stopgain SNV | c.1032dupT |
| 1 | FSTL4 | Exon9 | frameshift deletion | c.1074_1075delCA |
| 1 | FST | Exon2 | frameshift deletion | c.252delC |
| 1 | FTHL17 | Exon1 | stopgain SNV | c.442G>T |
| 1 | FTHL17 | Exon1 | stopgain SNV | c.358C>T |
| 3 | FTSJD2 | Exon23 | stopgain SNV | c.2299A>T |
| 5 | FUBP1 | Exon3 | splicing | c.121-1G>T |
| 1 | FUCA1 | Exon9 | splicing | c.1261-1G>C |
| 1 | FUS | Exon6 | frameshift deletion | c.605delG |
| 1 | FUS | Exon6 | frameshift deletion | c.608delG |
| 1 | FUT10 | Exon4 | frameshift insertion | c.811dupA |
| 1 | FUT6 | Exon3 | stopgain SNV | c.945C>A |
| 1 | FXC1 | Exon3 | stoploss SNV | c.310T>G |
| 2 | FXYD4 | Exon3 | splicing | c.37+2T>G |
| 1 | FYCO1 | Exon8 | frameshift insertion | c.934dupG |
| 1 | FYCO1 | Exon8 | frameshift insertion | c.931_932insG |
| 1 | FYCO1 | Exon8 | frameshift insertion | c.827_828insCT |
| 1 | FYCO1 | Exon8 | frameshift insertion | c.824dupA |
| 1 | FZD1 | Exon1 | frameshift deletion | c.1464_1465delTG |
| 1 | FZD1 | Exon1 | frameshift insertion | c.1744_1745insAA |
| 1 | FZD1 | Exon1 | frameshift deletion | c.1842delC |
| 1 | FZD4 | Exon2 | frameshift insertion | c.333_334insTC |
| 1 | G0S2 | Exon2 | stoploss SNV | c.311A>G |
| 3 | G3BP1 | Exon5 | stopgain SNV | c.439G>T |
| 1 | G3BP1 | Exon8 | frameshift insertion | c.744_745insG |
| 1 | GAB2 | Exon6 | frameshift deletion | c.1535_1536delCT |
| 1 | GAB4 | Exon5 | splicing | c.687-2_687-1insC |
| 1 | GABBR1 | Exon23 | frameshift insertion | c.2838dupC |
| 1 | GABPA | Exon4 | splicing | c.223-1G>C |
| 1 | GABRB1 | Exon9 | stopgain SNV | c.1144G>T |
| 1 | GABRB3 | Exon4 | stopgain SNV | c.371T>A |
| 12 | GAD1 | Exon12 | stopgain SNV | c.1129G>T |
| 1 | GAK | Exon6 | frameshift deletion | c.634_635delGC |
| 3 | GAL3ST2 | Exon1 | splicing | c.29+1G>A |
| 1 | GAL3ST3 | Exon3 | stopgain SNV | c.304G>T |
| 1 | GAL3ST4 | Exon4 | stopgain SNV | c.1085_1086insA |
| 1 | GALC | Exon7 | stopgain SNV | c.550A>T |
| 1 | GALNT14 | Exon10 | stopgain SNV | c.943C>T |
| 1 | GALNT14 | Exon5 | frameshift deletion | c.488delT |
| 1 | GALNT1 | Exon7 | frameshift deletion | c.1109delC |
| 1 | GALNT2 | Exon5 | frameshift insertion | c.491dupC |
| 1 | GALNT3 | Exon2 | frameshift deletion | c.148_149delTC |
| 1 | GALNT6 | Exon5 | frameshift insertion | c.785_786insA |
| 2 | GALNTL5 | Exon3 | splicing | c.368+1G>C |
| 1 | GALNTL5 | Exon6 | splicing | c.908+2T>A |
| 1 | GALT | Exon8 | frameshift deletion | c.701delT |
| 1 | GANC | Exon5 | stopgain SNV | c.493C>T |
| 1 | GANC | Exon8 | splicing | c.626-2A>G |
| 1 | GANC | Exon11 | frameshift insertion | c.1129dupG |
| 1 | GANC | Exon17 | stopgain SNV | c.1999C>T |
| 2 | GAPVD1 | Exon3 | frameshift insertion | c.218_219insGT |
| 4 | GARNL3 | Exon13 | frameshift deletion | c.1029delG |
| 8 | GART | Exon18 | frameshift deletion | c.2420delA |
| 1 | GAS2L2 | Exon6 | stopgain SNV | c.1120C>T |
| 2 | GAS2 | Exon2 | frameshift deletion | c.36delA |
| 1 | GATM | Exon5 | stopgain SNV | c.778C>T |
| 1 | GBA2 | Exon9 | stopgain SNV | c.1564C>T |
| 1 | GBAS | Exon6 | splicing | c.595+1G>T |
| 1 | GBE1 | Exon6 | splicing | c.691+2T>C |
| 1 | GBF1 | Exon11 | frameshift deletion | c.1158_1159delAC |
| 1 | GBF1 | Exon24 | frameshift insertion | c.3082_3083insT |
| 1 | GBGT1 | Exon7 | frameshift insertion | c.816_817insC |
| 13 | GBGT1 | Exon7 | stopgain SNV | c.363C>A |
| 1 | GBP3 | Exon10 | splicing | c.1363-1G>C |
| 3 | GBP3 | Exon8 | stopgain SNV | c.1360C>T |
| 6 | GBP4 | Exon8 | splicing | c.1197+1G>A |
| 1 | GBP4 | Exon2 | stopgain SNV | c.49G>T |
| 2 | GBP5 | Exon8 | frameshift deletion | c.1214delG |
| 1 | GBP5 | Exon7 | splicing | c.868+1G>A |
| 1 | GBP5 | Exon5 | stopgain SNV | c.622C>T |
| 2 | GBP5 | Exon5 | stopgain SNV | c.502_505dupGACT |
| 1 | GBP7 | Exon10 | frameshift insertion | c.1620dupC |
| 1 | GBP7 | Exon10 | stopgain SNV | c.1501G>T |
| 1 | GBP7 | Exon5 | frameshift deletion | c.553delC |
| 2 | GBP7 | Exon4 | splicing | c.191-2A>G |
| 1 | GCA | Exon6 | stopgain SNV | c.556C>T |
| 16 | GCC2 | Exon14 | stopgain SNV | c.3676delG |
| 2 | GCKR | Exon13 | frameshift insertion | c.1135dupA |
| 1 | GCKR | Exon13 | stopgain SNV | c.1141C>T |
| 1 | GCKR | Exon17 | frameshift deletion | c.1513_1514delAT |
| 11 | GCNT1 | Exon3 | stopgain SNV | c.137T>A |
| 1 | GCNT3 | Exon3 | stopgain SNV | c.212C>G |
| 1 | GCNT3 | Exon3 | stopgain SNV | c.451C>T |
| 1 | GCNT3 | Exon3 | stopgain SNV | c.907C>T |
| 1 | GCNT4 | Exon1 | stopgain SNV | c.1087C>T |
| 5 | GCNT7 | Exon5 | stoploss SNV | c.193T>C |
| 2 | GDF3 | Exon1 | frameshift deletion | c.106delG |
| 1 | GDF9 | Exon2 | stopgain SNV | c.517A>T |
| 1 | GDPD3 | Exon1 | frameshift deletion | c.49delA |
| 1 | GDPD3 | Exon1 | frameshift insertion | c.43_44insC |
| 1 | GDPD3 | Exon1 | frameshift deletion | c.37_38delGG |
| 1 | GDPD3 | Exon1 | frameshift insertion | c.28_29insACGG |
| 1 | GDPD4 | Exon14 | frameshift insertion | c.1400dupT |
| 3 | GDPD4 | Exon12 | frameshift insertion | c.1192dupA |
| 1 | GDPD4 | Exon9 | frameshift deletion | c.638delA |
| 1 | GEMIN4 | Exon2 | stopgain SNV | c.1522C>T |
| 1 | GEMIN5 | Exon26 | frameshift deletion | c.3789_3790delGG |
| 1 | GEMIN5 | Exon13 | frameshift insertion | c.1729dupC |
| 1 | GFI1 | Exon7 | splicing | c.925-14_925-5delCTCTCTCTCT |
| 6 | GFI1 | Exon7 | splicing | c.925-10_925-5delCTCTCT |
| 1 | GFM1 | Exon6 | frameshift deletion | c.720delT |
| 1 | GFM1 | Exon13 | frameshift deletion | c.1596delT |
| 1 | GFM2 | Exon10 | frameshift deletion | c.753delT |
| 1 | GGCX | Exon15 | stopgain SNV | c.2110C>T |
| 8 | GGN | Exon4 | stopgain SNV | c.1939C>T |
| 1 | GGT1 | Exon6 | stopgain SNV | c.295C>T |
| 1 | GGT1 | Exon9 | splicing | c.576-2A>G |
| 7 | GGT1 | Exon9 | splicing | c.733+1_733+1delG |
| 1 | GGT5 | Exon2 | frameshift deletion | c.301delA |
| 3 | GH2 | Exon4 | stopgain SNV | c.642G>A |
| 1 | GH2 | Exon4 | frameshift deletion | c.522delA |
| 1 | GHDC | Exon9 | stoploss SNV | c.1424G>T |
| 1 | GHITM | Exon9 | stopgain SNV | c.994C>T |
| 1 | GHRHR | Exon7 | stopgain SNV | c.728G>A |
| 5 | GHRHR | Exon13 | stopgain SNV | c.1173G>A |
| 1 | GHRL | Exon3 | frameshift insertion | c.198dupC |
| 1 | GHR | Exon10 | frameshift deletion | c.1802_1803delTA |
| 1 | GIMAP2 | Exon3 | stopgain SNV | c.466delC |
| 1 | GIMAP6 | Exon4 | splicing | c.86-2A>G |
| 2 | GIMAP7 | Exon2 | frameshift deletion | c.64delA |
| 1 | GIMAP8 | Exon2 | frameshift deletion | c.101_102delAT |
| 1 | GIMAP8 | Exon5 | frameshift insertion | c.1584dupT |
| 3 | GINS3 | Exon2 | frameshift deletion | c.348_349delCT |
| 4 | GJA10 | Exon1 | stopgain SNV | c.1477C>T |
| 6 | GJA1 | Exon2 | frameshift deletion | c.932delC |
| 1 | GJA3 | Exon2 | frameshift insertion | c.357_358insA |
| 1 | GJA3 | Exon2 | frameshift deletion | c.355delA |
| 3 | GJA4 | Exon2 | stopgain SNV | c.43C>T |
| 1 | GJA8 | Exon2 | frameshift deletion | c.1292_1293delTA |
| 1 | GJA8 | Exon2 | frameshift deletion | c.1297_1298delGT |
| 1 | GJA9 | Exon2 | stoploss SNV | c.1548A>T |
| 1 | GJA9 | Exon2 | frameshift deletion | c.1149_1153delCTCTA |
| 1 | GJB2 | Exon2 | frameshift deletion | c.167delT |
| 1 | GJB2 | Exon2 | stopgain SNV | c.71G>A |
| 3 | GJB2 | Exon2 | frameshift deletion | c.35delG |
| 2 | GJB4 | Exon2 | frameshift deletion | c.153delT |
| 2 | GJB4 | Exon2 | stopgain SNV | c.238C>T |
| 1 | GJB4 | Exon2 | stopgain SNV | c.384G>A |
| 1 | GJB7 | Exon3 | frameshift insertion | c.644dupT |
| 1 | GJB7 | Exon3 | frameshift deletion | c.374_378delTTATC |
| 1 | GJB7 | Exon3 | frameshift deletion | c.34_38delGGAGT |
| 1 | GJD4 | Exon2 | frameshift deletion | c.834_835delCA |
| 1 | GKN2 | Exon3 | splicing | c.66+2dupT |
| 1 | GLB1L2 | Exon6 | stopgain SNV | c.615T>A |
| 1 | GLB1L2 | Exon15 | splicing | c.1507+1G>C |
| 1 | GLB1L | Exon6 | stopgain SNV | c.504T>G |
| 1 | GLCE | Exon3 | frameshift deletion | c.131delG |
| 1 | GLDC | Exon5 | frameshift deletion | c.707delG |
| 1 | GLDC | Exon3 | stopgain SNV | c.389G>A |
| 1 | GLG1 | Exon6 | frameshift deletion | c.1152delA |
| 1 | GLI1 | Exon6 | frameshift insertion | c.437dupG |
| 1 | GLI1 | Exon10 | frameshift insertion | c.2698_2699insA |
| 1 | GLI1 | Exon10 | frameshift deletion | c.2703_2704delCG |
| 1 | GLIPR1L2 | Exon4 | frameshift insertion | c.666_667insC |
| 1 | GLIPR1 | Exon4 | frameshift deletion | c._del |
| 1 | GLIPR1 | Exon6 | stopgain SNV | c.675G>A |
| 1 | GLO1 | Exon4 | frameshift deletion | c.365delC |
| 2 | GLRA3 | Exon3 | frameshift insertion | c.243_244insC |
| 8 | GLRA3 | Exon3 | frameshift deletion | c.240delC |
| 1 | GLRA4 | Exon8 | stopgain SNV | c.1039A>T |
| 2 | GLS2 | Exon10 | splicing | c.929+1G>A |
| 3 | GLT1D1 | Exon8 | stopgain SNV | c.760C>T |
| 1 | GLT8D1 | Exon4 | frameshift deletion | c.188_189delAA |
| 1 | GLT8D2 | Exon6 | frameshift deletion | c.313_314delGA |
| 1 | GLTSCR2 | Exon3 | frameshift deletion | c.312delA |
| 1 | GLUD2 | Exon1 | stopgain SNV | c.412C>T |
| 1 | GLUD2 | Exon1 | frameshift insertion | c.673_674insT |
| 17 | GLYR1 | Exon13 | frameshift insertion | c.1140dupG |
| 1 | GMEB2 | Exon3 | frameshift deletion | c.181delG |
| 3 | GMEB2 | Exon3 | frameshift deletion | c.175delG |
| 1 | GML | Exon3 | splicing | c.181+1G>A |
| 1 | GMPR2 | Exon5 | frameshift deletion | c.438delG |
| 3 | GMPR | Exon8 | frameshift insertion | c.854_855dupAC |
| 2 | GMPR | Exon9 | frameshift insertion | c.969_972dupACTC |
| 1 | GNAI3 | Exon4 | frameshift insertion | c.364_365insC |
| 1 | GNAI3 | Exon8 | stopgain SNV | c.892G>T |
| 1 | GNAI3 | Exon8 | frameshift deletion | c.895_896delGG |
| 2 | GNAI3 | Exon8 | stopgain SNV | c.906delT |
| 1 | GNAI3 | Exon8 | frameshift deletion | c.1010delA |
| 3 | GNAL | Exon10 | frameshift deletion | c.1079_1080delTG |
| 1 | GNAT3 | Exon6 | frameshift deletion | c.621_624delGAGA |
| 2 | GNB1 | Exon7 | stopgain SNV | c.297G>A |
| 1 | GNB2 | Exon5 | frameshift deletion | c.207delG |
| 1 | GNB2 | Exon10 | frameshift insertion | c.985_986insG |
| 1 | GNB5 | Exon7 | stopgain SNV | c.534dupT |
| 2 | GNL1 | Exon9 | stopgain SNV | c.1270C>T |
| 1 | GNL1 | Exon9 | frameshift deletion | c.1250delT |
| 3 | GNLY | Exon1 | stopgain SNV | c.11G>A |
| 2 | GNLY | Exon2 | frameshift deletion | c.126delC |
| 2 | GNLY | Exon3 | splicing | c.255+2T>C |
| 1 | GNPTAB | Exon19 | frameshift deletion | c.3503_3504delTC |
| 1 | GNPTAB | Exon11 | frameshift deletion | c.1399delG |
| 1 | GNPTG | Exon9 | frameshift deletion | c.714delG |
| 2 | GNRH2 | Exon4 | frameshift deletion | c.337_341delGCCCC |
| 1 | GNRH2 | Exon4 | frameshift insertion | c.337_341dupGCCCC |
| 1 | GNS | Exon4 | splicing | c.459+1G>C |
| 2 | GOLGA1 | Exon3 | frameshift deletion | c.12delA |
| 1 | GOLGA3 | Exon16 | frameshift deletion | c.3215_3216delCC |
| 2 | GOLGA3 | Exon4 | frameshift deletion | c.512delA |
| 1 | GOLGA6A | Exon15 | stopgain SNV | c.1639C>T |
| 2 | GOLGA6B | Exon15 | frameshift insertion | c.1641dupG |
| 4 | GOLGA6C | Exon18 | stopgain SNV | c.2041C>T |
| 1 | GOLGA6C | Exon18 | stopgain SNV | c.2044C>T |
| 1 | GOLGA8B | Exon16 | stopgain SNV | c.1617T>A |
| 1 | GOLGB1 | Exon14 | stopgain SNV | c.7072C>T |
| 1 | GOLGB1 | Exon10 | stopgain SNV | c.1333C>T |
| 1 | GOLPH3L | Exon5 | frameshift insertion | c.542dupT |
| 1 | GON4L | Exon31 | frameshift deletion | c.6537delC |
| 2 | GOT1L1 | Exon9 | frameshift deletion | c.1087delA |
| 19 | GOT1L1 | Exon6 | frameshift insertion | c.696dupT |
| 1 | GP1BA | Exon2 | stopgain SNV | c.209dupA |
| 1 | GP2 | Exon5 | splicing | c.646+2T>A |
| 1 | GP6 | Exon8 | frameshift insertion | c.1752_1753insTCCC |
| 11 | GP6 | Exon8 | frameshift insertion | c.1206_1209dupTCTG |
| 1 | GPATCH3 | Exon7 | stopgain SNV | c.1421T>A |
| 1 | GPATCH3 | Exon1 | stopgain SNV | c.22G>T |
| 1 | GPC4 | Exon9 | frameshift insertion | c.1628_1629insA |
| 4 | GPC6 | Exon1 | stopgain SNV | c.38T>A |
| 6 | GPHN | Exon23 | frameshift deletion | c.2262delG |
| 1 | GPHN | Exon23 | frameshift deletion | c.2271_2272delAA |
| 5 | GPLD1 | Exon25 | frameshift deletion | c.2442delA |
| 4 | GPN3 | Exon7 | stopgain SNV | c.879T>G |
| 1 | GPNMB | Exon3 | frameshift deletion | c.319_332delAAGGAAGATGCCAA |
| 1 | GPNMB | Exon3 | splicing | c.367+2T>C |
| 1 | GPNMB | Exon6 | splicing | c.701-2A>C |
| 6 | GPNMB | Exon11 | stopgain SNV | c.1663G>T |
| 1 | GPR108 | Exon13 | frameshift deletion | c.1189_1190delCT |
| 1 | GPR111 | Exon5 | frameshift insertion | c.177_178insG |
| 1 | GPR112 | Exon6 | frameshift insertion | c.2463dupT |
| 1 | GPR112 | Exon23 | frameshift deletion | c.8862delG |
| 1 | GPR113 | Exon8 | stopgain SNV | c.1926C>A |
| 2 | GPR113 | Exon8 | stopgain SNV | c.1912C>T |
| 1 | GPR114 | Exon7 | frameshift insertion | c.608dupG |
| 1 | GPR115 | Exon5 | stopgain SNV | c.367C>T |
| 1 | GPR116 | Exon21 | stoploss SNV | c.4039T>A |
| 7 | GPR125 | Exon10 | frameshift deletion | c.1358delA |
| 4 | GPR125 | Exon9 | frameshift deletion | c.1148delG |
| 2 | GPR128 | Exon3 | frameshift deletion | c.332delA |
| 7 | GPR128 | Exon11 | frameshift deletion | c.1323delA |
| 1 | GPR133 | Exon5 | frameshift insertion | c.405dupA |
| 1 | GPR133 | Exon11 | frameshift insertion | c.1241_1242insCG |
| 1 | GPR135 | Exon1 | frameshift insertion | c.1027_1028dupTG |
| 8 | GPR142 | Exon1 | frameshift deletion | c.215delC |
| 2 | GPR142 | Exon3 | frameshift deletion | c.364delC |
| 2 | GPR149 | Exon1 | stopgain SNV | c.922C>T |
| 1 | GPR151 | Exon1 | frameshift insertion | c.949_950insG |
| 1 | GPR151 | Exon1 | frameshift deletion | c.663_676delTTTCTGGAGAGCTT |
| 1 | GPR151 | Exon1 | frameshift deletion | c.590_591delAG |
| 3 | GPR151 | Exon1 | stopgain SNV | c.283C>T |
| 1 | GPR155 | Exon8 | stopgain SNV | c.1453_1460dupATAATATC |
| 1 | GPR156 | Exon9 | frameshift deletion | c.2001_2005delACCAG |
| 1 | GPR172B | Exon5 | frameshift deletion | c.1292delG |
| 1 | GPR173 | Exon2 | frameshift deletion | c.594_595delAG |
| 1 | GPR173 | Exon2 | frameshift deletion | c.603delT |
| 1 | GPR179 | Exon3 | frameshift deletion | c.984delC |
| 1 | GPR179 | Exon1 | stopgain SNV | c.637A>T |
| 1 | GPR17 | Exon3 | stopgain SNV | c.64C>T |
| 7 | GPR183 | Exon2 | stopgain SNV | c.114C>G |
| 1 | GPR4 | Exon2 | frameshift insertion | c.459_460insGTCAT |
| 1 | GPR84 | Exon2 | stopgain SNV | c.652C>T |
| 1 | GPR84 | Exon2 | frameshift deletion | c.468delG |
| 1 | GPR87 | Exon3 | frameshift deletion | c.182delG |
| 2 | GPR89A | Exon8 | frameshift deletion | c.696_697delAA |
| 2 | GPR89B | Exon8 | frameshift deletion | c.697_698delAA |
| 1 | GPR97 | Exon11 | frameshift deletion | c.1473delG |
| 1 | GPR98 | Exon33 | frameshift deletion | c.7916delG |
| 1 | GPR98 | Exon44 | frameshift deletion | c.9620delA |
| 1 | GPR98 | Exon68 | splicing | c.13893+1G>A |
| 1 | GPR98 | Exon69 | stopgain SNV | c.13962T>G |
| 1 | GPRASP1 | Exon4 | frameshift insertion | c.4008_4009insT |
| 4 | GPRC5A | Exon2 | frameshift deletion | c.183delG |
| 1 | GPRC5C | Exon2 | frameshift deletion | c.122_123delGC |
| 4 | GPRC5D | Exon1 | frameshift deletion | c.821_837delCTTTACAAGGCAATGCC |
| 1 | GPRC5D | Exon1 | frameshift deletion | c.191delT |
| 1 | GPRIN1 | Exon2 | frameshift insertion | c.711_712insG |
| 1 | GPRIN1 | Exon2 | stopgain SNV | c.610delA |
| 1 | GPSM3 | Exon7 | frameshift insertion | c.266dupA |
| 2 | GRAMD1A | Exon9 | splicing | c.1048+1G>A |
| 1 | GRAMD1B | Exon10 | frameshift deletion | c.1047delC |
| 3 | GRAMD2 | Exon9 | frameshift deletion | c.710_713delACAG |
| 1 | GRAMD4 | Exon4 | frameshift insertion | c.426_427insT |
| 1 | GRB7 | Exon2 | frameshift deletion | c.105delG |
| 1 | GREB1 | Exon14 | frameshift insertion | c.1956_1957insC |
| 1 | GRHL2 | Exon15 | stopgain SNV | c.1705G>T |
| 4 | GRIA1 | Exon11 | splicing | c.1823+2T>A |
| 1 | GRIK3 | Exon13 | frameshift insertion | c.1899dupG |
| 1 | GRIN2B | Exon7 | stopgain SNV | c.1555C>T |
| 1 | GRIN3B | Exon3 | stopgain SNV | c.1724G>A |
| 1 | GRIP1 | Exon15 | splicing | c.1613-2dupA |
| 5 | GRIP2 | Exon21 | stopgain SNV | c.2788C>T |
| 1 | GRIP2 | Exon10 | frameshift insertion | c.1311dupT |
| 3 | GRIP2 | Exon2 | frameshift deletion | c.228delA |
| 1 | GRIPAP1 | Exon21 | frameshift deletion | c.1883delT |
| 1 | GRM2 | Exon2 | frameshift deletion | c.113delT |
| 1 | GRM3 | Exon3 | frameshift deletion | c.1012delC |
| 1 | GRM5 | Exon4 | stopgain SNV | c.1003A>T |
| 1 | GRN | Exon2 | frameshift deletion | c.95_96delTG |
| 4 | GRXCR2 | Exon2 | frameshift deletion | c.543delA |
| 1 | GSC | Exon3 | stopgain SNV | c.644_645insAGTCTT |
| 7 | GSG1 | Exon5 | stopgain SNV | c.601C>T |
| 1 | GSG2 | Exon1 | frameshift deletion | c.1348_1349delCT |
| 1 | GSG2 | Exon1 | frameshift insertion | c.2070_2071insC |
| 1 | GSS | Exon2 | frameshift deletion | c.4delG |
| 1 | GSTA1 | Exon5 | frameshift deletion | c.331_338delGTATGTCC |
| 1 | GSTA3 | Exon4 | frameshift deletion | c.172_181delGTTGAGATTG |
| 1 | GSTA5 | Exon4 | splicing | c.139+1G>A |
| 2 | GSTM4 | Exon4 | splicing | c.259+1G>A |
| 3 | GSTM4 | Exon7 | frameshift deletion | c.534delT |
| 1 | GSTO2 | Exon3 | splicing | c.35-2A>G |
| 1 | GSTT2 | Exon5 | stopgain SNV | c.586C>T |
| 1 | GTF2B | Exon3 | frameshift insertion | c.146_147insC |
| 2 | GTF2F1 | Exon9 | splicing | c.837-2A>G |
| 1 | GTF2H4 | Exon8 | frameshift deletion | c.709delC |
| 1 | GTF2H4 | Exon8 | frameshift insertion | c.717_718insGG |
| 2 | GTF2I | Exon16 | frameshift deletion | c.1336_1339delAAGA |
| 1 | GTF2IRD2 | Exon16 | stopgain SNV | c.1375G>T |
| 1 | GTF3C1 | Exon30 | frameshift deletion | c.4438delC |
| 1 | GTF3C1 | Exon20 | frameshift deletion | c.3188delG |
| 1 | GTF3C1 | Exon20 | frameshift insertion | c.3178_3179insTT |
| 1 | GTF3C5 | Exon11 | frameshift insertion | c.1507_1508insTA |
| 1 | GTPBP10 | Exon8 | stopgain SNV | c.839T>A |
| 1 | GTPBP5 | Exon4 | frameshift deletion | c.368delA |
| 1 | GTPBP8 | Exon2 | splicing | c.467+1G>T |
| 1 | GTSE1 | Exon6 | splicing | c.928-1G>T |
| 1 | GTSE1 | Exon8 | frameshift deletion | c.1487delC |
| 7 | GUCA1C | Exon1 | stopgain SNV | c.52G>T |
| 1 | GUCA2A | Exon2 | stopgain SNV | c.265G>T |
| 1 | GUCY2C | Exon3 | stopgain SNV | c.377dupA |
| 1 | GUCY2D | Exon12 | frameshift deletion | c.2299_2300delTG |
| 1 | GUCY2D | Exon15 | splicing | c.2944+1_2944+1delG |
| 1 | GUCY2F | Exon18 | frameshift deletion | c.3195delC |
| 3 | GUCY2F | Exon18 | frameshift deletion | c.3192delA |
| 1 | GUCY2F | Exon17 | splicing | c.3055+1G>A |
| 1 | GUCY2F | Exon11 | frameshift deletion | c.2240delG |
| 1 | GUF1 | Exon6 | splicing | c.669+1dupG |
| 1 | GUF1 | Exon8 | splicing | c.938+1G>T |
| 2 | GUF1 | Exon12 | frameshift deletion | c.1402_1403delGA |
| 1 | GUSB | Exon12 | stopgain SNV | c.1867C>T |
| 1 | GYG2 | Exon5 | splicing | c.23-2A>G |
| 1 | GYLTL1B | Exon11 | stopgain SNV | c.1359G>A |
| 1 | GYLTL1B | Exon13 | stopgain SNV | c.1870C>T |
| 1 | GZMA | Exon5 | stopgain SNV | c.758G>A |
| 1 | GZMH | Exon5 | stoploss SNV | c.740A>T |
| 1 | H2AFY2 | Exon2 | frameshift insertion | c.73dupG |
| 1 | H6PD | Exon4 | stopgain SNV | c.793C>T |
| 1 | HAAO | Exon7 | stopgain SNV | c.577G>T |
| 1 | HABP2 | Exon7 | stopgain SNV | c.529C>T |
| 1 | HABP2 | Exon13 | stopgain SNV | c.1516G>T |
| 1 | HACL1 | Exon10 | frameshift deletion | c.811delC |
| 7 | HADHB | Exon11 | frameshift deletion | c.960delC |
| 1 | HADH | Exon2 | frameshift deletion | c.142delG |
| 1 | HAO1 | Exon5 | stopgain SNV | c.787C>T |
| 1 | HAO2 | Exon7 | frameshift deletion | c.989delT |
| 4 | HAP1 | Exon11 | stopgain SNV | c.1847C>A |
| 1 | HAPLN4 | Exon3 | stopgain SNV | c.208_209insG |
| 1 | HAS2 | Exon4 | frameshift deletion | c.1363delA |
| 1 | HAUS1 | Exon4 | stopgain SNV | c.468T>A |
| 1 | HAUS3 | Exon5 | stopgain SNV | c.1711G>T |
| 1 | HAUS3 | Exon2 | frameshift deletion | c.811delT |
| 1 | HAUS4 | Exon4 | splicing | c.56-2A>T |
| 1 | HAVCR1 | Exon3 | frameshift insertion | c.147_148dupAG |
| 1 | HAVCR2 | Exon5 | frameshift deletion | c.572delA |
| 1 | HBB | Exon1 | stopgain SNV | c.47G>A |
| 1 | HCAR3 | Exon1 | frameshift insertion | c.1085_1086insTCCTT |
| 2 | HCAR3 | Exon1 | frameshift deletion | c.1072delC |
| 1 | HCAR3 | Exon1 | frameshift deletion | c.1070delA |
| 1 | HCFC1 | Exon24 | frameshift insertion | c.5953_5954insT |
| 1 | HCN3 | Exon8 | frameshift deletion | c.2296delC |
| 4 | HCN4 | Exon2 | stopgain SNV | c.905C>G |
| 1 | HDAC1 | Exon13 | frameshift insertion | c.1392_1393insG |
| 1 | HDAC1 | Exon13 | frameshift deletion | c.1399delG |
| 1 | HDAC4 | Exon11 | frameshift insertion | c.1124_1125insTGTTT |
| 14 | HDGFRP3 | Exon7 | splicing | c.607-1G>C |
| 3 | HEATR1 | Exon46 | splicing | c.6347-10_6347-3delTTTTTTTT |
| 1 | HEATR1 | Exon23 | stopgain SNV | c.3224T>G |
| 1 | HEATR7A | Exon12 | frameshift insertion | c.1116_1117insGC |
| 4 | HEATR7B2 | Exon38 | frameshift insertion | c.4278dupT |
| 1 | HEATR7B2 | Exon20 | stopgain SNV | c.2053C>T |
| 1 | HEATR7B2 | Exon10 | stopgain SNV | c.988C>T |
| 1 | HEATR7B2 | Exon5 | frameshift deletion | c.436_443delAGGATGAA |
| 1 | HEATR8 | Exon24 | stopgain SNV | c.3934C>T |
| 4 | HECTD1 | Exon14 | splicing | c.1933-12_1933-4delCTACACATT |
| 1 | HECW1 | Exon29 | frameshift insertion | c.4596_4597insA |
| 3 | HECW2 | Exon14 | stopgain SNV | c.2947A>T |
| 1 | HEG1 | Exon5 | frameshift deletion | c.1403delA |
| 20 | HELB | Exon4 | frameshift deletion | c.1566delG |
| 1 | HELB | Exon4 | frameshift deletion | c.1570delA |
| 6 | HEPACAM2 | Exon7 | stopgain SNV | c.1199C>A |
| 1 | HEPACAM2 | Exon2 | stopgain SNV | c.193C>T |
| 1 | HEPHL1 | Exon1 | stopgain SNV | c.13C>T |
| 1 | HEPHL1 | Exon12 | stopgain SNV | c.2187C>A |
| 1 | HEPHL1 | Exon17 | splicing | c.3045+2T>G |
| 1 | HEPHL1 | Exon20 | frameshift deletion | c.3340_3341delGC |
| 2 | HERC1 | Exon71 | frameshift insertion | c.13153dupC |
| 3 | HERC6 | Exon3 | stoploss SNV | c.367T>C |
| 1 | HERC6 | Exon6 | stoploss SNV | c.862T>C |
| 1 | HERC6 | Exon16 | frameshift insertion | c.2022dupA |
| 1 | HERPUD1 | Exon5 | frameshift deletion | c.541delT |
| 1 | HESX1 | Exon3 | splicing | c.357+1G>A |
| 1 | HEXA | Exon11 | frameshift insertion | c.1274_1277dupTATC |
| 1 | HFM1 | Exon22 | splicing | c.2427+1G>T |
| 3 | HFM1 | Exon11 | stopgain SNV | c.1327A>T |
| 1 | HFM1 | Exon9 | stopgain SNV | c.1029C>A |
| 1 | HFM1 | Exon7 | stopgain SNV | c.808A>T |
| 1 | HFM1 | Exon4 | frameshift deletion | c.455delC |
| 1 | HGSNAT | Exon15 | stopgain SNV | c.1516C>T |
| 1 | HHATL | Exon7 | frameshift insertion | c.694_695insCA |
| 2 | HHIPL2 | Exon9 | frameshift insertion | c.2046dupT |
| 1 | HHIPL2 | Exon6 | frameshift deletion | c._del |
| 1 | HHIPL2 | Exon2 | frameshift insertion | c.490dupG |
| 13 | HHIP | Exon9 | splicing | c.1424-1G>A |
| 1 | HHLA2 | Exon4 | splicing | c.418+1_418+1delG |
| 1 | HHLA2 | Exon6 | frameshift deletion | c.839delA |
| 1 | HHLA2 | Exon6 | stopgain SNV | c.870G>A |
| 1 | HIAT1 | Exon6 | frameshift deletion | c.496delA |
| 1 | HIBCH | Exon13 | frameshift deletion | c._del |
| 1 | HIC1 | Exon2 | frameshift deletion | c.1793delG |
| 1 | HIGD1B | Exon3 | stoploss SNV | c.298T>C |
| 1 | HINT3 | Exon2 | stopgain SNV | c.289A>T |
| 1 | HIP1R | Exon6 | frameshift deletion | c.491_492delCA |
| 1 | HIPK1 | Exon14 | stopgain SNV | c.2884C>T |
| 1 | HIPK3 | Exon16 | frameshift deletion | c.3132delT |
| 1 | HIST1H1T | Exon1 | stopgain SNV | c.502A>T |
| 1 | HIST1H1T | Exon1 | frameshift deletion | c.456_457delAG |
| 1 | HIST1H2AA | Exon1 | frameshift insertion | c.168dupA |
| 1 | HIST1H2AB | Exon1 | frameshift deletion | c.291_292delCT |
| 1 | HIST1H2AK | Exon1 | frameshift deletion | c.26delG |
| 1 | HIST1H2AL | Exon1 | frameshift deletion | c.160delG |
| 1 | HIST1H2BE | Exon1 | frameshift deletion | c.21delC |
| 1 | HIST1H2BE | Exon1 | frameshift deletion | c.61_65delAAGGC |
| 1 | HIST1H2BG | Exon1 | stopgain SNV | c.20C>G |
| 1 | HIST1H3A | Exon1 | stopgain SNV | c.166C>T |
| 3 | HIST1H4B | Exon1 | stopgain SNV | c.24dupT |
| 1 | HIST1H4B | Exon1 | stopgain SNV | c.15_16insT |
| 1 | HIST1H4D | Exon1 | frameshift deletion | c.294_295delTT |
| 1 | HIST1H4K | Exon1 | frameshift insertion | c.143_144insT |
| 1 | HIST1H4L | Exon1 | frameshift deletion | c.179_180delAA |
| 1 | HIST3H2BB | Exon1 | frameshift insertion | c.254dupA |
| 1 | HIVEP2 | Exon9 | frameshift deletion | c.5736_5737delTG |
| 1 | HIVEP3 | Exon7 | stopgain SNV | c.6078T>A |
| 2 | HIVEP3 | Exon7 | stopgain SNV | c.5638G>T |
| 1 | HJURP | Exon3 | splicing | c.118-2A>T |
| 1 | HK3 | Exon11 | stopgain SNV | c.1364T>G |
| 1 | HKDC1 | Exon11 | frameshift deletion | c.1604delG |
| 1 | HKDC1 | Exon15 | stopgain SNV | c.2163G>A |
| 1 | HKR1 | Exon6 | frameshift deletion | c.972_973delAG |
| 1 | HKR1 | Exon6 | stopgain SNV | c.1426C>T |
| 1 | HKR1 | Exon6 | stopgain SNV | c.1510C>T |
| 1 | HLA-A | Exon2 | frameshift insertion | c.260dupA |
| 17 | HLA-B | Exon2 | frameshift deletion | c.282_283delGG |
| 17 | HLA-B | Exon2 | frameshift insertion | c.279_280insAA |
| 5 | HLA-B | Exon2 | frameshift insertion | c.206_207insT |
| 16 | HLA-B | Exon2 | frameshift insertion | c.206_207insC |
| 13 | HLA-B | Exon2 | frameshift deletion | c.204delA |
| 1 | HLA-DPA1 | Exon2 | stopgain SNV | c.79C>T |
| 1 | HLA-DQB1 | Exon2 | stopgain SNV | c.316G>T |
| 3 | HLA-DQB1 | Exon2 | frameshift deletion | c.310delC |
| 3 | HLA-DQB1 | Exon2 | frameshift insertion | c.304_305insA |
| 13 | HLA-DRB1 | Exon3 | frameshift insertion | c.640_641insTG |
| 2 | HLA-DRB1 | Exon3 | frameshift insertion | c.583_584insA |
| 3 | HLA-DRB1 | Exon3 | frameshift deletion | c.511delA |
| 1 | HLA-DRB1 | Exon3 | splicing | c.370+1G>A |
| 2 | HLA-DRB1 | Exon2 | frameshift deletion | c.303_304delGG |
| 9 | HLA-DRB1 | Exon2 | frameshift deletion | c.301delC |
| 1 | HLA-DRB1 | Exon2 | frameshift insertion | c.299_300insAA |
| 6 | HLA-DRB1 | Exon2 | frameshift insertion | c.297_298insAA |
| 1 | HLA-DRB1 | Exon2 | frameshift insertion | c.295_296insGG |
| 2 | HLA-DRB1 | Exon2 | frameshift insertion | c.294_295insA |
| 2 | HLA-DRB1 | Exon2 | frameshift insertion | c.293dupA |
| 3 | HLA-DRB1 | Exon2 | frameshift insertion | c.196dupT |
| 1 | HLA-DRB1 | Exon2 | frameshift insertion | c.125_126insCATGA |
| 4 | HLA-DRB1 | Exon2 | splicing | c.100+1G>A |
| 12 | HLA-G | Exon4 | frameshift deletion | c.460delC |
| 1 | HLCS | Exon5 | frameshift deletion | c.768_769delGG |
| 1 | HLX | Exon4 | frameshift deletion | c.1354delG |
| 1 | HMCN1 | Exon31 | frameshift deletion | c._del |
| 4 | HMGXB3 | Exon12 | frameshift deletion | c.1984delG |
| 6 | HMMR | Exon11 | stopgain SNV | c.1054G>T |
| 1 | HNF1A | Exon4 | frameshift insertion | c.863_864insC |
| 1 | HNF1A | Exon4 | frameshift deletion | c.864delG |
| 1 | HNF1A | Exon5 | frameshift deletion | c.958delG |
| 1 | HNF1A | Exon6 | frameshift insertion | c.1230_1231insAC |
| 1 | HNF1A | Exon6 | frameshift insertion | c.1235_1236insTC |
| 4 | HNRNPA1L2 | Exon6 | frameshift deletion | c.76delA |
| 4 | HNRNPA1L2 | Exon6 | frameshift deletion | c.830delA |
| 1 | HNRNPD | Exon8 | splicing | c.854-1G>C |
| 1 | HNRPLL | Exon11 | splicing | c.1416+1G>A |
| 1 | HOMER2 | Exon2 | stopgain SNV | c.43C>T |
| 1 | HOOK2 | Exon5 | frameshift deletion | c.383delA |
| 1 | HOOK3 | Exon5 | stopgain SNV | c.397C>T |
| 6 | HORMAD1 | Exon3 | stopgain SNV | c.148G>T |
| 1 | HORMAD2 | Exon10 | splicing | c.569-1G>T |
| 1 | HOXA10 | Exon3 | splicing | c.959-2_959-1insAG |
| 1 | HOXA11 | Exon2 | frameshift deletion | c.912delA |
| 1 | HOXA11 | Exon1 | frameshift deletion | c.140delA |
| 1 | HOXA1 | Exon2 | frameshift deletion | c.972delG |
| 1 | HOXA1 | Exon1 | frameshift deletion | c.14delG |
| 2 | HOXA6 | Exon2 | frameshift deletion | c.545_548delGGCG |
| 3 | HOXA6 | Exon2 | frameshift deletion | c.540_541delGA |
| 3 | HOXA6 | Exon2 | frameshift deletion | c.503delT |
| 1 | HOXA6 | Exon2 | frameshift deletion | c.497delA |
| 1 | HPGD | Exon3 | frameshift deletion | c.310_311delCT |
| 1 | HPS4 | Exon14 | frameshift deletion | c.2043delC |
| 1 | HPSE2 | Exon9 | frameshift deletion | c.1291_1292delAA |
| 1 | HPSE | Exon13 | stopgain SNV | c.1480C>T |
| 2 | HPSE | Exon4 | stopgain SNV | c.388G>T |
| 1 | HRASLS | Exon4 | stopgain SNV | c.412C>T |
| 3 | HRNR | Exon3 | stopgain SNV | c.3232C>T |
| 1 | HRNR | Exon3 | stopgain SNV | c.2338C>T |
| 1 | HS6ST1 | Exon1 | stopgain SNV | c.199A>T |
| 1 | HS6ST2 | Exon2 | frameshift deletion | c.407delA |
| 3 | HS6ST2 | Exon2 | frameshift insertion | c.318dupC |
| 1 | HSD11B2 | Exon5 | frameshift insertion | c.818dupT |
| 1 | HSD17B13 | Exon4 | stopgain SNV | c.494C>G |
| 8 | HSD17B13 | Exon4 | frameshift deletion | c.465delC |
| 1 | HSD17B13 | Exon1 | frameshift insertion | c.144_145insTCCA |
| 2 | HSD17B14 | Exon7 | frameshift deletion | c.493delA |
| 1 | HSD17B14 | Exon6 | splicing | c.369+1G>T |
| 1 | HSD17B14 | Exon4 | stopgain SNV | c.235C>T |
| 3 | HSD17B2 | Exon5 | stoploss SNV | c.1163A>G |
| 2 | HSD17B4 | Exon7 | frameshift insertion | c.436_437insAC |
| 1 | HSD17B4 | Exon15 | frameshift deletion | c.1372_1373delGA |
| 1 | HSF2 | Exon9 | frameshift deletion | c.858delC |
| 1 | HSF4 | Exon12 | frameshift insertion | c.1038_1039insA |
| 2 | HSH2D | Exon4 | frameshift deletion | c.66_67delGA |
| 1 | HSH2D | Exon8 | stopgain SNV | c.880C>T |
| 1 | HSP90B1 | Exon5 | splicing | c.743+1G>A |
| 1 | HSPA13 | Exon3 | frameshift deletion | c.548_549delGA |
| 5 | HSPA6 | Exon1 | stopgain SNV | c.1309C>T |
| 1 | HSPA9 | Exon7 | frameshift deletion | c.626delG |
| 1 | HSPG2 | Exon55 | frameshift deletion | c.7018_7019delAC |
| 1 | HSPG2 | Exon40 | splicing |  |
| 1 | HSPG2 | Exon39 | frameshift deletion | c.4947_4948delCT |
| 1 | HSPG2 | Exon24 | frameshift insertion | c.3138dupG |
| 1 | HSPG2 | Exon24 | frameshift insertion | c.3132_3133insT |
| 3 | HTN3 | Exon5 | stopgain SNV | c.141T>A |
| 1 | HTR1A | Exon1 | frameshift insertion | c.272_273insA |
| 1 | HTR1E | Exon2 | frameshift insertion | c.239dupT |
| 1 | HTR3A | Exon3 | splicing | c.282+2T>G |
| 1 | HTR3E | Exon3 | stopgain SNV | c.350G>A |
| 1 | HTRA4 | Exon5 | splicing | c.999+2T>G |
| 1 | HYDIN | Exon54 | splicing | c.8847-1G>T |
| 1 | HYDIN | Exon41 | frameshift insertion | c.6341_6342dupTC |
| 1 | HYDIN | Exon35 | frameshift deletion | c.5310delC |
| 1 | HYDIN | Exon21 | stopgain SNV | c.3175C>T |
| 4 | HYDIN | Exon9 | stopgain SNV | c.1095dupT |
| 5 | IBSP | Exon7 | frameshift deletion | c.696delT |
| 1 | ICK | Exon15 | stopgain SNV | c.1783C>T |
| 1 | IDH3A | Exon2 | splicing | c.90+1G>A |
| 1 | IDH3G | Exon7 | frameshift deletion | c.460delG |
| 11 | IDI2 | Exon5 | stoploss SNV | c.684A>T |
| 1 | IDI2 | Exon4 | frameshift deletion | c.286delC |
| 1 | IFI27L1 | Exon3 | splicing | c.61+1G>A |
| 1 | IFI35 | Exon5 | frameshift deletion | c.537delG |
| 3 | IFI44L | Exon5 | stopgain SNV | c.873T>A |
| 1 | IFI44 | Exon4 | stopgain SNV | c.540T>A |
| 4 | IFIH1 | Exon15 | splicing | c.2807+1G>A |
| 3 | IFIH1 | Exon10 | stopgain SNV | c.1879G>T |
| 1 | IFIH1 | Exon9 | frameshift insertion | c.1764dupA |
| 7 | IFIH1 | Exon9 | splicing | c.1641+1G>C |
| 1 | IFIH1 | Exon5 | stopgain SNV | c.949C>T |
| 1 | IFIT1 | Exon2 | stopgain SNV | c.784C>T |
| 1 | IFIT3 | Exon2 | frameshift insertion | c.36_37insTG |
| 1 | IFIT3 | Exon2 | frameshift deletion | c.45_48delGCTG |
| 1 | IFIT3 | Exon2 | stopgain SNV | c.364C>T |
| 1 | IFLTD1 | Exon3 | stopgain SNV | c.382C>T |
| 1 | IFNA10 | Exon1 | stopgain SNV | c.373C>T |
| 1 | IFNA10 | Exon1 | frameshift deletion | c.9_13delGTCCT |
| 3 | IFNA5 | Exon1 | stopgain SNV | c.343C>T |
| 1 | IFNA5 | Exon1 | stopgain SNV | c.127C>T |
| 1 | IFNA6 | Exon1 | stopgain SNV | c.253C>T |
| 1 | IFNAR1 | Exon9 | frameshift insertion | c.1263dupT |
| 1 | IFNB1 | Exon1 | stopgain SNV | c.556C>T |
| 5 | IFNE | Exon1 | stopgain SNV | c.211C>T |
| 19 | IFNK | Exon1 | frameshift insertion | c.31_34dupTGTT |
| 1 | IFNW1 | Exon1 | frameshift deletion | c.229delA |
| 1 | IFT122 | Exon17 | stopgain SNV | c.2208G>A |
| 1 | IFT122 | Exon17 | frameshift insertion | c.2210_2211insGG |
| 1 | IFT140 | Exon31 | frameshift deletion | c.4328delG |
| 1 | IFT172 | Exon10 | splicing | c.786-2A>T |
| 1 | IFT172 | Exon10 | splicing | c.786-2A>G |
| 1 | IFT43 | Exon4 | splicing | c.310+2T>C |
| 3 | IFT74 | Exon2 | splicing | c.120+2dupT |
| 1 | IFT80 | Exon4 | frameshift deletion | c.301delA |
| 1 | IGDCC3 | Exon14 | frameshift deletion | c.2270delG |
| 1 | IGDCC3 | Exon14 | frameshift deletion | c.2264_2265delAG |
| 1 | IGDCC4 | Exon8 | stopgain SNV | c.1528G>T |
| 1 | IGDCC4 | Exon8 | frameshift deletion | c.1525delC |
| 3 | IGDCC4 | Exon8 | stopgain SNV | c.1518C>A |
| 3 | IGF2BP2 | Exon16 | stoploss SNV | c.1800delA |
| 1 | IGFBP1 | Exon4 | frameshift deletion | c.740delG |
| 1 | IGFBPL1 | Exon4 | frameshift deletion | c.824_830delATGACCG |
| 1 | IGFBPL1 | Exon4 | frameshift insertion | c.818_819insA |
| 4 | IGFLR1 | Exon5 | frameshift deletion | c.748_749delCT |
| 1 | IGHMBP2 | Exon8 | frameshift insertion | c.1079_1080insG |
| 2 | IGHMBP2 | Exon13 | frameshift deletion | c.1814delG |
| 2 | IGLL1 | Exon2 | frameshift deletion | c.258delG |
| 2 | IGSF10 | Exon6 | frameshift insertion | c.7350_7353dupATCA |
| 3 | IGSF10 | Exon4 | stopgain SNV | c.4804G>T |
| 1 | IGSF10 | Exon4 | frameshift deletion | c.3834_3835delCA |
| 1 | IGSF10 | Exon4 | frameshift deletion | c.1782delA |
| 1 | IGSF1 | Exon4 | frameshift deletion | c.212_213delTG |
| 1 | IGSF22 | Exon8 | splicing | c.696+1G>A |
| 1 | IGSF22 | Exon7 | stopgain SNV | c.694G>T |
| 2 | IGSF22 | Exon7 | splicing | c.479-2A>T |
| 1 | IGSF22 | Exon4 | splicing | c.110-2_110-2delA |
| 4 | IGSF3 | Exon9 | frameshift deletion | c.2464delG |
| 1 | IGSF3 | Exon7 | frameshift insertion | c.1585_1586insC |
| 1 | IKBKAP | Exon34 | stopgain SNV | c.3595A>T |
| 2 | IKBKAP | Exon19 | frameshift insertion | c.2032_2033insT |
| 3 | IKBKAP | Exon19 | frameshift deletion | c.2018delT |
| 1 | IKBKE | Exon18 | splicing | c.1835+1G>T |
| 4 | IKZF2 | Exon6 | stopgain SNV | c.391A>T |
| 2 | IKZF4 | Exon4 | frameshift deletion | c.468delT |
| 1 | IL12B | Exon4 | frameshift deletion | c.452_453delAT |
| 1 | IL12RB1 | Exon14 | stopgain SNV | c.1624C>T |
| 2 | IL17RB | Exon4 | splicing | c.354+2_354+5delTAAG |
| 1 | IL17RB | Exon11 | frameshift deletion | c.1153delC |
| 1 | IL18RAP | Exon3 | frameshift deletion | c.67delT |
| 1 | IL18RAP | Exon4 | frameshift deletion | c.167delT |
| 1 | IL19 | Exon5 | splicing | c.478-1G>A |
| 5 | IL1R1 | Exon5 | frameshift deletion | c.645delT |
| 2 | IL20 | Exon1 | frameshift deletion | c.19delG |
| 1 | IL20 | Exon1 | frameshift deletion | c.36delT |
| 1 | IL20 | Exon2 | splicing | c.225+1G>T |
| 1 | IL21R | Exon9 | frameshift deletion | c.1311_1312delGC |
| 1 | IL23R | Exon3 | frameshift deletion | c.210delC |
| 1 | IL28A | Exon6 | stopgain SNV | c.562C>T |
| 1 | IL4R | Exon4 | frameshift deletion | c.203delT |
| 1 | IL6ST | Exon10 | frameshift deletion | c.1089_1090delCT |
| 4 | IL8 | Exon2 | stopgain SNV | c.91G>T |
| 1 | IL9 | Exon5 | frameshift deletion | c.384delT |
| 1 | ILVBL | Exon14 | stopgain SNV | c.1548G>A |
| 1 | IMMT | Exon13 | stopgain SNV | c.1453C>T |
| 1 | IMP3 | Exon1 | stoploss SNV | c.554A>G |
| 1 | IMP5 | Exon1 | stopgain SNV | c.102G>A |
| 5 | IMPA1 | Exon4 | splicing | c.154-1G>C |
| 6 | IMPA2 | Exon7 | stopgain SNV | c.687C>A |
| 1 | INADL | Exon8 | frameshift deletion | c.1010delC |
| 1 | INADL | Exon14 | stopgain SNV | c.1677T>A |
| 1 | INCA1 | Exon8 | frameshift deletion | c.416delG |
| 5 | INCA1 | Exon5 | stopgain SNV | c.64C>T |
| 1 | INCENP | Exon10 | frameshift insertion | c.1584_1585insA |
| 1 | INCENP | Exon17 | frameshift deletion | c.2324delA |
| 1 | INCENP | Exon17 | frameshift insertion | c.2329dupG |
| 1 | INHBC | Exon2 | frameshift deletion | c.769delC |
| 2 | INHBC | Exon2 | frameshift deletion | c.853delG |
| 1 | INMT | Exon2 | stopgain SNV | c.318delG |
| 1 | INMT | Exon3 | stopgain SNV | c.364C>T |
| 16 | INMT | Exon3 | stoploss SNV | c.788G>C |
| 3 | INPP1 | Exon5 | splicing | c.266-2_266-2delA |
| 1 | INPP4B | Exon22 | stopgain SNV | c.2023G>T |
| 1 | INPP5D | Exon20 | stopgain SNV | c.2410C>T |
| 1 | INPP5J | Exon3 | splicing | c.168-2A>C |
| 1 | INPP5J | Exon6 | stopgain SNV | c.622C>T |
| 1 | INPP5K | Exon3 | frameshift deletion | c.228delC |
| 1 | INPPL1 | Exon26 | frameshift deletion | c.3011_3012delCT |
| 4 | INSC | Exon3 | stopgain SNV | c.364C>T |
| 1 | INSC | Exon6 | frameshift deletion | c.821delG |
| 1 | INSR | Exon14 | frameshift insertion | c.2736_2737insA |
| 1 | INSRR | Exon17 | stopgain SNV | c.2995C>T |
| 1 | INSRR | Exon9 | frameshift deletion | c.1888delC |
| 1 | INSRR | Exon6 | frameshift deletion | c.1232_1233delAC |
| 1 | INSRR | Exon4 | splicing | c.941+1G>T |
| 1 | INTS1 | Exon3 | frameshift deletion | c.144delA |
| 7 | INTS3 | Exon27 | stopgain SNV | c.2740A>T |
| 1 | INTU | Exon2 | frameshift deletion | c.310delA |
| 1 | IP6K2 | Exon3 | stopgain SNV | c.393G>A |
| 1 | IPO11 | Exon11 | splicing | c.1174+2T>G |
| 1 | IPO13 | Exon19 | frameshift insertion | c.2738_2739insCAGTA |
| 1 | IPO13 | Exon19 | frameshift deletion | c.2738delT |
| 2 | IPO5 | Exon6 | stopgain SNV | c.397G>T |
| 3 | IPO5 | Exon24 | splicing | c.2547+2T>C |
| 3 | IPO7 | Exon3 | frameshift deletion | c.224delC |
| 1 | IPO8 | Exon13 | stopgain SNV | c.1397T>G |
| 1 | IQCA1 | Exon2 | frameshift deletion | c.323delT |
| 1 | IQCE | Exon2 | splicing | c.82+1G>A |
| 2 | IQCE | Exon11 | frameshift deletion | c.847_856delCGGAGTGTCC |
| 1 | IQCE | Exon13 | frameshift insertion | c.1175_1176insT |
| 1 | IQCE | Exon13 | frameshift insertion | c.1181dupA |
| 1 | IQCF1 | Exon4 | stoploss SNV | c.616T>C |
| 1 | IQCF1 | Exon4 | stopgain SNV | c.295C>T |
| 1 | IQCF3 | Exon7 | stopgain SNV | c.200G>A |
| 8 | IQCF3 | Exon7 | stopgain SNV | c.217A>T |
| 1 | IQCG | Exon7 | stopgain SNV | c.655G>T |
| 2 | IQCH | Exon15 | stopgain SNV | c.2200C>T |
| 2 | IQGAP1 | Exon21 | frameshift deletion | c.2481delT |
| 10 | IQGAP1 | Exon31 | frameshift deletion | c.3948delT |
| 1 | IQGAP2 | Exon23 | stopgain SNV | c.2788C>T |
| 2 | IQGAP3 | Exon15 | splicing | c.1570+1G>A |
| 1 | IQGAP3 | Exon11 | frameshift deletion | c.1097delC |
| 3 | IRAK3 | Exon2 | splicing | c.198+1G>T |
| 1 | IRAK4 | Exon6 | stopgain SNV | c.505C>T |
| 3 | IRS1 | Exon1 | frameshift insertion | c.1791dupG |
| 1 | IRS2 | Exon1 | stopgain SNV | c.388C>T |
| 1 | IRS4 | Exon1 | frameshift insertion | c.1772dupG |
| 1 | IRX1 | Exon2 | frameshift deletion | c.403delA |
| 1 | IRX1 | Exon2 | frameshift deletion | c.588delC |
| 1 | IRX3 | Exon2 | frameshift insertion | c.706dupG |
| 1 | IRX4 | Exon5 | frameshift deletion | c.758delA |
| 1 | ISCU | Exon3 | frameshift deletion | c.278delT |
| 2 | ISM2 | Exon5 | stopgain SNV | c.978T>G |
| 1 | ISX | Exon2 | splicing | c.381+1G>A |
| 1 | ISYNA1 | Exon11 | stopgain SNV | c.1623C>A |
| 1 | ITGA10 | Exon9 | stopgain SNV | c.994C>T |
| 1 | ITGA10 | Exon12 | frameshift deletion | c.1377_1378delAA |
| 1 | ITGA1 | Exon11 | frameshift insertion | c.1215_1219dupTGTCA |
| 1 | ITGA2B | Exon24 | frameshift insertion | c.2375_2376insCT |
| 2 | ITGA2 | Exon3 | splicing | c.295+1G>C |
| 1 | ITGA4 | Exon24 | frameshift deletion | c.2577_2580delAGTG |
| 8 | ITGA5 | Exon28 | stopgain SNV | c.2920C>T |
| 1 | ITGA6 | Exon23 | stopgain SNV | c.2926C>T |
| 1 | ITGAD | Exon8 | stopgain SNV | c.736C>T |
| 2 | ITGAE | Exon7 | splicing | c.434-2_434-1insTTA |
| 3 | ITGAL | Exon27 | splicing | c.2976+2T>A |
| 1 | ITGAV | Exon15 | stopgain SNV | c.1507C>T |
| 1 | ITGAV | Exon18 | stopgain SNV | c.1930C>T |
| 1 | ITGB1BP2 | Exon11 | stopgain SNV | c.986C>G |
| 1 | ITGB3BP | Exon7 | frameshift deletion | c.454_457delTTTG |
| 2 | ITGB3 | Exon10 | frameshift insertion | c.1476dupG |
| 1 | ITGB6 | Exon14 | stopgain SNV | c.2245C>T |
| 1 | ITGB6 | Exon11 | frameshift deletion | c.1792delG |
| 1 | ITGB6 | Exon12 | splicing | c.1661-2A>G |
| 1 | ITGB6 | Exon2 | stopgain SNV | c.129G>A |
| 1 | ITGB7 | Exon12 | frameshift insertion | c.1654_1655insA |
| 1 | ITGB8 | Exon1 | stopgain SNV | c.49C>T |
| 1 | ITIH1 | Exon2 | stopgain SNV | c.121C>T |
| 1 | ITIH1 | Exon3 | frameshift deletion | c.300_301delTT |
| 1 | ITIH1 | Exon20 | stopgain SNV | c.2408G>A |
| 1 | ITIH2 | Exon4 | splicing | c.362+1G>C |
| 1 | ITIH6 | Exon11 | splicing | c.3238+2T>C |
| 13 | ITIH6 | Exon8 | splicing | c.1075+2dupT |
| 1 | ITIH6 | Exon7 | frameshift deletion | c.1026delG |
| 1 | ITM2A | Exon5 | frameshift deletion | c.558delC |
| 2 | ITPKC | Exon3 | splicing | c.1469+2T>G |
| 1 | ITPR3 | Exon25 | frameshift insertion | c.3178_3179insAT |
| 1 | ITPR3 | Exon26 | splicing | c.3283-1G>A |
| 1 | ITPRIPL1 | Exon3 | stopgain SNV | c.190C>T |
| 1 | ITPRIPL1 | Exon3 | stopgain SNV | c.1437G>A |
| 8 | ITSN1 | Exon29 | frameshift deletion | c.3514delG |
| 4 | ITSN2 | Exon20 | splicing | c.2257+1G>A |
| 1 | JAKMIP1 | Exon12 | stopgain SNV | c.1651A>T |
| 1 | JAKMIP3 | Exon15 | frameshift deletion | c.1950_1951delCA |
| 1 | JAKMIP3 | Exon15 | frameshift deletion | c.1955delT |
| 1 | JMJD4 | Exon6 | splicing | c.1107+1_1107+2insA |
| 5 | JUP | Exon8 | frameshift deletion | c.1494delC |
| 1 | KAAG1 | Exon1 | stopgain SNV | c.28G>T |
| 1 | KAL1 | Exon8 | stopgain SNV | c.1207A>T |
| 1 | KANK1 | Exon3 | stopgain SNV | c.972T>A |
| 1 | KANK2 | Exon11 | frameshift deletion | c.2380delG |
| 5 | KANK4 | Exon6 | splicing | c.2231+1_2231+1delG |
| 1 | KAT2A | Exon11 | frameshift deletion | c.1665_1666delGG |
| 2 | KAT6A | Exon18 | stopgain SNV | c.5263A>T |
| 1 | KAT7 | Exon2 | frameshift deletion | c.123delA |
| 1 | KAT7 | Exon12 | stopgain SNV | c.1450C>T |
| 1 | KATNA1 | Exon8 | stoploss SNV | c.935G>T |
| 1 | KATNAL2 | Exon9 | stopgain SNV | c.645G>A |
| 1 | KAZN | Exon2 | stopgain SNV | c.367C>T |
| 1 | KBTBD12 | Exon3 | stopgain SNV | c.1369C>T |
| 1 | KBTBD12 | Exon4 | frameshift deletion | c.1587_1590delGGGC |
| 1 | KBTBD3 | Exon4 | frameshift deletion | c.1065delT |
| 1 | KBTBD7 | Exon1 | frameshift deletion | c.486_487delCT |
| 1 | KBTBD7 | Exon1 | frameshift deletion | c.475_476delGC |
| 2 | KCMF1 | Exon7 | frameshift deletion | c.1002delA |
| 1 | KCNA1 | Exon2 | frameshift deletion | c.1129_1130delGA |
| 1 | KCNA1 | Exon2 | frameshift deletion | c.1135delT |
| 1 | KCNA3 | Exon1 | frameshift deletion | c.1495delT |
| 1 | KCNA6 | Exon1 | frameshift deletion | c.1422delA |
| 1 | KCNAB3 | Exon12 | splicing | c.927+1G>T |
| 1 | KCNAB3 | Exon7 | stopgain SNV | c.508C>T |
| 1 | KCND1 | Exon4 | frameshift insertion | c.1370dupA |
| 3 | KCND2 | Exon5 | stopgain SNV | c.1513G>T |
| 6 | KCNE1L | Exon1 | stopgain SNV | c.163A>T |
| 1 | KCNE2 | Exon2 | frameshift deletion | c.369_370delCT |
| 1 | KCNH1 | Exon2 | splicing | c.79+0_79+3dupGGTA |
| 1 | KCNH3 | Exon12 | frameshift deletion | c.2358delC |
| 1 | KCNH3 | Exon12 | frameshift insertion | c.2367_2368insA |
| 1 | KCNIP2 | Exon3 | stopgain SNV | c.265G>T |
| 1 | KCNJ11 | Exon2 | frameshift deletion | c.80delT |
| 1 | KCNJ15 | Exon4 | frameshift deletion | c.168delC |
| 1 | KCNJ15 | Exon4 | stopgain SNV | c.889C>T |
| 1 | KCNJ18 | Exon3 | frameshift insertion | c.719_720insT |
| 3 | KCNK16 | Exon5 | stopgain SNV | c.751C>T |
| 1 | KCNK16 | Exon2 | frameshift deletion | c.254_255delGC |
| 1 | KCNK18 | Exon3 | frameshift deletion | c.414_415delCT |
| 2 | KCNK1 | Exon1 | stopgain SNV | c.160C>T |
| 1 | KCNK4 | Exon5 | splicing | c.475-1G>A |
| 2 | KCNK4 | Exon5 | splicing | c.475-1_475-1delG |
| 1 | KCNK5 | Exon5 | frameshift insertion | c.746_747insGC |
| 2 | KCNMA1 | Exon20 | frameshift deletion | c.2144delA |
| 1 | KCNMB1 | Exon4 | frameshift deletion | c.357delC |
| 1 | KCNQ4 | Exon13 | frameshift deletion | c.1813delT |
| 2 | KCNQ5 | Exon14 | stopgain SNV | c.2513C>A |
| 2 | KCNRG | Exon1 | frameshift deletion | c.300delA |
| 1 | KCNT1 | Exon25 | frameshift deletion | c.2896delG |
| 7 | KCNT2 | Exon22 | stopgain SNV | c.2582C>G |
| 1 | KCNU1 | Exon27 | frameshift deletion | c.3392_3396delGGAAA |
| 1 | KCTD11 | Exon1 | frameshift deletion | c.85delG |
| 1 | KCTD12 | Exon1 | stoploss SNV | c.976T>A |
| 1 | KCTD17 | Exon8 | splicing | c.825-1G>T |
| 8 | KCTD18 | Exon7 | frameshift deletion | c.1159_1166delACCGCGCC |
| 1 | KDELC1 | Exon3 | frameshift deletion | c.421_422delCT |
| 1 | KDELR3 | Exon4 | frameshift deletion | c.371_374delTCTA |
| 2 | KDM1B | Exon10 | stopgain SNV | c.788T>G |
| 2 | KDM2B | Exon1 | frameshift deletion | c.83_84delCA |
| 2 | KDM3A | Exon22 | stopgain SNV | c.3400G>T |
| 1 | KDM3A | Exon22 | frameshift deletion | c.3411delA |
| 1 | KDM3B | Exon8 | frameshift deletion | c.1690_1691delCC |
| 2 | KDM5C | Exon24 | stopgain SNV | c.4107G>A |
| 1 | KDM5C | Exon21 | frameshift insertion | c.3699_3700insCC |
| 1 | KDM5C | Exon13 | frameshift deletion | c.2010delC |
| 5 | KDM6A | Exon9 | frameshift deletion | c.690delA |
| 1 | KEAP1 | Exon2 | frameshift insertion | c.242dupT |
| 1 | KEL | Exon3 | frameshift insertion | c.184dupT |
| 1 | KHDC1 | Exon5 | frameshift deletion | c.526_527delCT |
| 1 | KHDC1 | Exon5 | frameshift insertion | c.514_515insA |
| 1 | KIAA0020 | Exon8 | splicing | c.677+1G>A |
| 1 | KIAA0090 | Exon23 | splicing | c.2802+1_2802+1delG |
| 1 | KIAA0146 | Exon13 | frameshift deletion | c.1780delA |
| 1 | KIAA0182 | Exon15 | stopgain SNV | c.3308G>A |
| 1 | KIAA0195 | Exon20 | frameshift deletion | c.2618delC |
| 1 | KIAA0195 | Exon20 | frameshift deletion | c.2652delT |
| 1 | KIAA0195 | Exon28 | frameshift deletion | c.3577delA |
| 1 | KIAA0195 | Exon28 | frameshift deletion | c.3584delG |
| 1 | KIAA0196 | Exon13 | stopgain SNV | c.1582C>T |
| 1 | KIAA0319 | Exon4 | frameshift deletion | c.266delC |
| 1 | KIAA0368 | Exon1 | frameshift deletion | c.27_28delGA |
| 1 | KIAA0408 | Exon6 | stopgain SNV | c.2062C>T |
| 1 | KIAA0408 | Exon6 | frameshift deletion | c.1982delT |
| 1 | KIAA0415 | Exon11 | splicing | c.1312-2A>G |
| 1 | KIAA0430 | Exon11 | frameshift deletion | c.2433delG |
| 12 | KIAA0513 | Exon2 | stopgain SNV | c.325G>T |
| 1 | KIAA0528 | Exon23 | splicing | c.2447-2A>G |
| 16 | KIAA0528 | Exon14 | stopgain SNV | c.1564A>T |
| 1 | KIAA0556 | Exon15 | frameshift deletion | c.2373delC |
| 1 | KIAA0564 | Exon41 | frameshift deletion | c.5096delG |
| 1 | KIAA0564 | Exon36 | stopgain SNV | c.4408A>T |
| 1 | KIAA0564 | Exon20 | stopgain SNV | c.2333T>G |
| 1 | KIAA0564 | Exon18 | frameshift deletion | c.2132_2135delCTGA |
| 1 | KIAA0564 | Exon17 | splicing | c.1870-1G>C |
| 4 | KIAA0586 | Exon5 | frameshift deletion | c.428delG |
| 1 | KIAA0586 | Exon12 | splicing | c.1413-1G>C |
| 1 | KIAA0664 | Exon26 | frameshift deletion | c.3875delA |
| 1 | KIAA0753 | Exon18 | stopgain SNV | c.2686C>T |
| 1 | KIAA0753 | Exon10 | frameshift deletion | c.1784_1785delAA |
| 1 | KIAA0753 | Exon7 | stopgain SNV | c.1109T>A |
| 5 | KIAA0895 | Exon7 | frameshift deletion | c.1472_1473delTC |
| 1 | KIAA0907 | Exon10 | frameshift insertion | c.1254_1255insG |
| 2 | KIAA0907 | Exon10 | frameshift deletion | c.1253_1254delCT |
| 6 | KIAA0913 | Exon24 | frameshift insertion | c.5100dupC |
| 1 | KIAA0922 | Exon24 | splicing | c.2789+2T>C |
| 1 | KIAA1199 | Exon24 | frameshift insertion | c.3239dupT |
| 1 | KIAA1211 | Exon8 | frameshift deletion | c.924delT |
| 1 | KIAA1211 | Exon10 | frameshift deletion | c.3468delC |
| 7 | KIAA1239 | Exon2 | stopgain SNV | c.169C>T |
| 1 | KIAA1257 | Exon7 | frameshift deletion | c.914delC |
| 1 | KIAA1257 | Exon4 | frameshift deletion | c.509_510delTT |
| 1 | KIAA1279 | Exon3 | stopgain SNV | c.599C>A |
| 1 | KIAA1324L | Exon3 | frameshift deletion | c.409delG |
| 1 | KIAA1324L | Exon3 | frameshift deletion | c.398delG |
| 2 | KIAA1370 | Exon13 | frameshift deletion | c.3114delT |
| 20 | KIAA1407 | Exon9 | frameshift deletion | c.1245delC |
| 1 | KIAA1429 | Exon21 | stopgain SNV | c.4715C>G |
| 2 | KIAA1429 | Exon12 | splicing | c.2669-1G>A |
| 1 | KIAA1530 | Exon9 | frameshift deletion | c.1405delG |
| 1 | KIAA1549 | Exon13 | frameshift insertion | c.4480_4481insG |
| 1 | KIAA1549 | Exon2 | frameshift deletion | c.2660delG |
| 1 | KIAA1586 | Exon4 | frameshift insertion | c.391_392insCC |
| 1 | KIAA1586 | Exon4 | frameshift deletion | c.2090_2093delTTAG |
| 1 | KIAA1609 | Exon4 | splicing | c.154-1G>T |
| 1 | KIAA1614 | Exon5 | frameshift insertion | c.2703_2704insCA |
| 1 | KIAA1614 | Exon5 | frameshift deletion | c.2709delG |
| 1 | KIAA1731 | Exon24 | frameshift deletion | c.7051delT |
| 1 | KIAA1755 | Exon11 | splicing | c.2417+1G>T |
| 13 | KIAA1755 | Exon3 | stopgain SNV | c.1528C>T |
| 1 | KIAA1797 | Exon42 | splicing | c.4729-1G>T |
| 1 | KIAA1841 | Exon6 | stopgain SNV | c.561G>A |
| 1 | KIAA1841 | Exon21 | frameshift deletion | c.2082delT |
| 4 | KIAA1958 | Exon2 | frameshift deletion | c.111delT |
| 1 | KIAA2018 | Exon7 | frameshift deletion | c.3396delA |
| 1 | KIAA2018 | Exon7 | frameshift deletion | c.3047delA |
| 1 | KIF12 | Exon16 | stopgain SNV | c.1471C>T |
| 1 | KIF15 | Exon33 | stopgain SNV | c.3940G>T |
| 1 | KIF18A | Exon14 | stopgain SNV | c.2130delT |
| 1 | KIF18B | Exon14 | splicing | c.2285+2T>A |
| 1 | KIF1A | Exon36 | frameshift deletion | c.3604_3605delAT |
| 1 | KIF1A | Exon36 | frameshift deletion | c.3599delG |
| 2 | KIF1B | Exon38 | stopgain SNV | c.4151C>G |
| 1 | KIF20A | Exon7 | frameshift insertion | c.706dupG |
| 1 | KIF20B | Exon15 | frameshift deletion | c.1992delT |
| 1 | KIF20B | Exon20 | frameshift deletion | c.3533_3534delTT |
| 1 | KIF20B | Exon33 | stopgain SNV | c.5314C>T |
| 3 | KIF24 | Exon11 | stopgain SNV | c.3554G>A |
| 1 | KIF24 | Exon11 | splicing | c.1625+1G>A |
| 1 | KIF24 | Exon8 | frameshift deletion | c.1401_1404delGACA |
| 1 | KIF26B | Exon3 | frameshift insertion | c.509_510dupCC |
| 1 | KIF26B | Exon3 | frameshift insertion | c.627_628insA |
| 1 | KIF26B | Exon3 | frameshift insertion | c.859_860insT |
| 1 | KIF26B | Exon12 | frameshift insertion | c.3148dupC |
| 1 | KIF3A | Exon17 | stopgain SNV | c.2090T>G |
| 2 | KIF4A | Exon20 | splicing | c.2119-1G>T |
| 1 | KIF4B | Exon1 | frameshift insertion | c.1581dupT |
| 4 | KIF5A | Exon24 | frameshift deletion | c.2549delA |
| 7 | KIF5C | Exon19 | splicing | c.2240+2T>A |
| 7 | KIF6 | Exon11 | splicing | c.1181+2_1181+2delT |
| 1 | KIF6 | Exon8 | frameshift insertion | c.951_952insGC |
| 1 | KIF7 | Exon17 | stopgain SNV | c.3379C>T |
| 1 | KIFC3 | Exon8 | frameshift insertion | c.1064dupA |
| 2 | KIR2DL1 | Exon4 | stopgain SNV | c.621C>G |
| 1 | KIR2DL1 | Exon6 | frameshift deletion | c.809delA |
| 7 | KIR2DL1 | Exon6 | frameshift insertion | c.817dupA |
| 10 | KIR2DL4 | Exon6 | frameshift insertion | c.810dupA |
| 1 | KIR2DS4 | Exon3 | splicing | c.71-2A>G |
| 14 | KIR2DS4 | Exon7 | frameshift deletion | c.838_839delCA |
| 1 | KIR3DL1 | Exon3 | stopgain SNV | c.121C>T |
| 1 | KIR3DL1 | Exon9 | stopgain SNV | c.1283dupA |
| 1 | KIR3DL2 | Exon3 | frameshift deletion | c.320_321delCG |
| 1 | KIRREL2 | Exon15 | stopgain SNV | c.1721G>A |
| 1 | KIRREL3 | Exon17 | frameshift deletion | c.2273delA |
| 2 | KIT | Exon1 | frameshift deletion | c.3delG |
| 1 | KLHDC1 | Exon3 | stopgain SNV | c.246C>A |
| 1 | KLHDC4 | Exon7 | stopgain SNV | c.751C>T |
| 3 | KLHDC7A | Exon1 | stopgain SNV | c.754C>T |
| 1 | KLHDC8B | Exon4 | frameshift insertion | c.552_553insA |
| 1 | KLHL13 | Exon3 | frameshift deletion | c.65delA |
| 1 | KLHL1 | Exon1 | frameshift deletion | c.134delG |
| 1 | KLHL25 | Exon2 | frameshift deletion | c.1610delG |
| 5 | KLHL29 | Exon12 | frameshift insertion | c.2146dupT |
| 2 | KLHL32 | Exon6 | splicing | c.412-2A>C |
| 2 | KLHL32 | Exon6 | stopgain SNV | c.561T>G |
| 1 | KLHL38 | Exon1 | frameshift insertion | c.582_583insG |
| 1 | KLHL38 | Exon1 | frameshift deletion | c.578_579delAG |
| 2 | KLK14 | Exon3 | splicing | c.26+1G>A |
| 1 | KLK3 | Exon5 | stoploss SNV | c.786A>C |
| 2 | KLK4 | Exon5 | stoploss SNV | c.764A>C |
| 1 | KLK4 | Exon2 | stopgain SNV | c.190C>T |
| 1 | KLK7 | Exon6 | frameshift deletion | c.634delA |
| 2 | KLKB1 | Exon5 | frameshift insertion | c.451dupT |
| 3 | KLRC2 | Exon6 | frameshift deletion | c.676delC |
| 1 | KLRC3 | Exon7 | splicing | c.588-2A>G |
| 2 | KLRC3 | Exon6 | splicing | c.587+1G>T |
| 1 | KLRC4-KLRK1 | Exon11 | splicing | c.242-2A>G |
| 1 | KLRK1 | Exon8 | frameshift deletion | c.642delG |
| 1 | KNG1 | Exon7 | splicing | c.823-2A>G |
| 1 | KNTC1 | Exon7 | frameshift insertion | c.547dupA |
| 1 | KNTC1 | Exon16 | frameshift deletion | c.1234delG |
| 1 | KPNA1 | Exon10 | frameshift deletion | c.975_976delAG |
| 1 | KPNA4 | Exon4 | splicing | c.115-1G>A |
| 1 | KPNA5 | Exon6 | stopgain SNV | c.559C>T |
| 3 | KPNA7 | Exon8 | stopgain SNV | c.1213C>T |
| 2 | KPRP | Exon2 | frameshift insertion | c.1660dupC |
| 1 | KPRP | Exon2 | stoploss SNV | c.1738dupT |
[truncated: 247,881 more chars]
