# Supplementary material for: Germline whole exome sequencing and large-scale replication identifies FANCM as a likely high grade serous ovarian cancer susceptibility gene
Source: Oncotarget. 2017 Mar 3;8(31):50930–40. doi: 10.18632/oncotarget.15871 (PMC5584218; doi:10.18632/oncotarget.15871)
Supplement: Supplementary file 4 [file oncotarget-08-50930-s004.docx]

**Supplementary Table 3:** Pathway analysis of genes harbouring protein truncating mutations in 412 HGSOC cases

| **Pathway** | **Num. of**  **Genes** | **Normalized**  **Enrichment** | **P** | **FDR** |
| --- | --- | --- | --- | --- |
| DNA_REPAIR | 42 | 1.86 | 1.8E-03 | 0.26 |
| LIPID_BIOSYNTHETIC_PROCESS | 18 | 1.85 | 4.2E-03 | 0.16 |
| FATTY_ACID_METABOLIC_PROCESS | 24 | 1.78 | 8.3E-03 | 0.26 |
| POSITIVE_REGULATION_OF_PROTEIN_  METABOLIC_PROCESS | 15 | 1.75 | 1.0E-02 | 0.27 |
| RESPONSE_TO_ENDOGENOUS_STIMULUS | 63 | 1.75 | 2.7E-03 | 0.23 |
| RESPONSE_TO_DNA_DAMAGE_STIMULUS | 56 | 1.73 | 4.8E-03 | 0.22 |
| CELLULAR_LIPID_METABOLIC_PROCESS | 67 | 1.72 | 3.5E-03 | 0.21 |
| DNA_RECOMBINATION | 17 | 1.70 | 1.6E-02 | 0.22 |
| MONOCARBOXYLIC_ACID_METABOLIC_PROCESS | 29 | 1.70 | 1.4E-02 | 0.21 |
| NEGATIVE_REGULATION_OF_TRANSCRIPTION | 34 | 1.67 | 1.5E-02 | 0.24 |
| DNA_METABOLIC_PROCESS | 75 | 1.67 | 3.9E-03 | 0.22 |
| NEGATIVE_REGULATION_OF_  NUCLEOBASENUCLEOSIDENUCLEOTIDE_  AND_NUCLEIC_ACID_METABOLIC_PROCESS | 39 | 1.61 | 2.4E-02 | 0.33 |
| REGULATION_OF_CELL_PROLIFERATION | 63 | 1.58 | 1.9E-02 | 0.41 |
| POSITIVE_REGULATION_OF_DEVELOPMENTAL_PROCESS | 54 | 1.56 | 2.6E-02 | 0.43 |
| REGULATION_OF_CELLULAR_PROTEIN_METABOLIC_PROCESS | 37 | 1.54 | 4.5E-02 | 0.46 |
| NEGATIVE_REGULATION_OF_CELLULAR_METABOLIC_PROCESS | 50 | 1.53 | 3.7E-02 | 0.48 |
| CELL_PROLIFERATION_GO_0008283 | 93 | 1.53 | 1.7E-02 | 0.46 |
| REGULATION_OF_PROGRAMMED_CELL_DEATH | 73 | 1.52 | 2.6E-02 | 0.45 |
| REGULATION_OF_APOPTOSIS | 73 | 1.52 | 3.0E-02 | 0.43 |
| NEGATIVE_REGULATION_OF_METABOLIC_PROCESS | 51 | 1.52 | 4.2E-02 | 0.41 |
| RESPONSE_TO_CHEMICAL_STIMULUS | 78 | 1.50 | 3.1E-02 | 0.44 |
| REGULATION_OF_PROTEIN_METABOLIC_PROCESS | 39 | 1.49 | 6.5E-02 | 0.46 |
| MONOVALENT_INORGANIC_CATION_TRANSPORT | 23 | 1.48 | 8.1E-02 | 0.46 |
| NEGATIVE_REGULATION_OF_CELL_PROLIFERATION | 30 | 1.46 | 7.6E-02 | 0.49 |
| PROGRAMMED_CELL_DEATH | 90 | 1.46 | 3.5E-02 | 0.48 |
| LIPID_METABOLIC_PROCESS | 90 | 1.46 | 3.8E-02 | 0.47 |
| APOPTOSIS_GO | 90 | 1.46 | 3.8E-02 | 0.46 |
| REGULATION_OF_DEVELOPMENTAL_PROCESS | 98 | 1.43 | 4.2E-02 | 0.50 |
| CELLULAR_BIOSYNTHETIC_PROCESS | 67 | 1.43 | 6.6E-02 | 0.51 |
| CELL_CYCLE_PROCESS | 54 | 1.40 | 9.5E-02 | 0.58 |
| MITOSIS | 21 | 1.39 | 1.3E-01 | 0.60 |
| NEGATIVE_REGULATION_OF_CELLULAR_PROCESS | 133 | 1.38 | 4.1E-02 | 0.59 |
| RESPONSE_TO_STRESS | 135 | 1.38 | 4.0E-02 | 0.59 |
| ORGANIC_ACID_METABOLIC_PROCESS | 52 | 1.37 | 1.1E-01 | 0.59 |
| CARBOXYLIC_ACID_METABOLIC_PROCESS | 52 | 1.37 | 1.1E-01 | 0.58 |
| CELL_DEVELOPMENT | 116 | 1.35 | 6.9E-02 | 0.64 |
| CHROMOSOME_ORGANIZATION_AND_BIOGENESIS | 31 | 1.35 | 1.5E-01 | 0.63 |
| M_PHASE_OF_MITOTIC_CELL_CYCLE | 23 | 1.35 | 1.5E-01 | 0.61 |
| ION_TRANSPORT | 50 | 1.32 | 1.5E-01 | 0.69 |
| SECRETORY_PATHWAY | 17 | 1.31 | 1.9E-01 | 0.69 |
| NEGATIVE_REGULATION_OF_BIOLOGICAL_PROCESS | 142 | 1.31 | 7.8E-02 | 0.67 |
| INTRACELLULAR_SIGNALING_CASCADE | 170 | 1.31 | 6.1E-02 | 0.66 |
| APOPTOTIC_PROGRAM | 16 | 1.30 | 1.9E-01 | 0.68 |
| PROTEIN_AMINO_ACID_AUTOPHOSPHORYLATION | 15 | 1.30 | 2.0E-01 | 0.67 |
| PROTEIN_AUTOPROCESSING | 15 | 1.29 | 2.1E-01 | 0.68 |
| CATION_TRANSPORT | 39 | 1.29 | 1.8E-01 | 0.67 |
| RNA_PROCESSING | 32 | 1.28 | 2.0E-01 | 0.69 |
| METAL_ION_TRANSPORT | 33 | 1.28 | 1.9E-01 | 0.68 |
| POST_TRANSLATIONAL_PROTEIN_MODIFICATION | 118 | 1.27 | 1.2E-01 | 0.69 |
| ORGANELLE_ORGANIZATION_AND_BIOGENESIS | 134 | 1.27 | 1.2E-01 | 0.68 |
| REPRODUCTIVE_PROCESS | 33 | 1.27 | 2.1E-01 | 0.67 |
| M_PHASE | 31 | 1.26 | 2.2E-01 | 0.68 |
| COFACTOR_METABOLIC_PROCESS | 17 | 1.25 | 2.3E-01 | 0.69 |
| DEPHOSPHORYLATION | 21 | 1.25 | 2.3E-01 | 0.68 |
| MEMBRANE_LIPID_METABOLIC_PROCESS | 17 | 1.24 | 2.5E-01 | 0.71 |
| TRANSMEMBRANE_RECEPTOR_PROTEIN_TYROSINE_  KINASE_SIGNALING_PATHWAY | 20 | 1.23 | 2.6E-01 | 0.74 |
| PROTEIN_PROCESSING | 17 | 1.19 | 2.9E-01 | 0.84 |
| SECRETION | 39 | 1.19 | 2.7E-01 | 0.83 |
| NEGATIVE_REGULATION_OF_CATALYTIC_ACTIVITY | 20 | 1.18 | 2.9E-01 | 0.85 |
| SECRETION_BY_CELL | 25 | 1.16 | 3.2E-01 | 0.91 |
| POSITIVE_REGULATION_OF_CELLULAR_METABOLIC_PROCESS | 51 | 1.15 | 3.1E-01 | 0.92 |
| BIOSYNTHETIC_PROCESS | 104 | 1.15 | 2.8E-01 | 0.91 |
| REGULATION_OF_TRANSCRIPTION | 117 | 1.15 | 2.7E-01 | 0.91 |
| CELL_CYCLE_GO_0007049 | 85 | 1.15 | 2.9E-01 | 0.90 |
| REGULATION_OF_CELLULAR_COMPONENT_  ORGANIZATION_AND_BIOGENESIS | 32 | 1.14 | 3.3E-01 | 0.91 |
| REGULATION_OF_NUCLEOBASENUCLEOSIDENUCLEOTIDE_  AND_NUCLEIC_ACID_METABOLIC_PROCESS | 128 | 1.13 | 2.9E-01 | 0.92 |
| PROTEIN_AMINO_ACID_DEPHOSPHORYLATION | 18 | 1.13 | 3.4E-01 | 0.91 |
| REGULATION_OF_CATALYTIC_ACTIVITY | 68 | 1.13 | 3.3E-01 | 0.90 |
| POSITIVE_REGULATION_OF_METABOLIC_PROCESS | 53 | 1.13 | 3.3E-01 | 0.89 |
| VESICLE_MEDIATED_TRANSPORT | 54 | 1.13 | 3.3E-01 | 0.88 |
| STEROID_METABOLIC_PROCESS | 19 | 1.12 | 3.5E-01 | 0.88 |
| LOCOMOTORY_BEHAVIOR | 20 | 1.12 | 3.5E-01 | 0.87 |
| NUCLEOBASENUCLEOSIDENUCLEOTIDE_AND_NUCLEIC_  ACID_METABOLIC_PROCESS | 272 | 1.12 | 2.4E-01 | 0.86 |
| REGULATION_OF_MOLECULAR_FUNCTION | 81 | 1.12 | 3.3E-01 | 0.87 |
| SIGNAL_TRANSDUCTION | 393 | 1.11 | 2.3E-01 | 0.88 |
| TRANSPORT | 209 | 1.11 | 2.9E-01 | 0.87 |
| ACTIN_CYTOSKELETON_ORGANIZATION_AND_BIOGENESIS | 28 | 1.10 | 3.6E-01 | 0.87 |
| POSITIVE_REGULATION_OF_SIGNAL_TRANSDUCTION | 27 | 1.10 | 3.8E-01 | 0.88 |
| RAS_PROTEIN_SIGNAL_TRANSDUCTION | 22 | 1.10 | 3.7E-01 | 0.87 |
| CELL_CYCLE_PHASE | 43 | 1.09 | 3.8E-01 | 0.87 |
| MITOTIC_CELL_CYCLE | 37 | 1.08 | 3.9E-01 | 0.88 |
| ESTABLISHMENT_OF_LOCALIZATION | 227 | 1.08 | 3.4E-01 | 0.89 |
| REGULATION_OF_PROTEIN_KINASE_ACTIVITY | 36 | 1.07 | 4.0E-01 | 0.89 |
| REGULATION_OF_SIGNAL_TRANSDUCTION | 50 | 1.07 | 4.0E-01 | 0.89 |
| REGULATION_OF_KINASE_ACTIVITY | 37 | 1.07 | 4.0E-01 | 0.88 |
| IMMUNE_RESPONSE | 43 | 1.07 | 4.0E-01 | 0.88 |
| REGULATION_OF_TRANSFERASE_ACTIVITY | 37 | 1.07 | 4.0E-01 | 0.87 |
| REGULATION_OF_GENE_EXPRESSION | 144 | 1.06 | 3.9E-01 | 0.87 |
| MUSCLE_DEVELOPMENT | 21 | 1.06 | 4.2E-01 | 0.88 |
| REGULATION_OF_TRANSCRIPTION_FROM_RNA_  POLYMERASE_II_PROMOTER | 55 | 1.05 | 4.2E-01 | 0.88 |
| BIOPOLYMER_MODIFICATION | 172 | 1.05 | 4.1E-01 | 0.87 |
| REPRODUCTION | 54 | 1.05 | 4.3E-01 | 0.87 |
| POSITIVE_REGULATION_OF_HYDROLASE_ACTIVITY | 16 | 1.05 | 4.2E-01 | 0.86 |
| REGULATION_OF_RNA_METABOLIC_PROCESS | 97 | 1.05 | 4.3E-01 | 0.86 |
| PROTEIN_MODIFICATION_PROCESS | 167 | 1.05 | 4.2E-01 | 0.85 |
| REGULATION_OF_CELLULAR_METABOLIC_PROCESS | 168 | 1.04 | 4.2E-01 | 0.85 |
| REGULATION_OF_METABOLIC_PROCESS | 170 | 1.03 | 4.3E-01 | 0.86 |
| SMALL_GTPASE_MEDIATED_SIGNAL_TRANSDUCTION | 26 | 1.02 | 4.6E-01 | 0.89 |
| POSITIVE_REGULATION_OF_CELLULAR_PROCESS | 152 | 1.02 | 4.7E-01 | 0.89 |
| ACTIN_FILAMENT_BASED_PROCESS | 33 | 1.01 | 4.8E-01 | 0.90 |
| G_PROTEIN_COUPLED_RECEPTOR_  PROTEIN_SIGNALING_PATHWAY | 74 | 1.00 | 5.0E-01 | 0.92 |
| REGULATION_OF_HYDROLASE_ACTIVITY | 25 | 0.99 | 4.9E-01 | 0.92 |
| MAPKKK_CASCADE_GO_0000165 | 25 | 0.99 | 4.9E-01 | 0.91 |
| ENZYME_LINKED_RECEPTOR_PROTEIN_SIGNALING_PATHWAY | 36 | 0.99 | 4.9E-01 | 0.91 |
| TRANSCRIPTION | 155 | 0.99 | 5.3E-01 | 0.91 |
| REGULATION_OF_TRANSCRIPTIONDNA_DEPENDENT | 94 | 0.98 | 5.3E-01 | 0.91 |
| IMMUNE_SYSTEM_PROCESS | 67 | 0.98 | 5.2E-01 | 0.92 |
| POSITIVE_REGULATION_OF_BIOLOGICAL_PROCESS | 160 | 0.97 | 5.5E-01 | 0.92 |
| POSITIVE_REGULATION_OF_CATALYTIC_ACTIVITY | 41 | 0.97 | 5.2E-01 | 0.91 |
| CHROMATIN_MODIFICATION | 16 | 0.96 | 5.2E-01 | 0.92 |
| ESTABLISHMENT_AND_OR_MAINTENANCE_  OF_CHROMATIN_ARCHITECTURE | 19 | 0.96 | 5.2E-01 | 0.91 |
| CAMP_MEDIATED_SIGNALING | 15 | 0.96 | 5.2E-01 | 0.91 |
| SYSTEM_PROCESS | 156 | 0.96 | 5.9E-01 | 0.91 |
| G_PROTEIN_SIGNALING_COUPLED_TO_CAMP_  NUCLEOTIDE_SECOND_MESSENGER | 15 | 0.96 | 5.3E-01 | 0.91 |
| BIOPOLYMER_METABOLIC_PROCESS | 401 | 0.95 | 6.4E-01 | 0.91 |
| I_KAPPAB_KINASE_NF_KAPPAB_CASCADE | 26 | 0.95 | 5.4E-01 | 0.90 |
| BEHAVIOR | 32 | 0.93 | 5.6E-01 | 0.93 |
| PROTEIN_AMINO_ACID_PHOSPHORYLATION | 74 | 0.93 | 5.9E-01 | 0.93 |
| CYTOSKELETON_ORGANIZATION_AND_BIOGENESIS | 70 | 0.92 | 6.0E-01 | 0.93 |
| RNA_METABOLIC_PROCESS | 174 | 0.91 | 6.7E-01 | 0.95 |
| PHOSPHORYLATION | 81 | 0.91 | 6.2E-01 | 0.95 |
| RIBONUCLEOPROTEIN_COMPLEX_  BIOGENESIS_AND_ASSEMBLY | 15 | 0.87 | 6.1E-01 | 1.00 |
| SENSORY_PERCEPTION | 61 | 0.86 | 6.7E-01 | 1.00 |
| CELL_SURFACE_RECEPTOR_LINKED_  SIGNAL_TRANSDUCTION_GO_0007166 | 143 | 0.85 | 7.5E-01 | 1.00 |
| CELLULAR_DEFENSE_RESPONSE | 20 | 0.85 | 6.4E-01 | 1.00 |
| POSITIVE_REGULATION_OF_TRANSFERASE_ACTIVITY | 20 | 0.85 | 6.5E-01 | 1.00 |
| REGULATION_OF_RESPONSE_TO_STIMULUS | 16 | 0.84 | 6.4E-01 | 1.00 |
| CELLULAR_LOCALIZATION | 96 | 0.84 | 7.2E-01 | 1.00 |
| CHEMICAL_HOMEOSTASIS | 32 | 0.84 | 6.6E-01 | 1.00 |
| SECOND_MESSENGER_MEDIATED_SIGNALING | 39 | 0.84 | 6.7E-01 | 1.00 |
| PROTEIN_KINASE_CASCADE | 66 | 0.84 | 7.1E-01 | 1.00 |
| ESTABLISHMENT_OF_CELLULAR_LOCALIZATION | 87 | 0.83 | 7.3E-01 | 1.00 |
| MEMBRANE_ORGANIZATION_AND_BIOGENESIS | 43 | 0.83 | 6.9E-01 | 1.00 |
| TRANSCRIPTION_DNA_DEPENDENT | 130 | 0.83 | 7.7E-01 | 1.00 |
| RNA_BIOSYNTHETIC_PROCESS | 131 | 0.82 | 7.8E-01 | 1.00 |
| CELLULAR_PROTEIN_METABOLIC_PROCESS | 267 | 0.82 | 8.5E-01 | 0.99 |
| ANTI_APOPTOSIS | 22 | 0.82 | 6.7E-01 | 0.98 |
| CELLULAR_MACROMOLECULE_  METABOLIC_PROCESS | 272 | 0.80 | 8.9E-01 | 1.00 |
| EXTRACELLULAR_STRUCTURE_  ORGANIZATION_AND_BIOGENESIS | 15 | 0.80 | 7.0E-01 | 1.00 |
| REGULATION_OF_BIOLOGICAL_QUALITY | 96 | 0.80 | 7.8E-01 | 1.00 |
| SEXUAL_REPRODUCTION | 29 | 0.79 | 7.1E-01 | 1.00 |
| CELL_CELL_SIGNALING | 77 | 0.79 | 7.7E-01 | 1.00 |
| CYCLIC_NUCLEOTIDE_MEDIATED_SIGNALING | 22 | 0.79 | 7.1E-01 | 0.99 |
| RESPONSE_TO_EXTERNAL_STIMULUS | 72 | 0.78 | 7.7E-01 | 0.99 |
| G_PROTEIN_SIGNALING_COUPLED_TO_CYCLIC_  NUCLEOTIDE_SECOND_MESSENGER | 22 | 0.78 | 7.1E-01 | 0.99 |
| TRANSCRIPTION_FROM_RNA_  POLYMERASE_II_PROMOTER | 89 | 0.78 | 8.0E-01 | 0.98 |
| DNA_DEPENDENT_DNA_REPLICATION | 17 | 0.78 | 7.2E-01 | 0.98 |
| CELLULAR_COMPONENT_ASSEMBLY | 63 | 0.77 | 7.7E-01 | 0.98 |
| PROTEIN_METABOLIC_PROCESS | 295 | 0.75 | 9.4E-01 | 1.00 |
| DNA_REPLICATION | 31 | 0.75 | 7.6E-01 | 1.00 |
| CELLULAR_CATION_HOMEOSTASIS | 23 | 0.75 | 7.5E-01 | 0.99 |
| CELL_MIGRATION | 26 | 0.74 | 7.6E-01 | 0.99 |
| CATION_HOMEOSTASIS | 24 | 0.74 | 7.6E-01 | 0.99 |
| DEFENSE_RESPONSE | 72 | 0.74 | 8.2E-01 | 0.98 |
| POSITIVE_REGULATION_OF_I_KAPPAB_  KINASE_NF_KAPPAB_CASCADE | 18 | 0.74 | 7.5E-01 | 0.98 |
| RESPONSE_TO_WOUNDING | 45 | 0.73 | 8.0E-01 | 0.98 |
| MACROMOLECULE_LOCALIZATION | 68 | 0.72 | 8.3E-01 | 0.98 |
| TISSUE_DEVELOPMENT | 44 | 0.72 | 8.1E-01 | 0.98 |
| HOMEOSTATIC_PROCESS | 47 | 0.72 | 8.1E-01 | 0.97 |
| PROTEIN_TARGETING | 30 | 0.71 | 8.0E-01 | 0.98 |
| NEGATIVE_REGULATION_OF_APOPTOSIS | 28 | 0.70 | 8.1E-01 | 0.98 |
| NEGATIVE_REGULATION_OF_  PROGRAMMED_CELL_DEATH | 28 | 0.70 | 8.0E-01 | 0.97 |
| RESPONSE_TO_ABIOTIC_STIMULUS | 32 | 0.70 | 8.1E-01 | 0.97 |
| PROTEIN_LOCALIZATION | 61 | 0.69 | 8.5E-01 | 0.98 |
| NEUROLOGICAL_SYSTEM_PROCESS | 109 | 0.68 | 9.0E-01 | 0.98 |
| PROTEOLYSIS | 41 | 0.68 | 8.5E-01 | 0.98 |
| REGULATION_OF_I_KAPPAB_  KINASE_NF_KAPPAB_CASCADE | 20 | 0.66 | 8.4E-01 | 0.99 |
| INFLAMMATORY_RESPONSE | 31 | 0.66 | 8.5E-01 | 0.98 |
| GENERATION_OF_PRECURSOR_  METABOLITES_AND_ENERGY | 34 | 0.65 | 8.7E-01 | 0.99 |
| CELLULAR_CATABOLIC_PROCESS | 48 | 0.64 | 8.9E-01 | 0.99 |
| SKELETAL_DEVELOPMENT | 20 | 0.63 | 8.6E-01 | 0.99 |
| MICROTUBULE_BASED_PROCESS | 33 | 0.61 | 9.0E-01 | 1.00 |
| REGULATION_OF_TRANSLATION | 22 | 0.60 | 9.0E-01 | 1.00 |
| ION_HOMEOSTASIS | 28 | 0.59 | 9.0E-01 | 1.00 |
| NEGATIVE_REGULATION_OF_  DEVELOPMENTAL_PROCESS | 41 | 0.59 | 9.2E-01 | 1.00 |
| CATABOLIC_PROCESS | 53 | 0.58 | 9.3E-01 | 1.00 |
| ORGAN_DEVELOPMENT | 144 | 0.58 | 9.8E-01 | 1.00 |
| ANATOMICAL_STRUCTURE_DEVELOPMENT | 245 | 0.55 | 9.9E-01 | 1.00 |
| INTRACELLULAR_TRANSPORT | 68 | 0.54 | 9.6E-01 | 1.00 |
| INTRACELLULAR_PROTEIN_TRANSPORT | 38 | 0.54 | 9.5E-01 | 1.00 |
| ANATOMICAL_STRUCTURE_MORPHOGENESIS | 93 | 0.54 | 9.7E-01 | 0.99 |
| CELLULAR_CARBOHYDRATE_METABOLIC_PROCESS | 30 | 0.53 | 9.5E-01 | 0.99 |
| CELLULAR_HOMEOSTASIS | 39 | 0.53 | 9.5E-01 | 0.99 |
| ANATOMICAL_STRUCTURE_FORMATION | 17 | 0.49 | 9.6E-01 | 1.00 |
| SYNAPTIC_TRANSMISSION | 47 | 0.49 | 9.7E-01 | 0.99 |
| ANGIOGENESIS | 16 | 0.49 | 9.6E-01 | 0.99 |
| FEMALE_PREGNANCY | 15 | 0.44 | 9.8E-01 | 1.00 |
| TRANSMISSION_OF_NERVE_IMPULSE | 49 | 0.44 | 9.9E-01 | 0.99 |
| TRANSLATION | 36 | 0.40 | 9.9E-01 | 0.99 |
