# Supplementary material for: Germline whole exome sequencing and large-scale replication identifies FANCM as a likely high grade serous ovarian cancer susceptibility gene
Source: Oncotarget. 2017 Mar 3;8(31):50930–40. doi: 10.18632/oncotarget.15871 (PMC5584218; doi:10.18632/oncotarget.15871)
Supplement: Supplementary file 5 [file oncotarget-08-50930-s005.docx]

**Supplementary Table 5:** Predicted deleterious truncating mutations identified in 11 candidate genes

| **cDNA** | **Protein** | **Mutation type** | **Exon/intron** | **cases** | **controls** |
| --- | --- | --- | --- | --- | --- |
| ***APEX1*** | | | |  |  |
| c.182_183delCA | p.T61fs | frameshift | Exon 3 | 0 | 1 |
| c.457C>T | p.Q153* | nonsense | Exon 5 | 1 | 0 |
| c.872dupT | p.L291fs | frameshift | Exon 5 | 2 | 2 |
| All |  |  |  | **3** | **3** |
| ***APLF*** | | | |  |  |
| c.22C>T | p.Q8* | nonsense | Exon 1 | 0 | 1 |
| c.64G>T | p.E22* | nonsense | Exon 1 | 0 | 1 |
| c.157C>T | p.R53* | nonsense | Exon 2 | 0 | 1 |
| c.168+5A>G | In frame 24 aa del | splicing | Intron2 | 0 | 1 |
| c.202G>T | p.E68* | nonsense | Exon 3 | 1 | 0 |
| c.293delT | p.F98fs | frameshift | Exon 3 | 1 | 0 |
| c.342-1G>A | Stop at aa 117 | splicing | Intron 3 | 1 | 0 |
| c.688C>T | p.Q230* | nonsense | Exon 6 | 0 | 1 |
| c.703C>T | p.Q235* | nonsense | Exon 6 | 0 | 1 |
| c.1160+1G>C | Stop at aa 278 | splicing | Intron 7 | 0 | 2 |
| c.1287-2A>G | Stop at aa 435 | splicing | Intron 8 | 4 | 2 |
| c.1288A>T | p.K430* | nonsense | Exon 9 | 1 | 0 |
| c.1333+2T>C | Stop at aa 435 | splicing | Intron9 | 1 | 0 |
| c.1477dupG | p.E493fs | frameshift | Exon 10 | 0 | 3 |
| c.1492G>T | p.E498* | nonsense | Exon 10 | 0 | 3 |
| c.1528delA | p.R510fs | frameshift | Exon 10 | 84 | 111 |
| All |  |  |  | **93** | **127** |
| ***APTX*** | | | |  |  |
| c.133G>A | p.V45I / no start codon | splicing | Exon 3 | 1 | 0 |
| c.229C>T | p.Q77* | nonsense | Exon 5 | 1 | 0 |
| c.347delA | p.K116fs | frameshift | Exon 5 | 0 | 1 |
| c.771-5A>G | Stop at aa 267 | splicing | Intron 7 | 1 | 0 |
| c.837G>A | p.W279* | nonsense | Exon 8 | 2 | 2 |
| c.874+2T>C | Stop at aa 267 | splicing | Intron 8 | 0 | 1 |
| c.916C>T | p.R306* | nonsense | Exon 9 | 1 | 0 |
| All |  |  |  | **6** | **4** |
| ***EME1*** | | | |  |  |
| c.1386-2A>G | Stop at aa 488 | splicing | Intron 7 | 3 | 1 |
| c.1393C>T | p.R465* | nonsense | Exon 8 | 0 | 1 |
| c.1720C>T | p.Q574* | nonsense | Exon 9 | 0 | 1 |
| c.1724delC | p.P575fs | frameshift | Exon 9 | 1 | 0 |
| All |  |  |  | **4** | **3** |
| ***FANCL*** | | | |  |  |
| c.170G>A | p.W57* | nonsense | Exon 3 | 1 | 0 |
| c.211C>T | p.Q71* | nonsense | Exon 3 | 0 | 1 |
| c.335C>A | p.S112* | nonsense | Exon 5 | 0 | 1 |
| c.378delT | p.L126fs | frameshift | Exon 6 | 1 | 0 |
| c.707-2A>G | Stop at aa 274 | splicing | Intron 9 | 1 | 0 |
| c.1111_1114dupATTA | p.T372fs | frameshift | Exon 14 | 23 | 27 |
| All |  |  |  | **26** | **29** |
| ***MAD2L2*** | | | |  |  |
| c.594G>T | p.K198N | splicing | Exon 8 | 1 | 0 |
| All |  |  |  | **1** | **0** |
| ***PARP2*** | | | |  |  |
| c.44G>A | p.R15K / no start codon | splicing | Exon 1 | 1 | 0 |
| c.364-6A>G | Stop at aa 196 | splicing | Intron4 | 1 | 0 |
| c.941+1G>A | Stop at aa 274 | splicing | Intron9 | 0 | 1 |
| c.991dupA | p.I331fs | frameshift | Exon 10 | 0 | 1 |
| c.1049_1050delAG | p.E350fs | frameshift | Exon 11 | 0 | 2 |
| c.1269-13G>A | Stop at aa 446 | splicing | Intron12 | 1 | 0 |
| c.1330C>T | p.R444* | nonsense | Exon 13 | 1 | 0 |
| c.1369-2A>G | In frame 33 aa del | splicing | Intron 13 | 1 | 0 |
| c.1480C>T | p.Q494* | nonsense | Exon 15 | 0 | 1 |
| All |  |  |  | **5** | **5** |
| ***PARP3*** | | | |  |  |
| c.334-1G>T | In frame 63 aa del | splicing | Intron 3 | 0 | 1 |
| c.523-1G>A | Stop at aa 176 | splicing | Intron 4 | 0 | 3 |
| c.656-2A>G | Stop at aa 234 | splicing | Intron 5 | 1 | 0 |
| c.970C>T | p.R324* | nonsense | Exon 7 | 1 | 0 |
| c.1039C>T | p.Q347* | nonsense | Exon 8 | 6 | 8 |
| c.1297+4A>G | Stop at aa 377 | splicing | Intron 9 | 0 | 1 |
| All |  |  |  | **8** | **13** |
| ***POLN*** | | | |  |  |
| c.133+5_133+8delGTAA | No start codon | splicing | Intron 1 | 0 | 2 |
| c.213+1G>T | Stop at aa 47 | splicing | Intron 2 | 0 | 1 |
| c.628C>T | p.Q210* | nonsense | Exon 3 | 1 | 0 |
| c.714G>A | p.Q238Q / In frame 168 aa del | splicing | Exon 3 | 1 | 0 |
| c.767_768delAG | p.E256fs | frameshift | Exon 4 | 1 | 0 |
| c.1179+1G>A | Stop at aa 346 | splicing | Intron 6 | 3 | 2 |
| c.1248+1G>T | In frame 23 aa del | splicing | Intron 7 | 1 | 1 |
| c.1375-2A>G | In frame 28 aa del | splicing | Intron 9 | 1 | 0 |
| c.1457A>T | p.E486V | splicing | Exon 10 | 1 | 1 |
| c.1795G>T | p.E599* | nonsense | Exon 16 | 1 | 0 |
| c.2065G>C | p.G689R / Stop at aa 724 | splicing | Exon 18 | 1 | 0 |
| c.2308+1G>A | In frame 37 aa del | splicing | Intron 20 | 1 | 1 |
| c.2387+1G>A | Stop at aa 818 | splicing | Intron 21 | 1 | 0 |
| c.2455+1G>A | Stop at aa 849 | splicing | Intron 22 | 0 | 1 |
| c.2456-6C>G | Stop at aa 851 | splicing | Intron 22 | 0 | 1 |
| c.2509delC | p.Q837fs | frameshift | Exon 23 | 11 | 19 |
| c.2643delT | p.P881fs | frameshift | Exon 24 | 5 | 1 |
| All |  |  |  | **29** | **30** |
| ***RAD54L*** | | | |  |  |
| c.407+1G>A | Stop at aa 116 | splicing | Intron5 | 1 | 0 |
| c.678G>A | p.W226* | nonsense | Exon 7 | 0 | 1 |
| c.748G>T | p.E250* | nonsense | Exon 7 | 0 | 1 |
| c.767-2A>G | Stop at aa 263 | splicing | Intron7 | 1 | 0 |
| c.1093C>T | p.R365* | nonsense | Exon 10 | 1 | 1 |
| c.1525C>T | p.R509* | nonsense | Exon 14 | 0 | 2 |
| c.1610+1G>A | Stop at aa 515 | splicing | Intron14 | 2 | 1 |
| c.1897C>T | p.Q633* | nonsense | Exon 17 | 1 | 0 |
| c.2033G>C | p.R678T / Stop at aa 640 | splicing | Exon 17 | 1 | 0 |
| c.2139G>A | p.W713* | nonsense | Exon 18 | 1 | 0 |
| All |  |  |  | **8** | **6** |
| ***SMUG1*** | | | |  |  |
| c.7C>T | p.Q3* | nonsense | Exon 3 | 3 | 2 |
| c.143delC | p.S48fs | frameshift | Exon 3 | 0 | 1 |
| c.370C>T | p.R124* | nonsense | Exon 4 | 1 | 0 |
| All |  |  |  | **4** | **3** |
| Protein indicates known protein changes and for splicing variants it shows that predictions of the position of the premature stop or whether the loss of the closest exon would be predicted to cause an in-frame deletion. For some missense changes that may also affect splicing missense change and effect of splicing is shown | | | | | |
