# Supplementary material for: Germline whole exome sequencing and large-scale replication identifies FANCM as a likely high grade serous ovarian cancer susceptibility gene
Source: Oncotarget. 2017 Mar 3;8(31):50930–40. doi: 10.18632/oncotarget.15871 (PMC5584218; doi:10.18632/oncotarget.15871)
Supplement: Supplementary file 6 [file oncotarget-08-50930-s006.docx]

**Supplementary Table 6:** Predicted deleterious truncating mutations identified in *FANCM*

|  | **Mutation details** | | |  | **Patient details** | | | |
| --- | --- | --- | --- | --- | --- | --- | --- | --- |
|  | **Nucl. Change** | **Type** | **Predicted truncation** | **Exon** | **Age at dx** | **Tumor histology** | **OC FH** | **BC FH** |
| case | c.448C>T | nonsense | p Q150X | 1 | 47 | HGS | N/A | N/A |
| case | c.466C>T | nonsense | p.Q156* | 1 | 78 | HGS | No | Yes |
| control | c.466C>T | nonsense | p.Q156* | 1 | 62 | N/A | N/A | N/A |
| case | c.918+2T>A | splicing | In frame 53  aa del | 4 | 73 | HGS | No | No |
| case | c.1309+5G>T | splicing | Stop at aa 397 | 7 | 47 | HGS | No | No |
| control | c.1309+5G>T | splicing | Stop at aa 397 | 7 | 64 | N/A | N/A | N/A |
| case | c.1491dupA | frameshift | p.S497fs | 9 | 52 | HGS | No | No |
| case | c.1492C>T | nonsense | p.Q498* | 9 | 62 | unknown | No | No |
| case | c.1581+1G>A | splicing | Stop at aa 472 | 9 | 65 | HGS | Yes | No |
| case | c.1777C>T | nonsense | p.R593* | 10 | 66 | HGS | No | No |
| control | c.1777C>T | nonsense | p.R593* | 10 | 39 | N/A | N/A | N/A |
| case | c.1972C>T | nonsense | p.R658* | 11 | 69 | HGS | No | No |
| case | c.1972C>T | nonsense | p.R658* | 11 | 63 | HGS | No | No |
| control | c.1972C>T | nonsense | p.R658* | 11 | 33 | N/A | N/A | N/A |
| control | c.1972C>T | nonsense | p.R658* | 11 | 55 | N/A | N/A | N/A |
| case | c.2160+5G>A | splicing | Stop at aa 682 | 12 | 52 | HGS | No | No |
| case | c.2356C>T | nonsense | p.Q786* | 14 | 60 | HGS | No | No |
| case | c.2578G>T | nonsense | p.E860* | 14 | 66 | HGS | No | No |
| case | c.4194T>G | nonsense | p Y1398* | 14 | 63 | HGS | N/A | N/A |
| case | c.4194T>G | nonsense | p.Q1554* | 18 | 55 | clear cell | N/A | N/A |
| case | c.4853C>G | nonsense | p.S1618* | 20 | 58 | HGS | Yes | Yes |
| case | c.4923T>G | nonsense | p.Y1641* | 20 | 64 | LGS | No | Yes |
| case | c.5101C>T | nonsense | p.Q1701* | 20 | 75 | HGS | No | Yes |
| case | c.5101C>T | nonsense | p.Q1701* | 20 | 54 | HGS | No | Yes |
| control | c.5101C>T | nonsense | p.Q1701* | 20 | 73 | N/A | N/A | N/A |
| case | c.5101C>T | nonsense | p Q1701* | 20 | 39 | LGS | N/A | N/A |
| case | c.5101C>T | nonsense | p Q1701* | 20 | 49 | clear cell | N/A | N/A |
| case | c.5340+1G>T | splicing | In frame 187 aa del | 20 | 76 | HGS | No | No |
| case | c.5791C>T | nonsense | p.R1931* | 22 | 53 | HGS | No | No |
| case | c.5791C>T | nonsense | p.R1931* | 22 | 76 | HGS | No | Yes |
| case | c.5791C>T | nonsense | p.R1931* | 22 | 59 | HGS | No | No |
| case | c.5791C>T | nonsense | p.R1931* | 22 | 61 | HGS | No | No |
| case | c.5791C>T | nonsense | p.R1931* | 22 | 67 | HGS | No | Yes |
| case | c.5791C>T | nonsense | p.R1931* | 22 | 57 | HGS | No | No |
| case | c.5791C>T | nonsense | p.R1931* | 22 | 65 | serous (grade unkown) | No | No |
| case | c.5791C>T | nonsense | p.R1931* | 22 | 62 | HGS | No | Yes |
| case | c.5791C>T | nonsense | p.R1931* | 22 | 54 | HGS | No | No |
| case | c.5791C>T | nonsense | p.R1931* | 22 | 48 | HGS | No | No |
| case | c.5791C>T | nonsense | p R1931* | 22 | 42 | HGS | N/A | N/A |
| case | c.5791C>T | nonsense | p R1931* | 22 | 67 | HGS | N/A | N/A |
| case | c.5791C>T | nonsense | p R1931* | 22 | 70 | HGS | N/A | N/A |
| control | c.5791C>T | nonsense | p.R1931* | 22 | 73 | N/A | N/A | N/A |
| control | c.5791C>T | nonsense | p.R1931* | 22 | 50 | N/A | N/A | N/A |
| control | c.5791C>T | nonsense | p.R1931* | 22 | 57 | N/A | N/A | N/A |
| control | c.5791C>T | nonsense | p.R1931* | 22 | 57 | N/A | N/A | N/A |
| control | c.5791C>T | nonsense | p.R1931* | 22 | 45 | N/A | N/A | N/A |
| control | c.5791C>T | nonsense | p.R1931* | 22 | 64 | N/A | N/A | N/A |
| control | c.5791C>T | nonsense | p.R1931* | 22 | 61 | N/A | N/A | N/A |
|  |  |  |  |  |  |  |  |  |
| **OC FH: family history of ovarian cancer**  **BC FH: family history of breast cancer** | | | | | | | | |
