# Supplementary material for: Germline whole exome sequencing and large-scale replication identifies FANCM as a likely high grade serous ovarian cancer susceptibility gene
Source: Oncotarget. 2017 Mar 3;8(31):50930–40. doi: 10.18632/oncotarget.15871 (PMC5584218; doi:10.18632/oncotarget.15871)
Supplement: Supplementary file 7 [file oncotarget-08-50930-s007.docx]

**Supplementary Table 7:** Putative pathogenic missense variants identified in each gene based on *in silico* software programs SIFT, Polyphen-2 and Provean

| **cDNA position** | **Protein** | **Damaging*** | **Controls** | **Cases** | **Total** |
| --- | --- | --- | --- | --- | --- |
| **APEX1** |  |  |  |  |  |
| c.56C>G | p.T19R | No | 0 | 1 | 1 |
| c.62C>T | p.P21L | No | 3 | 1 | 4 |
| c.103A>C | p.K35Q | No | 4 | 1 | 5 |
| c.128C>T | p.A43V | No | 1 | 0 | 1 |
| c.190A>G | p.I64V | No | 1 | 1 | 2 |
| c.286G>A | p.E96K | Yes | 1 | 0 | 1 |
| c.319G>A | p.E107K | Yes | 1 | 0 | 1 |
| c.385A>G | p.S129G | No | 0 | 1 | 1 |
| c.407G>A | p.R136H | No | 1 | 0 | 1 |
| c.413G>A | p.C138Y | No | 1 | 1 | 2 |
| c.442G>A | p.D148N | No | 1 | 0 | 1 |
| c.455A>C | p.D152A | Yes | 1 | 0 | 1 |
| c.467G>A | p.R156Q | Yes | 0 | 1 | 1 |
| c.542G>A | p.R181Q | No | 0 | 1 | 1 |
| c.560G>A | p.R187H | Yes | 3 | 4 | 7 |
| c.577C>T | p.R193C | No | 0 | 1 | 1 |
| c.578G>C | p.R193P | Yes | 0 | 1 | 1 |
| c.578G>A | p.R193H | No | 1 | 1 | 2 |
| c.709C>T | p.R237C | Yes | 1 | 0 | 1 |
| c.743C>T | p.P248L | No | 1 | 1 | 2 |
| c.756C>A | p.S252R | Yes | 0 | 1 | 1 |
| c.808A>G | p.M270V | No | 0 | 1 | 1 |
| c.827A>G | p.K276R | No | 0 | 1 | 1 |
| c.916G>A | p.G306S | Yes | 0 | 1 | 1 |
| c.920G>A | p.S307N | Yes | 1 | 0 | 1 |
| c.931C>A | p.P311T | Yes | 0 | 1 | 1 |
| **APLF** |  |  |  |  |  |
| c.28C>T | p.R10W | No | 2 | 1 | 3 |
| c.31G>A | p.D11N | No | 1 | 0 | 1 |
| c.41C>T | p.P14L | Yes | 0 | 1 | 1 |
| c.56C>T | p.A19V | No | 0 | 1 | 1 |
| c.58C>T | p.P20S | No | 0 | 1 | 1 |
| c.86C>T | p.P29L | Yes | 0 | 1 | 1 |
| c.88C>A | p.L30M | Yes | 1 | 0 | 1 |
| c.125A>T | p.H42L | Yes | 18 | 28 | 46 |
| c.245A>G | p.Y82C | No | 39 | 58 | 97 |
| c.326T>C | p.M109T | No | 0 | 1 | 1 |
| c.413A>G | p.H138R | No | 1 | 0 | 1 |
| c.428C>T | p.A143V | No | 1 | 0 | 1 |
| c.505A>G | p.N169D | No | 2 | 4 | 6 |
| c.512A>G | p.D171G | No | 1 | 0 | 1 |
| c.518A>G | p.N173S | No | 1 | 0 | 1 |
| c.541G>A | p.E181K | Yes | 0 | 1 | 1 |
| c.545G>A | p.R182K | Yes | 2 | 3 | 5 |
| c.555C>G | p.I185M | Yes | 0 | 1 | 1 |
| c.575C>T | p.A192V | No | 0 | 2 | 2 |
| c.631A>G | p.I211V | No | 0 | 1 | 1 |
| c.666T>A | p.D222E | No | 0 | 2 | 2 |
| c.793A>G | p.I265V | No | 0 | 1 | 1 |
| c.808A>G | p.M270V | No | 0 | 1 | 1 |
| c.847G>A | p.E283K | No | 0 | 1 | 1 |
| c.890C>A | p.P297Q | Yes | 1 | 0 | 1 |
| c.937A>G | p.R313G | No | 0 | 1 | 1 |
| c.949A>G | p.K317E | No | 0 | 1 | 1 |
| c.974C>G | p.S325C | Yes | 1 | 0 | 1 |
| c.1016A>T | p.E339V | Yes | 1 | 0 | 1 |
| c.1073A>T | p.H358L | No | 1 | 0 | 1 |
| c.1130C>T | p.T377I | No | 0 | 3 | 3 |
| c.1142A>G | p.Y381C | Yes | 2 | 3 | 5 |
| c.1276T>C | p.S426P | Yes | 4 | 0 | 4 |
| c.1427A>G | p.Y476C | No | 1 | 0 | 1 |
| c.1439A>G | p.D480G | Yes | 0 | 1 | 1 |
| c.1480A>G | p.K494E | No | 0 | 1 | 1 |
| c.1509A>T | p.E503D | No | 1 | 0 | 1 |
| c.1517G>A | p.R506K | No | 1 | 1 | 2 |
| c.1519T>C | p.F507L | Yes | 0 | 1 | 1 |
| **APTX** |  |  |  |  |  |
| c.971A>T | p.Q324L | No | 23 | 19 | 42 |
| c.946C>T | p.P316S | Yes | 0 | 1 | 1 |
| c.926T>C | p.M309T | No | 0 | 1 | 1 |
| c.742T>A | p.L248M | No | 10 | 23 | 33 |
| c.740G>A | p.R247Q | No | 2 | 1 | 3 |
| c.671T>C | p.L224P | Yes | 1 | 0 | 1 |
| c.617C>T | p.P206L | Yes | 0 | 1 | 1 |
| c.587C>T | p.P196L | Yes | 1 | 1 | 2 |
| c.553G>A | p.D185N | Yes | 0 | 1 | 1 |
| c.457A>G | p.K153E | No | 1 | 0 | 1 |
| c.431C>A | p.S144Y | No | 55 | 78 | 133 |
| c.430T>C | p.S144P | No | 0 | 1 | 1 |
| c.426C>G | p.S142R | No | 0 | 2 | 2 |
| c.425G>A | p.S142N | No | 0 | 1 | 1 |
| c.370A>G | p.I124V | No | 1 | 0 | 1 |
| c.315G>T | p.K105N | No | 0 | 1 | 1 |
| c.280T>A | p.Y94N | No | 0 | 1 | 1 |
| c.262C>T | p.H88Y | No | 1 | 0 | 1 |
| c.199A>G | p.S67G | No | 0 | 1 | 1 |
| c.191A>G | p.N64S | No | 0 | 1 | 1 |
| c.170A>G | p.K57R | No | 1 | 0 | 1 |
| c.85C>A | p.R29S | Yes | 0 | 1 | 1 |
| c.80T>C | p.I27T | No | 0 | 4 | 4 |
| c.38G>A | p.R13Q | No | 1 | 3 | 4 |
| c.18G>T | p.W6C | Yes | 9 | 13 | 22 |
| c.8G>A | p.R3Q | No | 1 | 0 | 1 |
| **EME1** |  |  |  |  |  |
| c.15G>T | p.K5N | Yes | 2 | 2 | 4 |
| c.19T>C | p.S7P | No | 1 | 0 | 1 |
| c.38G>C | p.G13A | No | 3 | 1 | 4 |
| c.48C>A | p.D16E | Yes | 0 | 1 | 1 |
| c.65C>T | p.T22I | Yes | 0 | 2 | 2 |
| c.119G>A | p.R40K | No | 1 | 1 | 2 |
| c.124G>A | p.E42K | No | 1 | 0 | 1 |
| c.124G>C | p.E42Q | No | 0 | 1 | 1 |
| c.145A>G | p.I49V | No | 6 | 3 | 9 |
| c.210A>G | p.I70M | No | 0 | 1 | 1 |
| c.267T>G | p.D89E | No | 0 | 1 | 1 |
| c.275A>C | p.E92A | No | 0 | 1 | 1 |
| c.340G>A | p.D114N | No | 5 | 12 | 17 |
| c.389G>A | p.G130D | No | 1 | 0 | 1 |
| c.481C>G | p.L161V | No | 2 | 1 | 3 |
| c.543C>A | p.S181R | No | 1 | 0 | 1 |
| c.567T>A | p.N189K | No | 8 | 11 | 19 |
| c.604A>G | p.S202G | No | 1 | 0 | 1 |
| c.612G>T | p.K204N | Yes | 1 | 0 | 1 |
| c.782T>C | p.L261S | Yes | 1 | 0 | 1 |
| c.787A>T | p.M263L | No | 0 | 1 | 1 |
| c.824C>T | p.T275I | Yes | 0 | 1 | 1 |
| c.836G>A | p.R279H | No | 0 | 2 | 2 |
| c.845T>C | p.I282T | Yes | 1 | 0 | 1 |
| c.950G>A | p.R317Q | No | 0 | 1 | 1 |
| c.976G>A | p.D326N | No | 1 | 0 | 1 |
| c.996C>G | p.S332R | No | 0 | 1 | 1 |
| c.1039G>A | p.V347I | No | 1 | 0 | 1 |
| c.1061C>T | p.T354I | No | 0 | 2 | 2 |
| c.1103A>T | p.K368I | Yes | 0 | 1 | 1 |
| c.1243A>G | p.M415V | No | 1 | 0 | 1 |
| c.1276G>A | p.V426M | No | 0 | 1 | 1 |
| c.1297G>C | p.E433Q | No | 0 | 1 | 1 |
| c.1306G>A | p.A436T | No | 8 | 6 | 14 |
| c.1341C>G | p.D447E | No | 1 | 0 | 1 |
| c.1394G>A | p.R465Q | No | 2 | 0 | 2 |
| c.1435G>C | p.A479P | Yes | 9 | 5 | 14 |
| c.1474G>A | p.A492T | No | 2 | 0 | 2 |
| c.1541T>C | p.V514A | No | 1 | 0 | 1 |
| c.1558C>T | p.P520S | Yes | 1 | 0 | 1 |
| c.1609C>G | p.Q537E | No | 1 | 0 | 1 |
| c.1636C>T | p.R546C | Yes | 6 | 2 | 8 |
| c.1637G>A | p.R546H | No | 0 | 1 | 1 |
| c.1640G>C | p.R547P | Yes | 2 | 0 | 2 |
| c.1687T>A | p.S563T | Yes | 0 | 1 | 1 |
| c.1693C>T | p.R565C | Yes | 3 | 0 | 3 |
| c.1727A>G | p.H576R | No | 0 | 1 | 1 |
| **FANCL** |  |  |  |  |  |
| c.1091G>A | p.C364Y | Yes | 1 | 0 | 1 |
| c.1087G>A | p.E363K | No | 1 | 0 | 1 |
| c.1067G>A | p.S356N | No | 0 | 2 | 2 |
| c.1066A>G | p.S356G | Yes | 1 | 0 | 1 |
| c.980A>G | p.Q327R | No | 1 | 0 | 1 |
| c.978T>A | p.D326E | Yes | 1 | 0 | 1 |
| c.877T>G | p.L293V | No | 1 | 0 | 1 |
| c.834G>T | p.L278F | No | 2 | 1 | 3 |
| c.825C>A | p.N275K | No | 2 | 1 | 3 |
| c.824A>C | p.N275T | Yes | 1 | 0 | 1 |
| c.754A>G | p.M252V | No | 1 | 0 | 1 |
| c.733G>A | p.V245I | No | 1 | 0 | 1 |
| c.728T>C | p.I243T | Yes | 1 | 0 | 1 |
| c.722T>A | p.I241K | Yes | 1 | 0 | 1 |
| c.719C>A | p.S240Y | Yes | 1 | 1 | 2 |
| c.710A>G | p.N237S | No | 0 | 1 | 1 |
| c.692G>A | p.R231H | Yes | 1 | 0 | 1 |
| c.685A>G | p.T229A | No | 5 | 0 | 5 |
| c.664G>C | p.E222Q | No | 1 | 1 | 2 |
| c.655C>G | p.L219V | No | 0 | 1 | 1 |
| c.558C>A | p.S186R | Yes | 1 | 0 | 1 |
| c.523G>A | p.A175T | No | 0 | 1 | 1 |
| c.493T>G | p.Y165D | No | 0 | 1 | 1 |
| c.452C>A | p.T151N | Yes | 1 | 0 | 1 |
| c.354A>G | p.I118M | No | 1 | 0 | 1 |
| c.332A>G | p.Y111C | Yes | 1 | 0 | 1 |
| c.317C>T | p.P106L | Yes | 1 | 0 | 1 |
| c.252G>A | p.M84I | No | 0 | 1 | 1 |
| c.203G>C | p.R68P | No | 5 | 3 | 8 |
| c.191G>T | p.S64I | No | 0 | 1 | 1 |
| c.112C>T | p.L38F | Yes | 26 | 22 | 48 |
| c.108C>G | p.F36L | Yes | 2 | 0 | 2 |
| c.38C>A | p.P13H | Yes | 0 | 1 | 1 |
| c.19A>G | p.S7G | No | 1 | 0 | 1 |
| c.2T>C | p.M1T | No | 0 | 1 | 1 |
| **FANCM** |  |  |  |  |  |
| c.43A>G | p.S15G | No | 1 | 0 | 1 |
| c.53G>A | p.R18Q | No | 1 | 1 | 2 |
| c.59C>G | p.S20C | No | 1 | 0 | 1 |
| c.68C>A | p.P23Q | No | 0 | 1 | 1 |
| c.163G>A | p.D55N | Yes | 3 | 7 | 10 |
| c.171G>C | p.L57F | No | 29 | 34 | 63 |
| c.269C>T | p.P90L | Yes | 3 | 4 | 7 |
| c.356T>A | p.F119Y | Yes | 1 | 0 | 1 |
| c.488C>T | p.S163F | Yes | 0 | 1 | 1 |
| c.497C>A | p.A166D | Yes | 1 | 0 | 1 |
| c.504G>C | p.M168I | Yes | 1 | 0 | 1 |
| c.527C>T | p.T176I | No | 34 | 32 | 66 |
| c.538A>G | p.I180V | No | 0 | 3 | 3 |
| c.545G>C | p.C182S | No | 0 | 1 | 1 |
| c.547A>C | p.S183R | Yes | 4 | 3 | 7 |
| c.641A>G | p.D214G | Yes | 0 | 1 | 1 |
| c.650A>G | p.H217R | Yes | 1 | 0 | 1 |
| c.710A>G | p.N237S | No | 1 | 0 | 1 |
| c.752A>G | p.D251G | Yes | 0 | 1 | 1 |
| c.763G>A | p.V255M | Yes | 0 | 1 | 1 |
| c.775A>G | p.I259V | No | 1 | 2 | 3 |
| c.808C>T | p.R270C | Yes | 0 | 2 | 2 |
| c.874C>T | p.P292S | Yes | 0 | 1 | 1 |
| c.926A>C | p.E309A | Yes | 1 | 0 | 1 |
| c.938G>A | p.R313H | No | 1 | 0 | 1 |
| c.946A>G | p.I316V | No | 0 | 1 | 1 |
| c.950A>G | p.Q317R | No | 1 | 0 | 1 |
| c.1017T>A | p.D339E | Yes | 0 | 1 | 1 |
| c.1072G>A | p.E358K | Yes | 0 | 1 | 1 |
| c.1087A>G | p.I363V | No | 0 | 3 | 3 |
| c.1115A>G | p.E372G | Yes | 0 | 1 | 1 |
| c.1127A>G | p.Q376R | Yes | 0 | 1 | 1 |
| c.1192C>T | p.R398W | Yes | 0 | 1 | 1 |
| c.1222G>C | p.D408H | Yes | 1 | 1 | 2 |
| c.1237T>C | p.Y413H | Yes | 8 | 19 | 27 |
| c.1264C>T | p.R422C | No | 1 | 1 | 2 |
| c.1366G>A | p.V456I | No | 1 | 0 | 1 |
| c.1425_1427delTGA | p.475_476del | No | 0 | 1 | 1 |
| c.1438A>T | p.M480L | Yes | 0 | 1 | 1 |
| c.1439T>G | p.M480R | Yes | 0 | 1 | 1 |
| c.1440G>A | p.M480I | Yes | 0 | 1 | 1 |
| c.1457G>A | p.R486Q | Yes | 0 | 1 | 1 |
| c.1462A>G | p.S488G | Yes | 1 | 0 | 1 |
| c.1576C>G | p.L526V | No | 13 | 25 | 38 |
| c.1597C>T | p.R533C | Yes | 2 | 4 | 6 |
| c.1598G>A | p.R533H | Yes | 0 | 1 | 1 |
| c.1603G>A | p.G535S | Yes | 1 | 0 | 1 |
| c.1604G>A | p.G535D | Yes | 2 | 0 | 2 |
| c.1627A>G | p.T543A | Yes | 1 | 0 | 1 |
| c.1636G>A | p.G546S | Yes | 2 | 0 | 2 |
| c.1651G>T | p.D551Y | Yes | 0 | 1 | 1 |
| c.1660G>A | p.E554K | Yes | 0 | 1 | 1 |
| c.1667A>G | p.D556G | Yes | 1 | 1 | 2 |
| c.1703T>C | p.I568T | No | 0 | 1 | 1 |
| c.1729A>T | p.T577S | Yes | 0 | 1 | 1 |
| c.1741C>T | p.R581C | Yes | 1 | 1 | 2 |
| c.1742G>A | p.R581H | Yes | 0 | 1 | 1 |
| c.1756G>C | p.V586L | Yes | 0 | 1 | 1 |
| c.1760T>C | p.I587T | Yes | 2 | 0 | 2 |
| c.1775G>A | p.G592E | Yes | 1 | 0 | 1 |
| c.1814A>G | p.K605R | No | 0 | 1 | 1 |
| c.1846A>G | p.R616G | No | 0 | 1 | 1 |
| c.1849C>G | p.Q617E | No | 0 | 3 | 3 |
| c.1861T>C | p.F621L | No | 0 | 1 | 1 |
| c.1880G>A | p.R627Q | Yes | 1 | 0 | 1 |
| c.1880G>C | p.R627P | Yes | 0 | 1 | 1 |
| c.1918A>G | p.M640V | Yes | 0 | 1 | 1 |
| c.2084G>C | p.R695T | No | 0 | 1 | 1 |
| c.2182A>T | p.I728F | No | 0 | 1 | 1 |
| c.2190A>T | p.Q730H | Yes | 1 | 0 | 1 |
| c.2215T>C | p.W739R | Yes | 1 | 0 | 1 |
| c.2236A>G | p.T746A | Yes | 1 | 0 | 1 |
| c.2261G>A | p.R754Q | Yes | 0 | 1 | 1 |
| c.2267G>A | p.R756H | No | 0 | 3 | 3 |
| c.2302A>G | p.M768V | Yes | 0 | 1 | 1 |
| c.2330A>G | p.Y777C | Yes | 0 | 1 | 1 |
| c.2372C>A | p.T791N | No | 0 | 1 | 1 |
| c.2458A>G | p.N820D | No | 0 | 1 | 1 |
| c.2483C>T | p.S828L | No | 0 | 1 | 1 |
| c.2497G>A | p.D833N | No | 0 | 1 | 1 |
| c.2501A>G | p.E834G | Yes | 0 | 1 | 1 |
| c.2575A>G | p.K859E | No | 0 | 1 | 1 |
| c.2629T>C | p.S877P | No | 1 | 0 | 1 |
| c.2638A>G | p.N880D | No | 0 | 1 | 1 |
| c.2704G>A | p.D902N | No | 0 | 1 | 1 |
| c.2716A>T | p.I906F | No | 1 | 1 | 2 |
| c.2741A>G | p.E914G | No | 0 | 1 | 1 |
| c.2749A>G | p.I917V | No | 3 | 1 | 4 |
| c.2752A>G | p.K918E | No | 1 | 0 | 1 |
| c.2759C>T | p.P920L | No | 0 | 2 | 2 |
| c.2764G>A | p.V922M | No | 0 | 1 | 1 |
| c.2813C>T | p.A938V | No | 0 | 1 | 1 |
| c.2831C>T | p.S944F | Yes | 0 | 1 | 1 |
| c.2859A>C | p.K953N | No | 15 | 24 | 39 |
| c.2888T>C | p.F963S | No | 0 | 1 | 1 |
| c.2986T>C | p.Y996H | No | 0 | 1 | 1 |
| c.3016G>A | p.D1006N | No | 1 | 0 | 1 |
| c.3049C>T | p.L1017F | No | 0 | 1 | 1 |
| c.3062T>C | p.L1021P | No | 1 | 0 | 1 |
| c.3064T>C | p.F1022L | No | 0 | 1 | 1 |
| c.3113T>C | p.L1038P | No | 1 | 0 | 1 |
| c.3147A>C | p.L1049F | No | 1 | 0 | 1 |
| c.3175T>C | p.Y1059H | No | 1 | 0 | 1 |
| c.3293A>G | p.N1098S | No | 1 | 0 | 1 |
| c.3296G>A | p.R1099H | No | 5 | 2 | 7 |
| c.3350A>T | p.D1117V | Yes | 1 | 0 | 1 |
| c.3371C>A | p.P1124Q | No | 1 | 0 | 1 |
| c.3414A>T | p.E1138D | No | 0 | 1 | 1 |
| c.3436G>A | p.D1146N | No | 0 | 2 | 2 |
| c.3469G>A | p.E1157K | No | 0 | 1 | 1 |
| c.3557A>G | p.N1186S | No | 1 | 0 | 1 |
| c.3613G>A | p.D1205N | No | 0 | 1 | 1 |
| c.3658A>T | p.I1220F | No | 1 | 0 | 1 |
| c.3704G>T | p.G1235V | Yes | 2 | 1 | 3 |
| c.3731A>G | p.E1244G | No | 1 | 1 | 2 |
| c.3785G>A | p.R1262K | No | 1 | 0 | 1 |
| c.3827C>T | p.S1276L | No | 0 | 2 | 2 |
| c.3845C>A | p.P1282Q | No | 1 | 0 | 1 |
| c.3863A>G | p.N1288S | No | 2 | 3 | 5 |
| c.3869C>T | p.T1290I | No | 0 | 1 | 1 |
| c.3911A>G | p.N1304S | No | 0 | 1 | 1 |
| c.3935T>C | p.L1312P | No | 0 | 1 | 1 |
| c.3956A>G | p.E1319G | No | 0 | 1 | 1 |
| c.3959T>C | p.L1320S | No | 1 | 0 | 1 |
| c.3991C>T | p.P1331S | Yes | 0 | 1 | 1 |
| c.3992C>T | p.P1331L | No | 0 | 3 | 3 |
| c.3998A>C | p.Q1333P | No | 2 | 1 | 3 |
| c.4022T>C | p.L1341P | No | 0 | 1 | 1 |
| c.4084G>A | p.D1362N | No | 0 | 1 | 1 |
| c.4101A>C | p.K1367N | No | 0 | 1 | 1 |
| c.4147T>G | p.Y1383D | No | 1 | 0 | 1 |
| c.4216G>A | p.V1406I | No | 1 | 0 | 1 |
| c.4306A>G | p.N1436D | No | 0 | 1 | 1 |
| c.4366C>T | p.R1456C | No | 3 | 3 | 6 |
| c.4367G>A | p.R1456H | No | 0 | 2 | 2 |
| c.4465G>A | p.G1489R | No | 1 | 1 | 2 |
| c.4480G>T | p.G1494C | Yes | 1 | 0 | 1 |
| c.4489G>A | p.V1497I | No | 0 | 1 | 1 |
| c.4523C>G | p.A1508G | No | 2 | 0 | 2 |
| c.4556C>A | p.S1519Y | Yes | 0 | 1 | 1 |
| c.4606A>G | p.N1536D | No | 1 | 0 | 1 |
| c.4607A>G | p.N1536S | No | 0 | 1 | 1 |
| c.4627C>G | p.L1543V | No | 1 | 0 | 1 |
| c.4627C>T | p.L1543F | No | 1 | 2 | 3 |
| c.4709G>A | p.R1570H | No | 1 | 0 | 1 |
| c.4859A>G | p.E1620G | Yes | 0 | 1 | 1 |
| c.4872T>G | p.C1624W | Yes | 1 | 0 | 1 |
| c.4912A>G | p.S1638G | No | 1 | 1 | 2 |
| c.4933C>T | p.R1645C | Yes | 1 | 0 | 1 |
| c.4934G>A | p.R1645H | No | 0 | 1 | 1 |
| c.4946T>G | p.L1649R | Yes | 1 | 0 | 1 |
| c.5107C>G | p.H1703D | No | 1 | 2 | 3 |
| c.5108A>G | p.H1703R | No | 1 | 3 | 4 |
| c.5117A>C | p.N1706T | No | 0 | 1 | 1 |
| c.5144A>C | p.Q1715P | No | 0 | 1 | 1 |
| c.5155C>T | p.R1719C | No | 0 | 1 | 1 |
| c.5177C>T | p.P1726L | No | 0 | 2 | 2 |
| c.5183C>G | p.A1728G | No | 0 | 1 | 1 |
| c.5249C>T | p.P1750L | No | 1 | 0 | 1 |
| c.5263G>A | p.E1755K | No | 0 | 1 | 1 |
| c.5291C>T | p.T1764I | No | 1 | 0 | 1 |
| c.5309A>G | p.K1770R | No | 0 | 1 | 1 |
| c.5337G>T | p.Q1779H | No | 0 | 1 | 1 |
| c.5413A>G | p.T1805A | No | 0 | 1 | 1 |
| c.5440G>A | p.E1814K | No | 1 | 0 | 1 |
| c.5474A>G | p.H1825R | No | 0 | 1 | 1 |
| c.5545C>A | p.P1849T | Yes | 1 | 0 | 1 |
| c.5569G>A | p.V1857M | Yes | 9 | 7 | 16 |
| c.5578C>T | p.R1860C | Yes | 0 | 1 | 1 |
| c.5579G>A | p.R1860H | Yes | 0 | 1 | 1 |
| c.5656C>T | p.H1886Y | No | 3 | 3 | 6 |
| c.5679A>T | p.R1893S | Yes | 0 | 1 | 1 |
| c.5759G>C | p.S1920T | No | 0 | 1 | 1 |
| c.5770A>T | p.T1924S | No | 0 | 2 | 2 |
| c.5777T>C | p.I1926T | Yes | 1 | 0 | 1 |
| c.5782G>A | p.A1928T | No | 0 | 1 | 1 |
| c.5792G>A | p.R1931Q | No | 1 | 0 | 1 |
| c.5832G>T | p.L1944F | Yes | 2 | 0 | 2 |
| c.5866A>G | p.N1956D | Yes | 1 | 0 | 1 |
| c.5951A>T | p.Y1984F | Yes | 0 | 2 | 2 |
| c.6097C>A | p.Q2033K | No | 0 | 1 | 1 |
| c.6128G>A | p.R2043K | No | 0 | 1 | 1 |
| c.6143T>C | p.I2048T | No | 1 | 2 | 3 |
| **PARP2** |  |  |  |  |  |
| c.26C>A | p.T9N | No | 1 | 1 | 2 |
| c.41C>G | p.A14G | No | 1 | 0 | 1 |
| c.43A>G | p.R15G | No | 5 | 9 | 14 |
| c.67G>A | p.V23I | No | 0 | 1 | 1 |
| c.148A>G | p.M50V | No | 0 | 1 | 1 |
| c.197A>G | p.Q66R | No | 2 | 0 | 2 |
| c.440T>G | p.V147G | Yes | 1 | 0 | 1 |
| c.449G>A | p.R150K | Yes | 0 | 1 | 1 |
| c.454G>A | p.G152S | Yes | 0 | 1 | 1 |
| c.464G>A | p.G155E | Yes | 1 | 0 | 1 |
| c.475C>A | p.Q159K | Yes | 1 | 0 | 1 |
| c.482G>A | p.S161N | No | 4 | 1 | 5 |
| c.487G>A | p.V163M | No | 0 | 1 | 1 |
| c.503A>G | p.N168S | No | 0 | 1 | 1 |
| c.509A>G | p.N170S | No | 0 | 1 | 1 |
| c.540C>G | p.F180L | Yes | 0 | 2 | 2 |
| c.551C>T | p.T184M | Yes | 1 | 0 | 1 |
| c.610C>G | p.L204V | No | 2 | 0 | 2 |
| c.652A>G | p.T218A | No | 1 | 0 | 1 |
| c.785C>A | p.T262N | Yes | 0 | 1 | 1 |
| c.799C>T | p.L267F | Yes | 1 | 0 | 1 |
| c.859G>A | p.D287N | No | 0 | 1 | 1 |
| c.868C>T | p.R290W | Yes | 1 | 1 | 2 |
| c.880C>T | p.H294Y | No | 1 | 1 | 2 |
| c.895A>C | p.M299L | No | 0 | 1 | 1 |
| c.947G>A | p.R316H | Yes | 1 | 1 | 2 |
| c.950C>T | p.T317I | No | 1 | 0 | 1 |
| c.965G>A | p.R322Q | No | 4 | 6 | 10 |
| c.1031T>C | p.I344T | Yes | 0 | 1 | 1 |
| c.1043A>G | p.K348R | No | 0 | 1 | 1 |
| c.1069C>G | p.P357A | Yes | 0 | 1 | 1 |
| c.1072T>A | p.L358M | No | 0 | 1 | 1 |
| c.1081C>T | p.H361Y | Yes | 0 | 2 | 2 |
| c.1084T>G | p.Y362D | Yes | 0 | 1 | 1 |
| c.1108C>T | p.R370C | Yes | 1 | 1 | 2 |
| c.1109G>A | p.R370H | No | 0 | 1 | 1 |
| c.1229A>G | p.D410G | No | 1 | 0 | 1 |
| c.1234G>A | p.E412K | Yes | 3 | 2 | 5 |
| c.1331G>A | p.R444Q | Yes | 1 | 1 | 2 |
| c.1398G>T | p.M466I | Yes | 0 | 1 | 1 |
| c.1399T>C | p.S467P | Yes | 1 | 0 | 1 |
| c.1442A>G | p.N481S | No | 0 | 2 | 2 |
| c.1445C>T | p.T482I | No | 1 | 0 | 1 |
| c.1468G>A | p.V490I | Yes | 0 | 1 | 1 |
| c.1600A>G | p.S534G | Yes | 0 | 1 | 1 |
| c.1638T>G | p.I546M | No | 1 | 0 | 1 |
| c.1655A>G | p.Y552C | No | 0 | 1 | 1 |
| c.1670A>G | p.N557S | Yes | 1 | 0 | 1 |
| c.1691C>G | p.P564R | Yes | 1 | 0 | 1 |
| c.1708C>T | p.R570W | Yes | 0 | 1 | 1 |
| **PARP3** |  |  |  |  |  |
| c.51G>T | p.Q17H | No | 0 | 1 | 1 |
| c.53C>A | p.T18N | No | 0 | 3 | 3 |
| c.56A>G | p.E19G | No | 0 | 8 | 8 |
| c.57G>C | p.E19D | No | 0 | 4 | 4 |
| c.58G>C | p.G20R | No | 2 | 6 | 8 |
| c.59G>C | p.G20A | No | 3 | 10 | 13 |
| c.69G>C | p.K23N | No | 2 | 0 | 2 |
| c.79C>T | p.R27W | No | 1 | 2 | 3 |
| c.83A>C | p.Q28P | No | 0 | 1 | 1 |
| c.92G>C | p.R31T | No | 1 | 0 | 1 |
| c.110G>A | p.R37H | No | 1 | 0 | 1 |
| c.119C>G | p.A40G | No | 1 | 0 | 1 |
| c.142G>T | p.A48S | No | 2 | 1 | 3 |
| c.147G>T | p.E49D | No | 1 | 0 | 1 |
| c.151C>A | p.R51S | No | 1 | 0 | 1 |
| c.161G>T | p.R54L | No | 1 | 0 | 1 |
| c.227C>T | p.T76I | No | 1 | 0 | 1 |
| c.244A>T | p.I82F | Yes | 0 | 1 | 1 |
| c.260A>G | p.N87S | Yes | 0 | 1 | 1 |
| c.293G>A | p.S98N | No | 1 | 1 | 2 |
| c.319C>T | p.H107Y | No | 1 | 0 | 1 |
| c.322T>C | p.W108R | Yes | 0 | 1 | 1 |
| c.325G>A | p.G109S | Yes | 1 | 0 | 1 |
| c.379G>C | p.D127H | No | 0 | 1 | 1 |
| c.401A>T | p.K134M | Yes | 0 | 1 | 1 |
| c.409C>T | p.R137W | No | 1 | 3 | 4 |
| c.410G>A | p.R137Q | No | 0 | 1 | 1 |
| c.439C>T | p.R147W | Yes | 4 | 13 | 17 |
| c.517G>A | p.V173M | No | 0 | 1 | 1 |
| c.556C>T | p.R186W | No | 2 | 0 | 2 |
| c.637A>G | p.M213V | Yes | 0 | 1 | 1 |
| c.652C>G | p.L218V | No | 1 | 0 | 1 |
| c.682C>A | p.L228M | No | 0 | 1 | 1 |
| c.689A>G | p.K230R | No | 1 | 0 | 1 |
| c.745G>A | p.G249S | No | 1 | 0 | 1 |
| c.752C>T | p.T251M | No | 0 | 1 | 1 |
| c.757G>A | p.G253S | No | 1 | 0 | 1 |
| c.789C>G | p.H263Q | No | 0 | 1 | 1 |
| c.799G>A | p.V267I | No | 2 | 0 | 2 |
| c.827A>G | p.Q276R | No | 5 | 2 | 7 |
| c.898G>C | p.E300Q | No | 0 | 1 | 1 |
| c.941C>T | p.T314M | No | 0 | 1 | 1 |
| c.946G>C | p.E316Q | No | 0 | 1 | 1 |
| c.1103T>C | p.V368A | No | 2 | 2 | 4 |
| c.1138C>T | p.H380Y | Yes | 0 | 1 | 1 |
| c.1174G>A | p.G392S | Yes | 4 | 0 | 4 |
| c.1189G>A | p.V397M | No | 1 | 0 | 1 |
| c.1198G>A | p.A400T | Yes | 0 | 1 | 1 |
| c.1204C>T | p.L402F | Yes | 1 | 0 | 1 |
| c.1219C>T | p.R407C | Yes | 1 | 0 | 1 |
| c.1220G>A | p.R407H | Yes | 1 | 0 | 1 |
| c.1243C>T | p.R415C | Yes | 1 | 0 | 1 |
| c.1244G>A | p.R415H | Yes | 0 | 1 | 1 |
| c.1316G>T | p.G439V | No | 1 | 0 | 1 |
| c.1324C>G | p.H442D | No | 3 | 2 | 5 |
| c.1430T>C | p.I477T | No | 1 | 0 | 1 |
| c.1436G>A | p.R479Q | No | 1 | 0 | 1 |
| c.1447G>A | p.E483K | Yes | 1 | 0 | 1 |
| c.1505C>A | p.P502H | Yes | 1 | 0 | 1 |
| c.1561G>A | p.E521K | Yes | 1 | 0 | 1 |
| c.1592G>A | p.R531H | No | 1 | 0 | 1 |
| c.1597C>T | p.R533C | Yes | 1 | 0 | 1 |
| **POLN** |  |  |  |  |  |
| c.2684C>T | p.S895L | No | 1 | 0 | 1 |
| c.2672C>T | p.P891L | No | 2 | 0 | 2 |
| c.2671C>T | p.P891S | No | 1 | 0 | 1 |
| c.2648C>A | p.S883Y | No | 0 | 1 | 1 |
| c.2626A>T | p.N876Y | No | 0 | 3 | 3 |
| c.2609G>A | p.R870H | No | 0 | 1 | 1 |
| c.2609G>T | p.R870L | No | 1 | 1 | 2 |
| c.2590C>T | p.P864S | No | 1 | 0 | 1 |
| c.2548C>T | p.R850C | Yes | 0 | 2 | 2 |
| c.2435C>A | p.P812Q | No | 0 | 1 | 1 |
| c.2435C>T | p.P812L | No | 0 | 1 | 1 |
| c.2425G>A | p.V809M | No | 1 | 0 | 1 |
| c.2410G>A | p.E804K | Yes | 0 | 1 | 1 |
| c.2381C>T | p.T794M | Yes | 0 | 1 | 1 |
| c.2231C>A | p.P744H | Yes | 1 | 0 | 1 |
| c.2230C>A | p.P744T | No | 1 | 1 | 2 |
| c.2219G>C | p.G740A | Yes | 1 | 0 | 1 |
| c.2218G>A | p.G740S | Yes | 0 | 1 | 1 |
| c.2201G>A | p.C734Y | No | 1 | 0 | 1 |
| c.2198G>T | p.G733V | Yes | 1 | 1 | 2 |
| c.2194A>G | p.T732A | No | 0 | 2 | 2 |
| c.2168G>A | p.R723Q | No | 7 | 2 | 9 |
| c.2153T>C | p.I718T | Yes | 0 | 1 | 1 |
| c.2133T>G | p.F711L | Yes | 2 | 0 | 2 |
| c.2075G>A | p.R692Q | No | 1 | 2 | 3 |
| c.2057A>G | p.Y686C | Yes | 0 | 1 | 1 |
| c.2048C>T | p.A683V | Yes | 0 | 1 | 1 |
| c.2041G>A | p.V681M | No | 3 | 2 | 5 |
| c.2026C>A | p.Q676K | No | 0 | 1 | 1 |
| c.1996G>A | p.V666M | No | 1 | 0 | 1 |
| c.1939G>A | p.E647K | No | 1 | 0 | 1 |
| c.1916C>A | p.P639Q | Yes | 1 | 0 | 1 |
| c.1886T>C | p.L629S | Yes | 0 | 1 | 1 |
| c.1862C>T | p.A621V | Yes | 1 | 0 | 1 |
| c.1852A>G | p.T618A | No | 6 | 5 | 11 |
| c.1803G>C | p.K601N | No | 0 | 1 | 1 |
| c.1754A>G | p.H585R | Yes | 1 | 0 | 1 |
| c.1730C>T | p.P577L | Yes | 0 | 1 | 1 |
| c.1729C>A | p.P577T | Yes | 0 | 1 | 1 |
| c.1615C>T | p.H539Y | No | 1 | 0 | 1 |
| c.1547A>G | p.E516G | No | 0 | 1 | 1 |
| c.1520G>A | p.G507E | No | 1 | 0 | 1 |
| c.1520G>C | p.G507A | No | 0 | 1 | 1 |
| c.1517C>T | p.T506M | Yes | 1 | 1 | 2 |
| c.1454G>T | p.R485L | Yes | 0 | 1 | 1 |
| c.1436C>T | p.T479M | No | 8 | 2 | 10 |
| c.1403C>T | p.A468V | No | 0 | 1 | 1 |
| c.1399G>A | p.E467K | Yes | 1 | 0 | 1 |
| c.1394A>G | p.E465G | Yes | 0 | 1 | 1 |
| c.1379G>A | p.R460H | No | 0 | 1 | 1 |
| c.1378C>T | p.R460C | Yes | 0 | 2 | 2 |
| c.1348A>G | p.M450V | No | 1 | 0 | 1 |
| c.1264C>A | p.Q422K | No | 0 | 1 | 1 |
| c.1184A>G | p.Q395R | No | 1 | 1 | 2 |
| c.1159A>C | p.N387H | No | 1 | 0 | 1 |
| c.1075A>G | p.S359G | No | 1 | 0 | 1 |
| c.1057G>C | p.A353P | Yes | 1 | 0 | 1 |
| c.937A>G | p.K313E | No | 0 | 1 | 1 |
| c.928A>G | p.M310V | No | 0 | 1 | 1 |
| c.928A>C | p.M310L | No | 51 | 39 | 90 |
| c.913G>A | p.V305M | No | 1 | 1 | 2 |
| c.907C>T | p.R303W | No | 0 | 1 | 1 |
| c.826T>C | p.S276P | No | 0 | 1 | 1 |
| c.793C>T | p.P265S | No | 22 | 24 | 46 |
| c.734G>A | p.R245K | No | 0 | 1 | 1 |
| c.677T>C | p.M226T | No | 0 | 1 | 1 |
| c.656C>T | p.A219V | No | 0 | 1 | 1 |
| c.575C>T | p.A192V | No | 1 | 0 | 1 |
| c.572G>A | p.G191E | No | 1 | 0 | 1 |
| c.548G>T | p.G183V | No | 4 | 0 | 4 |
| c.488A>C | p.N163T | No | 1 | 0 | 1 |
| c.470A>G | p.K157R | No | 1 | 0 | 1 |
| c.452G>A | p.S151N | No | 2 | 0 | 2 |
| c.370C>G | p.Q124E | No | 1 | 1 | 2 |
| c.353T>C | p.L118S | No | 1 | 1 | 2 |
| c.319A>G | p.K107E | No | 0 | 2 | 2 |
| c.290C>T | p.T97I | No | 1 | 0 | 1 |
| c.254C>T | p.A85V | No | 0 | 1 | 1 |
| c.227T>C | p.L76S | No | 0 | 1 | 1 |
| c.167A>G | p.Y56C | No | 0 | 1 | 1 |
| c.151A>G | p.K51E | No | 0 | 1 | 1 |
| c.149A>C | p.N50T | No | 0 | 1 | 1 |
| c.83T>C | p.M28T | No | 1 | 0 | 1 |
| c.47C>T | p.P16L | Yes | 1 | 1 | 2 |
| **RAD54L** |  |  |  |  |  |
| c.65A>T | p.D22V | No | 1 | 0 | 1 |
| c.95C>G | p.P32R | No | 0 | 1 | 1 |
| c.145T>C | p.S49P | No | 0 | 1 | 1 |
| c.160C>T | p.P54S | No | 1 | 0 | 1 |
| c.223C>G | p.R75G | No | 0 | 1 | 1 |
| c.227G>C | p.S76T | No | 1 | 0 | 1 |
| c.259C>G | p.P87A | No | 1 | 0 | 1 |
| c.367C>T | p.P123S | Yes | 0 | 1 | 1 |
| c.374C>T | p.P125L | No | 3 | 0 | 3 |
| c.473G>C | p.R158T | Yes | 0 | 3 | 3 |
| c.515G>A | p.R172H | No | 0 | 1 | 1 |
| c.569C>T | p.T190M | Yes | 1 | 1 | 2 |
| c.604C>T | p.R202C | Yes | 24 | 17 | 41 |
| c.620G>A | p.C207Y | Yes | 0 | 1 | 1 |
| c.785G>T | p.R262L | No | 2 | 1 | 3 |
| c.796G>C | p.V266L | No | 0 | 1 | 1 |
| c.883T>C | p.C295R | Yes | 1 | 0 | 1 |
| c.901C>T | p.L301F | Yes | 0 | 1 | 1 |
| c.955C>T | p.R319W | Yes | 0 | 1 | 1 |
| c.956G>A | p.R319Q | Yes | 0 | 1 | 1 |
| c.1021T>C | p.F341L | Yes | 0 | 1 | 1 |
| c.1094G>A | p.R365Q | No | 0 | 1 | 1 |
| c.1138C>T | p.R380W | Yes | 1 | 1 | 2 |
| c.1298C>T | p.P433L | No | 3 | 0 | 3 |
| c.1316A>G | p.E439G | No | 0 | 1 | 1 |
| c.1351A>G | p.T451A | Yes | 0 | 1 | 1 |
| c.1389C>G | p.I463M | Yes | 1 | 1 | 2 |
| c.1492A>G | p.M498V | No | 1 | 0 | 1 |
| c.1517C>T | p.A506V | Yes | 0 | 1 | 1 |
| c.1532G>A | p.R511H | No | 0 | 1 | 1 |
| c.1542C>A | p.D514E | Yes | 0 | 1 | 1 |
| c.1592A>C | p.K531T | Yes | 1 | 0 | 1 |
| c.1598G>A | p.C533Y | Yes | 1 | 0 | 1 |
| c.1600C>T | p.R534C | Yes | 0 | 1 | 1 |
| c.1601G>C | p.R534P | Yes | 0 | 1 | 1 |
| c.1637C>T | p.T546M | Yes | 2 | 0 | 2 |
| c.1655G>A | p.R552Q | Yes | 2 | 2 | 4 |
| c.1679A>G | p.N560S | Yes | 0 | 1 | 1 |
| c.1748T>C | p.I583T | Yes | 1 | 0 | 1 |
| c.1759C>T | p.R587W | Yes | 4 | 8 | 12 |
| c.1849T>A | p.Y617N | Yes | 0 | 1 | 1 |
| c.1850A>G | p.Y617C | Yes | 1 | 0 | 1 |
| c.1858C>T | p.R620C | Yes | 1 | 0 | 1 |
| c.1934T>G | p.V645G | Yes | 1 | 0 | 1 |
| c.1970C>G | p.S657C | Yes | 3 | 2 | 5 |
| c.1996A>G | p.I666V | No | 1 | 0 | 1 |
| c.2063G>A | p.R688H | No | 1 | 0 | 1 |
| c.2072G>A | p.R691Q | No | 4 | 1 | 5 |
| c.2080C>G | p.P694A | No | 1 | 0 | 1 |
| c.2080C>T | p.P694S | No | 1 | 0 | 1 |
| c.2126G>A | p.C709Y | No | 1 | 0 | 1 |
| c.2129C>G | p.T710S | No | 1 | 0 | 1 |
| c.2138G>T | p.W713L | No | 1 | 0 | 1 |
| c.2213G>A | p.R738H | No | 33 | 32 | 65 |
| **SMUG1** |  |  |  |  |  |
| c.770A>G | p.E257G | No | 1 | 0 | 1 |
| c.700G>A | p.V234M | Yes | 0 | 1 | 1 |
| c.698A>G | p.Q233R | No | 0 | 1 | 1 |
| c.667C>T | p.R223W | Yes | 0 | 1 | 1 |
| c.659G>A | p.R220Q | Yes | 1 | 0 | 1 |
| c.622C>T | p.R208W | Yes | 1 | 0 | 1 |
| c.605T>G | p.V202G | Yes | 0 | 1 | 1 |
| c.547C>T | p.P183S | Yes | 0 | 1 | 1 |
| c.527A>G | p.N176S | Yes | 0 | 1 | 1 |
| c.523C>T | p.R175C | Yes | 1 | 0 | 1 |
| c.466T>C | p.F156L | Yes | 1 | 0 | 1 |
| c.313C>T | p.R105W | Yes | 5 | 15 | 20 |
| c.256C>G | p.P86A | Yes | 1 | 0 | 1 |
| c.218G>A | p.C73Y | Yes | 0 | 1 | 1 |
| c.212G>A | p.R71H | Yes | 0 | 1 | 1 |
| c.169C>A | p.P57T | Yes | 1 | 1 | 2 |
| c.143C>T | p.S48L | Yes | 3 | 5 | 8 |
| c.106C>T | p.L36F | No | 1 | 0 | 1 |
| c.67T>A | p.C23S | No | 0 | 1 | 1 |
| c.44G>T | p.G15V | No | 3 | 1 | 4 |
| c.2T>C | p.M1T | No | 0 | 1 | 1 |
| *Predicted damaging by 2 out of three *in silico* software programs: SIFT, Polyphen-2 and Provean | | | | | |
